# Cysteine pattern barcoding-based dataset filtration enhances the machine learning-assisted interpretation of *Conus* venom peptide therapeutics

**DOI:** 10.1371/journal.pone.0327578

**Published:** 2025-07-11

**Authors:** Rimsha Bibi, Noshaba Qasmi, Sajid Rashid

**Affiliations:** National Center for Bioinformatics, Quaid-i-Azam University, Pakistan; Instituto Butantan, BRAZIL

## Abstract

Crude cone snail venom is a rich source of bioactive compounds with significant therapeutic potential. In this study, we conducted a comprehensive analysis of 5,985 cone snail peptides across 82 *Conus* species to identify unique cysteine (Cys) patterns and associated frameworks. The classification of these Cys patterns, based on conserved framework combinations, enabled the generation of species-level pattern barcodes. These barcodes were then evaluated to assess the species correlations of individual sequences. By analyzing 151 known *Conus* peptide PDB files, we computed Cys disulfide linkages to assess overall stability profiles. Incorporating barcode data allowed us to filter the dataset and prepare it for machine learning (ML) processing. Random Forest (RF) modeling, a supervised learning technique, was used to predict the therapeutic potential of venom peptides. Feature extraction was based on known venom-derived approved peptide-based drugs. The dataset was split into a 70:30 train-test ratio. A total of 6,430 peptides (5,985 from cone snails and 445 from other venomous species) were used to evaluate model prediction capability. The proposed model achieved ideal accuracy (90.48%) in peptide therapeutic classification. Subsequent model outputs underwent further structural and binding pattern analysis against known targets, revealing significant similarities between the binding patterns of approved and novel peptides. The model’s performance could be further enhanced by incorporating additional datasets and optimizing feature selection, potentially broadening its applicability to larger peptide datasets. Overall, this study underscores the potential of ML in advancing pharmacological research on diverse venom peptides.

## Introduction

Multiple toxins evolved among microbes, plants, and animals as defense mechanisms and tools for prey capture [1], with many of these toxins being peptides. Venoms from animals such as wasps, spiders, scorpions, snakes, and cone snails primarily contain bioactive proteins, peptides, and small molecules, with venom peptides being the most dominant component. Venom peptides are particularly potent because they have evolved to target vital receptors in prey, causing effects like heart failure, tissue damage, and paralysis. Recently, they have gained recognition as safer, potent, and selective therapeutic agents [2]. These peptides are categorized on the basis of sequence similarity and common Cys patterns, which establish distinct disulfide associations [3].

Cys, a non-essential amino acid, is essential for metabolism, protein structure formation, and regulating cellular redox balance. Evolutionarily, Cys residues are more common in higher organisms, highlighting their specialized functions [4]. Venom peptides are grouped into Cys-poor and Cys-rich categories based on the number of Cys residues in their sequences. Cys-poor peptides lack extensive disulfide bridges, granting them greater flexibility [5]. In contrast, Cys-rich peptides form disulfide bonds, enhancing structural stability and resistance to proteolytic degradation [6]. These Cys-rich peptides are abundant in venoms from organisms like scorpions, snakes, and cone snails [7]. For example, scorpion venoms can contain up to 50% Cys-rich peptides [8], and black-necked spitting cobra (*Naja nigricollis*) venom is composed of about 70% cytotoxins, which are characterized by Cys-rich residues that form disulfide bonds, contributing to structural stability [9]. Snake venoms also contain Cys-rich peptides, with 9% of secretory proteins in spine-bellied sea snake venom (*Hydrophind curtus*) being Cys-rich [10]. Cone snail venoms, known for their toxic conotoxins, show significant composition variability, with conotoxins comprising 26–71% of total venom transcripts [11]. The high Cys content in these peptides helps form disulfide bonds that stabilize their three-dimensional structure, making them resistant to enzymatic degradation and environmental changes [12].

Cys-rich peptides play essential roles in the predatory and defensive mechanisms of venomous animals by targeting specific physiological pathways in prey [1]. Their structural features make Cys-rich peptides attractive to therapeutic candidates due to their stability and selectivity. Historical evidence of venom peptides in medicine includes a Scythian physician using steppe snake venom to stop a patient’s bleeding [13]. In modern medicine, venom peptides are employed to treat conditions like arthritis, gastrointestinal issues, and cancer [14,15]. Notably, ω-conotoxin MVIIA from *Conus magus* was FDA-approved as an analgesic (Ziconotide, Prialt®) for chronic pain management by acting as a Ca2^+^ channel blocker [16]. As these peptides increasingly demonstrate potential to target specific human receptors, demand grows for large-scale production and therapeutic exploration.

Conopeptides and conotoxins, derived from cone snails, are among the most diverse and abundant bioactive peptides in venoms, yet less than 0.1% of the estimated 1,000,000 potential conopeptides [17] from approximately 700 species have been studied pharmacologically [18]. Cone snails, renowned for their ornamental shells, are predatory carnivores that immobilize prey using complex venoms delivered via harpoon-like teeth [19,20]. Several research groups are working to identify the Cys patterns, frameworks, connectivity, and therapeutic potential of cone snail venom peptides. Cys patterns refer to a specific arrangement of Cys residues within a protein sequence, influencing disulfide bond formation [21], while a Cys framework represents a conserved pattern of Cys positions within a protein domain, defining its overall fold and disulfide connectivity [22]. Cys frameworks form conserved patterns with specific connectivity, where pairing of Cys residues form disulfide bonds [23]. Despite their significance, fewer than 40 conotoxin Cys frameworks have been reported, only 8 of which have known disulfide bond connectivity. A comparative analysis of Cys framework across all available species is yet to be reported, and only a small number of cone snail species have data on Cys patterns and connectivity. Due to lack of available 3D structures for cone snail proteins, venom peptide identification and characterization is a challenging task. Additionally, peptide extraction is dangerous, time-consuming, and costly due to the structural diversity and complex biochemical properties [24]. The absence of comprehensive databases and standardized protocols further complicates the task. An innovative approach is needed to accurately predict the therapeutic potential of venom peptides before extraction, addressing challenges related to safety, cost, and efficiency in drug discovery.

Machine learning (ML) has great potential for identifying the therapeutic potential of venom peptides by considering essential features linked to therapeutic behavior, integrating sequence data, structural information, and physicochemical properties [25,26]. For instance, Bedraouri’s group created an ML framework that successfully predicted analgesic peptides from scorpion venom [27]. Similarly, Wang et al. used a support vector machine (SVM) model to identify antimicrobial peptides from snake venom, effectively identifying peptides with strong antibacterial activity [28]. These cases underscore the value of ML in venom peptide research, accelerating the discovery and development of novel therapeutics. Although several ML-based models have been created for venom peptide applications, none has yet been designed to predict the therapeutic potential of venom peptides specifically, highlighting a significant research gap.

The primary objective of this study is to identify novel Cys patterns and frameworks, predict connectivity information, group *Conus* species based on common connectivities, and develop an ML model using diverse feature representation techniques to assess the therapeutic potential of venom peptides. This study aims to determine whether a model with low-dimensional features can achieve high accuracy, which would improve usability, explainability, and reliability. By harnessing ML, researchers can overcome traditional challenges and unlock the therapeutic potential of venom peptides, opening new avenues for treatments across various medical fields.

## Methodology

### Data collection, Cys framework and pattern analysis

**Fig 1:**
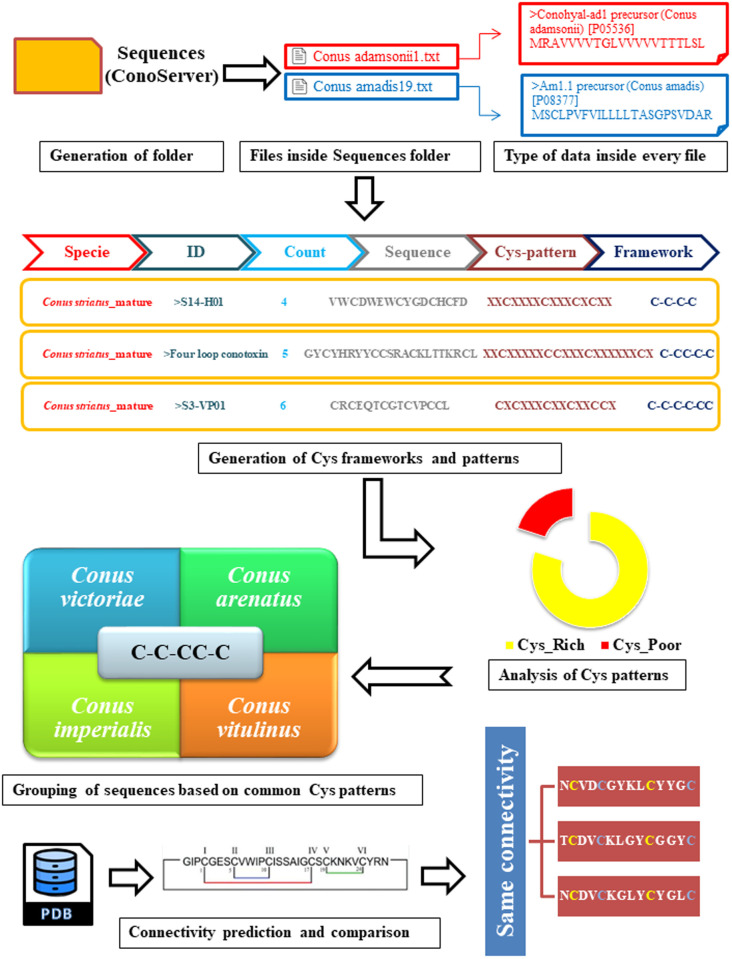
Flowchart scheme of Cys pattern identification, framework prediction and connectivity analysis. The arrow directions indicate the flow of work. Cone snail peptides isolated through ConoServer database were subjected to Cys pattern and framework evaluation. The sequences were categorized based on shared patterns and frameworks, and analyzed for connectivity differences among cone snail species.

The methodology in this study involved collecting mature toxin and protein precursor sequences from ConoServer, a dedicated database for the sequences and structures of conopeptides [29]. A total of 5,985 sequences from 119 *Conus* species were gathered. Each sequence was analyzed for amino acid variations at species level using a Python script, and they were organized into individual files. For example, sequences for *Conus geographus* were saved in a file named *Conus_geographus58.txt* within the sequence folder, containing 58 sequences for this species. The methodology for Cys framework prediction and pattern analysis is illustrated in Fig 1. Multiple analyses were conducted on the dataset to identify Cys frameworks and their patterning. These steps included determining the number of Cys residues and performing count-based categorization. Cys connectivities were predicted using an in-house cone snail PDB library, and sequences with similar Cys frameworks along with their patterns and connectivities were grouped into a separate file (*pattern.csv*) through comparative analysis.

### Cys pattern identification

After data collection, each sequence underwent analysis to count Cys residues and tag their positions. To simplify this process, intervening residues were replaced by “X.” Python modules Pandas [30] and RegEx [31] facilitated this task: Pandas enabled the data to be stored and managed in a two-dimensional, tabular structure [32], while RegEx matched text based on preset patterns, dividing each pattern into one or more sub-patterns to detect specific sequences. The input was the sequences folder, and the output was a CSV file with detailed sequence information.

### Cys pattern classification

To identify unique Cys patterns, sequences were classified as Cys-rich or Cys-poor, and sequences without any Cys residues were excluded. All potential Cys patterns were computed, revealing common patterns among cone snail species. Previously reported patterns from the literature [33] were also compared to annotate any biologically relevant peptides. A Python script containing these known patterns was applied to each sequence, adding the pattern as a dictionary key and identifying both matched and novel patterns. Unique and matching patterns were stored in separate files, which were then compared to identify known and novel Cys patterns. The final patterns were grouped in CSV files based on identical Cys patterns, and redundancy was minimized by removing duplicate patterns within species. This comparative analysis highlighted the diversity and uniqueness of Cys patterns across species.

### Disulfide connectivity prediction

Disulfide connectivity was predicted using a PDB library of cone snail peptides, compiling all data available in the PDB database (http://www.rcsb.org). The library included 3D structures of novel peptides with more than 70% sequence similarity to known peptides, totaling 151 PDB files. An in-house Python script was used to predict connectivities, resulting in CSV files with comprehensive connectivity information. Connectivities were then compared across species, and additional analysis contrasted cone snail Cys connectivities with those of FDA-approved or clinical trials peptides.

### ML model for therapeutic potential of conopeptides

The positive dataset consisted of known 35 venom-derived peptide drugs, either FDA-approved from DrugBank [34] or currently in clinical trials, with recognized therapeutic potential (Table 1). The negative dataset included 5405 sequences from ConoServer and UniProt, comprising venomous peptide sequences from cone snails, snakes, and other species (5405, 390, and 155 sequences, respectively).

**Table 1:** Overview of 35 venom-derived peptide drugs, including their sequences, pharmacological activities, and regulatory status (FDA-approved or in clinical trials).

Drug	Sequence	Pharmacological activity	Status	Reference
Cobrotoxin	LECHNQQSSQTPTTTGCSGGETNCYKKRWRDHRGYRTERGCGCPSVKNGIEINCCTTDRCNN	Inhibits acetylcholine receptors, impairs ion channels, acts as a neurotoxin.	Approved	https://www.uniprot.org/uniprot/P60770
Melittin	GIGAVLKVLTTGLPALISWIKRKRQQ	Targets the lymphatic systems.	Approved	https://pubchem.ncbi.nlm.nih.gov/compound/Melittin
Exenatide	HGEGTFTSDLSKQMEEEAVRLFIEWLKNGGPSSGAPPS	Glucagon-like peptide-1 (GLP-1) analogue for type 2 diabetes therapy.	Approved	https://pubchem.ncbi.nlmnih.gov/compound/45588096
Ziconotide	CKGKGAKCSRLMYDCCTGSCRSGKC (synthetic)	Blocks neuronal calcium channels, provides pain relief.	Approved	https://pubchem.ncbi.nlm.nih.gov/compound/16135415
Hainantoxin	MKASMFLALTGLALLFVVCYASESEEKEFSNELLSSVLAVDDNSKGEERECLGFGKGCNPSNDQCCKSSNLVCSRKHRWCKYEIGK	Blocks voltage-gated sodium channels.	Approved	https://www.uniprot.org/uniprot/D2Y2D7
Margatoxin (MgTX)	TIINVKCTSPKQCLPPCKAQFGQSAGAKCMNGKCKCYPH	Selectively inhibits Kv1.3 voltage-depended potassium channels.	Approved	https://pubchem.ncbi.nlm.nih.gov/compound/145808-47-5
Apitoxin	GLGVLLVLTGLPALISTILALAGG	Contains enzymes and polypeptides with immunogenic effects.	Approved	https://pubchem.ncbi.nlm.nih.gov/compound/133082063
Lixisenatide	HGEGTFTSDLSKQMEEEAVRLFIEWLKNGGPSSGAPPSKKKKK	GLP-1 receptor agonist for type 2 diabetes.	Approved	https://pubchem.ncbi.nlm.nih.gov/compound/90472060
Bivalirudin	FPRPGGGGNGDFEEIPEEYL	Thrombin inhibitor used as an anticoagulant.	Approved	https://pubchem.ncbi.nlm.nih.gov/compound/16129704
Eptifibatide	CXGDWPC	Inhibits platelet aggregation, preventing clot formation.	Approved	https://pubchem.ncbi.nlm.nih.gov/compound/448812
Vespid chemotactic peptide T	FLPILGKILGGLL	Induces neutrophil chemotaxis.	Approved	https://www.uniprot.org/uniprot/P17231
Protonectin	ILGTILGLLKGL	Antimicrobial peptide disrupts bacterial membranes.	Approved	https://www.uniprot.org/uniprot/P0C1R1
Arenicin-1	GFCWYVCYRNGVRVCYRRCN	Exhibits broad-spectrum antimicrobial activity.	Approved	https://www.uniprot.org/uniprot/Q5SC60
Aurelin	AACSDRAHGHICESFKSFCKDSGRNGVKLRANCKKTCGLC	Antimicrobial peptide with channel-blocking properties.	Approved	https://pubmed.ncbi.nlm.nih.gov/16890198/
Hepcidin	DTHFPICIFCCGCCHRSKCGMCCKT	Regulates iron metabolism and has antimicrobial activity.	Approved	https://pubchem.ncbi.nlm.nih.gov/compound/91864521
Hyastatin	MRVLLILVSLAALAHAESFLKSKTGYQGVQTLPGFIGGSQPHLGGGIGGGRPFISQPNLGGGIGSTRPFPRPQYGDYGSRNSCNRQCPSTYGGRGICCRRWGSCCPTNYKG	Strong antibacterial antimicrobial peptide.	Approved	https://www.uniprot.org/uniprot/C4NZN9
Refludan	LTYTDCTESGQNLCLCEGSNVCGQGNKCILGSDGEKNQCVTGEGTPKPQSHNDGDFEEIPEEYLQ	Used for stroke, deep vein thrombosis, and pulmonary embolism.	Approved	https://www.rxlist.com/refludan-drug.htm
Desirudin	VVYTDCTESGQNLCLCEGSNVCGQGNKCILGSDGEKNQCVTGEGTPKPQSHNDGDFEEIPEEYLQ	Selective thrombin inhibitor for thrombosis prevention.	Approved in some cases	https://pubchem.ncbi.nlm.nih.gov/compound/16129703
Defibrase	IGGDECDINEHPFLAFMYYSPRYFCGMTLINQEWVLTAAHCNRRFMRIHLGKHAGSVANYDEVVRYPKEKFICPNKKKNVITDKDIMLIRLDRPVKNSEHIAPLSLPSNPPSVGSVCRIMGWGAITTSEDTYPDVPHCANINLFNNTVCREAYNGLPAKTLCAGVLQGGIDTCGGDSGGPLICNGQFQGILSWGSDPCAEPRKPAFYTKVFDYLPWIQSIIA	Reduces fibrinogen levels, acts as an anticoagulant.	Clinically approved	https://www.uniprot.org/uniprotkb/P04971/entry
ω-CVID	CKSKGAKCSKLMYDCCTGSCSGTVGRC	Inhibits specific voltage-sensitive calcium channels.	Under clinical trails	https://pubmed.ncbi.nlm.nih.gov/23713957/
ShK-186	RSCIDTIPKSRCTAFQCKHSMKYRLSFCRKTCGTC	Potent blocker of Kv1.3 channels, affecting T cells.	Under clinical trails	https://www.uniprot.org/uniprotkb/P29187/entry
CGX-1160	ESEEGGSNATKKPYILRASDQVASGP	Analgesic peptide for chronic pain treatment.	Under clinical stage	https://www.uniprot.org/uniprotkb/Q9XYR5/entry
χ-MrIA	NGVCCGYKLCHOC-NH2	Inhibits norepinephrine transporter, providing analgesic effects.	Under clinical trail	https://pmc.ncbi.nlm.nih.gov/articles/PMC6470548/
Contulakin-G	QSEEGGSNATKKPYIL	Agonist of neurotensin receptors.	Under clinical trails	https://go.drugbank.com/drugs/DB05950
BLZ-100 (Tozuleristide)	MCMPCFTTDHQMARRCDDCCGGRGRGXCYGPQCLCR	Binds to cancer cells of neuroectodermal origin.	Under clinical trails	https://pmc.ncbi.nlm.nih.gov/articles/PMC8355837/
SOR-C13	DCSQDCAACSILARPAELNTETCILECEGKLSSNDTEGGLCKEFLHPSKVDLPR	It is TRPV6 calcium channel inhibitor that disrupts the function of TRPV6, a calcium channel overexpressed in solid tumor cancers.	Under clinical trails	https://www.uniprot.org/uniprotkb/P0C2P6/entry
Hi1a	NECIRKWLSCVDRKNDCCEGLECYKRRHSFEVCVPIPGFCLVKWKQCDGRERDCCAGLECWKRSGNKSSVCAPIT	Involves in to preventing heart damage during heart attacks and to protect donor hearts during transplantation.	Under clinical trials	https://www.uniprot.org/uniprotkb/A0A1L1QJU3/entry
Conantokin-G	GEXXLQXNQXLIRXKSN	Neuroprotective effects against excitotoxicity and showing promise in treating pain and other neurological conditions.	Under clinical trails	https://pubchem.ncbi.nlm.nih.gov/compound/Conantokin-G
Conotoxin M I	GRCCHPACGKNYSC	Blocks nicotinic acetylcholine receptors.	Research use only	https://www.uniprot.org/uniprot/P01521
Calciseptine	RICYIHKASLPRATKTCVEN TCYKMFIRQTQREYISERGCGCPTAMWPYQTECCKGDRCNK	Blocks L-type calcium channels, relaxes smooth muscles.	Research use only	https://www.uniprot.org/uniprot/P22947
μ-EPTX-Na1a	LKCHNTQLPFIYKTCPEGKNLCFKATLKKFPLKFPKRGCADNCPKNSALLKYVCCSTDKCN	Selectively inhibits Nav1.8 sodium channels.	Research use only	https://pubmed.ncbi.nlm.nih.gov/30804211/
Chlorotoxin	MCMPCFTTDHQMARKCDDCCGGKGRGKCYG PQCLCR	Binds glioma cells, blocking chloride channels.	Research use only	https://pubchem.ncbi.nlm.nih.gov/compound/86278273
Mastoparan	INLKALAALAKKIL	Functions as an antimicrobial agent.	Research use only	https://pubchem.ncbi.nlm.nih.gov/compound/6324633
SLPTX Family	MAFQVVLLSFALVVVLAVFDPCPSDCKCDVRSNQCRPVNDDVHPNVCINHYCIGVHLAKREQRPELPHGAWDDSSEEKDS EASLA	Targets histamine H1 and H2 receptors.	Research use only	https://www.uniprot.org/uniprot/A0A023VZH1
Centrocin 1b	MMIKVALVLCAIVATSMVCAKNFEEQDALDTLLNMMLSEEVASPDDAVALQGWFKKTFHKVSHAVKSDIHAGQRGCSALGFSPEEARVKILTAFPEMKEEDLTEEGVRAVCAGAHALGR	An Antimicrobial peptide (AMP) plays role in initiating immune system of various organisms.	Research use only	https://www.uniprot.org/uniprotkb/D8WN03/entry

**Fig 2:**
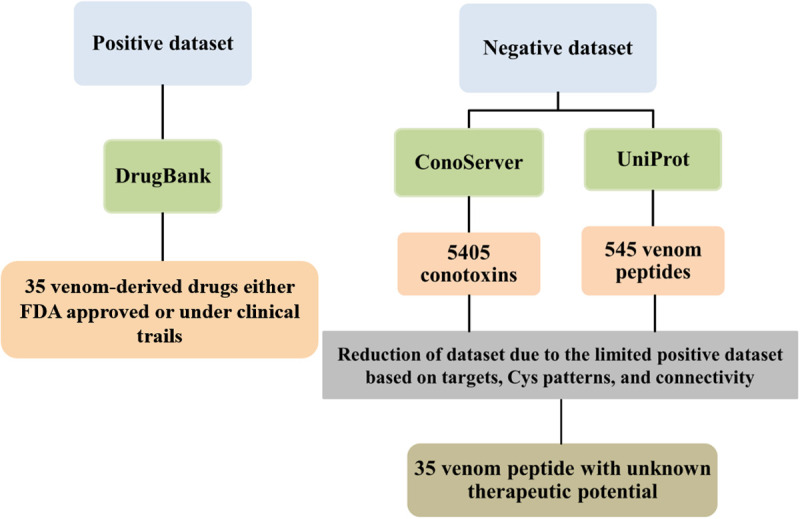
Schematic representation of positive and negative datasets used in this study.

Due to the limited availability of FDA-approved or clinically trials venom-based drugs, 35 unannotated sequences were used as an unregulated or negative dataset, based on the number of Cys residues, frameworks, targets, and connectivity patterns (Fig 2).

**Fig 3:**
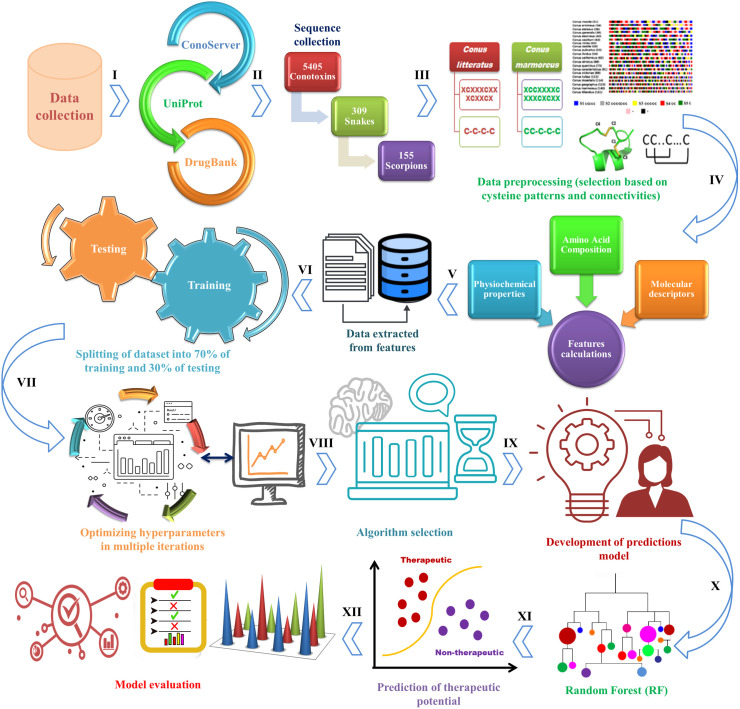
Steps involved in the prediction of venom peptide therapeutic potential. The first step involves the collection of venom peptide data through various databases. Data preprocessing involves cleaning and normalization of data by removing any redundancy followed by preparation of data for analysis. Feature isolation is performed to train the ML model via RF classifier. In model development, generation and optimization of model is carried out to classify individual peptides. Subsequently, predictions are made by applying selected features to identify peptides with high therapeutic potential. Finally, model validation is performed by analyzing and comparing the findings of model-derived peptide sequences with reported venom-derived FDA-approved drugs.

The ML model for predicting the therapeutic potential of cone snail venom peptides was developed through six stepsas illustrated in Fig 3. To create the model, two datasets (positive and negative) were prepared.

### Data preprocessing

Data preprocessing is a critical step in model development, transforming raw data into a clean and valid format to improve model accuracy. This process involves removing noise, deleting duplicates to prevent redundancy, eliminating any invalid characters, correcting errors, and managing outliers present in data [35]. Prior to applying ML models, data standardization is a crucial step, as it significantly impacts model performance. In this study, data preprocessing was performed using the StandardScaler module from the sklearn package [36], ensuring all features were appropriately scaled. Given the dataset imbalance, where *Conus* peptides vastly outnumber those from other venomous species, corrective measures were implemented to mitigate model bias toward the majority class. To address this, Stratified Sampling was applied, ensuring that both the training and test sets maintained proportional class distributions, thereby avoiding misleading evaluation results [37]. Furthermore, oversampling was applied using the RandomOverSampler module from the imbalanced-learn package [38]. This method increased the representation of the minority class, enhancing the model’s ability to learn effectively from all classes.

Hyperparameter tuning was conducted with GridSearchCV from sklearn to optimize model parameters [39]. Additionally, data filtration was based on Cys counts and connectivity patterns to maintain data consistency. Cys numbers, positions, and connectivity patterns for both positive and negative datasets were computed and compared using a Python script.

### Feature isolation

To use an ML model for interpreting biological data, sequences must undergo featurization, converting variable data types into numerical forms for model training. Defining the feature set, or input space, is essential to the accuracy of any ML model [40]. For estimating the therapeutic potential of venom peptides, three feature representation strategies were applied in ML model development:

### Amino acid composition

Amino acid composition (AAC) provides insights into peptide structure by determining the frequency or percentage of each amino acid in a sequence [41]. The descriptor values for the AAC feature set are represented by the following equation:


$$D\left(i\right)=\frac{n}{L}\;\forall \;i\;\in [1,20]$$ (1)

Where *L* is the length of peptide, *D(i)* is the descriptor value particular to amino acid *i* in the peptide, and *n* is the i^th^ amino acid’s frequency of occurrence.

The amino acid composition (AAC) for each peptide sequence was calculated using the ProteinAnalysis module from the ProtParam tool in the Bio.SeqUtils package [42] and used as a feature set for training the ML model.

### Physicochemical property evaluation

Physicochemical properties are crucial for characterizing the therapeutic potential of venom peptides in terms of stability and solubility [43]. Key properties include charge, hydrophobicity, molecular weight, and isoelectric point, all of which significantly influence a peptide’s ability to bind to receptors, enzymes, and ion channels, thereby affecting its therapeutic activity. Amphipathicity is another important factor, as it enables peptides to penetrate cell membranes and target cellular locations effectively [44]. These properties help maintain the peptide’s stability in a biological environment, preventing aggregation and enhancing the predictive accuracy of the ML model for therapeutic peptides. For each peptide in the dataset, various physicochemical properties (listed in Table 2) were calculated using different modules of Bio.SeqUtils, resulting in the creation of a multi-dimensional feature vector.

**Table 2:** Physiochemical properties are used as features in developing a ML model.

Property	Definition
Sequence length	Total number of amino acids found in a peptide sequence.
Sequence charge	The total charge of the sequence of peptides.
Isoelectric point	The point where a molecule has no net charge.
Charge density	Overall charge (at pH = 7)/ molecular weight of peptide sequence.
Instability index	Peptide stability is measured and computed using the instability value of every potential dipeptide in sequence [45].
Aromaticity index	The presence of relative frequency of aromatic amino acid in sequence [46].
Hydropathy index	Using the unique hydropathy index of each constituent amino acid, it measures the hydrophobic and hydrophilic propensity of peptides within a sequence [47].
Hydrophobicity	Measuring the hydrophobicity of peptides present in a sequence [48].

### Additional descriptor sets

Other descriptors included as input parameters for the ML model were hydrogen bond donors, acceptors, and LogP, calculated using the Descriptors function from the rdkit.chem package [49]. These features influence solubility, molecular interactions, and membrane permeability, which are critical for therapeutic efficacy, thereby enhancing the ML model’s ability to accurately predict the therapeutic potential of venom peptides [50]. These characteristics also govern the pharmacokinetic behavior of peptides in vivo and regulate their ability to bind to various biological targets. The pd.concat function from Pandas was used to combine all these features into a single dataset.

### Random Forest classifier

The Random Forest (RF) classifier was employed to predict the therapeutic potential of venom peptides due to its robustness, high accuracy, and stability compared to a single decision tree [51]. RF is an ensemble classifier that integrates a decision tree algorithm, a supervised ML approach used for classification and regression. It minimizes overfitting risks by using limited hyperparameter inputs, balancing bias and variance, making it a valuable tool for prediction, modeling, and data analysis across various domains [52]. RF extends the Classification and Regression Tree (CART) method by applying bagging (bootstrap aggregation) and voting to determine classification results. The RF model consists of ‘N’ decision trees generated from the training dataset, aimed at converting multiple weak classifiers into a robust single classifier. The number of trees in the model is determined by the number of generated bootstrap samples. For each bootstrap sample, a decision tree is constructed following these rules: If there are *M* input variables, the predictor variables considered at each node are randomly chosen as *m*, where *m* is less than *M*. The variable *m* is selected randomly through the total *M* variables. To identify the best predictor variable from the chosen *m*, a measure of purity, such as Gini or entropy, is calculated. The Gini index, denoted as *G*_*gini*_ *(D)*, is used to determine the optimal binary split point for each feature. G_gini_ reflects the uncertainty of the set *D*. In classification tasks with *N* classes, the G_gini_ index for a given sample set *D* is calculated with the following equation:


Ggini (D)=1−∑n=1N(|Cn|−D)2 (2)

In eq (2), *C*_*n*_ represents the subset of samples in *D* that belong to the nth class [53]. When subsets are defined as follows:


$$D_{1}=\left\{(x,y)\in D|A(x)=a\right\},D_{2}=D-D_{1}$$ (3)

The optimal split for the predictor variable *m* is utilized to split the nodes. The value of *m* remains fixed during the growth of the forest. Each tree is constructed to its full extent without pruning. The final output of the RF model is determined by aggregating the results of all classification trees through majority voting, selecting the most accurate prediction [53]. The most informative predictor variable provides the greatest contribution to the decision-making process. The generation of additional trees and their collective use in the decision-making process ensures a more reliable and robust outcome [54].

In this study, we utilized the sklearn package to classify peptides into two venomous sequence classes. The RFClassifier function was employed to build the model with optimal hyperparameters, and cross_val_score was used for cross-validation. The model was constructed using various threshold values, with the optimal threshold (yielding high accuracy and precision) chosen for the final model. The dataset was divided into training (70%) and testing/validation (30%) subsets. Optimizing the RF algorithm is essential for extracting high-quality features and selecting parameters, significantly reducing the model’s generalization error and improving classification accuracy. Based on ideal hyperparameters, we set 100 optimal estimators (trees). Predictions were made using the rf.predict function and evaluated through various performance metrics.

### Support Vector Machine (SVM) classifier

The Support Vector Machine (SVM) classifier, a robust ML model for classification and regression, was chosen for its ability to handle high-dimensional data while minimizing overfitting. SVM identifies an optimal hyperplane that maximizes the margin between classes by utilizing support vectors, which are the data points closest to the hyperplane and define the decision boundaries [55]. Its theoretical foundation is rooted in optimization principles and statistical learning theory [56]. SVM is particularly effective when training data is limited, and traditional statistical methods struggle to guarantee an optimal solution based on large datasets [57].

SVM can be categorized into linear and nonlinear types. Linear SVM utilizes a linear kernel function to separate data with a straight boundary, offering computational efficiency for applications such as drug design and document classification [58]. On the other hand, nonlinear SVM employs kernel functions to map input features into a higher-dimensional space, enabling better classification of complex datasets [59]. Various kernel functions, including radial basis function and polynomial, were selected according to task-specific requirements.

The SVM classifier was implemented using the sklearn package, while feature calculations were performed using the Bio.SeqUtils package. To address class imbalance and mitigate overfitting, the SMOTE (Synthetic Minority Oversampling Technique) algorithm was applied to increase the representation of the minority class. The SVC function was used to build the model with the best hyperparameters, which were identified using GridSearchCV and retrieved via *grid_search.best_params*. The model was trained and evaluated across different threshold values, selecting the optimal threshold where both accuracy and precision were maximized. Finally, predictions were made using *best_svm.predict*, and various performance metrics were employed to assess the model’s accuracy.

### Performance metrics

The performance evaluation metrics included two types of values: predicted and actual values.

The following metrics were used to assess model performance.

### Confusion matrix

A confusion matrix was generated based on the test dataset outcomes. This table provides insights into the model’s accuracy and is also referred to as an error matrix or contingency matrix. It can be utilized for both binary and multi-class classification problems [60]. The confusion matrix comprises four components:

#### True Negatives (TN).

The number of non-therapeutic peptides correctly classified as non-therapeutic.

#### True Positives (TP).

The number of therapeutic peptides correctly classified as therapeutic.

#### False Positives (FP).

The number of non-therapeutic peptides incorrectly classified as therapeutic.

#### False Negatives (FN).

The number of therapeutic peptides incorrectly classified as non-therapeutic.

### Classification report

A classification report was generated to evaluate the quality of the classification model, calculating recall, precision, F1 score, and support scores. The following equations (4–7) were used to compute these metrics:


$$\begin{array}{c} Precision=\frac{TP}{TP+FP} \end{array}$$ (4)

$$\begin{array}{c} Recall=\frac{TP}{TP+FN} \end{array}$$ (5)

$$\begin{array}{c} F1\;score=\;2*precision*\frac{recall}{\;precision+recall} \end{array}$$ (6)

$$\begin{array}{c} Accuracy=\;TP+\frac{TN}{TP+FP+TN+FN} \end{array}$$ (7)

### Receiver Operating Characteristic (ROC)

The Area Under the Receiver Operating Characteristic Curve (AUC-ROC) [61] serves as a performance metric for binary classification problems. It is plotted to estimate the True Positive (TP) rate (sensitivity) against the False Positive (FP) rate (1 – specificity) across various threshold settings. The AUC-ROC value ranges from 0 to 1, with higher values indicating better model performance. The ROC curve is used to evaluate how effectively the model distinguishes between positive and negative classes. Ideally, the curve should reside in the upper left corner of the plot, which signifies a high True Positive Rate (TPR) – correctly identifying most positive cases – coupled with a low False Positive Rate (FPR) – indicating few false positives [62].

### SHapley Additive exPlanations (SHAP)

SHAP is employed to assess the contribution of each feature to the model’s predictions, identifying the most influential features and their impact on the Random Forest (RF) model’s results. Each feature is assigned a relevance value that reflects its contribution to the output [63], based on principles of game theory. Positive SHAP values indicate features that positively influence predictions, while negative SHAP values denote features that have a detrimental effect; however, in some contexts, negative effects can also be significant. While SHAP analysis enhances the explainability of machine learning models, it has inherent limitations [64]. In SHAP analysis, each feature is considered independent of all others, and it solely clarifies a feature’s contribution to the model’s prediction without implying causation. Therefore, the results from SHAP should be interpreted with caution.

### Molecular docking analysis

3D structures of *Conus striatus* peptide (PDB ID: 1FYG) and *Conus magus* Ziconotide-bound human N-type voltage-gated calcium channel (Ca_v_2.2) (PDB ID: 7VFU) were isolated through RCSB PDB (*www.rcsb.org*). Interaction of Ca_v_2.2 and *Conus striatus* peptide (referred as peptide-cs) had been evaluated through molecular docking. PatchDock server [65] and embedded refinement tool FireDock [66] was utilized to describe their interaction patterns. PatchDock accomplishes docking analysis through a segmentation algorithm based on the structure geometry. It recapitulates docking transformations that yield good complementary molecular shapes based on a small number of steric clashes and wide interface areas. The PatchDock algorithm classifies the Connolly dot surface representation of the protein molecules as concave, convex, and flat patches [67]. The complementary patches are matched to generate the candidate transformations. A scoring function also evaluates each candidate transformation, which considers both the atomic desolvation energy and geometric fit [68] to measure each candidate transformation. Finally, the most suitable candidate solution is selected among the redundant solutions based on RMSD (Root Mean Square Deviation) clustering. Overall, three major steps are followed in the PatchDock analysis: (i) surface patch matching, (ii) molecular shape representation, and (iii) filtering and scoring [69]. PDBSum [70] and UCSF Chimera version 1.18 were employed to analyze the interactions.

## Results

### Categorization of sequences based on Cys count

**Fig 4:**
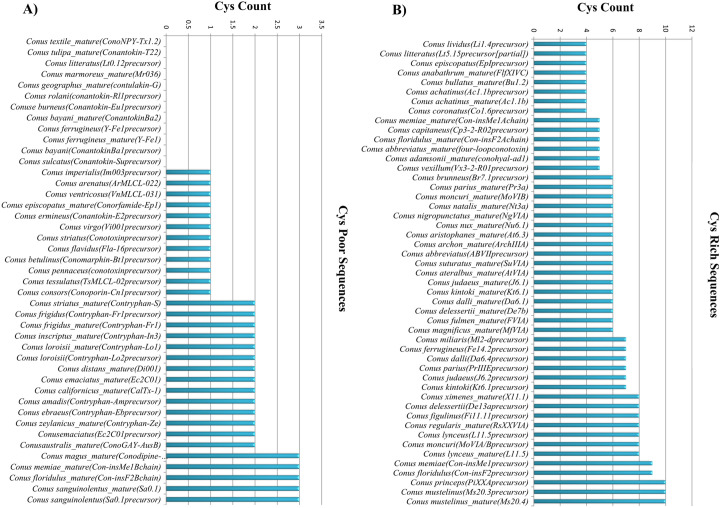
Plot illustrating sequence categorization based on Cys count. Data along X-axis represents species names along with their unique IDs according to ConoServer database. Cys count is present at the Y-axis. Species having the word “mature” next to its name indicates that its subtype’s sequence is mature; species without this word displays a protein precursor. **(A)** Species have low Cys count. **(B)** Cys rich species. Certain species sequences are present multiple times with different Cys counts.

Numerous sequences in our dataset have been classified as rich or poor based on the Cys number. There is no fixed threshold below which a sequence is considered as Cys-poor; instead, sequences with low Cys counts (<= 3) are categorized as poor, while >3 counts are designated as Cys-rich. In our dataset, we isolated 5421 and 563 Cys-rich and Cys-poor sequences, respectively. Fig 4 represents a portion of cone snail sequence classification based on the Cys count. A list of all cone snail sequences having low and high Cys counts is presented in the supplementary data (cys_poor.csv and cys_rich.csv). Evidently, sequences of common species exist in both categories indicating that depending on the amount of Cys count, multiple sequences of similar species may be categorized as either rich or poor. For instance*,* Lt0.12precursor and Lt5.15precursor [partial] of *Conus litteratus* exhibit varying Cys content. Although produced by the same species, the sequences differ due to variations in their amino acid composition (with one being partial), leading to differences in peptide structures and functions.

### Prediction of novel Cys patterns and frameworks

**Fig 5:**
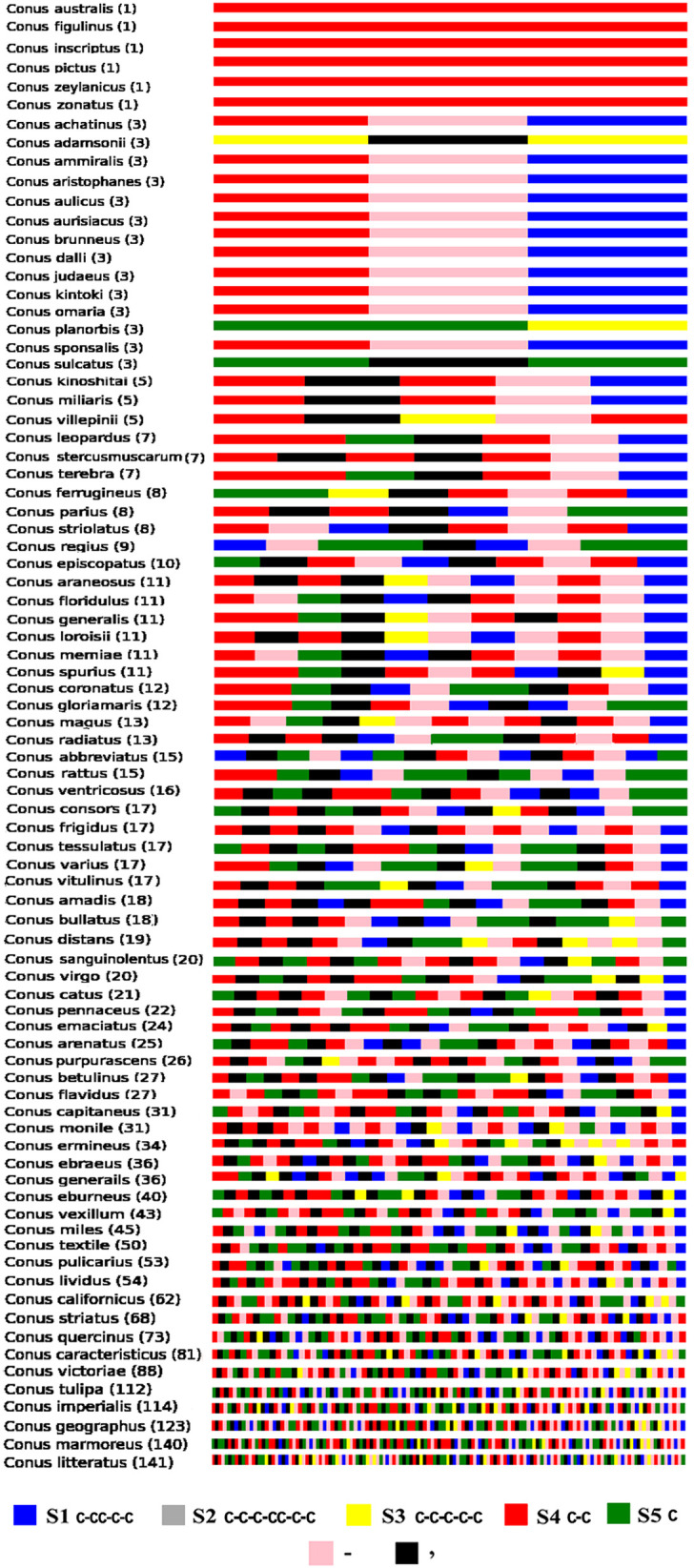
Barcode generation for Cone snail Cys patterns. For specific species, each single-colored strip represents a unique pattern. Each scaffold represents a unique set of patterns, as we have a large number of residues. We aim to visualize and analyze these patterns by representing them in the form of strips for clearer interpretation. For example, C-CC-C-C pattern is indicated by scaffold S1 in blue color. C-C-C-CC-C-C pattern is represented by S2 in the gray color. Similarly, C-C-C-C-C and C-C patterns are designated by S3 and S4 in yellow and red colors, respectively, while S5 scaffold in green indicates Cys. The intervening region between two scaffolds is indicated by pink color. The separation of two patterns belonging to same specie is indicated by black color comma (,). The number of unique patterns is labeled in small parenthesis along with the names of species.

In order to differentiate among distinct Cys patterns and frameworks (known and novel), sequence data of 119 cone snail species gathered via ConoServer were compared to the reported ones. Each pattern and framework is labeled as known upon its matching to a previously reported sequence pattern and framework. A significant portion of patterns and frameworks were considered as novel based on this comparison. These patterns and frameworks underwent preprocessing, which involved organizing them based on species name, identifying the corresponding scaffolds (specific Cys frameworks are represented by unique identifiers), and generating a barcode for each pattern and framework to facilitate their classification and comparison. Through this strategy, we extracted 82 cone snail species having novel Cys patterns and frameworks. Due to huge data volume, scaffold-based characterization revealed multiple instances of common Cys patterns and frameworks (Fig 5). The sequence details of these barcodes are listed in S1 Table.

**Fig 6:**
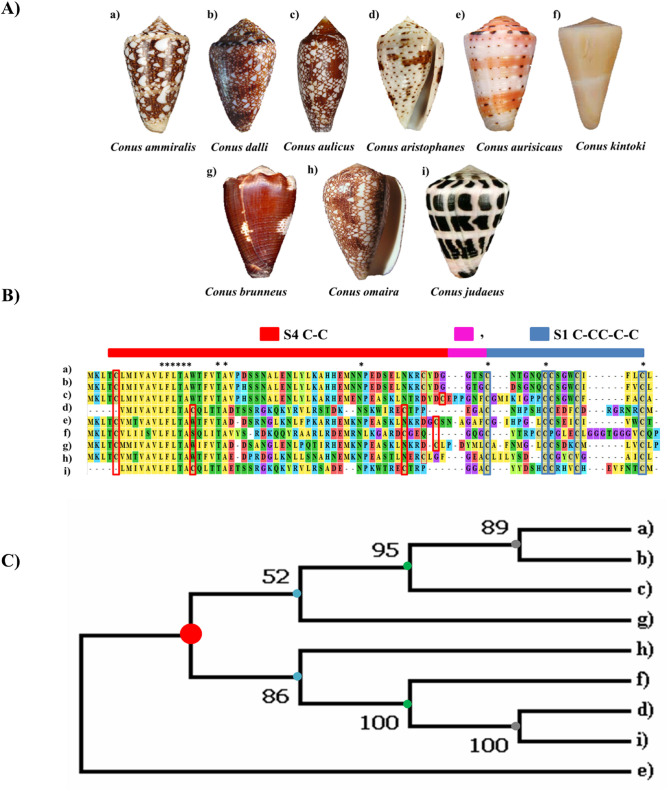
Comparative alignment and phylogenetic analysis of a unique Cys pattern in cone snail species. **(A)** Depiction of cone snail species (a to i) with a similar novel pattern. **(B)** Comparative alignment of pattern sequences of mentioned cone snail species. * denotes conserved alignment. The common residues are indicated in distinct colors. Barcode is indicated through the combination of S4 and S1 scaffold. **(C)** Phylogenetic analysis indicates that these species share a similar ancestral root highlighted by a red circle.

Multiple cone snail species exhibited distinct patterns having similar Cys count and arrangement despite having different sequences, indicating the presence of close association and selection among several species. This relationship can be explained through similarities in ancestry, evolution, geographic proximity, and other elements. We selected 9 cone snail species sharing a common Cys pattern (C-C-C-CC-C-C) and performed sequence alignment and phylogenetic analysis, which suggested that these species are related or share a common ancestor (Fig 6). Evidently, *Conus ammiralis* and *Conus dalli* shells exhibit similar coloring patterns, which may be due to a common genetic background for pigmentation. As both species are localized in similar ecological niches and are subjected to equivalent selective pressure, their shell designs have been evolved in a convergent manner. Likewise, it has previously been reported that *Conus aulicus* and *Conus omaria* share color pattern similarities in their shells as a result of divergence from the same ancestral root [71]. In terms of venom composition, *Conus achatinus*, *Conus aristophanes, Conus aulicus,* and *Conus aurisacus* venoms contain various conopeptides but all of them belong to α-conotoxin class. α-conotoxins are fascinating because they specifically antagonize Nicotinic acetylcholine receptors (nAChRs). These peptides exhibit exquisite subtype selectivity and have been essential in characterizing native nAChR isoforms involved in neurotransmitter release modulation, Parkinson disease pathophysiology, and nociception [72].

### Cys connectivity analysis

**Fig 7:**
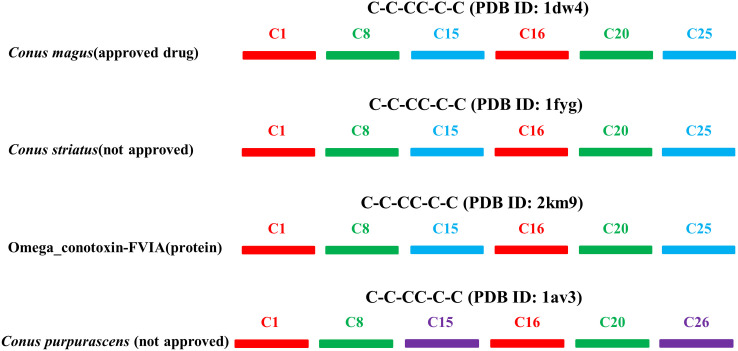
Comparative overview of an FDA-approved drug Ziconotide (PDB ID: 1dw4) and cone snail peptides having similar connectivity pairs. C1 exhibits connectivity with C16 (red), C8 shows connectivity with C20 (green), and C15 shows connectivity with C25 (blue) and C26 (purple) depending on species.

An in-house PDB library of cone snail peptides was utilized to confer the Cys connecting pairs. The connectivities of all available PDB files were computed and compared. Cone snail species having similar Cys count, patterns and connectivity pairs as of approved venom-derived drug were mentioned in S1 Fig. For instance, C-C-CC-C-C pattern of *Conus magus* extracted from sequence was exactly similar to that of *Conus magus* in terms of connectivity (Fig 7). We computed connectivity data for individual cone snail species through 151 currently available PDB files of cone snail peptides and presented in S2 Table.

Next, we gathered the PDB IDs of available cone snail venom-derived FDA approved drugs for connectivity analysis and compared with our data. The purpose of this process was to isolate novel peptides on the basis of common Cys patterns and their connectivities. For example, Ziconotide, an FDA-approved peptide isolated from *Conus magus* with a PDB ID: 1dw4, which has been reported for the management of severe chronic pain in patients refractory to other treatments and for whom intrathecal therapy is warranted [16], shared the same connecting pairs with *Conus striatus* and omega conotoxins_FVI-A (Fig 7). Given that *Conus striatus* peptide exhibits a high conservation and similar connectivity to ziconotide, our study proposes that it may likewise be utilized to treat pain. Similarly, *Conus purpurascens* has a comparable similarity in connectivity pattern with ziconotide, except the presence of a single connecting pair, which suggests that *Conus purpurascens* is a viable option that may result in a positive outcome. There are multiple such cases in our analysis, which are mentioned in S3 Table.

### Model performance evaluation

To determine the therapeutic potential of venom peptide sequences, the effectiveness of RF model was assessed through ten distinct threshold values (Table 3). The accuracy, precision, recall, TP, TN, FP, and FN values were increased following the threshold. The model performed efficiently and accurately at the threshold value of 0.5. As the threshold increased further, performance began to decline, indicating an uneven data distribution.

**Table 3:** RF performance evaluation at different threshold values. A row with a bold text indicates the optimal threshold at which the model performed ideally.

Threshold	Accuracy	Precision	Recall	F1-Score	TP	FP	TN	FN
0.1	0.476	0.476	1	0.645	10	2	9	0
0.2	0.524	0.5	1	0.667	10	2	9	0
0.3	0.571	0.526	1	0.689	10	2	9	0
0.4	0.762	0.667	1	0.8	10	2	9	0
**0.5**	**0.905**	**0.833**	**1**	**0.909**	**10**	**2**	**9**	**0**
0.6	0.810	0.875	0.7	0.778	10	2	9	0
0.7	0.762	1	0.5	0.667	10	2	9	0
0.8	0.714	1	0.4	0.571	10	2	9	0
0.9	0.571	1	0.1	0.182	10	2	9	0

**Fig 8:**
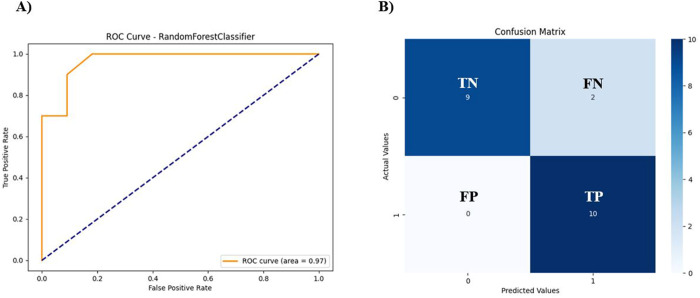
ROC and confusion matrix analysis to evaluate the model performance. **(A)** ROC curve indicates that with the increase of data, model performance is improved. Rapid fluctuations in the TP and FP of ROC curve have been observed because even slight modifications in the predictions may lead to significant variations in these metrics. It is due to small sample sizes for positive and negative classes. AUC is directly proportional to the model performance. Given that the AUC is 0.97, the model performance is significant, and it favors the randomly selected positive instances more. **(B)** Confusion matrix reveals a balance between the positive and negative datasets, resulting in a high similarity between TP and TN. Dark blue color indicates TP and TN, while light color shows FP and FN. Only 2 FN instances are predicted by model, indicating that although the sequences belong to a positive class, the model predicts them in the negative class. There is no sequence predicted as FP. 9 sequences are predicted as TN and 10 are identified as TP. Overall, the model generates more FNs than FPs.

Through ROC, we may assess how well the model discriminates positive and negative instances. ROC curve suggested an AUC of 0.97 for RF model (Fig 8A), indicating its reliability. Similarly, test data set confusion matrix (Fig 8B) revealed higher number of TP and TN, further strengthening the model accuracy.

A classification report summarizes the key performance metrics for each class in an ML model. It includes the weighted average of metrics across all classes, as well as precision, recall, F1-score, and support value for each class [73]. This report offers a comprehensive analysis of the model performance per class, highlighting the balance between recall and precision. Additionally, it shows the number of instances (support) for each class, indicating possible dataset or class imbalances. The classification report of our RF model was generated and listed in Table 4.

**Table 4:** Classification report representing various performance metrics of RF model.

Class	Precision	Recall	F1-Score	Support
0	1	0.818182	0.9	11
1	0.833333	1	0.909091	10
**Accuracy**	0.904762	0.904762	0.904762	0.904762
**Macro average**	0.916667	0.909091	0.904545	21
**Weighted average**	0.920635	0.904762	0.904329	21

Precision, defined as the proportion of TP predictions among all predictions, serves as a critical performance metric. As shown in Table 3, a high precision value of 0.8333 indicates that FN predictions, such as class_1 sequences predicted as 0, were rare. For class_0, the precision value was 1, signifying no FP predictions in this class. The lowest precision value was 0.8333 for class_1, while the highest was 1 for class_0. Notably, class_1 exhibited the highest recall value, indicating very few or no FP predictions. The lowest recall value for class_0 was 0.8182, with the highest for class_1 at 1. This interplay between precision and recall suggests that when one metric is low, the other tends to be high, thereby balancing overall predictions.

The harmonic mean of precision and recall, known as the F1-Score, was 0.9 in most cases, reflecting a balanced evaluation of model performance. Support values, representing the number of actual occurrences for each class in the dataset, confirmed that there was no imbalance. The accuracy of the RF predicted model was 0.9047, with a macro average of 0.9166 and a weighted average of 0.9206, indicating an overall ideal model quality.

### SHAP analysis

**Fig 9:**
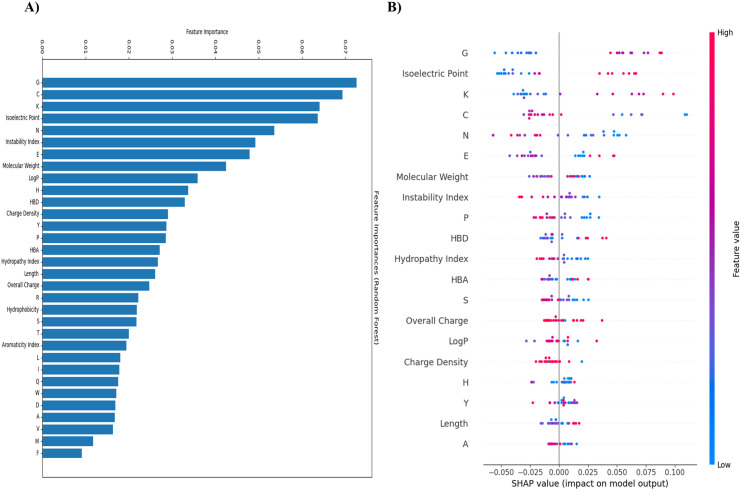
Feature importance plot and summary plot representing SHAP values for RF model features. **(A)** Features are presented on the X-axis and their importance has been mentioned on the Y-axis. Among all features Glycine, Cysteine, Lysine, and isoelectric point exhibit more impact on the model performance. In contrast, Valine, Methionine, and Phenylalanine are the least important. **(B)** The SHAP values are depicted at the X-axis. The feature names are shown at the Y-axis in ascending order with respect to importance. Every point in the plot has a color that corresponds to its related feature value; blue points exhibit low values and red points show high values. A data row (original dataset) is represented by each point. Glycine, isoelectric point, Lysine, and Cysteine are the most important features as described in the feature importance plot. These features affect the model in both positive and negative ways; however, the negative way (blue color) suggests that their affect is negligible. Naturally, for the functioning of certain features, they must have high negative values. For example, higher stability requires a lower instability index [74]. Higher values; however, point to a possible instability. Although the instability index value for our predicted model is negative, indicating a negative impact on the model, in reality, instability index negative value is beneficial for the stability of peptide sequence. More stability is indicated by lower values, and possible instability is indicated by higher values. Overall, this result suggested that the model has the potential of a reliable performance.

In this study, multiple features were employed to train the RF model, with their importance contingent upon the specific task. The contribution of each feature to the model’s predictions, quantified by SHAP values, helps identify the most crucial features and their influence on overall results. Ideally, features with positive SHAP values significantly enhance model performance; however, this depends on the nature of the task. Positive values do not always take precedence over negative ones. If numerous features negatively impact the model but are of lesser importance, their overall effect will be less significant compared to more influential positive values. Feature importance and summary plots of the SHAP values are illustrated in Fig 9.

### Comparative analysis between RF and SVM

**Fig 10:**
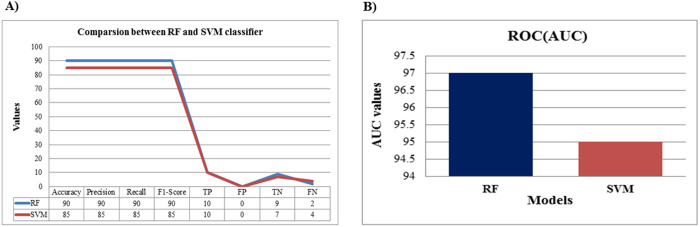
Comparative model performance analysis. **(A)** Detailed comparison of performance metrics between the RF and SVM models, with performance metrics plotted on the X-axis and their corresponding values on the Y-axis. The RF model (blue) outperformed the SVM model (red) across multiple metrics, including accuracy, precision, recall, F1-score, TN, and FN, highlighting its superior reliability in predicting the therapeutic potential of venom peptides. **(B)** AUC comparison bar plot between the RF and SVM models, with models represented on the X-axis and their corresponding AUC values on the Y-axis. The RF model (blue) demonstrates better performance with a higher AUC value compared to the SVM model (red).

To evaluate the effectiveness of both RF and SVM models, a comprehensive comparative analysis of their performance metrics was performed. Various evaluation metrics, including accuracy, precision, recall, F1-score, TP, TN, FP, and FN, were employed to thoroughly assess their predictive capabilities. A comparative plot was generated to visualize the differences in performance between the two models (Fig 10A).

The ensemble approach of RF, which combines multiple decision trees, enhances its generalization ability by reducing variance and minimizing overfitting. Additionally, RF effectively selects relevant features by randomly choosing subsets at each node [75], improving classification performance by considering multiple peptide characteristics such as shape, length, width, area, and perimeter [76]. Moreover, RF is commonly used for describing non-linear spatial data [77], making it a strong alternative to traditional linear models. Both models were further assessed using the AUC of the ROC curve. The RF model achieved a higher AUC value of 0.97, outperforming the SVM model, which attained an AUC of 0.95 (Fig 10B).

As an ensemble learning method, RF combines multiple decision trees to improve accuracy and generalization, reducing noise and maintaining high performance even with limited data [78]. Unlike SVM, which requires manual feature selection and is prone to overfitting, RF automatically selects and ranks important features, thus enhancing model efficiency [79]. For instance, in audio classification, RF achieved 90% accuracy, while SVM only reached 60% [80]. Additionally, RF has shown superior performance in regional land cover mapping, providing higher accuracy, kappa value, and individual accuracy. It efficiently handles large, coarse-resolution datasets and reduces confusion in classifying mixed classes-tasks where SVM struggles due to memory and computational limitations [81].

While models like tree-based gradient boosting methods, such as XGBoost and Gradient Boosting Machines (GBM), may offer higher accuracy in certain contexts, RF remains more stable and interpretable, making it ideal for understanding the complex interactions in venom peptides [82]. Moreover, RF provides clear feature importance, which helps in understanding the contribution of different peptide features to therapeutic efficacy [83]. These qualities make RF a well-suited model for predicting the therapeutic potential of venom peptides.

### Peptide therapeutic potential prediction using RF model

Based on the significant features identified during model training, the RF model was employed to predict the therapeutic potential of specific peptide sequences. The predictions utilized features from the training dataset, including amino acid composition, sequence length, isoelectric properties, physicochemical properties, and molecular weight. Sequences deemed to have therapeutic potential were assigned a value of 1, while those lacking therapeutic significance were given a value of 0. The input dataset, comprising 5,383 sequences with known and unique Cys patterns and frameworks, yielded reliable model predictions (S4 Table). A portion of the model predictions based on the negative dataset is listed in Table 5.

**Table 5:** RF model predictions based on a negative dataset.

Specie name	Sequence	RF Prediction
*Conus magus*	CKAAGKPCSRIAYNCCTGSCRSGKC	1
*Conus lividus (mature)*	TDSEECCLDSRCAGQHQDLCS	1
*Conus litteratus*	GCCARAACAGIHQELCGG	1
*Conus episcopatus (mature)*	GCCSDPRCNMNNPDYCR	1
*Conus emaciatus (mature)*	YAAVVNRASALMAHAALRDCCSDPPCAHNNPDC	1
*Conus eburneus (mature)*	LCPPMCRSCSNC	1
*Conus flavidus (mature)*	DPCCSNPSCAQTHPEIC	1
*Conus eburneus (mature)*	VPAEPILEEICPDMCNSGEGEIFCTCGSRQFVVTLPVIE	0
*Conus ferrugineus (mature)*	SPGSTICKMACRTGNGHKYPFCNCRR	1
*Conus diadema (mature)*	GCCGNPSCSIHIPYVCN	0
*Conus kinoshitai (mature)*	PGCCNNPACGKNRC	1
*Conus generalis (mature)*	TCRSSGRYCRSPYDRRRRYCRRITDACV	1
*Conus generalis*	KDAADLSALNDNNNCCNHPACAGKNSDLC	1
*Conus geographus (mature)*	KFLSGGFKEIVCHRYCAKGIAKEFCNCPDL	0
*Conus geographus*	ECCHPACGKHFSCH	1
*Conus bullatus (mature)*	DECSAPGAFCLIRPGLCCSEFCFFACF	0
*Conus brunneus (mature)*	DCLPDYMLCAFNMGLCCSDKCMLVCLP	0
*Conus betulinus (mature)*	ACAEFGHSCISATCCPGVTCVEIDEPVCLWD	0
*Conus quercinus (mature)*	DCTPCGPNLCCEPGKTCGTSTHHDHYGEPACV	1
*Conus miles (mature)*	CLDDGDDCEIGDDCCSGSCIFDEGDSFCEISYENYGGVS	0
*Conus omaria (mature)*	CVPHEGPCNWLTQNCCSGYNCIIFFCL	0
*Conus quercinus (mature)*	FPCNSNQCACLPAEGSSTSYQCQSLDASTDDCFDNECVTQSEW	1
*Conus brunneus*	SCGGSCFGGCWPGCSCYARTCFRD	1
*Conus quercinus (mature)*	ICRLEADVGPCSGTFPRWFYNSDMSKCQLFDYGGCRGNENRFDTEEECMELC	1
*Conus quercinus (mature)*	LCRLPAVPGPCRARQPRYFYNYKVGKCQRFNYGGCKGNTNRFLTLGECQTRC	1
*Conus quercinus (mature)*	CNLPKIVGPCKAYMPSFFYNTGTGQCERFVYGGCGGNANRFETKQECEGQCQR	1
*Conus imperialis (mature)*	SSTYDDEIATFCWSYWIGFQYSYPYTYVQPCTALGKACTANSDCCSKYCNTKICKINWE	1
*Conus imperialis (mature)*	KTSSTYDDEMATFCWSYWNEFQYSYPYTYVQPCLTLGKACTTNSDCCSKYCNTKMCKINWE	1
*Conus litteratus (mature)*	MKLTCVLIIAVLFLMDNQLITADYSRDEQVYRAVRLRDAMQKSKGSGSCAYISEPCDILPCCPGLKCNEDFVPICL	0
*Conus delessertii (mature)*	DCPTSCPTTCANGWECCKGYPCVNKACSGCTHA	1
*Conus delessertii (mature)*	DCPTSCPTTCANGWECCKGYPCVRQHCSGCNHR	1
*Conus litteratus (mature)*	CLEPLDLGDSTCSEVRIRWYYNTGNEICQTFQYTGCGGNNNNFYDENSCKQCCEWEYSCSKRFA	1
*Conus miles (mature)*	PECYNCFPNDDGHCVGTCCGEDSCKGGIRGCGCV	1
*Conus bayani (mature)*	KCPDNCPSTCPERDECCDGDSCLYNSYMRKYYCYDCGSGGPN	1
*Conus pulicarius (mature)*	CLLSLETGSTSCTDVRIRWYYNQGNEACQPFQYTGCGGNDNNFYNQNDCEHCCKMDLQCNSAS	1

### Structural analysis

**Fig 11:**
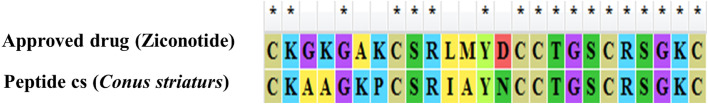
Sequence alignment between approved drug (1dw4) and peptide-cs (1fyg) sequence. * indicates the column in which fully conserved residues are present. 18 out of 25 residues are exactly similar.

To validate the model prediction accuracy, we selected a peptide sequence (peptide-cs) of *Conus striatus*, which was predicted as a positive contributor for therapeutics by our model. Alignment results (Fig 11) showed that peptide-cs sequence was closely matched to a previously FDA approved peptide sequence (Ziconotide, Prialt®) isolated from *Conus magus* [84].

**Fig 12:**
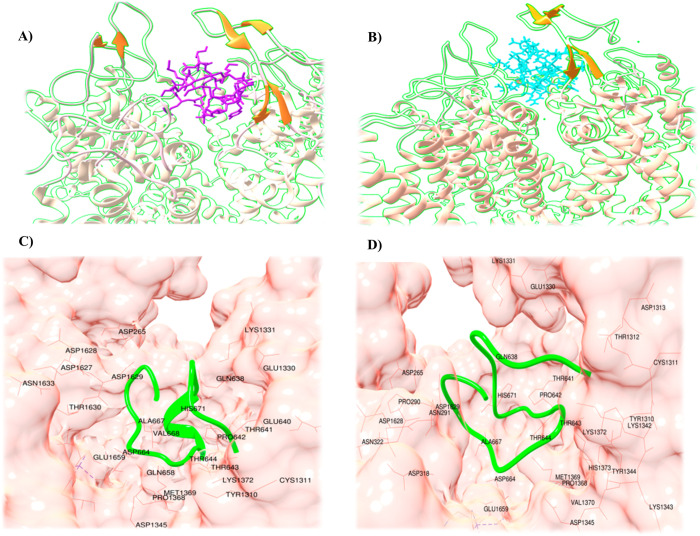
Binding analysis of Ziconotide (PDB ID: 1dw4) and *Conus striatus* peptide-cs (PDB ID: 1fyg) against Ca_v_2.2 channel (PDB ID: 7vfu). **(A)** *Conus magus* peptide is shown in magenta color and **(B)** Ziconotide is indicated in cyan color. Ca_v_2.2 ion channel is indicated in light pink colored ribbon representation. **(C and D)** Surface representation of Ca_v_2.2 channel demonstrating binding cavity (pink) for Ziconotide and peptide-cs (green ribbon), respectively.

PatchDock specific energy value for Ca_v_2.2 and peptide-cs complex was −15.28 kcal/mol. Ca_v_2.2 binding residues were quite similar for both peptides. Ziconotide exhibited binding with ASP1629, ALA667, ASP664, THE544, PRO642, HIS671, and VAL688, while peptide-cs binding was detected with ASP1629, ALA667, THR664, THR643, PRO642, HIS671, and THR641 residues of Ca_v_2.2 (Fig 12). The interaction analysis was conducted using PDBSum [70] to analyze salt bridges, disulfide bonds, hydrogen bonds, and non-bonded contacts. The interaction details are listed in S5 Table. These findings suggest that *Conus striatus* peptide-cs may exhibit promising therapeutic results like Ziconotide.

## Discussion

The current study provides a comprehensive evaluation of the structural diversity of cone snail venom peptides, focusing on the comparative analysis of Cys patterns, their frameworks, connectivity analysis, and barcoding, culminating in the development of a machine learning (ML) model to predict the therapeutic potential of venom peptides. Crude cone snail venom is enriched with multiple short (3–9 kDa) disulfide-rich peptides that primarily target the nervous system by activating membrane channels or receptors These peptides have garnered significant attention due to their biological potency and complexity [2,85].

Although cone snail peptides form unstructured motifs that are cleaved by proteases [86], they exhibit remarkable stability due to Cys disulfide linkages. These linkages help maintain a compact globular shape with a hydrophobic core, often containing a Cys residue [87]. Lavergne et al., (2015) made significant strides in identifying novel Cys patterns in cone snail venom through optimized deep-targeted proteotranscriptomic profiling. They reported nine unique *Conus* Cys frameworks and identified a total of 3,303 novel full-length conotoxin precursors belonging to nine empirical and 16 new gene superfamilies. Notably, they highlighted the existence of 212 conotoxins with pharmacologically active patterns, including the CC-C-C framework and the Inhibitor Cys Knot (ICK) [33].

In this study, we presented 5,985 cone snail sequences, encompassing both mature toxins and protein precursors, identifying 5,421 Cys-rich patterns within this dataset. The vast array of unique Cys patterns and related frameworks (including novel Cys patterns in 82 cone snail species) were systematically organized by species and patterns. Furthermore, we classified cone snail species based on common Cys patterns and a barcoding approach.

It has been established that conotoxins with multiple disulfide linkages adopt unique 3D conformations that enhance their biological activity, attributed to their Cys frameworks and connectivity [88]. In this study, we examined the Cys connectivity of 151 peptides, comparing these connectivities with venom-derived FDA-approved drugs or peptides in advanced clinical trials. We discovered numerous instances where novel peptides exhibited similar Cys patterns and connectivity profiles, indicating their potential for further validation in animal models or cell lines for therapeutic characterization.

We proposed ML model-based RF and SVM classifiers to predict the therapeutic potential of venom peptides. The RF model achieved an impressive overall accuracy of 90.48%, while the SVM model reached 85%. RF maintained high accuracy with an optimal threshold value of 0.5, outperforming SVM and indicating balanced instances across both datasets during training. Based on its superior performance, we chose RF over SVM. Khabbaz et al., also reported various ML models for predicting antimicrobial peptide toxicity based on amino acid (AA) physicochemical properties, selecting an RF model that exhibited an AUC of 0.883. The precision values for class 1 and class 0 were 83.33% and 100%, respectively, while the recall for class 1 and class 0 were 100% and 81.82%, respectively, which validated the model. The precision and recall values complemented each other, reflected in the F1-Score of 0.90 for both classes. These results were comparable to the RF model of antimicrobial peptides, which had a recall of 0.876 and an F1 score of 0.849 [89]. Additionally, a recent ML-aided screening framework for detecting antibiofilm peptides based on SVM models has been reported [90]. The proposed RF model for venom peptide classification exhibited competitive performance compared to SVM-based models such as AntiBFP, dPABBs [91], and BIOFIN [92]. While AntiBFP achieved the highest accuracy (97.9%) and MCC (0.960) for antibiofilm peptide prediction, dPABBs and BIOFIN recorded accuracies of 79.6% and 84.7%, respectively. These findings indicate that although SVM models like AntiBFP may deliver superior metrics for specific peptide classes, the RF model serves as a reliable alternative for predicting the therapeutic potential of venom peptides, underscoring its adaptability to complex peptide datasets.

Another study proposed an RF model for peptide classification based on docking studies [93], while Ahn et al., used an RF model to predict drug interactions [94]. Wu and his colleagues applied ML techniques to discover peptide-based drugs and their findings are quite comparable with proposed RF model [95].

Our model’s SHAP results are in good agreement with the SHAP analysis of the antibiofilm peptide predictions. According to Puchakayala et al., the most influential features for predicting antibiofilm activity include K, R, G, and aliphatic residues (A, L, I, and V) [90]. Similarly, to predict the therapeutic potential of venom peptides, our model proposed G, C, K, and isoelectric point as the most important features, whereas V, M, and F were the least influential.

The importance of G in both contexts can be attributed to its small molecular volume and low steric parameter, as described by Grantham [96] and Charton [97]. These properties confer peptides with greater conformational flexibility, which may aid in forming amphipathic structures necessary for biological activity. The identification of C as a key feature in our model aligns with its moderate hydrophobicity and role in forming disulfide bonds, enhancing structural stability and contributing to bioactivity, as seen in C-rich peptides reported for antibiofilm activity [98,99].

The minimal importance of V, M, and F in our model reflects their limited role in the therapeutic function of venom peptides. V’s hydrophobicity and contribution to amphipathic helices is less relevant, as venom peptides target specific receptors rather than broadly disrupting membranes. M’s sulfur side chain and F’s bulkiness may lack the precise structural or binding contributions needed for therapeutic efficacy, emphasizing the context-dependent nature of feature importance.

To validate our model’s predictive efficiency, we isolated and evaluated PDB files of available cone snail venom-derived FDA-approved drugs for connectivity analysis, comparing them with the test dataset. We identified multiple cone snail peptides sharing similar amino acid compositions, Cys patterns, or frameworks (S3 Table). For instance, peptide-cs, ziconotide (an FDA-approved peptide isolated from *Conus magus*) [16], omega conotoxin_FVI-A, and the *Conus purpurascens* peptide shared identical Cys connectivity patterns. Structural evaluation revealed close binding resemblance between peptide-cs and ziconotide against Ca_v_2.2. These peptides may potentially target Ca_v_2.2 in a manner similar to ziconotide, warranting further investigation to validate their roles in pain relief.

The Cys pattern barcoding method used in this study is specifically designed for Cys-rich venom peptides, which have well-conserved Cys frameworks and disulfide bond patterns crucial for their stability and biological activity. However, the applicability of this approach to other therapeutic peptides depends on the consistency of Cys residues across different peptide families. While Cys-rich peptides, such as defensins [100], cyclotides, and antimicrobial peptides, may share similar disulfide structures [101], peptides with more variable or flexible Cys arrangements might require modifications to the barcoding method. To broaden the scope of this approach to other peptide groups, further validation with diverse datasets spanning both venomous and non-venomous species is essential.

In conclusion, this study, grounded in Cys architecture, frameworks, and ML-assisted therapeutic value determination, may serve as a milestone for developing novel therapeutic strategies targeting specific biological pathways.

## Supporting information

S1 Fig *Conus* species having same Cys count, patterns and connectivities as FDA-approved venom-derived drug (1dw4). (DOCX)

S1 Table Sequence details of barcodes representing novel patterns among cone snail species. A comma is used to separate a subtype’s sequence from another.
(DOCX)

S2 Table Connectivity details of all the available cone snail PDB files. The “Pattern” column indicates the cysteine (Cys) pattern along with their corresponding positions in the sequence. The “C_labels” column represents the sequence-based Cys pattern, excluding connectivity information. The “Connecting_pairs” column specifies the pairs of cysteines that exhibit connectivity.
(DOCX)

S3 Table Comparative analysis of an FDA-approved drug Ziconotide (PDB ID: 1dw4) and cone snail peptides with comparable connectivity pairs. (DOCX)

S4 Table Predictions results of RF model on test dataset. (DOCX)

S5 Table Residues from *Conus magus* and *Conus striatus* peptides involved in interaction against Ca_v_2.2 channel. Common interactions are indicated by red and green colors, while black color indicates residues that were not involved in common interactions. Red color residues represent the Ca_v_2.2 channel, while green color depicts *Conus magus* and *Conus striatus* peptides, respectively.: indicates the interaction between the Ca_v_2.2 channel and peptide residues.
(DOCX)

## References

[1] Lewis RJ, Garcia ML. Therapeutic potential of venom peptides. Nat Rev Drug Discov. 2003;2(10):790–802. doi: 10.1038/nrd1197 14526382

[2] Pennington MW, Czerwinski A, Norton RS. Peptide therapeutics from venom: Current status and potential. Bioorg Med Chem. 2018;26(10):2738–58. doi: 10.1016/j.bmc.2017.09.029 28988749

[3] Trim CM, Byrne LJ, Trim SA. Utilisation of compounds from venoms in drug discovery. Prog Med Chem. 2021;60:1–66. doi: 10.1016/bs.pmch.2021.01.001 34147202

[4] Miseta A, Csutora P. Relationship between the occurrence of cysteine in proteins and the complexity of organisms. Mol Biol Evol. 2000;17(8):1232–9. doi: 10.1093/oxfordjournals.molbev.a026406 10908643

[5] Vijayasarathy M, Kumar S, Das R, Balaram P. Cysteine-free cone snail venom peptides: Classification of precursor proteins and identification of mature peptides. J Pept Sci. 2024;30(4):e3554. doi: 10.1002/psc.3554 38009400

[6] Tadokoro T, Modahl CM, Maenaka K, Aoki-Shioi N. Cysteine-rich secretory proteins (CRISPs) from venomous snakes: An overview of the functional diversity in a large and underappreciated superfamily. Toxins (Basel). 2020;12(3):175. doi: 10.3390/toxins12030175 32178374 PMC7150914

[7] Lavergne V, Alewood PF, Mobli M, King GF. CHAPTER 2. The structural universe of disulfide-rich venom peptides. Drug Discovery. Royal Society of Chemistry. 2015. p. 37–79. doi: 10.1039/9781849737876-00037

[8] Quintero-Hernández V, Jiménez-Vargas JM, Gurrola GB, Valdivia HH, Possani LD. Scorpion venom components that affect ion-channels function. Toxicon. 2013;76:328–42. doi: 10.1016/j.toxicon.2013.07.012 23891887 PMC4089097

[9] Kalita B, Utkin YN, Mukherjee AK. Current Insights in the mechanisms of cobra venom cytotoxins and their complexes in inducing toxicity: Implications in antivenom therapy. Toxins (Basel). 2022;14(12):839. doi: 10.3390/toxins14120839 36548736 PMC9780984

[10] Tan CH, Tan KY, Ng TS, Sim SM, Tan NH. Venom proteome of spine-bellied sea snake (*Hydrophis curtus*) from Penang, Malaysia: Toxicity correlation, immunoprofiling and cross-neutralization by sea snake antivenom. Toxins (Basel). 2018;11(1):3. doi: 10.3390/toxins11010003 30583590 PMC6356285

[11] Phuong MA, Mahardika GN, Alfaro ME. Dietary breadth is positively correlated with venom complexity in cone snails. BMC Genomics. 2016;17:401. doi: 10.1186/s12864-016-2755-6 27229931 PMC4880860

[12] Dutertre S, Lewis RJ. Use of venom peptides to probe ion channel structure and function. J Biol Chem. 2010;285(18):13315–20. doi: 10.1074/jbc.R109.076596 20189991 PMC2859489

[13] Bhattacharjee P, Bhattacharyya D. Therapeutic use of snake venom components: A voyage from ancient to modern India. MROC. 2014;11(1):45–54. doi: 10.2174/1570193x1101140402101043

[14] Lazarovici P. Snake- and spider-venom-derived toxins as lead compounds for drug development. Methods Mol Biol. 2020;2068:3–26. doi: 10.1007/978-1-4939-9845-6_1 31576520

[15] Harvey AL. Toxins and drug discovery. Toxicon. 2014;92:193–200. doi: 10.1016/j.toxicon.2014.10.020 25448391

[16] Brinzeu A, Berthiller J, Caillet J-B, Staquet H, Mertens P. Ziconotide for spinal cord injury-related pain. Eur J Pain. 2019;23(9):1688–700. doi: 10.1002/ejp.1445 31233255

[17] Fu Y, Li C, Dong S, Wu Y, Zhangsun D, Luo S. Discovery methodology of novel conotoxins from *Conus* species. Mar Drugs. 2018;16(11):417. doi: 10.3390/md16110417 30380764 PMC6266589

[18] Zhao Y, Antunes A. Biomedical potential of the neglected molluscivorous and vermivorous *Conus* species. Mar Drugs. 2022;20(2):105. doi: 10.3390/md20020105 35200635 PMC8878422

[19] Mas JM, Aloy P, Martí-Renom MA, Oliva B, Blanco-Aparicio C, Molina MA, et al. Protein similarities beyond disulphide bridge topology. J Mol Biol. 1998;284(3):541–8. doi: 10.1006/jmbi.1998.2194 9826496

[20] Olivera BM. *Conus* venom peptides: Reflections from the biology of clades and species. Annu Rev Ecol Syst. 2002;33(1):25–47. doi: 10.1146/annurev.ecolsys.33.010802.150424

[21] Song J, Yuan Z, Tan H, Huber T, Burrage K. Predicting disulfide connectivity from protein sequence using multiple sequence feature vectors and secondary structure. Bioinformatics. 2007;23(23):3147–54. doi: 10.1093/bioinformatics/btm505 17942444

[22] Li J, Liu H, Xiao S, Fan S, Cheng X, Wu C. De novo discovery of cysteine frameworks for developing multicyclic peptide libraries for ligand discovery. J Am Chem Soc. 2023;145(51):28264–75. doi: 10.1021/jacs.3c11856 38092662

[23] Benham CJ, Jafri MS. Disulfide bonding patterns and protein topologies. Protein Sci. 1993;2(1):41–54. doi: 10.1002/pro.5560020105 8443589 PMC2142305

[24] Robinson SD, Undheim EAB, Ueberheide B, King GF. Venom peptides as therapeutics: advances, challenges and the future of venom-peptide discovery. Expert Rev Proteomics. 2017;14(10):931–9. doi: 10.1080/14789450.2017.1377613 28879805

[25] Yousef M, Allmer J. Deep learning in bioinformatics. Turk J Biol. 2023;47(6):366–82. doi: 10.55730/1300-0152.2671 38681776 PMC11045206

[26] Yin S, Mi X, Shukla D. Leveraging machine learning models for peptide-protein interaction prediction. RSC Chem Biol. 2024;5(5):401–17. doi: 10.1039/d3cb00208j 38725911 PMC11078210

[27] Bedraoui A, Suntravat M, El Mejjad S, Enezari S, Oukkache N, Sanchez EE, et al. Therapeutic potential of snake venom: Toxin distribution and opportunities in deep learning for novel drug discovery. Med Drug Discov. 2024;21:100175. doi: 10.1016/j.medidd.2023.100175

[28] Wang G, Vaisman II, van Hoek ML. Machine learning prediction of antimicrobial peptides. Methods Mol Biol. 2022;2405:1–37. doi: 10.1007/978-1-0716-1855-4_1 35298806 PMC9126312

[29] Kaas Q, Yu R, Jin A-H, Dutertre S, Craik DJ. ConoServer: updated content, knowledge, and discovery tools in the conopeptide database. Nucleic Acids Res. 2012;40(Database issue):D325–30. doi: 10.1093/nar/gkr886 22058133 PMC3245185

[30] Bernard J. Python data analysis with pandas. Python Recipes Handbook: A Problem-Solution Approach. Apress. 2016. p. 37–48. doi: 10.1007/978-1-4842-0241-8_5

[31] Thomas GS, Thompson RC, Miyamoto MI, Ip TK, Rice DL, Milikien D, et al. The RegEx trial: a randomized, double-blind, placebo- and active-controlled pilot study combining regadenoson, a selective A(2A) adenosine agonist, with low-level exercise, in patients undergoing myocardial perfusion imaging. J Nucl Cardiol. 2009;16(1):63–72. doi: 10.1007/s12350-008-9001-9 19152130

[32] Gupta P, Bagchi A. Data manipulation with pandas. in Essentials of Python for Artificial Intelligence and Machine Learning. 2024. p. 197–235. doi: 10.1007/978-3-031-43725-0_6

[33] Lavergne V, Harliwong I, Jones A, Miller D, Taft RJ, Alewood PF. Optimized deep-targeted proteotranscriptomic profiling reveals unexplored *Conus* toxin diversity and novel cysteine frameworks. Proc Natl Acad Sci U S A. 2015;112(29):E3782-91. doi: 10.1073/pnas.1501334112 26150494 PMC4517256

[34] Knox C, Wilson M, Klinger CM, Franklin M, Oler E, Wilson A, et al. DrugBank 6.0: the DrugBank knowledgebase for 2024. Nucleic Acids Res. 2024;52(D1):D1265–75. doi: 10.1093/nar/gkad976 37953279 PMC10767804

[35] Luengo J, García-Gil D, Ramírez-Gallego S, García S, Herrera F. Big Data Preprocessing: Enabling Smart Data. Cham: Springer International Publishing. 2020. 1, p. 1–86. doi: 10.1007/978-3-030-39105-8

[36] Hao J, Ho TK. Machine learning made easy: A review of scikit-learn package in Python programming language. J Educ Behav Stat. 2019;44(3):348–61. doi: 10.3102/1076998619832248

[37] Sadaiyandi J, Arumugam P, Sangaiah AK, Zhang C. Stratified sampling-based deep learning approach to increase prediction accuracy of unbalanced dataset. Electronics. 2023;12(21):4423. doi: 10.3390/electronics12214423

[38] Sivarajan A, Bala Aditya A, Sivasankar E. Comparing the predictive accuracy of machine learning algorithms for neonatal mortality risk classification. In Advanced Machine Intelligence and Signal Processing. 2022. p. 325–39. doi: 10.1007/978-981-19-0840-8_24

[39] Shanthi DL, Chethan N. Genetic algorithm based hyper-parameter tuning to improve the performance of machine learning models. SN Comput Sci. 2022;4(2). doi: 10.1007/s42979-022-01537-8

[40] Virgolin M, Alderliesten T, Bosman PAN. On explaining machine learning models by evolving crucial and compact features. Swarm Evol Comput. 2020;53:100640. doi: 10.1016/j.swevo.2019.100640

[41] Nambiar P, Mitra D, Dutta A. Machine learning assisted screening framework for insecticidal peptides. Mater Today. 2023;72:41–6. doi: 10.1016/j.matpr.2022.05.455

[42] Mondal RK, Sen D, Arya A, Samanta SK. Developing anti-microbial peptide database version 1 to provide comprehensive and exhaustive resource of manually curated AMPs. Sci Rep. 2023;13(1):17843. doi: 10.1038/s41598-023-45016-3 37857659 PMC10587344

[43] Otović E, Njirjak M, Kalafatovic D, Mauša G. Sequential properties representation scheme for recurrent neural network-based prediction of therapeutic peptides. J Chem Inf Model. 2022;62(12):2961–72. doi: 10.1021/acs.jcim.2c00526 35704881

[44] Bolhassani A. Potential efficacy of cell-penetrating peptides for nucleic acid and drug delivery in cancer. Biochim Biophys Acta. 2011;1816(2):232–46. doi: 10.1016/j.bbcan.2011.07.006 21840374

[45] Guruprasad K, Reddy BV, Pandit MW. Correlation between stability of a protein and its dipeptide composition: a novel approach for predicting in vivo stability of a protein from its primary sequence. Protein Eng. 1990;4(2):155–61. doi: 10.1093/protein/4.2.155 2075190

[46] Lobry JR, Gautier C. Hydrophobicity, expressivity and aromaticity are the major trends of amino-acid usage in 999 Escherichia coli chromosome-encoded genes. Nucleic Acids Res. 1994;22(15):3174–80. doi: 10.1093/nar/22.15.3174 8065933 PMC310293

[47] Kyte J, Doolittle RF. A simple method for displaying the hydropathic character of a protein. J Mol Biol. 1982;157(1):105–32. doi: 10.1016/0022-2836(82)90515-0 7108955

[48] Wimley WC, White SH. Experimentally determined hydrophobicity scale for proteins at membrane interfaces. Nat Struct Biol. 1996;3(10):842–8. doi: 10.1038/nsb1096-842 8836100

[49] Saha I, Dang EK, Svatunek D, Houk KN, Harran PG. Computational generation of an annotated gigalibrary of synthesizable, composite peptidic macrocycles. Proc Natl Acad Sci U S A. 2020;117(40):24679–90. doi: 10.1073/pnas.2007304117 32948694 PMC7547232

[50] Lipinski CA, Lombardo F, Dominy BW, Feeney PJ. Experimental and computational approaches to estimate solubility and permeability in drug discovery and development settings. Adv Drug Deliv Rev. 2001;46(1–3):3–26. doi: 10.1016/s0169-409x(00)00129-0 11259830

[51] Parmar A, Katariya R, Patel V. A Review on Random Forest: An Ensemble Classifier. In International conference on intelligent data communication technologies and internet of things (ICICI) 2018. 2019. p. 758–63. doi: 10.1007/978-3-030-03146-6_86

[52] Quinlan JR. Induction of decision trees. Mach Learn. 1986;1(1):81–106. doi: 10.1007/bf00116251

[53] Vrtkova A. Predicting clinical status of patients after an acute ischemic stroke using random forests. In: 2017 International Conference on Information and Digital Technologies (IDT), 2017. p. 417–22. doi: 10.1109/dt.2017.8024330

[54] Xi E. Image classification and recognition based on deep learning and random forest algorithm. Wirel Commun Mob Comput. 2022;2022:1–9. doi: 10.1155/2022/2013181

[55] Mustafa Abdullah D, Mohsin Abdulazeez A. Machine learning applications based on SVM classification a review. QAJ. 2021;1(2):81–90. doi: 10.48161/qaj.v1n2a50

[56] Tian Y, Shi Y, Liu X. Recent advances on support vector machines research. Technol Econ Dev Econ. 2012;18(1):5–33. doi: 10.3846/20294913.2012.661205

[57] Abd Elkarim IS, Agbinya J. A review of parallel support vector machines (PSVMs) for big data classification. Aust J Basic Appl Sci. 2019. doi: 10.22587/ajbas.2019.13.12.10

[58] Chauhan VK, Dahiya K, Sharma A. Problem formulations and solvers in linear SVM: a review. Artif Intell Rev. 2018;52(2):803–55. doi: 10.1007/s10462-018-9614-6

[59] Khan SN, Khan SU, Aznaoui H, Şahin CB, Dinler ÖB. Generalization of linear and non-linear support vector machine in multiple fields: a review. Comput Sci Inf Technol. 2023;4(3):226–39. doi: 10.11591/csit.v4i3.p226-239

[60] Düntsch I, Gediga G. Confusion matrices and rough set data analysis. J Phys: Conf Ser. 2019;1229(1):012055. doi: 10.1088/1742-6596/1229/1/012055

[61] de Hond AAH, Steyerberg EW, van Calster B. Interpreting area under the receiver operating characteristic curve. Lancet Digit Health. 2022;4(12):e853–5. doi: 10.1016/S2589-7500(22)00188-1 36270955

[62] Nahm FS. Receiver operating characteristic curve: overview and practical use for clinicians. Korean J Anesthesiol. 2022;75(1):25–36. doi: 10.4097/kja.21209 35124947 PMC8831439

[63] Lundberg SM, Lee SI. A unified approach to interpreting model predictions, Adv Neural Inf Process Syst. 2017;30. doi: 10.48550/arXiv.1705.07874

[64] Molnar C, Casalicchio G, Bischl B. Interpretable machine learning – A brief history, state-of-the-art and challenges. In Joint European conference on machine learning and knowledge discovery in databases. 2020. p. 417–31. doi: 10.1007/978-3-030-65965-3_28

[65] Schneidman-Duhovny D, Inbar Y, Nussinov R, Wolfson HJ. PatchDock and SymmDock: servers for rigid and symmetric docking. Nucleic Acids Res. 2005;33(Web Server issue):W363-7. doi: 10.1093/nar/gki481 15980490 PMC1160241

[66] Andrusier N, Nussinov R, Wolfson HJ. FireDock: fast interaction refinement in molecular docking. Proteins. 2007;69(1):139–59. doi: 10.1002/prot.21495 17598144

[67] Connolly ML. Solvent-accessible surfaces of proteins and nucleic acids. Science. 1983;221(4612):709–13. doi: 10.1126/science.6879170 6879170

[68] Zhang C, Vasmatzis G, Cornette JL, DeLisi C. Determination of atomic desolvation energies from the structures of crystallized proteins. J Mol Biol. 1997;267(3):707–26. doi: 10.1006/jmbi.1996.0859 9126848

[69] Doss CGP, Chakraborty C, Chen L, Zhu H. Integrating in silico prediction methods, molecular docking, and molecular dynamics simulation to predict the impact of ALK missense mutations in structural perspective. Biomed Res Int. 2014;2014:895831. doi: 10.1155/2014/895831 25054154 PMC4098886

[70] Laskowski RA, Jabłońska J, Pravda L, Vařeková RS, Thornton JM. PDBsum: Structural summaries of PDB entries. Protein Sci. 2018;27(1):129–34. doi: 10.1002/pro.3289 28875543 PMC5734310

[71] Gong Z, Matzke NJ, Ermentrout B, Song D, Vendetti JE, Slatkin M, et al. Evolution of patterns on *Conus* shells. Proc Natl Acad Sci U S A. 2012;109(5):E234-41. doi: 10.1073/pnas.1119859109 22219366 PMC3277106

[72] Lebbe EKM, Peigneur S, Wijesekara I, Tytgat J. Conotoxins targeting nicotinic acetylcholine receptors: an overview. Mar Drugs. 2014;12(5):2970–3004. doi: 10.3390/md12052970 24857959 PMC4052327

[73] Schietgat L, Vens C, Cerri R, Fischer CN, Costa E, Ramon J, et al. A machine learning based framework to identify and classify long terminal repeat retrotransposons. PLoS Comput Biol. 2018;14(4):e1006097. doi: 10.1371/journal.pcbi.1006097 29684010 PMC5933816

[74] Enany S. Structural and functional analysis of hypothetical and conserved proteins of Clostridium tetani. J Infect Public Health. 2014;7(4):296–307. doi: 10.1016/j.jiph.2014.02.002 24802661

[75] Al-Rousan T. New framework for improving random forest classification accuracy. IJETER. 2022;10(2):67–79. doi: 10.30534/ijeter/2022/081022022

[76] Kaur PP, Singh S. Random forest classifier used for modelling and classification of herbal plants considering different features using machine learning. In Mobile Radio Communications and 5G Networks: Proceedings of Second MRCN 2021. 2022. p. 83–94. doi: 10.1007/978-981-16-7018-3_6

[77] Das P, Sachindra DA, Chanda K. Machine learning-based rainfall forecasting with multiple non-linear feature selection algorithms. Water Resour Manage. 2022;36(15):6043–71. doi: 10.1007/s11269-022-03341-8

[78] Cutler J, Dickenson M. Introduction to machine learning with Python. Computational Frameworks for Political and Social Research with Python. 2020. p. 129–42. doi: 10.1007/978-3-030-36826-5_10

[79] Han H, Jiang X. Overcome support vector machine diagnosis overfitting. Cancer Inform. 2014;13(Suppl 1):145–58. doi: 10.4137/CIN.S13875 25574125 PMC4264614

[80] Ansari MdR, Tumpa SA, Raya JAF, Murshed MN. Comparison between support vector machine and random forest for audio classification. In: 2021 International Conference on Electronics, Communications and Information Technology (ICECIT), 2021. 1–4. doi: 10.1109/icecit54077.2021.9641152

[81] Adugna T, Xu W, Fan J. Comparison of random forest and support vector machine classifiers for regional land cover mapping using coarse resolution FY-3C images. Remote Sensing. 2022;14(3):574. doi: 10.3390/rs14030574

[82] Chen T, Guestrin C. XGBoost: A scalable tree boosting system. In: Proceedings of the 22nd ACM SIGKDD International Conference on Knowledge Discovery and Data Mining, 2016. 785–94. doi: 10.1145/2939672.2939785

[83] Han R, Yoon H, Kim G, Lee H, Lee Y. Revolutionizing medicinal chemistry: The application of artificial intelligence (AI) in early drug discovery. Pharmaceuticals (Basel). 2023;16(9):1259. doi: 10.3390/ph16091259 37765069 PMC10537003

[84] Jain KK. An evaluation of intrathecal ziconotide for the treatment of chronic pain. Expert Opin Investig Drugs. 2000;9(10):2403–10. doi: 10.1517/13543784.9.10.2403 11060815

[85] Oliveira AL, Viegas MF, da Silva SL, Soares AM, Ramos MJ, Fernandes PA. The chemistry of snake venom and its medicinal potential. Nat Rev Chem. 2022;6(7):451–69. doi: 10.1038/s41570-022-00393-7 PMC9185726 35702592

[86] Mobli M, Undheim EAB, Rash LD. Modulation of ion channels by cysteine-rich peptides: From sequence to structure. Adv Pharmacol. 2017;79:199–223. doi: 10.1016/bs.apha.2017.03.001 28528669

[87] Undheim EAB, Mobli M, King GF. Toxin structures as evolutionary tools: Using conserved 3D folds to study the evolution of rapidly evolving peptides. Bioessays. 2016;38(6):539–48. doi: 10.1002/bies.201500165 27166747

[88] Heimer P, Schmitz T, Bäuml CA, Imhof D. Synthesis and structure determination of µ-Conotoxin PIIIA isomers with different disulfide connectivities. J Vis Exp. 2018;(140):58368. doi: 10.3791/58368 30346393 PMC6235375

[89] Khabbaz H, Karimi-Jafari MH, Saboury AA, BabaAli B. Prediction of antimicrobial peptides toxicity based on their physico-chemical properties using machine learning techniques. BMC Bioinformatics. 2021;22(1):549. doi: 10.1186/s12859-021-04468-y 34758751 PMC8582201

[90] Puchakayala HC, Bhatnagar P, Nambiar P, Dutta A, Mitra D. Design of a machine learning-aided screening framework for antibiofilm peptides. Digit Chem Eng. 2023;8:100107. doi: 10.1016/j.dche.2023.100107

[91] Sharma A, Gupta P, Kumar R, Bhardwaj A. dPABBs: A novel in silico approach for predicting and designing anti-biofilm peptides. Sci Rep. 2016;6:21839. doi: 10.1038/srep21839 26912180 PMC4766436

[92] Gupta S, Sharma AK, Jaiswal SK, Sharma VK. Prediction of biofilm inhibiting peptides: An in silico approach. Front Microbiol. 2016;7:949. doi: 10.3389/fmicb.2016.00949 27379078 PMC4909740

[93] Feng H, Wang F, Li N, Xu Q, Zheng G, Sun X, et al. A random forest model for peptide classification based on virtual docking data. Int J Mol Sci. 2023;24(14):11409. doi: 10.3390/ijms241411409 37511165 PMC10380188

[94] Ahn S, Lee SE, Kim M. Correction: Random-forest model for drug–target interaction prediction via Kullback–Leibler divergence. J Cheminform. 2022;14(1):67. doi: 10.1186/s13321-022-00653-0 36192818 PMC9531514

[95] Wu Q, Ke H, Li D, Wang Q, Fang J, Zhou J. Recent progress in machine learning-based prediction of peptide activity for drug discovery. Curr Top Med Chem. 2019;19(1):4–16. doi: 10.2174/1568026619666190122151634 30674262

[96] Grantham R. Amino acid difference formula to help explain protein evolution. Science. 1974;185(4154):862–4. doi: 10.1126/science.185.4154.862 4843792

[97] Charton M. Protein folding and the genetic code: an alternative quantitative model. J Theor Biol. 1981;91(1):115–23. doi: 10.1016/0022-5193(81)90377-5 7300379

[98] Zhang W, Xu X, Zhang J, Ye T, Zhou Q, Xu Y, et al. Discovery and characterization of a new crustin antimicrobial peptide from amphibalanus amphitrite. Pharmaceutics. 2022;14(2):413. doi: 10.3390/pharmaceutics14020413 35214145 PMC8877177

[99] Brogden KA. Antimicrobial peptides: pore formers or metabolic inhibitors in bacteria?. Nat Rev Microbiol. 2005;3(3):238–50. doi: 10.1038/nrmicro1098 15703760

[100] Lehrer RI, Bevins CL, Ganz T. Defensins and other antimicrobial peptides and proteins. Mucosal Immunology, Two-Volume Set. 2007. p. 95–110. doi: 10.1016/b978-012491543-5/50010-3

[101] Tyler TJ, Durek T, Craik DJ. Native and engineered cyclic disulfide-rich peptides as drug leads. Molecules. 2023;28(7):3189. doi: 10.3390/molecules28073189 37049950 PMC10096437

