# Supplementary material for: Cysteine pattern barcoding-based dataset filtration enhances the machine learning-assisted interpretation of Conus venom peptide therapeutics
Source: PLoS One. 2025 Jul 11;20(7):e0327578. doi: 10.1371/journal.pone.0327578 (PMC12250603; doi:10.1371/journal.pone.0327578)
Supplement: S1 Fig — (DOCX) [file pone.0327578.s001.docx]

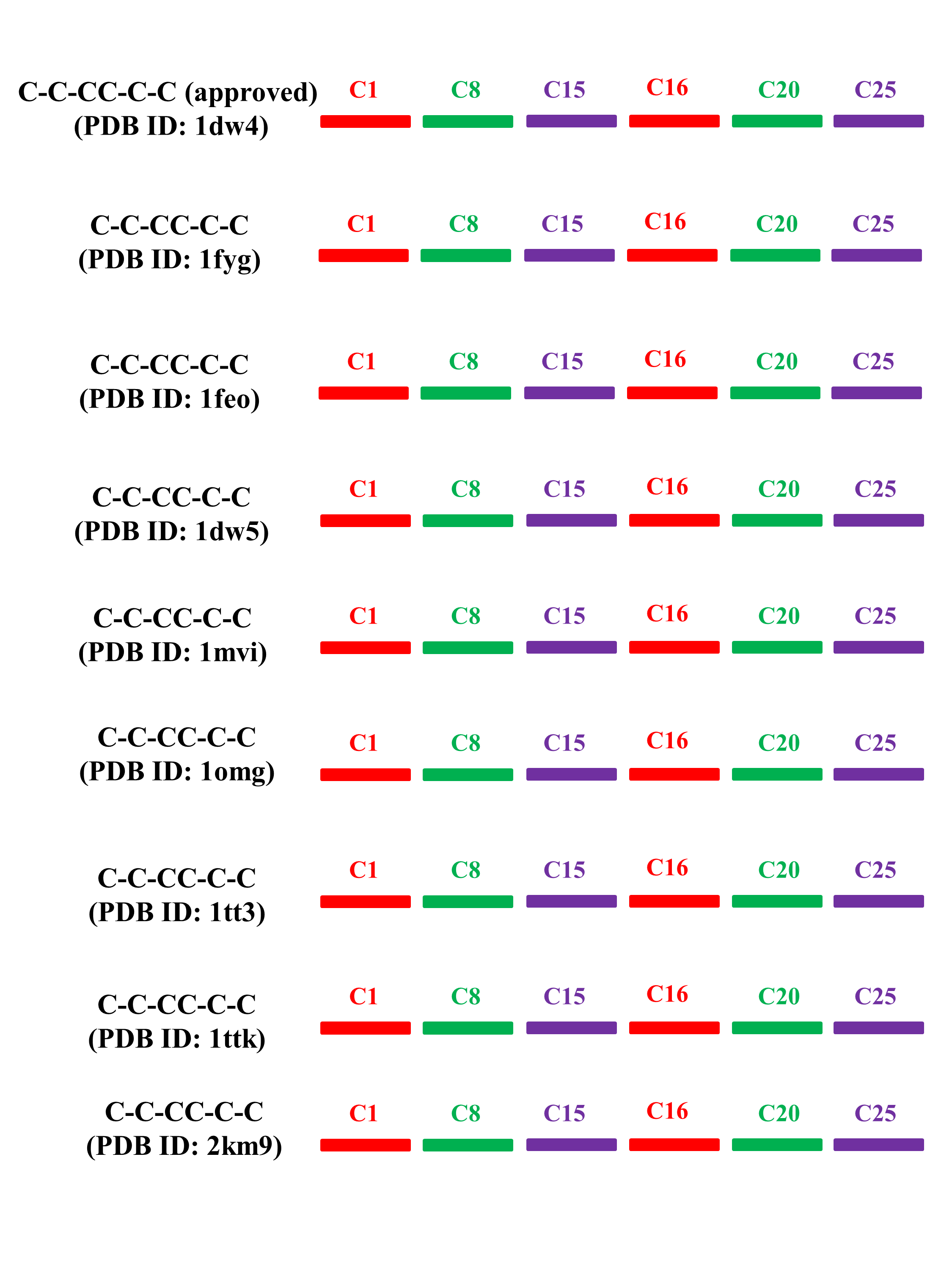


**Figure S1. Conus species having same Cys count, patterns and connectivities as FDA-approved venom-derived drug (1dw4).**
