# Supplementary material for: Cysteine pattern barcoding-based dataset filtration enhances the machine learning-assisted interpretation of Conus venom peptide therapeutics
Source: PLoS One. 2025 Jul 11;20(7):e0327578. doi: 10.1371/journal.pone.0327578 (PMC12250603; doi:10.1371/journal.pone.0327578)
Supplement: S1 Table — A comma is used to separate a subtype’s sequence from another. (DOCX) [file pone.0327578.s002.docx]

**Table S1. Sequence details of barcodes representing novel patterns among cone snail species.** A comma is used to separate a subtype's sequence from another.

| ***Specie*** | **Pattern** |
| --- | --- |
| *Conusabbreviatus* | C-CC-C-C,C-C-CC-C-CC,C-C-C-CC-C-C,C-C-C-CC-C-CC |
| *Conusachatinus* | C-C-C-CC-C-C |
| *Conusadamsonii* | C-C-C-C-C,C-C-C-C-C |
| *Conusamadis* | C-C,C-C,C-CC-C-C,C-CC-CC,C-CC-C-C-CC,C-C-C-CC-C-C |
| *Conusammiralis* | C-C-C-CC-C-C |
| *Conusaraneosus* | C-C,C-C,C-C-C-C-C-C-CC-C-C-C-C-C-CC-C-C |
| *Conusarenatus* | C,C-CC-CC,C-C-C-CC-C-C,C-CC-C-C-CC,C-C-C-C-C-CC-C-C,C-C-C-CC-CC-C-C |
| *Conusaristophanes* | C-C-C-CC-C-C |
| *Conusaulicus* | C-C-C-CC-C-C |
| *Conusaurisiacus* | C-C-C-CC-C-C |
| *Conusaustralis* | C-C |
| *Conusbayani* | C-C,C-C-C,C-C-C-C-C-C-C,C-C-C-C-C-C-C-C-C,CC-C-C-C-C-C-C-C-C-C-C-C,C-C-C-C-C-C-CC-C-C-C-C-C-C,C-CC-C-C-C-C-C-C-CC-C-C-C,CC-C-C-C-C-C-CC-C-C-C-C-C,C-C-C-CC-C-C-C-C-C-C-CC-C-C,C-CC-C-C-C-C-C-CC-C-C-C-C-C,C,C-C,C-C-C,C-CC-C-C,C-CC-CC,C-C-C-CC-C-C,C-CC-C-C-CC,C-C-C-C-CC-C-C-C,CC-C-C-CC-CC-C-C,C-CC-C-C-CC-CC-C-C,CC-C-C-C-C-C-C-C-C-C-C-C,CC-C-C-C-C-C-CC-C-C-C-C-C,C-CC-C-C-C-C-C-C-CC-C-C-C,C-C-C-C-C-C-C-CC-C-C-C-C-C-C,C-CC-C-C-C-C-C-CC-C-C-C-C-C,C-C-C-C-C-C-CC-C-C-C-C-C-CC-C-C,C-C-C-C-C-C-C-CC-C-C-C-C-C-CC-C-C,C-C-C-C-C-C-CC-C-C-C-C-C-C-CC-C-C-C |
| *Conusbetulinus* | C-C,C,C-C,C-CC-CC,C-CC-C-C-CC,CCC-C-C-C-C,C-C-C-CC-C-C,C-C-C-CC-CC-C-C |
| *Conusbrunneus* | C-C-C-CC-C-C |
| *Conusbullatus* | C-C,C-C,C-C-C-CC-C-C,C-CC-C-C-CC,CCC-C-C-C-C-C |
| *Conuscalifornicus* | C-C,C-C-CCC-C-C-C,C-C,C-C-C-C-C,C-C-C-CC,C-CC-C-C,C-C-C-CC-C-C,C-C-C-C-C-C-C,C-C-CCC-C-C-C,C-C-C-C-C-C-C-C-C,C-C-C-C-C-CC-C-C,C-C-CCC-C-CC-C-C,C-C-C-C-C-C-C-C-C-C-C |
| *Conuscapitaneus* | CC-C-C-C,CC-C-C-C,C-CC-CC,C-C-C-CC-C-C,C-CCC-C-C-C,C-CC-C-C-CC,C-C-C-C-CC-CC-C-C |
| *Conuscaracteristicus* | C-C,CC-C,CC-C-CC,C-C-CC-CC-C-C-C-C-CC-CC-C-C,C-C,CC-C,C-CC-CC,C-C-C-C-C,C-C-C-CC,C-CC-C-CC,CC-C-C-C,C-C-C-C-C-C-C,C-C-C-CC-C-C,C-CC-C-C-CC,CCC-C-C-C-C,C-C-C-C-C-CC-C-C,C-C-C-CC-CC-C-C,C-C-C-C-CC-CC-C-C,C-C-C-C-C-C-C-C-C-C-C |
| *Conuscatus* | C,C-C,C-C-C,CC-C-C-C,CC-C-C-C-C-C-C,C-C-C-CC-C-C |
| *Conusconsors* | C,C-C,C,C-C-C-CC-C-C,C-C-C-C-CC-C,C-CC-C-C-CC |
| *Conuscoronatus* | C-CC-CC,C-CC-C-C-CC,C-C-C-CC-C-C |
| *Conusdalli* | C-C-C-CC-C-C |
| *Conusdistans* | C-C,C-C,C-C-C-CC-C-C,CCC-C-C-C-C-C-C,C-C-C-C-C-C-C-C-C-C-C |
| *Conusebraeus* | C-C,CC-C-C-C,C-CC-C-C,C-C,CC-C-C-C,C-CC-CC,C-CC-C-C-CC,C-C-C-CC-C-C,C-C-C-C-C-C-CC-C-C-C-C-C-CC-C-C |
| *Conuseburneus* | C,C-C,C,C-C,C-CC-CC,C-C-C-C-C,CCC-C-C-C-C,C-C-C-CC-C-C,C-CC-C-C-CC,C-C-C-CC-CC-C-C,C-C-C-C-C-CC-C-C,C-C-C-C-CC-CC-C-C |
| *Conusemaciatus* | C-C,CC-C,C-C,C-CC-CC,C-CC-C-C-CC,C-C-C-C-C-CC-C-C,C-C-C-C-CC-CC-C-C |
| *Conusepiscopatus* | C,C-C-C-CC-C-C,C-C-C-CC-CC-C-C |
| *Conusermineus* | C-C,C,C-C,C-CC-CC,C-CC-C-C,C-C-C-CC-C-C,C-C-C-C-C-C-C,CC-C-C-C-C-C,C-C-C-C-C-C-C-C-C-C-C-C-C-C-C-C-C-C-C |
| *Conusferrugineus* | CCC-C-C-C-C,C-C-C-CC-CC-C-C |
| *Conusfigulinus* | C-C |
| *Conusflavidus* | C-C-C-CC,CC-C-C-CC-C,C,CC-C-C-CC-C,C-CC-C-C-CC,C-C-C-CC-C-C |
| *Conusfloridulus* | C-C-C,C-CC-C-C,C-C-C-C-C-CC-C-C |
| *Conusfrigidus* | C-C,C-C,C-C-C-CC-C-C,C-C-C-C-C-CC-C-C-C-C-C-CC-C-C |
| *Conusgeneralis* | C-CC-CC,C-C-C-C-C-C-C,C-C-C-CC-C-C |
| *Conusgeographus* | C-C,C-C-C,C-CC-C-C,C-C-CC-C-C-C,C-C-C-CC-C-C,C-C-CCC-C-C-C,CC-C-CC-C-C-C-C,CC-C-C-C-CC-C-C-C-C,C-C,C-CC-CC,C-C-C-C-C,CC-CC-CC,C-C-C-CC-C-C,CCC-C-C-C-C,C-C-C-C-C-C-C,C-CC-C-C-CC,C-C-CCC-C-C-C,CC-C-CC-C-C-C-C,C-C-C-C-C-CC-C-C,C-C-C-C-C-CC-C-C-C,CC-C-C-C-CC-C-C-C-C,C-C-C-C-C-C-C-C-C-C-C,C-C-C-C-C-C-CC-C-C-C-C-C-CC-C-C |
| *Conusgloriamaris* | C-CC-CC,C-C-C-CC-C-C,C-CC-C-C-CC |
| *Conusimperialis* | C-C,C-C-C,C-C-CC-C,C-C-C-C-C,C-CC-C-C,C-C-C-CC-C-C,C-C-C-C-C-C-CC,C-C-CC-CC-C-C-C-C,C,C-C-CC-C,C-CC-CC,CC-CC-CC,C-C-C-CC-C-C,CC-C-C-C-C-C,CCC-C-C-C-C,C-CC-C-C-CC,C-C-C-C-CC-C,C-C-C-C-C-C-CC,C-C-C-CC-CC-C-C,C-C-C-C-C-CC-C-C,C-C-C-C-C-C-C-C-C,C-C-C-C-CC-CC-C-C,C-C-CC-CC-C-C-C-C,C-C-CC-C-CC-C-C-C-C,C-C-C-C-CC-C-C-C-C-C |
| *Conusinscriptus* | C-C |
| *Conusjudaeus* | C-C-C-CC-C-C |
| *Conuskinoshitai* | C-C,C-C-C-CC-C-C |
| *Conuskintoki* | C-C-C-CC-C-C |
| *Conusleopardus* | C-CC-CC,C-C-C-CC-C-C |
| *Conuslitteratus* | C-C,C-C-C,CC-C,CC-C-C-C,CCC-C-C-C-C,C-C-C-CC-C-C,C-CC-C-C-C-C,C-C-C-C-C-C-C,C-C-C-C-C-C-C-C-C,CC-C-C-C-C-C-CC-C-C-C-C-C,CC-C-C-C-C-C-C-C-C-C-C-C-CC-C-C-C-C-C,C,C-C,C-C-C-C-C,C-CC-CC,CCC-C-C-C-C,C-CC-C-C-CC,C-C-C-CC-C-C,C-C-C-C-C-C-C,C-C-C-CC-CC-C-C,C-C-C-C-C-CC-C-C,C-C-C-C-CC-C-C-C-C-C,C-CC-C-C-CC-CC-C-C,C-C-C-C-C-C-CC-C-C-C-C-C,CC-C-C-C-C-C-CC-C-C-C-C-C,C-CC-C-C-C-C-C-C-C-C-C-C-C-CC-C-C-C-C-C |
| *Conuslividus* | C-C,CC-C,CC-C-C-CC-C,C-C,CC-C,C-CC-CC,C-CC-C-C-CC,CC-C-C-CC-C,C-C-C-CC-C-C,C-C-C-C-C-CC-C-C-C-C-C-CC-C-C,C-C-C-C-C-C-C-CC-C-C-C-C-C-CC-C-C |
| *Conusloroisii* | C-C,C-C,C-C-C-C-C-C-CC-C-C-C-C-C-CC-C-C |
| *Conusmagus* | C-C-C,C-C-C-C-C-C-C-C-C,C-C-C-CC-C-C |
| *Conusmarmoreus* | C,CC,C-C,C-C-C,C-C-CC-C,CC-CC-C,C-CC-C-C,CC-C-C-C,C-C-CC-C-C-C,CC-C-C-CC-C,CC-C-C-C-C-C-C,C,C-C,C-C-C,C-CC,C-CC-CC,C-CC-C-C,C-C-C-C-C,CC-C-C-C,C-CC-CC-C,C-CC-C-C-CC,C-C-C-CC-C-C,CC-C-C-CC-C,C-C-CC-C-C-C,CCC-C-C-C-C,CC-C-C-C-C-C-C,C-C-C-C-C-CC-C-C,CC-C-CC-C-C-CC,C-C-C-C-C-CC-C-C-C,C-C-C-C-CC-CC-C-C,C-C-C-C-C-C-C-C-C-C-C,C-C-C-C-C-CC-C-C-C-C |
| *Conusmemiae* | C-C-C,C-CC-C-C,C-C-C-C-C-CC-C-C |
| *Conusmiles* | C-C,C-C-CC-C-C-C,C-C,C-CC-CC,CC-CC-CC,C-C-C-CC-C-C,C-CC-C-C-CC,C-C-C-C-CC-CC-C-C,C-CC-C-C-CC-CC-C-C,C-C-C-C-C-C-C-CC-C-C-C-C-C-CC-C-C |
| *Conusmiliaris* | C-C,C-C-C-CC-C-C |
| *Conusmonile* | C-C,C-C,C-C-C-C-C-CC-C-C-C-C-C-CC-C-C,C-C-C-C-C-C-CC-C-C-C-C-C-CC-C-C,C-C-C-C-C-C-C-CC-C-C-C-C-C-CC-C-C |
| *Conusomaria* | C-C-C-CC-C-C |
| *Conusparius* | C-C,C-C,C-CC-C-C-CC |
| *Conuspennaceus* | C-C,C,C-C-C,C-CC-CC,C-CC-C-C,CC-CC-CC,C-C-C-CC-C-C |
| *Conuspictus* | C-C |
| *Conusplanorbis* | CCC-C-C-C-C |
| *Conuspulicarius* | C-C,C-CC-CC,C-C-CC-C-CC,C,C-C,C-CC-CC,C-CC-C-C,C-C-C-CC-C-C,C-CC-C-C-CC,C-C-C-C-C-C-C,C-C-C-CC-C-CC,C-C-C-C-C-C-CC-C,C-C-C-CC-CC-C-C,C-CC-C-C-CC-CC-C-C |
| *Conuspurpurascens* | C-C,C-C-C,C-C-C-C-C-C-C-C-C,C-C,C-C-C,C-C-C-CC-C-C,C-CC-C-C-CC |
| *Conusquercinus* | C-C-C,CC-C,C-C-C-C-C,C-CC-C-C,C-C-CC-C-C-C,CC-C-C-C-C-CC-C-C-C-C,C-C,C-C-C,CC-C,C-CC-CC,C-C-C-CC-C-C,C-CC-C-C-CC,C-C-CC-C-C-C,C-C-C-C-C-CC-C-C,C-C-C-CC-CC-C-C,CC-C-C-C-C-CC-C-C-C-C |
| *Conusradiatus* | C-C,C-C,C-CC-C-C-CC,C-C-C-CC-CC-C-C |
| *Conusrattus* | C-CC-CC,C-CC-C-C-CC,C-C-CC-C-C-CC |
| *Conusregius* | C-CC-C-C-CC,C-CC-C-C-CC |
| *Conussanguinolentus* | CC-C,CC-C,CC-C-C-C,C-C-C-CC-C-C,C-C-C-C-CCC-C-C |
| *Conussponsalis* | C-C-C-CC-C-C |
| *Conusspurius* | C-CC-CC,C-C-C-CC-CC-C-C,C-C-C-C-CC-CC-C-C |
| *Conusstercusmuscarum* | C-C,C-C,C-C-C-CC-C-C |
| *Conusstriatus* | C-C,CC-C-C-C,CCC-C-C,C-CC-C-C,C,C-C,C-CC-CC,CCC-C-C,CC-C-C-C,C-CC-C-C,C-CC-C-C-CC,C-C-C-CC-C-C,C-C-C-C-C-CC,C-C-C-C-C-C-C,C-C-C-CC-CC-C-C,C-C-C-C-CC-CC-C-C,C-CC-CC-C-C-C-CC-C-C-C-C |
| *Conusstriolatus* | C-C-C-CC-C-C,C-C-C-CC-CC-C-C |
| *Conussulcatus* | C,C |
| *Conusterebra* | C-CC-CC,C-C-C-CC-C-C |
| *Conustessulatus* | CC-C,C,C-CC-CC,C-CC-C-C-CC,C-C-C-CC-C-C |
| *Conustextile* | C-C,C-C-C,CC-CCC,C-CC-C-C,C,C-C,C-CC-CC,C-CC-CCC,CC-CC-CC,C-C-C-CC-C-C,C-CC-C-C-CC,C-C-C-CC-CC-C-C,C-C-C-C-C-CC-C-C |
| *Conustulipa* | C,C-C,C-C-C,CC-C,C-C-CC-C,CC-CC-C-C,C-C-C-CC-C-C,C-C-CC-C-C-C,C-CC-C-C-CC,C-C-CCC-C-C-C,CC-CC-C-C-C-CC-C-C-C,C,C-C,C-C-C-C-C,C-C-CC-C,CC-CC-C-C,C-C-C-CC-C-C,C-CC-C-C-CC,C-C-C-C-C-C-C,C-C-CCC-C-C-C,C-C-C-C-C-CC-C-C-C,C-C-C-C-C-C-CC-C-C-C-C,CC-CC-C-C-C-CC-C-C-C,C-C-C-C-C-C-CC-C-C-C-C-C-CC-C-C |
| *Conusvarius* | C-CC-CC,C-CC-C-C-CC,C-C-C-C-C-CC,C-C-C-CC-C-C |
| *Conusventricosus* | C-C,C,C-CC-CC,C-C-C-CC-C-C,C-CC-C-C-CC |
| *Conusvexillum* | CC-C-C-C,CC-C-C-C,C-CC-CC,C-C-CC-C,C-C-C-CC-C-C,C-CCC-C-C-C,C-CC-C-C-CC,C-C-CC-C-C-CC,C-C-C-C-CC-CC-C-C |
| *Conusvictoriae* | C-C,C-C-C,C-C-CC-C,CC-CCC,C-C-C-C-C-C-C-C-C,C-C-C-C-CC-C-C-C-C,C,C-C,C-CC-CC,C-CC-C-C,C-CC-CCC,C-C-C-CC-C-C,C-CC-C-C-CC,CCC-C-C-C-C,C-C-C-C-C-C-C-C-C,C-C-C-CC-CC-C-C,C-C-C-C-C-CC-C-C,C-C-C-C-C-C-C-C-C-C-C,C-C-C-C-C-C-C-C-CC-C-C-C-C |
| *Conusvillepinii* | C-C,C-C-C-C-C-C-C |
| *Conusvirgo* | C-C,C,C-C,C-CC-CC,C-C-C-CC-C-C,CCC-C-C-C-C,C-C-C-C-CC-CC-C-C |
| *Conusvitulinus* | C-C,C-C,CCC-C-C-C-C,C-CC-C-C-CC,C-C-C-CC-CC-C-C |
| *Conuszeylanicus* | C-C |
| *Conuszonatus* | C-C |
