# Supplementary material for: Cysteine pattern barcoding-based dataset filtration enhances the machine learning-assisted interpretation of Conus venom peptide therapeutics
Source: PLoS One. 2025 Jul 11;20(7):e0327578. doi: 10.1371/journal.pone.0327578 (PMC12250603; doi:10.1371/journal.pone.0327578)
Supplement: S2 Table — The “Pattern” column indicates the cysteine (Cys) pattern along with their corresponding positions in the sequence. The “C_labels” column represents the sequence-based Cys pattern, excluding connectivity information. The “Connecting_pairs” column specifies the pairs of cysteines that exhibit connectivity. (DOCX) [file pone.0327578.s003.docx]

**Table S2: Connectivity details of all the available cone snail PDB files**. The "Pattern" column indicates the cysteine (Cys) pattern along with their corresponding positions in the sequence. The "C_labels" column represents the sequence-based Cys pattern, excluding connectivity information. The "Connecting_pairs" column specifies the pairs of cysteines that exhibit connectivity.

| **PDB ID's** | **Pattern** | **C_labels** | **Connecting_pairs** |
| --- | --- | --- | --- |
| 1a0m_A | C2-C3-C8-C16 | CC-C-C | (2,8)(3,16) |
| 1ag7 | C2-C9-C13-C14-C19-C27 | C-C-CC-C-C | (2,14)(9,19)(13,27) |
| 1akg | C2-C3-C8-C16 | CC-C-C | (2,8)(3,16) |
| 1as5 | C4-C5-C10-C16-C21-C22 | CC-C-C-CC | (4,16)(5,21)(10,22) |
| 1av3 | C1-C8-C15-C16-C20-C26 | C-C-CC-C-C | (1,16)(8,20)(15,26) |
| 1b45 | C3-C4-C8-C14 | CC-C-C | (3,8)(4,14) |
| 1cnl | C2-C3-C8-C12 | CC-C-C | (2,8)(3,12) |
| 1cnn | C1-C8-C15-C16-C20-C26 | C-C-CC-C-C | (1,16)(8,20)(15,26) |
| 1d7t | C2-C8 | C-C | (2,8) |
| 1dfy | C2-C8 | C-C | (2,8) |
| 1dfz | C2-C8 | C-C | (2,8) |
| 1dg0 | C2-C8 | C-C | (2,8) |
| 1dg2 | C2-C3-C8-C15 | CC-C-C | (2,8)(3,15) |
| 1dw4 | C1-C8-C15-C16-C20-C25 | C-C-CC-C-C | (1,16)(8,20)(15,25) |
| 1dw5 | C1-C8-C15-C16-C20-C25 | C-C-CC-C-C | (1,16)(8,20)(15,25) |
| 1e74 | C2-C3-C8-C12 | CC-C-C | (2,8)(3,12) |
| 1e75 | C2-C3-C8-C12 | CC-C-C | (2,8)(3,12) |
| 1e76 | C2-C3-C8-C12 | CC-C-C | (2,8)(3,12) |
| 1eyo | C2-C9-C13-C14-C19-C24 | C-C-CC-C-C | (2,14)(9,19)(13,24) |
| 1f3k | C1-C8-C15-C16-C20-C24 | C-C-CC-C-C | (1,16)(8,20)(15,24) |
| 1feo | C1-C8-C15-C16-C20-C25 | C-C-CC-C-C | (1,16)(8,20)(15,25) |
| 1fu3 | C2-C9-C16-C17-C21-C26 | C-C-CC-C-C | (2,17)(9,21)(16,26) |
| 1fyg | C1-C8-C15-C16-C20-C25 | C-C-CC-C-C | (1,16)(8,20)(15,25) |
| 1g1p | C3-C10-C20-C21-C25-C29 | C-C-CC-C-C | (3,21)(10,25)(20,29) |
| 1g1z | C3-C10-C20-C21-C25-C29 | C-C-CC-C-C | (3,21)(10,25)(20,29) |
| 1g2g | C2-C3-C8-C12 | CC-C-C | (2,8)(3,12) |
| 1gib | C3-C4-C10-C15-C20-C21 | CC-C-C-CC | (3,15)(4,20)(10,21) |
| 1hje | C2-C3-C7-C13 | CC-C-C | (2,7)(3,13) |
| 1ien | C5-C6-C11-C19 | CC-C-C | (5,11)(6,19) |
| 1ieo | C4-C5-C10-C13 | CC-C-C | (4,13)(5,10) |
| 1im1 | C2-C3-C8-C12 | CC-C-C | (2,8)(3,12) |
| 1imi_19th | C2-C3-C8-C12 | CC-C-C | (2,8)(3,12) |
| 1imi_1st | C2-C3-C8-C12 | CC-C-C | (2,8)(3,12) |
| 1ixt | C2-C6-C12-C16-C18-C23 | C-C-C-C-C-C | (2,16)(6,18)(12,23) |
| 1jlo | C4-C5-C10-C16-C21-C22 | CC-C-C-CC | (4,16)(5,21)(10,22) |
| 1jlp | C4-C5-C10-C16-C21-C22 | CC-C-C-CC | (4,16)(5,21)(10,22) |
| 1k64 | C4-C5-C10-C18 | CC-C-C | (4,10)(5,18) |
| 1kcp | C1-C8-C15-C16-C20-C26 | C-C-CC-C-C | (1,16)(8,20)(15,26) |
| 1m2c | C2-C3-C8-C16 | CC-C-C | (2,8)(3,16) |
| 1mii | C2-C3-C8-C16 | CC-C-C | (2,8)(3,16) |
| 1mtq | C5-C6-C11-C19 | CC-C-C | (5,11)(6,19) |
| 1mvi | C1-C8-C15-C16-C20-C25 | C-C-CC-C-C | (1,16)(8,20)(15,25) |
| 1mvj | C1-C8-C15-C16-C20-C26 | C-C-CC-C-C | (1,16)(8,20)(15,26) |
| 1mxn | C2-C3-C8-C15 | CC-C-C | (2,8)(3,15) |
| 1mxp | C2-C3-C8-C15 | CC-C-C | (2,15)(3,8) |
| 1not | C2-C3-C7-C13 | CC-C-C | (2,7)(3,13) |
| 1nxn | C3-C9 | C-C | (3,9) |
| 1omc | C1-C8-C15-C16-C19-C26 | C-C-CC-C-C | (1,16)(8,19)(15,26) |
| 1omg | C1-C8-C15-C16-C20-C25 | C-C-CC-C-C | (1,16)(8,20)(15,25) |
| 1omn | C1-C8-C15-C16-C20-C26 | C-C-CC-C-C | (1,16)(8,20)(15,26) |
| 1p1p | C2-C3-C11-C14-C16-C23 | CC-C-C-C-C | (2,16)(3,11)(14,23) |
| 1pen | C2-C3-C8-C16 | CC-C-C | (2,8)(3,16) |
| 1pqr | C2-C3-C11-C14-C16-C24 | CC-C-C-C-C | (2,16)(3,11)(14,24) |
| 1q2j | C3-C4-C10-C15-C21-C22 | CC-C-C-CC | (3,15)(4,21)(10,22) |
| 1qfb | C2-C8 | C-C | (2,8) |
| 1qmw | C2-C3-C7-C13 | CC-C-C | (2,7)(3,13) |
| 1qs3 | C1-C6 | C-C | (1,6) |
| 1r9i | C4-C5-C11-C16-C21-C22 | CC-C-C-CC | (4,16)(5,21)(11,22) |
| 1rmk | C2-C9-C19-C20-C25-C30 | C-C-CC-C-C | (2,20)(9,25)(19,30) |
| 1tcg | C3-C4-C10-C15-C20-C21 | CC-C-C-CC | (3,15)(4,20)(10,21) |
| 1tch | C3-C4-C10-C15-C20-C21 | CC-C-C-CC | (3,15)(4,20)(10,21) |
| 1tcj | C3-C4-C10-C15-C20-C21 | CC-C-C-CC | (3,15)(4,20)(10,21) |
| 1tck | C3-C4-C10-C15-C20-C21 | CC-C-C-CC | (3,15)(4,20)(10,21) |
| 1tr6 | C1-C8-C15-C16-C19-C26 | C-C-CC-C-C | (1,16)(8,19)(15,26) |
| 1tt3 | C1-C8-C15-C16-C20-C25 | C-C-CC-C-C | (1,16)(8,20)(15,25) |
| 1ttk | C1-C8-C15-C16-C20-C25 | C-C-CC-C-C | (1,16)(8,20)(15,25) |
| 1ttl | C1-C8-C15-C16-C19-C26 | C-C-CC-C-C | (1,16)(8,19)(15,26) |
| 1ul2 | C2-C3-C8-C16 | CC-C-C | (2,8)(3,16) |
| 1v4q | C1-C8-C15-C16-C20-C26 | C-C-CC-C-C | (1,16)(8,20)(15,26) |
| 1wct | C2-C3-C8-C9 | CC-CC | (2,8)(3,9) |
| 1xga | C2-C3-C7-C13 | CC-C-C | (2,7)(3,13) |
| 1xgb | C2-C3-C7-C13 | CC-C-C | (2,13)(3,7) |
| 1xgc | C2-C3-C7-C13 | CC-C-C | (2,3)(7,13) |
| 1y62 | C7-C32-C53-C57 | C-C-C-C | (7,57)(32,53) |
| 1yz2 | C1-C8-C15-C16-C20-C24 | C-C-CC-C-C | (1,16)(8,20)(15,24) |
| 1zlc | C4-C5-C10-C18 | CC-C-C | (4,10)(5,18) |
| 2ajw | C2-C3-C8-C16 | CC-C-C | (2,8)(3,16) |
| 2ak0 | C2-C3-C8-C16 | CC-C-C | (2,8)(3,16) |
| 2bc7 | C3-C12 | C-C | (3,12) |
| 2br8_A | C125-C138-C188-C189 | C-C-CC | (125,138)(188,189) |
| 2c9t_A | C125-C138-C188-C189 | C-C-CC | (125,138)(188,189) |
| 2cco | C1-C8-C15-C16-C19-C26 | C-C-CC-C-C | (1,16)(8,19)(15,26) |
| 2efz | C2-C3-C8-C12-C14-C15 | CC-C-C-CC | (2,14)(3,12)(8,15) |
| 2fqc | C7-C11-C22-C24 | C-C-C-C | (7,22)(11,24) |
| 2fr9 | C2-C3-C7-C13 | CC-C-C | (2,7)(3,13) |
| 2frb | C2-C3-C7-C13 | CC-C-C | (2,7)(3,13) |
| 2gcz | C2-C3-C8-C16 | CC-C-C | (2,8)(3,16) |
| 2h8s | C2-C3-C8-C16 | CC-C-C | (2,8)(3,16) |
| 2i28 | C2-C3-C8-C13 | CC-C-C | (2,8)(3,13) |
| 2ifi | C2-C3-C8-C12 | CC-C-C | (2,12)(3,8) |
| 2ifj | C2-C3-C8-C12 | CC-C-C | (2,12)(3,8) |
| 2ifz | C2-C3-C8-C12 | CC-C-C | (2,12)(3,8) |
| 2igu | C2-C3-C8-C12 | CC-C-C | (2,12)(3,8) |
| 2j6d | C10-C35-C56-C60 | C-C-C-C | (10,60)(35,56) |
| 2jry | C5-C12-C18-C19-C21-C22-C27-C38 | C-C-CC-CC-C-C | (5,19)(12,22)(18,27)(21,38) |
| 2jtu | C5-C12-C18-C19-C21-C22-C27-C38 | C-C-CC-CC-C-C | (5,19)(12,22)(18,27)(21,38) |
| 2juq | C2-C3-C8-C12 | CC-C-C | (2,8)(3,12) |
| 2jur | C2-C3-C8-C12 | CC-C-C | (2,8)(3,12) |
| 2jut | C2-C3-C8-C12 | CC-C-C | (2,8)(3,12) |
| 2km9 | C1-C8-C15-C16-C20-C25 | C-C-CC-C-C | (1,16)(8,20)(15,25) |
| 2lo9 | C5-C6-C13-C17-C23-C24 | CC-C-C-CC | (5,17)(6,23)(13,24) |
| 2loc | C5-C6-C13-C17-C23-C24 | CC-C-C-CC | (5,17)(6,23)(13,24) |
| 2lxg | C1-C2-C4-C9-C15-C16 | CC-C-C-CC | (1,15)(2,9)(4,16) |
| 2lz5 | C2-C3-C8-C16 | CC-C-C | (2,8)(3,16) |
| 2m3i | C2-C3-C8-C15 | CC-C-C | (2,8)(3,15) |
| 2m61 | C1-C2-C6-C10-C13-C14 | CC-C-C-CC | (1,14)(2,10)(6,13) |
| 2m62 | C3-C4-C9-C12 | CC-C-C | (3,12)(4,9) |
| 2m6c | C2-C8 | C-C | (2,8) |
| 2m6d | C2-C8 | C-C | (2,8) |
| 2m6e | C2-C8 | C-C | (2,8) |
| 2m6f | C2-C8 | C-C | (2,8) |
| 2m6g | C2-C8 | C-C | (2,8) |
| 2m6h | C2-C8 | C-C | (2,8) |
| 2md6 | C3-C4-C9-C17 | CC-C-C | (3,9)(4,17) |
| 2mdq | C2-C3-C8-C16 | CC-C-C | (2,8)(3,16) |
| 2mfx | C3-C16 | C-C | (3,16) |
| 2mfy | C3-C16 | C-C | (3,16) |
| 2mg6 | C2-C8 | C-C | (2,8) |
| 2mto_11th | C3-C12 | C-C | (3,12) |
| 2mto_19th | C3-C12 | C-C | (3,12) |
| 2mto_1st | C3-C12 | C-C | (3,12) |
| 2mto_20th | C3-C12 | C-C | (3,12) |
| 2mtt | C2-C8 | C-C | (2,8) |
| 2mtu | C2-C8 | C-C | (2,8) |
| 2ns3 | C2-C3-C8-C13 | CC-C-C | (2,13)(3,8) |
| 2p4l | C5-C12-C18-C19-C21-C22-C27-C38 | C-C-CC-CC-C-C | (5,19)(12,22)(18,27)(21,38) |
| 2yen | C3-C4-C10-C15-C21-C22 | CC-C-C-CC | (3,15)(4,21)(10,22) |
| 3zkt | C2-C3-C9-C10 | CC-CC | (2,9)(3,10) |
| 4ttl | C2-C3-C8-C16 | CC-C-C | (2,8)(3,16) |
