# Supplementary material for: Cysteine pattern barcoding-based dataset filtration enhances the machine learning-assisted interpretation of Conus venom peptide therapeutics
Source: PLoS One. 2025 Jul 11;20(7):e0327578. doi: 10.1371/journal.pone.0327578 (PMC12250603; doi:10.1371/journal.pone.0327578)
Supplement: S3 Table — (DOCX) [file pone.0327578.s004.docx]

**Table S3. Comparative analysis of an FDA-approved drug Ziconotide (PDB ID: 1dw4) and cone snail peptides with comparable connectivity pairs.**

| **ID** | ***Organism_name*** | **Pattern** | **C_labels** | **Pair** |
| --- | --- | --- | --- | --- |
| 1omc | *Conus geographus* | C1-C8-C15-C16-C19-C26 | C-C-CC-C-C | (1,16)(8,19)(15,26) |
| 1tr6 | *Conus geographus* | C1-C8-C15-C16-C19-C26 | C-C-CC-C-C | (1,16)(8,19)(15,26) |
| 1ttl | *Conus geographus* | C1-C8-C15-C16-C19-C26 | C-C-CC-C-C | (1,16)(8,19)(15,26) |
| 2cco | *Conus geographus* | C1-C8-C15-C16-C19-C26 | C-C-CC-C-C | (1,16)(8,19)(15,26) |
| 1f3k | *Conus textile* | C1-C8-C15-C16-C20-C24 | C-C-CC-C-C | (1,16)(8,20)(15,24) |
| 1yz2 | *Conus amadis* | C1-C8-C15-C16-C20-C24 | C-C-CC-C-C | (1,16)(8,20)(15,24) |
| 1dw4 | *Conus magus* | C1-C8-C15-C16-C20-C25 | C-C-CC-C-C | (1,16)(8,20)(15,25) |
| 1dw5 | *Conus magus* | C1-C8-C15-C16-C20-C25 | C-C-CC-C-C | (1,16)(8,20)(15,25) |
| 1feo | *Conus magus* | C1-C8-C15-C16-C20-C25 | C-C-CC-C-C | (1,16)(8,20)(15,25) |
| 1fyg | *Conus striatus* | C1-C8-C15-C16-C20-C25 | C-C-CC-C-C | (1,16)(8,20)(15,25) |
| 1mvi | *Conus magus* | C1-C8-C15-C16-C20-C25 | C-C-CC-C-C | (1,16)(8,20)(15,25) |
| 1omg | *Conus magus* | C1-C8-C15-C16-C20-C25 | C-C-CC-C-C | (1,16)(8,20)(15,25) |
| 1tt3 | *Conus magus* | C1-C8-C15-C16-C20-C25 | C-C-CC-C-C | (1,16)(8,20)(15,25) |
| 1ttk | *Conus magus* | C1-C8-C15-C16-C20-C25 | C-C-CC-C-C | (1,16)(8,20)(15,25) |
| 2km9 | *omega_conotoxin-FVIA (protein)* | C1-C8-C15-C16-C20-C25 | C-C-CC-C-C | (1,16)(8,20)(15,25) |
| 1av3 | *Conus purpurascens* | C1-C8-C15-C16-C20-C26 | C-C-CC-C-C | (1,16)(8,20)(15,26) |
| 1cnn | *Conus magus* | C1-C8-C15-C16-C20-C26 | C-C-CC-C-C | (1,16)(8,20)(15,26) |
| 1kcp | *Conus purpurascens* | C1-C8-C15-C16-C20-C26 | C-C-CC-C-C | (1,16)(8,20)(15,26) |
| 1mvj | *Conus striatus* | C1-C8-C15-C16-C20-C26 | C-C-CC-C-C | (1,16)(8,20)(15,26) |
| 1omn | *Conus magus* | C1-C8-C15-C16-C20-C26 | C-C-CC-C-C | (1,16)(8,20)(15,26) |
| 1v4q | *Conus magus* | C1-C8-C15-C16-C20-C26 | C-C-CC-C-C | (1,16)(8,20)(15,26) |
| 1eyo | *Conus tulipa* | C2-C9-C13-C14-C19-C24 | C-C-CC-C-C | (2,14)(9,19)(13,24) |
| 1ag7 | *Conus geographus* | C2-C9-C13-C14-C19-C27 | C-C-CC-C-C | (2,14)(9,19)(13,27) |
| 1fu3 | *Conus textile* | C2-C9-C16-C17-C21-C26 | C-C-CC-C-C | (2,17)(9,21)(16,26) |
| 1rmk | *Conus marmoreus* | C2-C9-C19-C20-C25-C30 | C-C-CC-C-C | (2,20)(9,25)(19,30) |
| 1g1p | *Conus ermineus* | C3-C10-C20-C21-C25-C29 | C-C-CC-C-C | (3,21)(10,25)(20,29) |
| 1g1z | *Conus ermineus* | C3-C10-C20-C21-C25-C29 | C-C-CC-C-C | (3,21)(10,25)(20,29) |
