# Supplementary material for: Cysteine pattern barcoding-based dataset filtration enhances the machine learning-assisted interpretation of Conus venom peptide therapeutics
Source: PLoS One. 2025 Jul 11;20(7):e0327578. doi: 10.1371/journal.pone.0327578 (PMC12250603; doi:10.1371/journal.pone.0327578)
Supplement: S4 Table — (DOCX) [file pone.0327578.s005.docx]

**Table** **S4. Predictions results of RF model on test dataset**.

| **Sequence** | **Prediction** |
| --- | --- |
| AANAKLFAVRQSCCSTPPCALLYMEMCG | 0 |
| AANAKLFDVRQSCCSTPPCALLYMEMCG | 0 |
| GCCARAACAGIHQELCGX | 0 |
| GCCSHPACSGNNPYFCX | 0 |
| KCCSNPACNRYNKLC | 1 |
| MTREMWDECCEDPPCRNNHLEHCPA | 1 |
| TSNQITRGVWDECCKDPQCRQNHMQHCPA | 1 |
| AANAKLFDVRQSCCSAPLCALLYRVMCG | 0 |
| AAKVKYSKTPEECCPNPPCFATHSEICGGRR | 1 |
| GCCSHPACAGNNPHICS | 1 |
| MKLSVMFIVFLMLTMPMTCAGISRSATNGGEADVRAHDKAANLMALLQERMRPPLCKPSCTNCG | 0 |
| AADSNAADQIAQTVRDECCSNPSCAQTHPEVCRRRR | 1 |
| MGMRMMSTVFLLVVLATAVVSFTLDRASDGGNAAAKAFDLMALTARLDCCDIHACWSAHPELCGGRR | 0 |
| AANAKLSERLDPCCREPPCASTHIDRCG | 1 |
| AAKFKAPALMELTVREGCCSDPRCSGKHQDLCS | 1 |
| AANDKASVQIALTVQECCADSACSLTNPLICGRR | 0 |
| DCCPAKMFCCQW | 0 |
| DCCPAKLLCCNP | 0 |
| ATNNKATDLMALTVRGCCDDPSCRAENPFLCSWMG | 1 |
| AANNKATDLMALTVRGCCDDPPCRARYPFLCI | 0 |
| AANNKATDLMALTVRGCCADPSCSILMPYFCI | 0 |
| AANDKASVQIALTVQECCADAACSLTNPLICGRR | 0 |
| MGMRMMFIMFMLVVLATTVDTFTSDHALDAMNAAASNKASRLIALAVRGCCARAACAGIHQELCGGRR | 0 |
| AADSKVAARIAQIDRDPCCSYPACGANHPEICGGKR | 1 |
| MGMRMMFIMFMLVVLATTVDTFTSDRALDAMNAAASNKASRLIALAVRGCCARAACAGIHQELCGGRR | 0 |
| AADSKVATRIAQIDRDPCCSYPACGANHPEICGGKR | 1 |
| DCCPANLLCCNP | 0 |
| SCCPREFLCCL | 0 |
| AGLTDADLKTEKGFLSGLLNVAGSVCCKVDTSCC | 0 |
| AGLTDADLKTEKGFLSGLLNVAGSVCCKVDTSCCSNQ | 1 |
| QCCRPANMSCCQ | 1 |
| KVYCCLGVRDDWCCAGQIQI | 0 |
| AGLTDADLKTEKGFLSGLLNVAGSVCCKVDTSCCSSQ | 0 |
| AGLTDADLKTEKGFLSGLLNVADSVCCKVDTSCCSN | 0 |
| GCCARAACAGIHQELCX | 0 |
| AANNKATDLMALTVRGCCGNPSCSIHIPYVCN | 0 |
| TANNKATDLMALTVRGCCGNPSCSIHIPYVCN | 0 |
| DCCPSKLLCCNP | 0 |
| AGLTDADLKTEKGFLSGLLNVAGSVCCKVDTSCCSN | 0 |
| SCCPQEFLCCLYLV | 0 |
| PCCSIHDNSCCX | 0 |
| MGMRMMFTMFLLVVLAIAVVSFTSDRASDGRNDAAKAFHRIALTARTGCCEFPYCAENIPELCGGRR | 0 |
| AAKVKYSKTPEECCPNPPCFATNSDICGGRR | 1 |
| MGMRMMFTVFLLIVLATTVDSFTSDRASDGGNAAAKAFDLMALTARLDCCDIHACWMYCR | 0 |
| MGMRMMFTMFLLVVLSTTVVSLPVDHASNGRDAAADSNAADQIAQTARDPCCSNPSCAQTHPEICRRMLQNPLNHDMSPSA | 0 |
| MKVVLVLLAVLVAASAAPQKRFFLHDIQNWIHQLQNAINKAKDKFNELTSGLGVHFDRIVDLLVDQIDSTMTEAACIKLCEGSASKILGQASSMAGTVCAPVCTAALAKLEEAAG | 0 |
| MKFSVMFILSLVLTLSIADGLIRPSKIGGRTLRRHNPDSMDLQTRQIKTKNLCPHCPDGCHMDLTCID | 0 |
| AAKVKYSNTREECCPNPPCFATHSEICGGRR | 1 |
| MGMRMMFTMFLLVVLTTTVVSFNSDRESNHENRRTSNQITRGVWDECCKDPQCRQNHMQHCPAR | 1 |
| VPAEPILEQLCPEMCNRGELEFFCTCGSRQFVVTLPVIE | 0 |
| VPAEQMMEELCPDMCNRGEGEIICTCVLRRHVVSPSIR | 1 |
| VPAEPILEEICPDMCNSGEGEIFCTCGSRQFVVTLPVIE | 0 |
| SDGRNDAANDKASKLMVLRNECCDNPPCKSSNPDLCDWRS | 1 |
| AANNKATDLMALTVRGCCAHLPCALMYTACSWGR | 0 |
| QIKTKNLCPHCPDGCHMDLTCID | 0 |
| MGMRMMFTMFLLVVLTTTVISFNSDRDSNHVNRRASKRMTREMWDECCEDPPCRNNHLEHCPAR | 1 |
| IVDLLVDQIDSTMTEAACIKLCEGSASKILGQASSMAGTVCAPVCTAALAKLEEAA | 0 |
| WWEKQLGSWSTNCEWDSGCSGGCNGHYCTTW | 1 |
| AAKFKAPALMKRTVSQECCLDTRCAGKNLDECG | 1 |
| ECCDDPPCRQNNMEHCPAS | 1 |
| MGMRMMFTVFLLVVLATTVVSLTLDRASGGRRSGADNMIALLIIRKCCSNPACNRYNKLC | 0 |
| FDGRNAAPSDKASDLISLAVRGCCSHPACSGNNPYFCGGKR | 1 |
| ECCEDGWCCTAAPLTX | 0 |
| SDGRNDAASNKASHLIALAVRGCCARAACAGIHQELCGGGR | 1 |
| KVPCCLGVRDDWCCAGQIQI | 0 |
| DCCPRSILCCEFS | 0 |
| MVKYNAKRGTPTLSKRTSDPGRWWEKQLGSWSTNCEWDSGCSGGCNGHYCTTWR | 1 |
| ACYPPCFGSSVCYGGRCFFIGF | 0 |
| DECCSNPSCAQTHPEVC | 1 |
| SDDNDVAAEMMSGLIALAIDSCCSDSDCNANHPDMCS | 0 |
| MGMRMMFIVFLLVVLATTVVSFTLDHVLGLASEGRNAKAIDNALDQRDPKRQTPGCCWHPACGKNRCGRR | 0 |
| MFTVFLLVVLATTVGSFTLDRASDGRDAAANDKASDLIALTARRDPCCSNPACNVNNPQICG | 0 |
| GCCSDPRCNMNNPDYCX | 1 |
| MFTVFLLVVLATTVVSFTSDRASDSRKDAASGLIALTIKGCCSDPRCNMNNPDYCG | 0 |
| MGMRMMFIVFLLVVFASSVTLDRPSHGRYAAVVNRASALMAHAALRDCCSDPPCAHNNPDCR | 0 |
| MGMRMMFIVFLLVVFASSVTLDRASHGRYAAVVNRASALMAQAVLRDCCSNPPCAHNIHCA | 0 |
| MGMRMMFIVFLLVVFASSVTLDRASRGRYIPVVDRASALMAQADLRGCCSNPPCARLNPACG | 0 |
| MGMRMMFIVFLLVVFASSVTLDRAYHGRYAPVVDRASALKAQAVLRGCCSDPPCAHKHCR | 0 |
| MGMRMMFIVFLLVVLAPSVTLDRAYHDRYASVVDRASALTQAVLRGCCSDPPCAHKHCR | 0 |
| MGMRMMFIVFLLVVFASSVTLDRPYHGRYTPVVDRASALKAQAVLRGCCSDPPCAHKHCR | 0 |
| MGMRMMFIVFLLVVFASSVTLDRPYHGRYAPVVDRASALEAQAVLRGCCSDPPCAHKHCR | 0 |
| MGMRMMFTVFLLVVLATAVVSFTLDRPSDGGDAAAKAFDLMALTARLDCCDIHACWSAHPELCGGRR | 0 |
| LTQFECCSEVFSCC | 0 |
| YAAVVNRASALMAHAALRDCCSDPPCAHNNPDC | 1 |
| LPVFIILLLLVSPAATLPVESELERDLTQESPKDFGMRTEHFPLMIWVDCCPAYDCCVPDSD | 0 |
| YAAVVNRASALMAQAVLRDCCSNPPCAHNIHCA | 0 |
| GCCSDPPCAHKHC | 1 |
| LDCCDIHACWSAHPELCG | 0 |
| AALEDADMKTAKGILSNIMGNLGNIGNMAGSFCCSVYSGCCPE | 0 |
| TALEDADMKTEKGVLSGIMSNLGTVGNMVGGFCCTVYSGCCAE | 0 |
| KVPCCLGVRDDWCCAGQIQI | 0 |
| TLQRHWAKSLCCPEDAWCCSHDEX | 0 |
| GCCEDKTCCFI | 1 |
| TLQRHWAKFLCCPEDDWCC | 1 |
| FLGLIGPITSIAGKLCCTVSVSFCCNE | 0 |
| LCPPMCRSCSNC | 1 |
| DLCPHCPNGCHVDRTCI | 1 |
| ACYPPCFGSSVCYGGRCFFIGF | 0 |
| VPAEQMMEELCPDMCNRGEGEIICTCVLGRHVVSPSIR | 0 |
| ALAEPILEEICPDMCNSGEGEIFCACGSRQFVVTLPVIE | 0 |
| GCCSNPPCARLNPAC | 1 |
| MFTVFLLVVLATTVGSFTLDRASDGRDAAANDKASDLIALTARRDPCCYHPTCNMSNPQICG | 0 |
| MFIVFLLVVLATTVGSFTLDRVLEGRNAAAIDNALDQRDPKRQTPGCCWNPACVKNRCGRR | 1 |
| MFIVFLLVVLATTVGSFTLDRVLEGRNAKAIDNALDQRDPKRQTPGCCWNPACVKNRCGRR | 1 |
| HTLEMLLLLLLLSPLALGEGDGQAVAGDRNPSEARSTHEHFPQKLLGRIDGRDCQPCGHDVCCPP | 1 |
| MHTLEMLLLLLLLSPLALGEGDGQAVAGDRNPSEARSTHEHFLQRLLGRIDGRDCQPCGHDVCCPP | 1 |
| MGMRMMLTVFLLVVLATTVVSDHASDGRNTAAKVKYSKTQEECCPNPPCFAVNSALCGARR | 0 |
| MGMRMMFTVFLLVVLSTTVVSLPVDHASNGRDAAADSNAADQIAQTARDPCCSNPSCAQTHPEICRRMLQNPLNHDMSPSA | 0 |
| MGMRMMFTVFLLVALATTVASFTLDHASNGRNAAADDNMSDWIAQALKRCCGNPPCAHVNCRGRR | 0 |
| MGMRMMFTMLLLVVLATTVVSFTLDHAFDGRNTAANNKATDLMALPVRGCCSDPPCRHKHQDLCG | 0 |
| MGMRMMFTMLLLVVLATTVVSFTLDHAFDGRNIAANNKATDLMALPVRGCCSDPPCRHKHQDLCG | 0 |
| MGMRMMFTMFVLVVLATTVVSFPLDHASNGRDAAAYDKATPLIARDMEECCSHPACQGINPDYCD | 0 |
| MGMRMIFTVFLLVALATTVASFTLDRASNGRNAAADNKPSDWIALAIKQCCRNPKCSQQGCP | 0 |
| LIHGSDCQPCGQYVCCPPWKYAEY | 1 |
| DCKLCGHDVCCDWA | 0 |
| DCQPCGHDVCCPP | 1 |
| NTAAKVKYSKTQEECCPNPPCFAVNSALCGA | 1 |
| DPCCSNPSCAQTHPEIC | 1 |
| CCGNPPCAHVNCR | 1 |
| GCCSDPPCRHKHQDLC | 1 |
| DMEECCSHPACQGINPDYCD | 1 |
| CHLPPETGMCRAYIPMHFYNATLGRCQGFIYGGCNGNDN | 1 |
| DPCCSNPACNVNNPQIC | 1 |
| QTPGCCWHPACGKNRC | 1 |
| DDCCPDPSCRQNHPELCA | 1 |
| DCCPEKMWCCPL | 0 |
| TEHFPLMIWVDCCPAYDCCVPDSD | 0 |
| VPAEPILEEICPDMCNSGEGEIFCTCGSRQFVVTLPVIE | 0 |
| GPCCFSNPYCCNL | 1 |
| QTPGCCWNPACVKNRCX | 0 |
| ZTOGCCWHPACGKNRCX | 0 |
| SPGSTICKMACRTGNGHKYPFCNCRX | 0 |
| SSGSTVCKMMCRLGYGHLYPSCGCRX | 0 |
| DCCWPGRPDCCAP | 1 |
| QCCRNPKCSQQGCP | 1 |
| RDPCCYHPTCNMSNPQICX | 0 |
| MHTLEMLLLLLLLSPLALGEGDGQAVAGDRNPSEARSTHEHFLQKLLGRIDGRDCQPCGHDVCCPP | 1 |
| GCCSNPACMLKNPNLCX | 0 |
| MGMRMMFTVFLLVVLATTVVSFTSDRAPDGRNAAAKAFGLITPTVRKGCCSNPACMLKNPNLC | 0 |
| AAKVKYSLTPAECCPNPPCFAQHSDLCGA | 0 |
| AANAKLFAVMQSCCSTPPCALRHMDMC | 0 |
| AADGKPSDWISLVTPECCSNPPCNLQNC | 0 |
| DECCSNPPCAQAHPEVC | 1 |
| SDWFAWVIHSCCSNPPCAHVNC | 0 |
| AANSKVAARIAQIDRDPCCSYPACGANHPEICGGKR | 1 |
| AANDKASVQIALTVQECCADAACSLTNPLICGRR | 0 |
| AANDKASVQIALTVQECCADSACSLTNPLICGRR | 0 |
| AAKFKAPALMELTVRDECCSDPRCAVKHQDLCG | 1 |
| AAKFKAPALMERNVWEKCCLDPRCSGKHQNKCG | 1 |
| AAKFKAPALMKRTVREACCSDPRCSGQHQEKCG | 1 |
| AGNDKATDLMALTVRGCCSHPACAGNNPHICG | 0 |
| AANNKATDLMALRYHECCKHPPCRNTRPDLCGGRR | 1 |
| AAKVKYSLTPAECCTNPPCFAQHSDLCGARR | 1 |
| AAKVKYSLTPAECCPNPPCFAQHSNLCGA | 0 |
| AANNKATDLMALTVRGCCDDPSCSIHMPFFCF | 0 |
| AANNKATDLMALTVRGCCGNPSCSIHIPYVCN | 0 |
| AANAKLSERLDPCCRDPPCASTHTDICTRRR | 1 |
| AAKVKYSLTPAECCPNPPCFAQHSNLCGARR | 1 |
| AAKVKYSLTPAECCPNPPCFAQHSDLCGARR | 1 |
| AANAKLFAVMQSCCSTPPCALRHMDMCG | 0 |
| AADGKPSDWISLVTPECCSNPPCNLQNCR | 1 |
| AADSNAADQIAQTVRDECCSNPPCAQAHPEVCRRRR | 1 |
| AADYKRSDWFAWVIHSCCSNPPCAHVNCRRRR | 1 |
| KAVCCEVLPGTDCCF | 0 |
| HECCSYPACAYTHPELCG | 1 |
| DDCCLVASCWQRYPERCGG | 1 |
| TGCCEFPYCAENIPELCG | 0 |
| TGCCEYPYCAENNPELCG | 1 |
| IALIATRECCANPQCWSKNC | 1 |
| AANNKATDLMALTVRGCCADPSCSILMPYFCI | 0 |
| LDPCCRDPPCASTHTDICT | 1 |
| GCCGNPSCSIHIPYVCN | 0 |
| GCCADPSCSILMPYFCI | 0 |
| MGMRMMFTVFLLVVLATAVLPVTLDRASDGRNAAANAKTARLIAPFIRDYCCPRGPCMVWCG | 0 |
| MGMRMMFTMFLLVVLAIAVVSFTSDRASDGRNDAAKAFHRIALTARTGCCEFPYCAENIPELCGGRR | 0 |
| MGMRMMFTVFLLVVLATAVVSFSSDRASHGRNAAAKLFDRIALIATRECCANPQCWAKNCR | 0 |
| MGMRMMFTLFLLAVLSTTVVSFTLDRASNGRDAAADSKAADQIAQTVRDECCSNPSCAQTHPEICRRTLMLQNPLNHDMSPSA | 1 |
| MGMRMMFTMFLLVVLATTVVSFTSDHASDGRNTAANDKASDLMALRDGCCSSPSCSVNNPDICGGGR | 0 |
| NLQILCCKHTPACCT | 0 |
| NLQILCCKHTPKCCT | 0 |
| NLQILCCKHTPACC | 0 |
| NLQILCCKHSLECCT | 0 |
| VCCRPMQDCCS | 1 |
| IALIATRECCADPQCWAKNC | 0 |
| TGCCEFPYCAENIPELCG | 0 |
| IALIATRECCANPQCWAKNC | 1 |
| DECCSNPSCAQTHPEIC | 1 |
| DGCCSSPSCSVNNPDICGG | 0 |
| MFTVFLLVVFASSVTLDRASYGRYASPVDRASALIAQAILRDCCSNPPCAHNNPDCR | 0 |
| MFTVFLLVVLATTVVSFTSDRAFNYKNYRTSKRKVSEELNECCRIPPCLLDHADECES | 1 |
| GCCDDPSCSIHMPFFCF | 0 |
| AAKVKYSLTPAECCTNPPCFAQHSDLCGA | 0 |
| YHECCKHPPCRNTRPDLCG | 1 |
| GCCSHPACAGNNPHIC | 1 |
| TVREACCSDPRCSGQHQEKC | 1 |
| NVWEKCCLDPRCSGKHQNKC | 1 |
| LIAPFIRDYCCPRGPCMVWCX | 0 |
| DECCSDPRCAVKHQDLC | 1 |
| AANDKASVQIALTVQECCADAACSLTNPLIC | 0 |
| DPCCSYPACGANHPEICG | 1 |
| LTSEELIECCVIESCHSNHIDECES | 0 |
| KVSEELNECCRIPPCLLDHADECES | 0 |
| ASALIAQAILRDCCSNPPCAHNNPDC | 0 |
| MFTVFLLVVLATTVVSFTSDHAFYHKNRRTSKRLTSEELIECCVIESCHSNHIDECES | 0 |
| AANDKASVQIALTVQECCADSACSLTNPLIC | 0 |
| SGCCSNPACRVNNPNICX | 0 |
| MHTLEMLLLLLLLLPLAPGEGDGQAVAGDRNPSEARSTHEHFLQKLLGRIDGRDCQPCGHDVCCPP | 1 |
| EMLLLLLLLLPLAPGEGDGQAVAGDRNPSEARSTHEHFLQRLIRLIDGRDCKLCGHDVCCDWA | 0 |
| ESDGAHAKARADKPARSATNRQPGCCNNPACVKHRCG | 1 |
| GCCSHPPCNVNNPHICGX | 0 |
| EGCCSNPPCRHNHPEVCX | 0 |
| EGCCSNPPCRHNHOEVCX | 0 |
| MGMRMMFTVFLLVVLATAVLPVTLDRASDGRNAAANAKTPRLIAPFIRDYCCHRGPCMVWCG | 0 |
| IVRRACCSDRRCRWRCG | 1 |
| IVRRGCCSDPRCAWRCG | 1 |
| MGMRMMFTVFLLVVLATTVVSDRASNRENRRASNWNTRIAYIGCCDIPDCYNKNREQCLDESSG | 1 |
| MGMRMMFTVFLLVVLATAVLPVTLDRASDGRNAAANAKTARLIAPFIRDYCCPRGPCMVWCG | 0 |
| MGMRMMFTVFLLVVLATAVLPVTLDRASDGRNAAANAKTPRLIAPFIRDYCCHRGPCMVWCG | 0 |
| YCCHRGPCMVWCX | 0 |
| ACCSDRRCRWRCX | 0 |
| GCCSDPRCAWRCX | 0 |
| DWNGCCVKKAGCCPW | 0 |
| ESDGAHAKARADKPARSAMNRPPGCCNNPACGKNRC | 1 |
| CCIKFHPCCHN | 0 |
| TLQRLSIKYSCCPGIVSCCVIP | 0 |
| TLQRLSIEYSCCPGIVSCCVIP | 0 |
| ILQRYLDHPWCCAVKYSCCVPGIIPI | 0 |
| SCCGKNPGCCPWX | 0 |
| ADDTSGDDCVDTNEDCVNWASTGQCEANPSYMRENC | 1 |
| APAELILETICPHMCGIGIGEPFCNCRNKRDVVSSRII | 0 |
| ASNWNTRIAYIGCCDIPDCYNKNREQCLDESS | 1 |
| LIAPFIRDYCCPRGPCMVWCX | 0 |
| DYCCHRGPCMVWC | 1 |
| VCCRPVQDCCSX | 0 |
| LCCVTEDWCCEWW | 0 |
| MRYLPVFVILLLLIASIPSDTVQLKTKDDMPLASFHGNGRRILRMLSNKRLCCVTEDWCCEWW | 0 |
| MGMRMMFTVFLLVVLATTVVSFPSERASDGRDDTAKDEGSDMDKLVEKKECCHPACGKHFSCGR | 1 |
| MFTVFLLVVLATTVVSFPSERASDGRDDTAKDEGSDMDKLVEKKECCHPACGKHFSCGR | 1 |
| LPGLTSSGDSDESLPFLNTICCWSGACCG | 0 |
| GIVEDCCYNDCTDEKLKEYCHTLQ | 1 |
| VIVGDCCDNYCTDERLKGYCASLLGL | 0 |
| PGCCNNPACVKHRCG | 1 |
| MGMRMMFTMFLLVVLTTTVVSFNSDRESNHENRRTSNQITRGVWDECCKDPQCRQNHMQHCPAR | 1 |
| MGMRMMFTMFLLVVLTTTVVSFNSDRESNHENRRTSNQITRGMWDECCDDPPCRQNNMEHCPAS | 1 |
| VVLGPASDGRNAAANNKASDLIRQICCGYGDCGFVPNVCV | 0 |
| VVLGPASDGRNAPANNKVSDLIRQFCCGHYDCDFIPNVCG | 0 |
| MGMRMMFIIFLFVVLATTVVSFTSGRASDGRNAPANNKVSDLIRQFCCGHYDCDFIPNVCG | 0 |
| MGMRMMFTVFLLVILASTVTLDRASNGMNAAAIRKASALVAQIAYRDCCDDPACTVNNPGLCT | 0 |
| MGMRMMSIMFMLVVLATTVVSFTSDRALDAMNAAASKKASRLIALAVRGCCSHPACSGNHQELCDGRR | 0 |
| MGMRMMFIMFMLVVLATTVVSFTSDRALDAMNAAASKKASRLIALAVRGCCSHPACSGNHQELCDGRR | 0 |
| MGMRMMFIMFMLVVLATTVVSFTSDRASDGRNAEAKALDLIAATARPRCCSNPACGAGHPEICAGRR | 0 |
| MGMRMMFTVFLLVVLATTVVSFTSDRAFDGRNAAASDKASDLISLAVRGCCSHPACSVNNPYFCGGKR | 0 |
| LTLDRASDDTDVAAEIMSGLIALAIDSCCSDSDCNANHPDMCS | 0 |
| VVLGPASDGRNAAANVKAPDLIALTVRNDCCHNAPCRNNHPGICG | 0 |
| MGMRMMFIMFMLVVLATTVVTFTSDRALDAMNAAASNKASRLIALAVRGCCARAACAGIHQELCGGGR | 0 |
| MGMRMMFIMFMLVVLATTVDTFTSDHALDAMNAAASNKASRLIALAVRGCCARAACAGIHQELCGGRR | 0 |
| PCCPLIPGCC | 0 |
| CCPNLFYCCPD | 0 |
| GSVCCKVDTSCCSN | 1 |
| PGCCNNPACGKNRC | 1 |
| GCCARAACAGIHQELCX | 0 |
| NDCCHNAPCRNNHPGICX | 0 |
| SCCSDSDCNANHPDMCS | 1 |
| GCCSHPACSVNNPYFCGX | 0 |
| PRCCSNPACGAGHPEICA | 0 |
| MGMRMMFTVFLLVVLAATIVSFTSDRASDGRNVAAKAFHRIGRTIRDECCSNPACRVNNPHVCRRR | 0 |
| GCCSHPACSGNHQELCDX | 0 |
| QFCCGHYDCDFIPNVCX | 0 |
| QICCGYGDCGFVPNVCV | 0 |
| GMWDECCDDPPCRQNNMEHCPAS | 1 |
| GVWDECCKDPQCRQNHMQHCPAR | 1 |
| CCSDSDCNANHPDMCS | 1 |
| SCCPQEFLCCLYLVK | 0 |
| DCCDDPACTVNNPGLCTX | 0 |
| MHTLEMLLLLLLLLPLAPGEGDGQAVARDRNPSEARSTHEHFLQRLIRLIDGRDCKLCGHDVCCDWA | 1 |
| SDGRNDAAKAFDLISSTVKKGCCSHPACAGNNQHICGRRR | 1 |
| MGMRMMFTVFLLVVLATTVVSFPSERASDGRDDTAKDEGSDMEKLVEKKECCNPACGRHYSCKGGR | 1 |
| GVVEHCCHRPCSNAEFKKYCX | 0 |
| GVVEHCCHRPCSNAEFKKYC | 1 |
| TCRSSGRYCRSPYDRRRRYCRRITDACV | 1 |
| TCRSSGRYCRSPYDRRRRYCRRITDACV | 1 |
| TCRSSGRYCRSPYDRRRRYCRRITDACV | 1 |
| RILEDIVSTALATCCKFQFLNFCCNEK | 0 |
| TCCKFQFLNFCCNE | 1 |
| KCCPESPPCCHYF | 1 |
| CCPRRLACCII | 0 |
| GCCPNHSSCC | 1 |
| DCCQRNFLCC | 1 |
| VECPIYYCPDSVCC | 0 |
| ASSACPEPCAKGSTCIGKTC | 0 |
| ASRACNPPCTGLSMCQAGRCGYIRF | 0 |
| GIVEVCCDNPCTVATLRTFCH | 0 |
| QCPAPCYPNCEEC | 1 |
| IPCCSYPACAQSNIDLCG | 0 |
| KDAADLSALNDNNNCCNHPACAGKNSDLC | 1 |
| LVLIASAPGVDARPQTKDDALASFHDSAKRHLQRLVNARKCCPESPPCCHYFGRRK | 1 |
| IIVLLLVPSAHGIDAEAKTKSDESHASLHDKAKGILQRLSSSRGCCPNHSSCC | 1 |
| MSKLGVALLIFIVLFPLANLQLDGDQPADRHAEERRGLLHELYKLLRRSQTRVKEPEELRVECPIYYCPDSVCCKK | 0 |
| MKLSVMFIVFLMLTMPMTGDGNNRRAANGGEVGMPAYERAAKLLALLRERQCPAPCYPNCEEC | 0 |
| VFLLVALATTVVPFTSDRASASRNAATDNKASELKALNARIPCCSYPACAQSNIDLCGGRR | 0 |
| MGMRMMFTVFLLVVLAINVVSVTSYRASHGRKDAADLSALNDNNNCCNHPACAGKNSDLCG | 0 |
| CCHPACGRKYNC | 1 |
| GGCCSHPVCYVNYPQICR | 0 |
| ASDGRDDEAKDERSDMYESDRNGRCCHPACGRKYNCGR | 1 |
| ASDGRNAAADDKASDLVALAVRGGCCSHPVCYVNYPQICRGRR | 1 |
| MHTLEMLLLLLLLLPLAPGEGDGQAVAGDRNPSEARSTHEHFLQRLIRLIHGSDCQPCGQYVCCPPWKYAEYRRFT | 0 |
| MHTLEMLLLLLLLLPLALGEGDGQAVAGDRNPSEARSTHEHFLQRLIRLIHGSDCQPCGQYVCCPPWKYAEYRRFT | 0 |
| DDCCPNPSCRQNHPERC | 1 |
| GIVEVCCDNPCTVATLRTFCH | 0 |
| GCCSHPACSGNNPEYCRQ | 1 |
| NAAAKAFDLKGCCSHPACSGNYQEYCRESY | 1 |
| MFTVFLLVVLATTVVSFPSERASDGRDDTAKDEGSDMEKLVEKKECCNPACGRHYSCGR | 1 |
| MGMRMMFTVFLLVVLATTVVSFPSERASDGRDDTAKDEGSDMDKLVEKKECCNPACGRHYSCGR | 1 |
| MLSLPVFIILLLLASPAAPIPLETRIQSDLIRAALEDADMKNEKGLLNGLVGNLGEIGEIISTVCCSVYPKCCVEK | 0 |
| MGMRMMFTVFLLVVLATTVVSFTSRRGPKSRRGEPVPTTVINYGECCKDPSCWVKVKDFQCPGASPPN | 0 |
| MGMRMMFTVFLLVVLATTVVSSTSDRASDGRNAAASDKASDLITQVVKRCCGKPNDCLPSFAFVNGSCNGRR | 0 |
| MGMRMMFTVFLFLVLATTDVSFTLDRASDGGNAAAKKSAVIALSRRRCCGIKSCYENNKDMCGRRR | 0 |
| MGMRIMFTVFLLVALATIVVSFTSDRASDGRNAAAKAFDLKGCCSHPACSGNYQEYCRESY | 0 |
| MGMRMMFTVFLLVALATTVVSFTSDRASDRRNAAVKAFDLISSTVKKGCCSHPACSGNNPEYCRQGR | 1 |
| KFLSGGFKEIVCHRYCAKGIAKEFCNCPDX | 0 |
| ECCHPACGKHFSCX | 0 |
| IRDECCSNPACRVNNPHVC | 1 |
| GCCSHPACAGNNQHICX | 0 |
| ECCNPACGRHYSCKG | 1 |
| ECCNPACGRHYSCGK | 1 |
| ECCNPACGRHYSCX | 0 |
| AALEDADMKNEKGLLNGLVGNLGEIGEIISTVCCSVYPKCCVE | 0 |
| ADVDIDMAVFLEKLQDACCKNAPEFGCCT | 0 |
| RCCGIKSCYENNKDMC | 1 |
| EECCPNPPCFATHSEICG | 0 |
| CCGKPNDCLPSFAFVNGSCN | 0 |
| GEPVPTTVINYGECCKDPSCWVKVKDFQCPGASPPN | 1 |
| SFRFIPGGIKEIACHRYCAKGIASAFCNCPDKRDVVSPRI | 0 |
| SFRFIPGGIKEIACHRYCAKGIASAFCICPDKRDVVSPRI | 0 |
| MFTVFLLVVLATTVVSFPSERASDGRDDTAKDEGSDMEKLVEKKECCNPACGRHYSCKGGR | 1 |
| EEICRYMCSRKIDYHMCTCPSKRDAISSRIV | 1 |
| GCPPMCNPGCHNCS | 0 |
| QGWCCKENIACCV | 0 |
| QGWCCKENIACCI | 0 |
| AALEDADIKTEKGFLGSVFSNLGGITDLATGICCAIIERCCV | 0 |
| DCCEERWCCF | 1 |
| ICCAITDSCC | 0 |
| SFKFLSGGFKEIVCHRYCAKGIAKEFCNCPDKRDVVSSRIR | 1 |
| TVSQECCLDTRCAGKNLDEC | 1 |
| NAAANDKASGVNGCCSNPACHVDHAELC | 1 |
| EGCCSDPRCSGKHQDLCS | 1 |
| GILELAKTVCCSATGISICC | 0 |
| ILQRQCCPTISECCRV | 0 |
| TVRGHCCPYYPQCCPS | 1 |
| ASALISQAILRDCCSNPPCAHNNPDC | 1 |
| ASAPISQAILRDCCSNPPCAHNNPDC | 1 |
| MGMRMMFIVFLLVVFASSVTLDRASHGRYAAVVDRASALISQAILRDCCSNPPCAHNNPDCR | 0 |
| MGMRMMFIAFLLVVFASSVTLDRASHGRYAAVVDRASAPISQAILRDCCSNPPCAHNNPDCR | 0 |
| MFTVFLLVVLATTVVSFNSDRALGGRNAAAKASDKIASILGRRACCSYPPCNVNYPEICGGRG | 0 |
| MFTVFLLVVLATTVVSFNSDRALGGRNAAAKASDKIASILGRRRCCSYPPCNVSYPEICGGRR | 0 |
| MFTVFLLVVLATTVVPFNSDRDPALGGRNAAAIASDKIASTLRRGGCCSYPPCNVSYPEICGGRR | 0 |
| MFTVFLLVLLATTVVSFNSDRALGGRNAAAKASDKILSNLRRGGCCFHPVCYINLLEMCRQRG | 0 |
| MFTVFLLVVLATTVVSFNSDRDPALGGRNAAAIASDKIASTLRRGGCCSFAACRKYRPEMCGGRR | 0 |
| MFTVFLLVVLATTVVSFTSDHESDRGDAQTIQEVFEMFALDSDGCCWHPACGRHYCGRRR | 0 |
| MFTVFLLVVLATTVVSFNSDRDPALGGRNAAAIASDKIASTLRRGGCCSFPACRKYRPEMCGGRR | 0 |
| GIFSTLCCGVGINSCC | 0 |
| MFTVFLLVVLATTVVSFNSDRDPALGGRNAAAKASDKIASTLKRRGCCSYFDCRMMFPEMCGWRG | 0 |
| CCSYPPCNVSYPEICG | 1 |
| GCCSYPPCNVSYPEICG | 1 |
| GCCFHPVCYINLLEMCRQR | 0 |
| GCCSFAACRKYRPEMCG | 1 |
| GDAQTIQEVFEMFALDSDGCCWHPACGRHYC | 0 |
| GCCSFPACRKYRPEMCG | 1 |
| RGCCSYFDCRMMFPEMCGWR | 1 |
| MGMRMMFTVFLLVVLATTVVSFPSDRASDGRDDEAKDERSDMHESDRKEICCNPACGPKYSCGR | 1 |
| MLKMGVVLFTFQVLFPLATLQLDADQPVERHAENKQDLIPDERRGILLHALKQRVWCDWEWCYGDCHCFD | 0 |
| MFTVFLLVVLATTVVSFTSDRASDGRDDEAKDERSDMHESDRKGRAYCCHPVCGKNFDCGR | 1 |
| SDGRDDEAKDKRSDMYGSDRSGRCCHPACGRKYNCGR | 1 |
| SDGRDDEAKDERSDMHESDRKGRAYCCHPACGKNFDCGR | 1 |
| MGMRMMFIVFLLVVLATTVVSFPSDRASDGRDDEAKDVDMHESDRKGRAYCCHPACGKNFDCGR | 1 |
| MGMRMMFTVFLLVVLAITVVSFPLDRESDGANAEARTHDHEKHALDRNGCCRNPACESHRCG | 1 |
| ACCSYPPCNVNYPEICGGR | 1 |
| CCPKKPYCCPG | 1 |
| VMQLRYYNWIDCCFDGACCN | 0 |
| GCCEDKTCCFIX | 0 |
| QTCCGSKVFCCX | 0 |
| CCPPVIWCCX | 0 |
| GIFALAKSVCCTAAKLSFCC | 0 |
| GILELAKTVCCSATGISICC | 0 |
| RDCCKGNPGCCGWD | 1 |
| CCQTFYWCCVQX | 0 |
| ECCSHPACNVDHPEICR | 1 |
| GCCSRPPCIANNPDICX | 0 |
| QTCCGYRMCVPCx | 1 |
| DPCCGYRMCVPCX | 1 |
| GCCSRPPCIANDPDLC | 1 |
| RPQCCSHPACNVDHPEIC | 1 |
| PECCSDPRCNSSHPELCGX | 0 |
| SGCCVIDSNCCX | 0 |
| QCCWYFDISCCITV | 0 |
| VNCCPIDESCCS | 0 |
| VCCRPMQDCCSX | 0 |
| SLCCPEDAWCC | 0 |
| DGCPPHPVPGMHPCMCTNTC | 0 |
| TLQRHWAKSLCCPEDAWCCSHDE | 1 |
| DCCDPKEPCCFI | 1 |
| GVLSGIMSNLGTVGNMVGGFCCTVYSGCCSE | 1 |
| FLEGVISTIKDFAGKVCCSVSVNFCCPTA | 1 |
| YCCHPACGKNFDCX | 0 |
| SVEGVISTIKDFAVKVCCSVSLKFCCPTA | 0 |
| NNPPCCSGYVCEGVYCAVDV | 1 |
| PNPLERRIQSDLIRAALEDADMKTDERSVEGVISTIKDFAVKVCCSVSLKFCCPTA | 0 |
| NCCRRQICCX | 0 |
| NCCRRQICCX | 0 |
| NCCRRQICCX | 0 |
| ICCYPNVWCCD | 0 |
| KAICCDVFPGTDCCM | 0 |
| ICCNPACGPKYSCX | 0 |
| NLQILCCKHTPACCT | 0 |
| ECCEDGWCCTAAPLT | 0 |
| AGNDKATDLMALTVRGCCSHPACAGNNPHICG | 0 |
| AASDKASELMALAVRGCCSHPACAGNNPHICGRRR | 1 |
| AANNKATDLMALTVRGCCDDPPCRARYPFLCI | 0 |
| AANNKATDLMALTVRGCCADPSCSILMPYFCI | 0 |
| AANNKATDLMALTVRGCCGNPSCSIHIPYVCN | 0 |
| AANAKLSERLDPCCREPPCASTHTDICTRRR | 1 |
| AANAKLSERLDPCCREPPCASTHIDRCG | 1 |
| AAKVKYSNTPEECCPNPPCFATHSEICGVRR | 1 |
| AAKVKYSKTPEECCPNPPCFATNSDICGGRR | 1 |
| DPCCSYPACGANHPEICG | 1 |
| QSCCSTPPCALLYMEMC | 0 |
| AANAKLFDVGQSCCSAPLCALLYMVIC | 0 |
| DECCSNPSCAQTHPEVC | 1 |
| AANDKASVQIALTVQECCADAACSLTNPLIC | 0 |
| AANDKAPVQIVLTVQECCADSACSLTNPLIC | 0 |
| TVRDACCSDPRCSGKHQDLC | 1 |
| GCCSHPACAGNNPHIC | 1 |
| GCCSDPRCKHECX | 0 |
| DYCCRRPPCTLICX | 0 |
| GCCSHPACNVNNPHICX | 0 |
| GCCSDPRCRYRCR | 1 |
| GCCSDPRCRYRCX | 1 |
| TWEECCKNPGCRNNHVDRCRGQV | 1 |
| AAKFKAPALMKRTVRDACCSDPRCSGKHQDLCG | 1 |
| AAKVKYSKTPEECCPNPPCFATNSDICG | 1 |
| LDPCCREPPCASTHIDRC | 1 |
| LDPCCREPPCASTHTDICT | 1 |
| GCCGNPSCSIHIPYVCN | 0 |
| GCCADPSCSILMPYFCI | 0 |
| GCCDDPPCRARYPFLCI | 1 |
| YHECCKNPPCRNKHPDLC | 1 |
| AAKVKYSNTPEECCPNPPCFATHSEICGV | 0 |
| NCCPIDEESCCS | 1 |
| AANDKAPVQIVLTVQECCADSACSLTNPLICGRR | 0 |
| AADINAADQIAQTVRDECCSNPSCAQTHPEVCRRRR | 1 |
| VCCRPMQDCCS | 1 |
| VWCDWEWCYGDCHCFD | 1 |
| QTCCGYRMCIPC | 1 |
| AYCCHPVCGKNFDC | 1 |
| CCHPACGRKYNC | 1 |
| AYCCHPACGKNFDC | 1 |
| NGCCRNPACESHRCX | 0 |
| ARPKDRPSYCNLPADSGSGTKPEQRIYYNSAKKQCVTFTYNGKGGNGNNFSRTNDCRQTCQYPVG | 1 |
| KDRPSLCDLPADSGSGTKAEKRIYYNSARKQCLRFDYTGQGGNENNFRRTYDCQRTCLYT | 1 |
| MFTVFLLVVLATTVVSSPSDRASDGRNAAANEKASDVIALALKGCCSNPVCHLEHSNMCGRRR | 0 |
| MFTVFLLVVLATTVVSFPSDRESDGANDEARTDEPEEHGPDRNGCCRNPACESHRCG | 1 |
| MFTVFLLVVLATTVVSFPSDRASDGRDDEAKDERSDMHESGRKGRGRCCHPACGPNYSCGR | 1 |
| IMYDCCSGSCSGYTGRCX | 0 |
| GCCSNPVCHLEHSNMCX | 0 |
| NGCCRNPACESHRC | 1 |
| GRCCHPACGPNYSCX | 0 |
| IINWCCLIFYQCC | 0 |
| AANAKLFDVGQSCCSAPLCALLYMVICG | 0 |
| AANAKLFAVRQSCCSTPPCALLYMEMCG | 0 |
| AADSKVAARIAQIDRDPCCSYPACGANHPEICGGKR | 1 |
| MSTVFLLVVLATTVVSFTVDRASDGRDVAIDDRLVSLPQIAHADCCSDPACKQTPGCR | 0 |
| MFTVFLLVVLATTVASFIIDDPSDGRNIAVDDRGLFSTLFHADCCENPACRHTQGC | 0 |
| LVSLPQIAHADCCSDPACKQTPGC | 0 |
| AANDKASVQIALTVQECCADAACSLTNPLICGRR | 0 |
| GLFSTLFHADCCENPACRHTQGC | 1 |
| IINWCCLVFYQCC | 0 |
| IINWCCLTFYQCC | 0 |
| IMAGCCPRFYQCCYPX | 0 |
| IINWCCLIFYQCCL | 0 |
| RTCCSRPTCRMEYPELCGX | 0 |
| RTCCSRPTCRMEYPELCGX | 0 |
| MGMRMMFTVFLLVVLATTVVSFTSDSAFDSRNVAANDKVSDMIALTARRTCCSRPTCRMEYPELCGGRR | 0 |
| GCCSDPRCKHQCX | 1 |
| NIQIICCKHTPKCCTX | 0 |
| KPCCSIHDNSCCGIX | 0 |
| MGMRMMFTVFLLVVLATTVVSFTSDRASDGRKAAASDLITLTIKGCCSDPPCIANNPDLCGRRR | 0 |
| MGMRMMFTVFLLVVLATTVVFFTSDRASDGRKAAASDLITLTIKGCCSNPACMVNNPQICGRRR | 0 |
| ICCYPNEWCCD | 1 |
| CCQTFYWCCGQX | 0 |
| CCPGKPCCRIX | 0 |
| GCCSDPRCNYDHPEICX | 0 |
| RNCCRLQICCGRT | 1 |
| TLQIFSNILECCYSDEWCCEVDEELGL | 0 |
| ECCEDGWCCTAAPLTAP | 0 |
| DNETVCCERGALCCFV | 1 |
| LINTRCCPAQPCCRM | 1 |
| VNCCGIDESCCS | 0 |
| QPCCSIHDISCCGL | 0 |
| RCCSIHDNSCCGL | 1 |
| MHLSLARSAVLMLLLLYALGNFVGVQPEQITRDVDNGQLTDNRRNLQSEWKPVSLFKSLYKRQPRFESCQQDSDCDFQFFCWNNECHRIILI | 1 |
| GICCSKHPPCCSQFQ | 1 |
| RCCPPVIWCC | 0 |
| RLCCITEEWCCQWW | 0 |
| FCCFPEHWCCEW | 0 |
| ICCPLMELCCEW | 0 |
| RQCCPTFSECCEMEEEFGL | 1 |
| RDCCLIDEWCCEVDEEFGY | 0 |
| QTCCGFKFFCC | 1 |
| DNKTVCCKRGTLCCFV | 1 |
| CCQAFYWCCY | 1 |
| LINTRCCPGQPCCRM | 1 |
| LINTRCCPGQPCCRI | 0 |
| QPRFESCQQDSDCDFQFFCWNNECHRIILI | 1 |
| VPPEPILEIICPGMCDEGVGKEPFCHCTKKRDAVSSRI | 0 |
| AFQDIFMVALCPVFCFSNVALESCNCEEILDLVSSLIS | 0 |
| RDCCTNLLPCCY | 1 |
| VILLLLTASAPSVDARPKTEDVPLSSFRDNTKSTLQRLLKRVNCCGIDESCCS | 0 |
| MGMRMMFTVFLLVVLATTVVSSTSGRREFRGRNAAAKASDLVSLTDKKRGCCSDPRCNYDHPEICG | 1 |
| VILLLITSTPSVDARLKAKDNMPLASFHDNAKRTLQTRLINTRCCPGKPCCRIG | 0 |
| FPRPRICNLACRTGIGYKYPFCHCR | 1 |
| FPRPRICNLACRAGIGYKYPFCHCR | 1 |
| GIRGNCCMFHTCPIDYSRFNCP | 1 |
| GIRGNCCMFHTCPIDYSRFYCP | 0 |
| TVIMHNCCTRSFCKRIYPDLCS | 0 |
| MGMRMMFTVFLLVVLATTVVSFTLDRASDGASAAADLVARGIRGNCCMFHTCPIDYSRFNCP | 0 |
| MGMRMMFTVFLLVVLATTVVSFTLDRASDGANAAADLVARGIRGNCCMFHTCPIDYSRFYCP | 0 |
| MGMRMMFTVFLLVVLATTVISFTSDRASDDGNSAVDLKARTVIMHNCCTRSFCKRIYPDLCS | 0 |
| QCCPTIPECCRVX | 0 |
| QCCITIPECCRIX | 0 |
| RDCCSNPPCAHNNPDCX | 0 |
| HCCPIDLQCCPP | 0 |
| HCCPIDLPCCPP | 0 |
| VPAEQMMEELCPDMCNRGEGEIICTCVLRRHVVFPSIR | 0 |
| QCCPTMPECCRIX | 0 |
| NAAAKAFGLITPTVRKGCCSNPACMLKNPNLC | 0 |
| ASALIAQAILRDCCSNPPCAHNNPDC | 0 |
| VILLLLIASAPSVDAQPKTKDDVPLAPLHDNAKSALQHLNQRCCQTFYWCCGQGK | 0 |
| VILLLLIASIPSDAVQLKTKDDMPLASFHGNARRTLQMLSNKRICCYPNEWCCD | 0 |
| MGFRVLVLVVMATTSALPFTFSEEPGRSPFRPALRSEEAQALRHGLTLLLARRADGQPPDMRQPEMRRPEMRRPEVRQPEFAETPVGQKRGGLGRCIYNCMNSGGGLSFIQCKTMCY | 1 |
| MGFRVLVLVVMATTSALPFTFSEEPGRSPFRPALRSEEAQALRHGLTLLLARRADGQPPDMRQPEMRRPEVRRPEVRQPEFAETPVGQKRGGLGRCIYNCMNSGGGLSFIQCKTMC | 1 |
| MGFRVLVLVVMATTSALPFTFSEEPGRSPFRPALRSEEAQALRHGLTLLLARRADGQPPDMRQPEMRRPEMRRPEVRQPEFAELSVGQRRWDVDQCMYYCLTGVVGYSYTECETMCT | 1 |
| MGFRVLVLVVMATTFALPFTFFEEPGRSPFRPALRSEEAQALRHGLTLLLARRADGQPPDMRQPEMRRPEMRRPEVRQPEFAELSVGQRRWDVDQCMYYCLTGVVGYSYTECETMCT | 1 |
| NFPDILGDILCPFFCLSELGLDLGGEFCDCSEIPDLGLLPDQ | 0 |
| MGFRVLVLVVMATTSALPFTFSEEPGRSPFRPALRSEEAQALRHGLTLLLARRADGQPPDMRQPEMRRPEVRQPEFAELSVGQRRWDVDQCMYYCLTGVVGYSYTECQTMCT | 1 |
| GGLGRCIYNCMNSGGGLSFIQCKTMCY | 1 |
| WDVDQCMYYCLTGVVGYSYTECETMCT | 1 |
| WDVDQCMYYCLTGVVGYSYTECQTMCT | 1 |
| MGMRMMFVVFLLVVFASSVTLDRASYGRYASPVDRASALIAQAILRDCCSNPPCAHNNPDCR | 0 |
| MGMRMMFTVFLLVVLATTVVSFTSDRAPDGRNAAAKAFGLITPTVRKGCCSNPACMLKNPNLC | 0 |
| MGMRMMFVVFLLVVFASSVTLDRASYGRYASPPVDRASALIAQAILRDCCSNPPCAHNNPDCR | 0 |
| MGFRVLVLVVMATTSALPFTFFEEPGRSPFRPALRSEEAQALRHGLTLLLARRADGQPPDMRQPEMRRPEVRQLEFAELSVGQRRWDVDQCMYYCLTGVVGYSYTECQTMCT | 1 |
| YIPDDDESGVCYFLCLMGIDLDECSCPEIQDLGLFTDQ | 0 |
| QTCCGRTMCVPC | 0 |
| GCCSDPPCIANNPDLC | 0 |
| GVVEHCCKRACSNAEFMQFC | 0 |
| GVVEHCCYRPCSNAEFKKFC | 1 |
| GVVEHCCHRPCSNAEFKKFC | 1 |
| GVVEHCCHRPCSNAEFKKFCG | 1 |
| MKIYLCLAFLLLLASTIVDSGLLDKIETIRNWKRDDSYCDGCRCTILKKRFAHRLPAAGAHAEKSGHVGKKTATVLKSKVDIA | 1 |
| MGMRMMFTVFLFVVLATTGVSFTLDRASDGWNAAAKESDVIALTRRKCCSIPKCYKNNKKMCG | 0 |
| MGMRMMFTVFLLVVLTTTVVSFPSDIATEGRNAAAKAFDLISSTVRKGCCSNPACAGNNPHVCRQGR | 0 |
| MFTVFLLVVLATTVVSFPSDIATEGRNAAAKAFDLISSIVRKGCCSNPACAGNNPHVCRQGR | 0 |
| MFTVFLLVVLATTVVSFPSDIATEGRNAAAKAFDLISSIVKKGCCSHPACSGNNPEFCRQGR | 0 |
| MGMRMMFTVFLLVALATTVVSFTSDRASDRRNAAAKAFDLISSIVKKGCCSHPACSGNNPEFCRQGR | 0 |
| GGCCSHPACQNNPDYCX | 0 |
| NPASCCSCADVDPGRASRKTPKGEDQVFIKEKDRC | 1 |
| FDGRNAAGNDKMSALMALTTRGCCSHPVCSAMSPICG | 0 |
| FDGRNTSANNKATDLMALPVRGCCSDPPCRNKHPDLCGGRR | 1 |
| MFTVFLLVVLATAVVSFTSDRASDDGKAAASDLITLTIKGCCSRPPCIANNPDLCG | 0 |
| MGMRMMFTVFLLVVLATTVVSFTSGRRTFHGRNAAAKASGLVSLTDRRPECCSHPACNVDHPEICR | 0 |
| MGMRMMFTVFLLVVLATAVVSFTSDRASDDRKAAASDLITLTIKGCCSRPPCIANDPDLCG | 0 |
| NIQIICCKHTPACCTX | 0 |
| PCCSKLHDNSCCGLX | 0 |
| GCCSRPPCIANNPDLCX | 0 |
| GCCSDPPCRNKHPDLCX | 0 |
| DKQTCCGYRMCVPCX | 0 |
| GCCSHPVCSAMSPICX | 0 |
| GVVEHCCYRPCSNAEFKKFCG | 1 |
| TTEECCPFIVGCCS | 0 |
| ECCEDGWCCTAAX | 0 |
| KPCCSIHDNSCCGL | 1 |
| SDGRNDAAKASGLVSLTDRRPECCSDPRCNSSHPELCG | 1 |
| MFTVFLLVVLATTVVSFSSGRSTFRGRNAAAKASGLVSLTDRRPECCSDPRCNSSHPELCGGRR | 1 |
| MGMRMMFVVFLLVVLASTVVSSTSGRRAFHGRNAAAKASGLVSLTDRRPECCSDPRCNSSHPELCGGRR | 1 |
| MFTVFLLVVLATTVVSFTSGRSTFRGRNAAAKASGLVSLTDRRPQCCSHPACNVDHPEICR | 0 |
| QCCWYFDISCCLWP | 0 |
| KPCCSIHDSSCCGI | 0 |
| GVVEHCCKRACSNAEFMQFCG | 0 |
| EFALMFTVFLLVVLATTGVSFTLDRASDGGNAVAKKSDVTARFNWRCCLIPACRRNHKKFCG | 1 |
| GCCSNPACMVNNPQIC | 0 |
| VRCLEKSGAQPNKLFRPPCCQKGPSFARHSRCVYYTQSRE | 1 |
| RVGDDCCVGNVGTCCS | 1 |
| TVRGHCCPYYPQCCPS | 1 |
| SDGRNAAADDKASDPIALTVRGGCCSHPVCYTKNPNCGGRR | 1 |
| GGCCSHPVCYTKNPNCG | 1 |
| EILNIIDSISDVAKQICCQITVDCCVLDEE | 0 |
| EIVNIIDSISDVAKQICCEITVQCCVLDEE | 0 |
| TLLNILRDGDNCCIDKQGCCPW | 1 |
| GCCPRSFLCC | 0 |
| DCCLRHFLCCV | 0 |
| DCCRRNFLCC | 1 |
| GGCCSYPPCAVSNPQHC | 1 |
| SCCPNNPACCH | 1 |
| MGMRMMFIVFLLIVLATTVVSFTSDRAFDGGNAAADMITRAIGGTCCPIPSCASTFPRLCG | 0 |
| MGMRMMFILFLLVVLATTVVSFPSDRASDGVNTAAELIARATWDYCCPPYICDTHFPSHCK | 0 |
| GIHELAKSVCCTATGISICC | 0 |
| GCCSHPACSGNNPEFCRQ | 1 |
| KGCCSNPACAGNNPHVCRQ | 1 |
| KCCSIPKCYKNNKKMC | 1 |
| EPVPTTIIDFGECCRDPSCWVKVKDFKCPGAVPPS | 0 |
| MGMRMMFTVFLLVVLATTVVSSTSRSRPKSRRREPVPTTIIDFGECCRDPSCWVKVKDFKCPGAVPPS | 0 |
| GEPVPTTIINFGECCRDPSCWVKVKDFKCPGAVPPS | 0 |
| GVVEHCCKRACSNAEFMQFCGNS | 1 |
| MGMRMMFTVFLLVVLATTVVSSTSRSRPKSRRGEPVPTTIINFGECCRDPSCWVKVKDFKCPGAVPPS | 0 |
| MGMRMMFTVFLFVVLATTGVSFTLDRASDGGNAVAKKSDVTARFNWRCCLIPACRRNHKKFCG | 0 |
| ATWDYCCPPYICDTHFPSHC | 0 |
| AIGGTCCPIPSCASTFPRLC | 0 |
| GILELAKTVCCSATGISICC | 0 |
| GIISLAKSLCCTGIGISFCC | 0 |
| GIFALAKSVCCTAAKLSFCC | 0 |
| FNWRCCLIPACRRNHKKFCX | 0 |
| SNKRKNAAMLDMIAQHAIRGCCSDPRCRYRCR | 1 |
| MGMRMMFTVFLLVVLTTTVVSSTSVRASDGRNAAADNRASDLIAQIVRRGCCSHPACNVNNPHICG | 0 |
| MGMRMMFTMFLLVVLSTTVVSLPVDHASNGRDAAADSNAADQIAQTARDPCCSNPSCAQTHPEICRRMLQNPLNHDMSPSA | 0 |
| DMCCHPACMNHFNCX | 0 |
| MGMRMMFTVFLLVVLATTVVSFTSDRGSDGRNAAAKDKASDLVALTVKGCCSNPPCYANNQAYCNGRR | 0 |
| MKLSVMFIVFLMLTMPMTDGGIIRSANNGENADALAGDRATKVLELLLKSSCPPACCPTC | 0 |
| CCPGNFACCX | 0 |
| CCPKKPYCCPG | 1 |
| GCCSNPPCYANNQAYCN | 1 |
| SSCPPACCPTC | 0 |
| ILLLLIASAPSVDAQLKTKDDVPLASFHANVKRTLQKLLNKRCCQIVPQCCEWN | 0 |
| MLKMGVVLFIFLVLFPLATLQLDADQPVERHAENKQLLNPDERRGIILHALRKRCCHSSWCKHLC | 0 |
| MKLTCMMIVAVLFLTAWTFVTADDSGNGLENLFSKAHHEMKNPKDSKLNKRCLDGGEICGILFQAAAVGGALFSSAHETTVMSSTPLCATWLDL | 0 |
| MLKMGVVLFIVLVLFPLATLQLDADQPVERYAENKQLLNPDERRGIILHALGQRVCCPFGGCHELCTADDG | 0 |
| MGMRMMFTVFLLVVLATTVVSFTSNRVLDPAFRRRNAAAKASDLIALNARRPECCTHPACHVSNPELCS | 0 |
| MGMRMMFTMFLLVVLATTVVSFTSNRAFRRRNAVAKASDLIALNARRPECCTHPACHVSHPELCG | 0 |
| MGMRMMFTVFLLVVLATTVVSFTSDRGSDGRNAAAKDKASDLVALTVKGCCSNPPCYANNQAYCNGRR | 0 |
| MFTVFLLVVLTTTVVSFPSDSASGGRDDEAKDERSDMYELKRNGHCCHPACGGKYVKCGR | 1 |
| MGMRMMFTVFLLVVLATTVVSFTSDRASDGRKAAAKDKASDLVALTVKGCCSHPACSVNNPDICG | 0 |
| MGMRMMSTVFLLVVLATTVVSFTSDRASDGRKAAAKDKASDLVALTVKGCCSHPACSVNNPDICG | 0 |
| MKLTCMMIVAVLFLTAWTFATADDPRNGLENLFSKAHHEMKNPKDSKLNKRCIDGGEICDIFFFKLLQWVVHYSCLRMKLP | 0 |
| MKLTCMMIVAVLFLTAWTFATADDPRNGLENLFSKAHHEMKNPKDSKLNKRCLDAGEMCDLLIQNAAVGGALFSSAHKTTVMSSTPLCATWLDL | 0 |
| MKLTCMMIVAVLFLTAWTFAAADDPRNGLENLFSKAHHEMKNPKDSKLNKRCIDGGEICDIFFQTAAVGGALFSSAHETTVMSSTPLCATWLDL | 0 |
| MKLTCMMIVAVLFLTAWTFATADDPRNGLENLFSKAHHEMKNPKDSKLNKRCIDGGEICDIFFQTAAVGGALFSSAHETTVMSSTPLCATWLDL | 0 |
| MSGHTSVNFLLLSIVALGMVATVICLCDSYISSELCEHPEETCLLPQSYVLSVESIQTGSVYVLGSVPHISKTSG | 0 |
| NACCIVRQCC | 0 |
| PECCTHPACHVSNPELCX | 0 |
| VGVCCGYKLCHPC | 0 |
| NGVCCGYKLCHPC | 1 |
| CCQIVPQCCEWVSD | 0 |
| CCPGWELCCEWDDWW | 1 |
| NGCCRAGDCCS | 1 |
| NGCCRAGDCCSRFEIKENDF | 1 |
| SDGRNAAAKDKASDLVALTVKGCCSHPACSVNNPDICG | 1 |
| MFTVFLLVVLTTTVVSYPSDSASDGRDDEAKDERSDMYKSKRNGRCCHPACGKHFSCGR | 1 |
| ASDGRDDEAKDERSDMYELKRNGRCCHPACGAKYFKCGG | 1 |
| NGRCCHPACAKYFSCGR | 1 |
| GCCSHPPCFLNNPDYC | 1 |
| GCCSHPPCAMNNPDYC | 1 |
| ECCSDGWCCPAX | 0 |
| SCCPTILSCCFV | 0 |
| CCYETPGCCVIX | 0 |
| YCCYFDYSCCLYLR | 1 |
| YCCVYDYSCCLSWX | 0 |
| YDCCKTFECCHWX | 0 |
| MCCLGTSGCCPWX | 0 |
| MGMRMMFTVFLLVVLATTVVSFTSDRASDGGNAAASDLIALTIKGCCSRPPCALSNPDYCG | 0 |
| MGMRMMFTVFLLVVLATTVVSFTSDRASDDGNAAASDLIALTIKGCCSLPPCALSNPDYCG | 0 |
| MFTVFLLVVLATTVVSFTSDRASDGGNAAMSDLIALTIKGCCSHPPCFLNNPDYCG | 0 |
| MFTVFLLVVLATTVISFTSDRASDGGNAAASDLIALTIKGCCSHPPCAMNNPDYCG | 0 |
| GCCSHPACNVNNPHICGX | 0 |
| GCCSHPACSGNNREYCRES | 1 |
| GCCSHPPCAQNNQDYC | 1 |
| GCCSNPVCWQNNAEYCRES | 1 |
| ASDGRDDEAIDERSDMYELKRNGRCCHPACAKYFSCGR | 1 |
| ASDGRDDEAIDERSDMYELKRNGRCCHPACGKYFSCGR | 1 |
| ASDGRNAAANDKASGVNGCCSNPACHVDHAELCRRRR | 1 |
| NGHCCHPACGGKYVKC | 1 |
| NGRCCHPACGKHFSC | 1 |
| NGRCCHPACGAKYFKCG | 1 |
| CCQIVPQCCEWN | 0 |
| GRCCHPACAKYFSCX | 0 |
| NGRCCHPACGKYFSC | 1 |
| EDCCFIDDCWASHPEACGGGMELLRQDPLNRDMPPSA | 1 |
| GNWCCSARVCC | 1 |
| MFTVFLLVVLATTVVSPTSDRASDRRNAAAKAFDLRYSTAKRGCCSNPVCWQNNAEYCRESG | 1 |
| MFTVFLLVVLATTVVSFTSDRASDGGNVAASHLIALTIKGCCSHPPCAQNNQDYCG | 0 |
| MFTVFLLVVLSTTVVSSTSDRASDRRNAAAKASDLMYSTVKKGCCSHPACSGNNREYCRESG | 1 |
| NGRCCHPACAKYFSC | 1 |
| CCQVMPQCCEWN | 0 |
| CCPGWELCCEWDDGW | 1 |
| CCPGWELCCEWDEW | 1 |
| NTAAKVKYSKTQEECCPNPPCFAVNSALCGA | 1 |
| EDCCALHACWMYC | 0 |
| LDCCDIHACWSAHPELCG | 0 |
| TGCCEFPYCAENIPELCG | 0 |
| LDCCDIHACWMYC | 0 |
| DPCCSNPSCAQTHPEIC | 1 |
| ALCCYGYRFCCPIFX | 1 |
| LWQNTWCCRDHLRCCX | 0 |
| NECCDNPPCKSSNPDLCDWRS | 1 |
| ALCCYGYRFCCPNFR | 1 |
| GCCAHLPCALMYTACSW | 1 |
| GCCDDPSCRAENPFLCSWM | 1 |
| GCCDDPPCRARYPFLCI | 1 |
| GCCADPSCSILMPYFCI | 0 |
| GCCGNPSCSIHIPYVCN | 0 |
| LDPCCREPPCASTHTDICT | 1 |
| AAKVKYSKTPEECCPNPPCFATNSDICG | 1 |
| AANDKASVQIALTVQECCADSACSLTNPLIC | 0 |
| AANDKASVQIALTVQECCADAACSLTNPLIC | 0 |
| DPCCSYPACGANHPEICG | 1 |
| GTRCARCARTCRTACAT | 1 |
| EECCPNPPCFATNSDICG | 0 |
| QSCCSAPLCALLYRVMC | 0 |
| GCCSDPPCRHKHQDLCX | 0 |
| QSCCSTPPCALLYMEMC | 0 |
| QSCCAAPSCFMLC | 0 |
| LDPCCRDPPCASTHIDRC | 1 |
| AAKVKYSKTPEECCPNPPCFATHSEICG | 1 |
| AAKVKYSKTPEECCPNPPCFATHSNICG | 1 |
| APNVKDSKASGSCCDNPSCAVNNSHC | 1 |
| APNVKDSKASGSCCDNPSCAVNNRHC | 1 |
| AAKVKYSNTPEECCSNPPCFATHSEICG | 1 |
| STCCGYRMCVPCX | 0 |
| IHLKLGCCIMFTCCM | 0 |
| PGCCARLICCP | 0 |
| CCHSSWCKHLC | 1 |
| CLDGGEICGICFQAAAVGGALFSSAHETTVMSSTPLCATWLDL | 0 |
| LCDSYISSELCEHPEETCLLPQSYVLSVESIQTGSVYVLEACRIFTKTSX | 0 |
| VPAEPILEIICPEMCDKGVGEEPFCHCTEKRDAVSSRI | 0 |
| VLAELILEIICLEMCEEGVGEEPFCHCTEKRDAVFSRI | 0 |
| GPNTGELCDGVEQNCRYPYCFIVVCL | 0 |
| VPAEPILEIICPEMCDEGVGEEPFCHCTEKRDAVSSRI | 0 |
| ACCVYKICYPC | 0 |
| VCCPFGGCHELCTADDX | 0 |
| ECCTHPACHVSNPELCX | 0 |
| RPECCTHPACHVSNPELCS | 1 |
| PECCTHPACHVSHPELCX | 0 |
| GCCSHPACRVHYPHVCYX | 0 |
| GCCSNPPCYANNQAYCNX | 0 |
| GCCSHPACSVNNPDICX | 0 |
| CCITFESCCEFDL | 0 |
| FCCRTQEVCCEAIKNX | 0 |
| FLTQQSPRDFAKSVMQLLHYNWIDCCNYGVSDCCI | 0 |
| GCCSHPACNVDHPEICX | 0 |
| QCCSNPPCAHEHCR | 1 |
| EGCCSNPACRTNHPEVCD | 1 |
| MFTVFLLVVLTTTVVSFPSDRASDGRDDEAKDERSDMYESKRDGRCCHPACGQNYSCGR | 1 |
| MGMRMMFTVFLLVVLATTVVSFPSDRASDGRNAAANDKASDVITLALKGCCSNPVCHLEHSNLCGRRR | 0 |
| FLTQQSPRDFAKSIMQLLHYNRIDCCNYGVSDCC | 1 |
| CCHPACGQNYSC | 1 |
| NGRCCHPACARKYNCX | 0 |
| GRCCHPACGKNYSCX | 0 |
| CCHPACGKNYSCX | 0 |
| GCCSNPVCHLEHSNLCX | 0 |
| VCCGYKLCHPC | 1 |
| GICCGVSFCYPC | 0 |
| DGRCCHPACAKHFNCX | 0 |
| STCCGYRMCVPCX | 0 |
| STCCGYRMCVPCX | 0 |
| NCCIYENWCCEWI | 1 |
| MGMRMMFTLFLLAVLSTTVVSFTLDRASNGRDAAADSKAADQIAQTVRDECCSNPSCAQTHPEICRRTLMLQNPLNHDMSPSA | 1 |
| AANDKASDLMALRDGCCSDPACAVNHPDICGGGR | 0 |
| AANDKASDLMALRGGCCSRPPCILEHPEICGRRR | 1 |
| AANDKASDLMALRGGCCSRPPCILKHPEICGRRR | 1 |
| AAKFKAPALMELSVRQGCCSYPACAVSNPDICGGGR | 0 |
| AASDKASELMALAVRGCCSHPACAGNNPDICG | 0 |
| AANKKASDLMALTVRGCCVYPPCAVNHPDICG | 0 |
| HDNWIDCCHDGDCC | 1 |
| VMQLRYYNWIDCCFDGDCCN | 0 |
| GCCAMLTCCV | 1 |
| GCCARLTCCV | 1 |
| DCQPCGHNVCC | 1 |
| GCCVYPPCAVNHPDICRG | 0 |
| DECCPNPPCKASNPDLCDWRS | 1 |
| DGCCSDPACSVNHPDICGG | 0 |
| GCCSNPTCAGNNGNICX | 0 |
| GCCSNPACSVNHPELCX | 0 |
| GGCCSRPPCILEHPEIC | 0 |
| DGCCSDPACAVNHPDICGG | 0 |
| DECCSNPSCAQTHPEIC | 1 |
| IALIATRECCANPQCWAKNC | 1 |
| DGCCSSPSCSVNNPDICGG | 0 |
| QGCCSDPACAVSNPDICSG | 0 |
| MGMRMMFTVFLLVVLATAVVSFSSDRASHGRNAAAKVFDRIALIATRECCANPQCWAKNCR | 0 |
| DECCPDPPCKASNPDLCDWRS | 1 |
| DDCCPNPPCKASNPDLCDWRS | 1 |
| QCCANPPCKHVNCX | 0 |
| NECCDNPPCKSSNPDLCDWIS | 1 |
| QGCCSDPACAVSNPDICX | 0 |
| DGCCSNPSCSVNNPDICX | 0 |
| DGCCPNPSCSVNNPDICX | 0 |
| NECCDNPPCKSSNPDLCDWRS | 1 |
| GGCCSRPPCILKHPEIC | 0 |
| MGMRMMFTMFLLVVLATTVVSFTSDHASDGRNTAANDKASDLMALRDGCCSSPSCSVNNPDICGGGR | 0 |
| MGMRMMFTMFLLVVLAITVVSFTSDHASDGRNTAANDKASNLMALRDECCPDPPCKASNPDLCDWRS | 0 |
| MGMRMMFTMFVLVVLATTVVSFPLDHASNGRDAAAYDKATPLIARDMEECCSHPACQGINPDYCD | 0 |
| TLQSLSMARICCSRKLWCCGIPF | 0 |
| DVGDDCCSDNVGTCC | 1 |
| DPCCSNPSCAQTHPEIC | 1 |
| DMEECCSHPACQGINPDYCD | 1 |
| PQCCSHPACNVDHPEICD | 1 |
| ECCHPACGKHFSC | 1 |
| ECCNPACGRHYSCKG | 1 |
| NGRCCHPACGKHFSC | 1 |
| MFTVFLLVVLATTVVSFTSGRSTFRGRNAAAKASGLVSLTDRRPQCCSHPACNVDHPEICD | 0 |
| MFTVFLLVVLATTVVSFPSERASDGRDDTAKDEGSDMDKLVEKKECCHPACGKHFSCGR | 1 |
| MFTVFLLVVLATTVVSFPSERASDGRDDTAKDEGSDMEKLVEKKECCNPACGRHYSCKGGR | 1 |
| MFTVFLLVVLTITVVSFPSDRASDGRDDEAKDERSDMYKSKRNGRCCHPACGKHFSCGR | 1 |
| MHTLEMLLLLLLLLPLALGEGDGQAVAGERNPSETRSTREHLLQRLVGRDCQPCGHNVCCRSGNGGEYRRFT | 1 |
| MHTLEMLLLLLLLLPLALGEGDGQAVAGERNPSETRSTREHLLQRLVGRDCQPCGHNVCCRSGNDGEYRRFT | 1 |
| MHTLEMLLLLLLLLPLALGEGDGQAVAGERNPSEARSTREHLLQRLVGRDCQPCGHNVCCRSGNDGEYRRFT | 1 |
| AANDKASDLMALTVRGCCVYPPCAVNHPDICRGGR | 0 |
| MGMRMMFTMFLLVVLAITVVSFTSDHASDGRNTAANDKASKLMALRNECCDNPPCKSSNPDLCDWRS | 1 |
| SDGRNTAANDKASKLMALRNECCDNPPCKSSNPDLCDWRS | 1 |
| SDGRNTAANDKASNLMALRDDCCPNPPCKASNPDLCDWRS | 1 |
| MGMRMMFTVFLLVALATTVASFTLDRASNGRNAAADDKPSDWIALAIKQCCANPPCKHVNCR | 0 |
| SNGRNAAADDKPSDWIALAIKQCCANPPCKHVNCR | 1 |
| TSDHASDGRNTAANDKASKLMALRNECCDNPPCKSSNPDLCDWIS | 1 |
| MGMRMIFTVFLLVALATTVTSDRVSNGRKAAAKFKAPALMELSVRQGCCSDPACAVSNPDICSGGR | 0 |
| MGMRMMFTVFLLVVLATTVTSDRVSNGRKAAAKLKAPALMELSVRQGCCSDPACAVSNPDICGGGR | 0 |
| SDGRNTAANDKASDLMALRDGCCPNPSCSVNNPDICGGGR | 1 |
| SDGRNAAADDKASDLIGLAVRGCCSNPACSVNHPELCGRRR | 1 |
| FDGRNAAASDKASELMALAVRGCCSNPTCAGNNGNICG | 1 |
| AANDKASDLMALRDGCCSDPACSVNHPDICGGGR | 0 |
| MGMRMMFTMFLLVVLAITVVSFTSDHASDGRNTAANDKASNLMALRDECCPNPPCKASNPDLCDWRS | 0 |
| AANDKASNLMALRDECCPNPPCKASNPDLCDWRS | 1 |
| SDGRNTAANDKASDLMALRDGCCSNPSCSVNNPDICGGGR | 1 |
| LDPCCREPPCASTHIDRC | 1 |
| QGCCSYPACAVSNPDICGG | 0 |
| GCCVYPPCAVNHPDIC | 0 |
| VRAVMFIVFLMLTMPMTGAAVSRRAANGGEAVGDRAANLMALLQESLCPPGCYPSCTNCRYMFP | 0 |
| KLSVMFIVFLMLTMPMTGAAVSHRAANGGEAVGDRAANLMALLQESLCPPGCYPSCTDCRYMFP | 0 |
| MKLSVMFIVFQQMMLTMPMTGAAVSRRAANDGNGGEAVGDRAANLMALLQESLCPPGCYPSCTDCRYMFP | 0 |
| MKLSVMFIVFLMLTMPMTGAAVSHRAANGEAVGDRAANLMALLQESLCPPGCYPSCTDCRYMFP | 0 |
| MKLSVMFIVFLMLTVPMTGAAVSRRAANGGEAVGDRAANLMALLQERQCPPTCPSCDDC | 0 |
| MGMRMMFTVFLLVVLATTVDSFNVDRASDDRDSAAMHTEYDVIATDNCIPCSHPACGINRGKCR | 0 |
| MGMRMMFTVFLLVVLATTVDSFNSDRASDGRDAEVVSTESDVIVTCEPCMNPACGPNYGKCR | 0 |
| MGMRMMFAVFLLVVLATTVVSFNSDRASDGRNAAANVKASDLMARVLEKDCPPHPVPGMHKCVCLKTCR | 0 |
| MFTVFLLVVLVTTVVFSTSDHRPASNHENRRASKRISEMTWEECCTNPVCRQHYMHYC | 1 |
| MFTVFLLVILATTVVPFPSDRDPASNHENSKGSNRNAWLTPEECCAAPACREMILEFCLAGEAFAAALDGFRRLPYRLSSE | 0 |
| MFTVFLLVVLATTVVSFPSDRDPASNHENSKGSNRNAWFTPEECCAAPACRGMILEFCLAGEAFAAALDGFRRLPYRLSSE | 0 |
| SDGRNAGADRKGFGLISQMFKLSCCADPACKHTPGC | 1 |
| LDGRNAAADFETSDLLAMTIRGGCCSYPPCIANNPLCGGKR | 0 |
| MGMRMMFTVFLLVVLATTVVSFTSDRTSDGRNAAFNAFDLIALTARQNCCNVPGCWAKYKHLCGRKR | 0 |
| VVLGPASDGRNAAFNAFDLIALTARQNCCNVPGCWAKYKHLCGRKR | 1 |
| FPRPRICNLACRAGIGHKYPFCHCRX | 0 |
| GPGSAICNMACRLEHGHLYPFCNCDX | 0 |
| LLDYVTGACCAGLNFVCC | 1 |
| GCCSLPPCAANNPDYCX | 0 |
| GCCSLPPCALSNPDYCX | 0 |
| GCCSRPPCALSNPDYC | 1 |
| PCCSGWCFFACA | 1 |
| FDGRNAAANDKASDLVALTVRGCCSHPACSVNHPELCG | 1 |
| MGKLTILVLVAAVLLSTQAMVQDQPAGRDAVPRDDNPGGTSGKFVNAQRQDECEIGSWCQFCTALWCG | 0 |
| GCCSHPACSVNHPELCX | 0 |
| SCSCQKHFSCCDX | 0 |
| DECCAIPFCAKIFPGRCP | 0 |
| DECCAIPLCAKIFPGRCP | 0 |
| GIRGNCCMFHTCPIDYSRFYCP | 0 |
| GPGSAICNMACRLGQGHMYPFCNCNX | 0 |
| GPGSAICNMACRLEHGHLYPFCHCRX | 0 |
| SCSCKRNFLCCX | 0 |
| GCCSHPACAGNNPDIC | 1 |
| LLLLASPAASNPLETRIQSDSIRAALEDADMKTEKGFLSSIVGNLGTVGNLVGSVCCQITNSCCPED | 0 |
| GGCCSYPPCIANNPLCX | 0 |
| GCCPKQMRCCTLX | 0 |
| SGCCKHPACGKNRC | 1 |
| SGCCKHOACGKNRC | 0 |
| QSPGCCWNPACVKNRCX | 0 |
| RDPCCSNPVCTVHNPQICX | 0 |
| ACCSYPPCNVNYPEICGGR | 1 |
| SDGRDAAANDKATDLIALTARRDPCCSNPVCTVHNPQICG | 0 |
| MFTVFLLVVLATTVGSFTLDRASDGRDAAANDKATDLIALTARRDPCCSNPVCTVHNPQICG | 0 |
| MFTVFLLVVLVTTVVSFNSDRALGGRNAAAKASDKIASILGRRACCSYPPCNVNYPEICGGRG | 0 |
| DDCCPDPACRQNHPEICPS | 1 |
| DDCCPDPACRQNHPELCST | 1 |
| RAALEDADMKTEKGVLNAIFSNLGDLGNLVSSVCCKATTSCCPED | 0 |
| AALEDADMKTEKGFLSSIVGNLGTVGNLVGSVCCQITNSCCPED | 0 |
| CCPRKIWCCMIP | 0 |
| ECCPQSPPCCHYYYYGSW | 1 |
| SCCPEEITCCPWX | 0 |
| SCCPEEPCCFW | 1 |
| LSCCADPACKHTPGC | 1 |
| NAWFTPEECCAAPACRGMILEFCLAGEAFAAALDGFRRLPYR | 0 |
| NAWLTPEECCAAPACREMILEFCLAGEAFAAALDGFRRLPYR | 0 |
| ISEMTWEECCTNPVCRQHYMHYC | 1 |
| DAEVVSTESDVIVTCEPCMNPACGPNYGKC | 0 |
| DSAAMHTEYDVIATDNCIPCSHPACGINRGKC | 0 |
| QNCCNVPGCWAKYKHLCX | 0 |
| QCPPTCPSCDDC | 1 |
| AANLMALLQESLCPPGCYPSCTNCRYMFP | 0 |
| QDECEIGSWCQFCTALWC | 0 |
| VLEKDCPPHPVPGMHKCVCLKTC | 0 |
| SCCPSPTSCCPWX | 0 |
| GLNAIFSNLGDLGNLVSSVCCQSLLRVCCPED | 0 |
| GCCEDKTCCFIX | 0 |
| AANLMALLQESLCPPGCYPSCTDCRYMFP | 0 |
| LDCCDIHACWSAHPELCG | 0 |
| GARGCNTGYTAYGCNCC | 1 |
| SQCCAVKKNCCHV | 1 |
| MKFLLFLSVALLLTSFIETVTVNKAGMERPSRALVGRGCPAECPDTCSSSGSCAPDFIG | 0 |
| PVNEAGVERLFRALVGRGCPADCPNTCDSSNECSPNFPG | 1 |
| MKFLLFLSVALLLTSFIETEAGPVNEAGVERLFRALVGRGCPADCPNTCDSSNKCSPGFPG | 0 |
| MKFLLFLSVALLLTSFIETEAGPVNEARVERLFRALVGRGCPADCPNTCDSSNKCSPGFPG | 0 |
| MGDLSEADSMKHQLQRRDCGRCGLGQICDAGACRPSTMM | 1 |
| MKLSVTFIALMLTMTLTQGFVLQAIDGRDNSGLDDLSEADSMEHQLQRRDCGRCPLGQYCDAEAGMCKPTLIM | 0 |
| MTAKATLLVLALVVMATSGVSSASVAGGPVVNSDTVSRSDPERLSTRGCVANCQANQTGIDCIKYCGIGIGRRDITQQ | 0 |
| IRVVLGPASDGRDDEAKDKRSDMYESDRNGRCCHPSCGRKYNCGR | 1 |
| FCCPVIRYCCW | 0 |
| FCCPFIRYCCW | 0 |
| GCCSYPPCFATNSGYCX | 0 |
| GCCSYPPCFATNPDCX | 0 |
| GCCSYPPCFATNSDYCX | 0 |
| MFTVFLLVVLATTVVSFTSDRASDGRKDAASGLIALTMKGCCSYPPCFATNPDCGRRR | 0 |
| MFTVFLLVVLATTVVSFTSDRASDGRKDAASGLIALTIKGCCSYPPCFATNSDYCG | 0 |
| SCGYPCSHCGIPGCYPGX | 0 |
| MRFYIGLMAALMLTSILRTDSASVDQTGAEGGLALIERVIRQRDAADVKPVARTNEGPGRDPAPCCQHPIETCCRR | 0 |
| MRFYIGLMAALMLTSVLRTDSASVGQTGTKSELAVIERVIRQRDAADVKPVARQNEGPGRDPAPCCQHPIETCCRR | 0 |
| MRFYIGLMAALMLTSILRTDSASVGQTGTKSELALIERVIRQRDAADVKPVARHNDGPGRDPAPCCQHPIETCCRR | 0 |
| RIQSDLIRAALEDADMKNEKNILSSIMGSLGTIGNVVGNVCCSITKSCCASWLFPRLE | 0 |
| NSEDGSPYPGPGQQPNCCKWPIVTCCNR | 1 |
| DECCSNPACRVNNPHVCRRR | 1 |
| IRDECCSNPACRVNNPHVCRRR | 1 |
| AALEDADMKNEKNILSSIMGSLGTIGNVVGNVCCSITKSCCASW | 0 |
| SIIDSVKTFCCSTFNLGICCS | 0 |
| YRRRQCPPWCSGEPCRKGTC | 1 |
| MNVTVMFLVLLLLTMPLTDGFNIRATNGGELFGPVQRDAGNVLDHGFQRRRDCPPWCPTSHCNAGTC | 0 |
| MNVTVMFLVLLLLTMPLTDGFNIRATNGGELFGPVQRDAGNVLDHGFQRRRECPPRCPTSHCNAGTC | 0 |
| MNVTVMFLVLLLLTMPLTDGFNIRATNGGELFGPVQRDAGNVLDHGFQRRRECPPWCPTSHCNAGTC | 0 |
| VFNKAKDKFNELTSGLGVHFDRIVDLLIDQIDSGMTEAACIKVCESSASKLLGDASPMAGTVCAPVCTAALAKLEEVA | 0 |
| SCCARNPACRHNHPCV | 1 |
| SCCARNPACRHNHPCV | 1 |
| MKVSVTFIVLLTLTATLSHGFILRAIDGRENLGGWGNSQVENRQDYQIQTRGCPEDYPCKAGYVCDKGICRPLVIM | 0 |
| WDVDQCIYYCLNGVVGYSYTECQTMCT | 0 |
| GGVGRCIYNCMNSGGGLNFIQCKTMCY | 1 |
| MFRLGVFLLTFLLLVSMATSEYSRGRIMARASECVNECVESGHNTFHCERHCSNT | 0 |
| ICCNPACGPKYSCX | 0 |
| RWDVDQCIYYCLNGVVGYSYTECQTMCT | 0 |
| NGRCCHPACGKHFSC | 1 |
| MNVTVMFLVLLLTMPLTDGFNIRAINGGELFGLVQRDAGNALDHGFYRRGDCPPWCVGARCRAEKC | 0 |
| MGMRMMFIVFLLVVLATTVVSNRASNRENRRASNWNTRMIDCCDIPRCYNERPEECREESSG | 1 |
| LLGLVTGACCAVLKFSFCC | 1 |
| MGMRMMFTVFLLVVLATTVVSSTSDHDAASNHENRRASNRINQRTWEECCKRPPCRVQHMGHC | 1 |
| MGMRMMFIVFLLVVLATTVVSFTSDRTSDSRNAAANAFDLIARYLGIDCCRIPSCFAKYGSKCSKIR | 0 |
| MGMRMMFTVFLLVVLATTVVSFTSDRSPASDSRNAAANAFDLIARYLGIDCCRIPSCFAKYGSKCSKIR | 0 |
| CCHPSCGRKYNC | 1 |
| MGMRMMFTVFLLVVLATTVVSFTSDRTSDSRNAAANAFDLIARYLGIDCCRIPSCFAKYGSKCSKIR | 0 |
| MGMRMMFTVFLLVVLATTVLSFTSDRASEGRNAAAKDKASDLVALRVRGCCAIHECRLQNAAYCGGIY | 0 |
| MGMRMMFTVFLLVALATTVVPFTSDRASASRNAATDNKASELKALNARIPCCSYPACAQSNIDLCGGRR | 0 |
| MGMRMMFTVFLLVVLATTVVSFTSDRASEGRNAAAKDKASDLVALTVRGCCAIRECRLQNAAYCGGIY | 0 |
| MFTVFLLVVLATTVVSFPSDRGPASNHENSKGSNRNVWFTPDECCRDPPCREKILEYCLAGEAVAAAFGFRQLPYRLSSE | 0 |
| LATTVVSFTSDRESNHENRRASNRIPRAMWQECCADPPCRQNHMEHCPAN | 1 |
| SDRASASRNAATDDKASELKALNIRTVCCYYPTCAESNPYLCYGGR | 1 |
| MGMRMMFTVFLLVVLATTVVSFTSDRASDGRNAAANAFDLIALIARQNCCSIPSCWEKYKCS | 0 |
| MNVTVMFLVLLLTMPLTDGFNIRAINGGELFGLVQRDAGNALDHGFYRRGDCPPWCVGARCRAGKC | 0 |
| MGMRMMFIVFLLVVLATTVVSFASDRTSDGRNAAANAFDLIARSFTINCCKIPSCFAKYGSKCSEVH | 0 |
| MKVAVVLLVSLLAVTYALPEKRIFFGGIVDKVKDTFTKIFNKAKETFDKITDGFDVDFDEVVDKLIAQIHSTPTKAACKAACKKGAKLILKAAAPLASQVCGPACNAALAKLEKIADDINDDDD | 0 |
| AALEDADMKNEKNILSSIMGSLGTIGNVVGNVCCSITKSCCASEE | 0 |
| GCCSNPVCHLEHPNACX | 0 |
| MFTVFLLVVLATTVVSFPSDRASDGRNAAASDRASDAAHQGCCSNPVCHVEHPELCRRRR | 1 |
| MFTVFLLVVLATTVVSFPSNRESDGANAEVRTDEPEEHDELGGNGCCGNPDCTSHSCD | 0 |
| MFTVFLLVVLAATVVSFPSDRASDGRDDEAKDERSDMHESDRKGRGRCCHPACGPNYSCGR | 1 |
| ASDGRNAAANDKASDLVALTVRRGCCSHPVCNLNNPQMCRGRR | 1 |
| ASDGRNAAANDVISLVLKGCCSNPVCHVEHSYMCGRRR | 1 |
| ASDGRNAAADDKASDLVALAVRGGCCSHPVCYFNNPQMCRGRR | 1 |
| ASDGRNAAADDKASDLVALAVRGGCCSHPVCNLNNPQMCRGRR | 1 |
| ASDAAHQGCCSNPVCHVEHPELC | 0 |
| TDEPEEHDELGGNGCCGNPDCTSHSCD | 1 |
| GRCCHPACGPNYSC | 1 |
| RGCCSHPVCNLNNPQMCR | 1 |
| GCCSNPVCHVEHSYMC | 1 |
| GGCCSHPVCYFNNPQMCR | 1 |
| GGCCSHPVCNLNNPQMCR | 1 |
| CCPTFYFCCNL | 1 |
| MFTVFLLVVLATTVVSFPSDRASDGRNAAANDKASDVITLALKGCCSNPVCHLEHSNLCGRRR | 0 |
| ASDGRDDEAKDERSDMYESDRNGRCCHPACAERYNCGR | 1 |
| ASDGRNAAANDKASDVIPLALQGCCSNPVCHVDHPELCLRRR | 1 |
| DDTDAAAGEILEFLCPFFCIGGIGDEYCDCQE | 0 |
| DCCSRPPCRWSHPELCS | 1 |
| RPECCSHPACNVDHPEIC | 1 |
| GCCSVPPCIANHPELCV | 0 |
| MGMRMMFTVFLLVVLATTVVSFMSGRASHGRNAAASDLIALTIKGCCSVPPCIANHPELCG | 0 |
| GCCSVPPCIANHPELCX | 0 |
| GCCSVPPCTANHPELC | 1 |
| GCCSDPRCAWRCX | 0 |
| MGMRMMFTMFLLVVLATTVASINLDRAFDGRNAAADDKASDLIAQIVRRGCCSDPRCAWRCG | 0 |
| PGCCNNPACGANRCG | 1 |
| ESDGANAEPSADQAARSAVNRPPGCCNNPACGANRCG | 1 |
| ASGADTCCSNPACQVQHSDLC | 1 |
| TLQSLWMTRGCCPGNVLCC | 0 |
| DRPSYCNLPADSGSGTNREQRIYYNSARKQCLTFPYKGKGGNANNFSRTNDCRRTCQYPAA | 1 |
| RCCGYKMCHPC | 1 |
| GVCCGVSFCYPC | 1 |
| GGCCSHPACFASNPDYCX | 0 |
| GGCCSHPACAANNQDYCX | 0 |
| DRPSYCNLPADSGSGTNREQRIYYNSARKQCLTFTYNGKGGNANNFSRTNDCRRTCQYPAA | 1 |
| CCSHPACAANNQDYCX | 0 |
| WDAYDCIQFCMRPEMRHTYAQCLSICT | 1 |
| WDVNDCIHFCLIGVVGRSYTECHTMCT | 0 |
| WDVNDCIHFCLIGVVERSYTECHTMCT | 0 |
| NGRCCHPACGKHFSCX | 0 |
| MGFRVLVLIVMVTTSALPFTFSEESGRSPFRPALRSEEAQALRHGLTLLLARRADGQTPDMHQPEMRRPEMRRPEVRRPEVRQPEFAESPVGQKRWDAYDCIQFCMRPEMRHTYAQCLSICT | 1 |
| MGFRVLVLIVMVTTSALPFTFSEESGRSPFRPALRSEEAQALRHGLTLLLARRADGQPSDMRQPEMRRPEMRRPEVRRPEVRQPEFAESPVGQKRWDVNDCIHFCLIGVVGRSYTECHTMCT | 1 |
| DDETTFPCNSGRCACLPEDSHSYTCQSP | 1 |
| PECCSDPRCNSTHPELCG | 1 |
| QECCSYPACNLDHPELC | 1 |
| MFTVFLLVVLATTVVSSTSGRRAFRGRNAAAKASGLVGLTDRRPECCSDPRCNSTHPELCGGRR | 1 |
| MFTVFLLVVLTITVVSFPSDRASDGRDDEAKDERSDMYKSKRNGRCCHPACGKHFSCGR | 1 |
| MFTVFLLVVLATTVVSFTSDRAFRGRNAAAKASGLVGLTDKRQECCSYPACNLDHPELCG | 1 |
| CCFLSGCFECW | 1 |
| AGCCPTIMYKTGACRTNRC | 1 |
| TAFGLRKCCKKHHGCHPC | 1 |
| FDDLMLLLCCRQGPVCFIPLNEWPCSRM | 0 |
| TAFGLRLCCKRHHGCHPCGRT | 1 |
| MFTVFLLVVLTITVVSFPSDRASDGRDDEAKDERSDMYKSKRNGRCCHPACGKHFSCGR | 1 |
| AQLPRDRTARVPAEPILELICPHMCRLGIGEPPFCKCRNKRDVVSSRITSRKRSMAM | 1 |
| AQLPRDRTARVPAQPVLDIICPGMCLLGIGEPFCNCSNKRDVVSSQITRRKRSMAV | 0 |
| MGMRMMFTMFLLVVLATTVVSFASDRASDGRNAAAKDKASDLVALTVKGCCSHPACSVNNPDICG | 0 |
| GGCCSYPACSVEHQDLCD | 1 |
| AIKLTCCSELTCAGNYPNIC | 0 |
| NGRCCHPACGKHFSC | 1 |
| NAECCYYPPCYEAYPEICL | 0 |
| GCCSHPACSVNHPELC | 1 |
| IAMAISSGACCAYPPCFEAYPERCL | 0 |
| GGCCSHPACAVNHPELC | 1 |
| AISSGACCAYPPCFEAYPERCL | 0 |
| GGCCSYPPCIASNPKCG | 1 |
| GGCCSHPACGVNHPELCX | 0 |
| SATCCNYPPCYETYPESCL | 1 |
| TCCKFQFLNFCCNE | 1 |
| ACCKVKFLNFCCNE | 0 |
| VCCQFEFLNFCC | 1 |
| CCPESPPCCHYF | 1 |
| CCPRRLACCII | 0 |
| CCPNKPCCFI | 0 |
| TLQSLWMTRGCCPGNVLCC | 0 |
| KAVCCQVFPGSDCCPGF | 0 |
| KCCPESPPCCHYYYYF | 1 |
| DEDWVDCCHMPRCCVED | 0 |
| SECCIRNFLCCX | 0 |
| ACCPYEPSCCI | 0 |
| RPCCPRDTWCCX | 0 |
| TLQMLRGVQICCPYILWCCLIP | 0 |
| GCCSNPACILNNPNQCX | 0 |
| KCCSNPACNRYNPAICD | 1 |
| VWCDWEWCYGDCHCFD | 1 |
| CPEMCNEGSGGVACSCSKRRDVVSSFV | 1 |
| VPAPHIMVPIYLCPILCKVGGVNTPFCNCT | 0 |
| GCPEDYPCKAGYVCDKGICRPLVIM | 0 |
| IVDLLIDQIDSGMTEAACIKVCESSASKLLGDASPMAGTVCAPVCTAALAKLEEVA | 0 |
| IPIVCSLLCKAGISIPIFCNCT | 0 |
| IPVCSVLCNAGVDVPFCDCT | 0 |
| VSAESVLEIICPYLCPLGIGALSFCNCPNKRDVVSSRITS | 0 |
| VPAEPILELICPHMCRLGIGEPPFCKCRNKRDVVSSRITS | 0 |
| VPAQPVLDIICPGMCLLGIGEPFCNCSNKRDVVSSQIT | 0 |
| QCGKHCPTCRHC | 1 |
| ASRACNPPCTGNAMCQNGHCGYIRF | 1 |
| QCPAPCYPNCEEC | 1 |
| ASNWNTRMIDCCDIPRCYNERPEECREESS | 1 |
| MFKMGVVLFTFLVLFPLATLQLDADQPVERHAENKQDLIPDERRGILLHALKQRVWCDWEWCYGDCHCFD | 0 |
| TWEECCKRPPCRVQHMGHC | 1 |
| YLGIDCCRIPSCFAKYGSKCSKI | 0 |
| GCCAIHECRLQNAAYCGGIY | 1 |
| IPCCSYPACAQSNIDLCG | 0 |
| GCCAIRECRLQNAAYCGGIY | 1 |
| NVWFTPDECCRDPPCREKILEYCLAGEAVAAAFGFRQLPYRLSSE | 1 |
| AMWQECCADPPCRQNHMEHCPAN | 1 |
| TVCCYYPTCAESNPYLCYG | 1 |
| CLGCGEDPRCC | 1 |
| QNCCSIPSCWEKYKCS | 1 |
| HFYLRDEDWVDCCQMGDCCDE | 1 |
| VADDCCVGKVGTCC | 1 |
| APAELILETICPHMCGTGIGEPFCNCRNKRDVVSSRII | 0 |
| SFTINCCKIPSCFAKYGSKCSEVH | 0 |
| RICCPWFALCCYY | 0 |
| GCCKLFDVGFCCGK | 1 |
| MLKMGVVLFTFLVLFPLATLQLDADQPVERHAENKQDLIPDERRGILLHALKQRVWCDWEWCYGDCHCFD | 0 |
| DGCCSTPPCIANHPELC | 0 |
| VLSGTCCMGYDCWCP | 0 |
| MFTVFLLVVLATTVVSFPSDRDSDGADAEASDEPVEFERDENGCCWNPSCPRPRCTGRR | 1 |
| ALEDADTKNDPRLIGGLISTALAACCKVKFLNFCCNEK | 0 |
| MSASGRLLFVCLTLGLVFALLGNPIPDVGDAARDAGPDGGSLERSETIEGRQATLSERNTRNRDVPACPEPPAPNPPENTNCENPVC | 1 |
| MGMRMVFTVFLLVVLATTVVSFTSDRASDGRNAAANDKASDLAALAVRGCCHDIFCKHNNPDICG | 0 |
| DGANAEATDNKPGVFERDEKKCCWNRACTRLVPCSK | 1 |
| SDRASDGRNAAANDRASDLVALTVRGCCTYPPCAVLSPLCD | 1 |
| MFTVFLLVVLTTTVVSFPSDRASDGRNAAANDKASDVVTLVLKGCCSTPPCAVLYCGRRR | 0 |
| ASGADTCCSNPVCQMQHHDLC | 1 |
| GCCSNPVCHLEHSNLCX | 0 |
| NECCWNPDCTSHRCD | 1 |
| MGMRMRMMFTVFLLVVLANTVVSFPSDRDSDGADAEASDEPVEFERDENGCCWNPSCPRPRCTGRR | 1 |
| QKELVPSTITTCCGNGTGDNVDPKCMCDNTSSPKKKKRP | 1 |
| MSNIGIVVLIFLVLFPLATAQLDADQPADLEGEKRGGWLPKMYQQMKEVLNRGTRCGGYCTDDVCCKRSFLKGMALIAAEDKRRRTAMGQ | 0 |
| MKLSVMFIVALVLSLSMTDGLPRRAENGGRIFRQHSPDSMDPQTRQIKTRTLCPEHCTNGCNMDMTCI | 0 |
| MEKLIILVLVAAVLLSTHVLVRGEGDEPADRNAVPRDNQDGMSGKFIRVLSGTCCMGYDCWCPRKPV | 0 |
| GCCSHPACSVNNPDICX | 0 |
| NGCCIVRECC | 1 |
| DYCCHRGPCMVWCX | 0 |
| ECCTHPACHVSHPELCX | 0 |
| MGMRMMFTVFLLVVLATAVLPVTLDRASDGRNAAANAKTPRLIAPFIRDYCCHRGPCMVWCG | 0 |
| NERHVTCFYVKFGCKHTECITTIVFCWQTASDISSV | 0 |
| MKLSVMFIVFLMLTMPMTGDGNNRRAANGGEVGMPAYERAAKLLALLRERQCPAPCYPNCEEC | 0 |
| VFLMLTMPMTSDANNRHAANGGEAGELAGDRAAKLMALLQERQCGKHCPTCRHC | 1 |
| KECCTHPACHVSHPELCG | 1 |
| QKELVPSTITTCCGNGTGDNVDPKCMCDNTSSPQKEEKTRPQ | 1 |
| SACCGYKLCSPCGQA | 1 |
| MFTVFLLVVLATTVVSFSTDDESDGSNEEPSADQAARSAMNRPPGCCNNPACVKHRCGG | 0 |
| TLCPEHCTNGCNMDMTCI | 1 |
| MGMRMMFTVFLLVVLATTVVSLTLDRASGGRRSGADNMIALLIIRKCCSNPACNRYNPAICD | 0 |
| MGMRMMFIVFLLVVLATTVVSFTSDRASDGGNAAAKASDLIAQTIRGGCCSYPACSVEHQDLCDGRR | 0 |
| MGMRMMFTVFLLVVLATTVVSFTSDRASDGRNAAGNEMPDWVIRAIKLTCCSELTCAGNYPNICG | 0 |
| MFTVFLLVVLATTVVSYPSDSASDGRDDETKDEKSDMYKSKRNGRCCHPACGKHFSCGR | 1 |
| MFTVFLLVVLATTVVSFTSGRASGGRNAAAKASNRIALIVRNAECCYYPPCYEAYPEICL | 0 |
| MFTVFLLVVLATTVVSFTSDRAFRGRNSAANDKRSDLAALSVRRGCCSHPACSVNHPELCGRRR | 1 |
| MFTVFLLVVLATTVVSFTSGRASGGRNAAAKASNRIAMAISSGACCAYPPCFEAYPERCL | 0 |
| MFTVFLLVVLATTVVSFTSGRAFRGRNRAADDKRSDLAALSVRGGCCSHPACAVNHPELCG | 1 |
| MMFTVFLLVVLATTVVSFTSGRASGGRNAAAKASNRIARAISSGACCAYPPCFEAYPERCL | 0 |
| ITCCTRGTCAQHC | 1 |
| VFLLVVLATTVVSSTSGRAYDGRNAAADDKAFDLLAMTIRGGCCSYPPCIASNPKCGGRR | 0 |
| MFTVFLLVVLATTVVSFTSGRAFRGRNPAANDKRSDLAALSVRGGCCSHPACGVNHPELCG | 0 |
| MGMRMMFTVFLLVVLATTVVSFSTDDESDGSNEEPSADQTARSSMNRAPGCCNNPACVKHRCG | 1 |
| MFSVFLLVVLATTVVSSTSGGASGGRKAAAKASNRIALTVRSATCCNYPPCYETYPESCL | 0 |
| PPGCCNNPACVKHRCG | 1 |
| DENGCCWNPSCPRPRCT | 1 |
| GCCHDIFCKHNNPDICX | 0 |
| CCWNRACTRLVPCSK | 1 |
| GCCTYPPCAVLSPLCD | 1 |
| MRGLPVFVILLLLIASEPSVDARPKTKADVPLTSLNDNAKRTLQILRNKRACCPYEPSCCI | 0 |
| VPVGPALAYACSVMCAKGYDTVVCTCTRRRX | 0 |
| SPAASDPLEKRIQNDLIRAALEDADVENDPRILTPIINAAKLGCCKLFDVGFCCGKK | 1 |
| ALEDADTKNDPRLVGTLVSTALTVCCQFEFLNFCC | 1 |
| SCCGSSNTGSCCX | 0 |
| GCCSTPPCAVLYCX | 0 |
| GTRCGGYCTDDVCC | 1 |
| APGCCNNPACVKHRC | 1 |
| MLSVQLITPSSHGTAHLPRDDTDAAAGEILEFLCPFFCIGGIGDEYCDCQEKRDLDLFTDQ | 0 |
| SDGRNVAAKAFHRIGRTIRDECCSNPACRVNNPHVCRRR | 1 |
| NAAANDKASDVIPLALQGCCSNPVCHVDHPELCL | 0 |
| GIWCDPPCPKGETCRGGECSDEFNSDV | 1 |
| MGMRMMFTVFLLVVLTTTVVSFPSDSASDGRDDEAKDERSDMYELKRNGRCCHPACGKHFNCGR | 1 |
| MMFTVFLLVVLTTTVVSFPSDSASDGRDDEAKDERSDMYELKRNGRCCHPACGGKYFKCGR | 1 |
| DCGRCGLGQICDAGACRPSTMM | 1 |
| GCPADCPNTCDSSNKCSPGFPX | 0 |
| NGRCCHPACGKYYSCX | 0 |
| GCPADCPNTCDSSNECSPNFP | 1 |
| MGMRMMFTVFLLVVLTTTVVSYPSDSASDGRDDEAKDERSDMYESKRNGRCCHPACGKHFSCGR | 1 |
| DAADVKPVARTNEGPGRDPAPCCQHPIETCC | 1 |
| NSEDGSPYPGPGQQPNCCKWPIVTCCN | 1 |
| MGMRMMFTLFLLVVLTTTVVSYPSDSASDGRDDEAKDERSDMYELKRNGRCCHPACGKHFNCGR | 1 |
| TNEGPGRDPAPCCQHPIETCC | 1 |
| DAADVKPVARQNEGPGRDPAPCCQHPIETCC | 1 |
| DAADVKPVARHNDGPGRDPAPCCQHPIETCC | 1 |
| HNDGPGRDPAPCCQHPIETCC | 1 |
| MGMRMMFTVFLLVVLTTTVVSFPSDSASDVRDDEAKDERSDMYKSKRNGRCCHPACGKHFSCGR | 1 |
| DGRCCHPACGKYYSCX | 0 |
| AACKAACKKGAKLILKAAAPLASQVCGPACNAALA | 0 |
| CCHPACGKYFKCX | 0 |
| MGMRMMFTLFLLVVLTTTVVSFPSDSASDGRDDEAKDERSDMYKSKRNGRCCHPACGKHFSCGR | 1 |
| MFTVFLLVVLTTTVVSFPSDSASGGRDDEAKDERSDMYELKRNGRCCHPACGGKYVKCGR | 1 |
| QGCVCNANAKFCCGEX | 0 |
| NCPAGCRSQGCCM | 1 |
| MGMRMMFTVFLLVVLTTTVVSFPSDSASDGRDDEAKDERSDMYKSKRNGRCCHPACGKHFSCGR | 1 |
| MGMRMMFTVFLLVVLTTTVVSFPSDSASDGRDDEAKDERSDIYESKRDGRCCHPACGKYYSCGR | 1 |
| GCVANCQANQTGIDCIKYCGIGI | 1 |
| DCGRCPLGQYCDAEAGMCKPTLIM | 0 |
| SDCSGMSDGTSCGDTGVCQNGLCMGAGS | 1 |
| CCHPACGKHFNC | 1 |
| MGMRMMFTVFLLVVLATTVVSFTSYRASDGRNAAAKASDLIALTVRDCCSRPPCRWSHPELCS | 0 |
| MGMRMMFTVFLLVVLTTTVVSFPSDSASDGRDDEAKDERSDIYESKRNGRCCHPACGKYYSCGR | 1 |
| QPLVVSFPSDSASDGRDDEAKDERSDIYESKRNGRCCHPACGKYYSCGR | 1 |
| CCHPACGKYYSCX | 0 |
| MFTVFLLVVLTTTVVSFPSDRASDGRDDEAKDERSDMYKSKRNGRCCHPACGGKYFKCGR | 1 |
| MFTVFLLVVLTTTVVSYPSDSASDGRDDEAKDERSDMYKSKRNGRCCHPACGKHFSCGR | 1 |
| ECCHRQLLCCLRFVX | 0 |
| SDGRDDEAIDERSDMYELKRNGRCCHPACGKHFNCGR | 1 |
| GRCCHPACGKYYSCX | 0 |
| NGRCCHPACGKHFSCX | 0 |
| DGRCCHPACGKHFSC | 1 |
| MGMRMMFTVFLLVVLATTVVSFMSGRAFRDRNAAAKVSDLIALKARRPECCSHPACNVDHPEICR | 0 |
| GDCPPWCVGARCRAGKC | 1 |
| MGMRMMFTMFLLVVLATAVVSYTSDRGPTSDGGNAAAKAFDLMALIARDDCCLVASCWQRYPERCGGGR | 0 |
| MFTVFLLVVLTTTVVSFPSDSASDVRDDEAKDERSDMYKSKRNGRCCHPACGKHFSCGR | 1 |
| MFTVGLLVVLTTTVVSFPSDSASDVRDDEAKDERSDMYKSKRNGRCCHPACGKHFSCGR | 1 |
| MGMRMMFTVFLLVVLATAAVSFTSDRAYDGGNDAAKAFDWIALTARHECCSYPACAYTHPELCGGRR | 0 |
| ECPPWCPTSHCNAGTC | 1 |
| NGRCCHPACGKHFNCX | 0 |
| HVTCFYVKFGCKHTECITTIVFCWQTASDISSV | 0 |
| ECPPRCPTSHCNAGTC | 1 |
| RDCPPWCPTSHCNAGTC | 1 |
| GRCCHPACGGKYFKCX | 0 |
| CCHPACAERYNC | 1 |
| GCCSNPVCHLEHSNLC | 0 |
| GDCPPWCVGARCRAEKC | 1 |
| NGRCCHPACGGKYVKC | 1 |
| MGMRMMFTVFLLVVLATTVVSFMSGRASHGRNAAASDLIALTIKGCCSVPPCTANHPELCG | 0 |
| GCPAECPDTCSSSGSCAPDFI | 0 |
| MGMRMMSTVFLLVVLATAVVSFTLDRASDGGDAAAKAFDLMALTARLDCCDIHACWSAHPELCGGRR | 0 |
| ASDGRDDEAKDERSDMYELKRNGRCCHPACGKYFKCGR | 1 |
| LTTTVVSFPSDSASDGRDNEAKDERSDMYELKRNGRCCHPACGKYFKCGR | 1 |
| NGRCCHPACGKHFSCX | 0 |
| GIWCDPPCPEGETCRGGECSDEFNGDMX | 0 |
| MGMRMMFTVFLLVVLATTVVSFMSGRASHGRNAAASDLIALTIKGCCSVPPCIANHPELCV | 0 |
| QCPPWCSGEPCRKGTC | 1 |
| MGMRMMFTVFLLVVLATAVVSSTSDRASDGRNTASKVFDLIALTAREDCCFIDDCWASHPEACGGGMELLRQDPLNRDMPPSA | 0 |
| MGMRMMFTMFLLVVLAIAVVSFTSDRASDGRNDAAKAFHRIALTARTGCCEYPYCAENNPELCGGRR | 0 |
| GIWCDPPCPEGETCRGGECSDEFNGDLX | 0 |
| MGMRMMFTMFLLVVLAIAVVSFTSDRASDGRNDAAKAFHRIALTARTGCCEFPYCAENIPELCGGRR | 0 |
| YSRGRIMARASECVNECVESGHNTFHCERHCSNT | 1 |
| MGMRMMFTVFLLVVLATSVVSFNSDRASHGRNAAAKVFKRIALIATRECCANPQCWSKNCR | 0 |
| RPCCSLRWCSNYCRCGI | 1 |
| MLCLPVFIILLLLASPAASNPLKTRIQSDLIRAALEDADMKNEKNILSSIMGSLGTIGNVVGNVCCSITKSCCASEE | 0 |
| MLCLPVFIILLLLASPAASNPLEKRIQSDLIRAALEDADTKNDPRLLGLVTGACCAVLKFSFCCGKK | 0 |
| MLCLPVFIILLLLASPAASNPLEKRIQNDLIRAALEDADMENDPRSIIDSVKTFCCSTFNLGICCSKK | 0 |
| LPCCYIGWCSRRCNCGGYRP | 1 |
| MLCLPVFIILLLLASPAASNPLETRIQSDLIRAALEDADMKNEKNILSSIMGSLGTIGNVVGNVCCSITKSCCASEE | 0 |
| WWEGECYDWLRQCSSPAQCCSGNCGAHSKA | 1 |
| RPCCYIGWCSRRCNCGGYRP | 1 |
| MRCLPVFIILLLLIPSASSVDAQPKPKNDLLLAPFHDNAKRTLQLLRNKRICCPWFALCCYY | 0 |
| MRCLPVFIILLLLIASAPGVDAQPKTKYNAPLTSLHDNAKGILQEHWNKRCCPRRLACCIIGRK | 0 |
| MRCLPVFVILLLLIASAPSVDARPKTKDDMPLASFHDNAKRILQILQDRNACCIVRQCC | 0 |
| MRCVPVFIILLLLSPSAPSVDAHPMTKDDVPQASLHDDAKRTLQVPWMKRGCCAMLTCCVGR | 0 |
| GMSCECCKNSCDAEEILEYCPPLPSS | 0 |
| CLAKGDVCNLIDQDCCVGICFISAHKLP | 0 |
| CEMQCEQKKKHCCRVKRRKDPMCP | 1 |
| GCSMYCWKCFPCCPN | 0 |
| AYCCHPACGPNYSCGTSCS | 1 |
| MLCLPVFIILLLLVSPAATLRVQSKLERFLTQQSPRDFAKKVMQLRYYNWIDCCFDGDCCN | 0 |
| RPCCSLRWCSNYCRCGI | 1 |
| MRCVPVFIILLLLSPSAPSVDAHPMTKDDVPQASFHDDAKRTLQVPWMKRGCCARLTCCVGR | 0 |
| GCCCNPACGPKYSC | 1 |
| MLCLPVFIILLLLVSPAATLRVQSKLERFLTQQSPRDFAKSIMQLRHDNWIDCCHDGDCC | 0 |
| MRCLPVFIILLLLILPAPSADVQPKTKDHVHLASFLDSAKRTVRGHCCPYYPQCCPSG | 0 |
| MRCLPVFVILLLLIASAPSVDAQPKTKDDIPQQASFQDNAKRYLQVLESKRNCCRLQICCGRTK | 0 |
| MRCLLVFVILLQLTASAPGVDGRPKTEDDVSLSSVSDNAKSILQGLLDKRACCVYKICYPC | 0 |
| MGMRMMFTMCLLVVLATTVISFTSDRASNGRNAAAKDKASDLNALNVRGCCSHPACRVHYPHVCYGRR | 1 |
| MRCLPVFIILLLLIPSAPSTDVQPKTKDDVPLASFRDSVKRILQRQCCPTISECCRVG | 0 |
| MLCLPVFIILLLLVSPAAPNPSETNLQSDFTLADPRDADVKNRGILELAKTVCCSATGISICC | 0 |
| MLCLPVFIILLLLASPAAPTPFETNLQSDLTRAGPRDADMKSMRGIFSTLCCGVGINSCC | 0 |
| GYCYHRYYCCSRACKLTTKRCL | 1 |
| MRCLPVFIILLVLIASAPSVDARPQTKDDALASFHDSAKRHLQRLVNARKCCPESPPCCHYYYYFGRRK | 1 |
| MRCFPVFVFLLLLIASTPRVDARRKTKENMPLAHFHDNAKRTLQIFSNILECCYSDEWCCEVDEELGLKR | 1 |
| MRCFPVFIILLLLIASAPSFDARSKTEDDVPLSSFRGNGKRILRNLRDNETVCCERGALCCFVG | 0 |
| MLCLPLFIILLLLASPAVPEPLETSLQRNLIGAALRDAGMKSDNIFLRGICCSKHPPCCSQFQ | 0 |
| MRCLPIFVILLLLIASTPSVDARPKTKDDMPLASFNDNAKRILQLLLRKRCCSIHDNSCCGLG | 0 |
| MRCLPIFVILLLLIASTPSVDARPKTKDDMPLASFNDNAKRILQLLLRQPCCSIHDISCCGLG | 0 |
| MLCLPVFIILLLLASPAAPNPFETRLLNDLARADADMETEKYLGEVRKAVCCQVFPGSDCCPGF | 0 |
| MRCFPVFIILLLLISSASNVDAQQKTKDDASLASFQDNARRTLQSLWMTRGCCPGNVLCCGK | 0 |
| MKLSVMFIVFLMLTMPMTCAGISRSATNGGEADVRAHDKAANLMALLQERMCPPLCKPSCTNCG | 0 |
| AIGPCRAAIRQYYYNWEARQCQTFIYGGCGGNDNRFETLEECEQAC | 1 |
| MMLKMGVVLFVFLVLFPLATLQLDADQPVERDAENKQDNPDERKGFVSLALRRPCCSLRWCSNYCRCGI | 0 |
| MRCLAFLVVTLLLVTAMTTAARLGPAYDGWDAAADDEASDPIVLAVRDGCCSTPPCIANHPELCG | 0 |
| MRCLPVFIILLLLIASAPGVDAQPKTKYDAPLTSLHDNAKGILQEHWNKRCCPNKPCCFIGRK | 0 |
| MKLTCMMIVAVLFLTAWTFVTADDSGNGLENLFSKAHHEMKNPKDSKLNKRCLDGGEICGICFQAAAVGGALFSSAHETTVMSSTPLCATWLDL | 0 |
| MSGHTSVNFLLLSIVALGMVATVICLCDSYISSELCEHPEETCLLPQSYVLSVESIQTGSVYVLEACRIFTKTSG | 0 |
| MRCVPVFIILLLLIASAPGVDAQPKTKYNAPLTSLHDNAKGILQEHWNKRCCPRRLACCIIGRK | 0 |
| MKLTCMVMVAVLFFTPWTFAPADDPRNGLENLFPKAPPEMKNPEAFKLNKRGPNTGELCDGVEQNCRYPYCFIVVCL | 0 |
| MRCVPVFLILLGLIASAPSVDARPQTKDDALASFHDSAKRHLQRLVNARKCCPESPPCCHYFGRRK | 0 |
| TGYKGIACECCQHYCTDQEFINYCPPVTESSSSSSSAA | 0 |
| MLCLPVFIILLLLISSASNVDAQQKTKDDASLAPFQDNARRTLQSLWMTRGCCPGNVLCCGK | 0 |
| MRCLPIFVILLLLITSTPSVDARLKAKDNMPLASFHDNAKRTLQTRLINTRCCPAQPCCRMG | 0 |
| CSTHDLSKMEIDTNLDGVYSPHRSFCTCGSGEVYFTAKDRRNHSNYRVYVCGMPTEFCTAENPVRDP | 1 |
| MRCVPVFVILLLLIASTPSVDARPNPKDDVPLASFHEDANGILQMLWKKGRSCCPSPTSCCPWGKRK | 0 |
| KGFLSSIVGNLGTVGNLVCSVCCQITKSCCPED | 0 |
| MNLTCVLIIAVLFLTACQLLTADDSRNNQKYRAVRMRDALKNLKDSRGCSARNQRCPPNAAWVWCAVVSIHPDVFDIR | 1 |
| MRCLPIFVILLLLITSTPSVDARLKAKDNMPLASFHDNAKRTLQTRLINTRCCPGQPCCRMG | 0 |
| MRCLPVFVILLLLIASAPSVDAQPKTKDDVPLAPLHDNAKSALQHLNQRCCQAFYWCCY | 0 |
| MRCFPVFVILLLLIASAPSVDARPKTKDGMPLASFHDNATRNLQILCCKHTPACCTGK | 0 |
| MLCLPVFIIVLLLIASAPSFDARSKTKDDVPLSSFRDNAKRILQTLQTKRDCCKGNPGCCGWD | 0 |
| MLCLPVFIILLLLASPAAPMPSETNLQSDFTLADPRDADVKNRGILELAKTVCCSATGISICC | 0 |
| MLCLPVFIILLLLASPAAPMPSETNLQSDFTLADPRDADVKNRGIFALAKSVCCTAAKLSFCC | 0 |
| MLCLPVFIILLLLIASTPSIDARPKTKDDMPLASFNDNAKRILQILSRKPCCSIHDSSCCGLG | 0 |
| MRCLPVFIILLLLIASAPSVDAQPKTKYNAPLTSLHDNAKGILQEHWNKRCCPRRLACCIIGRK | 0 |
| MRCVPVFIILLLLSPSAPSVDAHPKTKDDVPLASFHDDAKRTLQRLWIKALCCYGYRFCCPNFR | 0 |
| MRCVPVFIILLLLSPSAPSVDAHPKTKDDVPLASFHDDAKRTLQRLWIKALCCYGYRFCCPIFGKGK | 0 |
| MRCLPVFVILLLLIASAPSVDAQPKTKDDVPLPHLHDNIQNTLQTLRKKVCCRPMQDCCSGK | 0 |
| MRCFPVFIILLLLIASAPSFDARSKTEDDVPLSSFRGNGKRILRNLRDNKTVCCKRGTLCCFVG | 0 |
| GITCECCYNHCSFRELVQYCN | 1 |
| MKLCVVIVLLMLAMPFNGGEASRFFNQHARSQRSGMKTRGIWCDPPCPKGETCRGGECSDEFNSDVGR | 0 |
| GITCECCFNQCTYYELLQYCN | 1 |
| MHCLPVFVILLLLTASAPSVDAQPKTKDDVFLSSFDDNAKSTLRRLQYKQTCCGFKFFCCR | 0 |
| MRCVPVFIILLLLSPSAPSVDAHPKTKDDVPLASFHDDAKRTLQRLWQNTWCCRDHLRCCG | 0 |
| MRCLPVFVILLLLIASAPSVDAQLKTKDDVPLASFHANVKRTLQKLLNKRCCQIVPQCCEWVSD | 0 |
| MRCLPVFVILLLLIASAPSVDARLKTKDMPLPSSHANIKRTLQIHRNKRCCPGWELCCEWDDWW | 0 |
| MRCLPVFVILLLLIASAPSVDARPKTKDDMPLASFHDNAKRILQILQDRNGCCRAGDCCS | 0 |
| MRCLPVFVILLLLIASAPSVDAQPKTKDDIPQASFLDNAKRYLQVLESKRNCCRRQICCGRTK | 0 |
| MRCLPIFVILLLLITSTPSVDARLKAKDNMPLASFHDNAKRTLQTRLINTRCCPGQPCCRIG | 0 |
| TSDCCFYHNCCC | 1 |
| MKLCVVIVLLMLAMPFNGGEASRFFNQHARSQRSGMKTRGIWCDPPCPEGETCRGGECSDEFNGDMGR | 0 |
| MKLCVVXVLLMLAMPFNGGEASRFFNQHARSQRSGMKTRGIWCDPPCPEGETCRGGECSDEFNGDLGG | 0 |
| MRCVPVFVILLLLIASAPSVDARLKTKDDMPLPSSHANIKRTLQIHRNKRCCPGWELCCEWDEW | 0 |
| MRCLPVFVILLLLIASVPSDAVQLKTKDDMPLPSFNGNARRTPRMLSNKRICCYPNVWCCD | 0 |
| MRCVPVFVILLLLIASAPSVDARLKTKDDMPLPSSHANIKRTLQMLRNKRCCPGWELCCEWDDGW | 0 |
| MRCLPVFVVLLLLIASAPSVDAQPKTKDDVPLAPLHDNIQNTLQTLRKKVCCRPMQDCCSGK | 0 |
| MHCLPVFVILLLLTASGLSVDARPKTEDDVPLSSFRDNTKSTLQRLLKRVNCCPIDESCCS | 0 |
| MRCLPVFVILLLLIASTPSDTVPLKTKDDMPQASFHGNARRTLQMLSKKQCCWYFDISCCITV | 0 |
| MRCLPVFVILLLLIASTPSVDARAKTRDDMSLASFHDDAKRILQILQDRSGCCVIDSNCCG | 0 |
| MRCLPVFVILLLLTASAPSVDARLKTKDDVPLSSFRDNAKSTLRRLQDKQTCCGRTMCVPCG | 0 |
| MRCLPVFVILLLLIASAPSVDARLKTKDDMPLPSSHANIKRTLQMLRNKRCCPGWELCCEWDDGW | 0 |
| MRCVPVFVILLLLIASAPSVDVQLKTKDDVPLASFHANVKRTLQILLNKRCCQVMPQCCEWN | 0 |
| MRCLPVFVILLLLIASAPSVDAQLKTKDDVPLASFHANVKRTLQKLLNKRCCQIVPQCCEWN | 0 |
| MRCLPVFVILLLLIASAPSVDARPKTKDDMPLASFHDNAKRILQILQDRNGCCRAGDCCSRFEIKENDFG | 0 |
| MRCLPVLIILLLLTASAPGVVVLPRTEDDVPMSSVYGNGKSILRGILRNGVCCGYKLCHPC | 0 |
| MRCLSIFVLLVLLVSSVVAELDVEGEIVKQLLTRGTLKDADFWKRLEMQGCVCNANAKFCCGEGR | 0 |
| MRCLSIFVLLVLLVSFAVAELDVEGEIVKQLLTRGTLKDADFWKRLEMQGCVCNANAKFCCGEGR | 0 |
| MRFLLLLTVALLLTCIMETDAEAKPEDLAERFRERSDCSGMSDGTSCGDTGVCQNGLCMGAGS | 0 |
| MRCLPVFVILLLLIASAPSVDAQPKTKDDVPLAPLHDNIRSTLQTLRKKVCCRPVQDCCSGK | 0 |
| LPCCDVGWCSRRCICDVYRP | 0 |
| SDGRDDEAKDERSDMHESDRNGRGCCCNPACGPKYSCGR | 1 |
| LPCCDVGWCSRRCNCDVYRP | 1 |
| MRCVPVFIILLLLIPSAPSADAQPKTKDDVPLASYHDNAERTLQRLWNQRHCCPIDLPCCPPG | 0 |
| MLCLPVFIILLLLSPSAPSVDAHPKTKNDVHRAILHDVAKRIHLKLGCCIMFTCCMGR | 0 |
| GIVCECCKNHCNIEELTEYCPPVTEGSG | 0 |
| MLCLPVFIIPLLLVSPAATLRVQSKLERFLTQQSPRDFAKSIMQLLHYNRIDCCNYGVSDCCK | 0 |
| GIVCECCKNHCNIEELTEYCPPVTEGSG | 0 |
| MLCLPVFIILLLLSPSAPSVDAHPKTKNDVRLDILNDVAKRIDLRPGCCARLICCPGR | 0 |
| MSGLGIMVLTLLLLVLMTTSHQDAGEKQAMQRDAKNFSRRRLVIRRPKTRECEMQCEQKKKHCCRVKRRKDPMCP | 1 |
| MLCLPVFIILLLLVSPAATLRVQSKLERFLTQQSPRDFAKSVMQLLHYNWIDCCNYGVSDCCIG | 0 |
| MRCVPVFVILLLLIASAASIDAQQKTKDDAPLTSLNDNALQQHWNKRCCPRKIWCCMIPR | 0 |
| MRCVPVFIILLVLIASAPSVDARPQTKDDALASFRDSIKRHLQTLLDARECCPQSPPCCHYYYYGSWK | 0 |
| MRCVPVFVILLLLIASTPSVDATQKTKDDMSLASFHDNAKRFLQTLRNTRSCCPEEITCCPWG | 0 |
| MRCVPVFVILLLLIASTPSVDATQKTKDDMSLASFHDNAKRILQTLRNTRSCCPEEPCCFWR | 0 |
| MRCVPVFVILLLLIASTPSVDARPNPKDDVPLASFHGADNANRILRTLWNLRGCCEDKTCCFIG | 0 |
| MFTVFLLVVLATTVVSFTSDRASDGRDDEAKDERSDMHESDRKGRAYCCHPACGPNYSCGTSCSRTL | 1 |
| MLCLPVFIILLLLASPAASNPLETRIQSDLIRAALEDADMKNRKRLFLSLRGLNAIFSNLGDLGNLVSSVCCQSLLRVCCPED | 0 |
| MRCLPVFVILLLLIASAPSVDAQLKTKDDVPLSSFRGHAKSTLRRLQDKQTCCGYRMCIPCG | 0 |
| MRCLPVLVILLLLTASAPSVDALPKPKDDVPLAPLHDNAKSILQSLWNKRDCCTNLLPCCY | 0 |
| QKELVPSTITTCCGNEPGTMCPKCMCDNTSSPQKEEKTRPQ | 1 |
| MRCLPVFIILLLLVPSAVSVDVQPETKNFMTLVSRDFAKKSLKGLSNKRDCCQRNFLCC | 0 |
| MRCLPVFVILLLLIASTPSVDAQLKTKDDMSLASFHDNVKRILQIRTTEECCPFIVGCCSR | 0 |
| MRCLPVFVILLLLIASTPSDTVPLKTKDDMPQASFHGNARRTLQMLSKKQCCWYFDISCCLWP | 0 |
| MRCLALLVVTLLLFTATATTGASNGMNAAASGEAPDSISLAVRDDCCPDPACRQNHPEICPSR | 0 |
| MRCLAFLVVTLLLFTATATTGASNGMNAAASGEAPDSISLAVRDDCCPDPACRQNHPELCSTR | 0 |
| MRCLPVFVFLLLLIASTPRVDARRKTKDMPLAPFHENAKRTLHILLNKRDCCLIDEWCCEVDEEFGYK | 0 |
| MRCLPVFVILLLLIASTPRVDARRKTKDNMPLAPFQENAKSTLQLLLNKRQCCPTFSECCEMEEEFGLK | 0 |
| MKLSVTFIVVLMLTTSLTCGFSPPSNNGERAFGSHDPDAADQLVREVRASSACPEPCAKGSTCIGKTC | 0 |
| MKLSVTFIVVLMLTTSLTCGFSLPSNKEERAFGPRDPDAADQLVREERASRACNPPCTGLSMCQAGRCGYIRFR | 1 |
| MRCLPVFVILLLLIASAPSVDARMEPKDMPLASSQANVKRIQQIRQNKRICCPLMELCCEW | 0 |
| MRCLPVFVILLLLIASIPSDAVQLKTKDDMPLASFHGNGRRTQRMLSNKRFCCFPEHWCCEW | 0 |
| MRCLPIFVILLLLIASIPSDAVQLKTKDDMPLASFHGNGRRTLRMLLNKRLCCITEEWCCQWW | 0 |
| MKVSVTFIVVLMLTTSLTCGLSNGETHDPDAADQLVREERASRACNPPCTGNAMCQNGHCGYIRF | 0 |
| MRCLPVLIILLLLTASAPGVVVLPKTEDDVPMSSVYGNGKSILRGILRNGVCCGYKLCHPC | 0 |
| MRCLPIFIILLLLIASAPSVDAQPKTKDDVSLASLHAQPKTKDDVSLASLHDNIKSTLQTLWNKRCCPPVIWCCG | 0 |
| MRCLASLVVTLLLFTATATTGASNHVNAAASGKASDSISLAARDDCCPNPSCRQNHPERC | 0 |
| STDCNGVPCQFGCCVTINGMMNAGNLIVSDLTRRGLLDNEGHCPAATES | 0 |
| MRCLPVFIILLLLITSAPSVNAKLKTKDVRLLPFHDNAMRTLQRLWKKCLGCGEDPRCC | 0 |
| MLCLRVLIILLLLASPAASNPLETRIQSDLIRAALEDADIKTEKGFLGSVFSNLGGITDLATGICCAIIERCCVK | 0 |
| QHGKISSEQHTMFDPIEGCCQQSCTTCFPC | 1 |
| MRCLPVFIILLLLIPSAPSVDAQPTTKDDVPLASLHDNAKRALQMFWNKRDCCPSKLLCCNP | 0 |
| MRCLPVFIILLLLIPSAPSVDAQPTTKDDVPLASLHDNAKRALQMFWNKRDCCPAKLLCCNP | 0 |
| MRCLPVFIILLLLIPSAPSVDAQPMTKDDVPLASLHDNAKRALQMFWNKRDCCPAKLLCCNP | 0 |
| MRCLLVFIILLLLIPSAPSVDAQPMTKDDVPLSSLHDNAKRALQMFWNKRDCCPAKMFCCQW | 0 |
| CCRRGYMGCIPCCF | 0 |
| MLCLPVFIILLLLASPAAPKSLETRIQNDLIRAGLTDADLKTEKGFLSGLLNVAGSVCCKVDTSCCSN | 0 |
| RPCCSLRWCSNYCRCGI | 1 |
| MRCLPVFIILLLLIPSAPSVDAQPTTKDDVPLASLHDNAKRALQMFWNKRDCCPANLLCCNP | 0 |
| MLCLPVFIILLLLASPAAPNPFETKLQSDSTRADADXETDKNLDAVRKAICCDVFPGTDCCMG | 0 |
| MLCLPVFIILLLLASPAAPNPLERRIQSDLIRTALEDADMKTPKGVLSGIMSNLGTVGNMVGGFCCTVYSGCCSEK | 0 |
| MHCLPVLVILLLLIASTPSVDARPNPKDDVPLASFHGAVNAKRYLRTLWNSRDCCDPKEPCCFIG | 0 |
| MRCLPVFIILLLLIPSAASAAQPETKDDAALASFYDNAKRTLQRHWAKSLCCPEDAWCCSHDEGK | 0 |
| RCCPGWELCCEWDDGCK | 1 |
| MRCLPVFIILLLLIPSAPSVDAQRKTKDDVPLASFHDNAKRTLKRLWNKRSCCPQEFLCCLYLVK | 0 |
| MRCLPVFVILLLLIASTPSIDARPKTKDDMPLASFNDNAKRILQILSRKPCCSIHDNSCCGLG | 0 |
| MRCVPVFIILLLLIPSASSVDVQPLTRDDVPLASFLDDARRTLRSPWMTRRCCPGNFACCGK | 0 |
| MLCLPVFIILLLLASPAAPNPLETRIQRDLIRAALEDADMKTNERFLEGVISTIKDFAGKVCCSVSVNFCCPTA | 0 |
| GIVCECCKHHCTKEEFTEYCH | 1 |
| MRCLQVFIIFLLLIPSPPSVDAQRKTKDDVPLASFHDNAKRTLKRLWNKRSCCPREFLCCLR | 0 |
| MLCLPVFIILLLLASPAAPKSLETRIQNDLIRAGLTDADLKTEKGFLSGLLNVAGSVCCKVDTSCC | 0 |
| MHCLPVFVILLLLIASAPGVDVQPKTKNFMTRASLRDFAKKTPKRLSKLRGCCPRSFLCCR | 0 |
| MRCLPVFVILLLLIASAPGVDVQPKTKYYVPRASRRDFAKKTPKRLSKLRGCCPRSFLCCR | 0 |
| MRCLPVFVILLLLIASAPSVDARPKTKDDVPLASFHGNAERTLLNILRDGDNCCIDKQGCCPWG | 0 |
| MLCLPVFIILLLLASPAAPNPLEKRIQSDLIRAALEDADMKTDEREIVNIIDSISDVAKQICCEITVQCCVLDEE | 0 |
| MLCLPVFIILLLLASPAAPNPLEKRIQSDLIRAALEDADMKTGEREILNIIDSISDVAKQICCQITVDCCVLDEE | 0 |
| MLCLPVFIILLLLIPSASSVDVQPLTKDDVPLASFLANARRTLQSLWMTRRCCPKKPYCCPGGK | 0 |
| HCTYHRHCCDLYCNKTTNVCLET | 1 |
| CLGSREQSVRDTSCCSMSCTNNICF | 1 |
| MLCLPVFIILLLLASPAAPKSLETRIQNDLIRAGLTDADLKTEKGFLSGLLNVAGSVCCKVDTSCCSSQ | 0 |
| MLKMGVVLFIVLVLFPLATLQLDADKPVERYAENKQLLNPDERRGIILHALGQRVCCSFGSCDSLCQCRDG | 0 |
| MLCLPVFIILLLLASPAAPKSFETKVQSDLTRTDGNMETEENLGEVRKVYCCLGVRDDWCCAGQIQI | 0 |
| MLCLPVFIILLLLVSPAATMPVDLEILKAPTKESRKDFEMRIELLRSKRQCCRPANMSCCQG | 0 |
| MLKMGVVLFIVLVLFPLATLQLDADKPVERYAENKQLLNPDERRGIILHALGQRVCCPFGGCHELCLCSDG | 0 |
| MLCLPVFIILLLLASPAAPKSLETRIQNDLIRAGLTDADLKTEKGFLSGLLNVAGSVCCKVDTSCCSNQ | 0 |
| MGMRMMFTVFLLVVLATAVVSFTSDRASRGRNAAAKVFDRIALIATRECCANPQCWAKNCRGRRCYSRTL | 0 |
| MGMRMMFTVFLLVVLATAVASFTSDRASHGRNAAAKLFDRIALIATRECCADPQCWAKNCRGRRCYSRTV | 0 |
| ACSKKWEYCIVPILGFVYCCPGLNLWSFRLRLIVMSSTPICATLA | 0 |
| MLCLPVFIILLLLIPSASSVDVQPLTKDDVPLASFLDDARRTLQSLWMTRRCCPGNFACCGK | 0 |
| MRCLPVFIILLLLIPSASSVDVQPLTKDDVPLASFLDDARRTLQSLWMTRRCCPGNFACCGK | 0 |
| MRCLPVFIILLLLIPSASSVDVQPLTKDDVPLASFLANARRTLQSLWMTRRCCPKKPYCCPGGK | 0 |
| MRCVPVFVILLLLIASVPSVDAQLKTKDDMPLASSHANVKRTLQILRNKRCCITFESCCEFDLK | 0 |
| MMLKMGVVLFVFLVLFPLATLQLNADQHVKRYAENKQDNPDERKGFVSLALRRPCCSLRWCSNYCRCGI | 1 |
| MMLKLRVVLFILLVLFPLATLQLDADQPVERDAENKQDNPDERKGFVSLALRRPCCSLRWCSNYCRCGI | 0 |
| MRAVVVVTGLVVVVVTTTLSLQDHDVKSASSPLSSSVDQGSSGDDCDEGLPPPDQPFRVVWNHPDNCEKINLHLPLDEYGIIFNKRRVFLGEEIQTLYDTGPWPYINKTGCFINGGLPQLFNQPDNSETCKILGKNRIEDFTGLGVLDFETWRAIYSTNFGTMENYQIESVNLVRKRHPDYSEKELKMVAEQEWQEAARKIMTDKLAAGQSLMPRGYWGYYLYPRTWDSKPDTKFRNNKIDWLWRQSTGLYPSIYIYYDVVSKTDSVITKFVSDTVGEAVRQNDFSPPNTPIYPYVMFQTMDNVFHKEDHMKISLGLSAKMGAAGVILWGSSQNYKDFTTQCSRLQ | 1 |
| LQDHDVKSASSPLSSSVDQGSSGDDCDEGLPPPDQPFRVVWNHPDNCEKINLHLPLDEYGIIFNKRRVFLGEEIQTLYDTGPWPYINKTGCFINGGLPQLFNQPDNSETCKILGKNRIEDFTGLGVLDFETWRAIYSTNFGTMENYQIESVNLVRKRHPDYSEKELKMVAEQEWQEAARKIMTDKLAAGQSLMPRGYWGYYLYPRTWDSKPDTKFRNNKIDWLWRQSTGLYPSIYIYYDVVSKTDSVITKFVSDTVGEAVRQNDFSPPNTPIYPYVMFQTMDNVFHKEDHMKISLGLSAKMGAAGVILWGSSQNYKDFTTQCSRLQ | 1 |
| MSCLPVFVILLLLTASGPSVDARLKTKDDVPLSSFRDNAKSTLRRLQDKSACCGYKLCSPCGQA | 0 |
| TSDCCFYHNCCC | 1 |
| MRCLPVFVILLLLIASAPSVDAQPKTKDDVPLAPSHDNAKSALQHLNQRCCPTFYFCCNLSGG | 0 |
| MRCVPVFIILLLLIPSAPSVDAQRKTKDDVPLASFHDNAKRTLKRLWNKRSCCPQEFLCCLYLVK | 0 |
| MRCVPVFIILLLLASPAAPKSLETRIQNDLIRAGLTDADLKTEKGFLSGLLNVAGSVCCKVDTSCCSN | 0 |
| MRCLPVFIILLLLIPSAPSVDAQPKTEDDVPLASLHDNAKLTLQGLWDKRCCPNLFYCCPDRRK | 0 |
| MRCLPVFIILPLLIPSAPSVDAQPMTEDDVPLASFHEQTLQELWNKRPCCPLIPGCCR | 0 |
| GIVCECCKNSCTYEEITEYCPPVTEGSG | 0 |
| MRCFPVFVILLLLIASAPSVDARPKTKDGMPLASFHDNATRNLQILCCKHTPACCTGK | 0 |
| MRCLPVFVILLLLIASAPSVDARPKTKDGMPLASFHDNATRNLQILCCKHTPKCCTGK | 0 |
| MRCFPVFVILLLLIASAPSVDARPKTKDGMPLASFHDNATRNLQILCCKHTPACCK | 0 |
| MRCFPVFVTLLLLIASAPSVDARPKTKDGMPLASFHDNATRNLQILCCKHSLECCTGK | 0 |
| MRCFPVFVVLLLLIASAPSVDAQPKTKDDVPLAPLHDNIQNTLQTLRKKVCCRPMQDCCSGK | 0 |
| MMLKMGVVLFVFLVLFPLATLQLDADQPVERDAENKQDNPDERKGFVSLALRRPCCYIGWCSRRCNCGGYRP | 1 |
| MRCLPVFVILLLLIASAPGVDAQPKTKYDVPLASRHDFAKKTPKRLSKPRDCCLRHFLCCV | 0 |
| MHCLPVLVILLLLIASTPSVDARPKTKDDVPLASFHGADNANRILRTLWNLRGCCEDKTCCFIG | 0 |
| MMLKMGVVLFVFLVLFPLATLQLNADQHVKRYAENKQDLNPDERKGLVSLDLRLPCCYIGWCSRRCNCGGYRP | 0 |
| MLCLPVFIILLLLIPSASSVDVQPLTKDDVPLASFLANARRTLQSLWMTRRCCPKKPYCCPGGK | 0 |
| MLCLPVFIILLLLVSPAATLPVGSELQRDLTHESPKDLGMRTDYLPFRDVGDDCCSDNVGTCCK | 0 |
| MLCLPVFIILLLLIPSASSVDVQPLTKDDVPLASYNDNARRTLQSLSMARICCSRKLWCCGIPF | 0 |
| MLCLPVFIILLLLASPAAPKSFETKVQSDLTRTDGNMETEENLDGNMETEENLGEVRKVPCCLGVRDDWCCAGQIQI | 0 |
| MRCVPVFVILLLLIASAPTVDAQLKTKDDMPLASFHANVKRTLQILRDKRFCCRTQEVCCEAIKNG | 0 |
| GIVCECCKNHCTDEEFTEYCPHVTESG | 1 |
| MMLKMGVVLFVFLVLFPLATLQLDADQPVERYAENKQDNPDERKGFVSLALRRPCCSLRWCSNYCRCGI | 0 |
| MRCLPVFVILLLLIASAPTVDAQLKTKDDMPLASFHANVKRTLQILRDKRFCCRTQEVCCEAIKNG | 0 |
| MKLSVTFIVVLMLTTSLTCGFNLFSNNGKRAYGRHDPNAADRLVREKQASRACYPPCFGSSVCYGGRCFFIGFR | 1 |
| MLCLPVFIILLLLASPAVPEPFETKLQRDLTRADADMENLAKVRKAVCCEVLPGTDCCFG | 0 |
| MRCLPVLIILLLLTASAPGVDVLPKTEDDVPLSSVYGNGKSILRGILRKGICCGVSFCYPC | 0 |
| MRCLPVFIILLLLIPSAASVAQPKTKDDVALASFYDNAKRTLQRHWAKSLCCPEDAWCC | 0 |
| MHCLPVLVILLLLIASTPSVDARPKTKDDVPPASFHGADDANRILQTLWNLRGCCEDKTCCFIG | 0 |
| MRCLPVFIILLLLILPAPSADVQPKTKDHVHLASFLDSAKRTVRGHCCPYYPQCCPSG | 0 |
| MMLKMGVVLFVFLVLFPLATLQLNADQHVKRYAENKQDLNPDERKGLVSLDLRLPCCDVGWCSRRCICDVYRP | 0 |
| MLCLPVFIIHLLLVSPAATLAVESELQRDLTHESPKDFGMRTEHLPVKRVGDDCCVGNVGTCCS | 0 |
| MMLKMGVVLFVFLVLFPLATLQLNADQHVKRYAENKQDLNPDERKGLVSLDLRLPCCDVGWCSRRCNCDVYRP | 1 |
| METLTLLWRASSSCLLVVLSHSLLRLLGVRCLEKSGAQPNKLFRPPCCQKGPSFARHSRCVYYTQSRE | 1 |
| MRCLPVFVILLLLIASAPGVDAQPKTKYDVPLASRHDFAKKTPKRLSKPRDCCRRNFLCC | 0 |
| MLCLPVFIILLLLASPAAPKSLETRIQNDLIRAGLTDADLKTEKGFLSGLLNVADSVCCKVDTSCCSN | 0 |
| VCCPFGGCHELCLCSD | 0 |
| MRCLPVFVILLLLIASAPSVDDNAKGTQHKRIINWCCLIFYQCC | 0 |
| MRCLPVFVILLLLIASAPSVDARPKTKDDIPLVSFQDHAKRILQTFESRYDCCKTFECCHWG | 0 |
| MHCLSVFVILLLLTASAPSVDAQPKTEDDVPLSSFHDDLQRTVRTLLDIRMCCLGTSGCCPWG | 0 |
| MRCLPVFIILLLLIASAPSVDVQRKTRNSMTRASLRNFEKKSIEVLKEKGPCCFSNPYCCNLR | 0 |
| MRCLPVFIVLLLLIVSAPGFDARPKTEDDVPLSSFHDDLQRTVRTLLDIRMCCLGTSGCCPWG | 0 |
| MRCLPVFVILLLLIASAPSVDAQLKTKDDVPLASFHDNAKGTQHKRIINWCCLIFYQCC | 0 |
| MRCLPVLVILLLLIASAPSVDAQLKTKDDVPLASFHDNAKGTQHKRIINWCCLIFYQCC | 0 |
| MRCLPVFVILLLLIASASSVDDNAKGTQHRRIMAGCCPRFYQCCYPG | 0 |
| MRCLAFLVVTLLLFTAMETTGASNRVNAAANGKTSDSISLAVRDDCCPDPSCRQNHPELCA | 0 |
| MRCLPVFVILLLLIASAPGVDDFAKKTPKRLSKPRDCCRRNFLCC | 0 |
| MLCLPVFIILLLLVSPAAPMPSETNLQSDFTLADPRDADVKNRGILELAKTVCCSATGISICC | 0 |
| MLCLPVFIILLLLVSPAAPMPSETNLQSDSTLADPRDAEMKNRGIISLAKSLCCTGIGISFCC | 0 |
| MLCLPVFIILLLLASPAAPMPSETNLQSDFTLADPRDADVKNRGIFALAKSVCCTAAKLSFCC | 0 |
| MLCLPVFIILLLLASPAAPMPSETNLQSDFTLADPRDADVKNRGIHELAKSVCCTATGISICC | 0 |
| MRCLPVFVILLLLIASASSVDAQLKTKDDVPLTSVHDNAKGTQHRRIMAGCCPRFYQCCYPG | 0 |
| MRCLPVFIILLLLASTAAVDVAGSKLKRRLERKPYQGSQAYVKKTAFGLRLCCKRHHGCHPCGRT | 1 |
| MRCLPVFVILLLLIASAPSVDDNAKGTQHKRIINWCCLTFYQCC | 0 |
| TRSGGACNSHDQCCINFCRKATSTCM | 1 |
| MRCFPVFIILLLLMASAPSFDARPKTEDDVPLSSFRDNLKRTLRTLLDPRRCCYETPGCCVIG | 0 |
| MLCLPVFIILLLLVSPAATLRVQSKLERFLTQQSPRDFAKKVMQLRYYNWIDCCFDGACCN | 0 |
| MLCLPVFIILLLLASPAAPKPFETKLPSDLTRADVDIDMAVFLEKLQDACCKNAPEFGCCTR | 0 |
| MQCLPVFTILLLLASTAAPNPLETRIQSDLTRADLEDSDTKTDERFITGLLGGLSAVGGITSLASRICCAITDSCC | 0 |
| MPCLPVFIILLLLISSAPSVDARPKTKYDVSRASFHVNAKRDRQSRWMERDCCEERWCCFR | 0 |
| MLCLPVFIILLLLASPAASNPLEKRIQSDLIRAALEDADTKNDPRLLDYVTGACCAGLNFVCCGKK | 0 |
| MRCLPVFVILLLLIASTPSVNARPKTKDLASFHDNAKRTQHIFWSKRNCCIYENWCCEWI | 0 |
| MRCLPVFVILLLLIASAPSVDAQPKTKDDIPQASFLDNAKRYLQVLESKRNCCRRQICCGRPS | 0 |
| MEKLTILLLVTAVLMSTQALMQSGIEKRQRAKIKFFSKRKTTVERWWEGECYDWLRQCSSPAQCCSGNCGAHSKAG | 1 |
| MKLVLAIILVLMLTTSLTCGFNHFSNNGKRAYGPHDRSAAHRLVSEKQARSLCHPPCTGLSMCHGGMCGYIRFR | 0 |
| TRSGGACNSHNQCCDDFCSTATSTCV | 1 |
| TRSGGACNSHNQCCDDFCSTATSTCI | 1 |
| THSGGACNSHDQCCNAFCSTATRTCV | 1 |
| MRCLPVFVILLLLTASGPSVDAKVHLKTKGDGPLSSFRDNAKSTLQRLQDKSTCCGYRMCVPCG | 0 |
| MRCLPVFIILLLLIASAPSFDALPKTEDNVPLSSFHDNLKRTRRIHLNIRECCSDGWCCPAG | 0 |
| MRCLPVFVILLLLIASTPSVDALQKTKDDMPLASFHDNVKRILQTLSNKRSCCPTILSCCFV | 0 |
| MRCLPVFVILLLLIASAPSVDARPKTKDDIPLVSFQDNAKRALQILSNKRYCCYFDYSCCLYLR | 0 |
| MYCLPVFIILLLLISSAPSTPPQPRNKDRVHLISLLDNHKQILQRDWNSCCGKNPGCCPWGK | 0 |
| MRCLPVFVILLLLIASAPSVDAQLKTKDDVPLASFHDNAKGTQHKRIINWCCLIFYQCCLRR | 0 |
| MRCLPVFIILLLLASTAAVDVAGSKLKRRLERKPYQGSQAYVKKTAFGLRKCCKKHHGCHPCGRK | 1 |
| MLCLPVFIILLLLVSPAATLPVTSKLHGLLTRRSLKNFWKRNLYLRDEDWVDCCHMPRCCVED | 0 |
| MRCLPVFIILLLLVASAPSADVQPKTKNSMTLASLRDFAKKGRKNLWRRSECCIRNFLCC | 0 |
| MRCLPVFVILLLLIGSAPSVDAQPMAIDNVPVASFDDNAKRTLQILLNKRPCCPRDTWCCGFPR | 0 |
| MLCLPVFITLLLLISSAPSVDAQTKTKNFLTLASLRDFAKKSPKRLTQFECCSEVFSCCR | 0 |
| MRCLPVFVILLLLIGSAPTVDAQPKTNDVSMASFDDNAKRTLQMLRGVQICCPYILWCCLIP | 0 |
| MKLSVMFIVFLMLTMPMTCAGISRRADNGGEAGALSGDRAANIMALLQARGCPPMCNPGCHNCS | 0 |
| MLCLPVFIILPVLSSAAGFPVESELQRDLTQESPKDLGMRNEHQLLKKVADDCCVGKVGTCCR | 0 |
| MRCLPVFVILLLLIASAPSVDAQLKTKDDVPLASFHDNAKGTQHKRIINWCCLVFYQCC | 0 |
| MKLSVTIILVLMLTTSLTCGFNHFSNNGKRAYGRHDPNAADRLVREKQASRACYPPCFGSSVCYGGRCFFIGFR | 0 |
| MRCVPVFIILLLLIPSASSAAVQPKTEKDDVPLASVHDSALRILSRQCCITIPECCRIG | 0 |
| MRCVPVFIILLLLIPSAPSAAVQPKTEKDDVPLASFHDSAMRILSRQCCPTIPECCRVG | 0 |
| MRCLPVFIILLVLSASAPSVDARPKNEDDVSLASFHDNAQRTLQRLLNKRSCCPNNPACCH | 1 |
| MRCVPVFIILLLLASPAASDPLEKRIQSDLIRAALEDADTKNDPRILEDIVSTALATCCKFQFLNFCCNEK | 0 |
| CCPGWELCCEWDDGCKGNDL | 1 |
| AANNKATDLMALRYHECCKNPPCRNKHPDLCGGRRRC | 1 |
| MLCLPVFIILLLLVSPAATLLVKSKLHGFLTRRSLKDFWKRHFYLRDEDWVDCCQMGDCCDEK | 0 |
| MRCLPVIVILLLLIASAPSVDAQPKTKDDIPQASFLDNAKRYLQVLESKRNCCRRQICCGRNH | 0 |
| MYCLPVFLILLLLISSAPSTPPQPRNKDRVNLVSLLDNQKQILQRDWNGCCVKKAGCCPWGK | 0 |
| VCCSFGSCDSLCQCRD | 1 |
| MLCVPVFIILFIIIPFAPTSESQPKTKEEVAKASVHDNAERTLQRLWNQSHCCPIDLQCCPPG | 0 |
| MRCLPVIVILLLLISSAAAVVEGPLRVNRRLRPRKAPVDMQARDWNWGRCCFLSGCFECW | 0 |
| MRCLPVVVFLLLLLSAAAAPGVGSKTERLPGLTSSGDSDESLPFLNTICCWSGACCGG | 0 |
| MLCLPVFITLLLLVSPSAALPVESELQRDLTQDSPKDFRIREPLLLSKMFDRSCCGSSNTGSCCGRYQRGS | 0 |
| MYCLPVFIILLLLIPSAPSVDVQPITKNDVILDSLRNVATKPLQRLLNTRCCIKFHPCCHNG | 0 |
| MLKMGVVLFIFLVLFPLATLQLDADQPVERYAKNKQLFNPHKRRGIILRAPGKRCCHRNWCDHLCSCCGS | 0 |
| ACCEPLWCDAGCYDGCC | 1 |
| DSRGTQLHRALRKATILSVSARCKLSGYRCKRPKQCCNLSCGNYMCG | 1 |
| DECSAPGAFCLIRPGLCCSEFCFFACF | 0 |
| MLKMGVVLFIVLVLFPLATLQLDADKPVERYAENKQLLNPDERREVILHALGQRVCCSFGSCDSLCQCCDG | 0 |
| ACCDPHWCDAGCYDGCC | 1 |
| GCCDPQWCDAGCYDGCC | 1 |
| TAEDSRGTQLHRALRKATKLPVSTRCITPGTRCKVPSQCCRGPCKNGRCTPSPSEW | 1 |
| MLKMGVVLFIVLVLFPLVTLQLDADKPVERYAENKQLLNPDERRGIILHALGQRVCCSFGSCDSLCQCCDG | 0 |
| CCSQDCLVCIPCCPNGSA | 0 |
| MLKMGVVLFIFLVLFTLATLQLDADQPVERYAKNKQLFNPHKRRGIILRAPGKRCCHWNWCDHLCSCCGS | 0 |
| IVDRCCNKGNGKRGCSRWCRDHSRCCX | 0 |
| MGMRMMVTVFLLGVLATTVVSLRSNRASDGRRGIVNKLNDLVPQYWTECCGRIGPHCSRCICPEVVCPKNG | 0 |
| MGMRMMVTVFLLVVLATTVVSLRSNRASDGRRGIVNKLNDLVPKYWTECCGRIGPHCSRCICPEVACPKNG | 0 |
| MGMRMMVTVFPLVVLATTVVSLRSNRASDGRRGIVNKLNDLVPKYWTECCGRIGPHCSRCICPGVVCPKRG | 0 |
| DSCVPDGDSCLFSRIPCCGTCSSRSKSCVX | 0 |
| LVVLATTVVSFRSNRASDGRKIAVNKRRRELVVPPGKLRECCGRVGPMCPKCMCPPRRC | 1 |
| ACCDPDWCDAGCYDGCC | 1 |
| SSCGYLGQPCCVVPRRAYCHGDLECNDVTMCV | 1 |
| VGERCCKNGKRGCGRWCRDHSRCCX | 0 |
| CATYGKPCGIQNDCCNICDPARRTCT | 1 |
| MLKMGVVLFIVLVLFPLATLQLDADKPVERYAENKQLLNPDERRGIILHALGQRVCCSFGSCDSLCQCCDG | 0 |
| CKRKGSSCRRTSYDCCTGSCRNGKCX | 0 |
| MLKMGVLLFTFLVLFPLATLQLDADQPVERYADNKQDLNPDERMIFLFGGCCRMSSCQPPPVCNCCAKQDLNPDER | 0 |
| VTDRCCKGKRECGRWCRDHSRCCX | 0 |
| CCRWPCPGSCRCC | 1 |
| MLKMGVVLFIFLVLFTLPTLQLDADQPVERYAENKQLLNPDERRTIILHALTQPVCCPFGECKSLCYCCGA | 0 |
| MGVVLFIFLVLFPLATLQLDADKPVERYAENKQLLNPDERRGIILHALGQRVCCSFGSCDSLCQCCDG | 0 |
| CCSRDCWVCIPCCPNGS | 0 |
| IPYCGQTGAECYSWCIKQDLSKDWCCDFVKTIARLPPAHICSQ | 0 |
| CCSRHCWVCIPCCPNGS | 0 |
| MLKMGVVLFIFLVLFPLATLQLDADQPVERYAKNKQLFNPHKRRGIILRAPGKRCCHWNWCDHLCSCCGS | 0 |
| GCCEPFWCDSGCTEGCC | 1 |
| GLYCCQPKPNGQMMCNRWCEINSRCCGRR | 1 |
| MLKMGVVLFIFLVLFPLATLQLDADQPVERYAKNKQLFNPHKRRGIILRASGKRCCHWNWCDHLCSCCGS | 0 |
| RCCRYPCPDSCHGSCCY | 1 |
| AEDSRGTQLHRALRKTTKLSLSIRCKGPGASCIRIAYNCCKYSCRNGKCS | 1 |
| MLKMGVVLFIVLVLFPLATLQLDADKPVERYVENKQLLNPDERRGIILHALGQRVCCSFGSCDSLCQCCDG | 0 |
| CCSRDCLVCIPCCPYGSD | 0 |
| ECCEPSWCDAGCTDGCC | 0 |
| MLKMGVVLFIFLVLFPLATLQLDADQPVKRYAKNKQLFNPHKRRGIILRAPGKRCCHWNWCDHLCSCCGS | 0 |
| CCSQDCLVCIPCCPNGS | 0 |
| DCLPDYMLCAFNMGLCCSDKCMLVCLP | 0 |
| TCVMFGSMCDKEEHSICCYECDYKKGICV | 0 |
| MLKMGVVLFIVLVLFPLATLQLDADKPVERYAENKQLLNPDERRGIILHALGQRVCCSFGSCDNLCQCCDG | 0 |
| MLKMGVVLFIVLVLFPLATLQLDADQPVERYAENKQLLNPDERRGIILHALGQRVCCPFGGCHELCYCCYG | 0 |
| CCGPTACLAGCKPCC | 0 |
| CCSQDCSVCIPCCPN | 0 |
| GILLPALRKFCCDSNWCHISDCECCY | 0 |
| GILLPALRKFCCDSNWCNISDCECCY | 0 |
| WFGHEECTYWLGPCEVDDTCCSASCESKFCGLW | 1 |
| CKPPGSPCRVSSYNCCSSCKSYNKKCG | 1 |
| LPSCCSLNLRLCPVPACKRNPCCTX | 0 |
| LPPCCTPPKKHCPAPACKYKPCCKS | 1 |
| ACAEFGHSCISATCCPGVTCVEIDEPVCLWD | 0 |
| CSEVGAACDTESNICCSGECFAVQGSTFGICE | 0 |
| KTTAESWWEGECSGWSVYCTWDSECCSGECTRSYCELW | 1 |
| CAGIGSFCGLPGLVDCCSGRCFIVCLP | 1 |
| WATIDECEETCNVTFKTCCGPPGDWQCVEACPV | 0 |
| APAKRWFGHEECTYWLGPCEVDDTCCSASCESKFCGLW | 0 |
| CIVGTPCHVCRSQSKSCNGWLGKQGYCGYC | 1 |
| CCKQSCTTCMPCCW | 0 |
| MEKLTILLLVVTVLVSTQAMIQGDMEKRRKAKINIFSKRKTTAERWWDGECRSWSNKCEWPWHCCSNDCEDYCTLW | 1 |
| MEKLTILLLVVTVLVSTQAMIQGDIEKRRKAKINIFSKRKTTAGRWWDGECRSWSNKCEWPWHCCSNDCEDYCTLW | 1 |
| MEKLTILLLVVTVLVSTQAMIQGDIKKRRKAKINIFSKRKTTAERWWDGECRSWSNKCEWPWHCCSNDCEDYCTLW | 1 |
| MSGLGFMVLTLLLLTFMATSHQDRGEKQATQRHAINVIRRRLITRWATIDECEETCNVTFKTCCGPPGDWQCVEACPV | 0 |
| VLLSVALFTQMATLRAEDICQQPLMAGRCQDVYERFFFNTSSGTCEAFIWGGCDGNANNFETFEACLAVC | 0 |
| LATVALLLTLVMGIDSAPAGQTEAGRVSLREGLENTFPCNPGRCACLPASGTTTSYVCQSTQSSTADCMDNECVTEAEW | 0 |
| MEFRRLVTVTLLLTLVMSIDSVPADETETGRVSLREDDRFPCNSNQCACLPAEGSSTSYQCQSLDASTDDCFDNECVTQSEWGGRR | 1 |
| MKLTGVLIISVLLLTASQLITADYSRDKRQYLAMRLRDGLRNFRGVRDCGEQGEGCYTRPCCSGLTCVGTPVGGLCQP | 0 |
| MLLLLLLLLPLAPGEGDGQAVAGDRNPSEARSTYKRFLQRPARRIDRRDCTPCGPNLCCEPGKTCGTSTHHDHYGEPACV | 1 |
| HTLEMLLLLLLLLPLAPGEGDGQAVAGDRNPSEARSTYKRFLQRPARRIDRRDCTPCGPNLCCEPGKTCGTSTHHDHYGEPACV | 1 |
| MHTLEMMLLLLLLLPLAPGEGDGQAVAGDRNPSEARSTYKRFLQRPARRIDRRDCTPCGPNLCCEPGKTCGTSTHHDHYGEPACV | 1 |
| MLKMGVVLFIVLVLFPLATLQLDADKPVERYAENKQLLNPDERRGIILHALGQRVCCRKEWCHARCTCCG | 0 |
| MLKMGVVLFIVLVLFPLATLQLDADKPVERYAENKQLLNPDERRGIILHALGQRVCCPFGGCHEFCLCCDG | 0 |
| MKLTCMMIVAVLFLTAWTLVMADDSNNGLANHFLKSRDEMEDPEASKLEKRACSKKWEYCIVPILGFVYCCPGLNLWSFRLRLIVMSSTPICATLA | 0 |
| MRCLPVFVILLLLIASAPSVDARLKTKDDMPLPSSHANIKRTLQMLRNKRCCPGWELCCEWDDGCKGK | 0 |
| GCCPFPACTHTIICRCC | 0 |
| KCCMRPICTCPCCIGPX | 0 |
| CCQAACSPWLCLPCC | 0 |
| MHLSLARSAVLILLLLFALGNFVGVQPGQITRDADHGINLRSLRKQMSRSPLVKGAFCGQACSSVKCPKKCFCHPEEKVCYREMRTKERD | 1 |
| CCQAGCSRYMCLPCCQ | 1 |
| ALPLDGDQPADQPAERMQDISPELNPLFHPVKRGCCSPWNCIQLRACPCCPN | 1 |
| LTAIPLDGDQPADQPAERMEDGESTPNHPWFDPVKRCCTALCSRYHCLPCC | 1 |
| LLFPLSALPLDGDQPADQPAERMQDISPEQNFWFDLVERGCCTIWNCVQLPGCPCC | 0 |
| DQPVERHAGNKRHLNPTIRRAMIIDANRREKCCEVGWCDSGCECC | 1 |
| DQPVERHAENKRHLIPAVMRAMTMNADRRVQCCRIEFCDEDCGCCYH | 1 |
| RVLFRSGDQPADQPAERMQDISPEQNPLFHPDKRRCCPMPGCFAGPFCPCCPP | 1 |
| ACSKKWEYCIVPILGFVYCCPGLICGPFVCV | 0 |
| MMLKMGVVLFIFLVLFPLATLQLDADQPVERYAENKQLLNPDEKRGILLPALRKFCCDSNWCHISDCECCYG | 0 |
| MQKLTILLLVAAVLLSTQALNQEKRPKEMINFLSKGKTNAERRKRQCEDVWMPCTSNWECCSLDCEMYCTQIG | 0 |
| MHTLEMLLLLLLLLPLALGEGDGQAVAGERNPSEARSTYKRFLQRPARRIDRRECTPCGPNLCCQPGKTCGTSTHHDHYGEPACV | 1 |
| MQKLTILLLVAAVLLSTQALNQEKRPKEMINVLSKGKTNAERRKRQCEDVWMPCTSNWECCSLDCEMYCTQIG | 0 |
| MIVAVLFLTAWTFVTAVPHSSDVLENLYLKALHETENHEASKLNVRDDECEPPGDFCGFFKIGPPCCSGWCFLWCA | 0 |
| MKLTSMMIVAVLFLTAWTFVTADDSGNGLENLFSKAHHEMKNPKDSKLNKRCLDGGEICGILFPSCCSGWCIVLVCA | 0 |
| MMIVAVLFLTAWTFVTADDSGNGLENLFSKAHHEMKNPKDSKLNKRCLDGGEICGILFPSCCSGWCIVLVCA | 0 |
| MKLTWMMIVAVLFLTAWTFVTADDSGNGLENLFSKAHHEMKNPKDSKLNKRCLDGGEICGILFPSCCSGWCIVLVCA | 0 |
| MLKMGVVLFIFLVLFPLATLQLDADQPVERYAKNKQLFNPHKRRGIILRAPGKRCCHWNWCDHLCLCCGS | 0 |
| MLKMGVVLFTFLVLFPLATLQLDADQPVERYAGNKQDLNPEERRGFILHALWHVQCCSWLWCDGGCDCCE | 1 |
| MMLKMGVVLFIFLVLFPLATLQLDADQPVERYAENKQLLNPDEKRGILLPALRKFCCDSNWCNISDCECCYG | 0 |
| YWTRSACCYIEEGEKCPASCKLCC | 1 |
| CCSRNCAVCIPCCPNWPA | 0 |
| CCDDSECSYSCWPCCY | 1 |
| CCNAGFCRFGCTPCCY | 1 |
| GCCGAFACRFGCTPCC | 1 |
| GCCVKQGCWNVETCTCCPADPYIPKPFPTRST | 0 |
| CCPPVACNMGCKPCC | 0 |
| MKLTSMMIVAVLFLTAWTFVTAVPHSSDVLENLYLKALHETENHEASKLNVRDDECEPPGDFCGFFKIGPPCCSGWCFLWCA | 0 |
| SCTDDFEPCEAGFENCCSKSCFEFEDVYVCX | 0 |
| MHTLEMMLLLLLLLPLAPGEGDGQAVAGDRNPSEARSTYKRFLQRPARRIDRRECTPCGPNLCCQPGKTCGTSTHHDHYGEPACV | 1 |
| MEKLTILLLVAAVLMSTQALIQDGGEKRQKEKIKFLSKRKSVAESWWEGECSGWSNGCYWDSECCSGECTQGYCDLW | 0 |
| CSEGGDFCSKNSECCDKKCQDEGEGRGVCLIVPQNVILLH | 1 |
| MIFLFGGCCRMSSCQPPPVCNCCAKQDLNPDER | 0 |
| GLYCCQPKPNGQMMCNRWCEINSRCCX | 0 |
| MQVALGLEEGWREVRDVENLGVVLFIVLVLFPLATLQLDADKPVERYAENKQLLNPDERRGIILHALGQRVCCSFGSCDSLCQCCDG | 0 |
| ASEGCRKKGDRCGTHLCCPGLRCGSGRAGGACRPPYN | 1 |
| LNDLVPQYWTECCGRIGPHCSRCICPEVVCPKNX | 0 |
| YWTECCGRIGPHCSRCICPEVACPKNX | 0 |
| YWTECCGRIGPHCSRCICPGVVCPKRX | 0 |
| LRECCGRVGPMCPKCMCPPRRC | 1 |
| APELVVTATTTCCGYDPMTICPPCMCTHSCPPKRKPX | 0 |
| DQSCPWCGFTCCLPNYCQGLTCTVI | 0 |
| CCSRHCWVCIPCCPNX | 0 |
| CCSQDCLVCIPCCPNX | 0 |
| EGLENTFPCNPGRCACLPASGTTTSYVCQSTQSSTADCMDNECVTEAEW | 1 |
| FPCNSNQCACLPAEGSSTSYQCQSLDASTDDCFDNECVTQSEW | 1 |
| CDPPGAGCSLWESDCCSHFCILRQSGPTC | 0 |
| ECSSYDEYCFPGIRDCCSGFCFIICI | 0 |
| CDPPGDSCSRVYNDCCSNYCILRQSGPTC | 1 |
| DCGEQGEGCYTRPCCSGLTCVGTPVGGLCQP | 1 |
| DCTPCGPNLCCEPGKTCGTSTHHDHYGEPACV | 1 |
| ECTPCGPNLCCQPGKTCGTSTHHDHYGEPACV | 1 |
| DCQDSGVVCGFPKPEPHCCSGWCLFVCA | 0 |
| TVDEECNEYCEDRDQNCCGKTNGEPRCAGICL | 1 |
| KSVAESWWEGECSGWSNGCYWDSECCSGECTQGYCDLW | 1 |
| KSVVESWWEGECSGWSVYCVNDWECCSGECGGSYCELW | 1 |
| KSVAESWWEGECSGWSNGCSWDSECCSGECIAGYCDFW | 1 |
| GRCRPGGVVCGFPKPGPYCCSGWCFFVC | 1 |
| GRCVKDGDACVFPVVGSVFCCSGFCVFVCI | 0 |
| KCAAAGEACVIPIIGNVFCCKGYCLFVCIS | 0 |
| CKGPGASCIRIAYNCCKYSCRNGKCS | 1 |
| CITPGTRCKVPSQCCRGPCKNGRCTPSPSEW | 1 |
| STSCVEAGSYCRPNVKLCCGFCSPYSKICMNFPKN | 0 |
| CKLSGYRCKRPKQCCNLSCGNYMCX | 0 |
| MEKLAILLLAAAILMLTQALIQDGEKHQRMKTHFLSKRKSVVESWWEGECSGWSVYCVNDWECCSGECGGSYCELW | 0 |
| MEKLTILLLVAAVLMSTQALIQDGGEKRPKEKIKFLSKRKSVAESWWEGECSGWSNGCSWDSECCSGECIAGYCDFW | 0 |
| MNTAGRLLLLCLALGLVFESLGIPVADDVEADRDTDPDDGDPRGRNSWRSTDCNGVPCQFGCCVTINGMMNAGNLIVSDLTRRGLLDNEGHCPAATES | 0 |
| MIVAVLFLTAWTFVMADDPRDGPDTAVRGGKRFWKARNEMNSAASKLNKRECLEADYYCVLPFVGNWMCCSGICVFVCIAQKY | 0 |
| MSKLGVVLLVFLVLLALTSPLQNGNRFAGNQARKVGVQHRKNGLASALRRSSCGYLGQPCCVVPRRAYCHGDLECNDVTMCV | 1 |
| MLLLLLLLLPLAPGEGDEPVDLPALRTQDFAPEHSPWFDPVKRCCSQDCLVCIPCCPNGSA | 0 |
| RMKMMLSALRQRGCCEPFWCDSGCTEGCC | 1 |
| MLKMGVVLFIFLVLFPLATPQLDADQPLARYAENKQDLNPNERMKIMLSALRQRACCEPLWCDAGCYDGCC | 0 |
| MLKMGVVLFTFLVLFPLATLQLDADQPAARYAENKQDLNPNERKKTMLSALRQRACCDPHWCDAGCYDGCC | 0 |
| MLKMGVVLFTFLVLFPLATLQLDADQPVARYAENKQDLNPNERKEMMLSALRQRACCDPDWCDAGCYDGCC | 0 |
| MLKMGVVLFTFLVLFPLATLQLDADQPAARYAENKQDLNPNERKEMMLSALRQRACCDPDWCDAGCYDGCC | 0 |
| MLKVGVVLFTFLVLFPLATLQLDADQPAARYAENKQDLNPNERKEMMLSALRQRACCDPDWCDAGCYDGCC | 0 |
| MLKMGVVLFVFLVLFPLATLQLDADQPVARYAENKQDFNPNERMKTMLSALRQRGCCDPQWCDAGCYDGCC | 0 |
| MLKMGVVLFTFLVLFPLATLQLDADQPVARYAENKQDFNPNERMKIMLSALRQRECCEPSWCDAGCTDGCC | 0 |
| MSGLGTMVLTLLLLVFMVTSHQDGGEKQATQRNAINVGRRKSITRRTVDEECNEYCEDRDQNCCGKTNGEPRCAGICLG | 1 |
| MIMRMTLTLFVLVVMTAASASGDALTEAKRIPYCGQTGAECYSWCIKQDLSKDWCCDFVKTIARLPPAHICSQ | 0 |
| LLLTAMVIVVATGNICRLERAIGPCRAAIRQYYYNWEARQCQTFIYGGCGGNDNRFETLEECEQAC | 0 |
| MKLTILLLVAAVLMSTQALIQGGRENPQKAKMNFLKARRLPGSRQIAEKCGGWICEDTWDCCYYAECKDGYCVEI | 0 |
| WWDGECRSWSNKCEWPWHCCSNDCEDYCTLW | 1 |
| MSKLGVVLLIFVVLLALTSPHHYGNRFAGYQARQMGVQQRKNALANALRRSGCGYLGEPCCVAPKRAYCHGDLECNSVAMCVN | 0 |
| DCQESGQGCTGSPPCCPGLSCSGTHAGGMCV | 1 |
| QIAEKCGGWICEDTWDCCYYAECKDGYCVEI | 0 |
| TTSTRKCKGPLVFCLENHECCSKFCDFSDIPLRYCSTP | 1 |
| TTSTRKCKGPLVFCLENHECCSKFCDFIDIPLRYCSTP | 0 |
| TTSTRKCKGPLVFCPENHECCSKFCDFIDIPLRYCSTP | 0 |
| SCGGSCFGGCWPGCSCYARTCFRD | 1 |
| CKGPGAKCLKTMYDCCKYSCSRGRC | 1 |
| TAASASGDALTEAKRIPYCGQTGAECYSWCIKQDLSKDWCCDFVKDIRMNPPADKCP | 1 |
| MLKMGVVLFTFLVLFPLATLQLDADQPVERYVENKQDLNPDGRREIILHALGQRLCCWIHWCHARCTCCG | 0 |
| MLKMGVVLFIVLVQFPLATLQLDADKPVERYAENKQLLNPDERRGIILHALGQRVCCSFGSCDSLCQCCDG | 0 |
| MEKLTILLLVVTVLVSTQAMIQGDIEKRRKAKINIFSKRKTTAERWWDGECRSWSNKCEWPWHCCSNDCEDYCTLW | 1 |
| ASDGRNAVVHERAPELVVTATTTCCGYDPMTICPPCMCTHSCPPKRKPGRRND | 1 |
| CLDDGDDCEIGDDCCSGSCIFDEGDSFCEISYENYGGVS | 0 |
| ICRLEADVGPCSGTFPRWFYNSDMSKCQLFDYGGCRGNENRFDTEEECMELC | 1 |
| ECREKGQGCNTALCCPGLECEGQSQGGLCVDN | 1 |
| ECREKVQGCTNTALCCPGLECEGQSQGGLCVDN | 1 |
| CIPHFDPCDPIRHTCCFGLCLLIACI | 0 |
| ECREKGQGCTNTALCCPAVECEGQSQGGLCVDN | 1 |
| CLGFGEACLILYSDCCGYCVGAICL | 0 |
| DDDCEPPGNFCGMIKIGPPCCSGWCFFACA | 0 |
| KTCQRRWDFCPGSLVGVITCCGGLICFLFFCV | 0 |
| KKGVTLREDDRFPCNAGNCACLPLDSYSYTCQSPTSSTANCEGNECRSEADW | 1 |
| KKGVTLREDDRFPCNAGNCACLPLDSYSYTCQSPTSSTANCEGNECVSEADW | 1 |
| CCNWPCSFGCIPCCY | 1 |
| ERVCCGYPMSCKSRACKPSYCCX | 0 |
| TCLARDELCGASFLSNFLCCDGLCLLICV | 0 |
| FGSFIPCAHKGEPCTICCRPLRCHEEKTPTCV | 0 |
| DQCTYCGIYCCPPKFCTSSGCRSP | 1 |
| YGNFPTCSETGEDCSAMHCCRSMTCRNNICAD | 1 |
| CLAEHETCNIFTQNCCEGVCIFICVQAPE | 0 |
| AARCCTYHGSCLKEKCRRKYCCX | 0 |
| CVPHEGPCNWLTQNCCSGYNCIIFFCL | 0 |
| CCGVPNAACPPCVCNKTCGX | 0 |
| CKPPGSKCSPSMRDCCTTCISYTKRCRKYY | 1 |
| RTKRDVCELPFEEGPCFAAIRVYAYNAETGNCEQLTYGGCEGNGNRFATLEDCDNACARY | 1 |
| ECREKGQGCTNTALCCPGLECEGQSQGGCAWTLSGV | 1 |
| RTKRDVCELPFEEGPCFAAIRVYAYNAETGDCEQLTYGGCEGNGNRFATLEDCDNTCARY | 1 |
| DGECGDKDEPCCGRPDGAKVCNDPWVCILTSSRCENP | 1 |
| MSTLGMTLLILLLLLPLATPDDVGQPPKRDTLRNLLKIGTRGQGGCVPPGGGRCKANQACTKGGNPGTCGFQYDLCLCLRN | 1 |
| MSKLVILAVLVLLPLVTAEHGRDEQAMQPEKKTMWTLWSLTRRGECDGKKDCITNDDCTGCLCSDFGSYRKCA | 1 |
| GCCRFPCPDSCRSLCC | 1 |
| SKCFSPGTFCGIKPGLCCSVRCFSLFCISFE | 0 |
| CLTTGEYCWLASSCCSYSCTNNVCF | 1 |
| CLTTGEYCWLASSCCGYSCTNNVCF | 1 |
| KKGVTLREDDRTFPCSSGLCACLPLDSYSYICLSPSSSTANCENDECISEDDW | 1 |
| KKGVTQREDDRTFPCNSGRCACQPLDSYSYTCQSPSSSTANCKNNVCVSEADW | 1 |
| KRGVTLREDDRFPCNAGNCACLPLDSYSYTCLSPTSSTANCESDECVSEDDW | 1 |
| CCGVPNAACHPCVCKNTCX | 0 |
| MKLTVMMIVAVLFLTAWTFATAEDPRHGLENLFSKAHHEMKNPEDSKLDKRCIPHFDPCDPIRHTCCFGLCLLIACI | 0 |
| MCCLPVFVILLLLIASAPSVDALPKTKDDMSLASFHDNAKRTLQILSNKRYCCVYDYSCCLSWG | 0 |
| ECKGKGQGCTNTALCCPGLECEGQSQGGLCVDN | 1 |
| ECRGKGQGCTNTALCCPGLECEGQSQGGLCVDN | 1 |
| MRFLHFLIVAVLLASFMESGAMPRNPKKKRGWDTPAPCRYCQWNGPQCCVYYCSSCNYEEAREEGHYVSSHLLERQGR | 1 |
| ECREKGQGCANTALCCPGLECEGQSQGGLCVDN | 1 |
| ECREKGQGCTNTAFCCPGLECEGQSQGGLCVDN | 1 |
| VCCPPESCTDRCLCCL | 0 |
| ECRKKGQGCTNTALCCPGLECEGQSQGGLCVDN | 1 |
| ECREKGQGCTNTALCCPGLECEGRSQGGLCVDN | 1 |
| EMILPALRQWQCCTMAWCDSGCLCCE | 0 |
| ECREKGQGCTNTALCCPGLECEGQGQGGLCVDN | 1 |
| CCDEEECSSACWPCCW | 1 |
| KGQPLCCPFGGCHELCHCCGS | 1 |
| CCKYGWTCVLGCSPCGC | 1 |
| CVKYLDPCDMLRHTCCFGLCVLIACI | 0 |
| VSEACEESCEDEEKHCCHENNGVYTCLRYCW | 1 |
| TATEECEEYCEDEEKTCCGLEDGEPVCATTCL | 1 |
| CFESWVACESPKRCCSHVCLFVCA | 0 |
| KKKRGWDTPAPCHYCQWKGPQCCVYYCSSCNYEEAREEGHYVSSHLLERQGR | 1 |
| ECREKGQGCTDTALCCPGLECEGQSQGGLCVDN | 1 |
| ECREKGQGCTNTALCCPGLECEGQSQGGWCVDN | 1 |
| MSGSGVLLLTLLLLVPLSALAKECSMYYCSGGDFCCPGLKCGDPTGKKICIEPGK | 0 |
| VCREKGQGCTNTALCCPGLECEGQSQGGLCVDN | 1 |
| MGVVLFIFLVLFPLATLQLDPDQPVERYAENKQLLNPDERRGIILHALGQRVCCPPESCTDRCLCCLG | 0 |
| MLKMGVLLFTFLVLFPLATLQLDADQPVEQYAGNKQDLNPDEKREMILPALRQWQCCTMAWCDSGCLCCE | 0 |
| VKRCCDEEECSSACWPCCWG | 1 |
| DDNPGGASGKFMNVLKGQPLCCPFGGCHELCHCCGS | 1 |
| DQPAERMQDDISSEHHPFFDPVKRCCKYGWTCVLGCSPCGC | 1 |
| MSGLGIMVLTLLLLVFMETSHQDAGEKQAMQRDAINVRRRRSITRRVSEACEESCEDEEKHCCHENNGVYTCLRYCWG | 1 |
| CKPPGSKCSPSMRDCCTTCISYTKRCRKYYN | 1 |
| MSRLGIMVLTLLLLVFIVTSHQDAGEKQATQRDAINFRWRRSLIRRTATEECEEYCEDEEKTCCGLEDGEPVCATTCLG | 1 |
| MEKLTILLLVAAVLMSTQALPQGGGENRLKENIKFLLKRKTAADRGMWGDCDDWLAACTTPSQCCTEVCDGFCRLWE | 0 |
| QPADQPAERMQDDISSEHHPFFDPVKRCCKYGWTCWLGCSPCGC | 1 |
| MSGSGAMLLGLLILVAMATSLDTREICWHQSECDDPNEWCCIMGTSYGSCQPVCRP | 0 |
| MEKLTILLLVAAVLMSTQAQNQEQRQQAKINFLSKRKPSAERWRRDCTSWFGRCTVNSECCSNSCDQTYCELYAFPSFGA | 1 |
| MSGSGAMLLGLLILVAMATSLDTREICWNHSECDDPSEWCCRMGSGHGSCQPVCRP | 0 |
| MSGSGAMLLGLLILVAMATSLDTREICWNHSECDDPSEWCCRMGSGHGSCLPVCRP | 0 |
| ECREKGQSCTNTALCCPGLECEGQSQGGLCVDN | 1 |
| MQKLIILLLVAAVLMSTQALFQEKRLKEKINFLSKEKADAEKQQKRYCSDQWKSCSYPHECCRWSCNRYCA | 1 |
| CIAESEPCNIITQNCCDGKCLFFCIQIPE | 0 |
| QKDLVVTATTTCCGYNPMTMCPPCMCTNTC | 0 |
| QKELVVTATTTCCGYNPMTSCPRCMCDSSCNKKKP | 1 |
| CDAPNAPCEKFDNDCCDACMLREKQQPICAV | 1 |
| MEKLTILLLVAAVLMLTQVVIQGVGEKHENTKMNFYKARKLAGNKHSRSSDEECVGLSGYCGPWNNPPCCSWWECEVYCAVPGPSFK | 0 |
| MGILTVLLPLVAVLVLTQVIVRSDQEKPLNRRRNIREAQALRRVRDCNIQEDACESNDDCCLGLFCKNEVCEYASPGRRAVENRAALIRENKP | 1 |
| MGILTVLLPLVAVLVLTQVIVRSDQDKPLNRRRNIREAQALRRVRDCNIQEDACESNDDCCLGLFCKNEVCEYASPGRRAVENRAALIRENKP | 1 |
| MLKMGVVLFVFLVLFPLATLQLDADQPVERYVENKQDLNADERMGLILPALRQQSCCDIQWCYDNCDCCI | 0 |
| MSKLGVVLFIFLVLFTMATLQLDADQPVDRYAENKQNLNPGERRKATRRVRRDCCSLSACVPPPACECCK | 1 |
| MSKLGVVLFIFLVLFTMATLQLDADQPVDRYAENKQNLNPGERRKATRRVRRDCCSMSACVPPPACECCK | 1 |
| MLKMGVVLFIFLVLFPLATLQLDADQPVERYAENKQLLSPDERREIILHALGTRCCSWDVCDHPSCTCCG | 0 |
| MLKMGVVLFTFLVLFPLATLQLDADQPRARYAENKQDFNRNERTKMILSAVSASMGRQRRCCLWPECGGCVCCY | 1 |
| MCCLPVFIILLLLIPSASSVDVQPLTKDDVPLASFLANARRTLQSLWMTRRCCPKKPYCCPGGK | 0 |
| CLNDGDDCDTGDDCCSGLCIFDEYFSYCDDSDPYYDDYDEYYY | 1 |
| CFNDGDDCEIGDDCCSGSCIFDEYFSYCDASDPYYDDYDEYYYE | 1 |
| CTDDSQFCNPSNHDCCSGKCIDEGDNGICAIVPENS | 1 |
| MEKLTILLLVAAVLLSIQALNQEKHQRAKINLLSKRKPPAERWWRWGGCMAWFGLCSRDSECCSNSCDVTC | 0 |
| CTDSGQFCDPTNHDCCSGNCIDEGGNGVCAFVREDVPKLY | 1 |
| TCRSSGRYCRSPYDCCRRYCRRITDACV | 1 |
| CTPSGGACYVASTCCSNACNLNSNKCV | 0 |
| ECREKGQGCTNTALCCPGLECEGQSQGGLCVDN | 1 |
| DCKHQNDSCAEEGEECCSDLRCMTSGAGAICVT | 1 |
| CLDDGDDCQVGDDCCSGSCVFDEGDSFCEISYENYGGVS | 0 |
| CFNDGDDCEIGDDCCSGSCIFDEGDSFCEISYENYGGVS | 0 |
| CFNDGDDCEIGDDCCSGSCIFDEGDSFCEISY | 0 |
| SSDEECVGLSGYCGPWNNPPCCSWWECEVYCAVPGPSF | 0 |
| VRDCNIQEDACESNDDCCLGLFCKNEVCEYASP | 1 |
| MGLILPALRQQSCCDIQWCYDNCDCCI | 0 |
| DCCSLSACVPPPACECC | 0 |
| DCCSMSACVPPPACECC | 0 |
| CCDWPCGIGCIPCCLP | 0 |
| CCEADCPMCQDMLCC | 0 |
| CCSWDVCDHPSCTCC | 0 |
| GCCGAFACRFGCTPCC | 1 |
| CCLWPECGGCVCCY | 0 |
| CCSICDTTCVYPTCCDDLISD | 0 |
| WWRWGGCMAWFGLCSRDSECCSNSCDVTC | 1 |
| CNDRGGGCSQHPHCCGGTCNKLIGVCL | 1 |
| CTPRHGVCFYSYFCCSKACNPSSKRCH | 1 |
| CTPPGGSCGGHAHCCSKSCNIMASTCND | 0 |
| CLGSGEQCVRDTSCCSMSCTNNICF | 0 |
| CTPRNGYCYYRYFCCSRACNLTIKRCL | 1 |
| CTPSGGACYYDYFCCSMTCNFNSKSCV | 1 |
| CADGGDLCDPSSDNCCSECIDEGGSGVCAIVADVV | 0 |
| CIATDDFCGLPGIGWNCCTGVCIIVCVX | 0 |
| NFCHPIGEPCFPKQFIPCCSGVCDVVCRGIF | 0 |
| NFCHPVGEPCFIGQIIPCCSGVCDVVCRGVFYSP | 0 |
| MFTVFLLVVLATTLVSIPSDRASDFRNAAVHERQKELVVTATTTCCGYNPMTSCPRCMCDSSCNKKKPGRRND | 1 |
| MFTVFPLVVLATTLVSIPSDRASDGRNAVVHERQPWLVPSKITNCCGYNTMEMCPTCMCTYSCRPKKKKPGRRND | 1 |
| ASDVRNAAVHERQKDLVVTATTTCCGYNPMTMCPPCMCTNTCKKSG | 1 |
| CTDSGQFCDPTDHDCCSGNCIDEGGNGVCAFVREDVPKLY | 1 |
| DCKPEGYSCAGEEPCCEGLRCMTSGGGAICVTQ | 0 |
| ECREKGQGCTNTALCCPGLECEGRGSKELC | 1 |
| ECREKGQGCTNTALCCPGLECEGQGSKELC | 1 |
| ECREKGQGCTNTALCCPGLECEGQSQGGCAWTISGV | 1 |
| RTKRDVCELPFEEGPCFAAIRVYAYNAKTGDCEQLTYGGCEGNGNRFATLEDCDNACARY | 1 |
| FAVTFICTPPGSDCNGHSDCCSNVCSTMSYVCQENWS | 1 |
| MMLKMGVVLFVFLVLFPLATLQLDADQPVKRYVGNKQDLYPDERRGFISPALRRRRCCTWQECDGNCRCCQR | 1 |
| CTHPGGACGGHHHCCSLFCNTAANACN | 1 |
| GCTHPGGACGGHHHCCSLFCNTAANACN | 1 |
| DCTPPDGACGFHYHCCSKFCITISSTCN | 0 |
| CTPPDGACGFHYHCCSKFCITVSSTCN | 0 |
| DCTPPGGACGFYYHCCSNYCITISSTCN | 1 |
| DCTPPGGACGFYYHCCSNYCVTISSTCN | 0 |
| CTHPGGACGFYYHCCSNYCITISSTCN | 1 |
| DCTPPGGACGFYYHCCSNYCITISSTCR | 1 |
| CTPSGGACGFYYHCCSNYCITISSTCR | 1 |
| QPWLVPSKITNCCGYNTMEMCPTCMCTYSCRPKKKKP | 1 |
| CTPPGGACGVYYHCCSNYCITISSTCR | 0 |
| CTPPEGGCLSSYECCSKICWRPRCYPS | 1 |
| CTPAGDACDATTNCCILFCNLATKKCEVPTFP | 0 |
| CTHPGGACGGHSHCCSLSCNTAANSCN | 1 |
| MTMGCTHPGGACGGHYHCCSQSCNTAANSCN | 1 |
| CTHPGGACGGHHHCCSQSCNTAANSCN | 1 |
| CTHPGGACGGHHHCCSQSCNTAATTCN | 1 |
| CTHPGGACGGHHHCCSQFCNTAANACN | 1 |
| CSPPGSYCFGPAACCSNFCSTMSDVCQENWSG | 1 |
| KTCQRRWDFCPGALVGVITCCGGLICLGVMCI | 0 |
| CLGFGEVCNFFFPNCCSYCVALVCL | 1 |
| CIPQFDPCDMVRHTCCKGLCVLIACSKTA | 0 |
| CLPGLATCNLHNNKCCNYCLIFWCS | 0 |
| MLKMGVVLLIFLVLFPLATLQLDADQPVERYAKNKQLFNPHKRRGIILRAPGKRCCHWNWCDHLCSCCGS | 0 |
| CRPRGMVCGFPKPGPYCCNGWCFFVCL | 0 |
| CTVNGVVCDPGNHNCCSGSCLDDEDTPVCGIHVEIQHVHMLS | 0 |
| CKDPSDFCDPVNNNCCNGDCLNQGETSVCAIVPVIV | 0 |
| GCSDFGSDCVPATHNCCSGECFGFEDFGLCT | 1 |
| CSDIGSYCRPATHLCCSGECFGIEDMGLCNS | 0 |
| SCLAPDSWCIMDVDECCETCVLFWCT | 0 |
| GRMCVHHGNGFCTPNSPCCGELRCHAALVGYRCRY | 1 |
| QCSPNGGSCSRHFHCCSLYCNKINTGVCIAT | 1 |
| TCNTPTRYCTLHRHCCSLHCHKTIHACA | 1 |
| MLKMGVVLFIVLVLFPLATLQLDADKPVERYAENKQLFNPHKRRGIILRAPGKRCCHWNWCDHLCSCCGS | 0 |
| NCLPLGQYCGTPIEDDDLCCSQHCSVVCVSALHWL | 0 |
| GCLGLGQICEVPGLAVDDCCSGNCEFFCMPEET | 0 |
| KCALERGEVCVVPILGTLACCRAFCSGVCL | 0 |
| CLAPQRWCSMHDDSLHDDNCCKTCIILWCS | 0 |
| AVDEECNEYCEERKDKNCCGRTNGEPRCATMCF | 1 |
| CVEDGDFCGPGYEECCSGFCLYVCI | 0 |
| CLAPQRWCSMHDDNCCKTCIILWCS | 0 |
| CCDRPCSIGCVPCC | 0 |
| CCDWPCNAGCVPCCF | 0 |
| MLKMGVVLFIFLVLFPLATLQLDADQPVERYAKNKQLFNPHKRRGTILRAPGKRCCHWNWCDHLCSCCGS | 0 |
| CCVVCNAGCSGNCCP | 0 |
| CCISPACNDSCYCCQ | 0 |
| CCDWPCTIGCVPCC | 0 |
| CKQLDEDCGYGYSCCEDLSCQPLIEPDTMEITALVCQIESA | 0 |
| DCGEQGEGCATRPCCEELSCVGSRPGGLCQY | 1 |
| DCGEQGEGCATRPCCEELSCVGSRPGGLCQ | 1 |
| SCARLGEMCNYHLPCCRPWRCRASRIGTRCLNKPRYRPV | 1 |
| SCGNLHEMCNYHLPCCRPWRCRASRTGTRCLNKPRYRPV | 1 |
| SCAQLGEPCSNNPCCPGVKCFTLPTPICIWN | 0 |
| SCGNLHESCSAHRCCPGLKCIGTAHGGLCRE | 1 |
| SCGNLHESCSAHRCCPGLMCNGEASICVPY | 0 |
| SCGNLHESCSAHRCCPGLMCFTLPTPICIW | 0 |
| CLAPQRWCSMHDDNCCKTCIILRCS | 1 |
| TVTEACEESCEEEEKEEEKHCCHVNNGVPSCARICW | 1 |
| TVTEACEESCEEEEKHCCHVNNGVPSCARICW | 1 |
| TCSSSSDCPTGQECCPDKLDEPEGSCANECIIT | 1 |
| EACYPPGTFCGIKPGLCCSELCLPAVCVG | 0 |
| MLKMGVVLFIFLVLFPLATLQLDADQPVERYAKNKQLFNPHKRRGIISRAPGKRCCHWNWCDHLCSCCGS | 0 |
| AEDSRGTQLHRALRKATKLSESTRCKRKGSSCRRTSYDCCTGSCRNGKCG | 1 |
| QRLCCGFPKSCRSRQCKPHRCCX | 0 |
| HPPCCLYGKCRRYPGCSSASCCQRX | 0 |
| GPPCCLYGSCRPFPGCYNALCCRKX | 0 |
| GCCGSYPNAACHPCSCKDRPSYCGQX | 0 |
| DCCGVKLEMCHPCLCDNSCKNYGKX | 0 |
| DCCGVKLEMCHPCLCDNSCKKSGKX | 0 |
| EACYAPGTFCGIKPGLCCSEFCLPGVCFGX | 0 |
| CRIPNQKCFQHLDDCCSRKCNRFNKCVX | 0 |
| DICRMPKVVGPCRAGITRYYYDTASAACRQFIYGGCQGNLNNFGSLEACQGKCAHH | 1 |
| LCRLPAVPGPCRARQPRYFYNYKVGKCQRFNYGGCKGNTNRFLTLGECQTRC | 1 |
| EDICQQPLMAGRCQDVYERFFFNTSSGTCEAFIWGGCDGNANNFETFEACLAVC | 0 |
| VCRMPKDSGPCRASIPRWYYDANTRSCRQFVYGGCQGNGNNFESQQDCQDYC | 1 |
| HPPCCMYGRCRRYPGCSSASCCQX | 0 |
| PCKKTGRKCFPHQKDCCGRACIITICP | 1 |
| PCKKSGRKCFPHQKDCCGRACIITICP | 1 |
| PCKTPGRKCFPHQKDCCGRACIITICP | 1 |
| MGVGLFIFLVLFPLATLQLDADQPVERYAKNKQLFNPHKRRGIILRAPGKRCCHWNWCDHLCSCCGS | 0 |
| TCNTPTQYCTLHRHCCSLYCHKHPRMCIIPVSGHEPL | 0 |
| MFLFKIWVVVLFIFLVLFPLATLQLDADQPVERYAKNKQLFNPHKRRGIILRAPGKRCCHWNWCDHLCSCCGS | 0 |
| ECTPPGGACGLPTHCCGFCDMANNRCL | 0 |
| ECTPPGGACGLPTHCCGFCDTANNRCL | 1 |
| SCGEEGEGCYTRPCCPGLKCIGTAHGGLCREE | 1 |
| SCGEEGEGCYTRPCCPERLKCIGTAHGALCREE | 1 |
| CCDRPCISSCSGRCC | 1 |
| SCGEEGEGCYTRPCCSLGLKCIGIRSHGGLCREE | 1 |
| VGCGDHCLSNAHCPPVCRDCTYDSGCQM | 1 |
| AGCGGHCLDNSFCPPACSDCSEIYAC | 0 |
| RDCRPVGQYCGIPYEHNWRCCSQLCAIICVS | 0 |
| TCNTPTQYCTLHRHCCSLYCHKTIHACA | 1 |
| MFTVFLLVVLATTVVSFTSDRASDDRNTNDKASRLLSHVVRGCCGSYPNAACHPCSCKDRPSYCGQGR | 1 |
| MRCLPVFVILLLLIPSAPCVDAHPKTKDDMPLASFHDNAKGTLQRFWKKRGCCPKQMRCCTLG | 0 |
| MKLTCVFIIAVLILTACHFIVADDTGDREWRDLEWLRSLKAHDKRAGCCPTIMYKTGACRTNRCR | 0 |
| SCTGSCSSSSFCPPGCDCFHAECT | 1 |
| CCPVCISSCSGRCC | 0 |
| MLKIGSGAIHLLVLFPLATLQLDADQPVERYAKNKQLFNPHKRRGIILRAPGKRCCHWNWCDHLCSCCGS | 0 |
| MFVKLGSWLLFIFLVLFPLATLQLDADQPVERYAKNKQLFNPHKRRGIILRAPGKRCCHWNWCDHLCSCCGS | 0 |
| GLIRPSKRCIGGGDPCEFHRPYTCCSGYCIVFVCA | 0 |
| GLSRPSKRCIGGGDPCEFHRGYTCCSEHCIIWVCA | 0 |
| GLTRPSKRCLAGSAPCEFHRGYTCCSGHCLIWVCA | 0 |
| CDAPTHYCTNYWECCSGYCEHSHCW | 1 |
| GLNRPSKRCLAGSAPCEFHKRSTCCSGHCIIWWCA | 1 |
| GLNRPSKRCLAGSARCEFHKPSTCCSGHCIFWWCA | 1 |
| MSGTGVLLLTLLLLVTMATSDDACSLLNGDDCGPGELCCTPSGDHQGTCETSCW | 0 |
| MKLSVKFLLFLMILPLIAGEDMSDNDAPKSVDVQRNVKRQGQSQFGEQCTGHLDCFGDLCCFDGYCIMTSWIWPCNW | 0 |
| TVKRSSKRGWWGPPSNCWVCTGFNKCCEHESHCMTFPTQYNRECK | 1 |
| MLKMGVVLFIVLVLFPLATLQLDADKPVERYAENKQLLNPDERRGIILHALGQRVCCPFGGCHELCLCCDG | 0 |
| MKFPLLFISLALAAFLTRVQDADSSVISKEKSVRDGEEFPCAGTMADCRGLADNSVCCDTGKCIGEVCYY | 0 |
| MKFYLLLTAALLLTAVIIEAAPTDHQDEARDLMREERDDKSNCPISHPNYCSFTPVCCKHECLSNNKCSSSEFIPGQ | 0 |
| MLKMGVVLFIVLVLFPLATLQLDADKPVERYAENKQLLNPDERRGIVLHALGQRVCCPFGGCHELCLCCDG | 0 |
| MLKMGVVLFIVLVLFPLATLQLDADQPVERYAENKRLLNPDERRGIILHALGQRVCCPFGGCHELCYCCDG | 0 |
| MMYCLPVVCILLLLIPSSATFVVESRLEKDQAQSFTGDAWKRVSPIHEMIQRSQCCAVKKNCCHVG | 0 |
| GLSRPSKRCIAGGQPCEFHRGYMCCSEHCIIFVCA | 0 |
| GLGRPSRECIEGSEPCEVFRPYTCCSGHCIIFVCAR | 0 |
| KCFEVGEFCGSPMLLGSLCCYPGWCFFVCVG | 0 |
| TCLEIGEFCGKPMMVGSLCCSPGWCFFICVG | 0 |
| MTSVQAVKNSTATIIMHVSFILVMFWVAWTLVMADDSNNGLANHFLKSRDEMEDPEASKLEKRACSKKWEYCIVPILGFVYCCPGLICGPFVCV | 0 |
| MMIVAVLFLTAWTLVMADDSNNGLANHFSKSRDEMEDPEASKLEERACRKKWEYCIVPIIGFIYCCPGLICGPFVCV | 0 |
| CKAESEACNIITQNCCDGKCLFFCIQIPE | 0 |
| CFESWVACESPKRCCSHVCLFVCT | 0 |
| GCLEVDYFCGIPFVNNGLCCSGNCVFVCTPQ | 0 |
| YCSDQWKSCSYPHECCRWSCNRYCA | 1 |
| GMWGDCDDWLAACTTPSQCCTEVCDGFCRLWE | 1 |
| LTIYMLLFLTALPLDGDQPADQPAERMEDDFITEHHPLFDPVKWCCDRPCISSCSGRCC | 0 |
| CCKYGWTCLLGCSPCGC | 1 |
| GCLEVDYFCGIPFANNGLCCSGNCVFVCTPQ | 0 |
| DDDCEPPGNFCGMIKIGPPCCSGWCFFACA | 0 |
| DCTSWFGRCTVNSECCSNSCDQTYCELYAFPS | 1 |
| CCGVPNAACHPCVCTGKC | 0 |
| CCGIPNAACHPCVCTGKC | 0 |
| MQLTHVLVVGLLVLTSFQPINAVTNRVDCSAPEDKSEPGYWCGLEPLCCYSGKCFVICFGSKPAGT | 0 |
| HECCKKGFCDPGCDCCDQ | 1 |
| CCKYGWTCWLGCSPCGC | 1 |
| CNLPKIVGPCKAYMPSFFYNTGTGQCERFVYGGCGGNANRFETKQECEGQCQR | 1 |
| MMSKLGVLLTIYMLLFPLTALPLDGDQPADQPAERMEDDFITEHHPLFDPVKRCCDRPCSIGCVPCC | 0 |
| DQPADRPAERMQDDFITEQHPLFNPVKRCCDWPCTIGCVPCC | 1 |
| MHPSLARSALVMLLLLFALGVQSGQITRDVDSADLKSLRKPMTLLGSLYKRVGCGDHCLSNAHCPPVCRDCTYDSGCQM | 0 |
| GQITRDVDSADLKSLRKPMTLLGSLYKRAGCGGHCLDNSFCPPACSDCSEIYACR | 1 |
| QGAPAVCTMQKDPGPCKMAITRYYFNVAIFDCTTFSYGGCLGNGNNFENYDECYETC | 0 |
| MQKLTILLLVAAVLLSTQALNQEKRPKEMINFLSKGKTNAERRKRQCEDVWMPCTSSHWECCSLDCEMYCTQIG | 0 |
| MQKLTILLLVAAVLMSTQALYQEKRPKEMINFLSKGKTNAERRKRQCEDVWMPCTSNWECCSLDCERYCTQIG | 0 |
| MQKLTILLLVAVVLMSTQALVQSDGKQRQKAKINFLSKRKTTAESWWEGECLGWSNGCTHPSDCCSNYCKGIYCDL | 0 |
| MQKLTILLLVAAVLMSTQALVQGDGKQRQKAKINFLSKRKTTAESWWEGECLGWSNGCTHPSDCCSNYCKGIYCDL | 0 |
| MQKLTILLLVAAVLMSTQALVQSDGKQRQKAKINFLSKRKTTAESWWEGECLGWSNGCTHPSDCCSNYCKGIYCDL | 0 |
| MEKLTILLLVAAVLLSTQVLVQGDGENPLKALIDILTGRMVSGNKPKSCVPIGRPCASNEQCCTRWCTPRRIC | 0 |
| MEKLTILLLVAAVLLSTQVLVQGDEENLLKPMIYFILIGRAIPGNTPKSCDQTGEPCVLNEQCCYGWCTNHGTCY | 0 |
| MEKLTILRLVAAVLLSTQVLVQGDEENPQKALIDILTGRMVSGKKPKDCLPIGSLCHSSEQCCSGWCSPKRVC | 0 |
| MQKLTILLLVAAVLMSTQALYQEKRRKEMINFLSKGKINAERRKRGCKATWMSCSSGWECCSMSCDMYCG | 1 |
| MMIVAVLFLTAWIFATADDPRNGLENLFSKAHHEMKNPKDSKLNKRCLDAGEMCDLFNSKCCSGWCIILFCA | 0 |
| MSKLGVVLLIFVVLLALTSPHHYGNRFAGYQARQMGVQQRKNALANALRRSGCGYLGEPCCVAPKRAYCHGDLECNNVAMCVN | 0 |
| MRCLPVFVILLLLIASAPSVDARLKTKDDMPLPSSHANIKRTLQMLRNKRCCPGWELCCEWDDGCKGNDLR | 0 |
| MHLSLARPAVLMLLLLFALGNFNGVQPGQITRDVDNSVRNLQSRWEPMSLLKSLNKPRSCTGSCSSSSFCPPGCDCFHAECT | 0 |
| MSGLGIMVLTLLLLVFMATSHQDAGVKRLMLRNKLQKRTCSSSSDCPTGQECCPDKLDEPEGSCANECIIT | 0 |
| MSGLGIMVLTLLLLVFMATSHQDAGDKQAMQRDAINVRRRRSITRRRSITRRTVTEACEESCEEEEKHCCHVNNGVPSCARICWG | 1 |
| SHQDAGDKQAMQRDAINVRRRRSTTRRTVTEACEESCEEEEKHCCHVNNGVPSCARICWG | 1 |
| MLCLPVFIILLLLASPAASNPLETRIQSDLIRAALEDADMNRKGFLSSIVGNLGTVGNLVCSVCCQITKSCCPED | 0 |
| KCCMRPICMCPCCIGAG | 0 |
| MKLVLAIVLILMLVSLSTGAEESGQEISMVGPPLYIWDPIPPCKQLDEDCGYGYSCCEDLSCQPLIEPDTMEITALVCQIESA | 0 |
| RDAIRNFKDSRSCARLGEMCNYHLPCCRPWRCRASRIGTRCLNKPRYRPV | 1 |
| DKQEYSAVRSRDGMRNSGVSRSCGNLHEMCNYHLPCCRPWRCRASRTGTRCLNKPRYRPV | 1 |
| SAVRSRDGMRNSGVSRSCGNLHESCSAHRCCPGLMCFTLPTPICIW | 0 |
| EDLKLAKKCKDPSDFCDPVNNNCCNGDCLNQGETSVCAIVPVIV | 0 |
| VLFPLATLQLDADQPVERYAENKQDLNPDERMKFILHALGQRRCCISPACNDSCYCCQ | 1 |
| STDKNSKLTRQCSPNGGSCSRHFHCCSLYCNKINTGVCIAT | 1 |
| MKLTYILIVAVLSLTAGTFVTADKSRHGPDTAGGWRKLFTKARYEMNPAASTLNKRGCLGLGQICEVPGLAVDDCCSGNCEFFCMPEETR | 0 |
| DSKLDTKKCALERGEVCVVPILGTLACCRAFCSGVCL | 0 |
| MSGLGIMVLTLLLLVFMATSHQDAGDKQAMQRDAINVRRRRSITRRAVDEECNEYCEERKDKNCCGRTNGEPRCATMCFG | 1 |
| MSGLGIMVLTLLLLVFMVTSHQDAGDKQAMQRDAINVRRRRSITRRTVTEACEESCEEEEKEEEKHCCHVNNGVPSCARICWG | 1 |
| MLKMGVVLFIVLVLFPLATLQLDADQPVERYAENKQLLNPDERRGIILHALGQRVCCPFGGCHELCYCCDG | 0 |
| MLKMGVVLFIVLVLFPLATLQLDADQPVERYVENKQLLNPDERRGIILHALGQRVCCPFGGCHELCYCCDG | 0 |
| MGVVLFIVRVLFPLATLQLDADQPVERYAENKQLLNPDERRGIILHALGQRVCCPFGGCHELCYCCDG | 0 |
| RDEMENSGASPLNERNCLPLGQYCGTPIEDDDLCCSQHCSVVCVSALHWL | 1 |
| RCCPMPGCFAGPFCPCCPP | 0 |
| MMLKMGVVLFVFLVLFPLATLQLDADQPVERYVENKQDLNLDERTGYILHVLGQRTCCPPGICSSDKCSCCG | 0 |
| CCHAPYCTPPHLGCPCC | 1 |
| CRPGGMICGFPKPGPYCCSGWCFVVCL | 1 |
| EDCEAGGRFCGFPKIGEPCCSGWCFFVCA | 0 |
| GCIEDKKYCGILPFANSGVCCSYLCIFVCVPKAP | 0 |
| ECRQPGEFCFPVVAKCCGGTCLVICI | 0 |
| CLASGETCWRDTSCCSFSCTNNVCF | 1 |
| CLGSGETCWLDSSCCSFSCTNNVCF | 1 |
| SCSGSGYGCKNTPCCAGLTCRGPRQGPICL | 1 |
| DCSGSGYGCKNTPCCDGLTCRGPHQGPICL | 1 |
| KTTAESWWEGECSGWSVYCTQHSECCSGECTGNYCELF | 1 |
| STDDCSTAGCKNVPCCEGLVCTGPSQGPVCQPLA | 0 |
| KSIPESWWEGECSGWSVHCTQHSDCCSGECTGSYCELY | 1 |
| VVTETCKEYCEDRDKTCCGLENGQPDCANLCL | 1 |
| SITRTEACYEYCKEQNKTCCGISNGRPICVGGCI | 1 |
| EVVTEECEEYCKEQNKTCCGLTNGRPRCVGVCF | 1 |
| WWEGECRGWSNGCTTNSDCCSNNCDGTFCKLW | 1 |
| WWEGDCTGWLDGCTSPAECCTAVCDATCKLW | 1 |
| KTTAESWWEGECRTWYAPCNFPSQCCSEVCSSKTGRCLTW | 1 |
| GVDCVGLSSYCGPWNNPPCCSWYTCDYYCKF | 1 |
| VVTEACEEYCEDRDKKTCCGLENGEPFCATLCF | 1 |
| DVDCVGWSSYCGPWNNPPCCSWYTCDYYCKL | 1 |
| CCDPDWCDAGCYDGCC | 1 |
| GCCEPDWCDSGCDDGCC | 1 |
| DCLGAGQFCAAHSWIGVGCCNSFCIVIC | 0 |
| FCLDQGEFCGWVIFSEDCCSGFCLFACI | 0 |
| ECCELEWCDGACDCCD | 1 |
| MLKLGMVLFIFLVLFPLATLHLDADQPVERNVNKQGLKPNERRFRFSAPRKRECCEWEWCDGACDCCN | 1 |
| MLKMGVVLFIFLVLFPLATLQLDADQPVERYAENKQLLNTDERREIILSALRRQCCDSNSCEYPKCLCCNG | 0 |
| MLKMGVVLFTFLVLFPLATLQLDADQPVERYAENKQDINPDERKAFITLALGQEGCCPSGPCHFAACNPPCCT | 0 |
| MLKMGVVLFIFLVLFPLATLQLDADQPVERYAENKQLLNTDERREIILSALRTRVCCPFGGCHELCQCCEG | 0 |
| MLKMGVVPFTFLVLFPLATLQLDADQPVERNAENKQDINPDERRGFITLALRHRGCCPIGPCMQSVCSPCCP | 0 |
| RAILHAPREQECCEPQWCDGGCDACC | 1 |
| MLKMGVVLFTFLVLFPLATLQLDADQPVERNAENKQDINPDERRGFITLALRHRGCCPIGPCLQSVCSPCCP | 0 |
| MLKMGVVLFTFLVLFPLATLQLDADQPVERYAENKQGLNPDERMKFILHALGQRRCCISPACNDTCYCCQDR | 0 |
| MLKLGMVLFIFLVLLPLATLHLDADQPVERNVNKQGLKPDERRRFRFSAPRKRECCELEWCDGACDCCD | 1 |
| CYDGGTSCNTGNQCCSGWCIFLCL | 1 |
| CTQSGELCDVIDPDCCNNFCIIFFCI | 0 |
| WCKQSGEMCNLLDQNCCEGYCIVLVCT | 0 |
| QCCTGSCLNCWPCC | 0 |
| CCSQDCSVCIPCCPPP | 0 |
| CCSQDCRVCIPCCPY | 0 |
| MLKMGVVLFIFLVLFPLATLQLDADQPVERYAENKQLLSPDERREIILHALGTRCCSWDVCDHPSCTCCG | 0 |
| SCIEKGDFCGWVILSESCCTGFCFYVCI | 0 |
| DCVAGGHFCGFPKIGGPCCSGWCFFVCA | 1 |
| EDCIAVGQLCVFWNIGRPCCSGLCVFACTVKLPX | 0 |
| TVDCGGVPCEFGCCRIIDGKEKCREIDCD | 0 |
| CLAKGDFCNLITQDCCDGICFIFCP | 0 |
| CQRANFVCDAFHHAAVCCEGVCVLVCAW | 0 |
| DCGEQGQGCYTRPCCPGLHCAAGATGGGSCQP | 1 |
| DCGGQGKGCYTQPCCPGLRCRGGGTGGGVCQP | 1 |
| DCGGQGEGCYTQPCCPGLRCRGGGTGGGVCQL | 1 |
| DCGGQGEGCYTQPCCPGLRCRGGGTGGGSCQP | 1 |
| WWDGECRLWSNGCRKHKECCSNHCKGIYCDIW | 1 |
| DCVEVDYFCGIPFVFNGLCCSGNCVFVCTPQ | 0 |
| CDGWSTYCHDDSCCSTCANSYCTLIKGV | 0 |
| TCCVRPWCDGACDCCVDP | 0 |
| RCCPMEWCDGDCFCCV | 0 |
| QCCGWEWCDDICGCCE | 0 |
| QINCCPWPCPDSCHYQCCH | 1 |
| RCCVHPACHDDCICCIT | 0 |
| RCCISPACHDECICCID | 0 |
| QCCDRNSCEYPKCLCCN | 1 |
| VCCPFGGCHELCQCCE | 0 |
| CCSWDVCDRPSCACC | 1 |
| QDCAAGGQFCGFPKIGGPCCSGWCLGVCA | 1 |
| GCTWWFGRCAEDGECCSNSCDQTYCELYAFPS | 1 |
| QCPYCVVHCCPPSYCQASGCRPP | 0 |
| MLKMGVMLFIFLVLFPLATLQLDADQPVERYAENKRLMSPDERRAILHAPRQRGCCEPDWCDSGCDDGCC | 0 |
| MLKMGVVLFIFLVLFTLATLQLDADQPVERYAENKRLMSPYKRRAILHAPREQECCEPQWCDGGCDACC | 0 |
| MLKMGVVLFIFLVLFTLATLQLDADQPVERYAENKQLLSPDERRDIILHALGQRRCCDPDWCDAGCYDGCC | 0 |
| MEKLTILLLVAAVLMWTQALIQEKRPKEKIKFLSKRKTTAESWWEGECSGWSVYCTQHSECCSGECTGNYCELF | 0 |
| MQKLTILLLVAAVLMSTQALIKGGGEKRPKEKIKFLSKRKTTAESWWEGECSGWSVYCTQHSECCSGECTGNYCELF | 0 |
| MSGLGIMVLTLLLLVSMATSHQDGGGKQATQRDAINVRRRRSITRRVVTEACEEYCEDRDKKTCCGLENGEPFCATLCFG | 1 |
| MEKLTILLLVAAVLMSTQALIQEKRPKEKIKFLSKRKSIPESWWEGECSGWSVHCTQHSDCCSGECTGSYCELY | 0 |
| MSGLGIMVLALLLLVFMATSHQDGGGKQATQRDAINVRRRRSITRRVVTETCKEYCEDRDKTCCGLENGQPDCANLCLG | 1 |
| CRTVGRPCHYSAQCCSDFCAHTISNYYYIKRCS | 1 |
| MSGLGIMVLTLLLLVSMATSHQDGRGKQATQRDAINVRRRRSITRTEACYEYCKEQNKTCCGISNGRPICVGGCIR | 1 |
| MSGLGIMVLTLLLLVSMATSHQDGGGKQATQRDAINVRRRRSITRREVVTEECEEYCKEQNKTCCGLTNGRPRCVGVCFG | 1 |
| MQKLTILLLVAAVLMSTQALIKGGGEKRPKEKIRFLSKRKTTAERWWEGECRGWSNGCTTNSDCCSNNCDGTFCKLW | 1 |
| MQKLTILLLVAAVLMSTQALIKGGGEKRPKEKIKFLSKRRTNAERWWEGDCTGWLDGCTSPAECCTAVCDATCKLW | 1 |
| MQKLTILLLVAAVLMSTQALIRGGVEKRQEAKRNFFSKRKTTAESWWEGECRTWYAPCNFPSQCCSEVCSSKTGRCLTW | 1 |
| MKLTILFLVAAVLMSTQALIQHDGEKSQKAKMKFLTARTLSAKTRGVDCVGLSSYCGPWNNPPCCSWYTCDYYCKF | 1 |
| MEKLTILLLVAAVLMSTQALIQHDGEKSQKAKMKFLTARTLSAKTRGVDCVGLSSYCGPWNNPPCCSWYTCDYYCKF | 1 |
| MKLTILFLVAAVLMSTQALIQHDGEKSQKAKMKFLTARTLSAKKRDVDCVGWSSYCGPWNNPPCCSWYTCDYYCKL | 0 |
| SCNNSCQQHSQCASHCVCLLNKCRTVN | 1 |
| MSGLEIMVLTLLLLVSMATSHQDGGEKQATQRDAINVRRRSITRTEACYEYCKEQNKTCCGISNGRPICVGGCIR | 1 |
| CLDQGSFCGTLVFGVACCSGFCIIVCL | 0 |
| QGCCPPGVCQMAACNPPPCCP | 0 |
| RCCISPACNDTCYCCQD | 1 |
| CCDWPCTIGCVPCC | 0 |
| MGFILSALRQQECCVYPFCDGGCYTCQDSCE | 0 |
| QCADLGEECHTRFCCPGLRCEDLQVPTCLMA | 1 |
| QCLPPLSLCNMADDDCCNDCVLFLCSYY | 0 |
| QCLPPLHWCNMVDDECCHFCVLLACV | 0 |
| QCLPPLSLCTMDDDECCDDCXLFLCLVTS | 0 |
| QCLPPLSLCTMDDDECCDDCILFLCLVTS | 0 |
| NTEEGCLPPLSLCTMADDECCHDCILFLCLVSP | 0 |
| MLKMEVVLFTFLVLFPLSTLQLETDQPVERYVENKQDLNPDESRNFMLPIVKKCCTACRMPPCKCCA | 0 |
| DCRALGEYCGLPYVHNSRCCSQLCGFICVPESP | 0 |
| VAEECEESCEDEEKHCCNTNNGPSCAPQCF | 1 |
| VTEECEENCEEEEKHCCNTNNGPSCAPQCF | 1 |
| VSEACEESCEEEEKHCCHENNGVYTCLRYCW | 1 |
| TATEECEEYCEDEEKTCCGEEDGEPVCARFCL | 1 |
| TATEECEEYCEDEEKTCCGLEDGEPVCATTCL | 1 |
| AAAEECEEYCEEEEKTCCGEEDGEPVCAEFCL | 1 |
| AATEECEEYCEDEEKTCCGLEDGEPVCATTCL | 1 |
| TATEECEESCEEEEKTCCGEXDGEPVCARFCL | 0 |
| VPEECEESCEEEEKTCCGLENGQPFCSRICW | 1 |
| VCVDGGTFCGFPKIGGPCCSGWCIFVCL | 1 |
| MSRLGIMVLTLLLLVYMATSHQDAGEKQATQRDAINFRWKRSLTRRTATEECEESCEEEEKTCCGEXDGEPVCARFCLG | 0 |
| MSRLGIMVLTLLLLVFIVTSHQDAGEKQATKRAAVNFRWRRSFTRRAAAEECEEYCEEEEKTCCGEEDGEPVCAEFCLG | 1 |
| CTHAYEACDATTNCCYMTCNLPTRKCRGPLF | 1 |
| CTPPSGYCYHPYYCCSRACNLTRKRCL | 1 |
| FKVTLSCTPPGTYCVGPSTCCSDVCSMSNVCQ | 0 |
| LFERYAENKQDLSPDERMGFILSALRQQECCVYPFCDGGCYTCQDSCE | 1 |
| MGMRMMFTVFLFVVLATTGVSFTLDRASDGWNAAAKESDVIALTRRKCCSIPKCYKNNKKCVADDAGAHSCHQNNQDMCG | 1 |
| MKLTSALIVAVLFLTAWIFVTADDSRNELENRFSKAHHEMKNPEASELNKRNCFPNGKFCGFPKVGKPCCSGVCLFACT | 0 |
| MKLTSALIVAVLFLTAWIFVTADDSRNELENRFSKAHHEMKNPEASELNKRGCFPSGSFCGLPKLAMQCCSGVCAFVCV | 0 |
| MKLTSALIVAVLFLTAWIFVTADDSRNELENRFSKAHHEMKNPEASELNKRNCFPNGKFCGLPKVGKPCCSGVCVFACV | 0 |
| MSRLGIMVLTLLLLVFIVTSHQDAGEKQATQRNAINFRWRRSFTRRAATEECEEYCEDEEKTCCGLEDGEPVCATTCLG | 1 |
| LTLSAPKGKCNGAPCTFGCCRSVNGKPRCNEFDC | 1 |
| MKTVAVFLVVALAVAYGQFFCPSSEDESLNCIETMGTTATCMKSNRGETYSYACGYCGKKKETCFGDKSQ | 1 |
| RRSLTRRVPEECEESCEEEEKTCCGLENGQPFCSRICWG | 1 |
| MSGLGILVLTLLLLVYMATSHQDAGEKQATQRDAINVRRRRSLTRRVAEECEESCEDEEKHCCNTNNGPSCAPQCFG | 1 |
| MSGLGIMLLTLLLLVFMETSHQDAGEKQATQRDAINVRRRRSLTRRVTEECEENCEEEEKHCCNTNNGPSCAPQCFG | 1 |
| MSGLGIMVLTLLLLVFMETSHQDAGEKQAMQRDAINVRRRRSITRRVSEACEESCEEEEKHCCHENNGVYTCLRYCWG | 1 |
| MSRLGIMVLTLLLLVFIVTSHQDAGEKQATQRAAINFRWKRSLTRRTATEECEEYCEDEEKTCCGEEDGEPVCARFCLG | 1 |
| MSRLGIMVLTLLLLVFIVTSHQDAGEKQATHRGAINFRWRRSLIRRTATEECEEYCEDEEKTCCGLEDGEPVCATTCLG | 1 |
| DGPADCCDMKTCCVDKMRTCLQRHTFNEKTWFPVVITKPATYVDLITKSWAVAMDIEHAC | 0 |
| MKTVAVFLVVALAVAYGQFFCPSSEDESLNCIETMGTTATCMKSNRGETYSYACGYCGKKKETCFGAKSQ | 0 |
| CTVDSDFCDPDNHDCCSGRCIDEGGSGVCAIVPVLN | 0 |
| QCSPNGGSCSRHYHCCSLWCNKDSGVCVATSYP | 1 |
| QCTPVGGSCSRHYHCCSLYCNKNIGQCLATSYP | 1 |
| RHGCCKGPKGCSSRECRPQHCCX | 0 |
| CLSPGSSCSPTSYNCCRSCNPYSRKC | 1 |
| SCSGRDSRCPPVCCMGLMCSRGKCVSIYGE | 0 |
| CRCEQTCGTCVPCC | 1 |
| ECCEWEWCDGACDCCN | 1 |
| CCSQDCWECIPCCPN | 0 |
| CCSRRCWVCIPCCPNGS | 0 |
| CCSQDCSVCIPCCPN | 0 |
| CCRLACGLGCHPCCX | 0 |
| QCCDSNSCEYPKCLCCN | 1 |
| VCCPFGGCHELCQCCE | 0 |
| GCCPIGPCMQSVCSPCCP | 0 |
| GCCPIGPCLQSVCSPCCP | 0 |
| GCCHLLACRMGCSPCCW | 0 |
| EIILHALGTRCCSWDVCDHPSCTCCX | 0 |
| CCILCWKCTYNCCRAW | 0 |
| CCDLPCNAGCVPCC | 0 |
| CCDDSECSYSCWPCCY | 1 |
| KAFITLALGQEGCCPSGPCHFAACNPPCCT | 0 |
| CCSALCSRYHCLPCC | 0 |
| CCTACRMPPCKCCA | 0 |
| QFFCPSSEDESLNCIETMGTTATCMKSNRGETYSYACGYCGKKKETCFGDKSQ | 1 |
| ATLRNPSLCSLLPDTGSCRAAFHMFYFDQFSKECKVFIYGGCDGNANRFLNSKACYKTCGN | 1 |
| QCTPVGGYCSRHYHCCSNHCIKSIGRCVAH | 1 |
| QCTPVGGYCFDHHHCCSNHCIKSIGRCVAH | 0 |
| QCTPVGGYCSRHHHCCSNHCIKSIGRCVAH | 1 |
| CSNFGSDCIPATHDCCSGECFGFEDMGLCT | 0 |
| SCGNLGESCSAHRCCPGLMCMGEASICIPY | 0 |
| NCGEQGEGCATRPCCAGLSCVGSRPGGLCQYD | 1 |
| QCADLGEECYTRFCCPGLRCKDLQVPTCLLA | 1 |
| CLEKGVLCDPSAGNCCSGECVLVCL | 0 |
| HGCCKGPEGCSSRECRPQHCC | 1 |
| KCCSIPKCYKNNKKCVADDAGAHSCHQNNQDMC | 1 |
| QVSWWCGKPEATCGKLYLKCCSGMCNKANWKCL | 1 |
| GCSARNQRCPPQCCMGLVCGREYPPRCV | 1 |
| QVSWWCGKPEATCGKLYLKCCSGRCNKANWKCLR | 1 |
| SRSCSGRDSRCPPVCCMGLMCSRGKCVSIYGEK | 0 |
| NCFPNGKFCGFPKVGKPCCSGVCLFACT | 0 |
| GCFPSGSFCGLPKLAMQCCSGVCAFVCV | 0 |
| NCFPNGKFCGLPKVGKPCCSGVCVFACV | 0 |
| QFFCPSSEDESLNCIETMGTTATCMKSNRGETYSYACGYCGKKKETCFGAKSQ | 1 |
| RCCTWQECDGNCRCCQ | 1 |
| CCTVCTTGCVVCC | 0 |
| GCCDYDWCDEFCYCCE | 1 |
| CCKYGWTCWLGCSPCCX | 0 |
| MQKLTILLLVTAVLLSTQAMIEDGGEKRPKEKIIFLSKGKRNAGRWKRDCKWWPYNCSGKEECCSGYCVGYCL | 1 |
| MEKLTILLLVAAVLMSTQAMFQGDGEKSRKAEINFSETRKLARTKQKRCDGWSTYCEVDSECCSEQCVNSYCTLFG | 1 |
| MQKLIILLLVAAVLMSTQALFQEKRPKEKIDLLSKRKTDAEKQQKRYCSDDWQPCSHFYDCCKWSCNNGYCP | 1 |
| MEKLTILLLVAAVLMSIQALNQEQHQRAKINLLSKRKPPAERWWWGGCTWWFGRCSTDSECCSNSCDQTYCELYRFPSRY | 1 |
| MEKLTILLLVAAVLLSTQVLVQGDVDKPQKATINFFTARMLLRNKPSMCVQKGDRCIRKKNCCSIWCTIINGRAKCFKKKN | 0 |
| MKLTCMMIVAVLFLTAWTFATADDSSNGLENLFSKAHHKMKNPEASKLNNRCLAKGDVCNLIDQDCCVGICFISAHKLP | 0 |
| MKVSSVLIVAMLTLTAGQLISASSHYSEDVQISPSVRSADEVENSENVKLSKRKCMEQGTYCSLILFSSSCCGDLCLFGFCIL | 0 |
| MEKLTILLLVAAVLMSIQAVNQEKHQRAKMNLLSKRKPPAERWWRWGGCMAWFGLCSKDSECCSNSCDVTRCELMPFPPDW | 0 |
| MEKLTILLFVAVVLMSTQALPQGGGEKRPRENIRFLSKRKSNAERWREGNCTPWLATCTNAPQCCTGVCYKRAYCALWE | 1 |
| MEKLTILLLVAAVLMSTQALMQEQRQKAKINLFSKRRLSAESWWEENGCSLWGPCTVNAECCSGDCDETCIFGS | 0 |
| MHQSLARSAVLMLLLLFALGNFVVVQSGLITRDVDNGQLTDNRRNLQSEWKPVSLFMSRRTCHKPCQRHSECASHCICLIDTCDDAG | 1 |
| MHQSLTRSAVLMLLLLFALGNFVVVQSGLITRDVDNGQLTDNRRNLQTEWNPLSLFMSRRSCNNSCQNHSDCASHCICTFRGCGAVNG | 1 |
| VLIIAVLFLTARQLTTAETSSRGKQKHRALRSTDKNSRMTKRCTPAGDACDATTECCILFCNLATKKCQVPTFP | 1 |
| VLVVMTAASASGDALTEAKRIPYCGQTGAECYSWCIKQDLSKDWCCDFVKTIARLPPAHICSQ | 0 |
| MSGLGIMVLTLLLLVFMVTSHQDSGEKRATQRNAINVRRRRSIIRRTVDEACNEYCEERNKNCCGRTDGEPVCAQACLG | 1 |
| MKLTWMMIVAVLFLTAWTFVTADDTRYKLENPFLKARNELQKLEASQLNERGCLDPGYFCGTPFLGAYCCGGICLIVCIET | 0 |
| VLIIAVLFLTACQLTTAETSSRGKQKHRALRSTDKNSKLTRGRTPPGGACGGHAHCCSQSCNILASTCNA | 1 |
| IPYCGQTGAECYSWCIKQDLSKDWCCDFVKTIARLPPAHICSQ | 0 |
| MEKLTILLLVAAVLMSTQALMQEQRQKAKINLFSKRKPSAERRWVDVGCTFLLGSCTADAECCSDNCVETYCDLWW | 0 |
| DCGGQGEGCYTQPCCPGLRCRGGGTGGGVCQL | 1 |
| MENLIILLLVAAVLTSTQALIQGREERQKAKINFLSKRKSNWERWWEGDCRTWDAPCNPAVECCFGVCRHRRCVLW | 1 |
| MEKLTILLLVAAVLMSTQALIQEQRQKAKINLFSKRKPSAERRWGSSECTYFFGPCTVDAECCSNSCDETYGYCEKWS | 1 |
| QPWLVPSKITNCCGYNNMEMCPTCMCTYSCRP | 0 |
| QKELVVTATTTCCGYNPMTSCPRCMCDSSCNKKKP | 1 |
| MRCLPVFVILLLLIASTPNVDAQLKTKDDMPQASFHDNAKQDQQIRLRTSDCCFYHNCCC | 0 |
| MLKMGVVLFIFLVLFPLATLQLDADQPVERYAENKQLLNPDERREIILHALGTRCCSWDVCDHPSCTCCGG | 0 |
| MLKMGMVLFIFLLLFPLATLQLDADQPVERYAENKQLLNPDERRGILLPALKQRVCCPFGDCHEICYCCGG | 0 |
| MLKMGVVLFTFLVLFPLATLQLNADRPVERYAANKQDLNPDETREMILHVLGQRLCCWSEMCHARCKCCG | 0 |
| MLKMGVLLFTFLVLFPLATLQLDADRPVERYAANKQDLNPEERRKFILHALGQWQCCTMQWCDKACYCCE | 0 |
| VIIIAVLFLTACQLIATASYARSERKHPDLRLSSRNSKISKRCLGSREQSVRDTSCCSMSCTNNICF | 1 |
| MEKLTILFLVAAVLLSTQVLVQGGETPLEAKINFYRSRLLSRFDPRLCGQAGDPCGSDAQCCSGSCYGGSYCR | 1 |
| MRCFPVFIILLLLIASAPCFDARTKTDDDVPLSSLRDNQKRTIRTRLNIRECCEDGWCCTAAPLTAP | 0 |
| MEKLTILLLVAAVLMSTQALIQEQRQKAKINLFSKRKPSAERWWGENDCRVFGSCTADEECCFNNCVQAYCFFV | 0 |
| MQKLIILLLVAAVLMSTQALFQEKRPMKKINFLSKGKTDAEKQRKRSCSDDWQYCEYPHDCCSWSCDVVCS | 0 |
| MEKLTILLLVAAVLMSIQALNQEQHQRAKINLLSKRKAPAERWWRWGGCLLWFGRCTKDSECCSDSCDRTYCELARFPEGW | 1 |
| MEKLTILLLVAAVLMSTQAMFQGGGEKRPKDKIKFLSKRKTNAESWWEGECLGWSNGCTQPSDCCSNNCKGRNCDIW | 1 |
| MEKLTILLHVAAVLMSTQALIQEQRQKAKINLFSKRKPSAERWWGDNGCSLWGSCTVDAECCLGNCGGMYCSLLR | 0 |
| MQKLIILLLVAAVLMSTQALFQEKRPKEKIDLLSKRKTDAEKQQKRYCSDDSQPCSHFYDCCKWSCNNGYCP | 1 |
| MQKLIILLLVAAVLMSTQALFQEKRRKEKIDLLSKRKTDAEKQHKRLCPDYTDPCSNAYECCSWNCHNGHCTG | 1 |
| MEKLTILLLVAAVLTSTQALIQGGGDERQKANINFLSRWDRECRAWYAPCSPGAQCCSLLMCSKATSRCILAL | 0 |
| MEKLTILLLVAAVLTSTQALIQGGADERQKAKINFLSRSDRECRGYNAPCSAGAPCCSWWTCSTQTSRCF | 1 |
| DCGEQGQGCYIYPCCPGLTCLGGGTGGGVCQPQ | 1 |
| DCGGQGEGCYTQPCCPGLRCRGGGTGGGACQL | 1 |
| DCGEQGQGCYTRPCCPGLHCAAGATGGGSCQP | 1 |
| QQCCPPVACNMGCEPCC | 0 |
| CCGPTACLAGCKPCC | 0 |
| QCCDSNSCEYPKCLCCN | 1 |
| GCCHLLACRMGCSPCCW | 0 |
| GCCHPSTCHVRKGCSRCCS | 1 |
| CCSWDVCDHPSCTCC | 0 |
| SCCNAGFCRFGCTPCCY | 1 |
| CCMTCFGCTPCC | 0 |
| GILLPALRKFCCDSNWCNISDCECCY | 0 |
| RCCISPACNDTCYCCQD | 1 |
| CTPRHGVCFYSYFCCSKACNPSSKRCL | 1 |
| CCHWPYCAPPPLGCRCC | 1 |
| GCTPPGGACGGHAHCCSQSCNILASTCNA | 1 |
| GCTPPGGACGGHAHCCSQSCDILASTCNA | 1 |
| MDAQTTLRVWGLVVLATAVVLSAPLHDNQVQLPVHSLEKRECPVSGGRRPFFHCMIACMTTSTDYLCRHHYCKDCAKGNQNGRMKSQVP | 1 |
| ECPVSGGRRPFFHCMIACMTTSTDYLCRHHYCKDCAKGNQNGRMKSQVP | 1 |
| MNMRMTLAVLILVALTTTVVLSTSRRKDEARKIPCMNQCVGQARKPAHQCCEICTDTGTWDCSK | 0 |
| KIPCMNQCVGQARKPAHQCCEICTDTGTWDCS | 1 |
| MTKHCTPPEVGCLFAYECCSKICWRPRCYPS | 0 |
| ECCEWEWCDGACDCCN | 1 |
| CTPPGGYCYHPDPCCSQVCNFPRKHCL | 1 |
| CTPPGGYCYHPDPCCSQYCNFPRKHCL | 1 |
| TVDEACNEYCEERNKNCCGRTDGEPVCAQACLX | 0 |
| DCGGQGEGCYTQPCCPGLRCRGGGTGGGSCQP | 1 |
| ECRRRGQGCTQSTPCCDGLRCDGQRQGGMCVDS | 1 |
| GCLDPGYFCGTPFLGAYCCGGICLIVCIET | 1 |
| CLGSRELCVRDTSCCSMSCTNNICF | 0 |
| CLGSREQCVRDTSCCSMSCTNNICF | 1 |
| CLGSGELCVRDTSCCSMSCTNNICF | 0 |
| MLKLGMVLFIFLVLFPLATLHLDADQPVERNVNKQGLKPNERRFRFSAPRKRECCEWEWCDGACDCCN | 1 |
| MLKMGVMLFIFLVLFPLATLQLDADQPVERYAENKQLLNPDEKRGILLPALRKFCCDSNWCNISDCECCYG | 0 |
| MLKMGVMLFIFLVLFPLATLQLDADQPVERYAENKQLLNTDERREIILSALRRQCCDSNSCEYPKCLCCNG | 0 |
| MLKMGVMLFIFLVLFPLATLQLDADQPVERYAENKQLLSPDERREIILHALGTRCCSWDVCDHPSCTCCG | 0 |
| MMLKMGVVLFTFLVLFPLATLQLDADQPVERYAENKQGLNPDERMKFILHALGQRRCCISPACNDTCYCCQDR | 0 |
| CTPGGEACDATTNCCFLTCNLATNKCRSPNFP | 1 |
| CTPAGGACDATTECCILFCNLATKKCQVPTFP | 0 |
| CTPAGDACDATTECCILFCNLATKECQVPAFP | 0 |
| CTPAGDACDATTECCILFCNLATKKCQVPTFP | 0 |
| CTPAGDACDATTKCCIPFCNLATKKCQVPTFP | 0 |
| CTAPGGACYADNTCCSNACNLNTKKCVLS | 1 |
| CTAPGGACYAAYTCCSNACNLNTKKCVLS | 1 |
| APWMVVTATTNCCGYTGPACHPCLCTQSCX | 0 |
| CTSPDGACNTPPQCCSKYCISISTTCN | 0 |
| QKELVVTATTTCCGYNPMTSCPRCMCDSSCNKKKKX | 0 |
| DECFSPGTFCGIKPGLCCSAWCYSFFCLTLTF | 0 |
| CCMTCFGCTPCC | 0 |
| VCCPFGDCHEICYCCG | 0 |
| LCCWSEMCHARCKCC | 1 |
| KFILHALGQWQCCTMQWCDKACYCCE | 0 |
| GCCGPTACIVGCKPCC | 0 |
| GCCHPSTCHMRKGCSRCCS | 1 |
| GCCNAGFCRFGCTPCCW | 1 |
| QHVSSEQHPLFDAVKGCCTHPCTLGCTPCCY | 1 |
| CCSWDVCDHPSCTCCG | 0 |
| CCDDSECDYNCWPCCIF | 0 |
| SDFRNAAVHERQKELVVTATTTCCGYNPMTSCPRCMCDSSCNKKKKPGRRND | 1 |
| FDGRNAAVNERAPWMVVTATTNCCGYTGPACHPCLCTQSCG | 1 |
| MFTVFLLVVLATTLVSIPSDRASDGRNAVVHERQPWLVPSKITNCCGYNNMEMCPTCMCTYSCRPKKKKPGHRND | 1 |
| MFTVFLLVVLATTLVSIPSDRASDFRNAAVHERQKELVVTATTTCCGYNPMTSCPRCMCDSSCNKKKPGRRND | 1 |
| ECRGYNAPCSAGAPCCSWWTCSTQTSRCF | 1 |
| WWGENDCRVFGSCTADEECCFNNCVQAYCFFV | 1 |
| SCSDDWQYCEYPHDCCSWSCDVVCS | 1 |
| WGGCLLWFGRCTKDSECCSDSCDRTYCELARFPEGW | 1 |
| SDVRNAAVHERQKELVPSKITTCCGYSPGTACPSCMCTNTCKKKNKKPGRRND | 1 |
| TNAESWWEGECLGWSNGCTQPSDCCSNNCKGRNCDIW | 1 |
| STGKRNAGKLTVTDDVEADRDTDPDDKDPSVHNSWRTVDCGGVPCEFGCCRIIDGKEKCREIDCD | 1 |
| CKQAGESCDIFSQNCCVGTCAFICIEX | 0 |
| WIDPSHYCCCGGGCTDDCVNC | 0 |
| CNNRGGGCSQHPHCCSGTCNKTFGVCL | 1 |
| CTDDSQFCDPNDHDCCSGECIDEGGRGVCAIVPEHV | 1 |
| GCCMPLSCMLLCEPCCX | 0 |
| MKKLTILLLIAAVLMLTQALIQEKRPEDEIKFLSKRKSGAQRWWDGECRLWSNGCRKHKECCSNHCKGIYCDIW | 1 |
| MLKMGVVLFIFLVLFPLATLQLDADQPVERYAENKQLLSPDERREIILHALGTRCCSWDVCDRPSCACCG | 0 |
| MLKMGVVLFVFLVLFPMATLQLDADQPVERYAENKQDHNADERMGYILPALRQRTCCVRPWCDGACDCCVDP | 0 |
| MLKMGVVLFVFLVLFPMATLQLDADQPVERYAENKQDLNADERMGLILPALRQRTCCVRPWCDGACDCCVDP | 0 |
| MEKLTILLLVAAILMSTQALNQEQRQQAKINLLSKKKPSAERWRRGCTWWFGRCAEDGECCSNSCDQTYCELYAFPSRAI | 1 |
| MLKMGVVLFVFLVLFPLATLQLDADQPVERYAENKQDLNADERMGYILPALSQQTCCVRPWCDGACDCCVDS | 0 |
| MLKMGVVLFVFLVLFPLATFQLDADQPVKRYAKYKQDLNTDKRMRLILPALRQRQCCGWEWCDDICGCCE | 0 |
| MFKMGVVLFTYLVLFPLATFQLDADQPVERYVENIQDLNPDERMEFILHALGQRRCCVHPACHDDCICCITR | 0 |
| MFKMGVVLFTYLVLFPLATFQLDADQPVERYAENKQDLNPDERMKFKLHNLGQRRCCISPACHDECICCIDR | 0 |
| MLKMGVMLFIFLVLFPLATLHLDADQPVERYAENKQLLNTDERREVILSALRRQCCDRNSCEYPKCLCCNG | 0 |
| MMLKMGVVLFIFLVLFPLATLQLDADQPVERYAENKQLLNTDERREIILSALRTRVCCPFGGCHELCQCCEG | 0 |
| MMLKMGVVLFVFLVLFPPATLQLDADQPVERYVGNKQDLNPDERRGFISPALRRRRCCTWQECDGNCRCCQR | 1 |
| GCCGAWACMAGCRPCC | 1 |
| GCCPALACAMGCRPCCX | 0 |
| MLKMGVVLFTFLVLFPLATLQLDADQHVERNAENKQDLIPDKRRVILLRALRQRRCCPMEWCDGDCFCCV | 0 |
| WWGDNGCSLWGSCTVDAECCLGNCGGMYCSLL | 1 |
| YCSDDSQPCSHFYDCCKWSCNNGYCP | 1 |
| LCPDYTDPCSNAYECCSWNCHNGHCTX | 0 |
| CRLGAESCDVISQNCCQGTCVFFCLP | 0 |
| CYDGGTGCDSGNQCCSGWCIFVCL | 1 |
| WGGCMAWFGLCSKDSECCSNSCDVTRCELMPFPPDW | 0 |
| LSAESWWEENGCSLWGPCTVNAECCSGDCDETCIFGS | 1 |
| WVDVGCTFLLGSCTADAECCSDNCVETYCDLWW | 0 |
| AVYGDCGGERCRFGCCKTDDGEEKCQHFGCP | 1 |
| NVNCGGVPCKFGCCREDRCREIDCD | 1 |
| NCPYCVVYCCPPAYCQASGCRPP | 0 |
| CTQSSEFCDVIDPDCCSGVCMAFFCI | 0 |
| QCPYCVVHCCPPSYCQASGCRPP | 0 |
| SCNNSCQNHSDCASHCICTFRGCGAVN | 1 |
| CLSGGEVCDFLFPKCCNYCILLFCS | 0 |
| GKPCHEEGQLCDPFLQNCCLGWNCVFVCI | 0 |
| CIPFLHPCTFFFPDCCNSICAQFICL | 0 |
| ATDCIEAGNYCGPTVMKICCGFCSPFSKICMNYPQN | 0 |
| CKGKGASCSRTMYNCCTGSCNRGKC | 1 |
| YECYSTGTFCGVNGGLCCSNLCLFFVCLFS | 1 |
| DECYPPGTFCGIKPGLCCSERCFPFVCLSLEF | 0 |
| TCHKPCQRHSECASHCICLIDTCDDA | 1 |
| YDCEPPGNFCGMIKVGPPCCSGWCFFACA | 0 |
| CVPYEGACNWLTQNCCDAVCVVFFCL | 0 |
| CIEYLEPCDFLRHTCCVGVCLLMACI | 0 |
| ECRAWYAPCSPGAQCCSLLMCSKATSRCILAL | 0 |
| WGSSECTYFFGPCTVDAECCSNSCDETYGYCEKWS | 1 |
| LCGQAGDPCGSDAQCCSGSCYGGSYC | 1 |
| WWEGDCRTWDAPCNPAVECCFGVCRHRRCVLW | 1 |
| WREGNCTPWLATCTNAPQCCTGVCYKRAYCALWE | 1 |
| DCKWWPYNCSGKEECCSGYCVGYCL | 1 |
| CDGWSTYCEVDSECCSEQCVNSYCTLF | 1 |
| YCSDDWQPCSHFYDCCKWSCNNGYCP | 1 |
| WWWGGCTWWFGRCSTDSECCSNSCDQTYCELYRFPSRY | 1 |
| PSMCVQKGDRCIRKKNCCSIWCTIINGRAKCF | 1 |
| SCTPPGGPCGYYNNCCSHQCNINRNKCE | 1 |
| CMEPDRRCSEWSPERCCTKCDYYRSICV | 1 |
| CYGFGEACLVLYTDCCGYCVLAVCL | 1 |
| DCQALWDYCPVPFLSSGDCCIGLICGPFICIGW | 0 |
| DCQDKWEFCIVPILGFVYCCPGLICGPFVCV | 0 |
| ASRLNKSCHLGGEYCGLFEVCCYGDCFIMCW | 0 |
| CYDSWTACDSPKLCCSGWCLFVCV | 0 |
| GKPCHKEGQLCDPFLQNCCLGWNCVFVCI | 0 |
| KCMEQGTYCSLILFSSSCCGDLCLFGFCIL | 0 |
| QKELVPSKITTCCGYSPGTACPSCMCTNTCKKKNKKPX | 0 |
| MTMGCTHPGGACAGHHHCCSQSCNTAANSCN | 1 |
| ECTPPGGACYYHSQCCGDFCQRYINSCL | 1 |
| ECTPPEGACNHPSHCCEDFCDRGRNRCM | 1 |
| ATDCIEAGNYCGPTVMKICCGFCSPYSKICMNYPKN | 0 |
| STSCMEAGSYCGSTTRICCGYCAYFGKKCIDYPSN | 1 |
| CRSSGSPCGVTSICCGRCYRGKCTX | 0 |
| CRPSGSPCGVTSICCGRCYRGKCT | 1 |
| CKLKGQSCRKTSYDCCSGSCGRSGKCX | 0 |
| DGCSSGGTFCGIHPGLCCSEFCFLWCITFID | 1 |
| VVIVAVLLLTACQLITAEDSRGTQKHRTLRSTARRSKSELTTRRRSSGSPCGVTSICCGRCYRGKCT | 1 |
| SDGRDDEAKDERSDMHESDRNGRGCCCNPACGPNYGCGTSCSRPSEPRR | 1 |
| CKAAGKPCSRIAYNCCTGSCRSGKCX | 0 |
| HNDLIRAGLTVCLSENRKRLTCSGLLNMAGSVCCKVDTSCCSSQ | 1 |
| MLKMGVVLFTFLVLFPLATLQLDADQPVERYVENKQDLNPDERSNFRLPLVRRCCSVSICQPPPVCECCA | 0 |
| MLKMGVVLFIFLVLFPLATLQLDADQPVERYAENKQLLNPDEKRGILLPALRKFCCDSNWCNISDCECCYG | 0 |
| MLKMGVVLFTFLVLFPLATLQLDADQPVERYVENKQDLNPDERSNFRLPLVRRCCSVSICQSPPVCECCA | 0 |
| MLKLGMVLFIFLVLFPLATLHLDADQPVERYAEDKQDLNRDERMGFILHALGQRQCCDWPWCDDCICCD | 0 |
| MLKMGVMLFTFLVLFPLATLQLDADQPVERYAEDKQDLNRDERMGFILHALGQRQCCDWPWCDDCICCD | 0 |
| MFKMGVVLFTYLVLFPLATFQLDADQPVERYAENKQDLNPDERMKFKLHNLGQRRCCISPACHDDCICCITR | 0 |
| MLKMGVMLFIFLVLFPLATLHLDADQPVERYAENKQLLNTDERREIILSALRRQCCDSNSCEYPKCLCCNG | 0 |
| MLKMRVVLFTFLVLFPLATLQLDADQPVERNAENKQDLIPDKRRVILLRALRQWRCCPKEWCNRDCSCCT | 0 |
| MLKLGMVLFIFLVLLPLATLHLDADQPVERNANKQGLKPDERRRFRFSAPRKRECCELEWCDGACDCCD | 1 |
| MLKMRVLLFTFLVLFPLVTLQLDADQPVERYAENKQDLNPDERREITLHALGKRCCPARMCMAACSCCD | 1 |
| QKELVPSVITTCCGYDPGTMCPPCRCTNSCPTKPKKPX | 0 |
| QNCCNGGCSSKWCKGHARCCX | 0 |
| CRCEQTCGTCVPCC | 1 |
| CCDDSECSYSCWPCCY | 1 |
| RCCISPACHEECYCCQ | 1 |
| QKELVPSKTTTCCGYSPGTMCPSCMCTNTCPPQK | 1 |
| QKGLVPSVITTCCGYDPGTMCPPCRCTNSCPKKPKKP | 1 |
| CKAAGKSCSRIAYNCCTGSCRSGKC | 1 |
| CTPDDGACAEPVQCCSTFCNPVTNMCIDWLGIGLSRSVL | 0 |
| CRTWNAPCSFTSQCCFGKCAHHRCIAW | 1 |
| QKSLVPSVITTCCGYDPGTMCPPCRCTNSCX | 0 |
| VDEECNEICGEQGKNCCGRSNGTPRCAKVCF | 1 |
| SITGRVVTEACEEACEQEEKNCCGITNGQPFCNFPCI | 1 |
| ACRKKWEYCIVPIIGFIYCCPGLICGPFVCV | 0 |
| CKGKGAPCRKTMYDCCSGSCGRRGKC | 1 |
| CMEAGSYCGSTTRICCGYCAYSASKNVCDYPSN | 1 |
| DGCSNAGGFCGIHPGLCCSEICLVWCT | 1 |
| MEKLTILLLVAAVLMSTQALIQSDGEKRQQAKINFLSXRKSTAESWWEGECKGWSVYCSWDWECCSGECTRYYCELW | 0 |
| GCCCNPACGPNYGCGTSCS | 1 |
| QNCCNGGCSSKWCRDHARCCX | 0 |
| SVIQRKATEECEEVCEQDEKHCCDGSDGTPRCTSRCF | 1 |
| MLKMGVVLFTFLVLFPLATLQLDADQPVERYAENKQDLNPNERMKMIMSALGQRRCCISPACHEECYCCQ | 0 |
| MGMRMMFTVFLSVVLATTVVSTPSDRASDGRNAAVHERQKGLVPSVITTCCGYDPGTMCPPCRCTNSCPKKPKKPGRRND | 1 |
| MRCLPVFIILLLLIASAPCFDARTKTDDDVPLSPLRDNLKRTIRTRLNIRECCEDGWCCTAAPLTGR | 0 |
| ADVPCGDGTCTFGCCENGICKELNCLDVSNTESSW | 0 |
| CIGLSNYCGPWNNPPCCSRWICESRYCDFPNVLS | 1 |
| KPSVMSWWGSKCNIFLMGCKVHADCCSNNCEGHCRLW | 1 |
| CKPPGSKCTPTVYNCCTSCNPYSHKCRTPW | 1 |
| TWEGKEVQPCKGPGSWCGGEEVPTECCEVCVFGWCT | 1 |
| LTRSCDPPGQECERLENNCCHACKIREKNPNVCSNE | 1 |
| DCSPSGDGCHTRTCCPGLRCSGPQQAQVCH | 1 |
| LTRSCDPPGYECERLENNCCDACKIRENNPNVCSNE | 1 |
| VSCGEYCGDYGDCPSSCPTCTSNLLKCM | 1 |
| CSGNPCSTERKCCKGYFCGEGKCLSRQRGTFRN | 1 |
| SSQSTCPYCQISCCPPAYCQPSGCRGP | 1 |
| DCGERDEPCCVNSSGVKYCESPWSCMHTTLLCEQN | 1 |
| MRIISFALKQWGCCDTGWCDAGCGCCV | 0 |
| DCCKEEWCDGGCYCCV | 1 |
| CCPWPCNNGCEPCC | 1 |
| TCCTACNIPPCKCCA | 0 |
| CAGIGSFCGLPGLVDCCSDRCFIVCLP | 0 |
| MLKMGVVLFIFLVLFPLATLQLDADQPVERYAENKQLLNPDEKRGILLPALRKFCCDSNWCNISDCECCYG | 0 |
| NACELDSSTGDDCTGTQICCNEPGSMSGECKETDECPD | 1 |
| RGCCNGRGGCSSRWCRDHARCCX | 0 |
| QKCCTGKKGSCSGRACKNLRCCAX | 0 |
| RCCTGKKGSCSGRACKNLKCCAX | 0 |
| MEKLTILLLVAAVLMSTQALIQGGLDERQKAKSNFFSKRKSNAESWWEGECRTWNAPCSFTSQCCFGKCAHHRCIAW | 1 |
| MSGLGIMVLTLLLLVFMATSRQDAGEKQATQRDAISVIGRRSIIRRRVDEECNEICGEQGKNCCGRSNGTPRCAKVCFG | 1 |
| MSGLGIMVLTLLLLVFMATSHQDAGEKQATQRDAISVIGRRSIIRRRVDEECNEICGEQGKNCCGRSNGTPRCAKVCFG | 1 |
| MSGLGIMVLTLLLLVFMATSRQDAGEKQATQRDAIKVIRRSVIQRKATEECEEVCEQDEKHCCDGSDGTPRCTSRCFG | 1 |
| MSGLGIMVLTLLLLVIMATSHQDAGEKQMTQRDAINVRRRRSITGRVVTEACEEACEQEEKNCCGITNGQPFCNFPCIG | 0 |
| KSTSCMEAGSYCGSTTRICCGYCAYSASKNVCDYPSN | 1 |
| MFTVFLLVVLTTTVVSFPSDSASDGRDDEAKDERSDMHESDRNGRGCCCNPACGPNYGCGTSCSRTI | 1 |
| MGMRMMFTVFLLVVLATTVVSFTSDRASDGRDDEAKDERSDMHESDRNGRGCCCNPACGPNYGCGTSCSRTL | 1 |
| MGMRMMFTVFLLVVLATTVVSFPSDRASDGRDDEAKDERSDMHESDRNGRGCCCNPACGPNYGCGTSCSRTL | 1 |
| MGMRMMFTVFLLVVLATTVVSFPSDRASDGRDDEAKDERSDMHESDRNGRGCCCNPACGPNYGCGTSCSRTI | 1 |
| MGMRMMFTVFLLVVLATTVVSTPSDRASDGRNAAVHERQKSLVPSVITTCCGYDPGTMCPPCRCTNSCG | 0 |
| MFTVFLLVVLATNVVSTPSDRASDGRNAAVHERQKSLVPSVITTCCGYDPGTMCPPCRCTNSCG | 0 |
| MGMRMMFTVFLSVVLATTVVSTPSDRASDGRNAAVHERQKELVPSVITTCCGYDPGTMCPPCRCTNSCPTKPKKPGRRND | 1 |
| VVIVAVLLLTASQLITAEDSRGTQKHRTLRSTARRSKSELTTRCRSSGSPCGVTSICCGRCYRGKCT | 1 |
| MFTVFLLVVLATTVVSIPSDRAYDGKNAAVHERQSWLVPSTITTCCGYSPGTMCPPCMCTNTC | 0 |
| EACYPPGTFCGIKPGLCCSELCLPAVCVG | 0 |
| YECYSTGTFCGINGGLCCSNLCLFFVCLTFS | 1 |
| STTKVSKSTSCMKAGSYCVATTRICCGYCAYFGKICIGYPKN | 0 |
| QSWLVPSTITTCCGYSPGTMCPPCMCTNTC | 0 |
| CCKVQCESCTSCC | 1 |
| MEKLTILLLVAAVLTSTQALIQDRKERQKAEINFLSKRKSTFWRWWDGDCRTWRAPCNPGVECCTDVCRHGRCVFW | 1 |
| CCPARMCMAACSCCD | 0 |
| QCCDSNSCEYPKCLCCN | 1 |
| GCCSOWNCIQLRACGCC | 0 |
| KCCMRPICMCPCCIGPX | 0 |
| RCCPMPGCFAGPFCPCCPV | 0 |
| IVRCCSATCKXSCVCCF | 0 |
| KCCTMSVCQPPPVCTCCA | 0 |
| FCGQACSSVKCPKKCFCHPEEKVCYREMRTKERD | 1 |
| CCSQDCRVCIPCCPH | 0 |
| QCCDWPWCDDCICCD | 0 |
| CCAIRLCNVYLCGSCCO | 0 |
| RCCVHPACHDDCICCID | 0 |
| RCCISPACHDECICCID | 0 |
| ECCEWEWCDGACDCCN | 1 |
| ECCELEWCDGACDCCD | 1 |
| CRCEQTCGTCVPCC | 1 |
| DEMKNPKAFELTECSSYGEFCFPGIKDCCMGYCFIMCLPTSPHD | 0 |
| CLERGEPCIMPIVGLFACCSSVCIIVCI | 0 |
| GCGGVCAYGESCPSSCNTCYSAQCTAQ | 1 |
| RCAHGTYYSNDSQQCLLNCCWWGGGDHCCR | 1 |
| RCCVHPACHDDCICCIT | 0 |
| CCELPCHGCVPCCWP | 0 |
| GCCSOWNCIQLRACOCCONX | 0 |
| GCCPFPACTTHIICRCCX | 0 |
| CCKVLCESCTPCC | 0 |
| CCHPNLCIGRSALGRKCTCC | 1 |
| CCRIEFCDEDCGCC | 1 |
| CCEVGWCDSGCECC | 0 |
| CCNWPRCNVYLCGPCC | 1 |
| CCPIQGCILGCTPCC | 0 |
| CCYEEECPPSCKLCC | 1 |
| CCTGQCHICWPCC | 0 |
| CCMALCSRYHCLPCCX | 0 |
| CCMRPVCTCPCCS | 0 |
| CCPYPSCIDIPFCDCC | 0 |
| CCPFPMCYQVPHCPCC | 0 |
| CCTIWNCVQLPGCPCC | 0 |
| GCCOOQWCGODCTSOCC | 0 |
| CCTALCSRYHCLPCC | 0 |
| CCAFOQWCGAGCIVOCC | 0 |
| LCCOOQXCGODCASOCC | 0 |
| KCCMRPICTCPCCIGP | 0 |
| GCCPYPKCIHVTFCKCC | 0 |
| KSTAESWWEGECKGWSVYCSWDWECCSGECTRYYCELW | 1 |
| CNDPGGSCTRHYHCCQLYCNKQESVCLENEPAF | 1 |
| MLKVGVVLLIFLVLMSSAIPQDRDFSGQQSDKKQIRQRDIFGSKMRRYSPNIQKRCIVGTPCHVCRSQSKSCNGWLGKQGYCGYCG | 1 |
| MFTVFLLVVLATTVVSIPSDRASDGRNAAVNERAPWLVPSTITTCCGYDPGTMCPPCMCNNTCKPTKKRPGRRND | 1 |
| PLFDKRQRCCNGRRGCSSRWCRDHSRCCGRR | 1 |
| MGMRMMFTVFLLVVLATTVVSIPSDRASDGRNAAVNERQTWLVPSTITTCCGYDPGTMCPTCMCDNTCKPKPKKSGRRND | 1 |
| MGMRMMFTVFLLVVLATTVVSIPSDRASDGRNAEVNERAPWLVPSTITTCCGYDPGSMCPPCMCNNTCKPKPKKSGRRNH | 1 |
| CRPSGSPCGVTSICCGRCSRGKCT | 1 |
| AADCIEAGNYCGPTVMKLCCGFCSPYSKICMNYPKN | 0 |
| CESYGKPCGIYNDCCNACDPAKKTCT | 1 |
| EGCSSGGTFCGIHPGLCCSEFCFLWCITFID | 1 |
| DGCSSGGTFCGIRPGLCCSEFCFLWCITFID | 1 |
| GCCCNPACGPNYGCGTSCSRPSEP | 1 |
| ECCELEWCDGACDCCD | 1 |
| CCSVSICQPPPVCECCA | 0 |
| GILLPALRKFCCDSNWCNISDCECCY | 0 |
| CCSVSICQSPPVCECCA | 0 |
| QKCCGEGSSCPKYFKNNFICGCC | 1 |
| QCCDWPWCDDCICCD | 0 |
| RCCISPACHDDCICCIT | 0 |
| CCGPTACLAGCKPCC | 0 |
| AGLTVCLSENRKRLTCSGLLNMAGSVCCKVDTSCCSSQ | 1 |
| APWLVPSTITTCCGYDPGSMCPPCMCNNTCKPKPKKSX | 0 |
| QTWLVPSTITTCCGYDPGTMCPTCMCDNTCKPKPKKSX | 0 |
| QRCCNGRRGCSSRWCRDHSRCC | 1 |
| MEKLTILILLAAVLVLAQALIKGGGEKRQKEKINFLSKRKTTAESWWEGECSGWSVYCTWDSECCSGECTRSYCELW | 0 |
| MSKLGVVLIIAVLFLTASQLITADFSRDKRVHHAVRLRDIMRNFRGTRACAEFGHSCISATCCPGVTCVEIDEPVCLWD | 0 |
| MMFKLGVLLTIYMLLFPFTALPLDGDQPADQPLERMQYDMLRAVNPWFDPVKRCCSRNCAVCIPCCPNWPA | 0 |
| CLTTGETCWLASSCCSFSCTNNVCF | 0 |
| CLTTGETCWLASSCCSFSCTNNICF | 0 |
| CLTTGEYCWLASSCCSYSCTNNVCF | 1 |
| CLFSGEYCWLDTSCCSKSCTNNVCF | 1 |
| CLTTGEYCWLASSCCSYSCTDNVCF | 1 |
| MGVVLFTFLVLFPLATLQLDADQPVERYAENKQDHYPGESRGILKSALRKCCTMSVCQPPPVCTCCA | 0 |
| MLKLGMVLFIFLVLLPLATLHLDADQPVERNVNKQGLKPDERRRFILHALGQRQCCDWPWCDDCICCD | 0 |
| MFKMGVVLFTYLVLFPLATFQLDADQPVERYVENIQDLNPDERMEFILHALGQRRCCVHPACHDDCICCIDR | 0 |
| MFKMGVVLFTYLVLFPLATFQLDADQPVERYVENIQDLNPDERMEFILHALGQRRCCVHPACHDDCICCITR | 0 |
| MFKMGVVLFTYLVLFPLATFQLDADQPAERYAENKQDLNPDERMKFKLHNLGQRRCCISPACHDECICCIDR | 0 |
| CLQFGSTCFLGDDDICCSGECFYSGGTFGICSX | 0 |
| MLKLGMVLFIFLVLFPLATLHLDADQPVERNVNKQGLKPDERRRFGFSAPRKRECCEWEWCDGACDCCN | 1 |
| MLKLGMVLFIFLVLFPLATLHLDADQPVERNVNKQGLKPNERRRFRFSAPRKRECCELEWCDGACDCCD | 1 |
| MLKLGMVLFIFLVLLPLATLRLDADQPVERNVNKQGLKPDERRRFRFSAPRKRECCELEWCDGACDCCD | 1 |
| CCIARQICEGCICCI | 0 |
| APWLVPSTITTCCGYDPGTMCPPCMCNNTCKPTKKRP | 1 |
| CCPKEWCNRDCSCCT | 1 |
| CCTQSCTTCFPCC | 0 |
| MEKLTILLLHAAVLMSTQALIQGGGEKRPKEKINFLSKRKIAAKRWWLGECDEWDWLCDQPSECCSATCSLWC | 0 |
| MSRLGIMVLTLLLLVFMVTSHQDSREKQATRRNAVNIGWRRSIIRRTVGEECNEYCEQRNKNCCGKTNGEPVCAQACLG | 1 |
| CLDAGEVCDIFFPTCCGYCILLFCA | 0 |
| CYDSGTSCNTGNQCCSGWCIFVCL | 0 |
| CYDGGTSCDSGIQCCSGWCIFVCF | 0 |
| YDCEPPGNFCGMIKIGPPCCSGWCFFACA | 0 |
| CVPYEGPCNWLTQNCCDATCVVFWCL | 0 |
| NYCQEKWDYCPVPFLGSRYCCDGLFCTLFFCA | 1 |
| WCKQSGEMCNLLDQNCCDGYCIVLVCT | 0 |
| WCKQSGEMCNVLDQNCCDGYCIVFVCT | 0 |
| CCGPTACMAGCRPCC | 0 |
| CKQADEPCDVFSLDCCTGICLGVCMW | 0 |
| TCKQKGEGCSLDVECCSSSCKPGGPLFDFDC | 1 |
| CKCPSCNFNDVTENCKCCIFRQPX | 0 |
| MEKLTILLLVAAVLMSTQALVERAGENHSKENINFLLKRKRAADRGMWGECKDGLTTCLAPSECCSEDCEGSCTMW | 1 |
| MQKLIILLLVAAVLMSTQALFQEKRPMKKIDFLSKGKTDAEKQQKRSCSDDWQYCESPTDCCSWDCDVVCSG | 0 |
| MEKLIILLLVAAVLMSTQALFQEKRTMKKIDFLSKGKADAEKQRKRNCSDDWQYCESPSDCCSWDCDVVCSG | 0 |
| MQKLIILLLVAAVLMSTQAVLQEKRPKEKIKFLSKRKTDAEKQQKRLCPDYTDPCSHAHECCSWNCYNGHCTG | 1 |
| MQKLIILLLVAAVLMSAQAVLQEKRPKEKIKFLSKRKTDAEKQQKRLCPDYTEPCSHAHECCSWNCYNGHCTG | 1 |
| MQKLTILLLVAAVLMSTQALNQEQHQRAKINLLSKRKPPAERWWRWGGCMLWFGRCTKDSECCSNSCDRTYCELARFPSDW | 1 |
| CGGYSTYCEVDSECCSDNCVRSYCTLFX | 0 |
| DGCSNAGAFCGIHPGLCCSEICIVWCT | 0 |
| GCCHPSTCHMRKGCSRCCS | 1 |
| CCNAGFCRFGCTPCCY | 1 |
| WWGENDCSWTGPCTVNAECCLGVCDETC | 1 |
| TVTEECEEDCEDEEKHCCNTNNGPSCARLCF | 1 |
| CCPPVACNMGCKPCCX | 0 |
| CCRTCFGCTPCCX | 0 |
| GCNNSCQEHSDCESHCICTFRGCGAVNX | 0 |
| GMWGKCKDGLTTCLAPSECCSGNCEQNCKMW | 1 |
| WREGSCTSWLATCTDASQCCTGVCYKRAYCALWE | 1 |
| WWRWGGCMAWFGLCSKDSECCSNSCDVTRCELMPFPPDW | 0 |
| QQCCPPVACNMGCEPCC | 0 |
| WWECGIWFSRCTKDSECCSNSCDQTYCELMPFPPDW | 1 |
| WCKQSGEMCNLLDQNCCDGYCIVFVCT | 0 |
| CLDAGEICDFFFPTCCGYCILLFCA | 1 |
| CIEQFDPCDMIRHTCCVGVCFLMACI | 0 |
| CYDGGTSCDSGIQCCSGWCIFVCL | 0 |
| DCQEKWDFCPAPFFGSRYCCFGLFCTLFFCA | 1 |
| VCCPFGGCHELCQCCEX | 0 |
| CCRLLCLSCNPCC | 0 |
| CCDDSECSYSCWPCCY | 1 |
| DCRGYDAPCSSGAPCCDWWTCSARTNRCF | 1 |
| APWTVVTATTNCCGITGPGCLPCRCTQTC | 0 |
| QKWLVHSKITYCCGYNKMDMCPPCMCTYSCPPLKKKRP | 1 |
| QSWLVPSTITTCCGYDPGTMCPPCRCNNTCKPKKPKPGK | 1 |
| MEKLTILLLVAAVLTSTQALIQGGGDERQKAKINFLSRSDRDCRGYDAPCSSGAPCCDWWTCSARTNRCF | 1 |
| MLKMGVVLFIFLVLFPLATLQLDADQPVERYAENKQLLNTDERREIILSALRTRVCCPFGGCHELCQCCEG | 0 |
| MLKMGVMLFIFLVLFPLATLQLDADQPVERYAENKQLLSPDERREIILSALRTRVCCPFGGCHELCQCCEG | 0 |
| MLKMGVVLFIFLVLFPLATLQLDADQPVERYAENKQLLNPDEKRGILLPALRTRVCCPFGGCHELCQCCEG | 0 |
| MRCFPVFIILLLLIASAPCFDARTKTDDDVPLSSLRDNLKRTIRTRLNIRECCEDGWCCTAAPLTGR | 0 |
| MRCFPVFIILLLLIASAPCFDARTKTDDDVPLSPLRDNLKRTIRTRLNIRECCEDGWCCTAAPLTGR | 0 |
| MRCFPVFIILLLLIASAPCFDARTKTDDDVPLSSLRDNLKRTIRTRLNIRGCCEDGWCCTAAPLTGR | 0 |
| MEKLTILLLVAAVLMSTQAMFQGDGEKSRKAEINFSETRKLARNKQKRCGGYSTYCEVDSECCSDNCVRSYCTLFG | 1 |
| MEKLTILLLVAAVLMSTQALNQEQHQRAKINLLSKRKPPAERWWECGIWFSRCTKDSECCSNSCDQTYCELMPFPPDW | 0 |
| MEKLTILLLVAAVLMSTQAMFQEKSRKAEINFSETRKLARNKQKRCGGYSTYCEVDSECCSDNCVRSYCTLFG | 1 |
| MFTVFLLVVLATTVVSIPSDRASDGRNAAVNERAPWLVPSTITTCCGYNPGTMCPPCRCDNTC | 0 |
| MAMNMSMTLSTFVMVVVAATVTGFTHLQVPSLSRMERDGPADCCDMKTCCVDKMRTCLQRHTFNEKTWFPVVITKPATYVDLITKSWAVAMDIEHAC | 0 |
| DYDCEPPGNFCGMIKIGPPCCSGWCFFACA | 0 |
| CLEFGELCNFFFPTCCGYCVLLVCL | 1 |
| CKAENELCNIFIQNCCDGTCLLICIQNPQ | 0 |
| QCGADGQFCFLPGLGLNCCSGLCLIVCVPT | 0 |
| CCQWPCSHGCIPCCYX | 0 |
| VCTPPEGYCTYHRDCCDLYCNKTTNVCLET | 1 |
| MRCLPVFVILLLLIASVPSVDAELKAKDDMPQASFHDNAERDQQKKTSDCCFYHNCCC | 0 |
| MEKLTILLLVAAVLLSIQALNQEKHQRAKINLLSKRKPPAERWWRWGGCMAWFGLCSKDSECCSNSCDVTRCELMPFPPDW | 0 |
| MFTVFLLVVLATTVVSIPSDRASDGRNAAVNERQSWLVPSTITTCCGYDPGTMCPPCRCNNTCKPKKPKPGKGRRND | 1 |
| MEKLTILLLVAVVLMSTQALPQGGGEKRPRENIRFLSKRKSNAERWREGSCTSWLATCTDASQCCTGVCYKRAYCALWE | 1 |
| APWLVPSTITTCCGYNPGTMCPPCRCDNTC | 0 |
| MLKMGVVLFIFLVLFPLATLQLDADQPVERYAENKQLLSPDERRGILLPALRKFCCDSNWCNISDCECCYG | 0 |
| MMLKMGVVLFIFLVLFPLATLQLDADQPVERYAENKQLLNPDEKRGILLPALRRFCCDSNWCNISDCECCYG | 0 |
| MMLKMGVVLFIFLVLFPLATLQLDADQPVERYAENKQLLNPDEKRGILLPALRKFCCDSNWCNISDCECCYG | 0 |
| MLKMGVVLFIFLVLFPLATLQLDADQPVERYAENKQLLNPDEKRGILLPALRKFCCDSNWCNISDCECCYG | 0 |
| MLKMGVVLFIFLVLFPLATLQLDADQPVERYAENKQLLSPDERREIILSALRRQCCDSNSCEYPKCLCCNG | 0 |
| MLKMGVVLFIFLVLFPLATLQLDADQPVERYAENKQLLSPDERREIILSALRRQCCDRNSCEYPKCLCCNG | 0 |
| MMLKMGVVLFIFLVLFPLATLQLDADQPVERYAENKQLLNPDERRGILLPALRKFCCDSNWCHISDCECCYG | 0 |
| MLKMGVVLFIFLVLFPLATLQLDADQPVERYAENKQLLNPDERREILLPALRKFCCDSNWCHISDCECCYG | 0 |
| MCCLPVFVILLLLIASAPSVDAQPKTKDDVPLAPLHDNAKSALQHLNQRCCQTFYWCCVQGK | 0 |
| MEKLTILLLVAAVLMSTQALIQDQRQKAKINLFSKRQAYARDWWDDGCSVWGPCTVNAECCSGDCHETC | 0 |
| MEKLTILLLAATVLMSTQALIQEQSQKAEINLFSKRKPSAERWRVDSECISFWGSCTVDADCCFNSCDETYGYC | 0 |
| MEKLTILLLVAAVLMSTQALIQEQRQKAKINLFSKRKPSAERWWGENDCSWTGPCTVNAECCLGVCDETC | 0 |
| MSGLGIMVLTLLLLVFMATSHQDAGEKQATQRDAINVRRRRSLARRTVTEECEEDCEDEEKHCCNTNNGPSCARLCFG | 1 |
| NSPLRIRVVLGPAFDGRNAAVNERAPWTVVTATTNCCGITGPGCLPCRCTQTCG | 0 |
| NSPLRIRVVLGPEPDGRNAAVNERQKWLVHSKITYCCGYNKMDMCPPCMCTYSCPPLKKKRPGRRND | 1 |
| MLKMGVVLFIFLVLFPLATLQLDADQPVERYAENKQLLSPDERREIILHALGTRCCSWDVCDHPSCTCCG | 0 |
| MHLSLARSAVLMLLLLFALGNFVVVQSGQITRDVDNGQLTDNRRNLQSKWKPVSLYMSRRGCNNSCQEHSDCESHCICTFRGCGAVNG | 1 |
| MEKLTILLLVAAVLMSTQALVERAGENRSKENIKFLLKRKRAADRGMWGKCKDGLTTCLAPSECCSGNCEQNCKMW | 1 |
| WRVDSECISFWGSCTVDADCCFNSCDETYGYC | 0 |
| MSGLGIMVLTLLLLVFMVTSHQDSGEKQATRRNAVNIGWRRSIIRRTTEECHEYCEDQNKNCCGLTDGEPRCAGMCLG | 1 |
| DWWDDGCSVWGPCTVNAECCSGDCHETC | 0 |
| NCPYCVVYCCPPAYCQASGCRPP | 0 |
| EGNCTPWLGGCTSPEECCPGNCETYCRAW | 1 |
| VDEECNEYCDDRNKECCGRTNGHPRCANVCF | 1 |
| GDEECNEHCEDRNKECCGRTNGHPRCANVCF | 1 |
| GDEECNEYCDDRNKECCGRTNGHPRCANVCF | 1 |
| SIAGRTTTEECDEYCEDLNKNCCGLSNGEPVCATACL | 1 |
| GCCRWPCPSRCGMARCCSS | 1 |
| KTTAESWWEGECYGWWTSCSSPEQCCSLNCENIYCRAW | 1 |
| CCDWPCTIGCVPCCLP | 0 |
| KTTAESWWEGECSGWSVYCTSDPECCSGECSSYYCELW | 1 |
| CAAFGSFCGLPGLVDCCSGRCFIVCLL | 0 |
| MLKMGVVLFVFLVLFPLATLQLDADQPVERYAENKQLVSPYERRQIILHALGQRDCCVMPWCDGACDCCVSS | 0 |
| MLKMGVVLFVFLVLFPLATLQLDADQPVERYAENKQLVSPYERRQIILHALGQRQCCDWQWCDGACDCCA | 0 |
| MLKMGVVLFVFLVLFPLATLQLDADQPVERYAENKQLVSPYERKEIILHALGQRQCCDWQWCDGACDCCA | 0 |
| MLKMGVVLFTFLVLFPLATLQLDADQPVERYAENKQGLNPDESREIILSALRQRDCCEQGWCDGGCDCCQ | 0 |
| MEKLTILLLLAAVLVLAQALIKKGGGEKRQKEKINFLSKRKTTAESWWEGECSGWSVYCTSDPECCSGECSSYYCELW | 0 |
| MEKLTILLLVAAVLMSTQALIQRGGAKRRKVNFFSIREPGAEDWREGNCTPWLGGCTSPEECCPGNCETYCRAWR | 1 |
| MSGLGIMVLTLLLLVFMATSHQDAGEKQATQRDAINVRRRRSITRRVDEECNEYCDDRNKECCGRTNGHPRCANVCFG | 1 |
| MSGLGIMVLTLLLFMFMATSHQDAGEKQATQRDAINVRRRRSITRRGDEECNEHCEDRNKECCGRTNGHPRCANVCFG | 1 |
| MLKMGVVLFTFLVLFPLATLQLDADQPVERYAENKQGLNPDERMKFILHALGQRRCCISPACNDTCYCCQDR | 0 |
| MSGLGIMVLTLLLFMFMATSHQDAGEKQATQRDAINVRRRRSITRRGDEECNEYCDDRNKECCGRTNGHPRCANVCFG | 1 |
| DCWPQYWFCGLQRGCCPGTTCFFLCF | 1 |
| QCCDWQWCDGACDCCA | 1 |
| MSGLGIMVLTLLLLVFMVTSHQDSGEKRATRRNAINIGWRRSIIRRASLDEECNEYCKQRTKNCCGKTNEEPVCARRCLG | 1 |
| GILLPALRKFCCDSNWCNISDCECCY | 0 |
| DCGEQGQGCYTRPCCPGLECRGGGTGGGVCQQ | 1 |
| DCGEQGQGCYTRPCCPGLGCRAGATGGGVCQQ | 1 |
| WWDGDCRTWRAPCNPGVECCTDVCRHGRCVFW | 1 |
| WWLGECDEWDWLCDQPSECCSATCSLWC | 0 |
| TTEECHEYCEDQNKNCCGLTDGEPRCAGMCL | 1 |
| TVGEECNEYCEQRNKNCCGKTNGEPVCAQACL | 1 |
| DCCEQGWCDGGCDCCQ | 1 |
| ASLDEECNEYCKQRTKNCCGKTNEEPVCARRCL | 1 |
| CCSQDCSVCIPCCP | 0 |
| CCVVCNAGCSGNCCS | 1 |
| RCCISPACNDTCYCCQD | 1 |
| CCSQDCRVCIPCCPN | 0 |
| CCSQDCRVCIPCCPY | 0 |
| CCSRYCYICIPCCPN | 0 |
| CCSRYCWKCIPCCPY | 1 |
| DCCVMPWCDGACDCCVSS | 0 |
| CCSQDCSVCIPCCPN | 0 |
| MSGLGIMVLTLLLLVFMATSHQDAGEKQATQRDAVNVRRRRSIAGRTTTEECDEYCEDLNKNCCGLSNGEPVCATACLG | 1 |
| VILLMSTQALIQSGVEKRSNKIKALSKRKTTAESWWEGECYGWWTSCSSPEQCCSLNCENIYCRAW | 1 |
| MHLSLARSAGLMWLLLFAVGNFVGVQPGQITRDVDNGQLADNRRNLQSLRKPMTLFKSLNKRVSCGEYCGDYGDCPSSCPTCTSNLLKCM | 1 |
| QCCDRNSCEYPKCLCCNX | 0 |
| GCCHPSTCHVRKGCSRCCP | 1 |
| GCCHLLACRMGCTPCCW | 0 |
| LPDDISSENNPFYDPVKWCCMTCFGCTPCCGSPASSSV | 1 |
| CCMTCFGCTPCC | 0 |
| CCDDSECDYSCWPCCIFS | 0 |
| CCSWDVCDHPSCTCCGX | 0 |
| GCCGAFACRFGCTPCC | 1 |
| QCCDSNSCEYPKCLCCNX | 0 |
| SCCNAGFCRFGCTPCCY | 1 |
| GCCHPSTCHVRKGCSRCCS | 1 |
| KFCCDSNWCHISDCECCYX | 0 |
| CCGPTACLAGCKPCCX | 0 |
| CCDDSECSTSCWPCCY | 1 |
| QVKPCRKEHQLCDLIFQNCCRGWYCVVLSCT | 1 |
| CAPFLHLCTFFFPNCCNGYCVQFICL | 0 |
| CYDSGTSCNTGNQCCSGWCIFVSCL | 0 |
| DDCEPPGNFCGMIKIGPPCCSGWCFFACA | 0 |
| RCCKFPCPDSCRYLCCX | 0 |
| CCGPTACLAGCKPCCY | 1 |
| CCGPTACLAGCKPCC | 0 |
| FCCDSNWCNISDCECCY | 1 |
| MNPAGRLLLLGLALGLLFESLGKPMADDVHAERDTDPGDKAPRAISAERADVPCGDGTCTFGCCENGICKELNCLDVSNTESSWKRWSLSGSR | 1 |
| MSTQALIEGDGEKKGQKAKINFLTARNLLGNKKARGRCIGLSNYCGPWNNPPCCSRWICESRYCDFPNVLS | 1 |
| MKKLIILLLVAAVLLSTQAWIQSDGEKRQKVKVKFLSKRKPSVMSWWGSKCNIFLMGCKVHADCCSNNCEGHCRLW | 0 |
| MQNSKGAKSTRDCSPSGDGCHTRTCCPGLRCSGPQQAQVCH | 1 |
| GMWGECKDGLTTCLAPSECCSEDCEGSCTMW | 1 |
| SCSDDWQYCESPTDCCSWDCDVVCSX | 0 |
| NCSDDWQYCESPTDCCSWDCDVVCSX | 0 |
| LCPDYTEPCSHAHECCSWNCYNGHCTG | 1 |
| CIEQFDPCEMIRHTCCVGVCFLMACI | 0 |
| CAPFLHPCTFFFPNCCNSYCVQFICL | 0 |
| CVDCRPGYKCCGVCTMNQCTGREIPKE | 1 |
| WWRWGGCMLWFGRCTKDSECCSNSCDRTYCELARFPSDW | 1 |
| MSRSGMALLVFLLLLSLVTNLQGKGEGQTMHQNKHRQTVRKLMTLRRTQKRNACELDSSTGDDCTGTQICCNEPGSMSGECKETDECPDRRR | 1 |
| NCPYCVVYCCPPAYCEASGCRPPX | 0 |
| MMWKLGVVLLIFLVLLPLTAPRQDGDGMAYTGRHVLHRMKNALKITKRDCGERDEPCCVNSSGVKYCESPWSCMHTTLLCEQN | 1 |
| MLKMGVVLFIFLVLFPMATFQLDAERPAEHLGNKQNLNRDERMRIISFALKQWGCCDTGWCDAGCGCCV | 0 |
| MATFQLDAERPAERYLGNKQNLNRDERMRIISSTLKQRDCCKEEWCDGGCYCCV | 1 |
| MLKIGVMLSIILVLFPLATLQLVAERPAAERYAENKQDLNPDERRNYLVDLGVERTCCTACNIPPCKCCA | 0 |
| GILLPALRKFCCDSNWCNISDCECCY | 0 |
| DCQEKWDYCPVPFLGSRYCCDGFICPSFFCAX | 0 |
| CRSSGSPCGVTGICCGRCYRGKCT | 1 |
| CTHPGGACGGHHHCCSLSCNTAANACN | 1 |
| VCCPFGGCHELCLCCDX | 0 |
| DCGAQGEGCYTRPCCAGLSCVGGHSGGLCQY | 1 |
| DCKADGAFCGIPFVKNWMCCSNLCIFACVPE | 0 |
| DCKALGEFCGIPYVHNSQCCSQLCGFICV | 0 |
| CQPNGYYCDFEFTPKCCLKCDYNRKYCQPY | 1 |
| RCVFCPKEPCCDGDQCMTAPGTGPFC | 0 |
| CTYWLGPCIVDSECCFNSCEERFCGLW | 0 |
| CTYWLGPCIVDSECCSNSCEERFCGLW | 0 |
| GGCTPCGPNLCCSEEFRCGTSTHHQTYGEPACLSY | 1 |
| TCDPYYCNDGKVCCPEYPTCGDSTGKLICVRVTD | 1 |
| WWEGECYDWLRQCSSPAQCCSGNCGAHCKAW | 1 |
| CWAGGSPCHLCSSSQVCIAPTGHPAIMCGRCVPILT | 0 |
| CIVGTPCHVCRSQSKSCNGWLGKQRYCGYC | 1 |
| TNCEAHSCSPSCPDECYCDTNEDTCHPE | 1 |
| CIVGTPCHVCRSQSKSCNGWLEKQRYCGYC | 1 |
| NGCNGNTCSNSPCPNNCYCDTEDDCHPD | 1 |
| KPSEDCGKTCETAENCPDDCSSCLSVEGTYRCA | 1 |
| GCLPDEYFCGFSMIGALLCCSGWCLGICMT | 1 |
| ADCSTYCFGMGICQSGCYCGPGHACMPN | 0 |
| NCGEQGEGCATRPCCSGLSCVGSRPGGLCQY | 1 |
| KVCLESGAVCVIPIIASFACCSAFCYGVCL | 0 |
| QCRVEGEICGMLFEAQCCDGWCFFVCM | 0 |
| VCIADDMPCGFGLFGGPLCCSGWCLFVCL | 0 |
| GCREGGEFCGTLYEERCCSGWCFFVCV | 1 |
| KTTAEKWWDGDCMTWYAPCRLDSQCCSGNCGKHRCLAW | 1 |
| KSTAESWWEGECSGWSVYCEYDSECCSGECGGYYCELW | 1 |
| QCSPVGGSCSHHYNCCSLYCNKNIGQCLATSYP | 1 |
| QCSANGGSCTRHYHCCSLYCNKDSSVCVATSYP | 1 |
| CTENGDVCDPENHNCCSGSCLDDEDPPVCGF | 1 |
| QCSPNGGYCTLHIHCCSNHCIKPIGRCVA | 0 |
| VPYCGQTGAECYSWCKEQHLIRCCDFVKYVGMNPPADKC | 1 |
| CDRSCTGVMGHPSCATCCACFTSAG | 0 |
| IPYCGQTGAECYSWCIKQDLSKDWCCDFVKDIRMNPPADKCP | 1 |
| IPYCGQTGAECYSWCIKQDLSKDWCCDFVKTIARLPPAHICSQ | 0 |
| CCMRPICMCPCCVN | 0 |
| CCQAACSPWLCLPCC | 0 |
| CCHAPYCTPPHLGCPCC | 1 |
| IPYCGQTGAECYSWCKEQHLIRCCDFVKYVGMNPPADKC | 0 |
| GCTPPGGACGYHGHCCDFCDTFGNLC | 1 |
| WWDGECRLWSNGCRKHKECCSNHCKGIYCDIW | 1 |
| CRPPGMVCGFPKPGPYCCSGWCFAVCLPV | 0 |
| MHLSLASSAALMLLLLFALGNFVGVQPGQIRDLNKGQLKDNRRNLQSQRKQMSLLKSLHDRNGCNGNTCSNSPCPNNCYCDTEDDCHPDRREH | 1 |
| MHLSTASSVALMFFLLFAFYGVQPELMTRDVDNGQLTDNRRNLRSRVKPTGLFKSRKPSEDCGKTCETAENCPDDCSSCLSVEGTYRCA | 1 |
| MHRSLAGSAVLMLLLLFALGNFVGVQPGLVTRDADNGQLMDNRRNLRLERKTMSLFKSLDKRADCSTYCFGMGICQSGCYCGPGHACMPNGR | 0 |
| CCEWPCSHGCIPCCY | 1 |
| CCEWPCHHGCIPCCYX | 0 |
| ECTRPGGACYYDSHCCRHVCHEVFNTCM | 1 |
| ECTPPGGACNIHPHCCEEFCDMANNRCLEM | 1 |
| MLKVGVVLLIFLVLMSSAIPQDRDFSGQQSDKKQIRQRDIFGSKMRRYSPNIQRRCIVGTPCHVCRSQSKSCNGWLEKQRYCGYCG | 1 |
| CCNCSSKWCRDHSRCCGR | 1 |
| YECCVWPHCDGGCSSCVRSCE | 1 |
| YECCVWPYCDGGCSSCVRSCE | 1 |
| GCCTACHIPPCTCC | 0 |
| GCCTGQGCWNVPICECC | 0 |
| CCTACRMPPCTCCA | 0 |
| CCNCSSKWCRDHSRCCX | 0 |
| NGCCNCSSKWCRDHSRCCX | 0 |
| RYVENKQDLSPDERMGIRSTVTSPEGQKCCLSGFCDGACRQCKDTCE | 1 |
| MHLSLASSAVLMLLLLFALGNFVGVQPGQITRDADNLRNLRSQWKKRGLFKSLDKRTNCEAHSCSPSCPDECYCDTNEDTCHPERRGH | 1 |
| MLKVGVVLLIFLVLMSSAIPQDRGQQSDKKQIRQRDIFGSKMRRYSPNIQKRCIVGTPCHVCRSQSKSCNGWLGKQRYCGYCG | 1 |
| MSKVGVVPLIFLVLLSIAALQNGDDPRRQRDEKQSPQGDILRSTLTKYSYNIQRRCWAGGSPCHLCSSSQVCIAPTGHPAIMCGRCVPILT | 0 |
| CAAPGASCSKYDNECCDACLLQYPNPPVC | 1 |
| GCMEDGDVCELGNHNCCSGSCLGFEDEGIC | 1 |
| CTNPGGYCVVPHHNECCSGQCDPSSIIGQC | 0 |
| CSYFGADCLPDSHDCCSGECFGFPDMGLCT | 0 |
| DCCPLPACPFGCNPCCX | 0 |
| MIMRMTLTLFVLVVMTAASASGDALTEAKRIPYCGQTGAECYSWCKEQHLIRCCDFVKYVGMNPPADKCR | 0 |
| MIMRMTLTLFVLVVMTAASASGDALTEAKRVPYCGQTGAECYSWCKEQHLIRCCDFVKYVGMNPPADKCR | 0 |
| MIMRMTLTLFVLVVMTAASASGDALTEAKRIPYCGQTGAECYSWCIKQDLSKDWCCDFVKDIRMNPPADKCP | 0 |
| MIMRMTLTLFVLVVMTAASASGDALTEAKRIPYCGQTGAECYSWCIKQDLSKDWCCDFVKTIARLPPAHICSQ | 0 |
| MMSTLGVLLTIGLLLFPLTALPLDGDQPADQPAERLQDISPKEIPGSDPFKRCCHAPYCTPPHLGCPCCGK | 0 |
| MCCIPVFIILLLLIPSAPSILGQPTTKGDVALASSYDDAKRILQRYLDHPWCCAVKYSCCVPGIIPI | 0 |
| MCCIPVFFILLLLIPSAPSILAQPTTKGDVALASSYDDAKRTLQRLSIEYSCCPGIVSCCVIP | 0 |
| MCCIPVFFILLLLIPSAPSILAQPTTKGDVALASSYDDAKRTLQRLSIKYSCCPGIVSCCVIP | 0 |
| MEKLTILLLVAAVLVSTQALIQGGEKRQKAMINFLSKREPFAKRWWGDERCTYWLGPCIVDSECCFNSCEERFCGLW | 0 |
| MEKLTILLLVAAVLVSTQALIQGGEKRQKAMINFLSKRKPSAKRWWGDERCTYWLGPCIVDSECCSNSCEERFCGLW | 0 |
| MSKLGVVLFTLLLLVPLVTPERDGGKWTMLAKNKKAMKRNLMDFITRTCDPYYCNDGKVCCPEYPTCGDSTGKLICVRVTD | 0 |
| MEKLTILLLVTAVLMSTQALMQSGIEKRQRAKIKFFSKRKTTAERWWEGECYDWLRQCSSPAQCCSGNCGAHCKAW | 1 |
| TVTEECEESCEDEEKHCCNTNNGPSCARICF | 1 |
| KVPEACEESCEEEEKHCCNTSNGLSCVRHCL | 1 |
| IPCGGTGGFCYWLMPCCDSRDFCDRSVCTRESDLVVHIT | 0 |
| IVTEECEEYCEDEEKDCCGLQNGQPFCAPFCL | 1 |
| DWCGDAGDACGTLKLRCCSGLCNQYSGTCT | 1 |
| DDECEPPGDFCGFFKIGPPCCSGWCFLWCA | 0 |
| ALRSTKVSKSPPCLVAGSSCRGTTRVCCGFCSHYGYKCRDRPTS | 1 |
| CVPSGGSCSRTAYSCCHGSCSGGRC | 1 |
| ECEMLCEQEEKHCCRIRNENIQCAPRCLGIGV | 1 |
| PCANLGRACDTVPCCLGVRCFESRTPTCLLKQRGV | 1 |
| GVWSECSDWLAGCSSPSECCSEKCDTFCRLW | 1 |
| CKSPGTPCSRTMRDCCTSCLSYSKKCR | 1 |
| CVGRDSKCGPPPCCMGMTCNYERVRKCT | 1 |
| WGLCSLPAEAGPCYASITRYYYDRKTQECTQFYYGGCGGNSNNFDTAEECDDVC | 1 |
| DICRMPKVVGPCMAGITRYYYDTASAACRQFIYGGCQGNLNNFGSLEACQGKCARH | 0 |
| DICRMPKVVGPCMAGITRYYYDTASAACRQFIYGGCQGNLNNFGSLEACQGKCARH | 0 |
| CQQPKAPGRCMAYMERYFFNSEKGACEQFIYGGCEGNENNFETLEACQTAC | 1 |
| DLCFQPMVVGLCKASFPNYYYNPALGTCQLFYYGGCGGNKNRFGTKDACLKTC | 1 |
| CTLPAERGPCMANLTMYFYNWTSEQCEEFNYGGCGGNPNNFHNMTECEATCSR | 1 |
| RDCCTPPKKCKDRQCKPQRCCAX | 0 |
| TPMCGATCAMACPNDFVEDEHGCPICKCREE | 0 |
| ACSGRGSRCPPQCCMGLTCGREYPPRC | 1 |
| EKLCGELYDGCHDQRCCPGLTCDTLFQCVRHS | 1 |
| EICQQPRQVGPCRAAFRRWFYNKFTRTCEQFIYGGCKGNGNNFQSLPECQDRC | 1 |
| QKELVPSTITTCCGNEPGTMCPKCMCDNTCPP | 0 |
| QKELVPSTITTCCGNEPGTMCPKCMCDNTCIISKLASF | 0 |
| QKELVPSIITTCCGNEPGTMCPKCMCDNTCPPKKKKRP | 1 |
| QKELVPLTITTCCGNEPGTMCPKCMCDNTCPPKKKKRP | 1 |
| QKELVLSTITTCCGNEPGTMCPKCMCDNTCPPKKKKRP | 1 |
| QKELAPSTITTCCGNEPGTMCPKCMCDNTCPPKKKKRP | 1 |
| QKELVPSTITTCCGNEPGTMCPKCMCDNTCPPKKKKRP | 1 |
| QRCCTGKKGSCSGKACKSLKCCAG | 1 |
| MCCRWPCPRQIDGEYCGCCL | 1 |
| VQPSECKLPAAKGPCKGKYRKVYFNNFKKQCRMFTYGGCGGNGNKFRNAKECYHKCAYGV | 1 |
| MCCRWPCPRHVDGKYCVCCL | 0 |
| ACSGRGSRCPPQCCMGLRCGRGNPQKCIGAHEDV | 1 |
| CCGKPNAACHPCVCNGSCS | 1 |
| CCGKPTSACHPCVCNGSCSG | 1 |
| CRLPSDTGPCRAAIRQFYYNWTERQCQDFIYGGCGGNDNRFETREECERAC | 1 |
| SVCTLQKDTGPCKMAIPRYYFNMDISDCDTFIYGGCFGNANNFETYEECDDTC | 1 |
| CNLPQIVGPCKAYMPSFFYNTGTGQCERFVYGGCGGNANRFETKQECQGQCQR | 1 |
| RDCCTPPRKCKDRRCKPMKCCAX | 0 |
| GCCIVPWCTGCYCCH | 0 |
| RDCCTPPKKCKDRRCKPLKCCAX | 0 |
| CKSPGTPCSRGMRDCCTSCLLYSNKCRRY | 1 |
| DCLPPLTWCSMTDDECCNDCVLFLC | 0 |
| CTVDSDFCDPGNHNCCSGKCIDEGGSGVCAIIP | 0 |
| ESDSGLAPGTCVLFGSMCKAKTASICCYKCDLEEGIC | 0 |
| CKLSGEDCGYGYSCCEDLSCRPLIEPDTLKITALVCQIESA | 0 |
| MHLSLARSAVLMLLLLFALGNFVVVQSGLITRDVDNGQLTDNRRNLQTEWNPLSLFMSRRSCNNSCQSHSDCASHCICTFRGCGAVNG | 0 |
| CCDDSECDYSCWPCCMF | 0 |
| AVTEECKEDCNDENKKCCGEENGEPVCATACL | 1 |
| CLPGTATCNLYNNLCCNYCLIFWC | 0 |
| VVTEACEETCEDEEKTCCGLENGEPVCARFCL | 1 |
| CKQADESCNVFSLDCCTGLCLGFCVS | 0 |
| CVPYEGPCNWLTQNCCDELCVFFCL | 0 |
| CYDGGTGCDSGNQCCSGWCIFACL | 1 |
| DCQALWDYCPVPLLSSGDCCYGLICGPFVCIGW | 0 |
| SCNNSCQSHSDCASHCICTFRGCGAVNX | 0 |
| VKPCRKEGQLCDPIFQNCCRGWNCVLFCV | 0 |
| VITEVCEGYCEVLRKHCCGTRDAIPVCNSACR | 1 |
| CRLGAESCDVISQNCCQGTCVFFCLP | 0 |
| MEKLTILLLAAAVLMSTQATIQGGGENRPKENIKYLSKSQRSAERGVWSECSDWLAGCSSPSECCSEKCDTFCRLWR | 1 |
| MSGLRIMVLTLLLLVLMTTSHQDAGEKQAMQRDAKNFSRRRLGIRKPKTRECEMLCEQEEKHCCRIRNENIQCAPRCLGIGV | 1 |
| MCCLPVFVILLLLITSAPSVDALPKTRDDVPLASFHGGYNARRILQRRQGWCCKENIACCI | 0 |
| CKSPGTPCSRGMRDCCTSCLSYSNKCRRY | 1 |
| ICQLEADVGPCSGTFPRWFYNSDMRKCQLFDYGGCRGNDNRFDTEEECMELC | 1 |
| ICQLEADVGPCSGTFPRWFYNSDMRKCQLFDYGGCRGNDNRFDTEEECMELC | 1 |
| VCSLPRERGPCSNYEIVWYYDTEEERCKRFYYGGCQGNGNRFANREECEGRCVR | 1 |
| DFVSICDMPEDPGPCRGRLPRWFYDPLDRQCRAFYWSGCQGNENNFLTVQECQQTCM | 1 |
| DFVSICDMPEDPGPCRGRLPRWFYDPLDRQCRAFYWSGCQGNENNFLTVQECQQTCM | 1 |
| VCKQAPSPGRCNAVFRRWYFNVHVAACSWFTYSGCGGNDNNFRSREECERMC | 1 |
| DPCQLPKDPGPCPNQHHNFFSDSEMGACKMFIYGGCYGNANNFRTLEECQATC | 1 |
| CQLPKDPGPCTSPIHRFFFNSETGACEVFIWGGCYGNANKFKTLEECQETC | 0 |
| DRCHLPPETGMCRAYMPMYFYNATLGRCQGFIYGGCNGNDNKFNTEEDCMKAC | 1 |
| TVTEECEEDCEDEEKHCCNTNNGPSCARLCF | 1 |
| FPCSSGRCACLPKDGSSTSFQCQSTSASTENCFDNHCITEDEW | 1 |
| MPSVQSVTRCCLLWLMLSVQLVTPGSRGTAQLPRDDLDETTREEICRYMCSRKIDYHMCTCPSKRDAISSRIVRRKRSMAV | 0 |
| MGMRMMFTVFLLVAWQRLVSSTSDRASDGRNAAASDKASDLITQVVKRCCGKPNAACHPCVCNGSCSGGR | 1 |
| MGMRMMFTVFLLVVLATTVVSSTSDRASDGRNAAASDKASDLITQVVKRCCGKPNAACHPCVCNGSCSGRR | 1 |
| MGMRMMFTVFLLVSTWQRLSFPPLQIVHLMAGMPQASDKASDLITQVVKRCCGKPTSACHPCVCNGSCSGGR | 0 |
| MCCLPVFVILLLLITSAPSVDALPKTRDDVPLASFHGGYNARRILQRRQGWCCKENIACCV | 0 |
| CKSPGSSCSPTSYNCCRSCNPYTKRCYX | 0 |
| QKELVPSTITTCCGNEPGTMCPKCMCDNTCPPKEEKTRPQ | 1 |
| FCCVFPWCTGCYCC | 1 |
| CCDDSECDYSCWPCCIFS | 0 |
| MHLSLAGSALLMLLLLFALGNFVAVQSGQISRDVDNGQLTDDRRNLQSQRKPMTLSRSPYKRALCGESCSLHSHCIGFCEYCSARTYTCI | 0 |
| MTLFRSLNKRSSCGEPCIQDEHCSGTCNICTGNLSKCM | 1 |
| MTLTKSAVLILVLLLAFDNFADVQPGLITMGGGRLSNLLSKRVSIWYCASRTCSAPADCNPCTCESGVCVDWL | 0 |
| MTLTKSAVLILVLLLAFDNFADVQPGLITMGGGRLSNLLSKRVRIWFCASRTCSAPADCNPCTCESGVCVDWL | 0 |
| MTLTKSAVLILVLLLAFDNFADVQPGLITMGGGRLSNLLSKRVSIWFCASRTCSTPADCNPCTCESGVCVDWL | 0 |
| MTLTKSAVLILVLLLAFDNFADVQPGLITMGGGRLSNLLSKRVSIWFCASRTCSAPADCNPCTCESGVCVDWL | 0 |
| MLKMGVVLFTFLVLFPLATFQLDADQPVERYAENKQDLSPDKRVEFILHALGQRFCCVFPWCTGCYCCR | 0 |
| MEFRRLVTVALLLSLVMSIDSVPADELEPGRVSPRETDTFPCYSNQCACLPKGGTTTSYQCQSTTASTDNCVNNECITEANWGGR | 1 |
| MLKMGVLLFTFLVLFPLTTLELDTDRPVERHAAIKQDLKPQERRGIRLHAPRDECCEPQWCDGACDCCS | 0 |
| MLKIGVVLFTLLVLFPLATLQLDADQPVERYAENKQDLNPNERMKMIMSALGQRRCCISPACHEECYCC | 0 |
| MLKMGVLLFTFLVLFPLAMFQLDADQPVERYAENKQDLNRDERMKIMLSALRQRQCCDWEWCDELCSCCW | 1 |
| MLKMGVLLFTFLVLLPLAMFQLDADQPVERYAENKQDLNRDERMKIMLSALRQRQCCDWEWCDELCSCCW | 1 |
| MKLTRVLIIAVLFLTAYQLTTVETYSRGKWMHRALRSTGKNPKVTRECSSPDESCTYHYNCCQLYCNKEENVCLENSPEV | 1 |
| MEKLTILLLVAALLMSTQGLIQSGGENRPKEKIKFLSKRKTVAESWWEGECLGWSNYCTSHSICCSGECILSYCDIW | 0 |
| MMSKLGVLLTICLLLFPLTAVPLDGDQPADRPAERKQDVSSEQHPFFDPVKRCCRRGYMGCIPCCF | 0 |
| MEKLTSLLLVAALLMLTQTLIQGGGEDRPNKKFLQKIKSTAKRECTAPSGYCDYPEECCEVECGRHYCDWWY | 1 |
| MLKIGVVLFTFLVLFPLATLQLDADQPVERYAENKQDLNPNERMKMIMSALGQRRCCISPACHEECYCCQ | 0 |
| MSGLGTMVLTLLLLVFMVTSHQDGGKKQATQRNAVNIRRRKSITQRTTDEKCNEYCEERDRNCCGKANGEPRCARMCFG | 1 |
| MHLSLTGSALLMLLLLFALGNFVGAQSGQISRDVDNGQLTDDHRNLQSQWNPMTLFRSLYKRSSCGDPCAQHASCTVTCTYCTGTLLQCI | 1 |
| MKLFVAIVLILMFLSLSAGAETSDNGVSRGGHPPDYWPVTPPSIVCLRSGEDCENNTPCCPGLSCRVSADLATLKLSLACD | 0 |
| MGVVLFTFLVLFPLATLQLDADQPVERYTENKQDLSPDDRMGFILPALRRYECCVWPYCDGGCSSCVRSCE | 0 |
| PVERYAENKQDLNPDERKDFILPIVRGCCTACHIPPCTCC | 1 |
| MMSKLGVLLTICLLLFPLTALTKEGDQPVGRPAERQHGKISSEQHTMFDPIEGCCQQSCTTCFPC | 0 |
| MLKMGVVLFTFLVLFPLATLQLDADQPVERYAENKQDLNPNERMKMIMSALGQRRCCISPACHEECYCCQ | 0 |
| MSKLGVVLLIFLVLLPLTSPHQNGNGFAGNQARQMGVQRRKIGLANALRRSSCGYLGQQCCIVPKRAYCHGDLECNPVAMCVA | 0 |
| MRCFPVFIILLLLIASAPCFDARTKTDDDVPLSPLRDNLKRTIRTRLNIRECCEDGWCCTAAPLTGR | 0 |
| GVVLFTILVLLPLATLLLEADQPVERQQDLNPQRGTRGIMKHVMSKGMSRRGCCTGQGCWNVPICECC | 0 |
| MEKLTILLLVAALLMSTQGLIQEKRQKAKITIFSKRKSNAERWWEGDCTDWLGSCSSPSECCYDNCETYCTLWK | 1 |
| MLKMEVVLFTFLVLFPLSALQLETDQPVERYAENKQDLNPDESRNFMLPIVKKCCTACRMPPCTCCA | 0 |
| MKLVLAIVLILMFLSLSAGAETSDNGVSRGGHRPQYWPVTPPSIVCLRSGEDCENNTPCCPGLSCRVSADLATLKLSLACD | 0 |
| MEKLTILLLVAAVLMSTQALVERAGENRSKENIKFLLKRKRAADRGMWGKCKDGLTTCLAPSECCSGNCEQNCKMW | 1 |
| MRNFRGFRSCSEQGEGCATRPCCPGLNCVGGIAGGLCQD | 1 |
| MSGSGVALITFLLLLSLMTNLQGGGEGQRMHQDKHRQTARKLFTLGRKVKRNDPCELDSPYDDCTGTQICCTQPGSMSGECKEADEC | 1 |
| MSGLGIMVLTLLLLVFMATSHQHRGGKQLMLRNNLQKRTCSSPSNCPTGQECCPDKVDEPEGFCADECIIT | 0 |
| MEKLTILLLVAALLMSTQGLIQSGGENRPKEKIKFLSKRKSVAESWWEGECLGWSNYCTSHSICCSGECILSYCDIW | 0 |
| MHTLEMMLLILLLMPLAPGEGDGRAMGGNRNPSEARSIHKRLLERPARRLDRRECTPCAPNLCCEPGSTCGTSSTHHGYGEPACVY | 1 |
| MLKMGVVLFTFLVLFPLATLQLDADQPVERYAENKQDLSPDKRVEFILHALGQRGCCIVPWCTGCYCCH | 0 |
| MLKMGVVLFTFLVLFPLATLQLDADQPRARYAENKQDFNRNERTKMILSALGQRRCCIWPECGSCVCCL | 0 |
| MMLKMGVVLFVFLVLFPLATLQLDADQPVERYVENKQDLNPDERRGSILPAQKQQRCCEAPFCNAGCTCCNP | 0 |
| MMLKMGVVLFVFLVLFPLATLQLDADQPVERYVGNKQDLNPDERRGFISPALRRRRCCTWQECDGNCRCCQR | 1 |
| VLIIAVLFLTGSELVTADYTRDKWQYRAASLRDAMRNFRDTRTCSPAGEVCTSKSPCCTGFLCSHIGGMCHH | 0 |
| VLIIAVLFLTASELVTADYTRDKWQYRAASLRDAMRNFRDTRTCSPAGEVCTSKSPCCTGFLCSHIGGMCHH | 0 |
| VLIIAVLFLTASELVTADYTRDKWQNRAASLRDAMRNFRDTRTCSPAGEVCTSKSPCCTGFLCSHIGGMCHH | 0 |
| VLIIAVLFLTDSELVTADYTRDKWQYRAASLRDAMRNFRDTRTCSPAGEVCTSKSPCCTGFLCSHIGGMCHH | 1 |
| VLIVAVPFLTAGELVTADYTRDKWQYRAASLRDAMRNFRDTRTCSPAGEVCTSKSPCCTGFLCSHIGGMCHH | 1 |
| VLIIAVLFLTASELVTADYTRDKWQYRAASLRDAMRNFRDTWTCSPAGEVCTSKSPCCTGFLCSHIGGMCHH | 0 |
| VLIIAVLFLTASELVTADYTRDKWQYRATSLRDAMRNFRDTGTCSPAGEVCTSKSPCCTGFLCSHIGGMCHH | 0 |
| MLKMGVVLFVFLVLFPLATFQLDADQPVKRYAKYKQDLNADKRMRLILPALRQRQCCGWEWCDDICGCCE | 0 |
| VLIIAVLFLTASELVTADYTRDKWQYRAASLRDAMRNFRDTRTCSPAGEVCTSKSPCCTGFLCTHIGGMCHH | 0 |
| VLIIAVPFLTASELVTADYTRDKWQYRAASLRDAMRNFRDTRCSPGGEVCTRHSPCCTGFLCNHIGGMCHH | 1 |
| VVIIAVLFLTASELVTADYTRDKWQYRAASLRDAMRNFRDTRCSPGGEVCTRHSPCCTGFLCNHIGGMCHH | 1 |
| VLIIAVLFLTASELVTADYTRDKWQYRAASLRDAMRNFRDTRCSPGGEVCTRHSPCCTGFLCNHIGGMCHH | 1 |
| VLIIAVLSLTASELVTADYTRDKWQYRAASLRDAMRNFRDTRCSPGGEVCTRHSPCCTGFLCNHIGGMCHH | 1 |
| VLIIAVLFLTASELVTADYTRDKWQYRAASLRDAMRNFRDTRCSPGGEVCTRHSPCCTGFLCNHIGGMCHP | 1 |
| VLIIAVLFLTASELVTADYTRDKWQYRAASLRDAMRNFRDTRCSPGGEACTRHSPCCTGFLCNHIGGMCHH | 1 |
| VLIIAVLFLAASELVTADYTRDEWQYRAASLRDAMRNFRDTRCSPGGEVCTRHSPCCTGFLCNHIGGMCHH | 0 |
| VLIIAVLFLTASELVTADYTRDKWQYRAASLRDAMRNFRDTRCSPGGEVCTSKSPCCTGFLCSHIGGMCHH | 0 |
| MLKMGVMLFTFLVLFPLATLQMDADQPVARYAENKQNLNPNERMKLMLSALRQQTCCEPSTCDGGCYHCC | 0 |
| MLKMGVMLFTFLVLFPLATLQMDADQPVARYAENKQNLNPNERMKLMLSALRQQECCKPSTCDGGCYHCC | 0 |
| MLKMGVVLFTFLVLFPLSTLQLNADQHVARYAENKQNFNPNERTKMMLPALRQQPCCSPSTCDGGCYHCC | 0 |
| MMLKMGVVLFVFLVLFPLATLQLDADQPVERYAENKQDLNPDGKREFILPAVGRTCCDLTWCDGNCRCCTPR | 0 |
| MMLKMGVVLFVFLVLFPLATLQLDADQPVKRYVGNKQDLNPDERRGFISPALRRRRCCTWQECDGNCRCCQR | 1 |
| MFKMGVVLFTYLVLFPLATFQLDADQPVERYAENKQDLNPDERMKFKLHNLGQRRCCISPACHDECICCIDR | 0 |
| MLKMGVMLFTFLVLFPLATLQLDADQPVERYAEDKQDLNRDERMGFILHALGQRQCCDWPWCDDCICCD | 0 |
| MMLKMGVVLFVFLVLFPLATLQLDADQPVERYVGNKQDLNPDERRGFISPALRRRRCCTWQECDGNCHCCQR | 1 |
| MLKMRVVLFTFLVLFPLATLQLDADQPRARYAENKQDFNRNERTKMILSAVSASMGRQRRCCFWPMCGGCDCCYL | 1 |
| MLKMRVVLFTFLVLFPLATLQLDADQPRARYAENKQDFNRNERTKMILSAVSASMDRQRRCCFWPMCGGCDCCYL | 1 |
| MLKMRVVLFTFLVLFPLATLQQDADQPRARYAENKQDFNRNERTKMILSAVSASMGRQRRCCFWPMCRGCDCCYL | 1 |
| MLKMGVVLFTFLVLFPLATLQLDADQPRARYAENKQDFNRNERTKMILSAVSASMGRQRRCCLWPECGGCVCCYL | 1 |
| MLKMGVVLFTFLVLFPLATLQLDADQPRARYAENKQDFNRNERTKMILSAVSASMGRQRRCCIWPECGSCVCCL | 1 |
| MLKMGVVLFTFLVLFPLATLQLDADQPRARYAENKQDFNRNERTKMILSAVSASMGRQRRCCLWPACWGCVCCY | 0 |
| MLKMRVVLFTFLVLFPLATLQQDADQPRARYAENKQDFNRNERTKMILSAVSASMGRQRRCCFWPACWGCVCCY | 1 |
| MLKMRVVLFTFLVLFPLATLQLDADQPRARYAENKQDFNRNERTKMILSAVSASMGRQRRCCLWPECGGCVCCY | 1 |
| MLKMGVVLFTFLVLFPLATLQLDADQPRARYAENKQDFNRNERTKMILSAVSASMGRQRRCCLWPECGGCVCCY | 1 |
| MLKMGVVLFTFLVLFPLATLQLDADQPRARYAENKQDFNRNERTKMILSAVSASMGRHRRCCFWPDCRGCYCCL | 1 |
| MLKMGVMLFTFLVLFHLAAPQLDADQPVARYAGNTPDFNPNERMKMMLSALRQRRCCISPACHKDCYCCL | 0 |
| MLKMRVVLFTFLVLFPLATLQLDADQPVARYAENKQNFNPNERMKMMLPALRQQPCCSPSTCDGGCYHCC | 0 |
| MGVVLFTFLVLFPLATLQLDADQPVERYVENKQDLNPDDRMGFILPALRRYECCVWPHCDGGCSSCVRSCE | 0 |
| MSKLGVVLLIFVVLLALTSPHHYGNRFAGYQARQMGVQQRKNALANALRRSGCGYLGEPCCVAPKRAYCHGDLECNNIAMCVN | 0 |
| MKKLTILLLIAAVLMLTQALIQEKRPEDEIKFLSKRKSGAQRWWDGECRLWSNGCRKHKECCSNHCKGIYCDIW | 1 |
| AVLFLTAWTFVTADSIRALEDLFAKARDKMENSGASPLNQRDCKALGEFCGIPYVHNSQCCSQLCGFICV | 0 |
| MSGLGIMVLTLLLLVFMEASHQDAGEKQATQRDAINVRRRRSLARRTVTEECEEDCEDEEKHCCNTNNGPSCARLCFG | 1 |
| KIDNCTLPAERGPCMANLTMYFYNWTSEQCEEFNYGGCGGNPNNFHNMTECEATCSR | 1 |
| LLLTATVIVVATGNRCRLPSDTGPCRAAIRQFYYNWTERQCQDFIYGGCGGNDNRFETREECERAC | 1 |
| LVTVALLLTFVMSIDSAPADQTETGRVSLREDQRFPCSSGRCACLPKDGSSTSFQCQSTSASTENCFDNHCITEDEW | 1 |
| MQGKISSEQHPMFDPIEGCCTQSCTTCFPCCLI | 0 |
| CCQAACSPWPCLPCC | 0 |
| RCCISPACHEECYCCQ | 1 |
| GCGYLGEPCCVAPKRAYCHGDLECNNIAMCVN | 0 |
| SSCGYLGQQCCIVPKRAYCHGDLECNPVAMCVA | 0 |
| RRSLAQRAVTEECKEDCNDENKKCCGEENGEPVCATACLG | 1 |
| INVRRRRSLTRRVVTEACEETCEDEEKTCCGLENGEPVCARFCLG | 1 |
| AINVRQRRSVTRRVITEVCEGYCEVLRKHCCGTRDAIPVCNSACRG | 1 |
| EKQAMERDAINIRWRRSLTRRIVTEECEEYCEDEEKDCCGLQNGQPFCAPFCLG | 1 |
| VLLNSAVDGIPCGGTGGFCYWLMPCCDSRDFCDRSVCTRESDLVVHITRGRRALHTRFFRR | 1 |
| FMETSHQDAGEKQSTQRDAINVRQGRSLTRRKVPEACEESCEEEEKHCCNTSNGLSCVRHCLG | 1 |
| MSGLGMMVLTLLLLVFMETSHQDAGEKKATQRDAINVRRRRSLAQRTVTEECEESCEDEEKHCCNTNNGPSCARICFG | 1 |
| VILMLLSLSTGAEKSGLEISMVGPPLYFWSPSPACKLSGEDCGYGYSCCEDLSCRPLIEPDTLKITALVCQIESA | 0 |
| DVCALPKVAGPCFAAFPRFYFDKTAGRCKTFTYGGCHGNQNNFRSLRACRNTCP | 1 |
| CTLQISTGTCPGYFPRWFYDPASGQCQSFIYSGCKGNANNFLSEEECHQACVS | 1 |
| HVVSLCMLPADTGPCFASMPRYYFDMDSYDCTTFVYGGCDGNGNNFRTYVECYGTCGLE | 1 |
| CCTVCTTGCVVCC | 0 |
| CCDRPCSIGCVPCCLP | 0 |
| CCNAGFCRFGCTPCCY | 1 |
| RDCCTPPKKCKDRQCKPQRCCA | 1 |
| CCGPTACLAGCKPCC | 0 |
| CCSQDCSVCIPCCPN | 0 |
| CCIGPKCMEECYCCV | 0 |
| CCEPQWCDGACDCCS | 1 |
| CCISPACHEECYCCQ | 0 |
| ACVELGEICATGFFLDEECCTGSCHVFCVL | 0 |
| SCGRRGKPCPCCRGFRCTGSFCRKWQ | 1 |
| DCVKAGTACGFPKPEPACCSSWCIFVCT | 0 |
| SPFDCSSPGAFCGLVPCCDSCNVLGRC | 0 |
| MLKMGVVLFTFLILFPLVTLQLDADQRVERYAENKQDLNPEERREIKLLALRQRCCIGPKCMEECYCCV | 0 |
| MLKMGVLLFIFLVLFPLTTLELDTDRPVERHAAIKQDLKPQERRGIRLHAPRDECCEPQWCDGACDCCS | 0 |
| MLKMGVVLFTFLVLFPLATLQLDADQPVERYAENKQDLNPNERMKMIMSALGQRRCCISPACHEECYCCQ | 0 |
| CRLPPETGMCRAYIPMYFYNATLGRCQGFIYGGCNGNDNKFNTEENCMKACHH | 1 |
| CLRSGEDCENNTPCCPGLSCRVSADLATLKLSLACD | 1 |
| DCGEQGQGCYTRPCCPGLECLGGGTGGGVCQP | 1 |
| GMWGKCKDGLTTCLAPSECCSGNCEQNCKMW | 1 |
| MTEVDCGGVSCEFHCCETINGVQRCREINCN | 1 |
| CCISPACHEECYCCQ | 0 |
| CCISPACHEECYCC | 0 |
| QCCDWEWCDELCSCCW | 0 |
| CCKVQCESCTPCC | 1 |
| DAMQKSKGSGSCAYISEPCDILPCCPGLKCNEDFVPICL | 0 |
| ECSSPDESCTYHYNCCQLYCNKEENVCLENSPEV | 1 |
| WPCKVAGSPCGLVSECCGTCNVLRNRCV | 0 |
| DECCEPQWCDGACDCCS | 1 |
| GECLGWSNYCTSHSICCSGECILSYCDIW | 0 |
| DKNSKLTRQCSPVGGSCSHHYNCCSLYCNKNIGQCLATSYP | 1 |
| STDKNSKLTRQCSANGGSCTRHYHCCSLYCNKDSSVCVATSYP | 1 |
| TDKNSKLTRQCSPNGGYCTLHIHCCSNHCIKPIGRCVA | 1 |
| KLFSKARDEMKNPEDSKLDKKKVCLESGAVCVIPIIASFACCSAFCYGVCL | 0 |
| MHTLEMLLLVLLLVPLAPGEGDGQAVGGDRNPSEARRAYKRLLQRPARRMDRGGCTPCGPNLCCSEEFRCGTSTHHQTYGEPACLSY | 1 |
| MPSVRWLLAVTTVAVVVIVMVLSGPYSGFRTPNQATVQMTRNAGHHEGKATKLQTGGTSSGIVDRCRLPPETGMCRAYIPMYFYNATLGRCQGFIYGGCNGNDNKFNTEENCMKACHH | 0 |
| FLTAWTFVTADDSINALEDLFSKARDEMENGEASTLNERDCKADGAFCGIPFVKNWMCCSNLCIFACVPE | 0 |
| MEKLTILLLVAALLMSTQALIQGGGEKRQKAKINFLSKRKSTAESWWEGECSGWSVYCEYDSECCSGECGGYYCELW | 0 |
| MKKLTILLLVAALLMSAQALIQGGVEKRQKAKMDFSKRKTTAEKWWDGDCMTWYAPCRLDSQCCSGNCGKHRCLAW | 1 |
| VSIWFCASRTCSTPADCNPCTCESGVCVDWL | 0 |
| VSIWFCASRTCSAPADCNPCTCESGVCVDWL | 0 |
| SCSEQGEGCATRPCCPGLNCVGGIAGGLCQD | 1 |
| NDPCELDSPYDDCTGTQICCTQPGSMSGECKEADEC | 1 |
| CTDEGGDCDPGNHNCCRGSCLVLQHKAICGIVYTMVS | 0 |
| TCSSPSNCPTGQECCPDKVDEPEGFCADECIIT | 1 |
| KSVAESWWEGECLGWSNYCTSHSICCSGECILSYCDIW | 0 |
| ECTPCAPNLCCEPGSTCGTSSTHHGYGEPACVY | 1 |
| GGHPPDYWPVTPPSIVCLRSGEDCENNTPCCPGLSCRVSADLATLKLSLACD | 1 |
| CVEPGSPCSKYDNECCDACMLSHPNPPVCIE | 0 |
| QLPAMPFPDSSITPKPTVPPDCAFKSCQTKAECCNGYYFCGAGFCIPNMNKIFDY | 0 |
| WWEGDCTDWLGSCSSPSECCYDNCETYCTLW | 1 |
| SSCGDPCAQHASCTVTCTYCTGTLLQCI | 0 |
| QCPVTGGPTPMYHCMMACMTTSTEYWCQHEYCSDCAE | 0 |
| ETDTFPCYSNQCACLPKGGTTTSYQCQSTTASTDNCVNNECITEANWG | 1 |
| ALCGESCSLHSHCIGFCEYCSARTYTCI | 0 |
| SSCGEPCIQDEHCSGTCNICTGNLSKCM | 0 |
| RVSIWYCASRTCSAPADCNPCTCESGVCVDWL | 0 |
| VRIWFCASRTCSAPADCNPCTCESGVCVDWL | 0 |
| CTDEGGDCDPGNHNCCRGSCLVLQHKAVCGILYTMVS | 1 |
| MMLKMGVVLFVFLVLFPLATLQLDADQPVERYAENKQDLNPDGKREFILPAMRRQGCCIEPLCYQYDCDCCRHL | 0 |
| QKELVPSTITTCCGNEPGTMCPKCMCDNTCPPKEERP | 1 |
| QKELVPSTITTCCGNEPGTMCPKCMCDNTCPPKEERQ | 1 |
| ECTHSGGACNSHNQCCNAFCDTATRTCV | 1 |
| ECTHSGGACNSHTQCCDDFCSTATSTCI | 1 |
| ECTRSGGACNSHTQCCDDFCSTATSTCI | 1 |
| MFTVFLLVVLATTVVSIPSDRASDGRNAVVHERAPELVVTATTTCCGYDPMTWCPSCMCTYSCPHQRKKPGRRND | 0 |
| MFTVFLLVVLATTVVSIPSDRASEGRNAVVHERAPELVVTATTTCCGYDPMTICPPCMCTHSCPPKRKPGRRND | 0 |
| MFTVFLLVVLATTVVSIPSDRASDVRNAAVHERQKDLVVTATTTCCGYNPMTICPPCMCTYSCPPKKKKPGRRND | 1 |
| MFTVFLLVVLATTVVSIPSDRASDGRNAAVHERAPWLVPSQITTCCGYNPGTMCPSCMCTNSC | 0 |
| ECTHSGGACNSHDQCCNTFCDTATRTCV | 1 |
| MMLKMGVVLFVFLVLFPLATLQLDADQPVERYAENKQDLHPNERTGFILPAMRRQGCCIEPLCYQYDCDCCRHL | 0 |
| MMLKMGVVLFVFLVLFPLATLQLDADQPVERYAENKQDLHPNERTGFILPAMRRRCCRLRRCKTHCHCCVYRP | 1 |
| MMLKMGVVLFVFLVLFPLATLQLDADQPVERDAENKQDLHPDERTGFILPAMRRRCCRLRRCKTHCHCCVYRP | 1 |
| MMLKMRAVLYVFLVLFPLATLQLDADQPVERYAENKQDLNPDGKREFILPAVGRTCCDLTWCDGNCRCCTPR | 1 |
| MLKMGVVLFIFLVLSPLATLQLDADQPVERYAENKQLLSPDERREIILHALGTRCCSWDVCDHPSCTCCG | 0 |
| MKLTYIMIVAVLLLTAWTFVTADDYKYGLENRFSKARNEVENPKASELDKRSCYEAGEFCFFSVPGLFICCSSVCLFHVCL | 0 |
| VLIIAVLFLTACQLTTAETYSRGRQKHRARRSTDKNSKWTRECTHSGGACNSHDQRCNAFCDTATRTCV | 1 |
| LNKRCAGIGSFCGLPGLVDCCSGRCFIVCLP | 0 |
| MMLKMGVVLFVFLVLFPLATLQLDADQPVERDAENKQDLHPDERTGFMLSAVGRRCCSLRRCEPHCHCCVQRH | 1 |
| MGMRMMFTVFLLVVLATTVVSIPSDRASDGRNAAVHERAPWLVPSQITTCCGYNPGTMCPSCMCTNTC | 0 |
| ECTHSGGACNSHDQCCNAFCDTATRACV | 1 |
| ECTRSGGACYSHNQCCDDFCSTATSTCV | 1 |
| CCRLRRCKTHCHCCVYRP | 1 |
| CCSQNCLTCIPCCPY | 0 |
| CCPPVACNMGCKPCC | 0 |
| CCGPTACLAGCKPCC | 0 |
| EFILPAVGRTCCDLTWCDGNCRCCTP | 0 |
| CCSWDVCDHPSCTCC | 0 |
| ECTHSGGACNSHDQCCNAFCDTATRTCV | 1 |
| PKASELDKRSCYEAGEFCFFSVPGLFICCSSVCLFHVCL | 0 |
| ECTRSGGACNSHTQCCDDFCDTATRTCV | 1 |
| ECTRSGGACNSHTQCCDDFCSTATSTCT | 1 |
| CTQTNGACYHRDTCCSKSCNLTINRCLAS | 1 |
| ECTDSGGACNSHDQCCNEFCSTATRTCI | 1 |
| ECTRSGGACNSHDQCCANFCRKATSTCM | 1 |
| ECAHSGGACNSHDQCCNAFCDTATRTCV | 1 |
| ECTRSGGACNSHTQCCDHFCSTATSTCI | 1 |
| ECTRSGGACYSHNQCCDDFCSTATSTCI | 1 |
| ECTRSGGACNSHTQCCNAFCDTATRTCV | 1 |
| MSKLGVVLLIFVVLLALTSPHHYGNRFAGYQARQMGVQQRKNALANAVRRSGCGYLGEPCCISPKRAYCHGDLECNNVAMCVN | 0 |
| MKLTYMMIVTVLILTTWTVVMADDPKNGLEILFSKARDEMKNPEASKLNKKTCLGKNALCGAPGVGVLVCCSFKCVVVCV | 0 |
| MSGLGIMVLTLLLLVFMATSHQHRGGKQLMLRNNLQKRTCSSPSNCPTGQECCPDKVDEPEGFCADECIIT | 0 |
| DCTPPGGACGGHAHCCSKSCNIMASTCQ | 0 |
| GDEECNEYCDDRNKECCGRTNGHPRCANVCFX | 0 |
| DAMQKSKGSGSCADLSEACDILLCCPGLKCNEDFIPICL | 0 |
| TCLGKNALCGAPGVGVLVCCSFKCVVVCV | 0 |
| TCSSPSNCPTGQECCPDKVDEPEGFCADECIIT | 1 |
| CTDEGGDCDPGNHNCCRGSCLVLQHKAICGIVYTMVS | 0 |
| CAGIGSFCGLPGLVDCCSGRCFIVCLP | 1 |
| CTDDSQFCNPSSHNCCSGTCIDEGGSGVCAIVPVTV | 0 |
| VSCGDTCINSDECPSSCNTCLHALCKS | 0 |
| GCCRWPCPSICGMARCCSS | 0 |
| CCSRHCWVCIPCCPNGS | 0 |
| CCSQDCSVCIPCCPN | 0 |
| GCCGAFACRFGCTPCC | 1 |
| SCCNAGFCRFGCTPCCY | 1 |
| CCILCWKCTYNCCRAW | 0 |
| ACCDPDWCDAGCYDGCC | 1 |
| VVIVAVLLLTACQLITANDSRGTQKHRALRSDTKLSMSTRRQGRGASCRKTMYNCCSGSCNRGRCG | 1 |
| CAAFGSFCGLPGLVDCCSGRCFIVCLL | 0 |
| GCGYLGEPCCISPKRAYCHGDLECNNVAMCVN | 1 |
| CCDWPCTIGCVPCCLP | 0 |
| APWTVVLSTTNCCGYNTMEFCPACMCTYSCPKKKKPGK | 1 |
| QPWLVPSTITTCCGYDPGTKCPPCRCNNTCKPKKPKPGK | 1 |
| CAGIGSFCGLPGLVDCCSGRCFIVCLP | 1 |
| AFDGRNAAVNERAPWTVVLSTTNCCGYNTMEFCPACMCTYSCPKKKKPGKGRRNN | 1 |
| MFTVFLLVVLATTVVSIPSDRASDGRNAAVNERQPWLVPSTITTCCGYDPGTKCPPCRCNNTCKPKKPKPGKGRRND | 1 |
| CCSQDCSVCIPCCPW | 0 |
| RCCISPACNDTCYCCQD | 1 |
| CCQAACSPWLCLPCCX | 0 |
| CCSRYCYICIPCCPN | 0 |
| DCCVMPWCDGACDCCVSSX | 0 |
| CCSQDCWVCIPCCPN | 0 |
| CCQAACSPWLCLPCC | 0 |
| CCRAACSPWLCLPCC | 0 |
| CCVVCNAGCSGNCCP | 0 |
| CCSRYCYICIPCCPN | 0 |
| CCVVCNAGCSGNCCS | 1 |
| GCCRWPCPSRCGMARCCSS | 1 |
| CCSLRRCEPHCHCCVQRH | 1 |
| GCCIEPLCYQYDCDCCRHL | 1 |
| ECTRSGGACNSHDQCCNAFCDTATRTCV | 1 |
| MLKVGVVLLALLLLVPLVAPEQDEGKWTMLAKNENTVKRKLMDFMARECDPYYCSNGKVCCPDYPTCGDSTGKLICVRVTD | 0 |
| CTPRNGVCFYSYFCCSRACNPSTKRCL | 1 |
| CTSPGGACYSASTCCSKACNLTTKRCVLS | 1 |
| CMHPEGGCRFSYECCSKICYTPSFTCYPS | 0 |
| CTPAGKACDATATCCVLFCNLVTNKCQVPRFP | 0 |
| CTDPGGACGNPGHCCSKFCITTSSTCN | 0 |
| WIVGVCRLPGDLCAGDASCCEHSCNIVHTCD | 0 |
| QVKPCRKEHQLCDLIFQNCCRGWYCLLRPCI | 0 |
| FAVIFTCTPPGSHCTGHSDCCSDFCSTMSDVCQ | 0 |
| VKPCSEEGQLCDPLSQNCCRGWHCVLVSCV | 0 |
| CAQSSELCDALDSDCCSGVCMVFFCL | 0 |
| CYDGGTGCDSGNQCCSGWCIFVCL | 1 |
| DCQEKWDYCPVPFLGSRYCCDGFICPSFFCA | 0 |
| DCQGEWEFCIVPVLGFVYCCPWLICGPFVCVDI | 0 |
| DCIPGGENCDVFRPYRCCSGYCILLLCA | 0 |
| ACKPKNNLCAITEMAECCSGFCLIYRCSX | 0 |
| MLKMGMVLFVFLVLFPLATFQLDADQPVERYVGNKQDLNPDERRGFISPALRQKRCCSESHCNAGCACCD | 1 |
| CLGGGEVCDIFFPQCCGYCILLFCT | 0 |
| MMLKMGVVLFVFLVLFPLATLQLDADQPVERYVENKQDLNPDERRGSILPAQKQQRCCEEPFCNAGCACCNP | 0 |
| CCDRPCGIGCVPCCLP | 0 |
| CCRTCFGCTPCC | 0 |
| MMLKMGVVLFVFLVLFPLATLQLDADQPVERYVGNKQDLNPDERRGFISPALRRRRCCNWQECDGNCHCCQR | 1 |
| MMLKMVVVLFVFLVLFPLATLQLDADQPVERYVGNKQDLNPDERRGFISLALSRRRCCTFSECDGNCHCCQG | 0 |
| MLKMGVVLFVFLVLFPLATFQLDADQPVKRYAKYKQDLNTDKRMRLILPALRQRQCCGWEWCDDICGCCE | 0 |
| MMLKMGVVLFVFLVLFPLATLQLDADQPVERYVENKQDLNPDERRGFILPALKQKRCCPASACNGACGCCE | 0 |
| MLKMGVVLFVFLVLFPMATLQLDADQPVERYAENKQDHNADERMGYILPALSQQTCCVRPWCDGACDCCVDP | 0 |
| MLKMGVVLFVFLVLFPLATLQLDADQPVERYAENKQDLNADERMGYILPALSQQTCCVRPWCDGACDCCVDS | 0 |
| RCCSESHCNAGCACCD | 1 |
| CCEEPFCNAGCACCNP | 0 |
| MGYILPALSQQTCCVRPWCDGACDCCVDS | 0 |
| MGYILPALSQQTCCVRPWCDGACDCCVDP | 0 |
| RCCPASACNGACGCCE | 1 |
| QCCGWEWCDDICGCCE | 0 |
| RCCTFSECDGNCHCCQ | 1 |
| RCCNWQECDGNCHCCQ | 1 |
| CCSQDCSVCIPCCPN | 0 |
| RCCTWQECDGNCRCCQ | 1 |
| TCCPPGICSSDKCSCC | 0 |
| STSCMKAGSYCRSTTRTCCGYCAYFGKFCIDFPSN | 1 |
| MLKMGVVLFTFLVLFPLATLQLDADQPAARYAENKQDFNPNERKEMMLSALRQRACCDPDWCDAGCYDGCC | 0 |
| CKGKGASCSRTMYNCCSGSCNRGKCGX | 0 |
| CKGKGASCRRTSYDCCTGSCRSGKCX | 0 |
| CDGHGVLCDYDSECCSGECTTTGAIEYC | 0 |
| CLGFGEACLMLYSDCCSYCVGAVCL | 1 |
| YECYLLVHFCGINGGLCCSNLCLFFVCLTFS | 1 |
| CNEAQEHCTQNPDCCSESCNKFVGRCLSD | 1 |
| ATPECSRAGCKNVPCCSGLKCTGPQSGPVCQPE | 1 |
| CTEEGEACEPEDHDCCSERCHETDHVCSPSVEVFELS | 1 |
| DAINVAPGTSITRTETDQECIDICKQEDKKCCGRSNGEPTCAKICL | 1 |
| LVEGACTSPSNCPTGQECCPDKLDEPEGSCANSCPFY | 1 |
| DAINVAPGTSIIGTETNQECIDICKQEDKNCCGKSNGVPTCGKICL | 0 |
| ATPECSRAGCKNVPCCSGLKCTGPQNGPVCQPE | 1 |
| APWLVPSQITTCCGYNPGTMCPSCMCTNTC | 0 |
| MEKLTILLLVAAVLLSTQVLVQGDGEKSQKRMDLLKGRMLSGNLNKRCDTWRDPCTYEHECCWQYHCGFRTCE | 1 |
| MSGLGFMVVTLLLLVPMATSQQDGGEKQAMQRDAINVAPGTSITRTETDQECIDTCKQEDKKCCGRSNGVPTCAKICLG | 0 |
| MSGLGFMVLTFLLLVSMATSKQDGRVRRRMLHTRLVEGACTSPSNCPTGQECCPDKLDEPEGSCANSCPFY | 1 |
| MSGLGFMVLTLLLLVPMATSQQDGGEKQAMQRDAINVAPGTSITRTETDQECIDICKQEDKKCCGRSNGEPTCAKICLG | 0 |
| MSGLGFMVLTLLLFVPMATSQQDGGEKQAMQRDAINVAPGTSIIGTETNQECIDICKQEDKNCCGKSNGVPTCGKICLG | 0 |
| ECDPYYCSNGKVCCPDYPTCGDSTGKLICVRVTD | 1 |
| DAINVAPGTSITRTETDQECIDTCKQEDKKCCGRSNGVPTCAKICL | 1 |
| CDTWRDPCTYEHECCWQYHCGFRTCE | 1 |
| CVSPGGVCQHKDECCSDRCEQSAIVSICKQ | 1 |
| CKGKGAPCTRLMYDCCHGSCSSSKGRCX | 0 |
| DECFSPGTFCGFKPGLCCSARCFSFFCISLEFX | 0 |
| DECFSPGTFCGIKPGLCCSARCFSFFCISLEFX | 0 |
| DECFSPGTFCGTKPGLCCSARCFSFFCISLEFX | 0 |
| YECYSTGTFCGINGGLCCSNLCLFFVCLTFS | 1 |
| QKCCGKGMTCPRYFRDNFICGCC | 1 |
| RCCGEGASCPRYFRNSQICSCC | 1 |
| RCCGEGASCPVYSRDRLICSCC | 1 |
| RCCRWPCPRKIDGEYCGCCLX | 0 |
| QGCCNGPKGCSSKWCRDHARCCX | 0 |
| QGCCGEPNLCFTRWCRNNARCCRQQ | 1 |
| GRCCDVPNACSGRWCRDHAQCCX | 0 |
| DGCYNAGTFCGIRPGLCCSEFCFLWCITFVDS | 0 |
| APELVVTATTTCCGYDPMTWCPSCMCTYSCPHQRKKP | 0 |
| APELVVTATTTCCGYDPMTICPPCMCTHSCPPKRKP | 0 |
| QKDLVVTATTTCCGYNPMTICPPCMCTYSCPPKKKKP | 0 |
| APWLVPSQITTCCGYNPGTMCPSCMCTNSC | 0 |
| CKGTGKPCSRIAYNCCTGSCRSGKCX | 0 |
| QKELVPSTITTCCGNEPGTMCPKCMCDNTCPPKEERPAQ | 1 |
| MLKMGVVLFTFLVLFPLATLQLDADQPAARYAENKQDLNPNERKEMMLSALRQRACCDPDWCDAGCYDGCC | 0 |
| MGMRMMFIVFLLVALATTVGSFTSDRASDGRNAAVNNKASHLIDNVIRDCCGVKLDMCHPCLCNNSCKQGQGKKRVWEMMKATDKRN | 1 |
| MQNSRRSKLARECLPKGAGCDEPFECCSHLCTGRLHTTC | 1 |
| MEKLTALILVATVLLTTQVLVQSDGERPLKRRVKEYAAKRLSALMRGSRQCLPQYHPCIIGNEECCPHLECKCLPKP | 0 |
| QKLTILLLVAAVLISTHILGQGDGEKRMKTKMDFFKARKHWAYKQARDCKGWLEECEEENCCDPYECIGFC | 1 |
| MSGLGIMVLTLLLLVSMATNHQDGRVRRLMLRNRLRQMMCTTDEDCPTGQECCPDNINDPQGFCVDDCIV | 1 |
| MSGLGIMVLTLLLLVFMETSHQDAGEKQATQRDAINVRRRRSLTRRVVTEACEESCEDEEKTCCGLENGEPVCARFCLG | 1 |
| LMLRNRLRQMMCSSNEDCPAGQECCPDNLGKPEGFCVDDCII | 1 |
| LLFALGTFVGVQLEQITRDVDNGQPTDNRHNLQSVWKQMSLLRSVVKRCVGSCDLNNPCSSGCICNGDKC | 1 |
| KLRTKMQNSRRSKFARTCRERGIGCDDPLECCSHQCTFTGFSTC | 1 |
| GNFVGVQPGQITRDMENGQLMDNRRDLRSPWKQMSLFRSFKLVCGKGCKSRPDCPTECFCYMDMC | 1 |
| VCSLAPETGNCRANIPRWYYDAQFGQCRQFVYGGCRGNSNNFETEQDCLNYCRR | 1 |
| ICQLEADVGPCSGTFPRWFYNSGMRKCQLFDYGGCRGNENRFDTEEECMELC | 1 |
| DACSLPLSTGKCEQQQTRWHYNYRSGSCEKFIYTGCLGNANNFPTADACQARC | 1 |
| EVCSLPRERGPCSNYEIVWYYDTAEQRCTRFYYGGCQGNGNRFANREECEERCVR | 1 |
| DICRMPKVVGPCRAGITRYYYDSASAACRQFIYGGCQGNLNNFGTLEACQGKCARH | 1 |
| DVCALPKVTGPCFAAFPRFYFDQTAGRCKTFTYGGCHGNQNNFRSLRACRNTCA | 1 |
| LCRLPAVPGPCRSRQPRYFYNYKVGKCQRFNYGGCKGNTNRFLTLGECQSRC | 1 |
| QKELVPSTITTCCGNEPGTMCPKCMCDNTCPPQKEKRP | 1 |
| CQLEPDTGLCRAAFRRFYYNWNEQQCQAFIYGGCGGNENRFKSREECEQAC | 1 |
| SRDKEGYRAVRSRLGVRHGMRFSECSTRGCTFSSHCCEGLRCQGTPQGGVCV | 1 |
| MSKLGVVLFTILVLLPLATLLLEADQPVERQQDLNPQRGTRGIMKHVMSKGMSRRGCCTGQGCWNVPICECCV | 0 |
| QKELVVTATTTCCGYNPMSMCPKCMCTYSCPHQQ | 0 |
| QKELVVTATTTCCGYNPMSMCPKCMCTYSCPHN | 0 |
| QQECCDPDWCDGACYNCC | 1 |
| DCHESGDFCGIPGVKNGLCCSQSCWVVCTPEW | 0 |
| ILSLSLLDRSTRCKGTGKSCSRIAYNCCTGSCRSGKCG | 1 |
| CKGTGKSCSRIAYNCCTGSCRSGKCX | 0 |
| QKELVPSTITTCCGHEPGTMCPKCMCDNTCPPQKEEKTRPQ | 1 |
| QKELVPSTITTCCGNEPGTMCPKCMCDNTCTPQKEEKTRPQ | 1 |
| QKELVPSTITTCCGHEPGTMCPKCMCDNTCPPKRRKTRPQ | 1 |
| QKELVPSTITTCCGHEPGTMCPKCMCDNTCPPKRERP | 1 |
| QKELVPSTITTCCGHEPGTMCPKCMCDNTCPPKREKTRPQ | 1 |
| QKELVPSTITTCCGHEPGTMCPKCMCDNTCPPKNEEKTRPQ | 1 |
| QKELVPSTITTCCGHEPGTMCPKCMCDNTCPPKKKKRPRPQ | 1 |
| QKELVPSTITTCCGHEPGTMCPKCMCDNTCPPKEERQ | 1 |
| QKELVPSTITTCCGHEPGTMCPKCMCDNTCPP | 0 |
| MMKMGAMQFTFLVLFLLVALQVDADQPAERHAENKQDLNPVERKEIIVPALRQNTNSDEEDCCIYPWCDGDCLCC | 0 |
| QKELVVTATTTCCGYNPMSICPKCMCTYSCPHQQKKRP | 1 |
| CNLPKETGPCRALDHSFFYDVNAGQCKHFIYGGCGGNANRFKTMAECKWSCA | 1 |
| DFVSICEMPEDPGPCRGRLPRWFYDPLDRQCRAFYWSGCQGNENNFLSVQECQQTCM | 1 |
| DFVSICEMPEDPGPCRGRLPRWFYDPLDRQCRAFYWSGCQGNENNFLSVQECQQTCM | 1 |
| QCLPQYHPCIIGNEECCPHLECKCLPKP | 0 |
| EEPCKERRQPCERNEECCNDSCNRFYC | 1 |
| ARDCKGWLEECEEENCCDPYECIGFC | 1 |
| QMMCTTDEDCPTGQECCPDNINDPQGFCVDDCIV | 0 |
| VVTEACEESCEDEEKTCCGLENGEPVCARFCL | 1 |
| QMMCSSNEDCPAGQECCPDNLGKPEGFCVDDCII | 0 |
| QCSPNGGYCTLHIHCCSNHCIKPIGRCVA | 0 |
| ECLPKGAGCDEPFECCSHLCTGRLHTTC | 1 |
| SCKERASSCETPSECCSGVCRTRIFYLC | 1 |
| CDAPNAPCEKFDNDCCDACMLREKQQPICAV | 1 |
| TCRSSGRYCRSPYDCCRRYCRRITDACV | 1 |
| CKGKGAGCDYSHECCSRQCTGRIFQTCN | 1 |
| GCLDPGYFCGTPFLGAYCCGGICLIVCIET | 1 |
| CVGSCDLNNPCSSGCICNGDKC | 0 |
| SFKLVCGKGCKSRPDCPTECFCYMDMC | 1 |
| QKELVPSTITTCCGHEPGTMCPKCMCDNTCPPKKKKRP | 1 |
| ECRRRGQGCTQSTPCCDGLRCDGQRQGGMCVDS | 1 |
| TCRERGIGCDDPLECCSHQCTFTGFSTC | 1 |
| FSECSTRGCTFSSHCCEGLRCQGTPQGGVCV | 1 |
| CIKDGEFCGTPVENDPLCCSLICDLVCL | 0 |
| QKELVPSTITTCCGNEPGTMCPKCMCDNTCPPQKEEKTRPQ | 1 |
| QKELVPSTITTCCGNEPGTMCPKCMCDNTCPPKRRKTS | 1 |
| QKELVPSTITTCCGNEPGTMCPKCMCDNTCPPKRRKTRPQ | 1 |
| QKELVPSTITTCCGNEPGTMCPKCMCDNTCPPKRRKT | 1 |
| QKELVPSTITTCCGNEPGTMCPKCMCDNTCPPKRKKRP | 1 |
| QKELVPSTITTCCGNEPGTMCPKCMCDNTCPPKREKTS | 1 |
| KEIIVPALRQNTNSDEEDCCIYPWCDGDCLCC | 0 |
| HAEHMQDDNSAAQNPWVIAIRQCCTSCNFGCQPCC | 0 |
| GCCTGQGCWNVPICECCV | 0 |
| QKELVPSTITTCCGNEPGTMCPKCMCDNTCPPKREKTRPQ | 1 |
| QKELVPSTITTCCGNEPGTMCPKCMCDNTCPPKNEEKTRPQ | 1 |
| QKELVPSTITTCCGNEPGTMCPKCMCDNTCPPKKKKRPRPQ | 1 |
| QKELVPSTITTCCGNEPGTMCPKCMCDNTCPPKKKDT | 1 |
| QKELVPSTITTCCGNEPGTMCPKCMCDNTCPPKKEEKTRPQ | 1 |
| QKELVVTATTTCCGYNPMSMCPKCMCTYSCPHQKKKRP | 1 |
| GCLDPGYFCGTPFLGAYCCGGICLIVCIET | 1 |
| CSRLMEPCTQHPQCCSNTCSKFTTKCIS | 1 |
| MLKTGVLLLIFLVLFPLATLQDADQPVERNVENKQDLNLDKRRGMKLLAQRQQECCDPDWCDGACYNCC | 1 |
| QKELVVTATTTCCGYNPMSMCPKCMCTYSCPHQQKKRP | 1 |
| QKELVVTATTTCCGYNPMSMCPKCMCTYSCPHQQRKRP | 1 |
| QKELVVTATTTCCGYNPMTMCPKCMCNYSCPHQKKKRP | 1 |
| GCCGKYPNAACHPCGCTVGRPPYCDRPSGGX | 0 |
| DDCIKPYGFCSLPILKNGLCCSGACVGVCADLX | 0 |
| EACYPPGTFCGIKPGLCCSELCLPAVCVGX | 0 |
| GRCCEGPNGCSSRWCKDHARCCX | 0 |
| CCSQDCRVCIPCCPY | 0 |
| CCSTNCAVCIPCCP | 0 |
| CAKLGASCRYRRCCPDLICIGGHVGRACRYPENYYYYY | 1 |
| GCCGPYPNAACHPCGCKVGRPPYCDRPSGGX | 0 |
| CQGRGASCRRTSYDCCTGSCRSGRC | 1 |
| CQGRGASCRKTSYDCCTGSCRSGRC | 1 |
| CQGRGASCRKTMYNCCSGSCRSGRC | 1 |
| CKSTGASCRRTPYDCCTGSCRSGRC | 1 |
| CKGKGASCRRTSYGCCTGSCRSGRC | 1 |
| ASFAWVGAHECYSTGTFCGFNGGLCCSNLCLFSCA | 1 |
| ASFAWVGAHECYSTGTFCGFNGGLCCSNLCLFFVCLTFS | 1 |
| CCLWPECGGCVCCY | 0 |
| CKSTGASCRRTSYDCCTGSCDRGRC | 1 |
| SAYAAWQCFLPKEDGLCKGKFSRYYFDSALRMCRRFIYGGCGGNGNRFITKDACLNTCLY | 1 |
| SAYAAWQCFLPKKEDGLCKGKFSRYYFDSALRMCRWFIYGGCGGNGNRFITKDACLNTCLY | 1 |
| QCTPHGGSCGLVSTCCGRCSVPRNKCE | 1 |
| MFTVFLLVVLATTVVSIPSDRASDVRNAAVHERQKELVVTATTTCCGYNPMSMCPKCMCTYSCPHQKKKRPGRRND | 1 |
| MFTVFLLVGLATTLVSIPSDGASDGKNAAVHERQKELVPSTITTCCGHEPGTMCPKCMCDNTCPPKKKKRPGRRND | 1 |
| MGMRMMFIVFLLVVLATTVVSFTSDRASDDRNTNDKASRLLSHVVRGCCGKYPNAACHPCGCTVGRPPYCDRPSGGGR | 1 |
| MFTVFLLVGLATTLVSIPSDGASDGKNAAVHERQKELVPSTITTCCGNEPGTMCPKCMCDNTCPPKKKKRPGRRND | 1 |
| CKGKGASCRRTSYDCCTGSCRLGRCX | 0 |
| DTVPGLSALTVDDDTVPDVCRQPLEVGPCKAAYPRYYYNHASDTCQLFYYGGCNGNENRFEDFSGCLFTCIYPWMAALGY | 1 |
| YGCSNAGAFCGIHPGLCCSELCLVWCT | 0 |
| CKGKGASCRRTSYDCCTGSCRSGRCX | 0 |
| CKSKGAKCSKLMYDCCSGSCSGTVGRCX | 0 |
| CKGKGQSCSKLMYDCCTGSCSRRGKCX | 0 |
| DCCGVKLDMCHPCLCNNSCKQGQGKKRVWEMMKATD | 1 |
| CKGKGASCRKTMYDCCRGSCRSGRCX | 0 |
| MRCLPVFIILLLLIPSAPCVDAQPKTKDDMLLASFHDNAKRTLQRFWKKRDCCPEKMWCCPLG | 0 |
| MRCLPVFVILLLLIPSAPCVDAQLKTKDDMPLASFHDNAKRTLQRFWKKRDCCPEKMWCCPLG | 0 |
| CKSTGASCRRTSYDCCTGSCRSGRCX | 0 |
| SAYAAWQCFLPKKEDGLCKSKFSRYYFDSALRMCRRFIYGGCGGNGNRFITKDACLNTCLY | 1 |
| ATSNRPCKPKGRKCFPHQKDCCNKTCTRSKCP | 1 |
| CCSKYCWECTPCCPYSS | 1 |
| CLGFGEACLMLYSDCCSYCVALVCL | 1 |
| QINCCPWPCPDSCHYQCCH | 1 |
| CTPCGPDLCCEPGTTCDRVLHHTHFGEPSCSY | 1 |
| ASEGCAKVGAPCRYRRCCRDLICIGGHVGRRCRYPDSYYYY | 1 |
| ASEWCGELGDGCYTLPCCRDLKCVVGHEGRVCQYPSRHSFGI | 1 |
| CKSKGAPCRRTSYDCCTGSCRSGRC | 1 |
| CKGRGGACTKAMFICCSGSCNRGRC | 1 |
| CATYGKPCGIQNDCCNTCDPARKTCT | 1 |
| YGCSNAGAFCGIHPGLCCSELCLGWCT | 1 |
| MLKMRVVLFTFRVLFPLATLQLDADQPRARYAENKQDFNRNERTKMILSAVSASMGRQRRCCLWPECGGCVCCY | 1 |
| ASEGCGEIGAPCRDRRCCPDLRCVDGHVGRACQEYMDY | 1 |
| MLKMRVVLFTFLVLFPLATLQLDADQPRARYAENKQDFNRNERTKMILSAVSASMGRQRRCCLWPECGGCVCCY | 1 |
| MGKRMVLTMFLLVVLATTVVSFTLDHASDSRGTAANDKATDQMALAVRLDWCDDADFLVDHPELCGWDVCCAYPPCRHKHQDCNQGR | 0 |
| MHTLEMLLLLLLLLPLAPGEGDGQAVAGDRNPGEARSTHKRFLQRPARRIHRHRCTPCGPDLCCEPGTTCDRVLHHTHFGEPSCSY | 1 |
| MHTLEMLLLLLLLLPLAPGEGDGQAVAGDRNPGEARSTHKRFLQRPARRIHRHRCTPCGPDLCCEPGTTCDTVLHHTHFGEPSCSY | 1 |
| MHTLEMLMFFVLMLLMALGEGDGQDVAGDRNLGEARSTHKRFLQRPARRIHRHRCTPCGPDLCCEPGTTCDTVLHHTHFGEPSCSY | 1 |
| MHTLEMLLLLLLLLPLAPGEGDGQAVAGDRNPGEARSTHKRFLQRPARRIHRHRCTPCGPDLCCEPGTTCDTVLHHTRFGEPSCSY | 1 |
| MHTLEMLLLLLLLLPLAPGEGDGQAVAGDRNPGEARSTHKRFLQRPARRIHRHRCTPCGPDLCCEPGTTCDTVLRHTHFGEPSCSY | 1 |
| MEKLTSLLLVAALLMLTQTLIQGGREGRPKKFLQKIKSTAKRECTAPSGYCDYPEECCEVECGRHYCDWWY | 1 |
| MMSKLGVLLTISLLLFPLTAVQLDGEQPVDLLALRTQDFAPEQSPWFDPVKRCCSKYCWECTPCCPYSSG | 0 |
| ASEGCGEIGAPCRDRRCCPDLRCVDGPVGRACQEYMDY | 1 |
| ASDSCRKLGERCASHRCCPRLRCGSGRAGGVCRYPYN | 1 |
| SSEGCGEIGAPCRDRRCCPDLRCVDGHVGRACQEYMDY | 1 |
| CTPCGPDLCCEPGTTCDTVLHHTHFGEPSCSY | 1 |
| CTPCGPDLCCEPGTTCDTVLHHTRFGEPSCSY | 1 |
| CTPCGPDLCCEPGTTCDTVLRHTHFGEPSCSY | 1 |
| SCGHSGAGCYSRPCCPGLHCSGTHAGGMCV | 1 |
| SCGHSGAGCYTRPCCPGLHCSGGQAGGLCV | 1 |
| MRNPKLSKLTKTCDPPGDSCSRWYNHCCSKLCTSRNSGPTCSRP | 1 |
| CYDVGDFCGIPFIKNGNCCSQFCVFVCTPEW | 0 |
| ECTAPSGYCDYPEECCEVECGRHYCDWWY | 1 |
| MMSKLGVLLTICLLLFPLTAVQLDGEQPVDLLALRTQDFAPEQSPWFDPVKRGCSMYCWKCFPCCPNG | 0 |
| NCVTKGGYCNEAENISCCTTLTCYAGSQHDSNPTCMTVLS | 1 |
| CQGRGASCRKTMYNCCSGSCNRGRC | 1 |
| KSTAESWWEGECLGWSNGCTQPSDCCSNKCDTYCELW | 1 |
| KSTAESWWEGECRTWNAPCSFTSQCCFGRCAHHRCIAW | 1 |
| KSTAESWWEGECRTWNAPCRLTSQCCFGRCAHHRCIAW | 1 |
| GWGGECRTWNAPCSFTSQCCFGRCANRRCIAW | 1 |
| DGCYNAGTFCGIRPGLCCSEFCFLWCITFVDSX | 0 |
| GMWGECSDWLATCISPSQCCSESCDKRCLAW | 1 |
| LDWCDDADFLVDHPELCGWDVCCAYPPCRHKHQDCNQ | 1 |
| MMLKMGVVLFVFLVLFPLATLQLDADQPVERYAENKQDLHPNERTGFILPAMRRQGCCIEPLCYQYDCDCCRYL | 0 |
| CVDPGEFCGPGFGDCCTGFCLLVCI | 1 |
| CSPPGSYCFGPAACCSNFCSTLSDVCQESWSG | 0 |
| FPCNPGGCACRPLDSYSYTCQSPSSSTANCEGNECVSEADW | 1 |
| GECDGKKDCITNDDCTGCLCSDFGSYRKCA | 1 |
| VCCSFGSCDSLCQCCDX | 0 |
| GQGGCVPPGGGRCKANQACTKGGNPGTCGFQYDLCLCLRN | 1 |
| SCLSSGALCGIDSNCCNGCNVPRNQCYX | 0 |
| DQCSYCGIYCCPPKFCTSAGCRSPX | 0 |
| TFEPNAEECIVDGRCKHRSDWPCEMSSGTTGRCDVSLGACGCSN | 1 |
| GCTPRNGACGYHSHCCSNFCHTWANVCL | 1 |
| GCTPRNGFCRYHSHCCSNFCHTWAIMCL | 0 |
| GCTPRNGFCRYHSDCCSNFCHTWAIMCL | 0 |
| CSPGGEVCTRHSPCCTGFLCNHIGGMCHP | 0 |
| VCCPFGECKSLCYCCGA | 0 |
| CCHRNWCDHLCSCCGS | 1 |
| CSPGGEVCTRHSPCCTGFLCNHIGGMCHH | 0 |
| CSPGGEVCTSKSPCCTGFLCSHIGGMCHH | 0 |
| TCSPAGEVCTSKSPCCTGFLCTHIGGMCHH | 0 |
| TCSPAGEVCTSKSPCCTGFLCSHIGGMCHH | 0 |
| CPNTGELCDVVEQNCCYTYCFIVVCL | 0 |
| ECCGSFACRFGCVPCCV | 1 |
| SKQCCHLPACRFGCTPCCW | 0 |
| MGCCPFPCKTSCTTLCCX | 0 |
| CTTMNCLKGHCGCSPDCGSC | 1 |
| CCHWNWCDHLCSCCGS | 1 |
| EDDRTFPCSSGLCACLPLDSYSYICLSPSSSTANCENDECISEDDW | 0 |
| EDDRTFPCNSGRCACQPLDSYSYTCQSPSSSTANCKNNVCVSEADW | 1 |
| EACYNAGTFCGIKPGLCCSAICLSFVCISFDLIDVFSSP | 0 |
| CKQADEPCDVFSLECCTGICLGFCTW | 0 |
| APELVVTATTTCCGYDPMTICPPCMCTHSCPPKGKP | 0 |
| APELVVTATTTCCGFDPMTWCPPCMCTYSCSHQRKKP | 0 |
| EDDGFPCNAGNCACLPLDSYSYTCQSPTSSTANCEGNECVSEADW | 1 |
| EDDRFPCNAGNCACLPLDSYSYTCQSPTSSTANCEGNECVSEADW | 1 |
| STRCQGRGASCRKTMYNCCSGSCNRGRCG | 1 |
| TRCKGKGAPCRKTMYDCCSGSCGRRGKCG | 1 |
| CINVGMCIYDGYCCSNRCWGGMCSPWR | 0 |
| GCGYLGEPCCVAPKRAYCHGDLECNSVAMCVN | 1 |
| IPYCGQTGAECYSWCIKQDLSKDWCCDFVKDIRMNPPADKCP | 1 |
| GCCTPPRKCKDRACKPARCCGP | 1 |
| MGMRMMFTVFLLVVLATTVVSIPSDRASDGRNAVVHERAPELVVTATTNCCGYNPMTICPPCMCTYSCPPKRKPGRRND | 0 |
| SKQCCHLAACRFRCTPCCW | 1 |
| CCRIACNLKCNPCCX | 0 |
| EDDRFPCNAGNCACLPLDSYSYTCQSPTSSTANCEGNECRSEADW | 1 |
| LCCWKEWCHARCTCCX | 0 |
| MAKLTVLLLVAAVLLSTQVLVQGDGETPQRARFFTARKFSGVNKKGCDPKWTICNNDAECCFPYSCENSNCQ | 1 |
| MFTVFLLVVLATSVVSIPSDRASDGGNAVVHERAPELVVTATTTCCGYDPMTICPPCMCTHSCPPKGKPGRRND | 0 |
| EDDRFPCNAGNCACLPLDSYSYTCLSPTSSTANCESDECVSEDDW | 1 |
| RDCQEKWEYCIVPILGFVYCCPGLICGPFVCV | 0 |
| LCCWIHWCHARCTCCX | 0 |
| SEQCCHLAACRFGCTPCCW | 0 |
| CFGRGGLCTWFDPSVCCSGICTFVDCW | 0 |
| MKLMLSALRQQTCCEPSTCDGGCYHCC | 1 |
| SCAQSGLSCDTRPCCDDKPCVPNGRQSMCG | 1 |
| GCKATWMSCSSGWECCSMSCDMYCX | 0 |
| DCLPIGSLCHSSEQCCSGWCSPKRVC | 0 |
| SCDQTGEPCVLNEQCCYGWCTNHGTCY | 1 |
| SCVPIGRPCASNEQCCTRWCTPRRIC | 1 |
| TTAESWWEGECLGWSNGCTHPSDCCSNYCKGIYCDL | 1 |
| ACRQKWEYCIVPILGFVYCCPGLICGPFVCV | 0 |
| ACRKKWEYCIVPIIGFIYCCPGLICGPFVLRLIGMSSTPICATPGLIFD | 0 |
| CPNTGELCDVVEQNCCYTYCFIVVCPI | 0 |
| ECLEADYYCVLPFVGNGMCCSGICVFVCIAQAY | 0 |
| ECLEADYYCVLPFVGNGMCCSGICVFVCNTT | 0 |
| CIDGGEMCDPFSSDCCSGWCIFFFCT | 0 |
| STDCNGVPCQFGCCVTINGNDECRELDC | 1 |
| NPKLSKLTKTCDPPGDSCSRWYNHCCSKLCTSRNSGPTCSRP | 1 |
| TTDEKCNEYCEERDRNCCGKANGEPRCARMCF | 1 |
| ECTAPSGYCDYPEECCEVECGRHYCDWWY | 1 |
| STDCNGVPCEFGCCVTINGNDECREIGCE | 1 |
| STDCNGVPCQFGCCVTINGNDECRDLIVSDLTRRGLLDNEGHCPAATES | 1 |
| SCGHSGAGCYTRPCCPGLHCSGGHAGGLCV | 1 |
| DVCELPFEEGPCFAAIRVYAYNAETGDCEQLTYGGCEGNGNRFATLEDCDNTCARY | 1 |
| DVCELPFEEGPCFAAIRVYAYNAETGNCEQLTYGGCEGNGNRFATLEDCDNACARY | 1 |
| ATPECSRAGCKNVPCCSGLKCTGPQNGPVCQPE | 1 |
| CLDAGEMCDLFNSKCCSGWCIILFCA | 0 |
| GCGYLGEPCCVAPKRAYCHGDLECNNVAMCVN | 1 |
| DDKSNCPISHPNYCSFTPVCCKHECLSNNKCSSSEFIPGQ | 1 |
| GDACSLLNGDDCGPGELCCTPSGDHQGTCETSCW | 1 |
| MKLMLSALRQQECCKPSTCDGGCYHCC | 1 |
| TKMMLPALRQQPCCSPSTCDGGCYHCC | 1 |
| MKMMLPALRQQPCCSPSTCDGGCYHCC | 1 |
| RCCISPACHKDCYCCL | 0 |
| CCFWPDCRGCYCCL | 1 |
| CCLWPECGGCVCCY | 0 |
| CCFWPACWGCVCCY | 0 |
| CCLWPACWGCVCCY | 0 |
| CCIWPECGSCVCCL | 0 |
| CNNRGGGCSQHPHCCSGTCNKTFGVCL | 1 |
| CCLWPECGGCVCCYL | 1 |
| CCSRDCWVCIPCCPNGSA | 0 |
| CCFWPMCRGCDCCYL | 0 |
| CCFWPMCGGCDCCYL | 0 |
| DACSLLNGDDCGPGELCCTPSGDHQGTCETSCW | 1 |
| QWPFQQWAPCTGHWDCPGDRCCFAGYCLETTPSCD | 1 |
| QGQSQFGEQCTGHLDCFGDLCCFDGYCIMTSWIWPCNW | 0 |
| SLCDKPHHNCIDGQTCYHTCCQNGLKCVRYP | 1 |
| GWWGPPSNCWVCTGFNKCCEHESHCMTFPTQYNREC | 1 |
| DVCELPFEEGPCFAAIRVYAYNAETGDCEQLTYGGCEGNGNRFATLEDCDNACARY | 1 |
| DGEEFPCAGTMADCRGLADNSVCCDTGKCIGEVCYY | 0 |
| CCSRHCWVCIPCCPNGSA | 0 |
| GCDPKWTICNNDAECCFPYSCENSNCQ | 1 |
| CNNRGGGCSQHPHCCSGTCNKIFGVCL | 1 |
| CKGKGAGCDYSHECCSRQCTGRIFQTCN | 1 |
| CIDGGEMCDPFSSDCCSGWCIFFFCI | 0 |
| ECREKGQGCTNTALCCPGLECEGQSQGGLCVDN | 1 |
| SQTQRLSKKCIEDNHACGLLHHSPYCCNGTCFIVCIP | 0 |
| DCCPLPACPFGCNPCCGWPALLSGPHQVMNNE | 0 |
| SLDTREICWHQSECDDPNEWCCIMGTSYGSCQPVCRP | 1 |
| GFILHALWHVQCCSWLWCDGGCDCCE | 0 |
| CCHWNWCDHLCLCCGS | 1 |
| CLESGSLCFAGYGHSSCCSGACLDYGGLGVGACR | 1 |
| ECLEADYYCVLPFVGNGMCCSGICVFVCIAQKY | 0 |
| CLDGGEICGILFPSCCSGWCIVLVCA | 0 |
| EATNRATKRGCLMCWGSNVRCCEKANACVSINYECPKARR | 1 |
| CRPPGMVCGFPKPGPYCCSGWCFAVCLPV | 0 |
| DDECEPPGDFCGFFKIGPPCCSGWCFLWCA | 0 |
| QCEDVWMPCTSNWECCSLDCEMYCTQIX | 0 |
| YCVPKSGLCTIFQPGKCCSGWCLIYRCT | 0 |
| DDCTTYCYGVHCCPPAFKCAASGCVRNN | 0 |
| ETGCKKDGSWCWIPSECCIESCLITCWY | 0 |
| QLKKLRRECYLEPGDSCFHDDGRGACCEGTCLFGINCVASW | 1 |
| CKSGGQACWFLLKKHNCCSGYCIVAVCAG | 0 |
| QLKKLRRECYLEPGDSCFHDDGRGACCEGTCFFGVACVPWS | 1 |
| ECSRKGEWCGLESVLCCNGGSWNCWFVCTA | 0 |
| SLDTREICWNHSECDDPSEWCCRMGSGHGSCQPVCRP | 1 |
| DDECEPPGDFCGFFKIGPPCCSGWCFLWCDLKLP | 0 |
| ECLEADYYCVLPFVGNGMCCSGICVFCLHSPKVLKLS | 1 |
| SLDTREICWNHSECDDPSEWCCRMGSGHGSCLPVCRP | 1 |
| CIDGGEICDYFFPNCCSGWCIILVCA | 0 |
| CIDGGEICDIFFLNCCSGWCIILVCA | 0 |
| CIDGGEMCDPFSSDCCSGWCIFFCI | 0 |
| STLGQRYCKASGSWCGIHKHRECCSGNCFFWCVYNGK | 1 |
| TRDCKTKGYACFASTECCVQDCWLVCLY | 1 |
| DCHSTGYLCFWWHECCSNFCIPLQQRCF | 0 |
| DCKPKNNLCLWSSECCSGICFPFAQRCT | 0 |
| CLDAGEMCDSFNSKCCSGWCIILVCA | 0 |
| SSCWFCSTGFNKCCESTGDCMTYPSEYNASCPEA | 1 |
| EAEATNRAIKRGWFGEESSCWWCTGFNKCCEAAAVCQSVNSACP | 1 |
| EADANRLSTRWCACGVNYYCCNEVCTWREDPCP | 1 |
| CRSPGSWCFYKHSNCCSGNCFLWCVQNGK | 1 |
| QLKKLRRECYLEPGDSCFHHDGRGACCEGTCFFGVACVPW | 1 |
| VCCPFGGCHELCYCCY | 1 |
| VCCSFGSCDNLCQCCD | 0 |
| VCCRKEWCHARCTCC | 1 |
| KANNAEAPEQEKRACTPNGSYCNILSGKLNCCSGWCLALICAG | 1 |
| VCCPFGGCHEFCLCCD | 1 |
| SDCDLYDDSCTGTEICCTPPGDYQGNCMEGEDCPSGGR | 1 |
| GWDTPAPCRYCQWNGPQCCVYYCSSCNYEEAREEGHYVSSHLLERQ | 1 |
| GWDTPAPCHYCQWKGPQCCVYYCSSCNYEEAREEGHYVSSHLLERQ | 1 |
| AKECSMYYCSGGDFCCPGLKCGDPTGKKICIEPGK | 1 |
| CLNGGEICGILFPSCCSGWCIVLVCA | 0 |
| DVCELPFEEGPCFAAIRVYAYNAKTGDCEQLTYGGCEGNGNRFATLEDCDNACARY | 1 |
| TNRVDCSAPEDKSEPGYWCGLEPLCCYSGKCFVICFGSKPAGT | 0 |
| MLKMGVVLFVFLVLFPMATLQLDADQPVERYAENKQDHNADERMGLILPALRQRTCCVRPWCDGACDCCVDP | 0 |
| CKGKGAKCSRLMYDCCTGSCRSGKC | 1 |
| EACYNAGTFCGIKPGLCCSAICLSFVCISFDF | 0 |
| ECLEADYYCVLPFVGNGMCCSGICVFVCIDPKVLKLS | 0 |
| ECIEGSEPCEVFRPYTCCSGHCIIFVCA | 0 |
| ECLEADYYCVLPFVGNWMCCSGICVFVCIAQKY | 0 |
| CIAGGQPCEFHRGYMCCSEHCIIFVCA | 0 |
| CIGGGDPCEFHRPYTCCSGYCIVFVCA | 0 |
| CIGGGDPCEFHRGYTCCSEHCIIWVCA | 0 |
| DECYPPGTFCGIKPGLCCSAICLSFVCISFDF | 0 |
| CLAGSAPCEFHRGYTCCSGHCLIWVCA | 0 |
| CLAGSAPCEFHKRSTCCSGHCIIWWCA | 0 |
| CLAGSARCEFHKPSTCCSGHCIFWWCA | 0 |
| EACYNAGSFCGIHPGLCCSEFCILWCITFVDSX | 0 |
| DGCYNAGTFCGIRPGLCCSEFCFLWCITFVDSX | 0 |
| CLAGSARCEFHKPSSCCSGHCIFWWCA | 0 |
| APELVVTATTNCCGYNPMTICPPCMCTYSCPPKRKPX | 0 |
| QKCCSGGSCPLYFRDRLICPCC | 0 |
| QGCCNVPNGCSGRWCRDHAQCCX | 0 |
| ECSESGEWCGLDPALCCGSSCFFTCN | 0 |
| YCSDSGGWCGLDPELCCNSSCFVLC | 1 |
| TRDCKTKGSVCFASSECCIQDCWFVCLY | 0 |
| CKGKGASCHRTSYDCCTGSCNRGKCX | 0 |
| CKGKGAPCRKTMYDCCSGSCGRRGKCX | 0 |
| ECLEADYYCVLPFVGNGMCCSGICVFVCIAQKVLKLS | 0 |
| CQGRGASCRKTMYNCCSGSCNRGRCX | 0 |
| DDECEPPGDFCGFFKIGPPCCSGWCFLWCV | 0 |
| GWFGEESSCWWCTGQNKCCEEAQVCQSVNYACPPARR | 1 |
| DDCTTYCYGVHCCPPAFKCAASPSCKQT | 0 |
| GCWLCLGPNACCRGSVCHDYCPR | 0 |
| GCWLCLGPNACCRGSVCHDYCPS | 0 |
| DDECEPPGDFCGFLKLGPPCCSGWCFLWCA | 1 |
| DDECEPPGDFCGLFKIGPPCCSGWCFLWCA | 0 |
| NCIPKNHFCGLLHHSRNCCTPTCLIVCF | 0 |
| DDECEPPGVFCGTFKIGPPCCSGWCFLWCA | 0 |
| GLSRPSKGCIGGGDPCEFHRGYTCCSEHCIIWVCA | 1 |
| DDECEPPGDFCGFFKIGPPCCSGWCFLWCS | 0 |
| ACRKKWEYCIVPILGFVYCCPGLICGPFVCV | 0 |
| CIPDHHGCGLLHHSRYCCNGTCFFVCIP | 1 |
| DDECEPPGDFCGFFKIGPPCCSGWCFLLGCA | 0 |
| DDECEPPGDFCGFFKIGPPCCSGWCFLWGCA | 0 |
| TRGCKSKGSFCWNGIECCGGNCFFACIY | 1 |
| DDECEPPGDFCGFFKNWVRLCCSGWCFLWCA | 1 |
| DDECEPPGDFCGTFLKIGPPCCSGWCFLWCA | 0 |
| TRGCKSKGSFCWNGIECCGGNCFFACVY | 1 |
| DDECEPPGVFCGTFKIGPPCCSGWCFLWCDLKLP | 0 |
| ECLEADYYCVLPFVGNGMCCSGICVFVCMAQKY | 0 |
| ECLEADYYCVLPFVSNGMCCSGICVFVCIAQKY | 0 |
| GCWICWGPNACCRGSVCHDYCPS | 0 |
| ECREQSQGCTNTSPPCCSGLRCSGQSQGGVCISN | 1 |
| MFTVFLLVVLATTVVSIPSDRASDGRNAVVHERAPELVVTATTTCCGFDPMTWCPPCMCTYSCSHQRKKPGRRND | 0 |
| GCCIEPLCYQYDCDCCRHL | 1 |
| GCCIEPLCYQYDCDCCRYL | 1 |
| CCDDSECSYSCWPCCY | 1 |
| CCEAPFCNAGCTCCNP | 0 |
| QCEDVWMPCTSSHWECCSLDCEMYCTQI | 0 |
| GCCGSFACRFGCVPCCV | 1 |
| SKQCCHLAACRFGCTPCCW | 0 |
| DCGPWCWGQNKCCPDESCRSLHESCT | 1 |
| TCCVRPWCDGACDCCVDP | 0 |
| QCCGWEWCDDICGCCE | 0 |
| IEEDCGYVPCEFGCCRIIDGKEKCREIDCQ | 1 |
| CCAPSACRLGCRPCCR | 1 |
| QCEDVWMPCTSNWECCSLDCERYCTQIX | 0 |
| EFILPAVGRTCCDLTWCDGNCRCCTP | 0 |
| RCCTWQECDGNCRCCQ | 1 |
| RCCIWPECGSCVCCL | 0 |
| RCDEEGTGCSSDSECCSGRCTPEGLFEFCE | 1 |
| CIDGGEICDIFFPNCCSGWCIILVCA | 0 |
| RCCISPACHDECICCID | 0 |
| CCDRPCSIGCVPCCLP | 0 |
| CPNTGELCDVVEQNCCYTYCFIVVCL | 0 |
| CCRLSCGLGCHPCCX | 0 |
| NSASLISSWVDNTNFCCCSHDCATICDDCF | 0 |
| DDFMRIMCGDEFCTYDCCEIVDGSSKCKQPDCP | 0 |
| CLDAGEMCDLFNSKCCSGWCIILVCA | 0 |
| VCCPFGGCHELCYCCDX | 0 |
| QCCDWPWCDDCICCD | 0 |
| TRGCKTKGTWCWASRECCLKDCLFVCVY | 1 |
| RCCTWQECDGNCHCCQ | 1 |
| MMSKLGVLLTICLLLFPLTAVQLDGDQPVDLPALRTQDFAPEHSPWFDPVKRCCSRDCWVCIPCCPNGSA | 0 |
| MKLTCMMIVAVLFLTAWTFVTADDSGNGMEILFPKAGHEMENLEVSNRVKPCRKEGQLCDPIFQNCCRGWNCVLFCV | 0 |
| MMSKLGVLLTVCPLLFPLTALPPDGDQPADRPAERMQDDISSDEHPLFDKRQNCCNGGCSSKWCRDHARCCGR | 1 |
| MKLTCMMIVAVLFLTAWTLVMADDSNNGLANLFSKSRDEMEDPEASKLEKRDCQALWDYCPVPLLSSGDCCYGLICGPFVCIGW | 0 |
| MNTAGRLLLLCLVLGLVFESLGIPVADDLEADRDTDPDEKDPSVHNYWRNVNCGGVPCKFGCCREDRCREIDCD | 1 |
| MKLTCMMIVAVLFLTAWTFVTAVPHSSNALENLYLKAHHEMNNPEDSELNKRCYDGGTGCDSGNQCCSGWCIFACL | 0 |
| MKLTCMMIVAVLFLTTWTFATAITRNGLGNLFPKNHHEMKNPEASKLNKRCVPYEGPCNWLTQNCCDELCVFFCL | 0 |
| VVIVAVLLLTACQLITADDSRGTQKHRTLRSKTKLSMSTRCKAAGKPCSRIAYNCCTGSCRSGKCG | 1 |
| VVIVAVLLLTACQLITAEDSRGTQKHRTLRSKTKLSMSTRCKAAGKPCSRIAYNCCTGSCRSGKCG | 1 |
| VVIVAVLLLTACQLITADDSRGTQKHRALRSDTKLSMSTRCKAAGKPCSRIAYNCCTGSCRSGKCG | 1 |
| MKLTCVMIVAVLFLTAWTFVTADDSKNGLENHFWKARDEMKNREASKLDKKDDCIKPYGFCSLPILKNGLCCSGACVGVCADLR | 0 |
| MKLTCVMIVAVLFLTAWTFVTADDSKNGLENHFWKARDEMKNREASKLDKKEACYPPGTFCGIKPGLCCSELCLPAVCVGG | 0 |
| MMSKLGVLLTVCLLLFPLTAVPMDGDQPADRPAERMQDDISSDEHPLFDKRQNCCNGGCSSKWCRDHARCCGR | 1 |
| MKLTCMMIVAVLFLTAWTFATADDPRNGLEKLFSNTHHEMKNPEASKLNKRCKQADESCNVFSLDCCTGLCLGFCVS | 0 |
| MSKLGVLLTICLLLFPLTAVPLDGDQPADRPAERMQDDISSERHPFFDRSKQCCHLPACRFGCTPCCW | 0 |
| MSKLGVLLTICLLLFPLTALPLDGDQPADQRAERTQAEKHSLPDPRMGCCPFPCKTSCTTLCCG | 1 |
| MMSKLGVLLTICLLLFPHTAVPLDGDQHADRPAERMQDDISSEHHPMFDAVKRREQNQFKSFTSVKLLDSRGERCCGPTACLAGCKPCCG | 1 |
| MMSKLGVLLTICLLLFPHTAVPLDGDQHADQPAERLQDDISSENNPMFNSIRRREQNQFKSFTSVKLLDSRGERCCGPTACLAGCKPCCG | 0 |
| MMSKLGVLLTICLLLFPHTAVPLDGDQPAERLQDDISSEHHPMFNSIRRREQNQFKSFTSVKLLDSRGERCCGPTACLAGCKPCCG | 0 |
| VLIIAVLFLTACQLTTAETSSRGKQKHRALRSTDKNSKMTRDCTPPGGACGFYYHCCSNYCVTISSTCN | 1 |
| VLIIAVLFLTACQLTTAETSSRGKQKHRALRSTDKNSRMTRDCTPPDGACGFHYHCCSKFCITISSTCN | 1 |
| VIIIAVLFLTACQLIATASYARSERKHPDLRLSGRNSKLSKRCLGSGEQCVRDTSCCSMSCTNNICF | 1 |
| MMSKLGVLLTICLLLFPLTAVPLDGDQPLDRHAERMHDGISPKRHPWFDPVKRCCKVQCESCTSCC | 0 |
| MMSKLGVLLTICMLLFPLAAFPLDGDQPADQPAERMQYDRLRAMNPWFDPIKRCRCEQTCGTCVPCC | 0 |
| MMSKLGALLTICLLLFSLTAVPLDGDQHADQPAQRLQDRIPTEDHPLFDLNKRCCDDSECSYSCWPCCYG | 0 |
| VLIIAVLFLTACQLTTAETSSRGKQKHRALRSTDKNSRLTRRCTPPGGSCGGHAHCCSKSCNIMASTCND | 1 |
| MMSKLGVLLTICLLLFPITGLPLDEDQHADLPALRAQAFEPEHSPWFDPVRRCCSQDCWECIPCCPN | 0 |
| VLIIAVLFLTACQLTTAETSSRGKQKHRALRSTDKNSRMTKRCTPSGGACYVASTCCSNACNLNSNKCV | 1 |
| IIAVLFLTACQLITAETYSRGEQKHRALSSTDKNSKLTRTCNTPTQYCTLHRHCCSLYCHKHPRMCIIPVSGHEPL | 0 |
| VLIIAVLFLTACQLTTAETSSRGKQKHRALRSTDKNSKMTRDCTPPGGACGFYYHCCSNYCITISSTCN | 1 |
| VVIVAVLLLTACQLITAEDSRGTQKHRALRSDTELSMSTRCKAAGKSCSRIAYNCCTGSCRSGKCG | 1 |
| VVIVAVLLLTACQLITAEDSRGTQKHRALRSDTKLSMSTRCKAAGKSCSRIAYNCCTGSCRSGKCG | 1 |
| VVIVAVLLLTACQLITADDSRGTQKHRALRSDTKLSMSTRCKAAGKSCSRIAYNCCTGSCRSGKCG | 1 |
| MMSKLGVLLTICMLLFPLAVFPLDGDQPADQPAERMQYDMLRAMNPWFDPIKRCRCEQTCGTCVPCC | 0 |
| MKLTCALIVTLLFLSITAGDSRGKHRYNALKSMSREANSTERECREKGQGCTNTALCCPGLECEGQSQGGLCVDN | 1 |
| MKLTCALIITLLFLSITAGDSRGKHRYNALKSMSREANSTERECREKGQGCTNTALCCPGLECEGQSQGGLCVDN | 1 |
| MKLTCVLIIAVLFLTAYQLATAASYAKGKQKHRALRPADKHLRLTKRCNDRGGGCSQHPHCCGGTCNKLIGVCL | 1 |
| VVIVAVLLLTACQLITAEDSRGTQKHRTLRSTARRSKSELTTRCRSSGSPCGVTGICCGRCYRGKCT | 1 |
| VVIVAVLFLTACQLITADDSRRTQKHRALRSTTKLSMLTRQCTPHGGSCGLVSTCCGRCSVPRNKCE | 1 |
| MKLTCMMIVAVLFLTAWTFVTADDSRNGLKNLFPKARHEMKNPDASKLNKRDGCSNAGGFCGIHPGLCCSEICLVWCT | 0 |
| MSRLGVLLTICLLLFPLTAVPLDGDQPADRPAERLQDDISSEHHPHFDSGRECCGSFACRFGCVPCCV | 0 |
| MLKWEWCYHLLVLFPLATLQLDADQPVERYAKNKQLFNPHKRRGIILRAPGKRCCHWNWCDHLCSCCGS | 0 |
| VVIVAVLLLTACQLITADDSRGTQKHRSLRSTTKVSKSTRCKAAGKSCSRIAYNCCTGSCRSGKCG | 1 |
| ECLEADYYCVLPFVGNGMCCSGICVFVCIAQKYKTVVTSSTPLCATWLDL | 0 |
| VVIVAVLLVTACQLITAEDSRGTQKHRTLRSTARRSKSELTTRCRSSGSPCGVTSICCGRCYRGKCT | 1 |
| VVIVAVLLLTACQLITADDSRGTQKHRSLRPTTKVSKSTSCMEAGSYCGSTTRICCGYCAYFGKKCIDYPSN | 1 |
| MKLTCMMIVAVLFLTAWTFVTAVPHSSDVLENLYLKALHETENHEASKLNVRDDECEPPGDFCGFFKIGPPCCSGWCFLWCA | 0 |
| MMFKLGVLLTICLVLFPLTALPLDGDQPADRPAERAQDDTSAAQNPRVDHVKRCCSICDTTCVYPTCCDDLISDK | 0 |
| MNTAGRLLLLCLALGLVFESLGIPVADDVKADRDTDPDDENPRDMSRLTLSAPKGKCNGAPCTFGCCRSVNGKPRCNEFDCG | 1 |
| MKLMCMMIVVVLFLTAWTFVTADDSRNGLENLFPKARLEMKNSEASRSRGRCRPRGMVCGFPKPGPYCCNGWCFFVCL | 0 |
| QVSWWCGKPGDTCGKLYLKCCSGRCSGSCSGKCLP | 1 |
| MKLTCMMIVAVLFLTAWTFVTAVPHSSDVLENLYLKALHETENHEASKLNVRDDECEPPGDFCGFFKIGPPCCSGWCFLWCA | 0 |
| MPSVRSVTCCCLLWMMLSVQLVTPGSPGTAQLSGHRTARFPRPRICNLACRAGIGYKYPFCHCRGKRDAVSSSMAV | 0 |
| GCCPPSLCDPGCDEGCCPVVTPAC | 0 |
| MPSVRSVTCCCLLWMMLSVQLVTPGSPGTAQLSGHRTARFPRPRICNLACRTGIGYKYPFCHCRGKRDAVSSSMAV | 0 |
| MKLTCVLIISVLFLTASQLITAVYSRDKQQYRAARLRDEMRNLKGARDCGEQGQGCYTRPCCPGLECRGGGTGGGVCQQ | 1 |
| MKLTCVLIISVLFLTASQLITAVYSRDKQQYRAARLRDEMRNLKGARDCGEQGQGCYTRPCCPGLGCRAGATGGGVCQQ | 1 |
| MKLTCMVIVAVLFLTAWTFVKADDSRNGLENLFPKARHEMKNSKASKLNKRCVEDGDFCGPGYEECCSGFCLYVCI | 0 |
| MMSKLGALLIICLLLFPLTAVPLDGDQPADRPAERMQNDISSEHHPMFDAIRGCCGAFACRFGCTPCC | 0 |
| AVLFLTACQLIAADDYRDLQEFPRRKMSDRMLNRKDMEKRCLAPQRWCSMHDDNCCKTCIILRCS | 1 |
| MMFKLGVLLTICLVLFPLTALPLDGDQPADRPAERMQDDTSAAQNPRVAFVERCCEADCPMCQDMLCCRK | 0 |
| MMSKLGVLLTICLLLFPITADPLVEDQPADRPADRMQDIATEQHPLFDPVKRCCDWPCGIGCIPCCLP | 0 |
| MKLTHVLIVAVLVLTVCHLTMAVCKSGGQACWFLLKKHNCCSGYCIVAVCAG | 0 |
| MKLTCMMIVAVLFLTAWTFATADDPRNGLENLFRKAHHEMKNPKDSKLNKRCLDAGEMCDLFNSKCCSGWCIILFCA | 0 |
| MKLTCMMIVAVLFLTAWTFATADDPRNGLENLFSKAHHEMKNPKDSKLNQRCLDAGEMCDLFNSKCCSGWCIILFCA | 0 |
| MKLTCMMIVAVLFLTAWTFATADDPRNGLENLFSKAHHGMKNPKDSKLNKRCLDAGEMCDLFNSKCCSGWCIILFCA | 0 |
| MYLLLGWVGGSGPYCSFMFMEHTFPAEWYQQGCRLYVGCVTVSCCLSYVSDCLRLLPLSLVLSLHCIGNW | 0 |
| MTSVQSVTCCCLLWLMLSVQPITPGSPGPAQLSRERSFKFLSGGFKEIVCHRYCAKGIAKEFCNCPDKRDVVSSRIRGRKRSKAM | 0 |
| MKLTCMMIVAVLFLTAWTFVTAVPHSSDALENLYLKALHETENHEASKLNVRDDECEPPGDFCGFFKIGPPCCSGWCFLWCA | 0 |
| MMSKLGVLLTICLLLFPLTALPMDGDEPADRPAERMQDNISSEQHPLFEERHGCCKGPKGCSSRECRPQHCCGRR | 1 |
| MPSVRSVTCCCLLWMMLSVQLVTPGSPGTAQLSGQRTARSSGSTVCKMMCRLGYGHLYPSCGCRGKRDVVSSSMAV | 0 |
| MPSVRSVTCCCLLWMMLSVQLVTPGSPGTAQLSGHRTARSPGSTICKMACRTGNGHKYPFCNCRGKRDVVSSSMAV | 0 |
| MKLTCMMIVAVLFLTAWTFVTAEDSRDARTPMCGATCAMACPNDFVEDEHGCPICKCREEKRTDETNLV | 0 |
| VVIVAVLLLTACQLITADDSRGTQKHRSLRSTTKVSKSTSCMEAGSYCGSTTRICCGYCAYFGKKCIDYPSN | 1 |
| MGVVLFIFLVLFPLATLQLDADQPVERYAKNKQLFNPHKRRESYCVRPGKRCCHWNWCDHLCSCCGS | 1 |
| VVIVAVLLLTACQLITAEDSRGTQKHRTLRSTARRSKSELATRCRSSGSPCGVTSICCGRCYRGKCT | 1 |
| VVIVAVLLLTACQLITAEDSRGTQKHRTLRSTARRSKSELTTRCRSSGSPCGVTSICCGRCYRGKCT | 1 |
| MKLTCMMIVAVLFLTAWTFATADDPRNGLGNIFSNAHHEMKNPEASKLNKRCRLGAESCDVISQNCCQGTCVFFCLP | 0 |
| VLIIAVLFLTACQLTTAETSSRGKQKHRALRSTDKNSKMTRDCTPPGGACGFYYHCCSNYCITISSTCR | 1 |
| MKLTCVMIVAVLFLTAWTFVMADDSGNGLENLFSKAHHEMKNPEASKLNKRCAQSSELCDALDSDCCSGVCMVFFCL | 0 |
| MKLTCVMIVAVLFLTTWTFVTADDSRYGLKNLFPKARHEMKNPEASKLNKRDGCSSGGTFCGIHPGLCCSEFCFLWCITFID | 0 |
| MKLTCMMIVAVLFLTTWTFVTADDSRYGLKNLFPKARHEMKNPEASKLNKRDGCSSGGTFCGIHPGLCCSEFCFLWCITFID | 0 |
| MKLTCVMIVAVLFLTAWTFVTADDSKNGLENHFWKARDEMKNREASKLDKKEACYPPGTFCGIKPGLCCSELCLPAVCVGG | 0 |
| MKLTCLMAVAVLFLTARTFVTADDSRNGLENLSPKARHEMKNPEASKSNKRYECYSTGTFCGINGGLCCSNLCLFFVCLTFS | 0 |
| MMSKLGVLLTICLLLFPLTAVQLDGDQPVDLPALRTQDFAPEHSPWFDPVKRCCSRHCWVCIPCCPNGSA | 0 |
| MGVVLFIFLVLFPLATLQLDADQPVERYAKNKQLFNPHKRRGIILCAPGKRCCHWNWCDHLCSCCGS | 0 |
| MMSKLGALLTICLLLFSLTAVPLDGDQHADQPAERLHDRLPTENHPLYDPVKRCCDDSECDYSCWPCCMFG | 0 |
| MKLTCALIITVLFLSITADDSRGKQGYRALKSIAGMLNSKTVRECREQSQGCTNTSPPCCSGLRCSGQSQGGVCISN | 1 |
| VLIIAVLFLTACQLITAETYSRGEQKHRALSSTDKNSKLTRTCNTPTRYCTLHRHCCSLHCHKTIHACA | 1 |
| MKLTCMLIIAVLFLTAWTFVTADDSGNGMENLFPKARHEMENLEDSKHRHQERPDTGDKEEMLLQRRVKPCSEEGQLCDPLSQNCCRGWHCVLVSCV | 1 |
| MKLTCLLIIAVLFLTAWTFVTADDSGNGMENLFPKARHEMENLEDSKHRHQERPDTGDKEEMLLQRQVKPCRKEHQLCDLIFQNCCRGWYCLLRPCI | 1 |
| MSKLGVVLCIFLVLFPLATLQLDADQPVERYAKNKQLFNPHKRRGIILRAPGKRCCHWNWCDHLCSCCGS | 0 |
| MMSKLGVLLTICLLLFPLTALPMDGDQPADRPAERMQDDISSERHPFFDRSEQCCHLAACRFGCTPCCW | 0 |
| MMSKLGVLLTICLLLFPLTALPMDGDEPANRPVERMQDNISSEQYPLFEKRRDCCTPPRKCKDRRCKPMKCCAGR | 1 |
| MSKLGVLLTICLLLFPLTALPMDGDEPANRPVERMQDNISSEQYPLFEKRRDCCTPPKKCKDRQCKPQRCCAGR | 1 |
| MKLTCMMIVAVLFLTAWTLVTADGTRDGLKNRFPKARLEMKNSEAPRSRGRCRPPGMVCGFPKPGPYCCSGWCFAVCLPV | 0 |
| CLGSREQCVRDTSCCSMSCTNNICC | 1 |
| MKLTCVMIVAVLFLTAWTFATADDPRNGLENLFLKAHHEMNPEASKLNERCLGGGEVCDIFFPQCCGYCILLFCT | 0 |
| VLIIAVLFLTACQLTTAVTSSRGKQKHRALRSTDKKFKVALLCSPPGSYCFGPAACCSNFCSTMSDVCQENWSG | 1 |
| MMSKLGALLTICLLLFSLTAVPLDGDQHADQPAQRLQDRIPTEDHPVRRCCDLPCNAGCVPCC | 0 |
| MSKLGVLLSICLLLFPLTALPMDEDQPADQLEDRMQDDISSEQYPSFVRRQKCCGEGSSCPKYFKNNFICGCC | 0 |
| MIRMGFFLTLTVAVLLTSLICTEAVPTDKRGMERLFDQVLLKDQRNCPYCVVYCCPPAYCQASGCRPP | 0 |
| VVIVAVLLLTACQLITADDSRGTQKHRALRSDTKLSMSTRCKGKGASCRRTSYDCCTGSCRSGRCG | 1 |
| VLIIAVLFLTACQLTTAVTSSRGEQKHRALRSTDKKFKVALLCSPPGSYCFGPAACCSNFCTTLSDVCQESWSG | 1 |
| VVIVAVLLLTACQLITANDSRGTQKHRALRSDTKLSMSTRCKSTGASCRRTSYDCCTGSCRSGRCG | 1 |
| VVIVAVLLLTACQLITANDSRGTQKHRALRSDTKLSMSARCKSTGASCRRTSYDCCTGSCRSGRCG | 1 |
| VVIVAVLLLTACQLITADDSRGTQKHRALRSDTKLSMSTRCKSTGASCRRTSYDCCTGSCRSGRCG | 1 |
| VVIVAVLLLTACQLITANDSRGTQKHRALRSDTKLSMSTRCQGRGASCRRTSYDCCTGSCRSGRCG | 1 |
| MMSKLGVLLTICVLLFPLTAVPLDGDQPADQPAERTQNEQHPLFDQKRGCCRWPCPSICGMARCCSS | 0 |
| MMYKFGVLLTICLLLFPLTAVQLDGDQPVDLPALRTQDFAPEHSPWFNPVKRCCSRHCWVCIPCCPNGS | 0 |
| MMSKLGVLLTICLLLFPITGLPLDEDQHDDLPALRAQDFEPEHSPWFDPVRRCCSQDCSVCIPCCPN | 0 |
| MKLTCVVFVAVPFLTASVFITADDSRNGIENLPRMRRHEMKNPKASKLNKRQCRVEGEICGMLFEAQCCDGWCFFVCM | 0 |
| MLKMGVMLFTFLVLFHLAAPQLDADQPVARYAGNKPDFNPNKRMKMMSALRKRGCCPPSLCDPGCDEGCCPVVTPAC | 0 |
| MLKMGVMLFTFLVQFHLAAPQLDADQPVARYAGNKPDFNPNKRMKMMSALRKRGCCPPSLCDPGCDEGCCPVVTPAC | 0 |
| MKLTYVLIIAVLFLTACQLITAETYSRGEQKHRALSSTDKNSKLTRTCNTPTQYCTLHRHCCSLYCHKTIHACA | 1 |
| MKLTCMMIVAVLFLTAWTFVTADDSGNGLENLFSKAHHEMKNPKDSKLNKRCLDGGEICGILFPSCCSGWCIVLVCA | 0 |
| SSTYDDEIATFCWSYWIGFQYSYPYTYVQPCTALGKACTANSDCCSKYCNTKICKINWE | 1 |
| MKLTCVMIVAVLFLTAWTFVTADSIRALEDLFAKAPDEMENSGASPLNERDCRPVGQYCGIPYEHNWRCCSQLCAIICVS | 0 |
| MKLTCMMIVAVLFLTAWTFVTADSIRALEDLFAKAPDEMENSRASPLNERDCRPVGQYCGIPYEHNWRCCSQLCAIICVS | 0 |
| KTSSTYDDEMATFCWSYWNEFQHSYPYTYVQPCLTLGKACTTNSDCCSKYCNTEMCKINWE | 1 |
| KTSSTYDDEMATFCWSYWNEFQYSYPYTYVQPCLTLGKACTTNSDCCSKYCNTKMCKINWE | 1 |
| MKVTCVLTLAVLILTIGQIANADSTLGQRYCKASGSWCGIHKHRECCSGNCFFWCVYNGK | 0 |
| MKLTCVMIVAVLFLTAWTLVMADDSNNGLANHFWKSRDEMEDPEASKLEKRDCQGEWEFCIVPVLGFVYCCPWLICGPFVCVDI | 0 |
| MSKLGALLIICLLLFPLTAVPMDGDQPADQPAERMQDDISSEHHPMFDAIRGCCGAFACRFGCTPCC | 0 |
| MKLTCMMFVAVLFLTASVFITADDSRNGIENLPRMRRHEMKNPKASKLNKRQCRVEGEICGMLFEAQCCDGWCFFVCM | 0 |
| MKLTCMMIVAVMFLTASIFITADNSRNGIENLPRMRRHEMKKPKASKLNKRVCIADDMPCGFGLFGGPLCCSGWCLFVCL | 0 |
| MKLTCMMIVAVLFLTAWIFITADNSRNGIENLPRMRRHEMKNPKASKLNKRGCREGGEFCGTLYEERCCSGWCFFVCV | 0 |
| MKLTCVMIVAVLFLTAWTFVTADDSRYGLKDLFPKERHEMKNPEASKLNQREACYNAGSFCGIHPGLCCSEFCILWCITFVDSG | 0 |
| MMSKLGVLLTVCLLLFPLTAVPMDGDQPADLPALRMQDFAPEHSPWFDPVRRCCSQDCRVCIPCCPH | 0 |
| MKLTCVMIVAVLFLTTWTFVTADDSRYGLKNLFPKARHEMKNPEASKLNKRDECYPPGTFCGIKPGLCCSAICLSFVCISFDF | 0 |
| MGVFRCCLAAALVVVCLSRMGGTEPLESNHEDERRADDTSGDDCVDTNEDCVNWASTGQCEANPSYMRENCRK | 1 |
| MKLMCMMIVAVLFLTAWTFVTADDSINGPENRRIWEKLLSKTRDEMKNPEASKLNKKECRQPGEFCFPVVAKCCGGTCLVICI | 0 |
| MPSVRSVTCCCLLWMMFSVQLVTPGSPGTAQLPRDRTARAPAELILETICPHMCGIGIGEPFCNCRNKRDVVSSRIIRRKRSMAV | 0 |
| MKLTCVMIVAVLFLTAWTFVTADDPRDGPDTAVGWRKLFSEARDEMKNREASKLNERGCIEDKKYCGILPFANSGVCCSYLCIFVCVPKAP | 0 |
| MMSKLGVLLTICMLLFPLAAFPLDGDQPADQPAERMQYDRLRAMNPWFDPIKRCRCEQTCGTCVPCC | 0 |
| MMSKLGVLLTICMLLFPLAAFPLDGDQPADQPAERMQYDMLRAMNPWFDPIKRCRCEQTCGTCVPCC | 0 |
| MKLTCMMIVAVLFLTAWTFVTADDSRNGLEYLFPKAHYEMNPEASKLNKKEDCEAGGRFCGFPKIGEPCCSGWCFFVCA | 0 |
| MKLTCMMIVAVLFLTAWTFVTADDSRNGLDYLFPKARHEMNPKASRDIKRCRPGGMICGFPKPGPYCCSGWCFVVCL | 0 |
| VVIVAVLLLTACQLITANDSRGAQKHRALRSDTKLSMSTRCKGKGASCRRTSYDCCTGSCRSGRCG | 1 |
| MPSVRSVTCCCLLWMMFSVQLVTPGSPGTAQLPRDRTARAPAELILETICPHMCGTGIGEPFCNCRNKRDVVSSRIIRRKRSMAV | 0 |
| MPSVRSVTCCCLLWMMFSVQLVTSDLTGTAWLSTHLDARIPSEENMEVLCPEMCNEGSGGVACSCSKRRDVVSSFVRRRKRSMV | 1 |
| VLIIAVLFLTACQLTTAETSSRGKQKHRALRSTDKKFKVALLCSPPGSYCFGPAACCSNFCSTLSDVCQESWSG | 1 |
| MKLTCMMIVAVLFLTAWTFVTADDSGNGLENHFSKSRHEMKNPKDSKLNKRCLDGGEICGILFPSCCSGWCIVLVCA | 0 |
| VVIVAVLLLTACQLITANDSRSTQKHRALRSDTKLSMSTRCKGKGASCRRTSYDCCTGSCRSGRCG | 1 |
| MKLTCVMIVAVLFLIAWTFVTADDSRNGLKNLFPKARHEMKNPEASKLNKRDGCSSGGTFCGIRPGLCCSEFCFLWCITFID | 0 |
| MMSKLGVVLTICLLPFPLTALPMDGDQPADLPALRTQDFEPERSPWFDPVKRCCSQDCRVCIPCCPY | 0 |
| MMSKLGVLLTICLLLFPLTAVPMDGDQPADLPALRTQDFEPERSPWFDPVRRCCSQDCSVCIPCCPPP | 0 |
| MMSKLGALLTICLLLFPLTALPLDGDQPADRPAERMQDDISSEQHPLFDKERQCCTGSCLNCWPCCG | 0 |
| VLIIAVLFLTACQLTTAVTSSRGEQKHRALRSTDKKFKVALLCSPPGSYCFGPAACCSNFCSTLSDVCQESWSG | 1 |
| MKLTCMMIVTVLFLTAWIFITADNSRNGIENLPRMRRHEMKNPKASKLNKRGCREGGEFCGTLYEERCCSGWCFFVCV | 1 |
| MKLTCVMIVAVLFLTAWTLVMADDSNNGLANLFSKLRDEMEDPEGSKLEKKDCQEKWDYCPVPFLGSRYCCDGFICPSFFCA | 0 |
| VLIIAVLFLTACQLTTAETSSRGKPKHRALRSTYKNSRMTKRCTPPEGGCLSSYECCSKICWRPRCYPS | 1 |
| FIVCQLIIADYSSDKQESRTVKLRNAIRNFKDSRSCGEEGEGCYTRPCCPGLKCIGTAHGGLCREE | 1 |
| FLTACQLITAETYSRGKQMHRALRSTDKNSKLTRECTPPGGACGLPTHCCGFCDMANNRCL | 1 |
| VVIVAVLLLTACQLITAEDSRGTQEHRALRSDTKLSMLTLRCESYGKPCGIYNDCCNACDPAKKTCT | 1 |
| MKLTCMMIVAVLFLTAWTFVTANDARNGLENLFPMARNEMKNHEASQLNERSCIEKGDFCGWVILSESCCTGFCFYVCI | 0 |
| MKLTCVMIVAVLFLTTWTFVTADDSRYGLKNLFPKARHEMKNPEASKLNKREGCSSGGTFCGIHPGLCCSEFCFLWCITFID | 0 |
| MKLTCMMIAAVLFLTTWTFVTADDSRYGLKNLFPKARHEMKNPEASKLNKREGCSSGGTFCGIHPGLCCSEFCFLWCITFID | 0 |
| MKLTCMMIVAVLFLTAWTFVTANDARNGLENLFPMARNEMKNHEASQLNKRCLDQGSFCGTLVFGVACCSGFCIIVCL | 0 |
| MEGRRLAVVLIVTSCLSALTVGDTVPGLSALTVDDDTVPDVCRQPLEVGPCKAAYPRYYYNHASDTCQLFYYGGCNGNENRFEDFSGCLFTCIYPWMAALGY | 1 |
| MVTLSSFKRLLKSLVRCCLQGGGMPSVRWLWAVTAVAVVVIVMALCGPYSGSRTHHQATVHQMTRNAGYHEGVATKLQTGGTSSGAVDRCHLPPETGMCRAYIPMHFYNATLGRCQGFIYGGCNGNDN | 0 |
| VVIVAVLLLTACQLITANDSRGTQKHRALRSDTKLSMSTRCQGRGASCRKTMYNCCSGSCNRGRCG | 1 |
| ADQCDDHPTVNGCTTPSFLDLQHEKTFTPACNRHDVCYGCGVKYGVTRTQCDEAFLRDMKEACRLERRRRKLTVNVN | 1 |
| VVIVAVLLLTACQLITANDSRGTQKHRALRSDTKLSMSARCQGRGASCRKTMYNCCSGSCNRGRCG | 1 |
| VVIVAVLLLTACQLITADDSRGTQEHRALRSDTKLSMLTLRCESYGKPCGIYNDCCNACDPAKKTCT | 1 |
| MMSKLGALLAICLLLFSLTAVPLDGDQHADQPAQRLQDRIPTEDHPLFDPNKRCCDDSECSYSCWPCCYG | 0 |
| MTSVQSVTCCCLLWLMLSVQPITPGSPGPAQLSRERSFRFIPGGIKEIACHRYCAKGIASAFCICPDKRDVVSPRIRRRKRSKAM | 0 |
| MMAKLGVLLTICLVLFPLTALPLDGDQLADRPAERMQDDNSPERSHWFDPVRRCCDLPCNAGCVPCC | 0 |
| MMSKLGVLLTICLLLFPITGLPLDEDQPADLPALRMEDFAPEHSPLFDPVKRCCILCWKCTYNCCRAW | 0 |
| MSKLGALFVICLLLFPLTAVPLDGDQPADQPAQRMQDDISSEHPLFDAVRGCCHLLACRMGCSPCCW | 0 |
| MMSKLGVLLTICLLLFPLTAVPLDGDQPADRPAERLQDDISSEHHPHFDSGRECCGSFACRFGCVPCCV | 0 |
| MKLTCMMIVAVLFLTAWTFVTADDSRYGLKNLFPKARHEMKNPEASKLNKRYGCSNAGAFCGIHPGLCCSELCLGWCT | 0 |
| VVIVAVLFLTACQLITADDSRRTQKHRALRSTTKRATSNRPCKPKGRKCFPHQKDCCNKTCTRSKCP | 1 |
| VLIIAVLFLTACQLTTAEASSRGKQKHRALRSTDKNSRMTKRCTPAGDACDATTNCCILFCNLATKKCEVPTFP | 1 |
| MMSKLGVLLTICLLLFPLTAVQLDGDQPADLPELRAQDFAPERSPWFDPVRRCCSQDCSVCIPCCPN | 0 |
| MMSKLGVLLTICLLLFSLTAVQLDGDQPVDLPALRTQDFAPEHSPWFNPVKRCCSRRCWVCIPCCPNGS | 0 |
| MSKLGALLTICLLLFSLTAVPLDGDQHADQPAQRLQDRIPTEDHPLFDPNKRCCDDSECSYSCWPCCYG | 0 |
| MKLTCMMIVAVLFLTAWTFVTADDSRNELKNLIRKRRDEMKNHEVSKLNKRDCLGAGQFCAAHSWIGVGCCNSFCIVICPF | 0 |
| MKLTCMMIVAVLFLTAWTFVTADDSKNGLENLFPMARNEMKNHEASQLNKRFCLDQGEFCGWVIFSEDCCSGFCLFACI | 0 |
| VVIVAVLLLTACQLITADDSRGTQKHRALRSDTKLSMSTRCQGRGASCRKTMYNCCSGSCNRGRCG | 1 |
| MSKLGALLIICLLLFPLTAVPMDGDQPADQPAERMQDDISFEQHPMFDATRSCCNAGFCRFGCTPCCY | 0 |
| MMSKLGALLTICLLLFPITGLPLDEDQPADLPALRMEDFAPEHSPLFDPVKRCCILCWKCTYNCCRAW | 0 |
| MKLTCMMIVAVLFLTAWTFVTADDSINGPDNRGIWGKPPSKARDEMKNPKASERNFCHPVGEPCFIGQIIPCCSGVCDVVCRGVFYSP | 0 |
| MKLTCMMIVAVLFLTAWTFVTADDSINGPDNRGIGGKPPSKGRDEMKNPKASVRNFCHPIGEPCFPKQFIPCCSGVCDVVCRGIF | 0 |
| MKLTCMMIVAVLFLTAWTFVTADDSINGPNNRQMWEKLLSKARDQMKNPEASKLDKRCIATDDFCGLPGIGWNCCTGVCIIVCVG | 0 |
| VMMIVAALLLTACQLSTAASHGRGKQEDPAGRWSGEMQDFEDLTLAKKCADGGDLCDPSSDNCCSECIDEGGSGVCAIVADVV | 0 |
| VLIIAVLFLTACQLTTAETSSRGEQKHRALRSTDKNSRMTKRCTPSGGACYYDYFCCSMTCNFNSKSCV | 1 |
| VLIIAVLFLTACQLTTAETSSRGEQKHRALRSTDKKFKVALLCSPPGSYCFGPAACCSNFCSTLSDVCQESWSG | 1 |
| VLIIAVLFLTACQLTTAVTSSRGEQKHRALRSTDKNSRLTKRCTPRNGYCYYRYFCCSRACNLTIKRCL | 1 |
| MKLTCVMIVAVLFLTAWTFATADDPRNGLGNLFSNAHHEMKNPEASKLNKRWCKQSGEMCNLLDQNCCEGYCIVLVCT | 0 |
| VLIIAVLFLTACQLTTAVTSSRGKQKHRALRSTDKKFAVTFICTPPGSDCNGHSDCCSNVCSTMSYVCQENWSG | 0 |
| MKLTCVMIIAVLFLTAWTFATADDSGNGLENLFSKAHHEMKNPKASKLNKRCTQSGELCDVIDPDCCNNFCIIFFCI | 0 |
| MKLTCLMIVAVLFLTAWTFVTAVPDSSNALENLYLKAHHEMNNPEDSELNKRCYDGGTSCNTGNQCCSGWCIFLCL | 0 |
| MKLTCVVIVAVLFLTAWTFATADDPRNGLGNLFSNVHHEMKNLEDSKLDKKCLGFGEACLMLYSDCCSYCVALVCL | 0 |
| VLIIAVLFLTACQLTTAETASRGKPKHRALRSTDKNSRMTRGCTHPGGACGGHHHCCSLFCNTAANACN | 1 |
| VLIIAVLFLTACQLTTAETSSRGKPKHRALRSTDKNSRMTMGCTHPGGACGGHYHCCSQSCNTAANSCN | 1 |
| VVIVAVLLLTACQLITANDSRGTQKHRALRSDTKLSMSTRCKSTGASCRRTSYDCCTGSCDRGRCG | 1 |
| VVIVAVLLLTACQLITANDSRGTQKHRALRSDTKLSMSTRCQGRGASCRKTSYDCCTGSCRSGRCG | 1 |
| MMSKLGVLLTICLLLFPLTAVPLDGDQPADRPAERLQDDISSEKQITNTPILDSGRECCGSFACRFGCVPCCV | 0 |
| VVIVAVLLLTACQLITANDSRGTQKHRALRSDTKLSMSTRCQGRGASCRKTMYNCCSGSCRSGRCG | 1 |
| VVIVAVLLLTACQLITANDSRGTQKHRALRSDTKLSMSTRCKSTGASCRRTPYDCCTGSCRSGRCG | 1 |
| VVIVAVLLLTACQLITANDSRGTQKHRALRSDTKLSMSTRCKGKGASCRRTSYGCCTGSCRSGRCG | 1 |
| GDCSCEGQICKCGYRVSPGKSGCACTCRNAK | 1 |
| MKLTCIMIVAVLFLTAWTFVTAVPHSSNALENLYLKAHHEMNNPEDSELNKRCYDGGTGCDSGNQCCSGWCIFVCL | 0 |
| MTSVQSVTCCCLLWLMLSVQPITPGSPGPAQLSRERSFRFIPGGIKEIACHRYCAKGIASAFCNCPDKRDVVSPRIRRRKRSKAM | 0 |
| MMSKLGALLTICLLLFSLTAVPLDGDQHADQPAERLQDDISSEHPMFDAVRRREQNQFKSFTSVKLLDSRGERCCGPTACLAGCKPCCG | 1 |
| MMSKLGVLLTICLLLFPLTAVPLDGDQPADRPAERMQDGISSEHHPFFDSVKKKQQCCPPVACNMGCEPCCG | 0 |
| MKLTCVVIVAVLFLTAWTLVMADDSNNGLANLFSKSRDEMEDPEAAKLEKNYCQEKWDYCPVPFLGSRYCCDGLFCTLFFCA | 0 |
| CIDGGEICDIFFPNCCSGWCIILVCVYCKLP | 0 |
| MKLTCMMIVAVLFLTAWTFVTAITSNGLENLFPKAHHEMKNPEASKLNKRCVPYEGPCNWLTQNCCDATCVVFWCL | 0 |
| MKLTCMVIVAVLFLTAWTFVTAITSNGLENLFPNAHHEMKNPEASKLNKRCVPYEGPCNWLTQNCCDATCVVFWCL | 0 |
| MKLTCVVIVAVLFLTAWTFVTAVPHSSNALENLYLKARHEMENPEASKLNTRYDCEPPGNFCGMIKIGPPCCSGWCFFACA | 0 |
| MKLTCMMIVAVLFLTAWTFVTAVPHSSNALENLYLKARHEMENPEASKLNTRYDCEPPGNFCGMIKIGPPCCSGWCFFACA | 0 |
| MKLTCVVIVAVLFLTAWTFVTAVPHSSNALENLYLKAHHEMNNPEASELNKRCYDGGTSCDSGIQCCSGWCIFVCF | 0 |
| MKLTCMMIVAVLFLTAWTFATADDSSNGLENLFSKAHHEMKNPEASKLNKRCIEQFDPCEMIRHTCCVGVCFLMACI | 0 |
| MKLTCMMIVAVLFLTAWTFVTADDSGNGLENLFSKAHHEMKNPEASNLNKRCAPFLHPCTFFFPNCCNSYCVQFICL | 0 |
| MKLTCVVIVAVLFLTAWTFVTAAPHSSNALENLYLKAHHEMNNPEDSELNKRCYDSGTSCNTGNQCCSGWCIFVCL | 0 |
| MMSKLGVLLTICLLLFPITGLPLDEDQHDDLPALRAQDFEPEHSPWFDPVRRCCSQDCSVCIPCCPN | 0 |
| VMIVAVLFLTACQLITADTSSRGKQKHRALRSTDKKRWIVGVCRLPGDLCAGDASCCEHSCNIVHTCD | 0 |
| VLIVAVLFLTACQLTTAETSSRGKQKHRALRSTDKNSKMTRCTDPGGACGNPGHCCSKFCITTSSTCN | 1 |
| VLIIAVLFLTACQLTTAETYPRGQQRHHALRSTDKNSKLTRGCTPRNGFCRYHSDCCSNFCHTWAIMCL | 1 |
| VLIIAVLLLTACQLTTAETSSRGKQKHRALRSTDKNSRMTKRCTPAGKACDATATCCVLFCNLVTNKCQVPRFP | 1 |
| VLIIAVLSLTACQLTTAETYPRGQQTHHALRSTDKNSKLTRGCTPRNGFCRYHSDCCSNFCHTWAIMCL | 1 |
| VLIVAVLFLTACQLTTAETYPRGQQRHHALRSTDKNSKLTRGCTPRNGFCRYHSHCCSNFCHTWAIMCL | 1 |
| VCCSFGSCDSLCQCCDC | 0 |
| DLCYQPMKVGPCRSKVPCYYFDHEYGKCQLFYYGGCRGNDNRFETKDACLHTC | 1 |
| ECREKGQGCTNAALCCPGLECEARVKVVCAWTIKWRLMFPLGALSCAA | 0 |
| AVCVTLVGKKPTVDVCSLPADPGPCEALDRRFFFDKVDGTCKPFNYGGCQGNGNRFDSKSRCERAC | 1 |
| MKLTCMMIVAVLFLTAWTFVTAVPHSSNALENLYLKAHHEMNNPEDSELNKRCYDSGTSCNTGNQCCSGWCIFVCL | 0 |
| MKLTCMMIVAVLFLTAWTFATADDSSNGLENLFLKAHHEMNPEASKLNERCLDAGEVCDIFFPTCCGYCILLFCA | 0 |
| MKLTCMMIVAVLFLTAWTFATADDSGNGLENLFSNAHHQMKNPEASKLNKRWCKQSGEMCNLLDQNCCDGYCIVLVCT | 0 |
| MKLTCMMIVAVLFLTAWTFATADDPRNGLGNLFSNAHHEMKNPEASKLNKRWCKQSGEMCNLLDQNCCDGYCIVLVCT | 0 |
| MKLTCVMIVAVLFLTAWTFATADDPRNGLGNLFSNAHHEMKNPEASKLNKRWCKQSGEMCNLLDQNCCDGYCIVLVCT | 0 |
| MKLTCLMIVAVLFLTAWTFATADDPRNGLGNLFSNAHHEMKNPEASKLNKRWCKQSGEMCNLLDQNCCDGYCIVLVCT | 0 |
| MPSVRSVACCCLLWMMLSVQLVTPGSPATAQLSGQRTARGPGSAICNMACRLEHGHLYPFCNCDGKRDVVSSSMAV | 0 |
| MMSKLGVLLTICLLLFALTAVPLDGDQPADRPAERMQDDISSERHPMFDAVRDCCPLPACPFGCNPCCG | 0 |
| MKLTCVMIVAVLFLTAWTFATADDPRNGLENLFSKTQHKMKNPEASKLNKRCKAENELCNIFIQNCCDGTCLLICIQNPQ | 0 |
| MKLTCVMIVAVLFLTAWTFATADDPRNGLDNRFSKARHEMNNRRASKLNKRCLEFGELCNFFFPTCCGYCVLLVCL | 0 |
| MKLTCLMIVAVLFLTAWTFVTAVPHSSNALENLYLKARHEMENPEASKLNTRDYDCEPPGNFCGMIKIGPPCCSGWCFFACA | 0 |
| MMSKLGVLLTICLLLFALTAFPLDGDQPADRPAERMQDDISSERHPMFDAVRDCCPLPACPFGCNPCCG | 0 |
| MMSKLGVLLTICLLLFPLTALPMDGDQPADQPAERLQDISPKETPGSDPFKRCCHWPYCAPPPLGCRCCGK | 0 |
| MKLTCMMIVAVLFLTAWTLVMADDSNNGLANHFSKSRDEMEDPEASKLEKRACRKKWEYCIVPIIGFIYCCPGLICGPFVCV | 0 |
| SKLGVLLTICLLLFPLTALPIDGDQPADQPAERMQDDISLEQNAFFDPVKRCCPVCISSCSGRCC | 0 |
| MKLTCMMIVAVLFLTAWTLVMADDSNNGLANHFLKSRDEMEDPEASKLEKRACSKKWEYCIVPILGFVYCCPGLICGPFVCV | 0 |
| MKLTCMMIVAVLFLTAWTLVMADDSNNGLANHFLESRDEMEDPEASKLEKRACSKKWEYCIVPILGFVYCCPGLICGPFVCV | 0 |
| MKLTCVVIVAVLFLTVWTFATADDSGNGLEKLFSNAHHEMKNPEASKLNERCLDAGEVCDIFFPTCCGYCILLFCA | 0 |
| MPSVRSVTCCCLLWMMFSVQLVTPGSPGTAQLSGHRTARFPRPRICNLACRAGIGHKYPFCHCRGKRDAVSSSMAV | 0 |
| MKLTTVLIVAVLVLAACQFTVTDNSGDDTENPSLRSAGENQNPDSTKTITARATRARTNMRRGLSRPSKGCIGGGDPCEFHRGYTCCSEHCIIWVCA | 1 |
| ETCDLSVEKRCSCSRHICCTHHALEQHKCMTWAKCMSVSLGVN | 1 |
| MTTLFYFLLMALGLLLYVCQSSFGNQHTRNSDTPKYRCGSDIPNSYMDLCFRKRNDAGKKRGQASPLWQRGGSLSMLKARAKRNEAFHLQRAHRGVVEHCCKRACSNAEFMQFCGNS | 1 |
| MTTSFYFLLMALGLLLYVCQSSFGNQHTRNSDTPKYRCGSDIPNSYMDLCFRKRNDAGKKRGQASPLWQRGGSLSMLKARAKRNEAFHLQRAHRGVVEHCCKRACSNAEFMQFCGNS | 1 |
| MTTSFYFLLVALGLLLYVCQSSFGNQHTRNSDTPWNRCGSQITDSYRELCPHKRNDAGKKRGQASPLWQRGGSLSMLKARAKRNEAFHLQRAHRGVVEHCCKRACSNAEFMQFCG | 1 |
| MTTSFYFLLMALGLLLYVCQSSFGNQHTRNSDTPKYRCGSDIPNSYMDLCFRKRNDAGKKRGQASPLWQRGGSLSMLKARAKRNEAFHLQRAHRGVVEHCCYRPCSNAEFKKFCG | 1 |
| MTTSFYFLLMALGLLLYVCQSSFGNQHTRNSDTPKYRCGSDIPNSYMDLCFRKRNDAGKKRGQASPLWQRGGSLSMLKARAKRNEAFHLQRAHRGVVEHCCYRPCSNAEFKKFCG | 1 |
| MTTSFYFLLMALGLLLYVCQSSFGNQHTRNSDTPKYRCGSEIPNSYIDLCFRKRNDAGKKRGRASPLWQRGGSLSMLKARAKRNEAFHLQRAHRGVVEHCCHRPCSNAEFKKFCG | 1 |
| MTTSFYFLLMALGLLLYVCQSSFGNQHTRNSDTPKYRCGSEIPNSYIDLCFRKRNDAGKKRGRASPLWQRGGSLSMLKARAKRNEAFHLQRAHRGVVEHCCHRPCSNAEFKKFCG | 1 |
| MKLTCMMIVAVLFLTAWTFATADDSGNGLENLFPKAHHEMKNPEASKLNKRCKQADEPCDVFSLDCCTGICLGVCMW | 0 |
| MKLTCMMIVAVLFLTAWTFATADDSGNGLEKLFSNAHHEMKNPEASKLNKRCKQADEPCDVFSLDCCTGICLGVCMW | 0 |
| VCCSFGSCDSLCQCCDGLAPNIHGAVLGGFIRTMSAFVDFTYHCATSLV | 0 |
| ECREKGQGCTNTALCCPGLECEGQSQGGLCVDNKWRLMFPLGALSCAA | 1 |
| ECREKGQGCTNTALCCPGLECEGQSQGGCAWTIKWRLMFPLGALSCAA | 1 |
| VLIIAVLFLTACQLTTAETYPRGQQRHHALRSTDKNSKLTRGCTPRNGFCRYHSHCCSNFCHTWAIMCL | 1 |
| MSKLGALLIICLLLFPLTAVPMDGDQPADRPAERMQDDISFEQHPMFDATRRCCNAGFCRFGCTPCCY | 0 |
| MMSKLGALLTICLLLFSLTTVPLDGDQHADQPAQRLQDRIPTEDHPLFDPNKRCCDDSECSYSCWPCCYG | 0 |
| MMSKLGALLTICLLLFSLTAVPLDGDQHADQPAQRLQDRIPTEDHPLFDPNKRCCDDSECSYSCWPCCYG | 0 |
| MKLTTVLVVALLVLAACQFTVTDNSGDDPENPSLRSAGENQNPDSTKTITAWATRDMTNMRRGLNRPSKRCLAGSARCEFHKPSSCCSGHCIFWWCA | 1 |
| MMSKLGVLLITCLLLFPLTAVPLDGDQPADQPAERLQDDISSENHPFFDPVKRCCRLLCLSCNPCCG | 0 |
| MKLTTVLVVALLVLAACQFTVTDNSGDDPENPSLRSVGENQNPDSTKTITAWATRDMTNMRRGLNRPSKRCLAGSARCEFHKPSSCCSGHCIFWWCA | 1 |
| MKLTCVMTVAVLFLTAWTFVTADDSRNGLKNLFPKARHEMKNPEASKLNKRDGCSNAGAFCGIHPGLCCSEICIVWCT | 0 |
| MKLTCVVIVAVLFLTAWTFVTADDTRYKLENPFLKARNELQKHEASQLNERGCLDPGYFCGTPFLGAYCCGGICLIVCIET | 0 |
| VLIIAVLFLTACQLTTAETSSRGKQKHRALRSTDKNSRMTTRCTPRNGVCFYSYFCCSRACNPSTKRCL | 1 |
| MEARRFAAVLILAICVLALGSGASRRQGAPAVCTMQKDPGPCKMAITRYYFNVAIFDCTTFSYGGCLGNGNNFENYDECYETCG | 0 |
| MSKLGALLTICLLLFSLTAVPLDGDQHADQPAQRLQDRIPTEDHPLFDPNKRCCPPVACNMGCKPCCG | 0 |
| MKLTCMMIVAVLFLTAWTFVTAVPHSSNALENLYLKARHEMENPEASKLNTRDDCEPPGNFCGMIKIGPPCCSGWCFFACA | 0 |
| MMFKLGVLLTICLLLFSLNAVPLDGDQPADQPAERLLDDISFENNPFYDPAKRCCRTCFGCTPCCG | 0 |
| VMIVAVLFLTACQLATADTSSRGKQKHRALRSTDKKFAVIFTCTPPGSHCTGHSDCCSDFCSTMSDVCQ | 0 |
| MEKLTILLLVAAVLLSTQVLVQCDGEKPKKTKLRFLKARMSSREEPCKERRQPCERNEECCNDSCNRFYC | 1 |
| MKLTTVLVVALLVLAACQFTVTDNSGDDPENPSLRSAGENQNPDSTKTITAWATRDMTNMRRGLNRPSKRCLAGSARCEFHKPSTCCSGHCIFWWCA | 1 |
| MMSKLGVLLTICLLLFPITADPLVEDQPADRPADRMQDIATEQHPLFDPVKRCCDRPCGIGCVPCCLP | 0 |
| MMFKLGVFLTICLLPFSLNAVPLDGDQPADQPAERLLDDISFENNPFYDPAKRCCRTCFGCTPCCG | 0 |
| MKLTCVMIVAVLFLTAWTLVMADDSNNGLANLFSKLRDEMEDPEGSKLEKKDCQEKWDYCPVPFLGSRYCCDGFICPSFFCA | 0 |
| MKLTCMMIIAVLFLTVWTFVANDDPRNGRQNRFSKARHGMKNRGAHRLDQRVCIKDGEFCGTPVENDPLCCSLICDLVCL | 1 |
| MKLTCMMIVAVLFLTAWTFATADDSGNGLENLFSNAHHQMKNPEASKLNKRWCKQSGEMCNLLDQNCCDGYCIVFVCT | 0 |
| MKLTCMMIVAVLFLTAWTFATADDSSNGLENLFPKAHHEMKNPEASKLNERCLDAGEICDFFFPTCCGYCILLFCA | 0 |
| MKLTCMMIVAVLFLTAWTFATADDSSNGLENLFLKAHHEMNPEASKLNERCLDAGEICDFFFPTCCGYCILLFCA | 0 |
| MKLTCMMIVAVLFLTAWTFATADDSSNGLENLFSKAHHEMKNPEASKLNKRCIEQFDPCDMIRHTCCVGVCFLMACI | 0 |
| MMFKLGVFLTICLLLFSLNAVPLDGDQPADQPAERLLDDISFENNPFYDPAKRCCRTCFGCTPCCG | 0 |
| MLKMGVVLCIVLVLFPLATLQLDADKPVERYAENKQLLNPDERRGIILHALGQRVCCPFGGCHELCLCCDG | 0 |
| MKLTCMMIVAVLFLTAWTFVTAAPHSSNALENLYLKAHHEMNNPEASELNKRCYDSGTSCNTGNQCCSGWCIFVSCL | 0 |
| MKLTCMMIVAVLFLTAWTFVTADDSRNGMENLFPKAGHEMENLEDSKHRHQERPDTGDKEEMLLQRQVKPCRKEHQLCDLIFQNCCRGWYCVVLSCT | 1 |
| VLIIAVLFLTACQLTTAETYPRGQQRHHALRSTDKNSKLTRGCTPRNGACGYHSHCCSNFCHTWANVCL | 1 |
| MKLTCMMIVAVLFLTAWTFATADDSSNGLGNLFLKAHHEMKNPEASKLNERCLDAGEVCDIFFPTCCGYCILLFCA | 0 |
| MMSKLGVLLTICLLLFPHTAVPLDGDQPADRYAERMQDDISSEHPMFDAVRGREQNQFKSFTSVKLLDSRGERCCGPTACLAGCKPCCG | 1 |
| MMSKLGALLTICLLLFSLTAVPLDGDQPADQPAERLQDDISSEHHPMFNSIRRREQNQFKSFTSVKLLDSRGERCCGPTACLAGCKPCCG | 0 |
| MMSKLGVLLTICLLLFPHTAVPLDGDQHADRPAERMQDDISSEHHPMFDAVKRREQNQFKSFTSVKLLDSRGERCCGPTACLAGCKPCCG | 1 |
| MMSKLGVLLTICLLLFPHTAVPLDGDQHADQPAERLQDDISFENNPMFNSIRRRVQNQFKSFTSVKLLDSRGERCCGPTACLAGCKPCCG | 1 |
| MMSKLGVLLTICLLLFPHTAVPLDGDQPADQPAERLQDDISSEHHPMFNSIRRREQNQFKSFTSVKLLDSRGERCCGPTACLAGCKPCCG | 0 |
| MMSKLGVLLTICLLLFPHTAVPLDGDQHADRPAERLQDDISSEHPMFDAVRRREQNQFKSFTSVKLLDSRGERCCGPTACLAGCKPCCG | 1 |
| MMSKLGVLLTICLLLFPHTAVPLDGDQPAERLQDDISSEHHPMFNSIRRREQNQFKSFTSVKLLDSRGERCCGPTACLAGCKPCCY | 0 |
| MMFKLGVLLTICLVLFPLTALPLDGEQPVDRHAEHMQDDNSAAQNPWVIAIRQCCTSCNFGCQPCC | 0 |
| VLIIAVLFLTACQLTTAETSSRGKQKHRALRSTYKNSRMTKRCMHPEGGCRFSYECCSKICYTPSFTCYPS | 1 |
| MKLTCMMIVAVLFLTAWTFATADDSGNGLEKLFSNAHHEMKNPEASNLNKRCAPFLHLCTFFFPNCCNGYCVQFICL | 0 |
| MSKLGALLIICLLLFPLTAVPLDGDQPADRPAERMQDDISSEHHPMFDAIRGCCHPSTCHVRKGCSRCCP | 0 |
| MMFKLGVLLTICLLLFSLNAVPLDGDQPADQPAERLPDDISSENNPFYDPVKWCCMTCFGCTPCCGSPASSSV | 0 |
| MMFKLGVLLTICLLLFSLNAVPLDGDQPADQPAERLPDDISSENNPFYDPVKRCCMTCFGCTPCCG | 0 |
| MMSKLGALLTICLLLFPLTAVPMDGDQPADQPAQRLQDRLPTEDHPLYDPVKRCCDDSECDYSCWPCCIFS | 0 |
| MSKLGVLLTICLLLFPLTALPLDGDQPADQAAERMQAEQHPLFDQKRRCCKFPCPDSCRYLCCG | 1 |
| MKLTCVLIITVLFLTACQLTTAVTYSRGEHKHRALMSTGTNYRLPKTCRSSGRYCRSPYDCRRRYCRRITDACV | 1 |
| MKLTYVLIVAMLVLVVCRADCFGRGGLCTWFDPSVCCSGICTFVDCW | 0 |
| VMIVAVLFLTACQLTTAETSSRGKQKHRALRSTDKNSRMTKRCTSPGGACYSASTCCSKACNLTTKRCVLS | 1 |
| MMSKLGVLLTICLLLFPHTAVPLDGDQHADQPAERLQDDISSEHHPMLNSIRRREQNQFMSFTSVKLRDSRGERCCGPTACMAGCRPCCG | 0 |
| MKLTCMMIVAVLFLTAWTFVTADDTRYRLENPFLKARNELQKHEASQLNERGCLDPGYFCGTPFLGAYCCGGICLIVCIET | 0 |
| MMSKLGALLIICLLLFPLTAVPLDGDQHADRPAERLQDDISSKHHPMFDAVRGCCHPSTCHMRKGCSRCCS | 0 |
| MMSKLGVLLTICLLLFPLTAVPLDGDQPADRPAERMQDGISSEHHPFFDSVKKKQQCCPPVACNMGCEPCCG | 0 |
| MMSKLGALLTICLLLFPLTAVPMDGDQPADRPAERMQDDISFEQHPMFDAVRGCCHLLACRMGCTPCCW | 0 |
| LGVLLPICLLLFPLTALPLDGDQPADRPAERMQDDFITEQHPLFDPVKRCCDWPCNAGCVPCCF | 0 |
| MKLTCALIITLLFLSITAGDSRGKHRYNALKSMSREANSTERECREKGQGCTNTALCCPGLECEGQGQGGLCVDN | 1 |
| MKLTCALIITLLFLSITAGDSRGKHRYNALKSMSREANSTERECREKGQGCTNTALCCPGLECEGRSQGGLCVDN | 1 |
| MKLTCMMIVAVLFLTAWTFVTAVPHSSDVLENLYLKALHETENHEASKLNKRCPNTGELCDVVEQNCCYTYCFIVVCL | 0 |
| MKLTCMMIVAVLFLTAWTFATADDPRNGLENLFSKAHHEMKNPEASKLNKRCPNTGELCDVVEQNCCYTYCFIVVCL | 0 |
| MMSKLGALLTICLLLFPLTAVQLDGDQPADLPELRAQDFAPERSPWFDPVRRCCSQDCSVCIPCCPN | 0 |
| MMSKLGVLLTICLLLFPLTAVQLDGDQPADLPALRAQDFAPERSPWFDPVRRCCSQDCSVCIPCCPN | 0 |
| MMSKLGVLLTICLLLFPLTAVQLDGDQPADLPELRAQDFAPERSPWFDPVRRCCSQDCSVCIPCCPN | 0 |
| MMSKLGVLLTICLLLFPLTAGQLDGDQPADLPALRAQDFAPERSPWFDPVRRCCSQDCSVCIPCCP | 0 |
| MMSKLGVLLTICLLLFPLTAVPLDGDQPADQPAERMQDDISSEQHPFFDPVKRCCVVCNAGCSGNCCS | 0 |
| MKLTCVVIVAVLFLTAWTFATADDPRNGLENLFSKAHHEMKNPEASKLNKRCPNTGELCDVVEQNCCYTYCFIVVCL | 0 |
| MMSKLGVLLTICLLLFPLTAVQLDGDQPADLPELRAQDFAPERSPWFDPVRRCCSQDCRVCIPCCPN | 0 |
| MMSKLGVLLTICLLLFPLTAVSLDGDQPADLPALRAQDFAPERSPWFDPVRRCCSQDCRVCIPCCPN | 0 |
| MMSKLGVLLTICLLLFPLTAVQLDGDQPADLPELRAQDFAPERSPWFDPVRRCCSQDCRVCIPCCPY | 0 |
| MSKLGALLIICLLLFPLTAVPLDGDQPADRPAERMQDDISSEHHPMFDAIRGCCHPSTCHVRKGCSRCCS | 0 |
| MMSKLGVLLTICLLLFPLTAVSLDGDQPADLPELRAQDFAPERSPWFDPVRRCCSQDCRVCIPCCPY | 0 |
| MMSKLGVLLTICLLLFPLTAVQLDGDQPADLPALRTQDISTDHSPWFDPVKRCCSRYCYICIPCCPN | 0 |
| MMSKLGVLLTICLLLFPLTVLPMDGDQPADLPALRTQDIATDQSPWFDPVKRCCSRYCWKCIPCCPY | 0 |
| MKLTCMMIVAVLFLTAWTFATADDPRNGLENLFSKAHHEMKNPKDSKLNKRCLDAGEMCDLFNSKCCSGWCIILVCA | 0 |
| MKLTCMMIVAVLFLTAWTFAAADDPRNGLENLFSKAHHEMKNPKDSKLNKRCLDAGEMCDLFNSKCCSGWCIILVCA | 0 |
| MKLTCMMIVAVLFLTAWTFATADDPRNGLENLFSKAHHEMKNPKDSKLNKRCIDGGEICDIFFPNCCSGWCIILVCA | 0 |
| GWCGDPGATCGKLRLYCCSGFCDCYTKTCKDKSSA | 1 |
| MKLTCVVIVVVLFLTAWTFVKADDSINGLENLFPKARHEMKNPEASKLNERCLEKGVLCDPSAGNCCSGECVLVCL | 0 |
| MKLTAVLMVAVLVLTACQLITANECSRKGEWCGLESVLCCNGGSWNCWFVCTA | 0 |
| MKLTCVVIVAVMFLTAWTFITADDSINGLEDRGIWGEPLSKARDEMNPEVSKRDCWPQYWFCGLQRGCCPGTTCFFLCF | 0 |
| MSKLGALLIICLLLFPLTAVPMDGDQPADQPAERMQDDISFEQHPMFDATRSCCNAGFCRFGCTPCCY | 0 |
| MMSKLGVLLTICLLLFPLTAVPLDGDQPADQPAERKQNEQHPLFDQKRGCCRWPCPSRCGMARCCSS | 1 |
| MMSKLGVLLTICLLLFPLTAVQLDGDQPADLPALRTQDIATDHSPWFDPVKRCCSRYCYICIPCCPN | 0 |
| MKLTCMMIVAVLFLTAWTSVTAVNTRGELENLFLRASHEMNSEASKLDKKVCVDGGTFCGFPKIGGPCCSGWCIFVCL | 0 |
| MTTSSYFLLMALGLLLYVCQSSFGNQHTRTFDTPKHRCGSEITNSYMDLCYRKRNDAGEKRGRASPLWQRRGSLSKLKARAKRNGAFHLPRDGRGVVEHCCHRPCSNAEFKKYCG | 1 |
| MTTSFYFLLMALGLLLYVCQSSFGNQHTRTFDTPKHRCGSEITNSYMDLCYRKRNDAGEKRGRASPLWQRRGFLSKLKARAKRNGAFHLPRDGRGVVEHCCHRPCSNAEFRKYCG | 1 |
| MSKLGVVLFLTACQLTTADLSSDEYHAVRSIDEMHDFKGSRATPECSRAGCKNVPCCSGLKCTGPQNGPVCQPE | 1 |
| MSKLGVVLFLTACQLLTADNSRDKQEYPTMRFRDQMRNAKGPKWIRSCAQSGLSCDTRPCCDDKPCVPNGRQSMCGG | 1 |
| MMSKLGVLLTICLLLFPHTAVPLDGDQPADQPAERLQDDVSSEHHPMFNSIRRREQNQFKSFTSVKLLDSRGERCCGPTACLAGCKPCCG | 1 |
| MMSKLGVLLTICMLLIPLTAVLLDRDQPADRHAERMKYMLRAMSPWFDPVKRCCKVLCESCTPCC | 0 |
| MKLTCVMIVAVLFLTAWTFVTAVPHSSDALENLYLKALHETENHEASKLNVRDDECEPPGDFCGFFKIGPPCCSGWCFLWCA | 0 |
| MMSKLGVLLIICLLLFSLNAVPLDGDQPADRYAERMQDDISSEHPLFDAVRGCCHLLACRMGCSPCCW | 0 |
| MKLTCMMIVAVLFLTAWTLVMADDSNNGLANHFLKSRDEMEDPEASKLEKRACRQKWEYCIVPILGFVYCCPGLICGPFVCV | 0 |
| MEGRRFAAVLILTICMLAPGAVVSRRRVQPSECKLPAAKGPCKGKYRKVYFNNFKKQCRMFTYGGCGGNGNKFRNAKECYHKCAYGVG | 1 |
| MKLTCMMIVAVLFLTAWTLVMADDSNNGLANHFSKSRDEMEDPEASKLEKRACRKKWEYCIVPIIGFIYCCPGLICGPFVLRLIGMSSTPICATPGLIFDRRVPFTGYELL | 0 |
| MKLTCMMIEAELFLTAWTFATADDPRNGLENLFSKAHHEMKNPEASKLNKRCPNTGELCDVVEQNCCYTYCFIVVCPI | 0 |
| MLKMGVMLFTFLVLFHLAAPQLDADQPVARYAGNKPDFNPNKRMKMMSALRKRGCCPPSLCDPGCDEGCCPVVTPAC | 0 |
| MTTSSYFLLMALGLLLYVCQSSFGNQHTRTFDTPKHRCGSEITNSYMDLCYRKRNDAGEKRGRASPLWQRRGSLSKLKARAKRNGAFHLPRDGRGVVEHCCHRPCSNAEFKKYCG | 1 |
| MKLTCMMIVAVLFLTAWTFVMADDPRDGPDTAVRGGKRFWKARNEMNSAASKLDKRECLEADYYCVLPFVGNGMCCSGICVFVCIAQAY | 0 |
| MKLTCMMIVAVLFLTAWTFATADDPRNGLENLFSKAHHEMKNPEASKLNKRCIDGGEMCDPFSSDCCSGWCIFFFCT | 0 |
| MNTAGRLLLLCLALGLVFESLGIPVADDVEADRDTDPDDGDPRGRNSWRSTDCNGVPCQFGCCVTINGNDECRELDC | 1 |
| MNTAGRLLLLCLALGLVFESLGIPVVDDVEADRDTDPDDGDPRGRNSWRSTDCNGVPCEFGCCVTINGNDECREIGCE | 1 |
| MNTAGRLLLLCLALGLVFESLGIPVADDVEADRDTDPDDGDPRGRNSWRSTDCNGVPCQFGCCVTINGNDECRDLIVSDLTRRGLLDNEGHCPAATES | 0 |
| MTTSFYFLLVALGLLLYVCQSSFGNQHTRNSDTPKHRCGSELADQYVQLCHGKRNDAGKKRGRASPLWQRQGFLSMLKAKRNEAFFLQRDGRGIVEVCCDNPCTVATLMTFCH | 1 |
| MTTSFYFLLVALGLLLYVCQSSFGNQHTRNSDTPKHRCGSELADQYVQLCHGKRNDAGKKRGRASPLWQRQGFLSMLKAKRNEAFFLQRDGRGIVEVCCDNPCTVATLRTFCH | 1 |
| MTTSFYFLLVALGLLLYVCQSSFGNQHTRNSDTPKHRCGSELADQYVQLCHGKRNDAGKKRGRASPLWQRQGFLSMLKAKRNEAFFLQRDGRGIVEVCCDNPCTVATLRTFCH | 1 |
| MNTAGRLLLLCLTLGLVFELLGIPVADDVEADRDADPDNGDPRGRISWRIEEDCGYVPCEFGCCRIIDGKEKCREIDCQ | 1 |
| MNTAGRLLLLCLALGLVFESLGIPVADDVEADRDTDRDDGNPRRDDFMRIMCGDEFCTYDCCEIVDGSSKCKQPDCP | 0 |
| MTTSFYFLLMALGLLLYVCQSSFGNQHTRTFDTPKHRCGSEITNSYMDLCYRKRNDAGKKRGRASPLWQRRGSLSQLKARAKRNGAFHLPRDGRGVVEHCCHRPCSNAEFKKYCS | 1 |
| MKLTCVVIVAVLFLTAWTFATADDPRNGLENLFSKAHHEMKNPEASKLNKRCPNTGELCDVVEQNCCYTYCFIVVCL | 0 |
| MKLTCMMIVAVLFLTAWTFVMADDPRDGPDTAVRGGKRFWKARNEMNSAASKLNKRECLEADYYCVLPFVGNGMCCSGICVFVCNTTKSN | 0 |
| MKLTCMMIVAVLFLTAWTFATADDPRNGLENLFSKAHHEMKNPKDSKLNKRCLDAGEMCDLFNSKCCSGWCIILFCA | 0 |
| MMSKLGVLLTICLLLFPLTAVPLDGDQHADRPADRMQDISSEQHPLFDPVKRCCDWPCTIGCVPCCLP | 0 |
| MMFKLGVLLTICLLLFSLNAVPLDGDQPADQPAERLPDDISSENNPFYDPVKRCCMTCFGCTPCCG | 0 |
| CTQDFDPCMPVCHECCTRSHFVVCRRPICL | 0 |
| MSKLGVLLTICLLLFPLTALPMDGDEPADRPAERMQDNISSEQHPLFEERHGCCKGPEGCSSRECRPQHCCGRR | 1 |
| MMSKLGVLLTICMLLFPLTALPLDGDQPADRPAERMQDDFISEQHPLFNPIKRCCDWPCTIGCVPCCK | 0 |
| VLIIALLFLTACQLTTAEASSRGKQKHRALRSTDKKFKVTLSCTPPGTYCVGPSTCCSDVCSMSNVCQ | 0 |
| VMIVAVLFLTACQLTTAETSSRGKQKHRALRSTDKNSRLTKRCTPPSGYCYHPYYCCSRACNLTRKRCL | 1 |
| VLIIAVLFLTACQLTTAETSSRGEQKHRAPRSTDKNSRMTKRCTHAYEACDATTNCCYMTCNLPTRKCRGPLFR | 1 |
| MEGRRVAAVLIVTICMLELEAATLRNPSLCSLLPDTGSCRAAFHMFYFDQFSKECKVFIYGGCDGNANRFLNSKACYKTCGNK | 0 |
| VMIVAVLFLTACQLTTAETSSRGKQKHRALRSTDKNSKMTRCTSPDGACNTPPQCCSKYCISISTTCN | 1 |
| VLIIALLFLTACQLTTAETSSRGKQKHRALRSTDKNSRMTMGCTHPGGACAGHHHCCSQSCNTAANSCN | 1 |
| MKLTCALIITLLFLSITAGDSRGKHRYNALKSMSREANSTERECRGKGQGCTNTALCCPGLECEGQSQGGLCVDN | 1 |
| MKLTCALIITLLFLSITAGDSRGKHRYNALKSMSREANSTERVCREKGQGCTNTALCCPGLECEGQSQGGLCVDN | 1 |
| MKLTCALIITLLFLSITAGDSRGKHRYNALKSMSREANSTERECKGKGQGCTNTALCCPGLECEGQSQGGLCVDN | 1 |
| VMIVAVLFLTACQLTTAETSSRGKQKYRVLRSADKNSKWTRECTPPGGACYYHSQCCGDFCQRYINSCL | 1 |
| VMIVAVLFLTACQLTTADTSSRGKQKYRVLRSTDKNSRLTRVCTPPEGYCTYHRDCCDLYCNKTTNVCLET | 1 |
| MKLTCALIITLLFLSITAGDSRGKHRYNALKSMSREANSTERECREKGQSCTNTALCCPGLECEGQSQGGLCVDN | 1 |
| MPSVRSVTCCCLLWMMLSVQLVTPGSPATAQLSGQRTARGPGSAICNMACRLGQGHMYPFCNCNGKRDVVSSSMAV | 0 |
| MPSVRSVTCCCLLWMMFSVQLVTPGSPATAQLSGQRTARGPGSAICNMACRLEHGHLYPFCHCRGKRD | 0 |
| MKLTCILAVGLLFLTAWTFVTADDSTGSENRGLWETLFPKARDEVENPEGDEVKKRDCHESGDFCGIPGVKNGLCCSQSCWVVCTPEW | 1 |
| LGVLLTICLLLFPLTAVPLDGDQPADQPAGRMQDDISSEQHPFFDPVKRCCVVCNAGCSGNCCP | 0 |
| MKLTCALIITLLFLSITAGDSRGKHRYNALKSMSREANSTERECREKGQGCTNTALCCPGLECEGQSQGGWCVDN | 1 |
| MKLTCALIITLLFLSITAGDSRGKHRYNALKSMSREANSTERECREKGQGCTDTALCCPGLECEGQSQGGLCVDN | 1 |
| MKLTCALIITLLFLSITAGDSRGKHRYNALKSMSREANSTERECREKGQGCANTALCCPGLECEGQSQGGLCVDN | 1 |
| MKLTCALIITLLFLSITAGDSRGKHRYNALKSMSREANSTERECREKGQGCTNTAFCCPGLECEGQSQGGLCVDN | 1 |
| MKLTCALIITLLFLSITAGDSRGKHRYNALKSMSREANSTERECRKKGQGCTNTALCCPGLECEGQSQGGLCVDN | 1 |
| VMIVAVLFLTACQLTTADTSSRGKQKYRVLRSTDKNSKWIRECTPPEGACNHPSHCCEDFCDRGRNRCM | 1 |
| MMSKLGVLLTICLLLFPLTAVRLDGDQHTDRPADRMQDIATEQHPLFDPVKRCCDWPCTIGCVPCCLP | 0 |
| MKLTCALIITLLFLSITAGDSRGKHRYNALKSMSREANSTERECREKGQGCTNTALCCPAVECEGQSQGGLCVDN | 1 |
| MKLTCALIITLLFLSITAGDSRGKHRYNALKSMSREANSTERECREKVQGCTNTALCCPGLECEGQSQGGLCVDN | 1 |
| MMSKLGVLVTICLLLFPLTAFPLDGDQPADHPAKRTQDRNSASLISSWVDNTNFCCCSHDCATICDDCF | 0 |
| MMSKLGVLLTVCLLLFPLTALRLVRDQPAERPAKRTQDDIPNGQDPLIDRQINCCPWPCPDSCHYQCCH | 1 |
| MMSKLGVLLTICLLLFPLTAVPLDGDQPADRPAERMQDDISSERHPFFDRSKQCCHLAACRFGCTPCCW | 0 |
| MMSKLGVLLTICLLLFPLTAVPLDGDQPADRPAERMQDDISSERHLFFDRSKQCCHLAACRFGCTPCCW | 0 |
| MKLSVVIIAALFLTACQLVTARNHARYLWESPRRKMQRTWEGKEVQPCKGPGSWCGGEEVPTECCEVCVFGWCT | 0 |
| MMSKLGILLTICLLLFPLTAVPLDGDQPADRPAERMQDDISSEHHPFFDPVKRCCRLSCGLGCHPCCG | 0 |
| MMSKLGILLTICLLLFPLTAVPLDGDQPADRPAERMQVDISSEHHPFFDPVKRCCRLSCGLGCHPCCG | 0 |
| MKVTCMMIVAVLFLTAWTFVTADDSISALEDLFAKAHDKMENSEASPLNERDCRALGEYCGLPYVHNSRCCSQLCGFICVPESP | 0 |
| MMSKLGVLVTICLLLFPLTAFGDQPADHPAKRTQDRNSASLISSWVDNTNFCCCSHDCATICDDCF | 0 |
| MNRMGFFLMLTAAVLLTSLVCTEAAPADEAKMERAQQSNRDRSRNPEKRCVDCRPGYKCCGVCTMNQCTGREIPKE | 1 |
| MKLTCALIITLLFLSITAGDSRGKHRYNALKSMSREANSTERECREKGQGCTNTALCCPGLECEGRGSKELC | 1 |
| CQLITALDSKGMEKRRALRSTTKLSKLRQVSWWCGKPEATCGKLYLKCCSGMCNKANWKCLR | 1 |
| MKLFMTFLLLLMILPLCQSSGLRQLLATNRFGSKDKPRSAVSKRCSGNPCSTERKCCKGYFCGEGKCLSRQRGTFRNGK | 1 |
| MKLTCALIITLLFLSITAGDSRGKHRYNALKSMSREANSTERECREKGQGCTNTALCCPGLECEGQSQGGCAWTISGV | 1 |
| MNRMGFFLMLTAAVLLTSLVCTEATPADESKVKRARWSRIEGSRLFRHRLPKSSQSTCPYCQISCCPPAYCQPSGCRGP | 1 |
| MKLTCALIITLLFLSITAGDSRGKHRYNALKSMSREANSTERECREKGQGCTNTALCCPGLECEGQSQGGCAWTLSGV | 1 |
| MMSKLGILLTICLLLFPLTAVPLDGDQPADRPAERMQDDISSEHHPFFDPFKRCCRLSCGLGCHPCCG | 0 |
| MMSKLGILLTICLLLFPLTAVPLDGDQPADRPAERMQDDISSEHHPLFDPVKRCCRLSCGLGCHPCCG | 0 |
| MKLMCVLIIAVLFLTASQLITADYSRDKQEYRAERLRDAMGKFKGSRSCGHSGAGCYSRPCCPGLHCSGTHAGGMCV | 0 |
| MMSKLGVLLTICLLLSPLTALPLDGDQPAERLQDDNSAAQNHWFDHVKRCCPWPCNNGCEPCCG | 1 |
| MKLTCALIITLLFLSITAGDSRGKHRYNALKSMSREANSTERECREKGQGCNTALCCPGLECEGQSQGGLCVDN | 1 |
| MKLMCVLIIAVLFLTASQLITADYSRDKQEYRAERLRDAMGKFKGSRSCGHSGAGCYTRPCCPGLHCSGGQAGGLCV | 0 |
| MKLTCVMIVALLFLTAWAFVTADDLINKLENRGGWAQARGWGKPFAMARDEASKLDNEKRCYDVGDFCGIPFIKNGNCCSQFCVFVCTPEW | 0 |
| MMSKLGILLTICLLLFPLTAVPLDGDQPADRPAERMQDDISSEHHSLFDPVKRCCRLSCGLGCHPCCG | 0 |
| MKLTCALIITLLFLSITAGDSRGKHRYNALKSMSREANSTERECREKGQGCTNTALCCPGLECEGQGSKELC | 1 |
| MMSKLGVLLTICLLLFPLTALPMDGDQPADQPAERLQDISPKEIPGSDPFKRCCHAPYCTPPHLGCPCCGK | 0 |
| MKLTCMMIVAVLFLTAWTLVMADDSNNGLANLFSKSRDEMEDPEASKLEKKDCQEKWDFCPAPFFGSRYCCFGLFCTLFFCA | 0 |
| MMSKLGVLLTICLLLFPLTALPMDGDQPQERKEDGKSAALQPWFDPVKRCCQAACSPWLCLPCCR | 0 |
| MPSVRSVTCCCLLWLMLSLTLVTPGSPGPAQLPGHRAARVPAEPILEEICPDMCNSGEGEIFCTCGSRQFVVTLPVIERKRSMAV | 0 |
| MKLTCVVIVAVLFLTAWTFVMADDSRYGLKDLFPKARHEMKNPEASKLNKRDECFSPGTFCGIKPGLCCSAWCYSFFCLTLTF | 0 |
| MSKLGVLLTICLLLFPLTALPMDGDQPVDRLAERMQDNISSEQHTFFEKRLPSCCSLNLRLCPVPACKRNPCCTG | 0 |
| MKLTCVVIVAVLFLTAWTFVTADDSRYGLKNLFPKARHEMKNPEASKLNKRDECYPPGTFCGIKPGLCCSERCFPFVCLSLEF | 0 |
| MKLTCVMIVAVLFLTAWTFVTADDSRNGLENLSPKARHEMKNPEASKSNKRYECYSTGTFCGVNGGLCCSNLCLFFVCLFS | 0 |
| MKLTCVMLVAVLFLTAWTFATADDSSNGLENLFSKAHHEMKNPEASKLNKRCIPQFDPCDMVRHTCCKGLCVLIACSKTA | 0 |
| ACQLITAEDSRGTQLHRALRSTSKVSKSTSCVEAGSYCRPNVKLCCGFCSPYSKICMNFPKN | 1 |
| MKLTCMMIVAVLFLTAWTFVTAVPHSSDVLENLYLKALHETENHEASKLNVRDDECEPPGDFCGFFKIGPPCCSGWCFLLGCA | 0 |
| MPSVRSVTCCCLLWLMLSLTLVTPGSPGPAQLPGHRAARVPAEQMMEELCPDMCNRGEGEIICTCVLRRHVVSPSIRGRKKIDGGVNAYSHF | 0 |
| MKLTCMMIVAVLFLTAWTFVTAVPHSSDVLENLYLKALHETENHEASKLNVRDDECEPPGDFCGFFKIGPPCCSGWCFLWGCA | 0 |
| MKLTCMMIVAVLFLTAWTFVTAVPHSSDVLENLYLKALHETENHEASKLNVRDDECEPPGDFCGTFLKIGPPCCSGWCFLWCA | 0 |
| MKLTCLMIVAVLFLTAWTFATADDPRNGLENFFSKTQHEMKNPEASKLNKRCKAESEACNIITQNCCDGKCLFFCIQIPE | 0 |
| MMSKLGVLLTICLLLFPITGLPLDEDQPADLPALRTQDFEPEHSPWFDPVRRCCSQNCLTCIPCCPY | 0 |
| MMSKLGALLTICLLLFPLTAVPLDGDQPLDRHAERMHDGISPKRHPWFDPVKRCCKVQCESCTPCC | 0 |
| MSKLGALLIICLLLFSLTAVPLDGDQHADQPAQRLQDRIPTEDHPLFDPNKRCCPPVACNMGCKPCCG | 0 |
| AACQLGTAASFARDKQDYPAVRSDGRQDSKDSTLDRIAKRCSEGGDFCSKNSECCDKKCQDEGEGRGVCLIVPQNVILLH | 1 |
| VLIIAVLFLTACQLTTAETSSRGKQKHRALRSTDKNSRMTKRCTPAGGACDATTECCILFCNLATKKCQVPTFP | 1 |
| MMSKLGALLTICLLLFSLTAVPLDGDQHADQPAQRLQDRIPTEDHPLFDPNKRCCPPVACNMGCKPCCG | 0 |
| MKLTCMMIVAVLFLTAWTFVTAVPHSSDVLENLYLKALHETENHEASKLNVRDDECEPPGDFCGFFKNWVRLCCSGWCFLWCA | 0 |
| MPSVRSVTCCCLLWLMLSLTLVTPGSPGPAQLPGHRAARVPAEPILEQLCPEMCNRGELEFFCTCGSRQFVVTLPVIERKRSMAV | 0 |
| MRSVQSVTCCCLLWLMLSLTLVTPGSSGPAQRPGHRAARVPAEPILEQLCPEMCNRGELEFFCTCGSRQFVVTLPVIERKRSMAV | 0 |
| MKLTCVLIIAVLFLMDNQLITADYPRDEQVYRAVRLRDAMQKSKGSGSCAYISEPCDILPCCPGLKCNEDFVPICL | 0 |
| MKLTCVVIVAVLFLTALADDSRNGLENRNEQERNENEMRDRRDCQDSGVVCGFPKPEPHCCSGWCLFVCA | 1 |
| MKLTCMMIVAVLFLTAWTFVTAVPHSSDVLENLYLKALHETENHEASKLNVRDDECEPPGDFCGFLKLGPPCCSGWCFLWCA | 0 |
| MKLTCMMIVAVLFLTAWTFVTAVPHSSDVLENLYLKALHETENHEASKLNVRDDECEPPGDFCGLFKIGPPCCSGWCFLWCA | 0 |
| MMSKLGVLLTICLLLFPLTALPMDGDQPAYRPAERRQHVSSEQHPLFDAVKGCCTHPCTLGCTPCCY | 0 |
| MKLTCMMIVAVLFLTAWTFATADDPRNGLENFFSKTQHEMKNPEASKLNKRCLAEHETCNIFTQNCCEGVCIFICVQAPE | 0 |
| MMSKLGALLTICLLLFSLTAVPLDGDQHADQPAERLHDRLPTENHPLYDPVKRCCDDSECDYNCWPCCIFG | 0 |
| MMSKLGVLLTICLLLFPLTALPMDGDQPVDRPAERMQGNISSEQHPMFDPIKRCCTQSCTTCFPCC | 0 |
| MKLTCLMIVAVLSLTGWTFVTADDSGNGLGNLFSNAHHEMKNPEASKLNKRCVPHEGPCNWLTQNCCSGYNCIIFFCL | 0 |
| MKLTCMVIITVLFLTASQLITADYSRDQRQYRAVRLGDEMRNFKGARDCGGQGEGCYTQPCCPGLRCRGGGTGGGSCQP | 1 |
| MKLTCMMIVAVLFLTAWTFVTAVPHSSDVLENLYLKALHETENHEASKLSVRDDECEPPGVFCGTFKIGPPCCSGWCFLWCA | 0 |
| MKLTCMMIVAVLFLTAWTFVTADDSKNALENRGGWGKPFPEARDEMKNPKASELRECSSYDEYCFPGIRDCCSGFCFIICI | 0 |
| MKLTCVLIIAVLFLTAYQLTTVETYSRGKWMHRALRSTGKNPKVTRECSSPDESCTYHYNCCQLYCNKEENVCLENSPEV | 1 |
| DSDSGLAPGTCILFGSMCKENVSICCYRCDIEENCCKS | 0 |
| MKFFLSSRLRSCLFSRTAYQLTTVETYSRGKWMHRALRSTGKNPKVTRECSSPDESCTYHYNCCQLYCNKEENVCLENSPEV | 1 |
| VLIIAVLFLTACQLTTAETYSRGRQKHRARRSTDKNSKWTRECTRSGGACNSHDQCCNAFCDTATRTCV | 1 |
| MLKMGVVLFIVLVLFPLATLQLDADKPVERYAENKQLLNPDERRGIILHALGQRVCCSFGSCDSLCQCCDC | 0 |
| VLIIAVLFLAACQLTTAETSSRGKQKHRALRSTDKNSRMTKRCTPAGDACDATTECCILFCNLATKECQVPAFP | 1 |
| MKLTCLMIVAVLFLTTWTFVTADDSRYGLKNLFPKARHEMKNPEASKLNKRDGCYNAGTFCGIRPGLCCSEFCFLWCITFVDSG | 0 |
| MKLTCMMIVAVLFLTAWTLVMADDSNNGLANHFLKSRDEMEDPEASKLEKRACRKKWEYCIVPILGFVYCCPGLICGPFVCV | 0 |
| MVSLGHVLFVILLPVLLPVAADDPDDQMLSQISLPSSSRSEYDDNDVSKRVFCNGFTGCGGRHRDRSRRQERYGKRLIPVLAKRPFCNSFGCYNGKRSLSGAGPALSTPVDPSRNNKARTMARMLDAAASARHEQQQQLLQQREQRGLESRDPAASGDLSKRLFCNGYGGCRGGKRTLYSPWLERMNEVADDRSARNALCTRLGWRE | 1 |
| MMSKLGVLLIICLLLFPLTAIPQDGDQPADRPAERMQDDISFEHHPMFDATRGCCNAGFCRFGCTPCCW | 0 |
| MSKLGALLIICLLLFPHTAVPLDGDQPADQPAERLQDDISSEHHPMFNSIRRREQSQFKSFTSVKLLDSRGERCCGPTACLAGCKPCCG | 1 |
| MKLTCMVIVAVLFLTANTFVTADDSGNGLENLFSKAHHEIKNPEASNLNKRCIPFLHPCTFFFPDCCNSICAQFICL | 0 |
| VLILTICMLAVAAGASRRWGLCSLPAEAGPCYASITRYYYDRKTQECTQFYYGGCGGNSNNFDTAEECDDVC | 1 |
| MMYKLGVLLIICLLLFPLTAVPQDGDQPADRPAERMQDDISFEHDRFFDPVKRCCKYGWTCWLGCSPCCG | 0 |
| VLIIAVLFLTACQLTTAETYSRGRQKHRARRSTDRNSKWTRECTDSGGACNSHDQCCNEFCSTATRTCI | 1 |
| MKLTCMMIVAVLFLTAWTFVTAVPHSSNVLENLYLKARHEMENQEASKLNMRDDDCEPPGNFCGFPKIGGPCCSGWCFFACA | 0 |
| VLIIAVLFLTACQLTTAETYSRGRQKHRARRSTDKNSKWTRECTDSGGACNSHDQCCNEFCSTATRTCI | 1 |
| CQLITADDSREKQGYSAVRSSDKIQDSDDLKLTKRCTENGDVCDPENHNCCSGSCLDDEDPPVCGF | 1 |
| MKLTCMMIVAVLFLTAWTFVTADDPRNGLENLFSKAHHEMKNPEASKLNKRCIDGGEMCDPFSSDCCSGWCIFFFCI | 0 |
| VLLLVTLCVQAEDKCQQPKAPGRCMAYMERYFFNSEKGACEQFIYGGCEGNENNFETLEACQTAC | 1 |
| VLILAICMLALGSGATRRRAVRSVCTLQKDTGPCKMAIPRYYFNMDISDCDTFIYGGCFGNANNFETYEECDDTC | 0 |
| MMSKLGALLTICLLLFSLTAVPLDGDQHADQPAQRLQDRIPTEDHPLFDPNKRCCDDSECSYSCWPCCYG | 0 |
| MMSKLGVLLTICLLLFPLTALPMDGDQPVDRPAERMQGKISSEQHPMFDPIEGCCTQSCTTCFPCCLI | 0 |
| MMSKLGVLLTICLLLFALTAVPLDGDQPADRPAERMQDDISSERHPMFDAVRDCCPLPACPFGCNPCCGWPALLSGPHQVMNNE | 0 |
| MMSKLGVLLTICLLLFPLTALPMDGDQPQERKEDGKSAALQPWFDPVKRCCQAACSPWPCLPCCR | 0 |
| MKLTCLMIIAVLFLTAWTFVMADDPRDEPEARDEMNPAASKLNERGCLEVDYFCGIPFVNNGLCCSGNCVFVCTPQGK | 0 |
| MTSVQSVTCCCLLWLMLSLTLVTPGSPGPAQLPGHRAARVPAEPILEEICPDMCNSGEGEIFCTCGSRQFVVTLPVIERKRSMAV | 0 |
| MMSKLGVLLTICLLLFPITADPLVEDQPADRPADRMQDIATEQHPLFDPVKRCCDRPCSIGCVPCCLP | 0 |
| MNCYLILTVALLLTSAMTGTTTAGQLNTKGVTLREDDRTFPCSSGLCACLPLDSYSYICLSPSSSTANCENDECISEDDW | 0 |
| MKVTCVLTLAVLILTVGQMVTADCRSPGSWCFYKHSNCCSGNCFLWCVQNGK | 0 |
| MKLTCALIITLLFLSITAGDSRGKHRYNALKSMSREANSTERECREKGQGCTNTALCCPGLECEGQSQGGLCVDN | 1 |
| MNCYLILTVALLLTSAMTGTTTAGQLNKKGVTLREDDRFPCNAGNCACLPLDSYSYTCQSPTSSTANCEGNECRSEADW | 1 |
| MMSKLGVLLTICLLLFPLTAVPLDGDQPADRPAERMQDDISSENHPMFDAIRGCCMPLSCMLLCEPCCG | 0 |
| MMSKLGVLLIICLLLFPLTAVPLDGDQPADRPAERMQVDIPTEHHPMFGAVRGCCGAWACMAGCRPCCG | 0 |
| MSKLGVLLTICLLLFPHTAVPLDGDQPAERLQDDISSEHHPMFNSIRRREQNQFKSFTSVKLLDSRGERCCGPTACLAGCKPCCG | 1 |
| MKLTCMMIVAVLFLTAWTFVTAVPHSSDVLENLYLKALHETENHEASKLNVRDDECEPPGVFCGTFKIGPPCCSGWCFLWCDLKLP | 0 |
| MKLTCMMIVAVLFLTAWTFVMADDPRDGPDTAVRGGKRFWKARNEMNSAASKLNKRECLEADYYCVLPFVGNGMCCSGICVFVCMAQKY | 0 |
| VLIIAVLLLTACQLTTAETYSRGRQKHRARRSTDKNSKWTRECTHSGGACNSHDQCCNAFCDTATRTCV | 1 |
| MKLTCVLIIAVLFLTAITADDSRDKQVYRAVGLIDKMRRIRASEGCRKKGDRCGTHLCCPGLRCGSGRAGGACRPPYN | 1 |
| ACRKKWEYCIVPIIGFIYCCPGLICGPFVCVLIGMSSTPICATPGLIFD | 0 |
| MKLTCVMIVAVLFLTAWTVVTAVPHSNKRLANLYLKARHEMKNPEASNVDKRCFESWVACESPKRCCSHVCLFVCT | 0 |
| MKLTCMMIVAVLFLTANTFVTADDSGNGMENLFPKAGHEMENLEASNRGKPCHEEGQLCDPFLQNCCLGWNCVFVCI | 0 |
| VLIIAVLFLTACQLTTAETYSRGRQKHRARRSTDKNSKWTRECTHSGGACNSHDQCCNAFCDTATRTCV | 1 |
| VLIIAVLFLTACQLTTAETYSRGRQKHRARRSTDKNSKWTRECTRSGGACNSHTQCCDDFCDTATRTCV | 1 |
| VLIIAVLFLTACQLTTAETYSRGRQKHRARRSTDKNSKWTRECTRSGGACNSHTQCCNAFCDTATRTCV | 1 |
| MKLTCVVIVAVLFLTANTFATADDPRNGLENLFLKAHHEMNPEASKLNERCLSGGEVCDFLFPKCCNYCILLFCS | 0 |
| VLIIAVLFLTACQLTTAETYSRGRQKHRARRSTDKNSKWTRECTRSGGACNSHTQCCDDFCSTATSTCT | 1 |
| VLIIAVLFLTACQLTTAETYSRGKQKHRALRSTDKNSKMTKKCTQTNGACYHRDTCCSKSCNLTINRCLAS | 1 |
| MKLTCMMIVAVLFLTAWTFATADDPRNGLENLFLKAHHEMNPEASKLNERCLSGGEVCDFLFPKCCNYCILLFCS | 0 |
| VLIIAVLFLTACQLTTAETSSRGEQKHRAPRSTDKNSRMTKRCTPGGEACDATTNCCFLTCNLATNKCRSPNFP | 1 |
| MKLTCMMIVAVLFLTAWTFVMADDPRDGPDTAVRGGKRFWKARNEMNSAASKLNKRECLEADYYCVLPFVGNGMCCSGICVFCLHSPKVLKLS | 0 |
| MMSKLRVLLIICLLLFPLTAVPLDGDQPADRPAERTQDDISSEHHPMFDAVRGCCPALACAMGCRPCCG | 0 |
| MKLTCMMIVAVLFLTAWTFVTAVPHSSDVLENLYLKALHETENHEASKLNVRDDECEPPGDFCGFFKIGPPCCSGWCFLWCDLKLP | 0 |
| MMSKLGVLLTICLLLFPHTAVPLDGDQHADRPAERLQDDISSEHHPMFDAVKRREQNQFKSFTSVKLLDSRGERCCGPTACLAGCKPCCG | 1 |
| MKLTCMMIVAVLFLTAWTFVTAVPHSSDVLENLYLKALHETENHEASKLNVRDDECEPPGDFCGFFKIGPPCCSGWCFLWCV | 0 |
| MKLTCMMIVAVLFLTAWTFVTAVPHSSDVLENLYLKALHETENHEASKLNVRDDECEPPGDFCGFFKIGPPCCSGWCFLWCS | 0 |
| MMSKLGALLIICLLLFSLTAVPLDGDQPADQPAERMQDNISSKHHPMFDAVRGCCHPSTCHMRKGCSRCCS | 0 |
| MMSKLGVLLTICLLLFPITGLPLDEDQHADLPALRAQAFEPEHSPWFDPVRRCCSQDCSVCIPCCPN | 0 |
| VLIIAVLFLTACQLATAETSSRGKQKHRALRSTDKTSRMTNRCTAPGGACYAAYTCCSNACNLNTKKCVLS | 1 |
| VLIIAVLFLTACQLTTAETSSRGKQKHRALRSTDKNSRMTKRCTAPGGACYAAYTCCSNACNLNTKKCVLS | 1 |
| MKLTCMMIVAVLFLTAWTFATADDPRNRLENFFSKTQHEMKNPEASKLNKRCIAESEPCNIITQNCCDGKCLFFCIQIPE | 0 |
| VLIIAVLFLTACQHTTAETSSRGKQKHRALRSTDKNSRMTKRCTAPGGACYAAYTCCSNACNLNTKKCVLS | 1 |
| VVIVALLFLTACQLITADNSKGTQKHRALRSTTKLSMLTRGCTPPGGACGYHGHCCDFCDTFGNLC | 0 |
| MNLTCMMIVAVLFLTAWTFVMADDSNNGLANLFSKSRYEMEDPEPSKLEKRKTCQRRWDFCPGALVGVITCCGGLICLGVMCI | 0 |
| LTCMMIVAVLFLTAWTFVTADDTRDGLKNLFPKARLEMKNSEASRSRGRCRPPGMVCGFPKPGPYCCSGWCFAVCLPV | 0 |
| VLIIAVLFLTACLLTTAVTSSRGXQKHRALRSTDKNSRMTKRCTAPGGACYAAYTCCSNACNLNTKKCVLS | 0 |
| VLIIAVLFLTACQLTTAETSSRGKQKHRALRSTDKNSRMTKRCTAPGGACYADNTCCSNACNLNTKKCVLS | 1 |
| VLIIAVLFLTACQLTTAETSSRGEQKHLALRSTDKNSRMSKRCTPPGGYCYHPDPCCSQVCNFPRKHCL | 1 |
| MKVTAVLMVAVLVLTACQLTTANTTDYVRRIPARKSTMSRRECSESGEWCGLDPALCCGSSCFFTCN | 0 |
| MKVTAVLMVAVLVLTACQLTTANTTDYVRRILARKSTMSKRYCSDSGGWCGLDPELCCNSSCFVLCG | 0 |
| MTSVQSVTCCCFLWLMLSVQLITPSPPGTAHLPRDGTDRNFPDILGDILCPFFCLSELGLDLGGEFCDCSEIPDLGLLPDQ | 0 |
| MSKLGVLLTICLLLFPITALPVDGDQPADRPVERMQDNISSEQHPFFEKRAARCCTYHGSCLKEKCRRKYCCGR | 1 |
| MTSVQSVTCCCFLWLMLSVQLISPSSPGTAHLPRDGTDRAFQDIFMVALCPVFCFSNVALESCNCEEILDLVSSLISRKRSMAM | 0 |
| MASVQSVTCCCLLWLMLSVQLVNPGSPGTAQLSGHRTARVPPEPILEIICPGMCDEGVGKEPFCHCTKKRDAVSSRIRRRKRSAAV | 0 |
| VIIIAVLFLTACQLIATASYARSERKHPDLRLSSRNSKLSKRCLGSRELCVRDTSCCSMSCTNNICF | 1 |
| MTSVQSVTCCCFLWLMLSVQLITPSPPGTAHLPRDGTDRYIPDDDESGVCYFLCLMGIDLDECSCPEIQDLGLFTDQ | 0 |
| MMSKLGALLTICLLLFSLTAVPLDGDQHADQPAERLQDDISSENNPMFNSIRRREQNQFKSFTSVKLLDSRGERCCGPTACLAGCKPCCG | 0 |
| MKLTCMMIVAVLFLTAWTVVTAVPHSNKRLANLYLKARHEMKNPEASNVDKRCFESWVACESPKRCCSHVCLFVCA | 0 |
| VLIIAVLFLTACQLTTAETSSRGKQKHRALRSTDKDSRMTKRCTPAGDACDATTKCCIPFCNLATKKCQVPTFP | 0 |
| CQLNTADDSRDKQEYRAVRLRDGMRNFKGSKRNCGEQGEGCATRPCCSGLSCVGSRPGGLCQY | 1 |
| MMSKLGVLLTICLVLFPLTALPLDGDQPANRPAERAQDDTSAAQNPWFDHVKRCCTVCTTGCVVCC | 0 |
| MKLTCMMIVAVLFLTAWTFATAEDPRNGLENLFSKAHHEMKNPEDSKLNKRCVKYLDPCDMLRHTCCFGLCVLIACI | 0 |
| CQLNTADDSRDEREYRAVRLRDAMRNFKGSKRDCGAQGEGCYTRPCCAGLSCVGGHSGGLCQY | 1 |
| MKLTCLMIVAVLFLTAWTFVTADDSRNGLENLSPKAPHEMKNPEASKSNKRYECYLLVHFCGINGGLCCSNLCLFFVCLTFS | 0 |
| MMSKLGVLLTICLLLFPITADPLVEDQPADLPAERMQDIATEQHPLFDPVKRCCDRPCSIGCVPCCLP | 0 |
| MMSKLGVLLITCLLLFPLTAVPLDGDQHADRPAERLQDDISSENHPFFDPVKRCCNAGFCRFGCTPCCY | 0 |
| MMSKLGVLLTICLLLFPLTALPMDGDEPANRPVERMQDNISSEQYPLFEKRRDCCTPPKKCKDRQCKPQRCCAGR | 1 |
| MKLTCVMTVAVLFLTAWTFVTAEDPRDGLRNLLSNARHEMKNPEASKLNERCLGFGEACLMLYSDCCSYCVGAVCL | 0 |
| MSKLGALLTICLLLFSLTAVPLDGDQHADQPAQRLQDRLPTEDHPLYDPVKRCCDDSECDYSCWPCCIFS | 0 |
| ACSQVGEACLPQKPCCPGCLCNHIGGMCHH | 0 |
| MKLTCMMIVAVLFLTAWTFVTADDSGNVLENLFSKAHHEMKNPKDSKLNKRCLDGGEICGILFPSCCSGWCIVLVCA | 0 |
| MEGRRLAAVLILAICMLSPGASASRRHVVSLCMLPADTGPCFASMPRYYFDMDSYDCTTFVYGGCDGNGNNFRTYVECYGTCGLE | 1 |
| MGRLTLVLCLLLLLLPTTQACYIRDCPEGGKRDVHETHPAKPCKCCSVRQCGAEYLLWSWG | 0 |
| VLIIAVLFLTACQLTTAETSSRGKQKHRALRSTDKNSRMTKRCTPAGDACDATTECCILFCNLATKKCQVPTFP | 1 |
| VLIIAVLFLTACQLTTAETSSRGKQNHRALRSTDKNSRMTKRCTPAGDACDATTECCILFCNLATKKCQVPTFP | 1 |
| MKLTSVVIVAVLFLAACQLTTSDGSRGTWKDRAVRSITKVSMLRWPCKVAGSPCGLVSECCGTCNVLRNRCV | 0 |
| MKLTCVLIIAVLFLMDNQLITADYSRDEQVYRAVRLRDAMQKSKGSGSCAYISEPCDILPCCPGLKCNEDFVPICL | 0 |
| GCCPPSLCDPGCDEGCCPVVTPAC | 0 |
| VLIIAVLFLTACQLTTAETSSRGKQKHRALRSTDKNSRMSKRCTPPGGYCYHPDPCCSQVCNFPRKHCL | 1 |
| MKLTCVVIVAVLFLTAWTFVTADDSRNGLENLFPKARHEMKNPEASKLNKRCVDPGEFCGPGFGDCCTGFCLLVCI | 0 |
| MKLTCVMIVAVLFLTAWTFATADDPRNGLGNLFSNAHHEMKNPEASKLNERCLGFGEVCNFFFPNCCSYCVALVCL | 0 |
| VLIIAVLFLTACQLTTAETSSRGKQKHRALRSTDKNSRMSKRCTPPGGYCYHPDPCCSQYCNFPRKHCL | 1 |
| MMYKLGVLLTTCLLLFPLTAVQLDGDQPVDLPALRTQDFAPERSPWFDPVKRCCSRDCWVCIPCCPNGS | 0 |
| MMYKLGVLLTICLLLFPLTAVPLDGDQPVDLPALRTQDFAPERSPWFDPVKRCCSRDCLVCIPCCPYGSD | 0 |
| MKLTCMMIVAVLFLTAWTFVTADDSGNGLENLFSKKDHEMKNPKDSKLNKRCLDGGEICGILFPSCCSGWCIVLVCA | 0 |
| MMSKLGVLLIICLLLFPLTAVPLDGDQPADRYAERMQDDISSEHHPMFDAVRGCCGPTACIVGCKPCCG | 0 |
| MKLTCVLIITVLFLTASQLITADYSRDQRQYRAVRLGDEMRNFKGARDCGGQGEGCYTQPCCPGLRCRGGGTGGGVCQL | 1 |
| MKLTCVLITTVLFLTASQLITADYSRDKRQYRAVRLRDEMRNFKGARDCGEQGQGCYIYPCCPGLTCLGGGTGGGVCQPQ | 1 |
| MKLTCVLIITVLFLTASQLITADYSRDQRQYRAVRLGDEMRNFKGARDCGGQGEGCYTQPCCPGLRCRGGGTGGGACQL | 1 |
| MMYKLGVLLTTCLLLFPLTAVQLDGDQPVDLPALRTQDFAPEHSPWFDPVKRCCSQDCLVCIPCCPNGS | 0 |
| MMSKLGVLLTICLVLFPLTALQLDGDQPADRPAERTQDISSEQYRKFDQRQRCCRWPCPGSCRCC | 0 |
| MSTLGVLLTICLLLFPLTALPLDGDQPADQSAERPAERTQDDIQQHPLYDPKRRCCRYPCPDSCHGSCCYK | 1 |
| MKLTCLMIVAVLFLTAWTFVMADDSNNGLANLFSKSRDEMEDTDPSKLENRKTCQRRWDFCPGSLVGVITCCGGLICFLFFCV | 0 |
| MKLTCVMIVAVLFLTAWTFVTAVPHSSNALENLYLKARHEMENPEASKLNTRDDDCEPPGNFCGMIKIGPPCCSGWCFFACA | 0 |
| MKLTCVMTVAVLFLTAWTFVTAEDPRDGLKNLLSNAHNEMKNPEASTLNERCLGFGEACLILYSDCCGYCVGAICL | 0 |
| MKLTCVLIITVLFLTASQLITADYSGDKRQYRAVRLRDEMRNFKGARDCGEQGQGCYTRPCCPGLHCAAGATGGGSCQP | 1 |
| MKLTCVLIITVLFLTASQLITADYSRDQRQYRAVRLGDEMRNFKGARDCGGQGEGCYTQPCCPGLRCRGGGTGGGSCQP | 1 |
| MKLTCMMIVALLFLTAWTFVTAVDSKNELENRGGWGQAGGWGKLFPMARDEMKNSEVSKLDNKRKCAAAGEACVIPIIGNVFCCKGYCLFVCIS | 0 |
| MKLTCMMIVALLFLTAWTFVTAVDSKNELENRGGWGQAGGWGKLFLMARDEMKNSDVSKLDNRGRCVKDGDACVFPVVGSVFCCSGFCVFVCI | 0 |
| MKLTCMMIVAVLFLTALADDSRNGLENRNEQERNEKEMRDRGRCRPGGVVCGFPKPGPYCCSGWCFFVC | 1 |
| TASQLVTADYTRDKWQYPAASLRGGMWNLRDTRACSQVGEACLPQKPCCPGCLCNHIGGMCHH | 1 |
| MMSKLGVLLTICLLLFPLTAVQLDGDQPVDLPALRTQDFAPEHSPWFNPVKRCCSRHCWVCIPCCPNGS | 0 |
| VIIFAVLFLTACQLIATASYARSERKHPDLRLSSRNSKLSKRCLGSGELCVRDTSCCSMSCTNNICF | 0 |
| MNCYLILTVALLLTSAMTGTTTAGQLNKKGVTLREDDGFPCNAGNCACLPLDSYSYTCQSPTSSTANCEGNECVSEADW | 1 |
| MASVQSLACCCLLWLMLSVQLVTPGSPGTAQLSGHRTARVPAPHIMVPIYLCPILCKVGGVNTPFCNCTKKREMVSS | 0 |
| VLIIAVLFLTACQLTTAETSSRGKQKHRALRSTDKYSRMTKHCTPPEVGCLFAYECCSKICWRPRCYPS | 0 |
| VLIIAVLFLTACQLTTAETSSRGEQEHLALRSTDKNSRMTKRCTPRHGVCFYSYFCCSKACNPSSKRCL | 1 |
| VLVIAVLFLTACQLTTAETSSRGKQKHRALRSTDKNSKLTRGCTPPGGACGGHAHCCSQSCNILASTCNA | 1 |
| MKLTCMMIVAVLFLTAWTFVTADDSRNGLENLSPKARHEMKNPEASKSNKRYECYSTGTFCGINGGLCCSNLCLFFVCLTFS | 0 |
| VIIIAVLFLTACQLIATASYARSERKHPDLRLSSRNSKLSKRCLGSREQCVRDTSCCSMSCTNNICF | 1 |
| MPSVRSVTCCCLLWMMFSLTLVTPGSPGPAQLPGHRAARVPAEQMMEELCPDMCNRGEGEIICTCVLRRHVVFPSIRGRKRSMAV | 0 |
| MKLTCMMIVAVLFLTAWTFVTADDPRNGLENLFRKAHHEMKNPEASKLNKRCIDGGEMCDPFSSDCCSGWCIFFCI | 0 |
| MKLTCMMIVAVLFLTAWTFATADDPRNGLENLFSKAHHEMKNPKDSKLNKRCIDGGEICDIFFLNCCSGWCIILVCA | 0 |
| MMFKLGVLLTICLLLFSLNAVPLDGDQHADQPAERREDDISFENNPFFDPVKRCCMTCFGCTPCCG | 0 |
| MMSVQSVTCCCLLWLMLSVQLVTPGSPGTAQLPRDRTARVSAESVLEIICPYLCPLGIGALSFCNCPNKRDVVSSRITSRKRSMAV | 0 |
| MKLTCMMIVAVLFLTAWTFATADDPRNGLENLFSKAHHEMKNPKDSKLNKRCIDGGEICDYFFPNCCSGWCIILVCA | 0 |
| MKLTCMMIVAVLFLTAWTFATADDPRNGLENLFSKAHHEMKNPKDSKLNKRCLDAGEMCDSFNSKCCSGWCIILVCA | 0 |
| MKLTCMMIVAVLFLTAWTFVTADDSGNGLENLFSKAHHEMKNPKDSKLNKRCLNGGEICGILFPSCCSGWCIVLVCA | 0 |
| MASVQSVACCCLLWLMLSVQLVTPGSPGTAQLSGHRTARIPVCSVLCNAGVDVPFCDCTKKREMVSS | 0 |
| MASVQSVACCCLLWLMLSVQLVTPGSPGTAQLSGHRTARIPIVCSLLCKAGISIPIFCNCTKKRDVVS | 0 |
| MSKLGVLLTICLLLLPLTALPMDEDQPADQPADRMQDDISSEQYPLFDKRQKCCGKGMTCPRYFRDNFICGCC | 1 |
| MSKLGVLLTICLLLFPLTALPMDGDQSVDRPAERMQDDISSEQYPLFNQKRRCCGEGASCPVYSRDRLICSCC | 0 |
| MKLTCMMIVAVLFLTAWIFVTADDSANGLENLPQTIRHEMKNPEASKLNKRDCLPDYMLCAFNMGLCCSDKCMLVCLP | 0 |
| MSKLGVLLTICLLLFPLFALPLDGDQPADRPAERMQDDISSEKHPLFDKRQGCCNGPKGCSSKWCRDHARCCGRR | 1 |
| MASVQSVACCCLLWLMLSVQLVTPGSPGTAQLSGHRTARVPAPHIMVPIYLCPILCKVGGVNTPFCNCTKKREMVSS | 0 |
| MKLTCMMIIAVLFLTAWTFATADDSGNGLENLFSKAHHEMKNPEASKLNKRCLAKGDFCNLITQDCCDGICFIFCP | 0 |
| MKLTCLMTVAVLFLTAWTFATAEDPRNGLENLFSKAHHEMKNPEDSKLDKRCVKYLDPCDMLRHTCCFGLCVLIACI | 0 |
| MCILIVAVLFLTAWTFVMADDPRDEPDTVVRGGKLFSRARDEMNPAASKLNERDCVEVDYFCGIPFVFNGLCCSGNCVFVCTPQG | 0 |
| VVIVAVLFLTACQLITADDSRRTQKHRALRSTTKLSLSTRCRIPNQKCFQHLDDCCSRKCNRFNKCV | 1 |
| MTTSSYFLLVALGLLLYVCQSSFGNPHTRDSGTTPDRDHSCGGELVDRLVKLCPSNRKRRGFPSMLKARAKRNEAFLLQRDGRVIVGDCCDNYCTDERLKGYCASLLGL | 1 |
| MTTSSYFLLVALGLLLYVCQSSFGSPHTSDSGTTLVRRRLCGSELVTYLGELCLGNRKRRGFPSMLKARAKRNEAFLLQRDGRGIVEDCCYNDCTDEKLKEYCHTLQG | 1 |
| MKLTCMMIVAVLFLTAWTFVTAVPHSSNALENLYLKAHHEMNNPEDSELNKRCYDGGTSCDSGIQCCSGWCIFVCL | 0 |
| MKLTCMMIVAVLFLTAWTFVIADDPRDGPDTAVRGGKRFWKARNEMNSAASKLNKRECLEADYYCVLPFVGNGMCCSGICVFVCIAQKY | 0 |
| VLIVAVLFLTACQLTAAETSSRSKQKYRVLRSTDKNSKWTRECTPPGGACNIHPHCCEEFCDMANNRCLEM | 1 |
| LMIVAVLFLTACQLTTAETSSRGKQKYRVLRSADENPKWTRECTRPGGACYYDSHCCRHVCHEVFNTCM | 1 |
| MKLTCVMIVAVLFLTVWTFATADDSGNGLEKLFSNAHHEMKNPEASKLNKRCKQADEPCDVFSLECCTGICLGFCTW | 0 |
| MKLTCMMIVAVLFLTAWTFVTADDSRYGLKDLFPKERHEMKNPEASKLNQREACYNAGTFCGIKPGLCCSAICLSFVCISFDLIDVFSSP | 0 |
| MKLTCMMIVAVLFLTAWTFVTADDSGNGMENLFPKAGHEMENLEASNRGKPCHKEGQLCDPFLQNCCLGWNCVFVCI | 0 |
| MKLTCVLIIAVLFLTAYQLATAASHAKGKQKHRALRPADKHFRFTKRCNNRGGGCSQHPHCCSGTCNKTFGVCL | 1 |
| MKLTCVMIVAVLFLTAWTFVTADDSKNGLENHFWKARDEMKNREASKLDKKEACYAPGTFCGIKPGLCCSEFCLPGVCFGG | 0 |
| MKLTCVLIIAVLSLTAYQLATAASHAKGKQKHRALRPADKHFRFTKRCNNRGGGCSQHPHCCSGTCNKIFGVCL | 1 |
| MKLTCMMIVAVLFLTAWTFVTADDSGNGLENLFSKAHHEMKNPEASKLNERCIEYLEPCDFLRHTCCVGVCLLMACI | 0 |
| MKRTCALIVVLLFLTAYQLTTTDDSRGRKGYRAERARTRITNSKLLKLTKRCVEPGSPCSKYDNECCDACMLSHPNPPVCIE | 0 |
| MSKLGALLTICLLLFPITALLMDGDQPADRPAERMDYDISSEVHRLLERRHPPCCMYGRCRRYPGCSSASCCQRG | 1 |
| MKLTCMVIVAVLFLTAWTSVMADGSINRPDIAEGWQKFFSKARDEMKNRAASELNKRCAGIGSFCGLPGLVDCCSGRCFIVCLP | 0 |
| MSKLGVLLTICLLLFPITALPMDGDQPADRLAERMQDNISSEEHPFEKRQRLCCGFPKSCRSRQCKPHRCCGR | 1 |
| MKLTCMMIVAVLFLTTWTFATAITRNGLENLFPKEHHEMKNPEASKLNKRCVPYEGACNWLTQNCCDAVCVVFFCL | 0 |
| MKLTCVMIVAVLFLTANTFVTAVPHSSNVLENLYLKARHEMENPEASKLNTRYDCEPPGNFCGMIKVGPPCCSGWCFFACA | 0 |
| VLIIAVLFLTACQLTTAVASSRGEQKHRALRSTDKKFKVALLCSPPGSYCFGPAACCSNFCSTLSDVCQESWSG | 1 |
| MSKLGVVLFLTACQLITPDSSRDKQEDPVVRSSDKVQRSKHRKLAKRCSEVGAACDTESNICCSGECFAVQGSTFGICE | 1 |
| MKLTCMMIVAVLFLTAWTFVTAVPHSSNALENFYLKAHHEMNNPEDSQLNKRCYDSWTACDSPKLCCSGWCLFVCV | 0 |
| MMIVAVLFLTAWTFVMADDPRDGPDTAVRGGKRFWKARNEMNSAASKLNKRECLEADYYCVLPFVGNGMCCSGICVFVCIAQKYKTVVTSSTPLCATWLDL | 0 |
| MIRMGFFLTLTVAVLLTSLICTEAVPTDKRGMERLFDHVLLKDQRQCPYCVVHCCPPSYCQASGCRPP | 0 |
| MKLTCMMIVAVLFLTAWTFVTADDSGNGLENLFSKAHHEMKNPKDSKFNKRCLDGGEICGILFPSCCSGWCIVLVCA | 0 |
| MKLTCVMIVAVLFLTANTFATADDPRNGLRDLFSIAHHEMKNPEASKLNEKCYGFGEACLVLYTDCCGYCVLAVCL | 0 |
| MMKLLMTFVLLLMILPLCQPSGRRQLPAMPFPDSSITPKPTVPPDCAFKSCQTKAECCNGYYFCGAGFCIPNMNKIFDYGK | 0 |
| VIIIAVLFLTACQLTTAETSSRGKQKHRALRSTDKNSKLTRGCTPPGGACGGHAHCCSQSCNILASTCNA | 1 |
| MKLTCMMIVAVLFLTAWTFATADDPGNGLRDLFSIAHHEMKNPEASKLNEKCYGFGEACLVLYTDCCGYCVLAVCL | 0 |
| VLIIAVLFLTACQLTTAETYSRGRQKHRARRSTDKNSKWTRECTRSGGACNSHDQCCANFCRKATSTCM | 1 |
| MKLTCMMIVAVLFLTAWTFATADDPRNGLRDLFSIAHHEMKNPEASKLNEKCYGFGEACLVLYTDCCGYCVLAVCL | 0 |
| MTFLLLLVSVCMMATGEERTKRDVCELPFEEGPCFAAIRVYAYNAETGDCEQLTYGGCEGNGNRFATLEDCDNACARY | 0 |
| MKLTCMMIVAVLFLTTWTFATAITSNGLDNRFSKAHHEMKNRRASRLNKSCHLGGEYCGLFEVCCYGDCFIMCW | 0 |
| MKLTCVLIISVLFLTASQLITAVYSRDKQQYRAARLRDEMRNLKGARDCGEQGQGCYTRPCCPGLECLGGGTGGGVCQP | 1 |
| MKLTCMMIVAVLFLTAWTLVMADDSNNGLANHFWKSRDEMEDPEASKLEKRDCQDKWEFCIVPILGFVYCCPGLICGPFVCV | 0 |
| VLIIAVLFLTACQLTTAETSSRGKQKHRALRSTDKNSKLTRGCTPPGGACGGHAHCCSQSCNILASTCNA | 1 |
| MPSVRSVTCCCLLWMMFSVQLVNPGSPGTAQLSRHRTARVPAEPILEIICPEMCDEGVGEEPFCHCTEKRDAVSSRIRRRKRSTAV | 0 |
| VIIIAVLFLTACQLTTAETSSRGKQKHRALRSTDKNSKLTRGCTPPGGACGGHAHCCSQSCDILASTCNA | 1 |
| VCCPHGGCHQICQCCGC | 0 |
| MPSVRSVTCCCLLWMMFSVQLVNPGSPGTAQLFRHRTARVLAELILEIICLEMCEEGVGEEPFCHCTEKRDAVFSRIRRRKRSTAV | 0 |
| MPSVRSVTCCCLLWMMFSVQLVNPGSPGTAQLSRHRTARVPAEPILEIICPEMCDKGVGEEPFCHCTEKRDAVSSRIRRRKRSTAV | 0 |
| ICCPQGGCHQLCQCCGC | 1 |
| MKLTCMMIVAVLFLTAWTFVMADDPRDGPDTAVRGGKRFWKARNEMNSAASKLNKRECLEADYYCVLPFVGNGMCCSGICVFVCIAQKY | 0 |
| MKLTCMMIVAVLFLTAWTLVMADDSNNGLANLFSKSRDEMEDPEASKLEKRDCQALWDYCPVPFLSSGDCCIGLICGPFICIGW | 0 |
| MKLTCMKIVAVLFLTAWTFVTADDSRNGLEYLFPKAHYEMNPEASKLNKKQDCAAGGQFCGFPKIGGPCCSGWCLGVCA | 0 |
| MKLTCMMIVAVLFLTASIFITADNSRNGIENLPRMRRHEMKKPKASKLNKRGCLPDEYFCGFSMIGALLCCSGWCLGICMT | 0 |
| MKLTCMMIVAVLFLTAWTFVTADSRNGLEYLFPKAHYEMNPEASKLNKKRDCVAGGHFCGFPKIGGPCCSGWCFFVCA | 0 |
| MMSKLGILLTICLLLFPLTAVPLDGDQPADRPAERMQDDISSEHHPFFDPVKRCCRIACNLKCNPCCG | 0 |
| MKLTCMMIVAVLFLTAWTFVTADDSGNGLENLFSKAHHKMKNPEASKLNKRCRLGAESCDVISQNCCQGTCVFFCLP | 0 |
| MLKMGVLLFTFLVLFPLATLQLDADQPVERYVENKQDLNPDERREIILHALGQRLCCWKEWCHARCTCCGSANIHGAVLGGFIHNNSVC | 0 |
| MLKWSVLFIFLVLFTLPTLQLDADQLVERHVENKQLLNPDERRRIISDALGQRICCPQGGCHQLCQCCGC | 0 |
| MLKMGVVLFIFLVLFTLPTLQLDADQLVERHVENKQLLNPDERRRIISDALGQRICCPQGGCHQLCQCCGC | 0 |
| MLKWSGAIHFLVLFTLPTLQLDADQLVERHVENKQLLNPDERRRIISDALGQRICCPQGGCHQLCQCCGC | 1 |
| MLKMGVVLFIFLVLFTLPTLQLDADQLVERHAENKQLLNPDERRRIISVALGQRVCCPHGGCHQICQCCGC | 0 |
| MMSKLGVLLAICLLMLPLTALPLDGDQPQERKEDGKSAALQPWFDPVKRCCQAACSPWLCLPCCG | 0 |
| MMSTLVVLLTICLLMLPLTARQLDADQLADQLAERMEDISADQNRWFDPVKRCCMRPICMCPCCVNG | 0 |
| MKTGMIICLLLIAFMDADGSPGDTLYSQKTADTDSGMKRFQKTFQKRRCVFCPKEPCCDGDQCMTAPGTGPFCG | 0 |
| MKLTCMMIVAVLFLTAWTFVTAVPHSSNALENLYLKAHHEMNNPKDSELNKRCYDGGTGCDSGNQCCSGWCIFVCL | 0 |
| MKLTCMVIVAVLFLTANTFVTAVPHSSNALENLYLKAHHEMNNPKDSELNKRCYDGGTGCDSGNQCCSGWCIFVCL | 0 |
| MKLTCMMIVAVPFLTAWTFVMADDPRDGPDTAVRGGKRFWKARNEMNSAASKLNKRECLEADYYCVLPFVGNGMCCSGICVFVCIAQKY | 0 |
| MKLTCMMIVAVLFLTAWTFVTADDPRDGPDTAVRGGKRFWKARNEMNSAASKLNKRECLEADYYCVLPFVGNGMCCSGICVFVCIAQKY | 0 |
| MMSKLGVLLTICLLLFPLTALPMDGDQPQERKEDEKSAALQPWFDPVKRCCQAGCSRYMCLPCCQ | 0 |
| MKLTCVMIVAVLFLTTWTFVTADDSRYGLKNLFPKARHEMKNPEASKLNKRDGCYNAGTFCGIRPGLCCSEFCFLWCITFVDSG | 0 |
| MKFFTCLLLLLVVLTVVFDNVDACDRSCTGVMGHPSCATCCACFTSAGKRHADGQHSRMKVRTGAKNLLKRMPLH | 0 |
| VVIVAVLLLTACQLITANDSRGTQKHRALRSDTKLSMSTRCKGKGASCRRTSYDCCTGSCRSGRCG | 1 |
| MKLTCVIVAVLFLTAWTFVTADDSGNGLENLFSKAHHEMKNPKDSKLNKRCLDGGEICGILFPSCCSGWCIVLVCA | 0 |
| MNTAGRLLLLCLALGLVFESLGIPVADDVEAVRDTDPDEKDPSVHNSLKAVYGDCGGERCRFGCCKTDDGEEKCQHFGCP | 1 |
| MKLTCMMIVAVLFLTAWTFVTADDSRNGLKNLFPKARHEMKNPEASKLNKRYGCSNAGAFCGIHPGLCCSELCLVWCT | 0 |
| MKLTILLLVAALLVLTQARTERRRVKSRKTSSTYDDEMATFCWSYWNEFQYSYPYTYVQPCLTLGKACTTNSDCCSKYCNTKMCKINWEG | 1 |
| MKLTCMMIIAVLFLTAWTFVTADDSGNGLENLFSKAHHEMKNPEASKLNKRCTQSSEFCDVIDPDCCSGVCMAFFCI | 0 |
| MMSKLGVLLTICLLLFPLTALPMDGDQTADKPAQRKLVISPTRRYWTRSACCYIEEGEKCPASCKLCC | 0 |
| MKLTCMMIVAVLFLTAWTFVTADDSKNGLENHFWKARDEMKNREASKLDKKEACYPPGTFCGIKPGLCCSELCLPAVCVGG | 0 |
| MKLTILLLVAALLVLTQALTERRRVKWQKRSSTYDDEIATFCWSYWIGFQYSYPYTYVQPCTALGKACTANSDCCSKYCNTKICKINWEG | 0 |
| MMSKLGVLLTICLLLFPVTALPMDGDQPADRLVERMQDNISSEQHPFFEKRRGGCCTPPRKCKDRACKPARCCGPG | 1 |
| MKLTCMMIVAVLFLTAWTFVTADDSKNELENRGGWEPFPEARDEMKNPKAFELTECSSYGEFCFPGIKDCCMGYCFIMCLPTSPHD | 0 |
| MKLTCMMIVAALFLTAWTFVTADNSKYELENRGGVWKLFPMARDEIKETARESERCLERGEPCIMPIVGLFACCSSVCIIVCI | 0 |
| MMSKLGVLLTICLLLFPLTALPMDGDQPADRPAERMQNDISSERHPFFDRSKQCCHLAACRFRCTPCCW | 0 |
| MEKLTILLLVAALLVLTQARTERRRVKSRKTSSTYDDEMATFCWSYWNEFQHSYPYTYVQPCLTLGKACTTNSDCCSKYCNTEMCKINWEG | 1 |
| VLLIAVLFLAACQLITSAIYVRSERKHPDMRSSIRNSMLTKKRCLTTGETCWLASSCCSFSCTNNVCF | 0 |
| VLLIAVLFLAACQLITSASYVRSERKHPGMRSSIRNSMLTKKRCLTTGETCWLASSCCSFSCTNNVCF | 0 |
| VLLIAVLFLAACQLITSASYVRSERKHPDLRSSIRNSMLTKKRCLTTGETCWLASSCCSFSCTNNVCF | 0 |
| VLLIAVLFLAACQLITSASYVRSERKHPDMRSSIRNSMLTKKRCLTTGETCWLASSCCSFSCTNNVCF | 0 |
| MMSKLGALLTICLLLFPITALLMDGDQPADRPAERMDYDISSEVHRLLERRHPPCCMYGRCRRYPGCSSASCCQGG | 1 |
| VVIVAVLFLTACQLITADDSRRTQKHRALRSTTKRATSNRPCKKTGRKCFPHQKDCCGRACIITICP | 1 |
| VVIVAVLFLTACQLITADDSRRTQKHRALRSTTKRARSNRPCKKSGRKCFPHQKDCCGRACIITICP | 1 |
| VLLIAVLFLAACQLITSASYVRSERRHPDLRSSIRNSMLTKKRCLTTGEYCWLASSCCSYSCTNNVCF | 0 |
| VLLIAVLFLAACQLITSAGYVRSERKHPDLRSSIRNSMLTKKRCLTTGEYCWLASSCCSYSCTNNVCF | 0 |
| VVIVAVLFLTACQLITADDSRRTQKHRALRSTTKGATSNRPCKTPGRKCFPHQKDCCGRACIITICP | 0 |
| MKLTCMMIVAVLFLTAWTFVTAVPHSSNVLENLYLKARHEMENPEASKLNTRYDCEPPGNFCGMIKVGPPCCSGWCFFACA | 0 |
| VLLIAVLFLAACQLITSASYVRSERKHPDLRSSIRNSMLTKKRCLTTGEYCWLASSCCSYSCTNNVCF | 0 |
| VLLIAVLFLAACQLITSASYVRSERKHPDLRSSIRNSMLTKKRCLTTGEYCWLASSCCSYSCTDNVCF | 0 |
| MEKLTILLLVAALLVLTQARTERRRVKSRKTSSTYDDEMATFCWSYWNEFQYSYPYTYVQPCLTLGKACTTNSDCCSKYCNTKMCKINWEG | 1 |
| MKLTCMMIVAVLFLTAWTFVMADDPRDGPDTAVRGGKRFWKARNEMNSAASKLNKRECLEADYYCVLPFVGNGMCCSGICVFVCIDPKVLKLS | 0 |
| MKLTCMMIVAVLFLTAWTFVTAVPHSSNVLENLYLKARHEMKNPEASKLNEKCYGFGEACLVLYTDCCGYCVLAVCL | 0 |
| VLIIAVLFLTACQLTTAETSSRGKQKHRALRSTDENSKLTRGCTPPGGACGGHAHCCSQSCNILASTCNA | 1 |
| MMFKLGVLLTICLLLFPLTALQLDWDQPGDHMLDISSEIDDRWFDPVRKCCMRPICMCPCCIGP | 0 |
| MMSKLGVLLTICLLLFPLSALPLDGDQPADQPAERMQDISPEQNPLFHPDKRGCCPFPACTTHIICRCC | 0 |
| VLIIAVLFLTACQLTIAETYSRGRQKHRARRSTDKNSKWTRECTRSGGACNSHTQCCDDFCSTATSTCI | 1 |
| MSGTTVLLLTCLFLVTMATSDCDLYDDSCTGTEICCTPPGDYQGNCMEGEDCPSGGR | 0 |
| MMSKLGALLIICLLLFSLTAVPLDGDQHADQPAQRLQDRIPTEDHPLFDPNKRCCPPVACNMGCKPCCG | 0 |
| MMSKLGVLLTICLLLFPHTAVPLDGDQPADQPAERLQDDISSEHHPMFNSIRRREQNQFKSFTSVKLLDSRGERCCGPTACLAGCKPCCG | 0 |
| MMSKLGVLLTICLLLFPHTAVPLDGDQPADLPAERLQDDISSEHHPMFNSIRRREQNQFKSFTSVKLLDSRGERCCGPTACLAGCKPCCG | 1 |
| VLIIAVLFLTACQLTTAETYSRGRQKHRARRSTDKNSKWTRECTRSGGACNSHTQCCDDFCSTATSTCI | 1 |
| MKLTCMMIVAVLFLTAWTFVMADDPRDGPDTAVRGGKRFWKARNEMNSAASKLNKRECLEADYYCVLPFVGNGMCCSGICVFVCIAQKVLKLS | 0 |
| VLIIAVLFLTACQLTTAETYSRGRQKHRARRSTDKNSKWTRECTHSGGACNSHNQCCNAFCDTATRTCV | 1 |
| MNLACVLIVAVLFLTASQLATAASYARDKQEYPAVRSSDEMQDSEDLTLTKECTDDSQFCDPNDHDCCSGECIDEGGRGVCAIVPEHV | 0 |
| MKLTCVLIIAVLFLTAYQLATAASHAKGKQKHRALRPADKHFRFTKRCNNRGGGCSQHPHCCSGTCNKTFGVCL | 1 |
| VLIIAVLFLTACQLTTAETYSRGRQKHRARRSTDKNSKWTRECTHSGGACNSHDQCCNTFCDTATRTCV | 1 |
| MMSKLGVLVTICLLLFPLTALPLDGDQPADHPAKRTQDHNLASPISAWIDPSHYCCCGGGCTDDCVNC | 0 |
| MMLKMGVVLFICLVLFPLATLQLDADQPVERLLENKQDLNPEKRSDFKMRAWRARGCCDYDWCDEFCYCCE | 1 |
| MMSKLGVLLTICLVLFPLTALPLDGDQPANRPAERAQDDTSAAQNPWFDHVKRCCTVCTTGCVVCC | 0 |
| MMSKLGVLLTICLLLFPLFALPQDGDQPADRPAERMQDDISSEQNSLLEKRVTDRCCKGKRECGRWCRDHSRCCGRR | 1 |
| VLIIAVLFLTACQLTTAETYSRGRQKHRARRSTDKNSKWTRECTHSGGACNSHTQCCDDFCSTATSTCI | 1 |
| VCKQPAETGRCCRVIQRYYFDMDSYDCKKFNYKGCGGNGNNFRTYNECYVTC | 1 |
| MGVVLFIVLVLFPLATLQLDADKPVERYAENKQLLNPDERRGIILHALGQRVCCSFGSCDSLCQCCDGLAPNIHGAVLGGFIRTMSAFVDFTYHCATSLV | 0 |
| MMSKLRVLLTICLLLFPLSALPLDGDQPADQPAKRMWNGKLAARKPRFDKYDLVRGCCPPQWCGPDCTSPCCG | 1 |
| MMSKLGVLLTICLLLFPLSVLPLDGDQLADQPARHAQSAERNARFHPVKRCCPFPMCYQVPHCPCCG | 0 |
| MMGKLGVVLFICLVLFPLETLQLEGGQQADRHVDQLEGNPNRETRTIEVRCTTMNCLKGHCGCSPDCGSC | 0 |
| AVLLLTACQLITAEDSRDTQKHRALRSDTKLSMLTLRCATYGKPCGIQNDCCNICDPARRTCT | 1 |
| MLNMGVVLFICLVLFPLATLQLDADQPVERHAENKRNLNPHERREIIMLALRGVNCCHPNLCIGRSALGRKCTCCG | 0 |
| MKLTCMMIVAVLFLTAWTFVTADDSGNGLENLFSKAHHEMKNPKDSNLNQRCLDGGEICGILFPSCCSGWCIVLVCA | 0 |
| MKLTCMMIIAVLFLTAWTFVMADDPRDEPEARDEMNPAASKLNERGCLEVDYFCGIPFANNGLCCSGNCVFVCTPQGK | 0 |
| MEALTTLKVCVLVVLTTTVELAPPANAGPVHGLWRRIQKRQCPVTGGPTPMYHCMMACMTTSTEYWCQHEYCSDCAE | 0 |
| MMSKLGALLTICLLLFSLTAVPLDGDQHADQPAQRLQDRIPTEDHPLFDPNKRCCDDSECSYSCWPCCYG | 0 |
| MMSKLGVLLITCLLLFPLTAVPLDGDQPADRPAERLQDDISSENHPFFDPVKRCCNAGFCRFGCTPCCY | 0 |
| MSKLRALLIICLLLFPLTAVPLDGDQPADRPAERMQNDISSEHHPMFDAIRGCCGAFACRFGCTPCC | 0 |
| DVLLSACKLPAKTGPCYSFVRKYYFDWDSVNCKTFIYGGCDGNSNNFCTYNECYSRCGPVN | 1 |
| MMSKLGVLLTICLLLFPLSVLPLDGDQPADQPAERMQDISAEQNPWFDPVKRCCNWPRCNVYLCGPCC | 0 |
| RVLLTICLLLFPLTAIPLGGDQPAERMRNVRSAVQDPRFDSVGWCCPIQGCILGCTPCC | 0 |
| TICLLLFPLTVVPLDGDQPAHQPAVRKHNIKSAVQLRQWDEEQQCCTGQCHICWPCC | 0 |
| MMFKLGVLLTICLLLFPLTGTALDGDQLAEHMLDISSGINDRWFDPVRKCCMRPVCTCPCCS | 0 |
| MMSKLGVLLTICLLLFPLSALPLDGDQPADQPAERVQDISPDQNPLFHLVKRGCCPYPKCIHVTFCKCC | 0 |
| RVLLTICLLLFPLSALPLDGDQPADQPARHMQSAERNPRFDPVKRCCPYPSCIDIPFCDCCG | 0 |
| MSKLEVVFFICLVLVPLATFQLEADQPVERHAENEYAENEYDLNPHDTSGIMIRAIRGCCVKQGCWNVETCTCCPADPYIPKPFPTRST | 0 |
| VLIIAVLFLTACQLTTAETYSRGKQKHRARRSTDKNSKWTRECTRSGGACNSHTQCCDDFCSTATSTCI | 1 |
| MKLTCVLIIAVLFLTASQLITADYSRDKQEYGAERLRDAMGKFKGSRSCGHSGAGCYTRPCCPGLHCSGGHAGGLCV | 0 |
| MMSKLGVLLTICLVLFPLTALPLDGDQPADRHAERMQDDTSAAQNPWFDHVKRCCTVCTTGCVVCC | 0 |
| MKLTCVLIITVLFLTASQLITADYSRDQRQYRAVRLGDEMRNFKGARDCGGQGEGCYTQPCCPGLRCRGGGTGGGVCQL | 1 |
| MTSVQSVTCCCLLWLMLSLTLVTPGSPGPAQLPGHRAARVPAEPILEEICPDMCNSGEGEIFCTCGSRQFVVTLPVIERKRSMAV | 0 |
| MKLTCVLIITVLFLTASQLITADYSRDQRQYRAVRLGDEMRNFKGARDCGGQGKGCYTQPCCPGLRCRGGGTGGGVCQP | 1 |
| VLIIAVLFLTACQLTTAETYSRGRQKHRARRSTDKNSKWTRECTRSGGACNSHTQCCDHFCSTATSTCI | 1 |
| MIRMGFFLTLTVAVLLTSLTCSEAVPTDKREMERLFDRILLKDQRQCPYCVVHCCPPSYCQASGCRPP | 0 |
| WRKTPEGADRTFLQQECYQEIRQKRCRTVGRPCHYSAQCCSDFCAHTISNYYYIKRCS | 1 |
| MNCYLILTVALLLTSAMTGTTTAGQLNKKGVTLREDDRFPCNPGGCACRPLDSYSYTCQSPSSSTANCEGNECVSEADW | 1 |
| MKLTCVLIITVLFLTASQLITADYSRDQRQYRAVRLGDEMRTFKGARDCGEQGQGCYTRPCCPGLHCAAGATGGGSCQP | 1 |
| MKLTCVLIITVLFLTASQLITADYSRGQRQYRAVRLGDEMRNFKGARDCGGQGEGCYTQPCCPGLRCRGGGTGGGSCQP | 1 |
| MKLTCVMIVAVLFLTAWTVVTAEPHSSNVLENLYLKAHHEMENPEASKLNTRDRCQRANFVCDAFHHAAVCCEGVCVLVCAW | 0 |
| MKLTCMVIIAVLFLMANQLITADYSRDEQVYRAVRLRDAMQKSKGSGSCADLSEACDILLCCPGLKCNEDFIPICL | 0 |
| MKLTCMMIVAVLFLTAWTFVTADDSGNGLENLFSKAHHEMKNPKDSKLNQRCLDGGEICGILFPSCCSGWCIVLVCA | 0 |
| VLIIAVLFLTACQLTTAETYSRGRQKHRARRSTDKNSKWTRECAHSGGACNSHDQCCNAFCDTATRTCV | 1 |
| MKLTCVIVAVLFLTAWTFVMADDPRDGPDTAVRGGKRFWKARNEMNSAASKLNKRECLEADYYCVLPFVGNGMCCSGICVFVCIAQKY | 0 |
| MMSKLGVLLTICLLLFPLFALPQDGDQPADRPAERMQDDISSEQNPLLEKRVGERCCKNGKRGCGRWCRDHSRCCGRR | 1 |
| MMSKLGVLLTICLLLFPLFALPQDGDQPADRPAERMQDDLSSEQHPLFEKRIVDRCCNKGNGKRGCSRWCRDHSRCCGRR | 1 |
| MKLTCVMIVTVLFLTAWTFVTADDSTYGLKNLLPNGRHEMMNPEAPKLNKKDECSAPGAFCLIRPGLCCSEFCFFACF | 0 |
| MKLTCMMIVAVLFLTAWTVVTAEPHSSNVLENLYLKAHHEMENPEASKLNTRDRCQRANFVCDAFHHAAVCCEGVCVLVCA | 0 |
| MMSKLGVLLTICLLLFPLTAVPLDGDQPADQPAERMQDDISSEQHPFFDPVKRCCVVCNAGCSGNCCP | 0 |
| VLIIAVLFLTACQLIATASYARSERKHPDLRLSSRNSKLSKRCLGSGELCVRDTSCCSMSCTNNICF | 0 |
| MKLTCMMIVAVLFLTAWTVVTAEPHSSNVLENLYLKAHHEMENPEASKLNTRDDDCEPPGNFCGMIKIGPPCCSGWCFFACA | 0 |
| MPSVRSVTCCCLLWMMFSLTLVTPGSPGPAQLPGHRAARALAEPILEEICPDMCNSGEGEIFCACGSRQFVVTLPVIERKRSMAV | 0 |
| MKLTCMMLVAVLFLTAWTFVTANVSRNGLENLFPEERHEMMNPEAAKLNNRDCVKAGTACGFPKPEPACCSSWCIFVCT | 0 |
| VLIIAVLFLTACQLTTAETYSRGRQKHRARRSTDKNPKWTRECTHSGGACNSHDQCCNAFCDTATRACV | 1 |
| MKLTCMMLVAVLFLTAWTFVTANVSRNGLENLFPEERHEMMNPNAAKLNNRDCVKAGTACGFPKPEPACCSSWCIFVCT | 0 |
| MPSVRSVTCCCLLWMMFSLTLVTPGSPGPAQLPGHRAARVPAEQMMEELCPDMCNRGEGEIICTCVLGRHVVSPSIRGRKRSMAV | 0 |
| MKLTCVVIVAVLFLTAWIFITADDSTNGLENRFRKARDNMKNAKASTLAEKKACVELGEICATGFFLDEECCTGSCHVFCVL | 0 |
| MMSKLGVLLTICLLLFPLTAVSLDGDQPADLPELRAQDFAPERSPWFDPVRRCCSQDCSVCIPCCPW | 0 |
| MKLTCMMIVAVLFLTAWTFVMADDPRDGPDTAVRGGKRFWKARNEMNSAASKLNKRECLEADYYCVLPFVSNGMCCSGICVFVCIAQKY | 0 |
| VLIIAVLFLTACQLTTAETYSRGRQKHRARRSTDKNSKWTRECTRSGGACYSHNQCCDDFCSTATSTCV | 1 |
| MSKLGVFLTICLLLFPLTALQLDGDQPADKPAQRKLKILPKRKHWTRFTCCYEEECPPSCKLCC | 0 |
| VLIIAVLFLTACQLTTAETYSRGRQKHRARRSTDKNSKWTRECTRSGGACYSHNQCCDDFCSTATSTCI | 1 |
| MMSKLGVLLTICLLLFPLTAVQLDGDQPADLPALRAQDFAPERSPWFDPVKRCCSQDCWVCIPCCPN | 0 |
| MMSKLGVLLTICLLLFPLTALPMDGDQPQERKEDGKSAALQPWFDPVKRCCQAACSPWLCLPCCG | 0 |
| MMSKLGVLLTICLLLFPLTALPMDGDQPQERKEDGKSAALQPWFDPVKRCCRAACSPWLCLPCCG | 0 |
| MNNTGRLLLLCLALGLVFGSLGKFVTDNGDADRDAGPVKRAAFIADRMTEVDCGGVSCEFHCCETINGVQRCREINCN | 0 |
| MKLTFVLIVAVLVLAVCNFTVADKANNAEAPEQEKRACTPNGSYCNILSGKLNCCSGWCLALICAG | 0 |
| MKLRVTFLLVLVILPSVTGEKSSERTLSGAALRSDRRTCSNKGEQCEDDSDCCWHLCCVDNKCAKLILLCNL | 1 |
| GRCRGFREDCSQHRDCCGDLCCNGNTCVITVIACPKW | 1 |
| YDAPYCSQEEVRECHDDCSGNPVRDACQCAYDPAGSPACDCYCVEPW | 1 |
| MLKMGVVLFIVLVLFPLATLQLDADQPVERYAENKQLLNPDERRGIILHALGQRVCCPFGGCHELCTAATVSAEHPWRCAGRFIQQRQRLLISCIIAPRLLSKNDEHDCTLVQISCSF | 0 |
| MMTRVFLAMFFLLVLTKGWPRLYDGDCTRGPNMHITCFKDQSCGLIVKRNGRLSCTLNCKCRRNESCLPSEEVDWDNRNMKIVICPKPWF | 0 |
| MKLTCMVIIAVLFLAACQLIIADYSRDMQEHPADRSRIKMKNFRRPTLTKRCVSPGGVCQHKDECCSDRCEQSAIVSICKQR | 0 |
| YDAPYCSQEEVRECDDDCSGNPVRDACQCAYDPAGSPACDCYCVEPW | 1 |
| MKLCLTFALVLAVLFLTACQLIIADYARDTQGYPALRSITKMKNSRRSRLTKRCDGHGVLCDYDSECCSGECTTTGAIEYCK | 0 |
| MKLTCVVIVAVLFLTACQFNAADDSRDKQENRLARLLHKKLNSADSGLLTKRCMEPDRRCSEWSPERCCTKCDYYRSICV | 1 |
| SSMCKQSNGPCSGNFDCCGNLCCTWDNTCGDKIGGC | 1 |
| MKLTCVVIVAVLFLTACQLITADDSRSTQKHRALRSTIKHSMLTRSCTPPGGPCGYYNNCCSHQCNINRNKCE | 1 |
| DCTPQNVRCEEKSECCPNLECKCTSAPDCNFYKCRT | 1 |
| VIIIAVLFLTACQLIATASYARSERKHPDLRLSSRNSKLSKRCLGSREQCVRDTSCCSMSCTNNICC | 1 |
| MKLFLVIVLNLMLLSLSTGAETSDNRASRSATALRDRLRRPKRCRNRFEECWEDSECCEELCCDGPNYCKDSIGRCH | 1 |
| QCTTRHQPCDEDEECCPYLECKCFTRPDCQSGYKCKP | 1 |
| RPKCCCVCGVVGRKCCSTWKDCHPVHLPCPSSG | 1 |
| MEKLTTLILVATVLTTIQVLVKSDRERPPKRTVEQYAAERLLALMRGSRQCKPKGDICEEDEECCPSLECECDSLPTCITNKRC | 0 |
| MKLVLAIILILMLISLSTGAETSDSRASMSATAETDGLLRVKSSMCKQSNGPCSGNFDCCGNLCCTWDNTCGDKIGGC | 0 |
| ACHTCDDGTECCDSRCSCPWNTCTCIPWX | 0 |
| YDAPYCSEEELQACDCSHNPVRDACLCQYDPAGSPACECFCVEPW | 1 |
| MKPSMTLLLILMILPSMTGEKSRERRLSSAAVKGFLRPARCTEFGKECNLPPECCGRMCCYNSLCMWSSKEC | 1 |
| TDVCKKSPGKCIHNGCFCEQDKPQGNCCDSGGCTVKWWCPGTKGD | 1 |
| DVCKKSPGKCIHNGCFCEQDKPQGNCCDSGGCTVKWWCPGTKGD | 1 |
| MKLTCVLIITVLFLTACQLTAAGNSRDKQEDPVVRSSGEVQRSEDIKLAKRCLESGSLCFAGYGHSSCCSGACLDYGGLGVGACR | 0 |
| MEKLTILILVATALLSIQVMVRGDGEKPLMGRIKRNAAAGLSALIRGKRCKGASALCEEDGECCSGDCKCMHASGCTNDINLRCAA | 0 |
| YDAPYCSEEELQACDCSHNPVRDACQCAYDPAGSPACDCYCVEPW | 1 |
| SCRNEGAMCSFGFQCCKKECCMSHCTDFCRNPD | 1 |
| SCRNEGAMCSFGFQCCKKKCCMSHCTDFCRNP | 1 |
| YDAPYCSQEEVRECQDDCSGNAVRDSCLCAYDPAGSPACECRCVEPW | 1 |
| YDAPYCSQEEVRECDDDCSGNPVRDACQCAYDPAGSPACDCFCVEPW | 1 |
| MLSSLIQAHERDSEESCKSYGGGPCPSGEDCCCPPGRSTGTCKRTCNNGSVCA | 1 |
| MGMRMMFTVFLSVVLATTLVSFTSGRRDKASHQKRDCPVTGGPNPFHHCKIACMSTGTEEYCNCVYCKDCVNSNGEKPAC | 1 |
| GDWGTCSWPGQECEHDSDCCGSFCCVGRRCLHIYFPCNLSRS | 1 |
| MNFYLLLTVTLLLASFTGGDARRIQGMDIYRHFVRRDCGRDCVGCDNPANCCCGGQTCVNGNKCE | 1 |
| RDCGKDCVGCDNPANCCCGGQTCVNGNKCE | 1 |
| RDCGRDCVGCDNPANCCCGGQTCVNGNKCE | 1 |
| CKGASALCEEDGECCSGDCKCMHASGCTNDINLRCAA | 1 |
| MNFYLLLTVTLLLASFTGGDARRIQGMDIYRHFVRRDCGKDCVGCDNPANCCCGGQTCVNGNKCE | 1 |
| MKLTCMMIVAVLFLTAWTFATADDPRNGLENLFSKAHHEMKNPKDSKLNKRCIDGGEICDIFFPNCCSGWCIILVCVYCKLP | 0 |
| HKWGACSLLGKDCGHSPDCCGDLCCIGGKCVMTDIPCHYFPF | 0 |
| MKLTCMVIIAVLFLTACQLITADLSSDEYHAVRSIDEMHDFKGSRATPECSRAGCKNVPCCSGLKCTGPQSGPVCQPE | 0 |
| TCSDLGQACVHESDCCAQMCCLNKKCAMTMPPCNFY | 0 |
| WPRLYDGDCTRGPNMHITCFKDQSCGLIVKRNGRLSCTLNCKCRRNESCLPSEEVDWDNRNMKIVICPKPWF | 1 |
| MEKLTVLILVATVLLTIQVLGQSDGDKHLKRRPKQYATKRLSALMKGHRQCTGAGFECEETPECCPNLTCKCSGSPLCARYRCKP | 1 |
| MEKLTILVLVATVLLAIQVLVQSDREKPLKGRVKQYAAKRLSALMRGPRQCTPKNQICEEDGECCPNLECKCFTRPDCQSGYKCRP | 0 |
| MRFYLLLTVTLLLTSFTGGDAGSVQGKKAQGRFVRRDCNTGCVPCGDRCCCVPHSCIDSKCLGHATPHRAY | 1 |
| DCPTRCPTTCANGWECCKGYPCVNKACSGCTHGK | 1 |
| MKLTCVVIVAVLFLTACQLITADDSRRTQKHRALRSTTKRATSNRPCKPKGRKCFPHQKDCCNKTCTRSKCP | 1 |
| MKLTCVVIVAVLFLTACQLITADDSRRTQKHRALRSTTKLSMLTRQCTPHGGSCGLVSTCCGRCNVPRNKCE | 1 |
| MMSKLGCLLTICLVLFPLTALPLDGDQPAERPAKRTQDDIPNGQDPLIDRQINCCPWPCPDSCHYQCCH | 0 |
| MMTRVFIAMFFLLALTEGWPRLTDSDCELGRNMHITCKQLDQCGVIEKKDGQLTCKLRCKCKPGKRCLRKENIDWSDITTRIYHCPWP | 1 |
| MRFYLLLTVALFLTSITGGDAGRMLRMRGGGHVVRRSCASNCVSCGSGCCCSPNSCVDSTCTHHSTPH | 0 |
| MEKLTVLILVATVLLTIQVLAQSDGDKHLMKRSKQYATKRLSALMRGHRQCIPQNVSCEEDDECCSNLECKCTSAPDCNFPKCRA | 0 |
| MKLTCVPIVAMLFLMACQLITADYSREKHGYSAEKSSDKIQDSFYSKLTKRCTDEGGDCDPGNHNCCRGSCLVLQHKAICGIVYTMVSR | 0 |
| FIPCTGSEGYCHSHMWCCNSFDVCCELPGPATCTREEACETLRIA | 0 |
| CLSEGSPCSMSGSCCHKSCCRSTCTFPCLIPX | 0 |
| CAPPGSSCNHTDNKCCISACCFGACVLPCQVP | 0 |
| GDWGMCSGIGQGCGQDSNCCGDMCCYGQICAMTFAACGP | 1 |
| TCSDLGQACVHESECCAQMCCLHKKCAMTHCNFY | 0 |
| GTCSYLGEGCKRDSDCCGHFCCGGKTCVITARPCKV | 1 |
| QCTGAGFECEETPECCPNLTCKCSGSPLCARYRCKP | 1 |
| QCTPKNQICEEDGECCPNLECKCFTRPDCQSGYKCRP | 1 |
| RDCNTGCVPCGDRCCCVPHSCIDSKCLGHATPHRAY | 1 |
| LCDSYISSELCEHPEETCFCPNHMCCPLSPYRQDQCMYWEACHTF | 1 |
| DCPVTGGPNPFHHCKIACMSTGTEEYCNCVYCKDCVNSNGEKPAC | 1 |
| SCDSEFSSEFCEQPEERICSCSTHVCCHLSSSKGDQCMTWNRCLSAQTGN | 1 |
| SCDSEFSSEFCEQPEERICSCSTHVCCHLSSRKRDQCMTWNRCLSAQTGN | 1 |
| SRCFPPGIYCTPYLPCCWGICCGTCRNVCHLRI | 0 |
| WPRLTDSDCELGRNMHITCKQLDQCGVIEKKDGQLTCKLRCKCKPGKRCLRKENIDWSDITTRIYHCPWP | 1 |
| SCDSEFSSEFCERPEESCSCLTHSCCHWSRRDQCMTPQRCISAQKGN | 1 |
| DCIPEGSHCGISSDCCKSTCCMETCEDPCRYTW | 1 |
| CISEESPCSSSGSCCQRSCCYGNCRFPCLQP | 1 |
| CAGNEEACTVASDCCGSFNCCNGYCDAPCRQP | 1 |
| TRSFADLPDDWGMCSDIGEGCGQDYDCCGDMCCDGQICAMTFMACMF | 0 |
| TCSNKGEQCEDDSDCCWHLCCVDNKCAKLILLCNL | 1 |
| GTCSGRGQECKHDSDCCGHLCCAGIKCHFTYFPC | 1 |
| RRCFPPGTFCSRYLPCCSGRCCSGWCTRRCFPRF | 1 |
| QFDCVDGGEQCRVNSNCCSYLCCPKSIGKRCAIQRSVGCDDFRI | 1 |
| SCDSEFSSEFCEQPEERICSCSTHVCCHLSSSKRDQCMTWNRCLSAQTGN | 1 |
| MKLTCVVIVAVLLLTACQLITANDSRGTQKHRALRSDTKLSMSTRCKSKGAKCSKLMYDCCSGSCSGTVGRCG | 1 |
| SRCFPPGIYCTPYLPCCWGICCGTCRNVCHLRF | 0 |
| RRCFPLGTFCSRYLPCCSGMCCSGWCTRRCAPRF | 1 |
| RRCFPPGTFCSRYLPCCSGRCCSGWCTRRCFPRF | 1 |
| CFPPGIYCTPYLPCCWGICCGTCRNVCHLRIX | 0 |
| CRSGKTCPRVGPDVCCERSDCFCKLVPARPFWRYRCICL | 1 |
| CRSGKTCPRVGPDECCERSDCFCKMVPARPYWRNKCICL | 1 |
| WPRLYDSDCVRGRNMHITCFKDQTCGLTVKRNGRLNCSLTCSCRRGESCLHGEYIDWDSRGLKVHICPKPWF | 1 |
| QCTPKDAPCDDNNQCCSGLECKCFNMPDCQSGSTCRV | 1 |
| MKLTCVLIVAVLFLTACQLTTAASYARSERQHPDLGSSDQNSKLTKRCLASGETCWRDTSCCSFSCTNNVCF | 1 |
| MKLTCVLIVAVLFLTACQLTTAASYARSEREHPDLGSSDQNSKLTKRCLASGETCWRDTSCCSFSCTNNVCF | 1 |
| MKLTCVLIVAVLFLTACQLTTAASYARSERQHPDLGSSDQNSKLTKRCLGSGETCWLDSSCCSFSCTNNVCF | 0 |
| MMKLTCVLIIAVLFLTACQLTTAETRDEYRAVRSSDEVRNSRSCSGSGYGCKNTPCCAGLTCRGPRQGPICL | 1 |
| MMKLTCVLIIAVLFLTACQLTTAETRDEYRAVRSSDEVRNSRSTRDCSGSGYGCKNTPCCDGLTCRGPHQGPICL | 1 |
| MMKLTCVLIIAVLFLTACQLTTAETRDEYRAVRSSDEVQNSRSTDDCSTAGCKNVPCCEGLVCTGPSQGPVCQPLA | 0 |
| MKLTCVVIVAVLLLTACQLITADDSRGTQKHRALRSDTKLSMSTRCKSKGAKCSKLMYDCCSGSCSGTVGRCG | 1 |
| MMTRVFFAMFFLMALTEGWPRLYDSDCVRGRNMHITCFKDQTCGLTVKRNGRLNCSLTCSCRRGESCLHGEYIDWDSRGLKVHICPKPWF | 1 |
| DVCDSLVEGRCIHNGCYCDRSAPHGNCCHTDGCTVAFWCPGTKWD | 1 |
| MKLTCVVIVAMLLLTACQLITANDSRGTQKHRALRSDTKLSMSTRCKSTGASCRRTSYDCCTGSCRSGRCG | 1 |
| LCDSYISSELCEHPEETCFCPNHMCCPLSPYRQDQCMYWEACHIF | 0 |
| RGVCSTPEGSCVHNGCICQNAPCCHPSGCNWANVCPGYLWDKN | 1 |
| RSCASNCVSCGSGCCCSPNSCVDSTCTHHSTPH | 1 |
| QCIPQNVSCEEDDECCSNLECKCTSAPDCNFPKCRA | 1 |
| CFPPGIYCTPYLPCCWGICCGTCRNVCHLRIGKRATFQE | 0 |
| SRCFPPGIYCTPYLPCCWGICCDTCRNVCHLRF | 0 |
| CSSGKTCGSVEPVLCCARSDCYCRLIQTRSYWVPICVCP | 0 |
| LCDSYISSELCEHPEETCFCPNHMCCPLSPYRQDQCMYWEACHIF | 0 |
| SRCFPPGIYCTPYLPCCWGICCDTCRNVCHLRI | 0 |
| CFPPGVYCTRHLPCCRGRCCSGWCRPRCFPRFX | 0 |
| GVCSTPEGSCVHNGCICQNAPCCHPSGCNWVNVCPGFLWDKN | 0 |
| MSTLKMMLLILLLLLPLATFDSDGQAIPGGGIPSAVNSRVGRLLGGDEKSGRSLEKRCSSGKTCGSVEPVLCCARSDCYCRLIQTRSYWVPICVCP | 0 |
| CRLEGSSCRRSYQCCHKSCCIRECKFPCRWVX | 0 |
| MSTLKMMLLILLLLLPMATFDSDGQAIPGGGIPSAVNSRVGGDEKSGRSLEKRCRSGKTCPRVGPDVCCERSDCFCKLVPARPFWRYRCICL | 0 |
| MSTLKMMLLILLLLLPMATFDSDGQAIPGGGIPSAVNSRVRGDEKSGRSLEKRCRSGKTCPRVGPDECCERSDCFCKMVPARPYWRNKCICL | 0 |
| MEKLTILILVATVLLAIQVLGQGEGEKPPKEWVQQYAAKRLWALMKGPRQCTPKDAPCDDNNQCCSGLECKCFNMPDCQSGSTCRV | 0 |
| CLRDGQSCGYDSDCCRYSCCWGYCDLTCLIIX | 0 |
| DCTTCAGEECCGRCTCPWGDNCSCIEWX | 0 |
| LCDSYISSELCEHPEETCFCPNHMCCPLSPYRQDQCMYWEACLIF | 0 |
| SCDSEFSSEFCEQPEERICSCSTHVCCHLSSRQKGSQCTTWNRCLSAQTGN | 1 |
| RDCGEGCGTCGGGCCCIPDSCVDSQCQSLRGA | 1 |
| MKLTCVVIVAVLLLTACQLITADDSRGTQKHRALRSDTKLSMSTRCKGTGKPCSRIAYNCCTGSCRSGKCG | 1 |
| MKLTCVVIVAVLLLTACQLITADDSRGTQRHRALRSDTKLSMSTRCKGKGASCRRTSYDCCTGSCRSGKCG | 1 |
| MKLTCVVIVAVLLLTACQLITTDDSRGTQKHLALRSDTKLSMSTRCKGKGAPCTRLMYDCCHGSCSSSKGRCG | 1 |
| MKLTCVVIVAVLLLTACQLITALHSRGTQKHRALGSTTKLTLSTRCLSPGSSCSPTSYNCCRSCNPYSRKCRG | 1 |
| ALGNCVPVPGQCIGNGCFCDREAPHGNCCDTDGCTSLFWCPGSKA | 1 |
| MRVYLLLTVTLLLTSFTGADAGPMPANGHFVRRDCQWGCEGCHNRAGCCCGDQVCVDNNRCEP | 1 |
| MRVYLLLTVTLLLTSFTGADAGPMPANGHFVRRDCQWGCEGCRNRAGCCCGDQVCVDNNRCE | 1 |
| MKLTCVIIVVALFLTACHAKDKQEHPAVRGSDDMQDSEDLKLAKKCTVDSDFCDPDNHDCCSGRCIDEGGSGVCAIVPVLN | 0 |
| MKLTCVLIIAVLFLTACQLTTGETYSRGEQKDHALRSTDKNSKLTRQCSPNGGSCSRHYHCCSLWCNKDSGVCVATSYP | 1 |
| MKLTCVLIIAVLFLTACQLTTGEQKDHAQRSADRNSKLTRQCTPVGGSCSRHYHCCSLYCNKNIGQCLATSYP | 1 |
| MKLTCVLIIAVLFLTACQLTTGEQKDHALRSTDKNSKLTRQCTPVGGYCSRHYHCCSNHCIKSIGRCVAH | 1 |
| MKLTCVLIIAVLFLTACQLTTGETYSRGEQKDHALRSTDKNSKLTRQCTPVGGYCFDHHHCCSNHCIKSIGRCVAH | 1 |
| MKLTCVLIIAVLFLTACQLTTGEQKDHALRSTDKNSKLTRQCTPVGGYCSRHHHCCSNHCIKSIGRCVAH | 1 |
| MKLTCVLIVALLFLTACQLTTADDSRDKQEDPLVRSHRKMQKSEDPKMAERCSNFGSDCIPATHDCCSGECFGFEDMGLCT | 1 |
| MKLTCVLIVAVLFLTACQLIAADDSRDLKRFSRRKMRDGMLNTKNTEEGCLPPLSLCTMADDECCHDCILFLCLVSP | 0 |
| MKLTCVLIVAVLFLTACQLIAADDSRDLKRFSRRKMRDGMLNTKNTKRQCLPPLSLCTMDDDECCDDCILFLCLVTS | 0 |
| MKLTCVLIVAVLILTACQLIAADDSRDLKRFSRRKMRDGMLNTKNMKRQCLPPLSLCTMDDDECCDDCXLFLCLVTS | 0 |
| MKLTCVLIVAVLFLTACQLIAADDSRDLQKFPRRKMRDGMLNTKNTKRQCLPPLHWCNMVDDECCHFCVLLACV | 0 |
| MKLTCMVIVTMLFLMACQLITADYSRERREYSDVRSSDKIQDSEDSKLTKRCTEEGEACEPEDHDCCSERCHETDHVCSPSVEVFELSRRKE | 1 |
| MKLTCVLIIAVLFLTACQLITADYSRDKQEYRAVRLRDAMRYSRVRRQCADLGEECHTRFCCPGLRCEDLQVPTCLMA | 0 |
| MKTVAVFLVVALAVAYGQFFCPSSEDESLNCIETMGTTATCMKSNRGETYSYACGYCGKKKETCFGDKKPVTDYQCQTRKIPNPCGGAA | 1 |
| MKLCLTFCQLITADLSSDEYRAVKSIDEMHDFKGSRATPECSRAGCKNVPCCSGLKCTGPQNGPVCQPE | 1 |
| MKLTCMVIIAVLFLTACQLITADLSSDEYHAVRSIDEMHDFKGSRATPECSRAGCKNVPCCSGLKCTGPQNGPVCQPE | 0 |
| MNLTCVLIIAVLFLTACQLLTADDSRNNQKYRAVRMRDALKNLKDSRGCSARNQRCPPQCCMGLVCGREYPPRCV | 1 |
| MKLTCVVIVAVLLLTACQLITALDSKGMEKRRALRSTTKLSKLRQVSWWCGKPEATCGKLYLKCCSGRCNKANWKCLR | 1 |
| MKLTCVLIIAVLFLIVCQLNTADDSRDKQEYRAVRLRDAIRNSRGSRSCGNLGESCSAHRCCPGLMCMGEASICIPY | 0 |
| MKLTCVLIIAVLFLMACQLITANDPRDNQKYRAVMMKDALKNFKDSRSCSGRDSRCPPVCCMGLMCSRGKCVSIYGEK | 0 |
| MKLTCALIVAMLFLTACQLTTTDDSRGRQKYPTERLRVKMRNPKLSKLTKTCDPPGDSCSRWYNHCCSKLCTSRNSGPTCSRP | 1 |
| MKLTCVVIVAVLLLTACQLITADDSRGTQRHRALRSDTKLSMSTRCKGKGASCSRTMYNCCSGSCNRGKCG | 1 |
| RDCGEGCGTCGGGCCCIPDSCVDSQCQSHVGPN | 1 |
| RDCGEGCGTCGGGCCCIPDSCVDSQCQSRVRPN | 1 |
| RDCGEGCGTCGGRCCCMPNSCVDSQCQGRVGPN | 1 |
| MKLTCVLIVAVLFLTACQLIAADDSRDLKRFSRRNMRDGMLNTKNTKRQCLPPLSLCNMADDDCCNDCVLFLCSYY | 1 |
| MSGLGIMVLTLLLLVSMATSHRYAREKQATRRDAVNVRRRSRPKTKECERYCELEEKHCCCIRSNGPKCSRICIFKFWC | 1 |
| MKLTCVVIVAVLLLTACQLITADDSRGTQKHRSLRSTTKVSKSTSCMKAGSYCRSTTRTCCGYCAYFGKFCIDFPSN | 0 |
| MKLTCMLIIAVLFLTACQLSTNASYARSKQKHRVLRSTDKNSKLTQRCNEAQEHCTQNPDCCSESCNKFVGRCLSD | 1 |
| MKLTCVLIIAMLFLIVCQLNTADDSTDKQEYRAVKLRDAMRNFKGSKRNCGEQGEGCATRPCCAGLSCVGSRPGGLCQYD | 1 |
| RTCSRRGHRCIRDSQCCGGMCCQGNRCFVAIRRCFHLPF | 1 |
| MEKLTIRILVAAVLLAIQVLIQSDGGNPLKGRVKQYAAKRLSALMRGPRQCTTRHQPCDEDEECCPYLECKCFTRPDCQSGYKCKPR | 1 |
| MMPVILPLLLSLAIRGGDGQAIQGDRDLIAKLFKRYQEHGLSVKRACHTCDDGTECCDSRCSCPWNTCTCIPWGK | 0 |
| MKLSVTFLLILVILPSVTGEKSSERTLSGAALRDKWGTCSLLGKGCRHHSDCCWDLCCTGKTCVMTVLPCHFFPL | 0 |
| MKLTCVVIVAVLLLTACQLLTADDSRGTQKHRALRSDTKLSMSTRCKGKGASCSRTMYNCCTGSCNRGKCG | 1 |
| MKLTCVVIVAVLLLTACQLLTADDSRGTQKHRSLRSTTKVSKATDCIEAGNYCGPTVMKICCGFCSPFSKICMNYPQN | 0 |
| QFFCPSSEDESLNCIETMGTTATCMKSNRGETYSYACGYCGKKKETCFGDKKPVTDYQCQTRKIPNPCGGAA | 1 |
| DVCDSLVEGRCIHNGCQCDRSAPHGNCCDTDGCTSLWWCPKTKWD | 1 |
| GPSSCKADEEPCEYHADCCNCCLSGICAPSTNWILPGCSTSSFFKI | 0 |
| GCKKDRKPCSYHADCCNCCLSGICAPSTNWILPGCSTSSFFKI | 0 |
| GHVSCGKDGRACDYHADCCNCCLGGICKPSTSWIGCSTNVFLTR | 0 |
| CHHEGLPCTSGDGCCGMECCGGVCSSHCGNX | 0 |
| CHHEGLPCTSGDGCCGMECCGGVCSSHCGNGRRRQVPLKSFGQR | 1 |
| DDCRPEAAYCEYNEQCCIDKCCQASCSDACRTP | 1 |
| SRPKTKECERYCELEEKHCCCIRSNGPKCSRICIFKFWC | 1 |
| MKLTCVVIVAVLLLTACQLITALHSRGTQKHRALGVTTKLTLSTRCLSPGSSCSPTSYNCCRSCNPYSRKCRG | 1 |
| MKLSVTFLLILVILPSVTGEKSSERTLSGAALRDKWGTCSLLGKGCRHHSDCCWDLCCTGKTCVMTVLPCLFLSLIVRWT | 0 |
| MEKLTVLILVATVLLTIQVLAQSDRDKHLKRRPKQYATKRLSALMRGTRDCTPQNVRCEEKSECCPNLECKCTSAPDCNFYKCRT | 1 |
| MEFRRLVTVALLLALVLSIDAEPMQGMEAEGHFVRRDCGEGCGTCGGGCCCIPDSCVDSQCQSLRGA | 0 |
| MEFRRLVTVALLLALVLSIDAEPMQGMEAEGHFVRRDCGEGCGTCGGRCCCMPNSCVDSQCQGRVGPN | 0 |
| SVCDFGSCVHNGCYCEEHRPCCTPGSCSSWWPRCPGSMMDP | 1 |
| DCPTSCPTTCANGWECCKGYPCVNKACSGCTHX | 0 |
| ANTGGLCGMPPGVCYPNGCACGQDTPCCHPSGCNRYNYCGPLLE | 1 |
| MSGMGVLLLVLLLVMPLAAFHQDGEGEATRRSGGLKRDCPTSCPTTCANGWECCKGYPCVRQHCSGCNHGK | 0 |
| DVCDSLVGGNCIHNGCWCDQEAPHGNCCDTDGCTAAWWCPGTKWD | 0 |
| GVCSTPEGSCVHNGCICQNAPCCHPSGCNWANVCPGFLWDKN | 0 |
| MKLTCMMIVAVLFLTAWTLVMADDSNNGLANHFSKSRDEMEDPEASKLEKRACRKKWEYCIVPIIGFIYCCPGLICGPFVCVLIGMSSTPICATPGLIFDRRVPFTGYELL | 0 |
| DCPTSCPTTCANGWECCKGYPCVRQHCSGCNHX | 0 |
| GVCSTPEGSCVHNGCICQNAPCCHPSGCNWVNVCPGFLWDRS | 0 |
| DKWGTCSLLGKGCRHHSDCCWDLCCTGKTCVMTVLPCHFFPL | 1 |
| RDCQWGCEGCHNRAGCCCGDQVCVDNNRCEP | 1 |
| GVCSTPEGSCVHNGCICQNAPCCHASGCNWANVCPGFLWDKN | 0 |
| MKLTCVIVAVLFLTACQLTTAGDSRDKEGYRAVRSILSMQDGIDSRECRRRGQGCTQSTPCCDGLRCDGQRQGGMCVDS | 1 |
| MEFRRLVTVALLLALVLSIDAEPMQGMEAEGHFVRRDCGEGCGTCGGGCCCIPDSCVDSQCQSHVGPN | 0 |
| RDCQWGCEGCRNRAGCCCGDQVCVDNNRCE | 1 |
| MEFRRLVTVALLLALVLSIDAEPMQGMEAEGHFVRRDCGEGCGTCGGGCCCIPDSCVDSQCQSRVRPN | 0 |
| DKWGTCSLLGKGCRHHSDCCWDLCCTGKTCVMTVLPCLFLSLIVRWT | 0 |
| WCSERVSDETCVMMCRCLNHECCPLPPPSQNRCMPSDHCDFMSGRT | 1 |
| MKLLCVLIVAMLPLMACHLIIADDSSEKQGYLTVRTSDKIQESEDSKLTERCFNDGDDCEIGDDCCSGSCIFDEYFSYCDASDPYYDDYDEYYYE | 0 |
| MKLTCMVIVAVLLLTACQLITAEDSRGTQKHRTLRSTARRSKSELTTRCRPSGSPCGVTSICCGRCSRGKCT | 1 |
| MKLTCMVIVAVLLLTACQLNTADGSRDKQEDRLVKLFNKRWNSPDSRLTRKCQPNGYYCDFEFTPKCCLKCDYNRKYCQPY | 1 |
| DVCDSLVEGRCIHNGCWCDEEAPHGNCCDTAGCTAWWWCPGTKWD | 0 |
| DVCDSLVGGHCIHNGCWCDQDAPHGNCCDTGGCVWWWCPGTKWD | 0 |
| DVCDSLVDGRCIHNGCFCERDAPNGNCCDTDGCTARWWCPGTKWD | 1 |
| DVCDSLVDGRCIHNGCYCERDAPNGNCCNTDGCTARWWCPGTKWD | 1 |
| CDSDGTSCTSNMECCGYGCCSGTCQTPCRFGP | 1 |
| CSDEGASCEKKSDCCFLSCCWSVCDRPCRLVP | 1 |
| MMMRVFIAMFFLLALVEAGWPRLYDKNCKKNILRTYCSNKICGEATKNTNGELQCTMYCRCANGCFRGQYIDWPNQQTNLLFC | 0 |
| MKLTCVVIVVVLFLTACQLITADDSRRTQKHRALRSTTKLSLSTRCRIPNQKCFQHLDDCCSRKCNRFNKCV | 1 |
| MKLTCVLIVAVLILTACQFTAADDMEYPKWLRGLSTDXSERGCWLCLGPNACCRGSVCHDYCPR | 0 |
| CRTEGMSCEENQQCCWRSCCRGECEAPCRFGPX | 0 |
| MFRVTSVLLVIVLLNLVVLTNACHMDCSKMTCCSGICCFYCGRPMCPGTRRALLQRLVGHQR | 0 |
| MKLALTFLLILMILPLMTGEKTSDDLELRGVESLRAIFRDRRCSDNIGATCSDRFDCCGSMCCIGGQCVVTFAECS | 0 |
| MFRVTSVGFILLVIVLLNLVVLTDAYYHQCTLTEEGNPCTYDWQCCHMICCYNSCTFGCKRRRRFRLLDKR | 0 |
| DLITNSYTRGKPRHVTSWRNLRTRDVCKKSPGKCIHNGCFCEQDKPQGNCCDSGGCTVKWWCPGTKGD | 1 |
| MEKLTILVLVATVLLAIQVLVQSDGEKPLKRRVKQYAAKRLSALMRGPRQCTPRNQRCEGDAECCPNLVCKCFTRPDCQSGYKCDTS | 1 |
| MVIMKLALTFLLILLILPLMNGEKTSGDHELRGVDSLKAILRDRRCSDDTGATCSNRFDCCESMCCIGGHCVISTVGCP | 0 |
| MKLALTFLLILMILPLMTGEKTSGDHELRGVDSLKAILRDRRCSDDTGATCSDNSDCCGDMCCLSNTCVVTIVACS | 0 |
| DLITNSYTRGKPRHVTSWRNLRTDVCKKSPGKCIHNGCFCEQDKPQGNCCDSGGCTVKWWCPGTKGD | 1 |
| DLITNSYTRGKPRHVTSWPKLRALGNCVPVPGQCIGNGCFCDREAPHGNCCDTDGCTSLFWCPGSKAG | 0 |
| MKLTCVVIVAVLLLTACQLITADDSRGTQKHRALRSTTKLSTSTRCKGKGAKCSRLMYDCCTGSCRSGKCG | 1 |
| DLITNSYTRGKPRHVTSWRNLKTRDVCDSLVEGRCIHNGCQCDRSAPHGNCCDTDGCTSLWWCPKTKWD | 1 |
| MRLTTMHSVILMLLLVFAFDNVDGDEPGQTARDVDNRNFMSILRSEGKPVHFLRAIKKRDCTGQACTTGDNCPSECVCNEHHFCTGKCCYFLHA | 1 |
| DLITNSYTRGKPRHVTSWRNLKTRDVCDSLVEGRCIHNGCYCDRSAPHGNCCHTDGCTVAFWCPGTKWD | 1 |
| MKLTCVVIVAVLLLTACQLITADDSRGTQKHRALRSTTKLSMSTRCKGKGAKCSRLMYDCCTGSCRSGKCG | 1 |
| DVCDSLVEGRCIHNGCWCDEEAPHGNCCDTAGCTAWWWCPGTKFD | 0 |
| DVCDSLVGGHCIHNGCWCDQDAPHGNCCDTDGCTAAWWCPGTKWD | 0 |
| DVCDSLVGGHCIHNGCWCDQEAPHGNCCDTDGCTAAWWCPGTKWD | 0 |
| FCTEIGKDCGTSWECCEDCCIHGTCSHESNCANFKL | 0 |
| MKLMCVLIVSVLVLTACQLSTADDTRDKQKDRLVRLFRKKRDSSDSGLLPRTCVMFGSMCDKEEHSICCYECDYKKGICV | 0 |
| MKLTCVVIVAVLLLTACQLIIAEDSRGTQLHRALRKATKLSVSTRTCVMFGSMCDKEEHSICCYECDYKKGICV | 0 |
| MKLTCVLIVAVLFLTACQLATAENSREEQGYSAVRSSDQIQDSDLKLTKSCTDDFEPCEAGFENCCSKSCFEFEDVYVCGVSIDYYDSR | 0 |
| MKLICVFIVAVLLLTACQLNAADDSRDTQKHRALRSTTKLSMSKKDSCVPDGDSCLFSRIPCCGTCSSRSKSCVG | 1 |
| ECTPMHRACEEDEECCPNLECKCSSNPDCQSGYKCRP | 1 |
| MKLTCVLIVAVLVLTACQFTAAIDSRDGQDNPALRSTGGMQRKSQTQRLSKKCIPDHHGCGLLHHSRYCCNGTCFFVCIP | 0 |
| MMSKLGVLLIICLLLCPLTAVPQDGDQPADQPAERMQDDISSEHHPFFDPVKRCCKYGWTCWLGCSPCGC | 0 |
| MTLTFLLVVALCMLTTCHTENYRDSQKVSPVRSIGKTQFARSLRLSERYCVPKSGLCTIFQPGKCCSGWCLIYRCT | 0 |
| MKLTCVPIVAMLFLMACQLITADYSREKHGYSAEKSSDKIQDSFYSKLTKRCTDEGGDCDPGNHNCCRGSCLVLQHKAVCGILYTMVSR | 1 |
| MKLTCVPIVAMLFLMACQLITADYSREKHGYSAEKSSDKIQDSFYSKLTKGCTDEGGDCDPGNHNCCRGSCLVLQHKAVCGILYTMVSR | 0 |
| CRVENKCPHTVCCDRSRCSCKLIRTRPLMYHVCVC | 0 |
| CGGTGHSCNEPAGELCCRRLKCVNSRCCPTTDGC | 1 |
| MATSLLSPLLVAMLGFLLHVHVARAGLEHTCTLETRMQGAHPQGICGSKLPDIVHTVCQVMGRGYAGGQRQLRKRTSMIDSDDMEAEGGSRGGFLMSKRRALSYLQKETNPLVMAGYERRGIQKRHGEQGITCECCYNHCSFRELVQYCN | 1 |
| MKLTCVPIVAMLFLMACQLITADYSREKHGYSAEKSSDKIQDSFYSKLTKRCTDEGGDCDPGNHNCCRGSCLVLQHKAICGIVYTMVSR | 0 |
| CKGQSCSSCSTKEFCLSKGSRLMYDCCTGSCCGVKTAGVT | 1 |
| QCTPRNQRCEGDAECCPNLVCKCFTRPDCQSGYKCDTS | 1 |
| MKLALTFLLILMILPLTTGGKKSDNQALKRLGARKFNENLSELNSACDDAWETCAWSRTCCSRNCCRGICVSRYYECP | 1 |
| MCLSLGQRCGRHSNCCGYLCCFYDKCVVTAIGCGHY | 1 |
| MCLSLGQRCERHSNCCGYLCCFYDKCVVTAIGCGHY | 1 |
| MKMSVTFLLILMILPLFTGEWQSGSRLSALKKRLLEKRLLQKRFCTEIGKDCGTSWECCEDCCIHGTCSHESNCANFKLR | 1 |
| NWSWCSGSGEGCDYHSECCGERCCIESMCIGDGVACWP | 0 |
| MKLTCVVIVAVLFLTACQLITADDSRSTQRHRALRSTTKLSMSTRCKPPGSKCSPSMRDCCTTCISYTKRCRKYYN | 0 |
| WCSGSGEGCDYHSECCGERCCIESMCIGDGVACWP | 1 |
| RDCQTGCVGCHNPAGCCCGGQVCVDNKRCEGGFY | 1 |
| KFNENLSELNSACDDAWETCAWSRTCCSRNCCRGICVSRYYECP | 1 |
| MKLTCVLIVVVLFLTACQLIPADYSRDTPGYPAWKLKTKMQNSRRWKLAKRCKGKGAGCDYSHECCSRQCTGRIFQTCN | 1 |
| CQAYGESCSAVVRCCDPNAVCCQYPEDAVCVTRGYCRPPATVLT | 0 |
| GAVPCGKDGRQCRNHADCCNCCPIGTCAPSTNWILPGCSTGQFMTR | 1 |
| TLSGATLTGGRGMCSLLGQRCGDHSDCCWDMCCASEMCVVTFLPC | 0 |
| GCKKDRKPCSYQADCCNCCPIGTCAPSTNWILPGCSTGPFMAR | 1 |
| RCSDDTGATCSDNSDCCGDMCCLSNTCVVTIVACS | 0 |
| CTSEGYSCSSDSNCCKNVCCWNVCESHCRHPGKRTRLQGFFKH | 1 |
| RRCFPPGTFCSRYLPCCSGRCCSGWCTRRCFPRF | 1 |
| CLRDGQSCGYDSDCCRYSCCWGYCDLTCLIN | 1 |
| MQKATVLLLAILLLLPLSTAQDAEGSQEDAAQREVDIATRCGGTGHSCNEPAGELCCRRLKCVNSRCCPTTDGC | 1 |
| MEKLTILVLVATVLLAIQVLVQSDREKPLKGRVKQYAAKRLPALMRGPRQCTPKNQICEEDGECCPNLECKCFTRPDCQPGYKCRP | 0 |
| MTFYLLLTVTLLLTVSTGGDAGPRRANGLQKHFVRRDCRTGCVGCFNPNGCCCGGQVCVNGNHCEYKSSWF | 1 |
| MEFRRLATVALLLALVLSIDAEPMQGMEAEGHFVRTDCDEGCMKCGSGCCCSPNSCIDSQCRGQVPPQ | 0 |
| DCTGQACTTGDNCPSECVCNEHHFCTGKCCYFLHA | 1 |
| MEKLTILILVATVLLAIQVLVQSDREKPLKGRVKQYAAKRLSALLRGPRECTPMHRACEEDEECCPNLECKCSSNPDCQSGYKCRP | 0 |
| VLIIAVLFLTACQLTTAETSSRGEQKHCALRSTDKNSRMTKRCTPRHGVCFYSYFCCSKACNPSSKRCH | 1 |
| MRFFFLLLTVALFLTSITGDDAERMLGMKEGGYVREDCGSDCMPCGGECCCEPNSCIDGTCHHESSPN | 0 |
| MEKLTILILVATVLLAIQVLVQSDGENPVKGRVKHYAAKRFSALFRGPRECTTKHRRCEKDEECCPNLECKCLTSPDCQSGYKCKP | 0 |
| MKLTCVLIIAVLFLTACQLATAKTYSKGRQKHRALRSTDKNIKLTRRCTPDDGACAEPVQCCSTFCNPVTNMCIDWLGIGLSRSVL | 0 |
| MKLTCVLIITVLFLTACQLTTAVTYSRGEHKHRALMSTGTNYRLPKTCRSSGRYCRSPYDCCRRYCRRITDACV | 1 |
| MKLTCVVIVAVLLLTACQLLTADDSRGTQKHRALRSDTKLSMSTRCKGKGAPCRKTMYDCCSGSCGRRGKCG | 1 |
| MKLTCVLIVIMLFLTVCPLITADHSRDKQEHPAMRLKDRIRYLRRGKLTRDCKHQNDSCAEEGEECCSDLRCMTSGAGAICVT | 0 |
| MEKLTILILVATVLLAIQVLVQSDREKPLKGRVKQYAAKRLSALLRGPRECTPMHRACEEDEECCPNLECKCSNNPDCQSGYKCRP | 0 |
| MKRLTILILVATALLSTQVMVGGDGEKPLMRKNAANRLLAPMRGKRCKTEDYLCQKDEDCCSGFDCRCTVNANCSPPHIRCKP | 0 |
| MKLTCMVIVAVLLLTACQLITADDSRGTQKHRTLRSKTKLSMSTRCKAAGKPCSRIAYNCCTGSCRSGKCG | 1 |
| MKLTCMVIVAVLLLTACQLITADDSRGTQKHRSLRSTTKVSKATDCIEAGNYCGPTVMKICCGFCSPYSKICMNYPKN | 0 |
| MKLTCVVIVAVLLLTACQLITADDSRGTQKHRSLRSTTKVSKATDCIEAGNYCGPTVMKICCGFCSPYSKICMNYPKN | 0 |
| MKLTCVVIIAVLLLTACQLITADDSRGVQKHRSLRSTTKVSKSTSCMEAGSYCGSTTRICCGYCAYFGKKCIDYPSN | 0 |
| MKLTCVMIVAVLLLTACQLITADDSRGTQKHRSLRSTTKVSKSTSCMEAGSYCGSTTRICCGYCAYFGKKCIDYPSN | 0 |
| MKLTCVVIVAVLLLTACQLITAEDSRGAQKHRTLRSTARRSKSELTTRCRPSGSPCGVTSICCGRCYRGKCT | 1 |
| MKLTCVMIVAVLLLTACQLITAEDSRGTQKHRTLRSTARRSKSESTTRCRSSGSPCGVTSICCGRCYRGKCT | 1 |
| MKLTCALIVAMLLLTACQLITTDDFRGRQQYRTARSRTKMQNYKIFRLTKRCDAPNAPCEKFDNDCCDACMLREKQQPICAV | 0 |
| ECTPMHRACEEDEECCPNLECKCSNNPDCQSGYKCRP | 1 |
| RCSDDTGATCSNRFDCCESMCCIGGHCVISTVGCP | 0 |
| MKLTCVVIVAVLLLTACQLITADDSRGTQEHRALRSDTKLSMLTLRCESYGKPCGIYNDCCNACDPAKKTCT | 0 |
| CVPPSRYCTRHRPCCRGTCCSGLCRPMCNLWY | 1 |
| TCSNKGQQCGDDSDCCWHLCCVNNKCAHLILLCNL | 1 |
| QCTPKNQICEEDGECCPNLECKCFTRPDCQPGYKCRP | 1 |
| GHVPCGKDGRKCGYHADCCNCCLSGICKPSTSWTGCSTSTFD | 1 |
| RDCRTGCVGCFNPNGCCCGGQVCVNGNHCEYKSSWF | 1 |
| TDCDEGCMKCGSGCCCSPNSCIDSQCRGQVPPQ | 1 |
| QCTQQGYGCDETEECCSNLSCKCSGSPLCTSSYCRP | 1 |
| DVCDSLVGGRCIHNGCYCERDAPNGNCCNTDGCTARWWCPGTKWD | 1 |
| DVCDSLVGGRCIHNGCWCERSAPHGNCCNTSGCTARWWCPGTKFD | 1 |
| QCTPQNVKCEEDDECCSNLECKCSTVPDCNFPKCRP | 1 |
| MKLTCVMIVAVLLLTVCKVVTSDQLKKLRRECYLEPGDSCFHHDGRGACCEGTCFFGVACVPW | 0 |
| MKLTCVLIVAVLILTACQVIAADEAEATNRAIKRGWFGEESSCWWCTGFNKCCEAAAVCQSVNSACP | 0 |
| MKLTCVLIVSVLILTACQFTAAVDCHSTGYLCFWWHECCSNFCIPLQQRCF | 0 |
| MKLTCVLIIAVLFLTACQLATAKTYSTGRQKHRALRSTDKNIKLSRRCNDPGGSCTRHYHCCQLYCNKQESVCLENEPAF | 1 |
| MKLTCVLIVAVLILTACQVIAADSSCWFCSTGFNKCCESTGDCMTYPSEYNASCPEA | 0 |
| CFPPGVYCTRHLPCCRGRCCSGWCRPRCFPRY | 1 |
| LCDSYISSELCEHPEETCFCPNHMCCPLSPYRQDQCMYWEACHIF | 0 |
| CHMDCSKMTCCSGICCFYCGRPMCPGT | 0 |
| CSDNIGATCSDRFDCCGSMCCIGGQCVVTFAECS | 0 |
| YYHQCTLTEEGNPCTYDWQCCHMICCYNSCTFGCKRRRRFRLLD | 1 |
| CTSEGYSCSSDSNCCKNVCCWNVCESHCGHH | 1 |
| SRCFPPGIYCTPYLPCCWGICCGTCRNDNSSLTFLQFCLPFFFF | 0 |
| CRLEGSSCRRSYQCCHKSCCIRECKFPCRWD | 1 |
| MKLTCVVIIAVLILTACQFTTADDCKPKNNLCLWSSECCSGICFPFAQRCT | 0 |
| MKLTCVVIVAVLLLTACQLITAEDSRGTQKHRTLRSTARRSKSELTTRCRPSGSPCGVTSICCGRCSRGKCT | 1 |
| MKLTCVVIVAVLLLTACQLITADDSRGTQKHRSLRSTTKVSKAADCIEAGNYCGPTVMKLCCGFCSPYSKICMNYPKN | 0 |
| NCKKNILRTYCSNKICGEATKNTNGELQCTMYCRCANGCFRGQYIDWPNQQTNLLFC | 1 |
| MKLTCVVIVAVLLLTACQLITADDSRGTQKHRALRSDTKLPMSTRCKLKGQSCRKTSYDCCSGSCGRSGKCG | 1 |
| CRVENLCPHTVCCDRSRCSCKLIRTRPLMYHVCVC | 0 |
| MTSVQSATCCCLLWLVLCVQLVTPDSPATAQLSRHLTARVPVGPALAYACSVMCAKGYDTVVCTCTRRRGVVSSSI | 0 |
| MKRTCALIVAVLFLTACQIIATNDSRGRQKYPTEKLRAKMRNSELFKLTKRCDPPGAGCSLWESDCCSHFCILRQSGPTCGD | 1 |
| MKLTCALIVAMLLLTACQLTTADASRGRQEYPTKRLRAKMLNSKFIKLIKRCAAPGASCSKYDNECCDACLLQYPNPPVC | 0 |
| MKLTCVSIVAVLFLAACQLITADDSREKQGYSAVRSSDKIQDSDDLELTKGCMEDGDVCELGNHNCCSGSCLGFEDEGICAVGVEYY | 0 |
| RVTCVLIIAVLFLTACQLITADYSRDKQENPVERSRIKMIKSWRPKLNKRCTNPGGYCVVPHHNECCSGQCDPSSIIGQC | 0 |
| MRVTCVLIIAVLFLTACQLITADYSRDKQENPVERSRIKMIKSWRPKLNKRCTNPGGYCVVPHHNECCSGQCDPSSIIGQC | 0 |
| CRAEGTYCENDSQCCLNECCWGGCGHPCRHPX | 0 |
| CLRGSVCMCFDARGEEVVVRQCDNHVRPFCHRPEYRVHVHVDGWPDSASVPLHVYGLCLPPSGSRYLQVHRGCHWTDMVVPSGGAHSCRLHD | 1 |
| DADDVPCSTNGCNGNHNCSTNCNCTFNMASRCLNCVC | 1 |
| MTMTCVLIIAVLFLTACQLITADYSRDKQENPVERSRIKMIKSWRPKLNKRCTNPGGYCVVPHHNECCSGQCDPSSIIGQC | 0 |
| QFFCPDSENDPLNCLETMASAATCMKSKSDGTYSYACGYCGKKKETCFGDKVPVTDYNCQRHKIVNHCGGPVV | 1 |
| CLLIVAMLFLTACQLATADDSRDKQEDHLLRSHREKQKSEDPKMAVRCSYFGADCLPDSHDCCSGECFGFPDMGLCT | 1 |
| MKLTCLLIVAMLFLTACQLATADDSRDKQEDHLLRSHREKQKSEDPKMAVRCSYFGADCLPDSHDCCSGECFGFPDMGLCT | 1 |
| NCQNGPNMHHTYRCRSRQRCAIIRKRNGQLTCELKCKCESVGDCLQGEVVDWDVRTVKTYTCP | 1 |
| MMFRLTSVLLVIVLLNLVVLTNACHMDCSKMICCSGICCFYCGLPSCDDTRRALLQRLLGHQR | 0 |
| ELARTTHCGGTGESCGRPEDPNCCGLQKCVSSKCCPTTDAC | 1 |
| CSTKMCGEDCCPSTSCECETVGSQSNEVGCSCPV | 1 |
| CSTKVCGEDCCSSSSCECETVGSQDNEVGCSCPVF | 1 |
| CSTKMCGDDCCPSTSCECDVVGSQGNEMGCSCPVLLL | 0 |
| GCKPPNSSCERDSECCSGECKCSNAAECAGFSPECV | 1 |
| GHVPCGKDGRKCGYHADCCNCCLSGICKPSTSWTGCSTSTFD | 1 |
| GHVPCGKDGRKCGYHADCCNCCLSGICKPSTSWTGCSTSTVQLTR | 1 |
| GHVSCGKDGRACDYHADCCNCCLGGICKPSTSWIGCSTNVFLTR | 0 |
| GHVPCGKDRRKCGYHADCCNCCLSGICKPSTSWTGCSTSTFLLTR | 1 |
| GHVPCGKDGRKCGYHADCCNCCLSGICKPSTSWTGCSTSTFLLTR | 1 |
| GHVPCGKDGRKCGYHADCCNCCLSGICKPSTSWTGCSTSTFN | 1 |
| MKLTCALIVAMLLLTACQLTTTDDSRGRQKYPTERLRAKMRNSKLFKLTKRCDPPGDSCSRVYNDCCSNYCILRQSGPTC | 1 |
| MKLTCVLIVAMLFLTACQLIAADDYRDLQEFPRRKMSDVILNTKDTEKRCLPGTATCNLYNNLCCNYCLIFWC | 0 |
| MKLTCVLIVAVLFLTACQLIAADDFRDLQKFPRRKMSDRMPNTKGVERDCLPPLTWCSMTDDECCNDCVLFLC | 0 |
| MKLTCVIIVVALFLTACHAKGKQEYPAVRGSDEMQDSEDLKLAKKCTVDSDFCDPGNHNCCSGKCIDEGGSGVCAIIP | 0 |
| RPKCCCVCGVVGRKCCSTWDKCHPVHLPCPSSX | 0 |
| ECTTKHRRCEKDEECCPNLECKCLTSPDCQSGYKCKP | 1 |
| MDARRVAAVLLVLFMARSLLKGSAKDARCLEPLDLGDSTCSEVRIRWYYNTGNEICQTFQYTGCGGNNNNFYDENSCKQCCEWEYSCSKRFARR | 1 |
| MEGRRFAAALILAICMLAPGASASRRDVLLSACKLPAKTGPCYSFVRKYYFDWDSVNCKTFIYGGCDGNSNNFCTYNECYSRCGPVNRR | 1 |
| MWVVLVCLLGLCLFTNADDRCTLQISTGTCPGYFPRWFYDPASGQCQSFIYSGCKGNANNFLSEEECHQACVSR | 1 |
| DVCDSLVDGRCIHNGCFCEESKPNGNCCDTDGCTAAWWCPGTKWD | 1 |
| DVCDSLVDGRCIHNGCFCEESKPNGNCCDTGGCTAAWWCPGTKWD | 1 |
| QCTPQNVQCEEDDECCSNLECKCSTVPDCNFPKCRP | 1 |
| DVCDSLVDGRCIHNGCFCEESKPNGNCCDTDGCTARWWCPGTKWD | 1 |
| MKLTCVLIAAVLLLAVCQLDSADATGYMRKNPSLRSPKRTRGCKSKGSFCWNGIECCGGNCFFACIY | 0 |
| MKLTCVLIAAVLLLAVCQLDSADATGYMRKNPSLRSPKRTRGCKSKGSFCWNGIECCGGNCFFACVY | 0 |
| MKLTCVLIAAVLPLAVCQFDSTDASAYMRKNPSLRSPKRTRDCKTKGSVCFASSECCIQDCWFVCLY | 0 |
| GHVPCGKDGRKCGYHTHCCNCCLSGICKPSTSLIGCSTSSFT | 0 |
| MKLMCVLIVAMLFLTVCQLITADDSRDKQEYPAERWRTKMQNSKRTTSTRKCKGPLVFCPENHECCSKFCDFIDIPLRYCSTP | 0 |
| MKLTCVLIVAMLFLTVCQLITPDDSRDKQEYPAERWRTKMQNSKRTTSTRKCKGPLVFCLENHECCSKFCDFSDIPLRYCSTP | 0 |
| MKLTCVLIVAMLFLTVCQLIAPDDSRDKQEYPAERWRTKMQNSKRTTSTRKCKGPLVFCLENHECCSKFCDFSDIPLRYCSTP | 0 |
| LVVLTNACYPEGEYCEYNYQCCESSCCVNYCRYPCRKRAQLQELLR | 1 |
| DVCDSLVGGRCIHNGCYCERSAPNGNCCDTAGCTVLWWCPGTKWD | 1 |
| MKLTCVLIVALLFLTACQLITADYSRDDYRAVMGLGRRNSKDSRDCQESGQGCTGSPPCCPGLSCSGTHAGGMCVG | 1 |
| MQKATVLILAILLLLPLSTAQDDEGSQENAAERELARTTHCGGTGESCGRPEDPNCCGLQKCVSSKCCPTTDAC | 1 |
| DVCDSLVGGRCIHNGCWCERSAPHGNCCNTSGCTARWWCPGTKWD | 1 |
| DVCDSLVGGRCIHNGCYCERDAPNGNCCDTDGCTARWWCPGTKWD | 1 |
| DVCDSLVGGHCIHNGCWCDQEAPHGNCCDTGGCVWWWCPGTKWD | 1 |
| DVCDSLVDGRCIHNGCWCDQEAPHGNCCDTDGCTAAWWCPGTKWD | 1 |
| HASRSATAWRDRHLSPKALLCGGVRASCSRHDDCCGSLCCFGTSTGCRVAVRPCW | 1 |
| MKLTCVVIVAVLFLTACQLNDADDSRYKHETRLVTLFRRRRESDSGLAPGTCVLFGSMCKAKTASICCYKCDLEEGIC | 0 |
| MKLTCVLIVAMLFLTVCQLITADDSRDKQEYPAERWRTKMQNSKRTTSTRKCKGPLVFCLENHECCSKFCDFIDIPLRYCSTP | 0 |
| GPSFCKANGKPCSYHADCCNCCLSGICAPSTNWILPGCSTSSFFKI | 0 |
| GAVPCGKDGRQCRNHADCCNCCPFGTCAPSTNRILPGCSTGMFLTR | 1 |
| GAVPCGKDGRQCRNHADCCNCCPIGTCAPSTNWILPGCSTGPFMTR | 1 |
| MRFYLLLTVALFLTSITGGDARLMQGMEATRPFGRRDCQRGCHGCSNVPNGCCCGNLVCQNEQRCVPKEG | 1 |
| MMSKLGVLLTICLLLFPITCLPLDEDQHADLPALRAQDFEPEHSPWFDPVRRCCSQDCSVCIPCCPN | 0 |
| CLEPLDLGDSTCSEVRIRWYYNTGNEICQTFQYTGCGGNNNNFYDENSCKQCCEWEYSCSKRFA | 1 |
| TRIGAVLKGHWCGYPGERGCRYHSQCCGDMCCYDRKCVVTAMPC | 1 |
| MMFRLTSVGSFLLVIVFLNLVVLTNACDLEGMFCMYDFECCLSECCMGICAFGCTKRAQRQKLLRSFGQR | 0 |
| ASICYGTGGRCTKDKHCCGWLCCGGPSVGCVVSVAPC | 1 |
| AWPCGGVRASCSRHDDCCGSLCCFGTSTGCRVAVRPCW | 0 |
| ALLCGGTHARCNRDNDCCGSLCCFGTCISAFVPC | 0 |
| MKLTCVAIVAVLLLTACQLITAEDSRGTQLHRALRKTTKLSVSTRCKGPGAKCLKTMYDCCKYSCSRGRC | 1 |
| MEKLTILILVATALLSTQVMVGGDGKKPLMGRIKGNAAKSLSAFLRGKGCKAPNSSCERDSECCSGDCQCSNAAECAGFSPECA | 0 |
| MEKLTILILVATALLSTQVMVGGDGKKPLMGRIKGNAAKSLSAFLRGKGCKPPNSSCERDSECCSGECKCSNAAECAGFSPECV | 0 |
| MRKHTQTKKKKKTTLGMVLLLLLVLLPLGNSDGDGDRQAMDRDHTASKERGAPILRLRRHMDHGRSINKRCSTKMCGDDCCPSTSCECDVVGSQGNEMGCSCPVLLLRK | 1 |
| CYPEGDYCEYHYQCCKGSCCFAYCRDPCRRV | 1 |
| MSTLGMVLLLLLVLLPLGNSDGDGDRQAMDRDHTASKERGAPILRLRRHMDHGRSINKRCSTKVCGEDCCSSSSCECETVGSQDNEVGCSCPVFGK | 1 |
| TCDSEFGETCEQEKVCSCSNHGCCEAASSAKTDQCMTQGACVAWLSDNVKRRVIQKQ | 1 |
| MKLTCVVIVAVLFLTACQLATADISGGMRKHRALRSTTKLSRSPFDCSSPGAFCGLVPCCDSCNVLGRCGSGLHV | 0 |
| MKLTCVLIIAVLFLTACQLVTADYSGDEQQYRAMRLIDAMRNFGDTRSCGRRGKPCPCCRGFRCTGSFCRKWQ | 1 |
| MVRVFIAMFFLWALTEGWPRLHDRNCQNGPNMHHTYRCRSRQRCAIIRKRNGQLTCELKCKCESVGDCLQGEVVDWDVRTVKTYTCP | 1 |
| MKVVAVFLVVALAVAYGQFFCPDSENDPLNCLETMASAATCMKSKSDGTYSYACGYCGKKKETCFGDKVPVTDYNCQRHKIVNHCGGPVV | 1 |
| TCDSEFGETCEQEKVCSCSNHGCCEAASSAKTDQCMTQGACMAWLSDNVKRRAIQKQ | 1 |
| SCKYGEWTEKCGQPGETCECPNHVCCDLKRDVCMPSKSCKALIHKIVF | 1 |
| SCNGRISSENCEQPGEQICSCPNHECCHFHPPKRDQCMTRGMCYMALAGMP | 1 |
| MMLELRVVLYILLVLLPPTTLQEKEEDDTSDALVNQLDEKIADGTILVRDLSRDADDVPCSTNGCNGNHNCSTNCNCTFNMASRCLNCVC | 0 |
| MKLTCVLIITVLFLTACQLTTAVTYSRGKQKHRALRSTDKTSWLTKPCSRLMEPCTQHPQCCSNTCSKFTTKCIS | 1 |
| SWGPACQTRMCGGICCGSDSCGCKGNECDCP | 1 |
| MHTLGRTSRLEALAPGASIKMARIFFFFFSSSGDLGAPIPHLQSQERRCLRGSVCMCFDARGEEVVVRQCDNHVRPFCHRPEYRVHVHVDGWPDSASVPLHVYGLCLPPSGSRYLQVHRGCHWTDMVVPSGGAHSCRLHD | 1 |
| MSTLGMVLLLLLVLLPLGNSDGDGDRQAMDRDRTASKERSAPRLRLRRHMDHGRSINKRCSTKMCGEDCCPSTSCECETVGSQSNEVGCSCPV | 1 |
| CKTEDYLCQKDEDCCSGFDCRCTVNANCSPPHIRCKP | 1 |
| CYPEGEYCEYNYQCCESSCCVNYCRYPCR | 1 |
| MEKLTILILVATVLLAIQVLVQSDGEKPLKGRVKQNAAKRLSALLRGPRECTPMHRACEEDEECCPNLECKCSNNPDCQSGYKCRP | 0 |
| GPRCWVGRVHCTYHKDCCPSVCCFKGRCKPQSWGCWSGPT | 1 |
| GAVPCGKDGRQCRNHADCCNCCPIGTCAPSTNWILPGCSTGQFMTR | 1 |
| GPSFCKADEKPCEYHSDCCNCCLSGICAPSTNWILPGCSTSSFFKI | 0 |
| GCKAPNSSCERDSECCSGDCQCSNAAECAGFSPECA | 1 |
| GPSFCKADEKPCEYHADCCNCCLSGICAPSTNWILPGCSTSSFFKI | 0 |
| GPSFCKANGKPCSYHADCCNCCLSGICKPSTNVILPGCSTSSFFRI | 0 |
| GPSFCKADEKPCKYHADCCNCCLGGICKPSTSWIGCSTNVFLT | 0 |
| GCKKDRKPCSYHADCCNCCLSGICAPSTNWILPGCSTSTFT | 0 |
| ECKTNKMSCSLHEECCRFRCCFHGKCQTSVFGCWVDPX | 0 |
| ECTPMHRACEEDEECCPNLECKCSNNPDCQSGYKCRP | 1 |
| QCTGAGFECEETPECCPNLTCKCSGSPLCTRYRCKT | 1 |
| RGCGTCDGKTCCGSCECPWGNCTC | 1 |
| MEKLTVLILVATVLLTIQVLGQSDRDKHLKRRPRQYATKRLSALMKGHRQCTGAGFECEETPECCPNLTCKCSGSPLCTRYRCKT | 1 |
| RDCQRGCHGCSNVPNGCCCGNLVCQNEQRCVPKEX | 0 |
| SGCGTCDGKTCCGRCECIWGNCRCRPW | 1 |
| SCGVRISSEICEQPEERICSCSNHMCCPLNPSQRDQCMARNVCFIMIGIYG | 0 |
| WCSERVSDETCVMMCRCLNHECCPLPPPSQNRCMPSDHCDFMSGRT | 1 |
| CIPEGTFCQFNADCCLSQCCWGSCGNPCRFP | 0 |
| GGIPCTGSGGFCSSYMWCCNSFDVCCEEFPGPARCMSESACSSIERWAQYTHFF | 0 |
| CDLEGMFCMYDFECCLSECCMGICAFGCT | 0 |
| CFTCNGGECCGSCMCSWDKDDCICV | 0 |
| SCEERISSEKCEAPGERICSCSNHVCCQLSAAKKDQCMTPFMCRTASGGN | 1 |
| CTSEGYSCSSDSNCCKNVCCWNVCESHCRHPGKR | 1 |
| CIPEGTYCQFNADCCLSQCCWGSCGNPCRFP | 0 |
| CHMDCSKMICCSGICCFYCGLPSCDDT | 0 |
| MEKLTVLILVATVLLAIQVLVQSDREKPLKGGVKQYAAKRLSALLRGPRECTPMHRACEEDEECCPNLECKCSNNPDCQSGYKCRP | 1 |
| MQKATVLLLALLLLLPLSTAQDAEGSQEDAAQREVDIATRCGGTGDSCNEPAGELCCRRLKCVNSRCCPTTDGC | 1 |
| MKLTCMVIVAVLLLTTCHLITADDSRGTQKHRSLRSTTKVSKSTSCMKAGSYCVATTRICCGYCAYFGKICIGYPKN | 0 |
| RGVCSTPEGSCVHNGCICQNAPCCHESGCNWANVCPGFLWDKN | 1 |
| MKQTCVLIIAVLFLIACQLIIADYSSDKQGYRAVRFRDAMRIVKGSKRDCGEQGEGCATRPCCEELSCVGSRPGGLCQYG | 0 |
| MKLVLAIVLILMLLSLSTGAETSDNSASSSATALRDRLLGRKVRTCQGMGESCSSNYDCCGSLCCAGASLFNGCVMGFVAC | 0 |
| MEKLTVLILVATVLLTIQVLAQSDRDKHLKRRPKQYATKRLSALMRGTRDCTPQNVRCEEKSECCPNLECKCTSAPDCNFYKCRT | 1 |
| MKLLCVLIVAMLPLMACHLIIADDSSEKQGYLTVRTSDKIQESEDSKLTERCFNDGDDCEIGDDCCSGSCIFDEGDSFCEISYRKLWWVSR | 0 |
| MKLTCVLIVAALFLTACQLIATDDSRDLQEFPRRKMSDVMLNTKDTEKRCLPGLATCNLHNNKCCNYCLIFWCS | 0 |
| MKLLCVLIVAMLPLMACHLIIADDSSEKQGYLTVRTSDKIQESEDSKLTERCFNDGDDCEIGDDCCSGSCIFDEGDSFCEISYENYGGVSR | 0 |
| MKLTCVLIVAVLFLTACQLIAADDYRDLQEFPRRKMSDRMLNRKDMGKRCLAPQRWCSMHDDSLHDDNCCKTCIILWCS | 0 |
| MKLLCVLIVAMLPLMACHLIIADDSSEKQGYLTVRTSDKIQESEDSKLTKRCLDDGDDCQVGDDCCSGSCVFDEGDSFCEISYENYGGVSR | 0 |
| MKLTCVLIVAVLFLTCQLIAADDYRDLQEFPRRKMSDGMLNRKDMEKRCLAPQRWCSMHDDNCCKTCIILWCS | 0 |
| MKLTCVLIVAVLFLTACQLIAADDYRDLQEFPRRKMSDRMLNRKDMEKRCLAPQRWCSMHDDNCCKTCIILWCS | 0 |
| EKSSEHSLSGATLGRDRRTCSGRGLRCIRDFHCCGGMCCYGNRCVVAVRWCYDLRF | 1 |
| QCTGAGFECEETPECCPNLTCKCSGSPLCTRYRCKT | 1 |
| DREKSSERSLSGAILRGVRRTCSRRGHRCRRDSQCCGGGYMCCKGNRCFVAIRRCFHLPF | 1 |
| MKLTCVLVIAVLFLTACQLIAADYSRDKQENSAVRSRDGMRNSGVSRSCGNLHESCSAHRCCPGLMCNGEASICVPY | 0 |
| TGEKSSERSLSGATLRRDRRTCSRPGHRCRLDFHCCGVMCCQGNRCLMGAIRRCFPFSF | 1 |
| MKMTCVLVIAVLFLTACHLTAADYSRDKQEYSAVRSRDGMRNSGVSRSCGNLHESCSAHRCCPGLKCIGTAHGGLCRE | 1 |
| EDCGSDCAPCGGECCCEPNSCIDGTCHHESSPN | 1 |
| VCCPFGGCHELCTAATVSAEHPWRCAGRFIQQRQRLLISCIIAPRLLSKNDEHDCTLVQISCSF | 0 |
| MKLTCVLIISVLFLMVCQLNTADDSRDKQEYRAVRLRDALRSSRGSRSCAQLGEPCSNNPCCPGVKCFTLPTPICIWN | 0 |
| MEKLTFLILVATVLLTIHVLVQSDGDKHLKRRPKQYATKRLSALMRGHRQCTPQNVKCEEDDECCSNLECKCSTVPDCNFPKCRP | 1 |
| MKLTCVLIITVLFLTACQLTTAVTYSRGEHKHRALMSTGTNYRLPKTCRSSGRYCRSPYDCCRRYCRRITDACV | 1 |
| MKLLCVLIVAMLPLMACHLIIADDSSEKQGYLTMRTSDKIQESEDSKLTKRCLDDGDDCEIGDDCCSGSCIFDEGDSFCEISYENYGGVSR | 0 |
| DVCESVAGRCIHNGCWCERSAPHGNCCNTSGCTARWWCPGTKWD | 1 |
| DVCDSLVDGRCIHNGCFCEESKPNGNCCDTGGCVWWWCPGTKWD | 1 |
| MSKTGLVLVVLYLLSSPVNLQQNEDDQAFSKIETRDRPECYNCFPNDDGHCVGTCCGEDSCKGGIRGCGCV | 1 |
| DKQKYRAVKLRDAIRNFKDSRSCGQQGQVCFSNLPCCSGLRCCVFAVGNWCLTSCI | 1 |
| DVCDSLVDGRCIHNGCFCEESKPNGNCCDSGGCVWWWCPGTKWD | 1 |
| MEKLTVLILVATVLLTIQVLGQSDGDKHLKRRPKQYATKRLSARMRGHRQCTGAGYECEETPECCPNLTCKCSGSPLCTRYSCQA | 1 |
| MTTSSYFLLVALGLLLYVRQSFSTHEHTCQLDDPAHPQGKCGSDLVNYHEEKCEEEEARRGGTNDGGKKRRRASPLRKRRRFISMLKARAKRRGYQGIACECCQHYCTDQEFINYCPPVTESSSSSSSAV | 1 |
| MTTSSYFLLVALGLLLYVRQSFSTHEHTCQLDDPAHPQGKCGSDLVNYHEEKCEEEEARRGGTNDGGKKRRRASPLWKRRRFLSMLKARAKRTGYKGIACECCQHYCTDQEFINYCPPVTESSSSSSSAA | 1 |
| MKLTCVLIIAVLFLIACQLIIADYSSDKQESRTVKLRNAIRNFKDSRSCGEEGEGCYTRPCCPERLKCIGTAHGALCREE | 1 |
| MKLTCVLIIAVLFLTACQLITAETYSRGKQMHRALRSTDKNSQLTRECTPPGGACGLPTHCCGFCDTANNRCL | 0 |
| QFFCPDSENDPLNCVETMATTAMCMQSKKDKSYSYACGYCGKKKESCFGDKVPVTDYNCKSRKIVNPCGGPAL | 1 |
| MKLTCVLIIAVLFLTACQLITAETYSRGKQHLMHRALRSTDKNSKLTRECTPPGGACGLPTHCCGFCDMANNRCL | 0 |
| MKLTCVLIVAVLVLTACQFTAAISQTQRLSKKCIEDNHACGLLHHSPYCCNGTCFIVCIP | 0 |
| MKLTCVLIVAVLILTACQVIAADEATNRATKRGCLMCWGSNVRCCEKANACVSINYECPKARR | 0 |
| CFPPGTFCSRYLPCCSGRCCSGWCTRRCSPRY | 1 |
| MKLTCVLIAAMLLLAVCQLDSADATETGCKKDGSWCWIPSECCIESCLITCWY | 0 |
| MEKLTFLILVATVLLTIHVLVQSVGDKHLKRRPKQYATKHLSALMRGHRQCTQQGYGCDETEECCSNLSCKCSGSPLCTSSYCRP | 1 |
| MKLSCVLIIAVLFLMACQLNTLSADDSTDKQEYRAVKLRYAMRNFIDSKKGRMCVHHGNGFCTPNSPCCGELRCHAALVGYRCRY | 1 |
| MKLTCVLIVAVLFLTACQIIAADDYRDLQKFPRRKMSDRMLNRKDMKKSCLAPDSWCIMDVDECCETCVLFWCT | 0 |
| MKLTCVLIVAVLFLTACQLSTADDSRDEQQDPLVRSHREEQKAEDPKTAERCSDIGSYCRPATHLCCSGECFGIEDMGLCNS | 1 |
| MKLTYVIVAVLFLTACQLIDADDSRYMHKTRLATLFRRRRDSDSGLAPGTCILFGSMCKENVSICCYRCDIEENCCKS | 0 |
| QCTGAGYECEETPECCPNLTCKCSGSPLCTRYSCQA | 1 |
| MKLVLAIVLILMLLSLSTGAEMSDNHASRSATALTDRLLGPKASICRGTGGRCTKDKHCCGWLCCGGPSVGCATSFAPCN | 0 |
| MKLTCVMIVAVLVLTVCKVVTSDQLKKLRRECYLEPGDSCFHDDGRGACCEGTCLFGINCVASW | 0 |
| DVCDSLVGGRCIHNGCWCERSAPHGNCCNTSGCTATFWCPGTLFD | 1 |
| MKLMCVSIVAVLFLTACQLITAEYAREKQGIGNDSAVRSSDKIQDSDDLKLTKRCTVNGVVCDPGNHNCCSGSCLDDEDTPVCGIHVEIQHVHMLSR | 0 |
| PECYNCFPNDDGHCVGTCCGEDSCKGGIRGCGCV | 1 |
| MKLTCVLIVVVLFLTACQLITTDDSTGKQRYQAWKLRSKMQNSVLSRLSKRCDEEGTGCSSDSECCSGRCTPEGLFEFCE | 1 |
| MKLTCVVIVAVLLLTACQLITADDSRGTQKHRALRSTTNLSMSTRCKPPGSKCTPTVYNCCTSCNPYSHKCRTPWG | 1 |
| QKELVPSTITTCCGNEPGTMCPKCMCDNTCPPKKRKDQAAETTDAPGPSEPRHAALCLTCFTFRLFVPLELYNSIH | 1 |
| QKELVPSTITTCCGNEPGTMCPKCMCDNTCPPKRRKDQAAETTDAPGPSEPRHAALCLTCFTFRLFVPLELYNSIH | 1 |
| SKPKTPECKRICKLEEKKCCCVRSEGPKCSRLCGLPMFC | 1 |
| GRPSARYDAPYCSEEELQACDCSHNPVRDACQCAYDPAGSPACDCYCVEPWRR | 1 |
| SCDSEFSSEFCEQPEERICSCSTHVCCHLSSSKRDQCMTWNRCLSAQTGN | 1 |
| QKELVPSTITTCCGHEPGTMCPKCMCDNTCPPKKRKDQAAETTDAPGPSEPRHAALCLTCFTFRLFVPLELYNSIH | 1 |
| EDCGSDCMPCGGECCCEPNSCIDGTCHHESSPN | 1 |
| DPSASLLSGGKDDLSVKRECGTCNGAECCGRCSCPFGTCSCVTIGK | 1 |
| DVCDSLVGGRCIHNGCYCERSAPNGNCCDTAGCTVLWWCPGTKFD | 1 |
| MMLSVTFLLILMILPSVTGEKSSEHTLRRLKLARIFRGGCSEIGEGCGHHFDCCGDMCCFHGTCAVSATGLGCDHF | 0 |
| QKELVPSTITTCCGHEPGTMCPKCMCDNTCPPKRRKDQAAETTDAPGPSEPRHAALCLTCFTFRLFVPLELYNSIH | 1 |
| MKLFLATVFILMLLSLNTGAETSDNRATRSATALRDRLLRPKRCQAQYENCWKNSQCCEEYCCTGASYCDHSIGRCDMGK | 1 |
| MEKLTILLLVAALLMSTQALIQGGGERRKRVTNKCGPKPVAECWWDDECKGWSNYCGQSPEECCSADCAFYCKLW | 1 |
| MRGSRQCTPKGQPCEEDGECCSNLECKCFTRPDCTSGYKCKD | 1 |
| MKLTCVLIVAVLILTACQVIAADGWFGEESSCWWCTGQNKCCEEAQVCQSVNYACPPARR | 0 |
| MEKLTMLILVAAAVLSIQVTAGGDGEKPLMGRITRNAANGLSALMRGKRCKGYHAECERDSECCSGDCHCLNAADCAGFTHKCR | 1 |
| MRFYLLLTVTLLLTSFTGGDARLMQETKTDRLENRDCATGCEGCNNPAKCCCGTQVCVNGNTCQ | 1 |
| QFFCPDSENDPLNCVETMATTATCMQSKKDQSYSYACGYCGKKKESCFGDKEPVTDYNCKSRNIVNPCVGPAL | 1 |
| CAVTHEKCSDDYDCCGSLCCVGICAKTIAPCK | 0 |
| QFFCPDSEDDPLNCVETMATTAMCMQSKKDKSYSYACGYCGKKKESCFGDKVPVTDYNCKSRNIVNPCGGPAL | 1 |
| QFFCPDSEDDPLNCVETMATTATCMQSKKDKSYSYACGYCGKKKESCFGDKVPVTDYNCKSRNIVNPCGGPAL | 1 |
| DRPSARYDAPYCSEEELQACDCSHNPVRDACLCQYDPAGSPACECFCVEPWRR | 1 |
| MKLTCVLIIAVLFLIACQLIIADYSSDKQGYRAVRFRDAMRNVKGSKRDCGEQGEGCATRPCCEELSCVGSRPGGLCQ | 1 |
| QKELVPSTITTCCGNEPGTMCPKCMCDNTCPPKKKKDQAAETTDAPGPSEPRHAALCLTCFTFRLFVPLELYNSIH | 1 |
| MKLTCVLIVAVLVLTACQFTAAIDSRGGQENPAPRSTGLMRRKSQTERNCIPKNHFCGLLHHSRNCCTPTCLIVCF | 0 |
| MKLTCALIVAVLFLTACQLIATDDSRGMQKHLAKRSRAKRLNYRLTRSCDPPGQECERLENNCCHACKIREKNPNVCSNE | 1 |
| ALLCGGVRASCSRHDDCCGSLCCFGTSTGCRVAVRPCW | 0 |
| MKLTCALIVAVLFLTACQLIATDDSRGMQKHLAKRSRAKMLNYRLTRSCDPPGYECERLENNCCDACKIRENNPNVCSNE | 1 |
| MKLTCVMIVAVLVLTVCKVVTSDQLKKLRRECYLEPGDSCFHDDGRGACCEGTCFFGVACVPWS | 0 |
| MKLLCVLIVAMLPLMACHLIIADDSSEKQGYLTVRTSDKIQESEDSKLTERCLNDGDDCDTGDDCCSGLCIFDEYFSYCDDSDPYYDDYDEYYY | 1 |
| MKLTCVLIVAVLFLTACQLITAANYARDEQEYPAVRSSDVMQDSEDLTLTKKCTDDSQFCNPSNHDCCSGKCIDEGDNGICAIVPENS | 0 |
| DVCDSLVGGRCIHNGCWCERSAPHGNCCNTGGCVWWWCPGTKWD | 1 |
| MKLTCVLIVAVLFLTACQLITAANYARDKQEYPAVRSSDEMQDSEDLTLTKKCTDSGQFCDPTNHDCCSGNCIDEGGNGVCAFVREDVPKLY | 1 |
| MKLTCVLIVAVLFLTACQLITAANYARDKQEYPAVRSSDEMQDSEDLTLTKKCTDSGQFCDPTDHDCCSGNCIDEGGNGVCAFVREDVPKLY | 1 |
| MKLTCVLIVIMLFLTVCPLITADYSRNKQEHPAMRLKDKIRYLRRGKWTRDCKPEGYSCAGEEPCCEGLRCMTSGGGAICVTQ | 1 |
| MKLTCALIITLLFLSITAGDSRGKHRYNALKSMSREANSTERECREKGQGCTNAALCCPGLECEARVKVVCAWTIKWRLMFPLGALSCAA | 0 |
| MKLTCALIITLLFLSITAGDSRGKHRYNALKSMSREANSTERECREKGQGCTNTALCCPGLECEGQSQGGLCVDNKWRLMFPLGALSCAA | 1 |
| MKLTCALIITLLFLSITAGDSRGKHRYNALKSMSREANSTERECREKGQGCTNTALCCPGLECEGQSQGGCAWTIKWRLMFPLGALSCAA | 0 |
| GRPSARYDAPYCSQEEVRECQDDCSGNAVRDSCLCAYDPAGSPACECRCVEPWRR | 1 |
| GTCSGRGQECKHDSDCCGHLCCAGITCQFTYIPCK | 1 |
| TCSNKGQLCGDDSDCCWHLCCVDNKCAHLILLCNL | 0 |
| EKSSERTLLGPLLKGNWCGLLGERGCRYHTDCCGDLCCFDDMCVMTVLPCDFPY | 0 |
| LCTPRNEPCYEDGECCPNLECRCRTVADCQAGYKCRV | 1 |
| MKTVAVFLVVALAVAYGQFFCPSSKDEPLNCIETMASTPTCMKSTADESLSYACGYCGKKKETCSGDKVPVSNYNCQIRKIPNPCGGPAL | 0 |
| MLKMPVLLLAILLLLPLATAQDDKRSQAHATQRRDAPPCAGSQSPCDEPAGQSCCGTLKCVSNRCCPTTDGC | 1 |
| MKLTCVLIVAVLILTACQVIAADEADANRLSTRWCACGVNYYCCNEVCTWREDPCP | 0 |
| MSGTGVLLLTLLLLVAMAASDMLSSLIQAHERDSEESCKSYGGGPCPSGEDCCCPPGRSTGTCKRTCNNGSVCA | 0 |
| MEKLTVLILVATVLLTIQVLGQSDRDKHLKRRPKQYATKRLSARMRGHRQCTGAGYECEETPECCPNLTCKCSGSPLCTRYSCQA | 1 |
| QFFCPDSENDPLNCVETMATTATCMQSKKDKSYSYACGYCGKKKESCFGDKVPVTDYNCKSRNIVNPCGGPAL | 1 |
| MKLTCVLIAAVLLLAVCQLDSADAITRDCKTKGYACFASTECCVQDCWLVCLY | 0 |
| TLLGPLLKGNWCGLLGERGCRYHTDCCGDLCCFDDMCVMTVLPCDFPY | 0 |
| CTDEGEYCTDDLQCCKLQCCRASCSDICRFP | 1 |
| TCSRPGHRCRLDFHCCGVMCCQGNRCLMGAIRRCFPFSF | 1 |
| CRNRFEECWEDSECCEELCCDGPNYCKDSIGRCH | 1 |
| TCSRRGHRCRRDSQCCGGGYMCCKGNRCFVAIRRCFHLPF | 1 |
| DAPPCAGSQSPCDEPAGQSCCGTLKCVSNRCCPTTDGC | 1 |
| MNLTCVLIINVLFLTACQVITADDSRDKQIYRAVRSRDGMRNFRSSRPCANLGRACDTVPCCLGVRCFESRTPTCLLKQRGV | 1 |
| CKGYHAECERDSECCSGDCHCLNAADCAGFTHKC | 1 |
| QCTPKGQPCEEDGECCSNLECKCFTRPDCTSGYKCKD | 1 |
| MKLTCVVIVAVLLLTACQLITADDSRGTQKHRALRSSTKLTLSTRCVPSGGSCSRTAYSCCHGSCSGGRCG | 0 |
| DCDYDYGKTCEGGEACECSNHICCDVLSQAKKDQCVRGHDVCYLLNQSSRRRRAIQMQ | 1 |
| MKLTCVLIIAVLFLIACQLIIADYSSDKQEYRTVKLRNAIRNFKDSRSCGEEGEGCYTRPCCSLGLKCIGIRSHGGLCREE | 1 |
| MKLTCVVIVAVLLLTACQLITALDSRGTQKHHALRSTTKLSMLRTSRDWCGDAGDACGTLKLRCCSGLCNQYSGTCTG | 1 |
| MKLTCVVIVAVLLLTACQLITADDSRGTQKHRALRSSTKLTLSTRCKSPGTPCSRTMRDCCTSCLSYSKKCRG | 1 |
| TCSLPGDGCIRDFHCCGHMCCQGNKCVVTVRRCFNFPY | 1 |
| MNLTCVLIIAVLFLTACQLIAADDSRDNQKHRAVRMRDALKNFKDSRACSGRGSRCPPQCCMGLTCGREYPPRCG | 0 |
| MNLTCVLIIAVLFLTACQLIAADDSRDKQKYRAVRLGDEMQIFKTREKLCGELYDGCHDQRCCPGLTCDTLFQCVRHS | 1 |
| MKCTVLLLISGVISCNDKQKYRAVRLGDEMQIFKTREKLCGELYDGCHDQRCCPGLTCDTLFQCVRHS | 1 |
| TCSGRGLRCIRDFHCCGGMCCYGNRCVVAVRWCYDLRF | 1 |
| QFFCPSSKDEPLNCIETMASTPTCMKSTADESLSYACGYCGKKKETCSGDKVPVSNYNCQIRKIPNPCGGPAL | 1 |
| GACRLEGMFCIHSPECCLQDCCSGICNSGACGKRAQHQRLHL | 1 |
| MNLTCVLIIAVLFLTTCQLITADDSRDKQKYRAVRLGDEIQIFKTRRRRCVGRDSKCGPPPCCMGMTCNYERVRKCT | 1 |
| CTEFGKECNLPPECCGRMCCYNSLCMWSSKEC | 1 |
| MKLTCVLIIAVLFLTACQLITGEQKDHALRSTDKNSKLTRQCSPNGGYCTLHIHCCSNHCIKPIGRCVA | 0 |
| CRAEGTYCENDSQCCLNECCWGGCGHPCRHP | 1 |
| MKLTCVLIVAVLFLTVCQLIPADYSRDKPGYPAWKLRTKMQNSRRSKLARSCKERASSCETPSECCSGVCRTRIFYLC | 0 |
| MKLTCVVIVAVLLLTACQLITADDSRGTQKHRALRSSTKLTLSTRCKSPGTPCSRGMRDCCTSCLLYSNKCRRY | 1 |
| MKLTCVVIVAVLLLTACQLITADDSRGTQKHRALGSTTELSLSTRCKSPGSSCSPTSYNCCRSCNPYTKRCYG | 1 |
| MKLTCVMIVAVLFLTACQLTTAGDSRDKEGYRAVRSIPSMQDGIDSRECRRRGQGCTQSTPCCDGLRCDGQRQGGMCVDS | 1 |
| MPRLEVMLLVLLLLPLPHFYAGGHAMQGNGRGHGMDQSFLRSVRMSQQRRSWGPACQTRMCGGICCGSDSCGCKGNECDCP | 1 |
| MKLTCALIVAMLLLTACQLITTDDFRGRQQYRTARARTKMQNYKIFRLTKRCDAPNAPCEKFDNDCCDACMLREKQQPICAV | 0 |
| MKLTCVLIVVVLFLTACQLIPADYSRDTPGYPAWKLKTKMQNSRRWKLAKRCKGKGAGCDYSHECCSRQCTGRIFQTCN | 1 |
| VTNKCGPKPVAECWWDDECKGWSNYCGQSPEECCSADCAFYCKLW | 1 |
| RDCATGCEGCNNPAKCCCGTQVCVNGNTCQ | 1 |
| ECSSDCVAECPNGNECCDGDLCVYSSVLETYYCIGCGSGGGE | 1 |
| KCPDNCPSTCPERDECCDGDSCLYNSYMRKYYCYDCGSGGPN | 1 |
| MKLVLAIVVILMLLSLSTGAEMSDNHASMSANALRDRLLGPKALLCGGTHARCNRDNDCCGSLCCFGTCISAFVPC | 0 |
| MKVTCVLVLTLMALTVCQVATAYCINVGMCIYDGYCCSNRCWGGMCSPWR | 0 |
| MEKLTILILVATVLLAIQVLVQSDGEKPLKGRVKQNAAKRLWVHMKGPRLCTPRNEPCYEDGECCPNLECRCRTVADCQAGYKCRV | 1 |
| RECGTCNGAECCGRCSCPFGTCSCVTI | 0 |
| MKLVLAIVVILMLLSLSTGAEMSDNHASRSATARRDRHLSPKAWPCGGVRASCSRHDDCCGSLCCFGTSTGCRVAVRPCW | 0 |
| MKLVLAIVVILMLLSLSTGAEMSDNHASRSATALRDRLLSPKASICYGTGGRCTKDKHCCGWLCCGGPSVGCVVSVAPCK | 0 |
| CNGRGEWCSTHRSCCDSGDVCCITTPVGPICTRGCSGRIIPQRRGAQLRHFF | 1 |
| MKLTCVVIVAVLLLTACQLITTEDSRGTQKHRALGSTTELSLSTRCKSPGSSCSPTSYNCCRSCNPYTKRCYG | 1 |
| RCQAQYENCWKNSQCCEEYCCTGASYCDHSIGRCDM | 1 |
| MKLTCVVIVAVLLLTACQLIAALDSRGMLKHRALRSTKVSKSPPCLVAGSSCRGTTRVCCGFCSHYGYKCRDRPTS | 0 |
| ASICRGTGGRCTKDKHCCGWLCCGGPSVGCATSFAPCN | 1 |
| MRFFFLLLTVALFLTSITGDDAERMLGMKEGGYVREDCGSDCAPCGGECCCEPNSCIDGTCHHESSPN | 0 |
| SCGQQGQVCFSNLPCCSGLRCCVFAVGNWCLTSCI | 0 |
| VRTCQGMGESCSSNYDCCGSLCCAGASLFNGCVMGFVAC | 0 |
| CLLSLETGSTSCTDVRIRWYYNQGNEACQPFQYTGCGGNDNNFYNQNDCEHCCKMDLQCNSAS | 1 |
| SDPQACEPTISGGEMICRDEVCASTGCNCGYNIAKAHCYCACP | 0 |
| MKLFLAIVLILMLQFLSTGAETSDNHASRSTTALRDWLLGPKAKRCAVTHEKCSDDYDCCGSLCCVGICAKTIAPCK | 0 |
| MEKLTVLILVATVLLTIQVLGQSDRDKHLKRRPRQYATKRLSALMKGHRQCTGAGFECEETPECCPNLTCKCSGSPLCTRYRCKT | 1 |
| MKLFLAIVLILMLLSLSTGAETSDNHASRSATALRDWLLGPLAKRCAVTHEKCSDDYDCCGSLCCVGICAKTIAPCK | 0 |
| QCKPKGDICEEDEECCPSLECECDSLPTCITNKRC | 1 |
| MKLTCVLIAAVLLLAVCQLDSADATAYMRKDPSLRSPKRTRGCKTKGTWCWASRECCLKDCLFVCVY | 0 |
| TCSSRGHRCTRDFHCCGGMCCYGYRCVVAFRPCYNHSV | 1 |
| MSGLGIMVLTLLLLVSMAISHRYAREKQATRRDVVNIRRRSKPKTPECKRICKLEEKKCCCVRSEGPKCSRLCGLPMFC | 0 |
| MKLTCALIVAMLFLTACQLTTTDDSRGRQKYPTERLRVKMRNPKLSKLTKTCDPPGDSCSRWYNHCCSKLCTSRNSGPTCSRP | 1 |
| CGGTGDSCNEPAGELCCRRLKCVNSRCCPTTDGC | 1 |
| SCDSEFSSEFCERPEESCSCSTHTCCHWARRDQCMKPQRCISAQKGNX | 0 |
| GRPSARYDAPYCSQEEVRECDDDCSGNPVRDACQCAYDPAGSPACDCYCVEPWRR | 1 |
| MKLTCVVIVAVLFLTACQLITAETYSRGEQKHRALSSTDKNSKLTRTCNTPTQYCTLHRHCCSLYCHKTIHACA | 1 |
| MEKLTVLILVAIVLLTIQVLGQSDRDKHPKRRPRQYATKRLSALMKGHRQCTGAGFECEETPECCPNLTCKCSGSPLCTRYRCKT | 1 |
| MTTSSYFLLVALGLLLYVFQSSFGGEHVCWLGDPNHPQGICGPQVADIVEIRCEEKEAEQGGANNARANTGRTSSLMKRRGFLSLLKKRGKRDEGSPLQRSGRGIVCECCKHHCTKEEFTEYCH | 1 |
| QKIVCDQEEMFCTIDGECCLHECCLGKCSSPCIP | 0 |
| VPCQQGGGKCSSDLKCCDGRDVCCGTSGSATCTIESECSGERITHTHRALHARFF | 1 |
| IPCNEGGGWCSTHMWCCDLFHVCCDSPGQAVCKTDSECSWPHIPQNRGALYTRFF | 0 |
| GGCSEIGEGCGHHFDCCGDMCCFHGTCAVSATGLGCDHF | 1 |
| MEKLTVLILVATVLLMIQVLAQSGGDKHLKRRPKQYATKRLSALMRGHRQCTPQNVQCEEDDECCSNLECKCSTVPDCNFPKCRP | 1 |
| MKLTCVLIIAVLILTACQFIAADNTEYRKWRRSGTSTGMRLGSRDCGPWCWGQNKCCPDESCRSLHESCT | 1 |
| DCTPQNVRCEEKSECCPNLECKCTSAPDCNFYKCRT | 1 |
| GRPSARYDAPYCSQEEVRECDDDCSGNPVRDACQCAYDPAGSPACDCFCVEPWRR | 1 |
| CIPEGSSCSSSGSCCHKSCCRWTCNQPCLIPX | 0 |
| MKLTCLLIIAVLFLTACQLITANDPRDNQEYRAVRMKDALNFKDSRACSGRGSRCPPQCCMGLRCGRGNPQKCIGAHEDV | 0 |
| CNGRGEWCSTHRSCCDSGDVCCITPVGPICTRGCSGRIIPQRRGAQLRHFF | 1 |
| MSSPLRMDVTFLLAAIAVTWVCGLKIGFPGFSTPPRSFIQHPKRTLCPEDCDIASPFKCEESPTCLRLFQVCNGRWDCEHGSDEDNALCAAVLRPLECMIWEFLEGQRDWILPNLFNDANTDLVAHALHEAYSMGDLQSYLNLTDQNIENIRNSTRGAIVGDPRPLMALGMPDRAWPEVMYLLKELYNLGLDVWAE | 0 |
| MKLCVTFLLVLVILPSVTGEKSSERTRSFADLPDDWGMCSDIGEGCGQDYDCCGDMCCDGQICAMTFMACMF | 0 |
| MKLCVTFLLVLVILPSVTGEKSSERTLSGAALRVDRGTCSYLGEGCKRDSDCCGHFCCGGKTCVITARPCKV | 1 |
| MSGHTSVNFLLLSIVALGMVATVICSCDSEFSSEFCEQPEERICSCSTHVCCHLSSSKRDQCMTWNRCLSAQTGNRRSTHMQKRFLRMPRDLAD | 0 |
| MFGHTSVSFLLLSIVALGMVATVICSCDSEFSSEFCEQPEERICSCSTHVCCHLSSSKRDQCMTWNRCLSAQTGNRRSTHMQKRFLRMPRDLAD | 0 |
| NNKVCCNQRNLPMSEAEILDKKVITLTHVNKKAVTSCPGSDIDGCCPGYTMCMSTNAQNNVHTAHTSCLNRPCFGPC | 1 |
| MVRVTSVGCFLLVIVFLNLIVPTTTSDCIPEGSHCGISSDCCKSTCCMETCEDPCRYTWKRAKLQELFRQR | 0 |
| MTTSSYFLLVALGLLLYVCQSSFGGEHVCWLDDPNHPEGICGPQVSDIVEIRCEEKEAEQGGANNARAYTGRTSSLMKRRGFLSLLKKRGKRDEGSLQRSGRGIVCECCKHHCTKEELTEYCH | 1 |
| MLKMGVVLFIVLVLFPLATLQLDADQPVERYAENKQLLNPDERRGIILHALGQRVCCPFGGCNMIPLLSSGLRRGIILHALGQRVCCPFGGCHELCYCCDG | 0 |
| MVRVTSVSCFLLVIVFLNLVVLTNACISEESPCSSSGSCCQRSCCYGNCRFPCLQPGKRAKLREFFRQR | 0 |
| MTTSSYFLLVALGLLLYLCQSSFGTEHTCEPGASPHPQGKCGPELAEFHETMCEVEESLQGGTDDARKKRGRASLLRKRRGFLSMLKARAKRNEASPLPRAGRGIVCECCKNSCTYEEITEYCPPVTEGSG | 1 |
| MYTGCKMEGRRFAAVLILAICMLAPRASASRPHVLRRVCKQPAETGRCCRVIQRYYFDMDSYDCKKFNYKGCGGNGNNFRTYNECYVTCGRKYVNWWYWRHP | 1 |
| MVRVTSVGCFLLVIVFLNLVVPTNACAGNEEACTVASDCCGSFNCCNGYCDAPCRQPGKRVKLREFFRQR | 0 |
| MTTSSYFLLVALGLLLYLCQSSFGTEHTCEPGASPHPQGKCRPELAEFHETMCEVEESLQGGTDDARKKRGRASLLRKRRGFLSMLKARAKRNEASPLPRAGRGIVCECCKNSCTYEEITEYCPPVTEGSG | 1 |
| MPSVRSVTCCCLLWMMFFHAFATGMGGCIWTILLFHAYGAHFPGRIVSARLLSLCWVYRCLLLPVLCLWLFASSASESCSFVTLYWELVTRCR | 0 |
| MKLCVTFLLVLVILPSVTGEKSSEHTLSGAALRGDRGTCSGRGQECKHDSDCCGHLCCAGIKCHFTYFPCK | 0 |
| MKLCVTFLLVLVILPSVTGEKSSERTLSGAALRGDRRTCSDLGQACVHESECCAQMCCLHKKCAMTHCNFY | 0 |
| MDARRVAAVFLLLSMARCVRADPDPRCLLSLETGSTSCTDVRIRWYYNQGNEACQPFQYTGCGGNDNNFYNQNDCEHCCKMDLQCNSASR | 1 |
| MKLCVTFLLVLVTLPLVTGEKSSERSLSGVILRGVRRTCSLPGDGCIRDFHCCGHMCCQGNKCVVTVRRCFNFPY | 0 |
| MKLCVTFLLVLVTLPLVTGEKSSEHSLSGATLRRDRRTCSSRGHRCTRDFHCCGGMCCYGYRCVVAFRPCYNHSV | 1 |
| MMFRLTSVGCFLLVIVFLNLVVLTDACTDEGEYCTDDLQCCKLQCCRASCSDICRFPGKRMHGHGLLRFFGQR | 0 |
| VGCFLLVIVFLNLAGVQVVLGGACRLEGMFCIHSPECCLQDCCSGICNSGACGKRAQHQRLHLRRF | 0 |
| MKLCLTFLLVLMILASVTGEKSSKHTLSRAARVKNRGPSFCKADEKPCKYHADCCNCCLGGICKPSTSWIGCSTNVFLTR | 0 |
| MATSSCFLLVTLGLLLHVQQAFLHEHTCSPSEPAAPGGICGSNLAELHSFLCEKELEDYSGSALKKRGRPSRRMKRRDFLSALKTRVKRKEGRSVKRSPTSGMSCECCKNSCDAEEILEYCPPLPSS | 1 |
| MKLCLTFLLVLMILASVTGEKSSKHTLSRAARVKNRGPSFCKADEKPCEYHADCCNCCLSGICAPSTNWILPGCSTSSFFKI | 0 |
| MTPFILLLLFSLTIRCGDGKAIQEDRDPSAGLLTGDKNHDLSVKRGCGTCDGKTCCGSCECPWGNCTCRKWGK | 1 |
| MTPFILLLLFSLTIRCGDGKAIQEDRDPSAGLLTGDKNHNLSVKSGCGTCDGKTCCGRCECIWGNCRCRPWGK | 1 |
| MTTSSYFLLVALGLLLYVCQSSFGGEHVCGSNQPNHPNGKCGSKMADYLEEQCEEEEAAHGGTNDARATTGRALSLSKRRGFLSMLKRRGKRNEASPLQRAGRGIVCECCKNHCTDEEFTEYCPHVTESG | 1 |
| MMSTKGITLFLCLLLLALATSVNGGQGTRRSRMTRALHGGRPSARYDAPYCSEEELQACDCSHNPVRDACLCQYDPAGSPACECFCVEPWRR | 1 |
| MTPFILLLLFSLTIRCGDGKAIQEDRDPSAGLLWGDKNHDLSVKRRCFTCNGGECCGSCMCSWDKDDCICV | 0 |
| MVGRTSVSFLLFSIMVLGMVATVICSCEERISSEKCEAPGERICSCSNHVCCQLSAAKKDQCMTPFMCRTASGGNRRRRSTQVQDRFLRMARGLAD | 1 |
| MMFRLASVSCFLLVIVFLDLVVLTNACYPEGDYCEYHYQCCKGSCCFAYCRDPCRRVGKKAQLQEFLRHR | 1 |
| MKLCVTFLLVLVILPSVTGVKSSERTLSGAALRGDRGTCSGRGQECKHDSDCCGHLCCAGITCQFTYIPCK | 0 |
| MKLCVTFLVVLVILPSVTGEKSSERTLSGAALRGDRRTCSNKGQLCGDDSDCCWHLCCVDNKCAHLILLCNL | 1 |
| MVRVTSVGCFLLVIVFLNVVVLTNACAPPGSSCNHTDNKCCISACCFGACVLPCQVPGKRAKLLEFFRQR | 0 |
| MKLCVTFLLVLVILPSVTGEESSERTLSGATLTGDRRTCSDLGQACVHESDCCAQMCCLNKKCAMTMPPCNFY | 0 |
| MVGHTAVRFLLLSILLLHMAAMVSCDCDYDYGKTCEGGEACECSNHICCDVLSQAKKDQCVRGHDVCYLLNQSSRRRRAIQMQKRSRGMLRGLA | 1 |
| MVRVTSVGCFLLVIVSLNLVVLTNACIPEGSSCSSSGSCCHKSCCRWTCNQPCLIPGKRAKLLEFFRQR | 0 |
| MSGHTSVSFLLLSIVALGMVATVICSCDSEFSSEFCERPEESCSCSTHTCCHWARRDQCMKPQRCISAQKGNGRRRLIHMQK | 0 |
| MMFRLTSVSCFLLFIVFLNLVVLTNACIPEGTYCQFNADCCLSQCCWGSCGNPCRFPGKREKLQEFFRQR | 0 |
| MKLCLTFLLVLMILASVTGEKLSEQTLRRAARKNKGPRCWVGRVHCTYHKDCCPSVCCFKGRCKPQSWGCWSGPT | 1 |
| MSGHTSVSFLLLSIVALGMVATVICSCDSEFSSEFCERPEESCSCLTHSCCHWSRRDQCMTPQRCISAQKGNGRRRLTHMQK | 0 |
| MMFRVTSVGCLLLVIVFLNLVVPTSACRAEGTYCENDSQCCLNECCWGGCGHPCRHPGKRSKLQEFFRQR | 1 |
| MKLTCVLVVLLLLLPYGDLITNNYIRGAARKVTPWRRNLKTRDVCDSLVGGHCIHNGCWCDQDAPHGNCCDTDGCTAAWWCPGTKWD | 0 |
| QXPSTAELCKINSNACSVPFSXIPCQKXFLAACDRHDTCYHCGKHFGFKQDDCDDAFFRDMTALCAHGTDDEGXCPX | 0 |
| MMSTKGITLFLCLLLLALATSVNGGQGTRRSRMTRALHGGRPSARYDAPYCSQEEVRECHDDCSGNPVRDACQCAYDPAGSPACDCYCVEPWRR | 1 |
| MKLCVAFLLVLVILPSVIGGKPSERTLSGATRRGDRRMCLSLGQRCERHSNCCGYLCCFYDKCVVTAIGCGHY | 0 |
| MFRLTSVGCILLVIAFLNLVGLTNACTSEGYSCSSDSNCCKNVCCWNVCESHCRHPGKRTRLQGFFKHRR | 0 |
| MTTLGMTMLVLLLLLPLATCLGDGERSPWDSLLRALRSDPQACEPTISGGEMICRDEVCASTGCNCGYNIAKAHCYCACP | 0 |
| MMFRLTSVSCILLVIAFLNLVGLTNACTSEGYSCSSDSNCCKNVCCWNVCESHCGHHGKRATFQ | 0 |
| MFGHTSVSFLLLSIMALGMVATVICLCDSYISSELCEHPEETCFCPNHMCCPLSPYRQDQCMYWEACHIFRKPVGSR | 0 |
| MARFLSILLCFAMATGLAAGIRYPDRVLGRCSTHDLSKMEIDTNLDGVYSPHRSFCTCGSGEVYFTAKDRRNHSNYRVYVCGMPTEFCTAENPVRDPKKGNRWLQCRCRQYKMVIYRDWLVLCE | 1 |
| MALTWPSSPPVLLTLLLSLLALQLCAVYGSYEHTCTLATRSRGAHPSGICGRNLARIVSVLCTPRGYVSNWFTKRSAPNKPAETFVDQNLRGVLLNKREALSYLRPREPRATRGTFGSQGITCECCFNQCTYYELLQYCN | 1 |
| MAMSMSMTLSVFVMVVMAATVTGFTQLKKPDLSRMKRNNKVCCNQRNLPMSEAEILDKKVITLTHVNKKAVTSCPGSDIDGCCPGYTMCMSTNAQNNVHTAHTSCLNRPCFGPCK | 0 |
| MMFRVTSVGCFLLVIVFLNLSPGFTYTRTALQRTLSQTGKRDYPEHVWEWHLSRCSDEGASCEKKSDCCFLSCCWSVCDRPCRLVPGKRAQLQKFLRHR | 1 |
| MMFRVTSVGCFLLVIVFLNLFVLTNACDSDGTSCTSNMECCGYGCCSGTCQTPCRFGPGKRERLQEFFQHR | 1 |
| MRCYLLSTVTLLLIWFSGGDAGPRHAIGDFVRRDCQTGCVGCHNPAGCCCGGQVCVDNKRCEGGFY | 1 |
| MKLTCVLIIAVLCLTVCQLITADYLRDKQKYRSVRLRDGMLNFKGSRQCADLGEECYTRFCCPGLRCKDLQVPTCLLA | 0 |
| MKLTCVLVVLLLLLPYGDLITNNYIRGAARKVTPWRRNLKTRDVCDSLVGGHCIHNGCWCDQEAPHGNCCDTDGCTAAWWCPGTKWD | 0 |
| MMFRVTSVGCLLLVIVFLNLVVPTSACRAEGTYCENDSQCCLNECCWGGCGHPCRHPGKRSKLQEFFRQR | 1 |
| MMFRLTSVGCFLLAIVLLNVAVLTNASCRNEGAMCSFGFQCCKKKCCMSHCTDFCRNPGKRAHGHGLLRFWGQR | 0 |
| MMFRLTSVGCFLLVIVLLNVAVLTNASCRNEGAMCSFGFQCCKKECCMSHCTDFCRNPDKRAHGHGLLRFWGQR | 0 |
| KESCTKHSNGCSTPLRLPCQEYFRPACDIHDNCYHCGTIFGISRKECDDAFLKDMNTLCKKLGSNSATCPARG | 1 |
| KESCTKHSNGCSTPLRLPCQEYFRPACDIHDNCYHCGTIFGISRKECDDAFLKDMNTLCKKLGSNSATCPARG | 1 |
| MKLTCVLVVLLLVLPFGDLITTSNTEDNKRGATPWQNSLKARGVCSTPEGSCVHNGCICQNAPCCHPSGCNWANVCPGFLWDKN | 0 |
| MKLTCALIVAVLFLMACQLITAENSRGSREYSAVRSSDKIRDSDDRQLTKECTQDFDPCMPVCHECCTRSHFVVCRRPICLRRIF | 0 |
| MKLTCMLVVLLLVLPFGDLIANTGGLCGMPPGVCYPNGCACGQDTPCCHPSGCNRYNYCGPLLE | 0 |
| MKVTCVLVVLLLLLPYGDLLGNSVCDFGSCVHNGCYCEEHRPCCTPGSCSSWWPRCPGSMMDP | 0 |
| MKLTCVLVVLLLFLPYGDLITNNYIGGAARKVTPWRRNLKTRDVCDSLVGGNCIHNGCWCDQEAPHGNCCDTDGCTAAWWCPGTKWD | 0 |
| MKLTCVLVVLLLVLPFGDLIGVCSTPEGSCVHNGCICQNAPCCHPSGCNWVNVCPGFLWDRS | 0 |
| FLLLSITVLGMVATVICSCNGRISSENCEQPGEQICSCPNHECCHFHPPKRDQCMTRGMCYMALAGMPGRRSTQMQERFLRIPRGLAE | 0 |
| MFGNTSVSFLLLSITVLGMVATVICSCKYGEWTEKCGQPGETCECPNHVCCDLKRDVCMPSKSCKALIHKIVFRRSTRV | 0 |
| MKLTCVLVVLLLVLPFGDLITTSNTEDNKRGATPWQNSLKARGVCSTPEGSCVHNGCICQNAPCCHASGCNWANVCPGFLWDKN | 0 |
| MFGHTSVRFLLLSIMLLGMAEMVLCTCDSEFGETCEQEKVCSCSNHGCCEAASSAKTDQCMTQGACMAWLSDNVKRRAIQKQKRFLDMVRGLA | 1 |
| MKLTCVLVVLLLVLPFGDLITTSNTEDNKRGATPWQNSLKARGVCSTPEGSCVHNGCICQNAPCCHPSGCNWVNVCPGFLWDKN | 0 |
| MFGHTSVRFLLLSIMLLGMAEMVLCTCDSEFGETCEQEKVCSCSNHGCCEAASSAKTDQCMTQGACVAWLSDNVKRRVIQKQKRFLGMVRGLA | 0 |
| MKLCVTFLLVLVILPSVTGEKSSERTRIGAVLKGHWCGYPGERGCRYHSQCCGDMCCYDRKCVVTAMPC | 0 |
| MKLCVTFLLVLVILPSVTGEESSERTLSGATLTGGRGMCSLLGQRCGDHSDCCWDMCCASEMCVVTFLPCK | 0 |
| RTTELCKINSNGCSVQFDWVPCQEHFLPACDIHDNCYFCGAHFSLSRLNCDDAFLSDMIALCADGTDEESDCPAKRK | 1 |
| TSEQSCKRFSNGCSTPLPLPCQEYFRPACDRHDSCYQCGAHFGINRKQCDDAFSDHMHALCDELGLLGMCPARRK | 1 |
| MMPVILLLLLSLAIRCADGKAVQGDSDPSASLLTGDKNHDLPVKRDCTTCAGEECCGRCTCPWGDNCSCIEWGK | 0 |
| MMFRVTSVGCFLLVILSLNLVVLTNACLSEGSPCSMSGSCCHKSCCRSTCTFPCLIPGKRAKLREFFRQR | 0 |
| MKLCVTFLLILVILPSVTGEKSSKRTLSGAALRGDWGMCSGIGQGCGQDSNCCGDMCCYGQICAMTFAACGP | 0 |
| MFGHTSVSFLLLSIMALGMVPTVICWCSERVSDETCVMMCRCLNHECCPLPPPSQNRCMPSDHCDFMSGRTGRRRSTQMLRAIAN | 0 |
| MMFRVTSVGCFLLVIVLLHVAAVTSDDCRPEAAYCEYNEQCCIDKCCQASCSDACRTPGKRVHGHGLLRFFGQR | 1 |
| KESCTKHSNGCSTPLRLPCQEYFRPACDIHDNCYHCGKTFGISREECDKAFLKDMNTLCKKLGSNSATCPARG | 1 |
| LGMVATVICSCESGVSGETCDLSVEKRCSCSRHICCTHHALEQHKCMTWAKCMSVSLGVNGRRSIQMQDRFLRMLRAFDD | 1 |
| KESCTKHSNGCSTPLRLPCQEYFRPACDIHDNCYHCGTIFRISRKECDDAFLKDMNTLCKKLGSNFATCPARG | 1 |
| MTTSSYFLLVALGLLLYVCRSSFGSEHTCESDASPHPQGVCGSPLAEAVEAACELEESLQGGTGKKRGRASLLRKRRAFLSMLKARAKRNEASPLQRSGRGIVCECCKNHCNIEELTEYCPPVTEGSG | 1 |
| MTTSSYFLLVALGLLLYVCRSSFGSEHTCESDASPHPQGVCGSPLAEAVEAACELEESLQGGTGKKRGRASLLRKRRAFLSMLKARAKRNEASPLQRSGRGIVCECCKNHCNLEELTEYCPPVTEGSG | 1 |
| MTTSSYFLLVALGLLLYVCRSSFGSEHTCESDASPHPQGVCGSPLAEAVEAACELEQSLQGGTGKKRGRASLLRKRRAFLSMLKARAKRNEASPLQRSGRGIVCECCKNHCNIEELTEYCPPVTEGSG | 1 |
| MKLTCVLVVLLLLLPYGDLITNSYIRGAARKVTPWRRNLKTRDVCDSLVDGRCIHNGCFCEESKPNGNCCDTGGCVWWWCPGTKWD | 0 |
| RTDELCKINSNGCSVPSSWIPCQQHFLAACDRHDTCYMCGAHFSLTQKDCDDAFLVHMTALCGHGTDDEGFCLEKR | 1 |
| MFGHTSVSFLLLSIMALGMVPTVICWCSERVSDETCVMMCRCLNHECCPLPPPSQNRCMPSDHCDFMSGRTGRRRSTQMLRTIAN | 0 |
| MFGHTSVSFLLLSIMVLGVVATVICSCGVRISSEICEQPEERICSCSNHMCCPLNPSQRDQCMARNVCFIMIGIYGGRRSTRMQERFLRMSRGLADYSV | 0 |
| MTTSSYFLLVTLGLLLYVCRSSFGTEHTCESDASPHPQGVCGSPLAEAVEAACELEEYLQGGTGKKRGRASPLRKRRAFLSMLKARAKRNEASPLQRSGRGIVCECCKNHCNIEELTEYCPPVTEGSG | 1 |
| STAELCKINSNGCSVPSSWIPCQQHFLAACDRHDTCYLCGAHFNLTQNDCDNAFLAHMIALCADGTDDEGFCLEKR | 1 |
| MMFRLTSVWCLLVIVLLNSAVDGFIPCTGSEGYCHSHMWCCNSFDVCCELPGPATCTREEACETLRIALGRRAQYKRFFRR | 0 |
| MFGHTSVSFLLLSIMALGMVATVICSCDSEFSSEFCEQPEERICSCSTHVCCHLSSSKRDQCMTWNRCLSAQTGNRRSTHMQKRFLRMPRDLAD | 0 |
| MMFRLTSVSCFLLFIVFLNLVVLTNACIPEGTFCQFNADCCLSQCCWGSCGNPCRFPGKREKLQEFFRQR | 0 |
| MFGHTSVSFLLLSIMALGMVATVICLCDSYISSELCEHPEETCFCPNHMCCPLSPYRQDQCMYWEACHIFRKPVGSR | 0 |
| MKLCATFLLVLVTLPLVTGEKSSERSLSGAILRGVRRTCSRRGHRCIRDSQCCGGMCCQGNRCFVAIRRCFHLPF | 1 |
| SRAELCKINSNGCSVPFPDVPCQEHFRPACDIHDNCYLCGALFGFTQGKCDRAFLTHMTALCARGTDDEGFCLEKR | 1 |
| MSGHTSVNFLLLSIVALGMVATVICLCDSYISSELCEHPEETCFCPNHMCCPLSPYRQDQCMYWEACRIFRKPVGSRSTHMQKKIPSYAT | 0 |
| KESCTKHSNGCSTPLRLPCQEYFRPACDIHDNCYHCGKTFGISREECDEAFLKDMNALCEKLGRDSATCPATR | 1 |
| KESCTKHSNGCSTPLRLPCQEYFRPACDIHDNCYHCGKTFGISREECDKAFLKDMNTLCKKLGSNFATCPARG | 1 |
| MKCLQLLLVLLLISTIAALYQDGRATQRRDGNIRTMSDLLNIQKRECSSDCVAECPNGNECCDGDLCVYSSVLETYYCIGCGSGGGE | 0 |
| MSGHTSVNFLLLSIVALGMVATVICLCDSYISSELCEHPEETCFCPNHMCCPLSPYRQDQCMYWEACHIFRKPVGSRSTHMQKIPSYAT | 0 |
| MSGHTSVNFLLLSIVALGMVATVICSCDSEFSSEFCEQPEERICSCSTHVCCHLSSRKRDQCMTWNRCLSAQTGNRRSTHMTKRFLRMPRDLAD | 0 |
| MFGHTSVSFLLLSIVALGMVATVICSCDSEFSSEFCEQPEERICSCSTHVCCHLSSSKGDQCMTWNRCLSAQTGNRRSTHMQKRFLRMPRDLAD | 0 |
| MMFRLTSVGCILLVIAFLNLVGLTNACTSEGYSCSSDSNCCKNVCCWNVCESHCRHPGKRTRLQGFFKHRR | 0 |
| MSGHTSVNFLLLSIVALGMVATVICLCDSYISSELCEHPEETCFCPNHMCCPLSPYRQDQCMYWEACHTFRKPVGSRSTHTQKKIPSYAT | 0 |
| MNCLQLLLVLLLISTITALYPDGWATLRRGKTIRRMSNLLNIQKRKCPDNCPSTCPERDECCDGDSCLYNSYMRKYYCYDCGSGGPN | 1 |
| GCTLVNNCRKRNGACNGDCHCKGKICKCSSSARPWKPGCACTCRNA | 1 |
| MNMSMTLSVFVMVVMAATVIGSTLSQEPDLSRMKRYSRSCCINETYECLKGYPGQEHIYYSKCHFDAAAPCGADVYPGCCNGYMYCIAIYVQDNGLEPTHNYCKDRPCNPPSR | 0 |
| MMFRLTSVGCFLLVIACLNLFQVVLTRRCFPPGVYCTRHLPCCRGRCCSGWCRPRCFPRFGKRATFQE | 0 |
| MMCRLTSLCFLLVIVLLNSAVDGIPCHGPGGWCSTHMWCCDPHNVCCDFPGAARCTRKRECSWPTIPQGRLAQLSHFFRR | 0 |
| MVMSMVAASGTENHAMCKVHSDGCSVPLGLPFFYKSRFTPACHIHDVCYGCGAKYGRTKEDCDIAFLQDMIHACHTARRRRSSRNKRTLCVSVATNMYYKAVHWFANPHYHTADDLDPHCHENWVPKCLPPKYH | 1 |
| MMFRFTSVSCFLLVIVCLNLVVLTNACRLEGSSCRRSYQCCHKSCCIRECKFPCRWVGKRATFRELILHH | 0 |
| TCDPTPDCRTTVCETDTGPCCCPHGYNCQTTNSGRRACVLVCPHNCPP | 1 |
| MPKLAVVLLVLLILPLSYFDAAGGQAVQGDRRGNGLARYLQRNGRDNEAECQIDTPGSSWGKCCMTRMCGTMCCSRSVCTCVYHWRRGHGCSCPG | 1 |
| AVKKTCIRSTPGSNWGRCCLTKMCHTLCCARSDCTCVYRSGKGHGCSCTS | 1 |
| MPKLEMMLLVLLIFPLSYFIAAGGQVVQVDRRGDGLAGYLQRGDRDVQDCQVSTPGSKWGRCCLNRVCGPMCCPASHCYCVYHRGRGHGCSC | 0 |
| MPKLEMMLVVLLIFPLFYFDAAGGQAVQGDRRGDGLARYLQRGDRNDESECIISTPGSSWGRCCLTRMCGTMCCPRSGCYCVYHWRRGHGCACSD | 1 |
| MSTLGMLLLVALLLPLDNPADNGDGQAKPRSRNLRSLDFGRTPRRLDKRTCDPTPDCRTTVCETDTGPCCCPHGYNCQTTNSGRRACVLVCPHNCPP | 1 |
| GCTLVNNCEKNGACNGDCHCKGKICRCSSSARPWKPGCACTCRNA | 1 |
| MPRYFFHQSSQTCELFYYGGCGGNSNNFRTLEGCEGACVSGEAVQDVCALPKVAGPCFAAFPRFYFDKTAGRCKTFTYGGCHGNQNNFRSLRACRNTCPGN | 1 |
| MMFRLTSVSCFLLVIACLNLVVLTNACLRDGQSCGYDSDCCRYSCCWGYCDLTCLIIGKRATFQELILHR | 0 |
| MPRLEVMLLVLLILAGGQAVQGDGQGDGMDRYLQRGDRADYAMCFRHRPGSLWGRCCLTKMCGSACCNHSFCRCTHDKDMGHGCAC | 1 |
| DVRECQVDTPGSSWGKCCMTRMCGTMCCSRSVCTCVYHWRRGHGCSCPG | 1 |
| DNEAECQIDTPGSSWGKCCMTRMCGTMCCSRSVCTCVYHWRRGHGCSCPG | 1 |
| MAMNVSMTLGVFVMIVVAAIVIDSTRSQKSEPGRKKRYNRQCCIDKTYDCLKKYRGRENTFASVCQQEAAVYCGAWDEAEGCCYGYSHCMSMYAQQSGLDVAHNGCKDRKCDNPGR | 1 |
| FELLPSQDRSCCIQKTLECLENYPGQASQRAHYCQQDATTNCPDTYYFGCCPGYATCMSINAGNNVRSAFDKCINRLCFDPGHX | 0 |
| MPKLAVVLLVLLILPLSYFDAAGGQAVQWDRRGNGLARYLQRGDRDVRECQVDTPGSSWGKCCMTRMCGTMCCSRSVCTCVYHWRRGHGCSCPG | 1 |
| KAARGWLFNEVETCELGGLGDPCSGSGDCCCDQCLCSGSYEHCTQNPDRWFCCRTYGN | 1 |
| MPKLAVVLLVLLILPLSYFDAGGQAVQGDWRGNRLARDLQRGGRDDESECIINTRDSPWGRCCRTRMCGSMCCPRNGCTCVYHWRRGHGCSCPG | 0 |
| ALVSQCRPCPTCRECKCRECKCRECQCRIHSCLSAWDSRGIWMRT | 1 |
| GCDPTDGCQTTVCETDTGPCCCKPNFTCQISNSGTKSCSCSGQPSDCPV | 1 |
| MSKSGMVLFVLLLLLPLAFPKLVPAGRSVARRYGNLGAKREVPLTCHPPSTPNLNGPWQDQKCCLTKQCSPTNCCTSTSCVCDMSICDCSGM | 0 |
| DDESECIINTRDSPWGRCCRTRMCGSMCCPRNGCTCVYHWRRGHGCSCPG | 1 |
| MEKLIILLLVASLLVTTDSVVKGKKAARGWLFNEVETCELGGLGDPCSGSGDCCCDQCLCSGSYEHCTQNPDRWFCCRTYGN | 1 |
| SSCSGTCYDSANCDGTCYCREDNCWCTGDLSCACQCA | 1 |
| MSTLGILLPIALLLPLANPAENGDGQAMPRTRNLRSLSFGRTLRRLEKRGCDPTDGCQTTVCETDTGPCCCKPNFTCQISNSGTKSCSCSGQPSDCPV | 1 |
| MSNMGAMFVLLLLFTLASSQWEEDVQARKTHLKSDFYRTLPRFARGCTISCEYQDNRCRGECHCPGKTNCYCTSGHHNKGCGCAC | 1 |
| MMLFMFAAIIFTMATATVSATQCNSVQTLCSWEGMGELCNCSATGSCPQNDEHKIVVGTQDIYVCQQISDFEVCTGSVAIDSSLTELNCRCSSNAYKIEGSQVVCD | 0 |
| MMLSSYVPVYIAALTTMMAISGEASSSISFWNNNATCIEDETCGYKYSSFGLGVQYCMCPGSILCDIDGPATAVLLGENKTYYTCEAPTDYQECGANEYPTEIIIMENRNLNRTTKFVCRCPNEHHRFNQTLRRLECY | 0 |
| MPKLAVVLLVLLILPLSYFDAAGGQAVQGDRRGNGLARYLQRGDREVQECQVDTPGSSWGKCCMTRMCGTMCCSRSVCTCVYHWRRGHGCSCPG | 1 |
| DRDVQDCQVSTPGSKWGRCCLNRVCGPMCCPASHCYCVYHRGRGHGCSC | 1 |
| MNMSMTLSVFVMVVMAATVIGSTLSQEPDLSRMERNSRSCCINKTYQCLKGYPGQEHIYITKCHFDAAAPCGADVYPGCCNGYMYCIGIYVQDNGLEPTHNYCKDRHCNPPSR | 0 |
| MMFRLTSVSCFLLVIACLNLFQVVLTRRCFPPGVYCTRHLPCCRGRCCSGWCRPRCFPRYGKRATFQ | 0 |
| EVQECQVDTPGSSWGKCCMTRMCGTMCCSRSVCTCVYHWRRGHGCSCPG | 1 |
| NDESECIISTPGSSWGRCCLTRMCGTMCCPRSGCYCVYHWRRGHGCACSD | 1 |
| MMFRLTSVSCFLLVIACLNLFQVVLTSRCFPPGIYCTPYLPCCWGICCGTCRNVCHLRIGKRATFQE | 0 |
| MKLTCVLIVAVLILAACQFTAANMARYGKTQIARSDVKSIDARRPKCCCVCGVVGRKCCSTWKDCHPVHLPCPSSG | 0 |
| CTDSCEFQGVLCRGTCDCYGTNNCYCAINHNKQCICVCTN | 1 |
| MMFRLTSVSCFLLVIACLNLFQVVLTSRCFPPGIYCTPYLPCCWGICCDTCRNVCHLRIGKRATFQE | 0 |
| MPKLEMMLLVLLILPLPYFDAAGGQSVHVDGYGDGLARYLQRGERAVKKTCIRSTPGSNWGRCCLTKMCHTLCCARSDCTCVYRSGKGHGCSCTS | 1 |
| MMFRLTSVSCFLLVIACLNLFQVVLTSRCFPPGIYCTPYLPCCWGICCGTCRNVCHLRFGKRATFQE | 0 |
| NCDSPNFQAWAMCCLESRCGTGGCCSQEVCDCNGPQSKECNCPP | 1 |
| SVPISCVNPSTPNLQGSWQDKKCCSTKLCSPTNCCESSTCSCVEGSCQCL | 1 |
| MAMNMSMTLSMFVMVVVAATVTGSTRFQKRGLRHMKRDDCCIGNTYGCLKRRPGQEHEQVMPCKHEATIRCPGSDIDGCCPGYATCMSIFAKDNLIPAHYHCEKRPCYT | 0 |
| MAMNMSMTLSVFVMVVVAATITGFTHLQEPDPSRMKRYDRGCCINRTYQCLKRYRNRENKFASFCQQEAAVNCGTFTSGKGCCYGYMHCMMINAETQTRETAHQYCQNRSCDPEE | 1 |
| MATFNIVLAALFASVILVDFAVQARRCHPPCEYGERCVSANRRERRHRHNNVCVPVRCLFQARRGRCLNFDRRYHFNTLTMSCTRVHTGACYGRNNRFSSSENCELTCAPRR | 1 |
| DVQECQVVTPGSKWGRCCLNRVCGPMCCPASHCYCIYHRGKGHGCSC | 1 |
| MMFRLTSVGCFLLVIACLNLFQVVLTRRCFPLGTFCSRYLPCCSGMCCSGWCTRRCAPRFGKRATFQE | 0 |
| DVQECQVVTPGSKWGRCCLNRVCGPMCCPASHCYCIYHRGRGHGCSC | 1 |
| MPRLEMMLLVLLILPLPYFNAAGGQAVQGDGQGDRMDRYLQRGDRAVIATCHPNYPGSPWGRCCTTKMCGSVCCNYAHCSCVYHSDMGDGCSC | 1 |
| MMFRLTSVSCFLLVIVCLNLFQVVLTRRCVPPSRYCTRHRPCCRGTCCSGLCRPMCNLWYGKRATFQE | 0 |
| DVQDCQVSTPGSKWGRCCLNRVCGPMCCPASHCYCVYHRGRGHGCSC | 1 |
| SAKLLAHTTRDSCPSGTNCPSKICCNGNCCSKSSCRCETNQATKERVCVC | 1 |
| MMLKMGAMFVLLLLFILPSSQQEGDVQARKTHLKRGFYGTLAMSTRGCSGTCHRREDGKCRGTCDCSGYSYCRCGDAHHFYRGCTCSCQG | 1 |
| MMLKMGAMFVLLLLFTLASVQQEDVQARKTRLKRDFHRALRQSPLGCPGTCGKGGKCVGSCECTMYKNCYCSNNGIRQPGCSCTCPTR | 1 |
| MATNMLMTLSVFVMVVMAATVVGSTPLPEPELSRSVRDSRTCCIDSTLQCLRGYPGEEYTYATMCNLEASGPCGLSVYQGCCNGYMNCIRINVGNLRLEGAHNACKNRRC | 0 |
| DAQGCQVVTPGSKWGRCCLNRVCGPMCCPASHCYCIYHRGRGHGCSC | 1 |
| MMFRLTSVGCFLLVIACLNLFQVVLTRRCFPPGTFCSRYLPCCSGRCCSGWCTRRCFPRFGKRATFQE | 0 |
| QSPLGCPGTCGKGGKCVGSCECTMYKNCYCSNNGIRQPGCSCTCPT | 1 |
| GCSGTCHRREDGKCRGTCDCSGYSYCRCGDAHHFYRGCTCSCQ | 1 |
| ADYAMCFRHRPGSLWGRCCLTKMCGSACCNHSFCRCTHDKDMGHGCAC | 1 |
| MPKLAVVLLVLLILPLSYFDAAGGQVVQGDRRGNGLARYLQRGDRDVRECQVNTPGSSWGKCCMTRMCGTMCCARSGCTCVYHWRRGHGCSCPG | 1 |
| MPKLAVVLLVLLILPLSYFDAAGGQVVQGDRRGNGLARYLQRGDRDVRECQVNTPGSKWGKCCMTRMCGTMCCARSGCTCVYHWRRGHGCSCPG | 1 |
| MLKLEMMLVVLLILPLFYFDAGGQVVQGDWRSDGLARYLQRGDRDVRECNINTPGSSWGKCCLTRMCGTMCCARSGCTCVYHWRRGHGCSCPG | 1 |
| MLKLEMMLVVLLILPLFYFDAGGQVVQRDWRSDGLARYLQRGDRDVRECNINTPGSSWGKCCLTRMCGPMCCARSGCTCVYHWRRGHGCSCPG | 1 |
| MMLKMGAMFAILLLFALSSSQQEGDVQARKIRLRNDFLRTSRMIFTRGCGGSCHTSPGCGGNCECNSPVPCYCSGTETCVCVCSG | 0 |
| MMFCVTSVRCFLLVIVFLNLVVLTNACRTEGMSCEENQQCCWRSCCRGECEAPCRFGPGKRAQLQEFFQHR | 1 |
| MSKSGMLLFVLLLLLPLAIPELAPAGRSVTHHFRDFGAKRSVPISCVNPSTPNLQGSWQDKKCCSTKLCSPTNCCESSTCSCVEGSCQCL | 0 |
| MMFRLTSVSCFLLVIACLNLFQVVLTSRCFPPGIYCTPYLPCCWGICCGTCRNDNSSLTFLQFCLPFFFFLRPSHPLFLLLPAR | 0 |
| MMFRLTSVSCFLLVIVCLNLVVLTNACRLEGSSCRRSYQCCHKSCCIRECKFPCRWDGKRATFQ | 1 |
| MMFRLTSVSCFLLVIVCLNLFQVVLTRRCFPPGTFCSRYLPCCSGRCCSGWCTRRCFPRFGKRATFQE | 0 |
| GCTRTCGGADCTGSCECTFSSNCGCEFHGGPGAWGCACVCS | 1 |
| MSTLGMMLLILLLLVPLATFADDGPTMRGHRSAKLLAHTTRDSCPSGTNCPSKICCNGNCCSKSSCRCETNQATKERVCVC | 1 |
| DECGLGTHRPVKEVIDNVRTMYYCDCRAGDAERSITVSRCDDNNQKQDDVILTYCGLEQTTGCNTNPYTAAKHDSSGDKPQFYCSCLNYKYEQSHADSRYWTIRCYMGDICD | 1 |
| DECGLGTHRPVKEVIDNVRTMYYCDCRAGDAERSITVSRCDDNNQKQDDVILTYCGLEQTTGCNTNPYTAAKHDSSGDKPQFYCSCLNYKYEQSHSDYRHWTIRCYMGNICD | 1 |
| GCGGSCHTSPGCGGNCECNSPVPCYCSGTETCVCVCS | 1 |
| LHCHEISDLTPWILCSPEPLCGGKGCCAQEVCDCSGPVCTCPPCL | 0 |
| DDCCIGNTYGCLKRRPGQEHEQVMPCKHEATIRCPGSDIDGCCPGYATCMSIFAKDNLIPAHYHCEKRPCYT | 1 |
| CHPPCEYGERCVSANRRERRHRHNNVCVPVRCLFQARRGRCLNFDRRYHFNTLTMSCTRVHTGACYGRNNRFSSSENCELTCAP | 1 |
| AVIATCHPNYPGSPWGRCCTTKMCGSVCCNYAHCSCVYHSDMGDGCSC | 1 |
| MKLTCVVIVAALLLTACQLITALDCGGTQKHRALRSTIKLSLLRQHRGWCGDPGATCGKLRLYCCSGFCDCYTKTCKDKSSA | 0 |
| MPKLEMMLLVLLILPLSYFSAAGGQVVQGDWRGDGLARYLQRGDRDAQGCQVVTPGSKWGRCCLNRVCGPMCCPASHCYCIYHRGRGHGCSC | 0 |
| MPKLEMMLLVLLILPLSSFSAAGEQVVQGDQRSDGLARYLQRGGRDVQECQVVTPGSKWGRCCLNRVCGPMCCPASHCYCIYHRGRGHGCSC | 0 |
| MPKLEMMLLVLLILPLSSFSAAGEQVVQGDRRSDGLARYLQRGGRDVQECQVVTPGSKWGRCCLNRVCGPMCCPASHCYCIYHRGKGHGCSC | 1 |
| GCTISCGYEDNRCQGECHCPGKTNCYCTSGHHNKGCGCAC | 1 |
| AVMMMMAMPVKADQCDDHPTVNGCTTPSFLDLQHEKTFTPACNRHDVCYGCGVKYGVTRTQCDEAFLRDMKEACRLERRRRKLTVNVNMDCPDMANAFHTAVWAFGHSHYVDAGTPNSDCLEELDKSCLP | 1 |
| MVRRTSVSCCVLLVIVLLNLGSAIMVQKKQKIVCDQEEMFCTIDGECCLHECCLGKCSSPCIPGKRALRDDLLSFIRQR | 0 |
| MAMSMSMTLSVFVMVVMAATVTQLKKPDLSRMKRYDRRCCINKTYECLKNYRNRENKFASFCQQEAAVYCGTFESGTGCCYGYMNCMMLNAERDGQGKAHGYCQHRTC | 1 |
| MAMSMSMTLSVFVMVVMAATVTQLKKPDLSRMKRYDRRCCINKTYECLKNYRNRENKFASFCQQEAAVYCGTFESGTGCCYGYMNCMMINAERDGREKAHGYCQHRIC | 1 |
| MMLRLTSVSCFLLVIACLNLFQVVLTRRCFPPGTFCSRYLPCCSGRCCSGWCTRRCSPRYGKRATFQE | 0 |
| SGSTCTCFTSTNCQGSCECLSPPGCYCSNNGIRQRGCSCTCPGTX | 0 |
| GCTRTCGGPKCTGTCTCTNSSKCGCRYNVHPSGWGCGCACSX | 0 |
| MPKLEMMLLVLLILPLYYFSAAGGQVVQGDWRGEGLARYLQRGDRDAQECQVVTPGSKWGRCCLNRVCGPMCCPASHCYCIYHRGRGHGCSC | 1 |
| MKLTCVVIVAVLLLTACQLITALDSRGMEKCRALRSTTKLSKLRQVSWWCGKPGDTCGKLYLKCCSGRCSGSCSGKCLP | 1 |
| GCCINRTYQCLKRYRNRENKFASFCQQEAAVNCGTFTSGKGCCYGYMHCMMINAETQTRETAHQYCQNRSCDPEE | 1 |
| ALADVGECTHCGGADCTGSCTCTNWSSCVCKYFSSSGAGECGCACYD | 1 |
| HAFLLWADGDCTCGKTSSCRTGTCGCRGSNCKCERGVYRSAEKCHCVCPQ | 1 |
| SGSTCTCFTSTNCQGSCECLSPPGCYCSNNGIRQRGCSCTCPGT | 1 |
| MPKLEMMLLVLLIFPLSYFIAAGGQVVQVDRRGDGLAGYLQRGDRDVQDCQVSTPGSKWGRCCLNRVCGPMCCPASHCYCVYHRGRGHGCSC | 0 |
| MPKLAVVLLVLLILPLSYFDVAGGQAAEGDRRGNGLARYPQRGGRDNEAECQINTPGSSWGKCCMTRMCGTMCCARSGCTCVYHWRRGHGCSCPG | 1 |
| CCINKTYECLKNYRNRENKFASFCQQEAAVYCGTFESGTGCCYGYMNCMMLNAERDGQGKAHGYCQHRTC | 1 |
| GCSGTCRRHRDGKCRGTCECSGYSYCRCGDAHHFYRGCTCTC | 1 |
| MMSKMGAMFVLLLLFTLASSQQEGDVQARKTRPKSDFYRALPRSGSTCTCFTSTNCQGSCECLSPPGCYCSNNGIRQRGCSCTCPGT | 1 |
| MMSKMGAMFVLLLLFTLASSLQEGDVQARKTRLKSDFYRALARDDRGCTRTCGGPKCTGTCTCTNSSKCGCRYNVHPSGWGCGCACSG | 1 |
| GCGGTCYDSQHCDGTCYCPAGDCYCGTEGPHSGCTCIC | 1 |
| PCQSVRPGRVWGKCCLTRLCSTMCCARADCTCVYHTWRGHGCSCVM | 1 |
| PARDCRGTCGSTDRCTGTCECEGYESCSCSNSGQHSGCTCSC | 1 |
| GCSGTCHRRQNGECQGTCDCDGHDHCDCGDTLGTYSGCVCIC | 1 |
| GCSGTCHRREDGKCRGTCDCSGYSYCRCGDAHHFYRGCTCTC | 1 |
| GCSGTCHRREDGKCRGTCDCSGYSYCRCGDAHHFYRGCTCSCQ | 1 |
| MMSKMGAMFVLLLLFTLASSLQEGDVQARKTRLKSDFYRALADVGECTHCGGADCTGSCTCTNWSSCVCKYFSSSGAGECGCACYD | 0 |
| MMSKMGAMFVLLLLFTLASSQQEGDVQARKTRPKSDFYRALPRSGSTCTCFTSTNCQGSCECLSPPGCYCSNNGIRQRGCSCTCPGTG | 1 |
| MMSKMGAMFDLLLLFTLASSQQEGDVQARKTRLKSGFHRALAMDDRGCTRTCGGADCTGSCECTFSSNCGCEFHGGPGAWGCACVCSG | 1 |
| QYPLGCPGTCGKGGKCVGTCECTNYTNCYCSSNGFRQPGCSCTCPT | 1 |
| QYPLGCPGTCGKGGKCVGTCECTNYTNCYCSSKGFREPGCSCTCPT | 1 |
| QYPLGCPGTCGKGGKCVGTCECTNYTNCYCSGKGFRQPGCSCTCPT | 1 |
| FGPICTCFKSQNCRGSCECMSPPGCYCSNNGIRERGCSCTCPGT | 1 |
| QYPLGCPGTCGKGGKCVGTCECTNYTNCYCGKGFRQPGCSCTCPT | 1 |
| QYDRKCTLVNNCQQNGACNGDCSCEGQICKCGYRVSPGKSGCACTCRNA | 1 |
| CCINKTYECLKNYRNRENKFASFCQQEAAVYCGTFESGTGCCYGYMNCMMINAERDGREKAHGYCQHRIC | 1 |
| MFVLLLLFTLASSQQEGDVQARKTRLKSDFYRAWRGCTLVNNCRKRNGACNGDCHCKGKICKCSSSARPWKPGCACTCRNAK | 1 |
| MMFRLTSVSCFLLVIACLNLVVLTNACLRDGQSCGYDSDCCRYSCCWGYCDLTCLINGKRATFQ | 0 |
| MMSKMGAMFVLLLLFTLASSQQEGDVQARKTRLKSDFYRALPRFGPICTCFKSQNCRGSCECMSPPGCYCSNNGIRERGCSCTCPGTG | 1 |
| MSTLGMLLLIALLLPLTNPADNGDGQAKPRSRNLRSLDFMRTHRRLDKRGCDPTDGCKKALCNTDTGPCCCQHGHNCQTQPSGRRACVRNCPHNCP | 1 |
| MMFRLTSVSCFLLVIVCLNLFQVVLTRRCFPPGTFCSRYLPCCSGRCCSGWCTRRCFPRFGKRATFQE | 0 |
| MFVLLLLFTLASSQQEGDVQARKTRLKSDFYRAWRGCTLVNNCEKNGACNGDCHCKGKICRCSSSARPWKPGCACTCRNAK | 1 |
| GCDPTDGCKKALCNTDTGPCCCQHGHNCQTQPSGRRACVRNCPHNCP | 1 |
| NSRSCCINKTYQCLKGYPGQEHIYITKCHFDAAAPCGADVYPGCCNGYMYCIGIYVQDNGLEPTHNYCKDRHCNPPS | 1 |
| YSRSCCINETYECLKGYPGQEHIYYSKCHFDAAAPCGADVYPGCCNGYMYCIAIYVQDNGLEPTHNYCKDRPCNPPS | 1 |
| MSGHTSVNFLLLSIVALGMVATVICLCDSYISSELCEHPEETCFCPNHMCCPLSPYRQDQCMYWEACLIFRKPVGSRSTHMQKKFLRMPRDLADQCV | 0 |
| LHCHEISDLTPWILCSPEPLCGGKGCCAQGVCDCSGPACTCPPCL | 0 |
| LHCHEISDLTPWILCSPEPLCGGKGCCAQEVCDCSGTACTCPPCL | 0 |
| LHCYEISDLTPWILCSPEPLCGGKGCCAQEVCDCSGPACTCPPCL | 0 |
| MPKLEMMLLVLLIFPLSYFIAAGGQVVQVDRRGDGLAGYLQRGDRDVQDCQVSTPGSKWGRCCLNRVCGPMCCPASHCYCVYHRGRGHGCSC | 0 |
| LHCHEISDPTPWILCSPEPLCGGKGCCAQEVCDCSGPVCTCPPCL | 0 |
| MPKLAVVLLVLLILPLSYFDAAGGQAVQGDWRGNRLARDLQRGGRDDESECIINTRDSPWGRCCRTRMCGSMCCPRNGCTCVYHWRRGHGCSCPG | 1 |
| TCCIDSTLQCLRGYPGEEYTYATMCNLEASGPCGLSVYQGCCNGYMNCIRINVGNLRLEGAHNACKNRRC | 1 |
| DAQECQVVTPGSKWGRCCLNRVCGPMCCPASHCYCIYHRGRGHGCSC | 1 |
| LHCHEISDLTPWILCSPEPLCGGKGCCAQEVCDCSGPACTCPPCL | 0 |
| AVDSVQPTRQCMSCGPEGVGQCVGPSVCCGLGLGCLMGTPETEVCQKENESSVPCAISGRRCGMDNTGNCVADGI | 0 |
| MPKLEMMLLVLLILPLSYFSAAGGQVVQGDLHSDVLARYLQRGDRDARECQVNTPGSRWGKCCLNRMCGPMCCPESHCYCIYHRRRGHGCSC | 1 |
| MPKLEMMLLVLLILPLSYFSAAGGQVVQGDLRSDVLARYLQRGDRDARECQVNTPGSRWGKCCLNRMCGPMCCPESHCYCVYHRRRGHGCSC | 1 |
| TGQKSILGSPGCHLVGHTRVVRIPDCVPFQITTNACRGFCVSYAIPSPYQTLVYNPNHIITSRAACCDIIDTLDIPVQVTCVDGVREIVFKSARSCACSIC | 0 |
| TTLQCHVRSYTFRATKPPIVNENGDPVTCQGDVRVSSCWGRCDSSEIGDYKMPFKISNHPVCTYTGRVSRTVRLSQCAGYPDPTVQVFDATGCACQFCNSETQLCEKLNX | 0 |
| MKLFMFAAIIFTMASTTVRAEQCANNRKVCTWDGQGDTNCDCIGTACHEDDAHKVSVAGSAFYTCQPISAFRVCDGSEDVMDAGFSELYCRCSGGSYTISNGEVVCD | 0 |
| DARECQVNTPGSRWGKCCLNRMCGPMCCPESHCYCIYHRRRGHGCSC | 1 |
| DARECQVNTPGSRWGKCCLNRMCGPMCCPESHCYCVYHRRRGHGCSC | 1 |
| KCNFDKCKGTGVYNCGESCSCEGLHSCRCTYNIGSMKSGCACICTYY | 1 |
| YNRQCCIDKTYDCLKKYRGRENTFASVCQQEAAVYCGAWDEAEGCCYGYSHCMSMYAQQSGLDVAHNGCKDRKCDNP | 1 |
| MMLKMGAMFVLLLLFILPSSQQEGDVQARKTHLKSGFYGTLAMSTRGCSGTCRRHRDGKCRGTCECSGYSYCRCGDAHHFYRGCTCTC | 1 |
| MPKLAVVLLVLLILPLSYFDAAGGQAAEGDRRGNGLARYLQRGGRDNEAECQINTPGSSWGKCCLTRMCGPMCCARSGCTCVYHWRRGHGCSCPG | 0 |
| CVLCVTFLLILVILPSLTGEKSSERTLSGAALRHKWGACSLLGKDCGHSPDCCGDLCCIGGKCVMTDIPCHYFPF | 0 |
| MMSKMGAMFVLLLLFTLASSQQEGDVQARKTHPKREFQRILLRSGRKCNFDKCKGTGVYNCGESCSCEGLHSCRCTYNIGSMKSGCACICTYY | 1 |
| VDPCAQYANGCSVPLHMPLFYKTLFTPSCNRHDVCYRCGAKYGISKDTCDSAFLHHMEAACAVHDASRRHISLQSSSSSSASHLQKRSACTVFAKDVFYEAVHIFGGLFYHDVDGTASFCSEPTAVSCLHD | 1 |
| REVPLTCHPPSTPNLNGPWQDQKCCLTKQCSPTNCCTSTSCVCDMSICDCSGM | 1 |
| KVGLPFVLLLLLTSTSPKQERDVQARKRSLNSDLYRSLARSTRGCGGTCYDSQHCDGTCYCPAGDCYCGTEGPHSGCTCIC | 1 |
| DNEAECQINTPGSSWGKCCMTRMCGTMCCARSGCTCVYHWRRGHGCSCPG | 1 |
| MFVLGLSMMLAVAGRVVMGVDPCAQYANGCSVPLHMPLFYKTLFTPSCNRHDVCYRCGAKYGISKDTCDSAFLHHMEAACAVHDASRRHISLQSSSSSSASHLQKRSACTVFAKDVFYEAVHIFGGLFYHDVDGTASFCSEPTAVSCLHD | 0 |
| DVQDCQVSTPGSKWGRCCLNRVCGPMCCPASHCYCVYHRGRGHGCSC | 1 |
| DDESECIINTRDSPWGRCCRTRMCGSMCCPRNGCTCVYHWRRGHGCSCPG | 1 |
| DLRQCTRNAPGSTWGRCCLNPMCGNFCCPRSGCTCAYNWRRGIYCSC | 1 |
| MSGHTSVNFLLLSIVALGMVATVICLCDSYISSELCEHPEETCFCPNHMCCPLSPYRQDQCMYWEACHIFRKPVGSRSTHMQKKIPSYARDLADQCV | 0 |
| DVRECNINTPGSSWGKCCLTRMCGPMCCARSGCTCVYHWRRGHGCSCPG | 1 |
| MMLKMGAMFVLLLLFTLASSQQEGDIQARKTHLKSGFYRTLPRFARGCTISCGYEDNRCQGECHCPGKTNCYCTSGHHNKGCGCAC | 1 |
| QCTLVNNCDRNGERACNGDCSCEGQICKCGYRVSPGKSGCACTCRNA | 1 |
| LKMGAMFVLLLLFTLASSHREGDIQARKTHLKSDFYRTLPRFARGCTISCGYEDNRCQGECHCPGKTNCYCTSGHHNKGCGCAC | 1 |
| DNEAECQINTPGSSWGKCCLTRMCGPMCCARSGCTCVYHWRRGHGCSCPG | 1 |
| GCTISCEYQDNRCRGECHCPGKTNCYCTSGHHNKGCGCAC | 1 |
| SSSISFWNNNATCIEDETCGYKYSSFGLGVQYCMCPGSILCDIDGPATAVLLGENKTYYTCEAPTDYQECGANEYPTEIIIMENRNLNRTTKFVCRCPNEHHRFNQTLRRLECY | 1 |
| LKMGAMFVLLLLFTLASSQQEGDVQARKTSLKSDFYRALRQYDRQCTLVNNCDRNGERACNGDCSCEGQICKCGYRVSPGKSGCACTCRNAK | 1 |
| MMFRLTSVSCFLLVIACLNLFQVVLTSRCFPPGIYCTPYLPCCWGICCDTCRNVCHLRFGKRATFQE | 0 |
| DVRECNINTPGSSWGKCCLTRMCGTMCCARSGCTCVYHWRRGHGCSCPG | 1 |
| DVRECQVNTPGSSWGKCCMTRMCGTMCCARSGCTCVYHWRRGHGCSCPG | 1 |
| LHCHEISDLTPWILCSPEPLCGGKGCCAQEVCDCSGPVCTCPPCL | 0 |
| MMLKMGAMFVLLLLFILPSSQQEGDVQARKTHLKRGFYGTLAMSTRGCSGTCHRREDGKCRGTCDCSGYSYCRCGDAHHFYRGCTCSCQG | 1 |
| MMLKMGAMFVLLLLFILPSSQQEGDVQARKTHLKRGFYGTLAMSTRGCSGTCHRREDGKCRGTCDCSGYSYCRCGDAHHFYRGCTCTC | 1 |
| TQCNSVQTLCSWEGMGELCNCSATGSCPQNDEHKIVVGTQDIYVCQQISDFEVCTGSVAIDSSLTELNCRCSSNAYKIEGSQVVCD | 0 |
| MSTRGCSGTCHRRQNGECQGTCDCDGHDHCDCGDTLGTYSGCVCIC | 1 |
| MKLLLTLLLGSALMCITLADECGLGTHRPVKEVIDNVRTMYYCDCRAGDAERSITVSRCDDNNQKQDDVILTYCGLEQTTGCNTNPYTAAKHDSSGDKPQFYCSCLNYKYEQSHSDYRHWTIRCYMGNICD | 1 |
| MMLKMGAMFVLLLLCSFTFTNIKARRTFWKRDLYGDLAGRSSCSGTCYDSANCDGTCYCREDNCWCTGDLSCACQCA | 1 |
| DNKACCTNAIYQCLKRNPGQESYNTPPCHHAATTRCPGSHIDGCCPKYATCMSVNAQNNLETAHTYCLPRPCFDPCE | 1 |
| MAMNMSMTLSVFVMVVMAATVTGFTQLKKPDLSRMKRDNKACCTNAIYQCLKRNPGQESYNTPPCHHAATTRCPGSHIDGCCPKYATCMSVNAQNNLETAHTYCLPRPCFDPCE | 1 |
| MVMPLVLSLALTPPPLCHATLVDPRTTLQCHVRSYTFRATKPPIVNENGDPVTCQGDVRVSSCWGRCDSSEIGDYKMPFKISNHPVCTYTGRVSRTVRLSQCAGYPDPTVQVFDATGCACQFCNSETQLCEKLNG | 1 |
| MWKMGVMLCVVFLLSPLASLRQEGEVQARKIDLKSNLYGAWKRPARDCRGTCGSTDRCTGTCECEGYESCSCSNSGQHSGCTCSC | 1 |
| MLRLITAAVLVSACLAYPQKKRTPPQTRPTSRALVSQCRPCPTCRECKCRECKCRECQCRIHSCLSAWDSRGIWMRT | 0 |
| MKLLLTLLLGSALMCITLADECGLGTHRPVKEVIDNVRTMYYCDCRAGDAERSITVSRCDDNNQKQDDVILTYCGLEQTTGCNTNPYTAAKHDSSGDKPQFYCSCLNYKYEQSHADSRYWTIRCYMGDICD | 1 |
| MMCRLTSLCCLLVIVLLNSAVDGIPCNEGGGWCSTHMWCCDLFHVCCDSPGQAVCKTDSECSWPHIPQNRGALYTRFFRR | 0 |
| MSRMGVVCFLLLLFTLASTRQEGDAEARKIDNLNNRHAFLLWADGDCTCGKTSSCRTGTCGCRGSNCKCERGVYRSAEKCHCVCPQ | 1 |
| MMCRLTSLCCLLVIVLLNSAVDGVPCQQGGGKCSSDLKCCDGRDVCCGTSGSATCTIESECSGERITHTHRALHARFFRR | 0 |
| MPLVLSLALTPPPLCHATLVDPRTTLQCHVRSYTFRATKPPIVNENGDPVTCQGDVRVSSCWGRCDSSEIGDYKMPFKISNHPVCTYTGRVSRTVRLSQCAGYPDPTVQVFDATGCACQFCNSETQLCEKLNG | 1 |
| MPKLEMMLLVLLILPLPYCNAAGVTTVQWGGHGDGLDRYLQRGVRDVHRPCQSVRPGRVWGKCCLTRLCSTMCCARADCTCVYHTWRGHGCSC | 0 |
| MMCRMTSLCCLLVIVLLDSAVDACNGRGEWCSTHRSCCDSGDVCCITTPVGPICTRGCSGRIIPQRRGAQLRHFFRR | 1 |
| MMCRMTSLCCLLVIVLLNSAVDACNGRGEWCSTHRSCCDSGDVCCITPVGPICTRGCSGRIIPQRRGAQLRHFFRR | 1 |
| MPKLEMMLLVLLILPLCYIDAVGPPPPWNMEDEIIEHWQELHCHEISDLTPWILCSPEPLCGGKGCCAQEVCDCSGPVCTCPPCL | 0 |
| MPKLEMMLLVLLILPLCYIDAVGPPPPWNMEDEIIEHWQKLHCHEISDLTPWILCSPEPLCGGKGCCAQEVCDCSGPVCTCPPCL | 0 |
| MPKLEMMLLVLLILPLCYIDAGLLEPWVLEEKEIIQRWIDRNCDSPNFQAWAMCCLESRCGTGGCCSQEVCDCNGPQSKECNCPP | 0 |
| MPKLEMMLLVLLILPLCYIDAVGPPPPWNMEDEIIEHWQKLHCHEISDLTPWILCSPEPLCGGKGCCAQGVCDCSGPACTCPPCL | 0 |
| MPKLEMMLLVLLILPLCYIDAVGPPPPWNMEDEIIEHWQKLHCHEISDLTPWILCSPEPLCGGKGCCAQEVCDCSGTACTCPPCL | 0 |
| MPKLEMMLLVLLILPLCYIDAVGPLPPWNMEDEIIEHWQKLHCYEISDLTPWILCSPEPLCGGKGCCAQEVCDCSGPACTCPPCL | 0 |
| MPKLEMMLLVLLILPLCYIDAVGPPPPWNMEDEIIEHWQKLHCHEISDPTPWILCSPEPLCGGKGCCAQEVCDCSGPVCTCPPCL | 0 |
| MPKLEMMLLVLLILPLCYIDAVGPPPPWNMEDEIIEHWQKLHCHEISDLTPWILCSPEPLCGGKGCCAQEVCDCSGPACTCPPCL | 0 |
| MPKLEMMLLVLLILPLCYIDAVGPPPPWNMEDEIIEHWQKLHCHEISDLTPWILCSPEPLCGGKGCCAQEVCDCSGPVCTCPPCL | 0 |
| MMCRLTSLCCLLVIVLLNSAVGGGIPCTGSGGFCSSYMWCCNSFDVCCEEFPGPARCMSESACSSIERWAQYTHFFRR | 0 |
| MSGHTSVNFLLLSIVALGMVATVICSCDSEFSSEFCEQPEERICSCSTHVCCHLSSRQKGSQCTTWNRCLSAQTGNRRSTHIDKKIPYVCHVILPTSLCDKRAD | 1 |
| MPKQEKMMLVLLILPLPYCNAAGVTTVQWGGHGDGLDRYLQRGVRDIHRPCQSVRPGRVWGKCCLTRLCSTMCCARADCTCVYHTWRGHGCSCVM | 1 |
| MKLLAPVLWAMAALGVTWLVAVDSKESCTKHSNGCSTPLRLPCQEYFRPACDIHDNCYHCGTIFGISRKECDDAFLKDMNTLCKKLGSNSATCPARGKREVTSHRATSIAHSRLWKTALDQKSFLNRKARQAILLTPNSCLYWANNFYMAVHVFGARSYSRTTDPKDCQGLKHCLPNH | 1 |
| CVFDCGTDCSGSCSCDNAPFDWCSCSGPTSKGCKCTCRCRNPTEC | 1 |
| LSLPNHDVKSATSSRSSSDYQGSSGDDCDEGLPPPDQPFRVVWNHPDNCERIKLHLPLDDYGIIFNKLRVFLGEEIQTLYDTGPWPYISETGKFINGGLPQSFNHPDNDGETQRILKKHRPENFTGLGVLDFETWRAIYSTNFGPMTIYQNESVKLVKEQHPDYDQKKLTKVAEKEWQQAAKDIMSNKLKIAQEVMPRGHWGYYLYPRTWDNKRDTKFRNDKINWLWRQSTGLYPSIYIYDFSKTESAITKFVSDTVGEAVRVQKEFSPPNTPIYPYVMFQTMDNIFHYEDHLKISLGLSAKMGAAGVVLWGTSKHYKESTRQWQCQQLQEHIRTVLGPLVKNVTQMMTDCSRAICEGHGRCVHNSHDVILGETESQRLSDLCSTRQSRFRDYHCRCYSAWEGACCQTLRPSRCQKREQRNVHGGGDLID | 1 |
| ECVFDCGTDCSGSCSCDNAPFDWCSCSGPTSKGCKCTCRCRNPTELFKYYSFYHCLDITYSKWF | 1 |
| CLSFCLLFTLPSSQREEDVQARKTHPKSDFYGTRPRSAERCTDSCEFQGVLCRGTCDCYGTNNCYCAINHNKQCICVCTN | 1 |
| MTRSVMQMGRLTLVLCLLLLLLLTTQACYIQNCLRVGKRHVETTHPCMSCSFGQCVGPRICCGPGGCEMGTAEANKCIAEDKNSTPCQVLGDHCDLNT | 0 |
| MAMNMSMTLSTFVMVVVAATVTGFTHLQVPSLSRMERDGPADCCDMKTCCVDKMRTCLQRHTFNEKNMVSSCYHAGDICGSYNEIVGCCYGYRTCMLRQLTSMRLQTAHAVCQHTDCFNP | 0 |
| TTRDCCIEATYQCLLNHHCQEHAHVTGCHWEAAGPCGVDPVEGCCYGYMYCMGMWMEACNIDPVHNECKNEYCYTP | 0 |
| MAMNMSMTLIVFVMVFILATVIDSTLLQEPDLSRVKRTTRDCCIEATYQCLLNHHCQEHAHVTGCHWEAAGPCGVDPVEGCCYGYMYCMGMWMEACNIDPVHNECKNEYCYTP | 0 |
| DGPADCCDMKTCCVDKMRTCLQRHTFNEKNMVSSCYHAGDICGSYNEIVGCCYGYRTCMLRQLTSMRLQTAHAVCQHTDCFNP | 1 |
| MKMLESALWILAALALPWIAAQDSRTDELCKINSNGCSVPSSWIPCQQHFLAACDRHDTCYMCGAHFSLTQKDCDDAFLVHMTALCGHGTDDEGFCLEKRKRREASSMSITTPLIQLRLLEKHMPLNSLWDHDSRQPQQRYIYDNCTDWALTYYNTVQRFGWWYFYDTANATYCPQFEPCMPEVSSSA | 0 |
| MKMLESALWILAALAMPWIAAQDSSTAELCKINSNGCSVPSSWIPCQQHFLAACDRHDTCYLCGAHFNLTQNDCDNAFLAHMIALCADGTDDEGFCLEKRKRREASSMSLTTPLRQLRLLEKLMPLNSLWDHDSRQPQQRYIYDNCTDWASTYYNTVQSFGSFYFYDTANATYCPQFEPCMPEVSSSA | 0 |
| MFVLLLLFTLASSQQEGDVQARKTSLKSDFYRALRKYNKRECVFDCGTDCSGSCSCDNAPFDWCSCSGPTSKGCKCTCRCRNPTEC | 1 |
| MKLLAPVLWAMAALGVTWLVAVDSKESCTKHSNGCSTPLRLPCQEYFRPACDIHDNCYHCGKTFGISREECDEAFLKDMNALCEKLGRDSATCPATRKRGVTSHRATSIAHSRLWKTALDQKSFLNRKARQSIQPPTSCQVWAQNYHWAVQLVGGSSYSGTTDLEACQGFEHCLPNH | 1 |
| MRAVVVVTGLVVVVVATALSLPNHDVKSATSSRSSSDYQGSSGDDCDEGLPPPDQPFRVVWNHPDNCERIKLHLPLDDYGIIFNKLRVFLGEEIQTLYDTGPWPYISETGKFINGGLPQSFNHPDNDGETQRILKKHRPENFTGLGVLDFETWRAIYSTNFGPMTIYQNESVKLVKEQHPDYDQKKLTKVAEKEWQQAAKDIMSNKLKIAQEVMPRGHWGYYLYPRTWDNKRDTKFRNDKINWLWRQSTGLYPSIYIYDFSKTESAITKFVSDTVGEAVRVQKEFSPPNTPIYPYVMFQTMDNIFHYEDHLKISLGLSAKMGAAGVVLWGTSKHYKESTRQWQCQQLQEHIRTVLGPLVKNVTQMMTDCSRAICEGHGRCVHNSHDVILGETESQRLSDLCSTRQSRFRDYHCRCYSAWEGACCQTLRPSRCQKREQRNVHGGGDLID | 1 |
| DVQARKTSLKSDFYRALRKYNKRECVFDCGTDCSGSCSCDNAPFDWCSCSGPTSKGCKCTCRCRNPTELFKYYSFYHCLDITYSKWF | 1 |
| MKLLAPVLWAMAALGVTWLVAVDSKESCTKHSNGCSTPLRLPCQEYFRPACDIHDNCYHCGTIFGISRKECDDAFLKDMNTLCKKLGSNSATCPARGKREVTSHRATSIAHSRLWKTALDQKSFLNRKARQVFFLLPTTCQGWAKNYHLAVKLAGANSYSVTTDLETCQGLEHCLPNH | 1 |
| MKMLESALWILAVLALPRIAAQDSRTTELCKINSNGCSVQFDWVPCQEHFLPACDIHDNCYFCGAHFSLSRLNCDDAFLSDMIALCADGTDEESDCPAKRKRREASSMSTTPVRQLQLLEKLMGRNSLSDHDPRRPLPRSSTCTGWAQTYYNFVRWFGASNYNETPDATYCSDYEECMPEV | 1 |
| MKMLASVLWTMAALGVTWLLAEDSTSEQSCKRFSNGCSTPLPLPCQEYFRPACDRHDSCYQCGAHFGINRKQCDDAFSDHMHALCDELGLLGMCPARRKRQVASGRATPIARSTLLKRALPQKSSLNREARVFFAPTFCHEWATTYYSVVRMAGAGLFFETIFDPADCQGLEACMPDH | 1 |
| MKLLAPVLWAMAALGVTWLVAVDSKESCTKHSNGCSTPLRLPCQEYFRPACDIHDNCYHCGKTFGISREECDKAFLKDMNTLCKKLGSNSATCPARGKREVTSHRATSIAHSRLWKTALDQKSFLNRKARQVFFLLPTTCQGWAKNYHLAVKLAGANSYSVTTDLETCQGLEHCLPNH | 1 |
| MKLLAPVLWAMAALGVTWLVAVDSKESCTKHSNGCSTPLRLPCQEYFRPACDIHDNCYHCGTIFRISRKECDDAFLKDMNTLCKKLGSNFATCPARGKREVTSHRATSIAHSRLWKTALDQKSFLNRKARQPILLTPNSCLYWANNFYMAVHVFGARSYSRTTDPKDCQGLKHCLPNH | 0 |
| MKVLESALWTLAALALPRIAAQDSSRAELCKINSNGCSVPFPDVPCQEHFRPACDIHDNCYLCGALFGFTQGKCDRAFLTHMTALCARGTDDEGFCLEKRKRREASSMSITTPLRQLRLLEKLLPPNSLSDRDPRQPHPRSATCTEWALMYFDAVQAMGWLYFYNTANTTYCPRFKTCMPEV | 1 |
| MKLLAPVLWAMAALGVTWLVAVDSKESCTKHSNGCSTPLRLPCQEYFRPACDIHDNCYHCGKTFGISREECDKAFLKDMNTLCKKLGSNFATCPARGKREVTSHRATSIAHSRLWKTALDQKSFLNRKARQVFFLLPTTCQGWAKNYHLAVKLAGANSYSVTTDLETCQGLEHCLPNH | 1 |
| MWMTISMFVVVVTAATVVGSTPLEERQPGDCCHLVFYECAAEACTFTDDPGCTEMCWDVATDVCGGDPDSGFCSSFFGCFNDCVFTESGPHFLCYELCKMVPCWLVK | 0 |
| MSRLPWAMCLMLLMLLLLGTAQGCFIRNCPRGGKRAVDSVQPTRQCMSCGPEGVGQCVGPSVCCGLGLGCLMGTPETEVCQKENESSVPCAISGRRCGMDNTGNCVADGI | 0 |
| DEPADCCNMKTCCVDKMSACLRRHPFNEINMASSCYQEANSKCGSYNEIVGCCYGYRICMLRHVGRMRLQRAHAVCQHTDCYNPCD | 1 |
| DGPADCCDMKTCCVDKMRTCLQRHTFNEKNMVSSCYHEAGDICGSYNEIVGCCYGYRTCMLRQLTSMRLQTAHAVCQHTDCFNPCD | 1 |
| DEPADCCDMKTCCVDKMSACLRRHPFNEINMASSCYQEANSKCGSYNEIVGCCYGYRICMLRHVGRMRLQRAHAVCQHTDCYNPCD | 1 |
| TPPPHNDCCKMKMCCAIKTEECLKTHSDQQHVYITICYQEASHTCGQYNEIVGCCYGYRNCMLINVQQLGLRQAQQTCSNRNCLNPCQ | 1 |
| DGPADCCDMKTCCVDKMRTCLQRHTFNEKNMVSSCYHEAGDICGSYNEIVGCCYGYRSCMLRQLTSMRLQTAHAVCQHTDCFNPCD | 1 |
| SGPADCCRMKECCTDRVNECLQRYSGREDKFVSFCYQEATVTCGSFNEIVGCCYGYQMCMIRVVKPNSLSGAHEACKTVSCGNPCA | 1 |
| MAMNMSMTLSTFVMVVVAATVTGFTHLQEPNLSRMERTPPPHNDCCKMKMCCAIKTEECLKTHSDQQHVYITICYQEASHTCGQYNEIVGCCYGYRNCMLINVQQLGLRQAQQTCSNRNCLNPCQ | 0 |
| MAMNMSMTLSTFLMVVVAATVIGFTHLQVPSLSRMERDEPADCCNMKTCCVDKMSACLRRHPFNEINMASSCYQEANSKCGSYNEIVGCCYGYRICMLRHVGRMRLQRAHAVCQHTDCYNPCD | 0 |
| MAMNMSMTLSTFVMVVVAATVTGFTHLQVPSLSRMERDGPADCCDMKTCCVDKMRTCLQRHTFNEKNMVSSCYHEAGDICGSYNEIVGCCYGYRSCMLRQLTSMRLQTAHAVCQHTDCFNPCD | 0 |
| QPGDCCHLVFYECAAEACTFTDDPGCTEMCWDVATDVCGGDPDSGFCSSFFGCFNDCVFTESGPHFLCYELCKMVPCWLV | 1 |
| MAMNMSMTLSTFVMVVVAATVTGFTHLQVPSLSRMERDGPADCCDMKTCCVDKMRTCLQRHTFNEKNMVSSCYHEAGDICGSYNEIVGCCYGYRTCMLRQLTSMRLQTAHAVCQHTDCFNPCD | 0 |
| NEPLDCCNMKTCCVRSMYECLQRHPGNENNMVSSCYHEAGDICGSYNEIVGCCYGYRTCILRHVNPMRIHRAHDVCKHTDCYSPCE | 1 |
| DEPLDCCEMKKCCVRSMYECLQDHRGNEYDMATECYQKAGDICGSYNEIVGCCQGYEICILKNVPHTGLQRAHDLCESTDCYNPCQ | 1 |
| MAMNMSVMLSAFVMVVVSATVTGFTHLQEPDLSRMERSPPPHNDCCKMKECCAQTTELCLKEFPNEEHIYTSTCYQRASHACGQFNEIVGCCYGYRQCMLQNVQNLGLNWANQQCKEWNCLNPCE | 0 |
| SPPPHNDCCKMKECCAQTTELCLKEFPNEEHIYTSTCYQRASHACGQFNEIVGCCYGYRQCMLQNVQNLGLNWANQQCKEWNCLNPCE | 1 |
| MAMSMSMTLSVFVMVVMAATVTGFTHLQVPNLSRMERDEPLDCCEMKKCCVRSMYECLQDHRGNEYDMATECYQKAGDICGSYNEIVGCCQGYEICILKNVPHTGLQRAHDLCESTDCYNPCQ | 0 |
| MAMNMSMTLSTFLMVVVAATVIGFTHLQVPSLSRMERDEPADCCDMKTCCVDKMSACLRRHPFNEINMASSCYQEANSKCGSYNEIVGCCYGYRICMLRHVGRMRLQRAHAVCQHTDCYNPCD | 1 |
| MAMSMSMTLSVFIMGVVAATVTGFTDLQVPNLSRMERNEPLDCCNMKTCCVRSMYECLQRHPGNENNMVSSCYHEAGDICGSYNEIVGCCYGYRTCILRHVNPMRIHRAHDVCKHTDCYSPCE | 0 |
| THPCMSCSFGQCVGPQICCGLGGCEMGTAEANKCIEEDDDQTPCQVLGDHCDLNNLDIEGHCVADGICCVDDTCAIHSSC | 0 |
| MNMRMTIVVFVVVATAATLVGSTPLEERQTVPSSCCVRKTVECAMYTCPEVPLSCTEECYKEKATPLCGGDPGDGCCPDFIRCYIDCLTNGPGQPLPCYQKCDNGSCLR | 0 |
| MAMNMWMTTSVFLVAVTATTVIGSTPSQEQERERRTDVDLCCATRIYYCLKDNGCLPQRECTVACDVPDDCANCCQAYLDCAMSCIWAYELPTGTEEEDPLRDCHNQCKDGC | 1 |
| QPTKPCMNCSFGQCVGPRVCCGAGRCEIGSTEADRCEEENEDPVPCKVLGQHCVLNNPGNVNGNCVDGGIGICCVDDTCAIHRRCD | 1 |
| MAMNMSMTLCMFVMVVVAATVIDSTQLQEPDLSRMRRSGPADCCRMKECCTDRVNECLQRYSGREDKFVSFCYQEATVTCGSFNEIVGCCYGYQMCMIRVVKPNSLSGAHEACKTVSCGNPCA | 1 |
| HPTKPCMYCSFGQCVGPHICCGPTGCEMGTAEANMCSEEDEDPIPCQVFGSDCALNNPDNIHGHCVADGICCVDDTCTTHLGCL | 0 |
| AVQPTRQCMSCGPGGVGQCVGPSVCCGLGLGCLMGTPETEVCQKENESSVPCAISGRRCGMDNTGNCVADGICCVEDACSFNSLCRVDTDQEDSVSARQELLTLI | 0 |
| RDVDDGLGVRPCMFCSFGQCVGPHICCGAGGCEIGTLEASTCHEENENPIPCHVFGDRCLLKHPGNVHGNCVSPGVCCTDDTCSMHVGCL | 0 |
| MTPIVFVTVVMAATVIGSTPLQEQELNRNDRDIQECCANKANECLRNDGCYQSQEESCAKRCYYTDTSSCGDQADVVCCFDYQYCMTECLFPHQDLYPEMLGDCYDYCKVYEQC | 1 |
| AEECSRAEALAKCYLPKDPGPCNARKPRYYFNRYLNTCQEFIYGGCHGNANRFYTMEDCLGCCLLSVCRQPAEPGLCNAYMERYYFDLDSYDCKPFIYGGCNGNDNKFHTYNECYGRCGLE | 1 |
| DVNCEGLAYHECCRNEMRICILECSSVWDADSCWDYCYSSAATNCECHPRDDCCPGFLESYSSCLFHDGLEEYECWEQARYVPCW | 1 |
| TDVDLCCATRIYYCLKDNGCLPQRECTVACDVPDDCANCCQAYLDCAMSCIWAYELPTGTEEEDPLRDCHNQCKDGC | 1 |
| DIQECCANKANECLRNDGCYQSQEESCAKRCYYTDTSSCGDQADVVCCFDYQYCMTECLFPHQDLYPEMLGDCYDYCKVYEQC | 1 |
| MGRRDSGRAVAQRYRGVTRGVTVIACLMVVCACVGLCDATGQKSILGSPGCHLVGHTRVVRIPDCVPFQITTNACRGFCVSYAIPSPYQTLVYNPNHIITSRAACCDIIDTLDIPVQVTCVDGVREIVFKSARSCACSICRRE | 0 |
| KCCAIALYQCLRDEGCLESGSSCQITCTFPHDCFEICCHDYMLCVYNCLHGREEGEDIMRVCHTACTDTVCSE | 0 |
| QTVPSSCCVRKTVECAMYTCPEVPLSCTEECYKEKATPLCGGDPGDGCCPDFIRCYIDCLTNGPGQPLPCYQKCDNGSCL | 1 |
| MNMRTTISVLAVAVMATTVTASPLLQDQERDTDDRKCCAIALYQCLRDEGCLESGSSCQITCTFPHDCFEICCHDYMLCVYNCLHGREEGEDIMRVCHTACTDTVCSE | 0 |
| MGRRDSGRAVAQRYRGVTRGVTVIACLMVVCACVGLCDATGQKRHSWEAPGCHLVGHTRVVRIPDCVPFQITTNACRGFCVSYAIPSPYQTLVYNPNHIITSRAACCDIIDTLDIPVQVTCVDGVREIVFKSARSCACSICRRE | 0 |
| MTMNMSLTLSLFVMVVIAATVVGSTPLEGKDVNCEGLAYHECCRNEMRICILECSSVWDADSCWDYCYSSAATNCECHPRDDCCPGFLESYSSCLFHDGLEEYECWEQARYVPCW | 0 |
| EAMRPCKYCSFGQCVGPQICCGDRGCEMGSEEANKCREEDEDSTPCQVFGWPCTLNNPGNTNGKCVANCIGICCVTDTCVVSSECQ | 1 |
| MEGHRFAAVLILTICMLALGAGAFRLHGSRAEECSRAEALAKCYLPKDPGPCNARKPRYYFNRYLNTCQEFIYGGCHGNANRFYTMEDCLGCCLLSVCRQPAEPGLCNAYMERYYFDLDSYDCKPFIYGGCNGNDNKFHTYNECYGRCGLE | 1 |
| MTMPVTLSLLVTIAMAATVLDSTLLRGNDPSCEDQVYHECCRHEMHSCAEECLSASDMDFCWEPCTQSAAVQCGRQTSSACCRPFLECFSACLNDGDVLYDCWIRCRHVPC | 0 |
| GNDPSCEDQVYHECCRHEMHSCAEECLSASDMDFCWEPCTQSAAVQCGRQTSSACCRPFLECFSACLNDGDVLYDCWIRCRHVPC | 1 |
| IPCHGPGGWCSTHMWCCDPHNVCCDFPGAARCTRKRECSWPTIPQGRLAQLSHFFIPCHGPGGWCSTHMWCCDPHNVCCDFPGAARCTRKRECSWPTIPQGRLAQLSHFF | 0 |
| LLLLLLLTTQACYIRNCPGGGKRDVEDRNPTKLCMHCNFGQCVGPNICCGARGCEMGTAEAKKCRLEDDDPTPCRVIGEHCVMNNPGNRNGNCVGGGIGICCVDDTCATHTGCQ | 1 |
| MPMGRLTLVLYLLLLLLLTTQACFIRNCPKGGKRDVEEDRALMKPCMFCNSGQCVGPQMCCGEAGCHMGTAEANRCAEEDNDPTPCRVFGDHCIMNTPGNIHGNCVGNGIGICCVDDACSIHPGCL | 0 |
| ACFIRNCPKGGKRNVDEGPTKPCMFCSFGQCVAPHTCCGEKGCEMGTVDANMCQEENESPIPCHVFGKRCLLNHPGNSHGNCVTYGICCSHDTCTVHLACM | 0 |
| MTRSAMQMGRLTLVLCLLLLLLLTTQACFITNCPVGGKRHVEATHPCMSCSFGQCVGPQICCGLGGCEMGTAEANKCIEEDDDQTPCQVLGDHCDLNNLDIEGHCVADGICCVDDTCAIHSSC | 0 |
| MGRLTMALCWLLLLLLTTQACYITNCPRGGKRDVDDGLGVRPCMFCSFGQCVGPHICCGAGGCEIGTLEASTCHEENENPIPCHVFGDRCLLKHPGNVHGNCVSPGVCCTDDTCSMHVGCL | 0 |
| MGRLTLVLCLLLLLLLTTQACYIQNCLRVGKRHVETTHPCMSCSFGQCVGPRICCGPGGCEMGTAEANKCIAEDKNSTPCQVLGDHCDLNNLDNIEGHCVADGICCVEDTCAIHSSC | 0 |
| MTRSVMQMGRLTLVLCLLLLLLLTTQACYIQNCLRVGKRHVETTHPCMSCSFGQCVGPRICCGPGGCEMGTAEANKCIAEDKNSTPCQVLGDHCDLNNLDNIEGHCVADGICCVEDTCAIHSSC | 0 |
| MQMGRLTLVLCLLLLLLLTTQACYIQNCLRVGKRHVETTHPCMSCSFGQCVGPRICCGPGGCEMGTAEANKCIAEDKNSTPCQVLGDHCDLNNLDNIEGHCVADGICCVEDTCAIHSSC | 0 |
| MQMGRPTLLPCLLLLLVLSTQACFIRNCPEGGKRDVHMIQPTKPCMNCSFGQCVGPRVCCGAGRCEIGSTEADRCEEENEDPVPCKVLGQHCVLNNPGNVNGNCVDGGIGICCVDDTCAIHRRCD | 0 |
| MQMGRLTLVLCLLLLLLLTTQGCFIRNCPKGGKRDVDERQATKACMSCSFGQCVGPQICCGAGGCEMGTVEAKRCSEEDEDPIPCQVIGNHCALDNPGHCAAYGICCVDDTCTTHSGCL | 0 |
| MQMGRPTLLPCLLLLLVLSTQACFIRNCPKGGKRNVDEGPTKPCMFCSFGQCVGPNICCGARGCEMGTTEANRCIEEDEDPIPCWVFGDHCILNNRGNIHGNCVANGICCGDDTCTTHWGCL | 0 |
| MQMGRLTLVLCLLLLLLLTTQGCFIRNCPVGGKRDVDERQPVKACTYCSFGQCVGPHICCGAGGCEMGTAEANKCSEEDEDTIPCQVTGNPCTLNNPGNIQGHCVAYGICCVDNTCTTHSGCL | 0 |
| MQMGRLTLVLCLLLLLLLTTQACFIRNCPKGGKRDVDERQLVNACMFCSFGQCVGPHICCGDGGCQMGTPEASRCSQEDESPIPCQVIGNHCALNNPGNIHGHCVAYGICCVDDTCTTHSGCL | 0 |
| MKCSVLQMSRLSWAMCLMLLMLLLLGTAQGCFIRNCPRGGKRAVDALQPTRQCMSCGPEGVGQCVGPSVCCGLGLGCLMGTPETEVCQKENESSVPCAISGRRCGMDNTGNCVADGICCVEDACSFNSLCRVDTDQEDSVSARQELLTLIRRLLVNRQYD | 0 |
| MKCSVLQMSRLSWAMCLMLLMLLLLGTAQGCFIRNCPRGGKRAVDALQPTRQCMSCGPDGVGQCVGPSVCCGLGLGCLMGTPETEVCQKENESSVPCAISGRHCGMDNTGNCVADGICCVEDACSFNSLCRVDTDQEDSVSARQELLTLIRRLLVNRQYD | 0 |
| MKCSVLQMSRLSWAMCLMLLMLLLLGTAQGCFIRNCPRGGKRAVDAVQPTRQCMSCGPGGVGQCVGPSVCCGLGLGCLMGTPETEVCQKENESSVPCAISGRRCGMDNTGNCVADGICCVEDACSFNSLCRVDTDQEDSVSARQELLTLIRRLLVNRQYD | 0 |
| MKCSVLQMSRLSWAMCLMLLMVLLLGTAHGCFIRNCPRGGKRAVDAVQPTRQCMPCGPDGMGQCVGPSVCCGLGLGCLMGTPETEVCQKENESAVPCAISGRHCGMDNTGNCVADGICCVEDACSFNSLCRVDTDQEDSVSARQELLTLIRRLLVNRQYD | 0 |
| MTRSALQMGRLTLVLCLLLQLVLVTQACFLGNCLNDGERDVDGREAMRPCKYCSFGQCVGPQICCGDRGCEMGSEEANKCREEDEDSTPCQVFGWPCTLNNPGNTNGKCVANCIGICCVTDTCVVSSECQKESKSGIRVGCQRS | 1 |
| MTCFRSAVYPLLLTATVIVVATGNRCRLEPDAGLCRAAIDQFYYNWNERQCQTFIYGGCGGNDNRFGTREECEQACTVPSRNRDCNWPKETGPCRARFPSFFYDLNTGKCQDFIYGGCGGNANRFDTLDDCEQRCAVVCQEPKQVGLCRAAFRRWFYNRNSMMCERFIYGGCGGNRNNFQTFSECRDQCMLK | 1 |
| GDTSAECCLTLLGCYNTCMLDHISSDCWEHCKSENTIQCSLDFSLYYCNSFQSCYQICIRTKHESHCFRGCKYEAMGDCLETGVVECCPGFMMCYNNCEHSHAYCMTHCTKGSC | 0 |
| MNMRMMLTVLIVVVFTRMALCSTEDGRLARQRRGDTSAECCLTLLGCYNTCMLDHISSDCWEHCKSENTIQCSLDFSLYYCNSFQSCYQICIRTKHESHCFRGCKYEAMGDCLETGVVECCPGFMMCYNNCEHSHAYCMTHCTKGSC | 0 |
