# Supplementary material for: Cysteine pattern barcoding-based dataset filtration enhances the machine learning-assisted interpretation of Conus venom peptide therapeutics
Source: PLoS One. 2025 Jul 11;20(7):e0327578. doi: 10.1371/journal.pone.0327578 (PMC12250603; doi:10.1371/journal.pone.0327578)
Supplement: S5 Table — Common interactions are indicated by red and green colors, while black color indicates residues that were not involved in common interactions. Red color residues represent the Cav2.2 channel, while green color depicts Conus magus and Conus striatus peptides, respectively.: indicates the interaction between the Cav2.2 channel and peptide residues. (DOCX) [file pone.0327578.s006.docx]

Table S5. Residues from *Conus magus* and *Conus striatus* peptides involved in interaction against Cav2.2 channel. Common interactions are indicated by bold and Italic residues, while other simple black residues were not involved in common interactions. Residues in bold represent the Cav2.2 channel, while Italic residues depicts *Conus magus* and *Conus striatus* peptides, respectively. : indicates the interaction between the Cav2.2 channel and peptide residues.

| **Known peptide** | **Novel peptide** |
| --- | --- |
| **Glu1659 :** *Ser19* | **Glu1659 :** *Ser19* |
| **Met1369 :** *Tyr13* | **Met1369 :** *Tyr13* |
| **Tyr1310 :** *Thr17* | **Tyr1310 :** *Thr17* |
| **Met1369 :** *Gly18* | **Met1369 :** *Gly18* |
| **Thr643 :** *Leu11* | **Thr643 :** *Leu11* |
| **Pro642 :** *Leu11* | **Pro642 :** *Leu11* |
| **His671 :** *Leu11* | **His671 :** *Leu11* |
| **Ala667 :** *Leu11* | **Ala667 :** *Leu11* |
| **Asp1629 :** *Cys25* | **Asp1629 :** *Cys25* |
| **Gln638 :** *Ser9* | **Gln638 :** *Ser9* |
| **Asp1629 :** *Gly5* | **Asp1629 :** *Gly5* |
| **Asp265 :** *Ser22* | **Asp265 :** *Ser22* |
| **Lys1372 :** *Tyr13* | **Lys1372 :** *Tyr13* |
| **Met1369 :** *Gly18* | **Met1369 :** *Gly18* |
| **Tyr1310 :** *Thr17* | **Tyr1310 :** *Thr17* |
| **Tyr1310 :** *Cys16* | **Tyr1310 :** *Cys16* |
| **Asp664 :** *Arg10* | **Asp664 :** *Arg10* |
| **Cys1311 :** *Arg10* | **Cys1311 :** *Arg10* |
| **Tyr1310 :** *Cys1* | **Tyr1310 :** *Cys1* |
| Thr644 : Lue11 | Tyr1344 : Cys16 |
| Glu640 : Met12 | Lys1331 : Lys6 |
| Lys1372 : Met12 | Lys1372 : Asn14 |
| Lys1372 : Tyr13 | Tyr1344 : Asn14 |
| Pro1368 : Tyr13 | Lys1342 : Cys1 |
| Gln658 : Tyr13 | His1373 : Tyr13 |
| Asp664 : Arg10 | Asn322 : Arg21 |
| Glu1330 : Tyr13 | Glu1330 : Lys6 |
| Glu1330 : Lys2 | Glu1330 : Pro7 |
| Met1369 : Gly18 | Tyr1310 : Cys16 |
| Asp1627 : Lys4 | Asp1629 : Lys24 |
| Asp1628 : Arg21 | Asp318 : Arg10 |
